# Transcriptomic analysis of aerobic respiratory and anaerobic photosynthetic states in *Rhodobacter capsulatus* and their modulation by global redox regulators RegA, FnrL and CrtJ

**DOI:** 10.1099/mgen.0.000125

**Published:** 2017-07-08

**Authors:** Joseph E. Kumka, Heidi Schindel, Mingxu Fang, Sebastien Zappa, Carl E. Bauer

**Affiliations:** ^1^​ Molecular and Cellular Biochemistry, Indiana University, Bloomington, USA; ^2^​ Biochemistry, Indiana University Bloomington, Simon Hall MSB, 212 S Hawthorne Dr, Bloomington, IN 47405-7003, USA

**Keywords:** transcriptomics, redox regulation, RegA, FnrL, CrtJ

## Abstract

Anoxygenicphotosynthetic prokaryotes have simplified photosystems that represent ancient lineages that predate the more complex oxygen evolving photosystems present in cyanobacteria and chloroplasts. These organisms thrive under illuminated anaerobic photosynthetic conditions, but also have the ability to grow under dark aerobic respiratory conditions. This study provides a detailed snapshot of transcription ground states of both dark aerobic and anaerobic photosynthetic growth modes in the purple photosynthetic bacterium *Rhodobactercapsulatus*. Using 18 biological replicates for aerobic and photosynthetic states, we observed that 1834 genes (53 % of the genome) exhibited altered expression between aerobic and anaerobic growth. In comparison with aerobically grown cells, photosynthetically grown anaerobic cells showed decreased transcription of genes for cobalamin biosynthesis (−45 %), iron transport and homeostasis (−42 %), motility (−32 %), and glycolysis (−34 %). Conversely and more intuitively, the expression of genes involved in carbon fixation (547 %), bacteriochlorophyll biosynthesis (162 %) and carotenogenesis (114 %) were induced. We also analysed the relative contributions of known global redox transcription factors RegA, FnrL and CrtJ in regulating aerobic and anaerobic growth. Approximately 50 % of differentially expressed genes (913 of 1834) were affected by a deletion of RegA, while 33 % (598 out of 1834) were affected by FnrL, and just 7 % (136 out of 1834) by CrtJ. Numerous genes were also shown to be controlled by more than one redox responding regulator.

## Abbreviations

CBB, Calvin–Benson–Bassham; COG, cluster of orthologous groups; DEG, differentially expressed gene; DMA-PP, dimethylallyl diphosphate ; IPP, isopentenyl diphosphate; LH, light harvesting; PCA, principle component analysis; qRT-PCR, quantitative reverse transcriptase PCR; RC, reaction center; RNA-seq, RNA sequencing; TMAO, trimethylamine N-oxide.

## Data Summary

Raw sequence data from these RNA-seq studies can be accessed via the National Center for Biotechnology Information Sequence Read Archive; accession number: PRJNA357604 (url – https://www.ncbi.nlm.nih.gov/bioproject/?term=PRJNA357604).

## Impact Statement

Since the 1950s, it has been well established that anoxygenic photosynthetic bacteria extensively regulate synthesis of their photosystem and metabolism in response to the presence or absence of oxygen. Anoxygenic photosynthetic bacteria are of particular significance as they house a simplified photosystem that evolutionarily predates that of the oxygen evolving photosystems present in cyanobacteria and chloroplasts. Indeed, the anoxygenic photosystem predates the presence of oxygen on Earth, so the regulation of photosynthesis in response to the presence of oxygen represents a more modern control event that is linked to the oxidation conditions of our planet. This study provides a detailed analysis of global transcription changes that occur when a photosynthetic cell transitions from anaerobic to aerobic photosynthetic growth conditions. These results highlight massive transcription changes that allow these cells to thrive under these very different growth conditions and also identify the relative contributions of the redox responding regulatory control mechanisms that control the transition from anoxygenic to oxygenic environments.

## Introduction

Purple anoxygenic photosynthetic bacteria are among the most diverse micro-organisms studied in regard to their ability to generate metabolic energy. This includes aerobic and anaerobic respiratory, chemoautotrophic, photoautotrophic and photoheterotrophic growth modes [1]. This growth versatility should promote *Rhodobacter* species as attractive organisms for the production of useful chemicals; unfortunately, little is known about the transcription changes that provide control in driving carbon metabolism or transcriptional allocation under different growth conditions. For example, it is not known what percentage of the *Rhodobacter capsulatus* transcriptome is dedicated to biosynthesis of haem, cobalamin and bacteriochlorophyll under anaerobic photosynthetic conditions, or to homeostasis of iron and other metals under aerobic respiratory conditions. Understanding regulatory changes in gene expression can provide a foundation to the metabolic changes that allow this genus to thrive under so many different environmental conditions. This type of analysis can also provide an insight to how these cells can use transcription to control the flux of metabolic pathways that can lead to downstream applications in the production of useful chemicals such as hydrogen (H_2_) and polyhydroxybutyrate for renewable biofuel and biodegradable plastics, respectively [2–5].

One way to obtain a global snapshot of the number of transcripts dedicated to different metabolic pathways involves the use of RNA-sequencing (RNA-seq). This technique can provide genome-wide transcriptome profiles that reveal the range of expression of individual genes and collectively yield an immense amount of information on the activity of metabolic pathways. There are examples in the literature that bid to prescribe the global transcriptomic picture to a single photosynthetic bacterial species as it pertains to unperturbed photosynthetic and aerobic growth states [6–10]. However, many of these studies are focused on a single growth state and often use a low number of biological replicates, which limits their ability to detect small, yet significant, changes in gene expression. As with all transcriptomic methods, RNA-seq does come with limitations, as this technique can generate false positives and false negatives. This problem can be minimized through the use of a large number of biological replicates, although it should be noted that while a larger replicate size does minimize false discovery, it is not entirely eliminated [11]. In the course of our transcriptome studies on redox regulators RegA, FnrL and CrtJ, we have obtained 18 biologically independent RNA-seq data sets for *R. capsulatus* grown under dark aerobic and illuminated anaerobic photosynthetic conditions [12–14]. Collective analysis of these data sets provides a detailed and robust snapshot of the *R. capsulatus* global transcriptome during growth under dark aerobic respiratory versus illuminated anaerobic photosynthetic conditions. We have also determined the relative contributions of the well-characterized redox-responding transcription factors RegA, FnrL and CrtJ in the regulation of the metabolic physiology that occurs under these different growth conditions.

## Methods

### Strains, media, growth conditions and RNA extraction

The *R. capsulatus* parental strain SB1003 and the Δ*regA,* Δ *fnrL and* Δ *crtJ* clean deletion derivatives have been previously described [12, 14–16]. These strains were routinely grown in 3 g peptone l^−1^, 3 g yeast extract l^−1^ (PY) liquid broth or on agar plates, with liquid media supplemented with 2 mM MgCl_2_ and 2 mM MgSO_4_. Dark aerobic cultures were grown as follows: a dark aerobic overnight culture in PY medium was subcultured by dilution to an optical density at 660 nm of 0.03 into 5 ml PY medium in a 50 ml flask shaken at 200 r.p.m. In the case of photosynthetically grown cells, photosynthetic overnight starter cultures were grown as anaerobic cultures in 18 ml filled screw-capped tubes that were illuminated with a bank of 75 W tungsten filament light bulbs at an intensity of ∼30 µmol m^−2^ s^−1^. These cells were then subcultured by dilution into fresh PY medium to an optical density at 660 nm of 0.03 and grown anaerobically in screw-capped tubes with similar illumination. Both dark aerobic and anaerobic photosynthetically grown cells were stopped at the optical density of 0.3 in an ice/water bath, and the cells transferred into 2 ml Eppendorf tubes and centrifuged at 6000 r.p.m. for 3 min at 4 °C. The entire 2 ml cell pellet was then used for extracting total RNA using a Bioline Isolate II RNA extraction kit. Briefly, the bacterial pellet was dissolved in 100 µl TE (10 mM Tris-HCl, 1 mM EDTA, pH 8) buffer containing 10 mg lysozyme ml^−1^ and incubated for 3 min at room temperature. After isolation of total RNA, the DNA was removed by the addition of 1 unit Turbo DNAse and further incubated for 30 min at 37 °C. A clean-up step was performed with a Zymogen Direct-zol RNA extraction kit or RNeasy MinElute Cleanup kit according to the manufacturers' instructions. To check for residual DNA, quantitative reverse transcriptase PCR (qRT-PCR) of the *rpoZ* gene was performed with and without reverse transcriptase.

### RNA-sequencing library preparation

Total RNA was submitted to the University of Wisconsin-Madison Biotechnology Center (Madison, WI, USA), where it was verified for purity and integrity with a NanoDrop2000 spectrophotometer and an Agilent 2100 BioAnalyzer, respectively, and converted into sequence libraries. Samples that met Illumina sample input guidelines were prepared according the TruSeq Stranded Total RNA Sample Preparation Guide (15031048 E) using the TruSeq Stranded Total RNA kit (Illumina) with minor modifications, using 2 µg total RNA for each library preparation.

### Data processing, computer software and data analysis for RNA-sequencing

All computations were performed on a custom-built computer running Ubuntu 13.10 equipped with an Asus Z9PE-D8 WS motherboard, 2× Intel Xeon E5-2630 V2 CPU and 128 GB DDR3-1600 RAM. Each FastQ file was checked for quality using FastQC and further trimmed using the Trimmomatic program with a sliding window of 5 : 25 and a minimum length of 40 bases. The reads were aligned using the Bowtie2 program and final raw gene counts were generated using the HTSeq-count program. Raw counts generated from the HTSeq-count program were used to generate differentially expressed genes (DEGs) with the DESeq2 package in R. DESeq2-normalized sequencing counts were used to determine relative transcript levels of individual genes in the chromosome. Raw data can be accessed via the National Center for Biotechnology Information Sequence Read Archive server under the accession number PRJNA357604.

### qRT-PCR validation of RNA-seq differential expression

We validated the RNA-seq data by performing qRT-PCR on two sets of 14 genes. One set of validation genes were chosen that had a wide range of positive and negative fold-changes. A second set of genes were used that did not show statistically significant differential expression in the RNA-seq data set. Total RNA was isolated from three biological replicates as described above. qRT-PCR was used to determine gene expression levels using the SensiFAST SYBR Hi-ROX One-Step kit (Bioline), according to the manufacturer’s instructions using 1 ng RNA per 20 µl reaction. The reactions were performed on a StepOnePlus Real-Time PCR system (Life Technologies). Fold-changes and statistical analysis of gene expression were calculated using the rest 2009 program [17]. Primers were designed with the Primer3 web server and are listed in Table S1 (available with the online Supplementary Material) [18]. Target gene expression was normalized using expression of *rpoZ* as the reference gene [18].

## Results and Discussion

### Technical overview of differential expression between anaerobic photosynthetic and dark aerobically grown cells

In this study, we analysed genome-wide changes in mRNA expression by performing an RNA-seq analysis of 18 independent RNA preparations derived from dark aerobically grown cells and 18 independent replicates from anaerobic photosynthetically grown cells. Both of these conditions involved cells grown in similar complex peptone yeast extract growth medium.

We analysed reproducibility of independent replicates by performing principle component analysis (PCA), which showed that photosynthetic and aerobic samples form distinct clusters that do not overlap (Fig. S1). Additional pairwise comparison of individual data sets was undertaken by analysing Pearson correlation coefficients, which showed that all 18 aerobic and photosynthetic replicates exhibited a high degree of correlation within each data set, with *r* coefficients ranging from 0.80 to 1.0 (Table S2). The Pearson correlations, along with PCA clustering, showed that the measured aerobic and anaerobic expression levels formed two distinct transcriptional states that were highly reproducible. In addition, RNA-seq data sets were validated using quantitative PCR, which showed excellent correlation with the RNA-seq data sets (*R* ^2^=0.95) (Fig. S2). Data with the lowest fold-difference detected by qRT-PCR were 1.8 for *hemC* and 1.7 for *feoB2* (Table S3). Genes that did not show statistically significant qRT-PCR fold-changes (as determined using the relative expression software tool rest 2009) were *hemB* and *cycY*, both of which had fold-changes far below 2 (Table S3).

Statistically significant changes in expression were called using DESeq2 on Benjamini–Hochberg adjusted *P* values. A *P* value ≤0.05 was used as a cut-off, with the results reported as log2 fold-changes in expression as defined as anaerobic photosynthetic expression divided by dark aerobic expression [10, 19]. Overall, this analysis is indicative that 942 genes underwent an increase in expression and 900 a decrease in expression in photosynthetic conditions relative to the level observed in anaerobic conditions. Using a more stringent *P* value of ≤0.01 reduced the number of DEGs to 765 that showed an increase and 755 genes with a decrease in expression, respectively (Fig. S3).

### Transcript expression levels show that photosynthesis genes are some of the highest expressed genes during both photosynthesis and aerobiosis

**Fig. 1.**
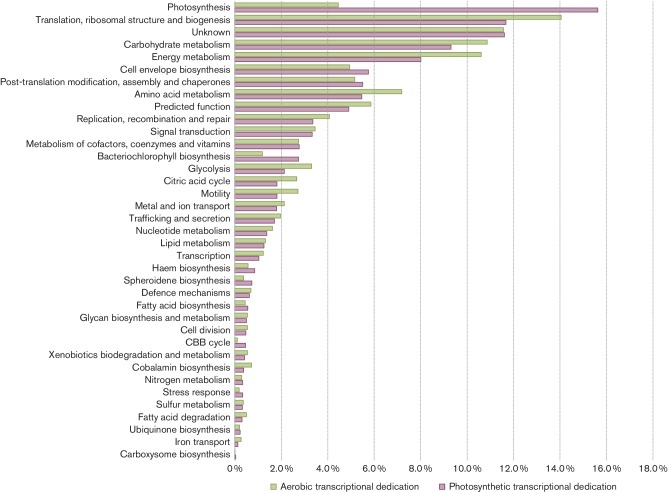
Cellular transcription dedication levels. Percentage of read counts per pathway as compared to the global total read counts obtained from aerobic respiration (green) and anaerobic photosynthetic (purple) samples.

To help address the functional roles of genes and gene families, we catalogued genes into ‘clusters of orthologous groups’ (COG) categories. Analysis of expression levels in individual COG categories showed that photosynthesis genes, not surprisingly, were some of the highest-expressed genes observed during the photosynthetic growth state (Fig. 1, Table S4). Under aerobic conditions, the highest expressed COG categories were genes involved in cell envelope biosynthesis, translation, motility, followed by carbohydrate and energy metabolism (Fig. 1, Table S4). Genes that encoded light harvesting and reaction centre structural proteins of the photosystem, *puc*, *puf* and *puh* [20, 21], collectively comprised 12 % of all photosynthetic transcripts (sequencing reads) and 2.8 % of all aerobic transcripts (Table S4). The light harvesting I and II structural proteins encoded by the *pufB*, *pufA, pucB* and *pucA* genes [20, 21] were the highest expressed photosystem genes, collectively comprising 7.7 % of all sequencing reads under photosynthetic conditions. These were followed by the reaction centre structural transcripts encoded by the *pufL*, *pufM* and *puhA* genes that collectively comprised 2.8 % of all sequencing reads. Interestingly, transcription of some photosynthesis genes, such as *crt* and *bch* genes, was not completely turned off under aerobic conditions and the genes were in fact some of the higher expressed genes as compared to other COG categories (Fig. 1). One interpretation is that a high basal level of *bch* and *crt* expression allows these organisms to undertake rapid synthesis of the photosystem upon a shift from aerobic to anaerobic conditions.

Although the photosynthetic COG category dominated the transcription profile, we observed that the single highest transcribed gene under both photosynthetic and aerobic conditions was the porin family protein-encoding gene *ompU* (rcc00259) with 4.2 and 3 % of all sequencing reads dedicated for photosynthetic and aerobic growths, respectively (Table S4). This is an interesting response, given that it has been observed that photosynthetically grown *R. capsulatus* cells excrete large amounts of outer-membrane vesicles containing haem and bacteriochlorophyll biosynthesis intermediates bound to this porin [22, 23]. Similar tetrapyrrole/membrane protein complexes have also been reported for *Rhodobacter sphaeroides* and *Rhodospirillum rubrum* [24–28].

**Fig. 2.**
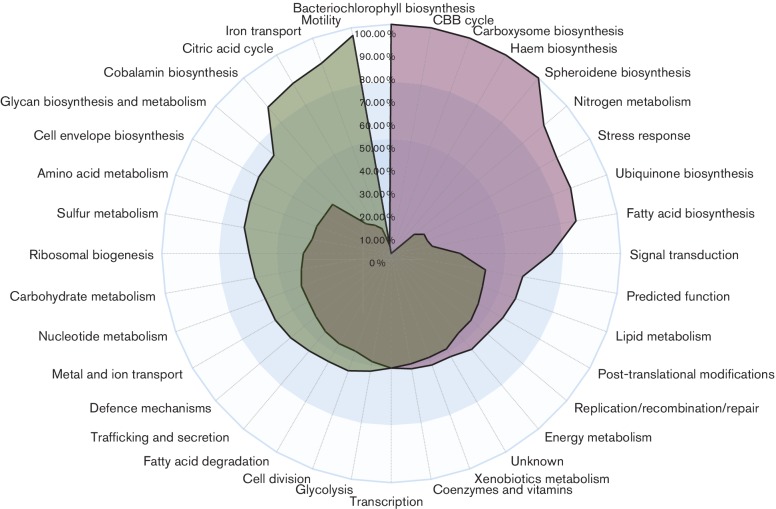
Pathway power-balance chart. This shows the percentage of genes in each subgrouping that were positively differentially expressed during photosynthesis (purple) and during aerobiosis (green). The outer ring constitutes 100 %, the middle circle (light blue) 75 % and the inner circle (dark blue) 50 % of the genes in each category that undergo an increase in expression under photosynthetic (purple) or aerobic (green) growth conditions.

To assess how individual metabolic pathways were behaving within a given growth state, the total percentage of DEGs for a specific pathway was accessed as a ratio of the number of genes that were up-regulated to the total number of genes within the pathway. Based on this method of analysis, it was observed that pathways involved in haem, bacteriochlorophyll and carotenoid (spheroidene) biosynthesis [29, 30] were 100 % induced with respect to the photosynthetic state (Fig. 2). Expression of the light harvesting and reaction centre structural genes (photosynthesis category) was also systematically induced under photosynthetic conditions. The observation of a concomitant increase in expression of photosystem genes (*bch, crt, puf, puh* and *puc* genes) in anaerobic photosynthetically grown cells confirms previous data with β-galactosidase reporter plasmids that showed similar levels of expression increases [15, 31–33]. Also notable were increases in photosynthesis gene expression of a large number of genes involved in ubiquinone and fatty acid biosynthesis. This may be expected, as these cells anaerobically induce the synthesis of intracytoplasmic membrane vesicles that house ubiquinone and components of the photosystem. Another notable change in anaerobic photosynthetic metabolism was a reduction in the expression of most genes involved in the citric acid cycle.

### Redox and oxidative stress mitigation during photosynthesis

**Fig. 3.**
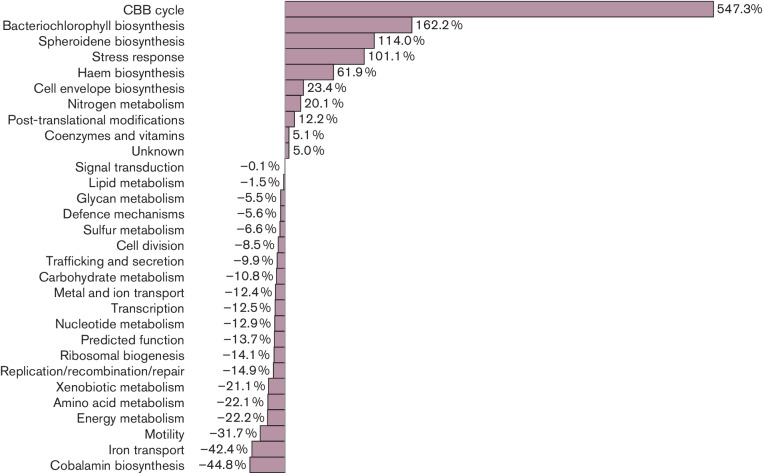
Global transcription changes of various COG categories during aerobic photosynthesis. Per cent change is defined to be the total number of counts during photosynthesis as compared to that of aerobiosis. These changes reversed for the aerobic growth mode. Genes in each COG category are listed in Tables S2–S5

The Calvin–Benson–Bassham (CBB) carbon fixation genes showed the highest increase in expression from aerobic to anaerobic photosynthetic conditions (Fig. 3, Table S5). This increase was occurring in rich growth medium where carbon was in excess. Presumably this was in response to a role as an electron sink [34]. In a similar vein, nitrogen fixation genes were also largely activated in photosynthetic conditions where nitrogen was also in excess. Like for carbon fixation, nitrogenase is also capable of functioning as an electron sink through the production of H_2_ via a side reaction (2H^+^+2 e^-^ →H_2_) [35, 36].

Interestingly, the group with the fourth highest level of gene induction during photosynthesis was the ‘stress response’ genes (Fig. 3, Table S5) that include chaperones that refold proteins such as Hsp70, HspQ, Hsp15, DnaJ etc. One explanation for why the expression of these proteins significantly increases during photosynthetic growth is that stress response proteins could be mitigating photosynthesis-driven photo-oxidative damage [37, 38].

### Iron-transport genes are primarily expressed during aerobiosis

Iron transport is important for the maintenance of the global iron supply for biosynthesis of cofactors such as haem and enzymes that utilize iron in their active sites. Free intracellular iron is also toxic, so its transport is highly regulated. Analysis of the expression of iron-transport genes for ferrous and ferric iron, hemin and siderophores showed that these iron-transport genes were expressed at significantly higher levels aerobically then during photosynthetic growth conditions (Figs 3 and S4, Table S5). During aerobic respiration, the total transcript level of iron-transport genes was 0.27 % of all transcripts compared to 0.13 % photosynthetically, which signals a twofold difference between these two states (Fig. 1, Table S5).

Under photosynthetic anaerobic growth, iron is predominantly in a reduced (Fe^2+^) ferrous state, which is very soluble. In iron-limiting conditions, ferrous iron is transported either by the Feo iron-transport system or by the EfeUOB elemental ferrous iron system [39, 40]. These ferrous iron transporters are typically induced only under conditions of iron starvation. In our nutrient rich growth conditions, the expression of *efe* ferrous iron-transport genes was actually decreased twofold- to fourfold relative to aerobic growth, which suggests that ferrous iron was plentiful in this rich growth medium (Fig. S4, Table S5). Expression of other genes belonging to iron homeostasis was down 42 % during photosynthesis (Fig. 3). Notable exceptions were a member of the FeoA family (*rcc02028*) and a TonB-dependent receptor proposed to be involved in siderophore transport (*rcc01049*), which were increased approximately fourfold during photosynthesis (Fig. S4, Table S5). Their actual role in iron transport (if any) will need to be experimentally verified.

Under aerobic conditions, iron is in the oxidized ferric (Fe^3+^) state, which is largely insoluble (molar solubility <10^−18^ M at pH 7; [41]), requiring a siderophore uptake system that has a high affinity for Fe^3+^ (*K*_d_ <10^−20^ M; [42, 43]). In Gram-negative species, siderophore transport is accomplished by a TonB-dependent ferric (Fe^3+^) siderophore receptor that transports ferrisiderophores through the outer membrane, followed by transport through the cytoplasmic membrane via an ABC transporter [44, 45]. Expression of the *fep* operon (*rcc01442* and *rcc01434*), which encodes for proteins involved in ferric (Fe^3+^) siderophore transport, exhibited a preference for aerobiosis (Fig. S4, Table S5). The *exbB–exbD–tonB* operon encoding for the outer-membrane ferric (Fe^3+^) siderophore receptor also showed an increased aerobic expression relative to photosynthesis (Fig. S4, Table S5). The Fe^3+^ iron ABC transporter encoded by *rcc02578* was expressed more than 20-fold higher during aerobic growth (Fig. S4, Table S5). Within the *fep* operon are two genes that are annotated as ABC transporters (*rcc01439*-*rcc01440*). Based on sequence analysis, these genes show a good degree of identity (>60 %) to the YbtP and YbtQ iron ABC transporters from *Rubrivivax gelatinosus* and its homologs in *Yersinia pestis* and *Pseudomonas aeruginosa* [46, 47]. These cytoplasmic transporters were increased in *R. capsulatus* in aerobic conditions (Table S5). An additional ferric transport system encoded by the *R. capsulatus* genome is the *fhu* ferrichrome operon that also had a threefold increased in aerobic expression (Fig. S4, Table S5).

Finally, *R. capsulatus* encodes a *hmu* operon (*rcc00094–* *rcc00098*) that presumably encodes a haem uptake transporter [39]. Its expression was increased more than 300 % during aerobic growth (Fig. S4, Table S5). The expression of the iron-storage protein bacterioferritin encoding gene (*bfr*) was also 30 % higher under aerobic growth conditions. In fact, *bfr* had by far the highest expression among the iron-transport genes in either growth state comprising 0.12 % of aerobic and 0.08 % of photosynthetic transcripts (Table S5). Presumably *R. capsulatus* aerobically increases expression of this iron sequestration protein so as to mitigate the formation of damaging oxygen radicals produced via Fenton chemistry that spontaneously occurs between free iron and oxygen [48]. There was also a recent report that ferrous iron can induce anaerobic toxicity via reduction of other metals, such the reduction of Cu^+2^ to Cu^+1^, providing additional reasons why these cells likely highly induce bacterioferritin under anaerobic conditions [49].

### Haem, bacteriochlorophyll and cobalamin tetrapyrrole branches have different expression profiles

**Fig. 4.**
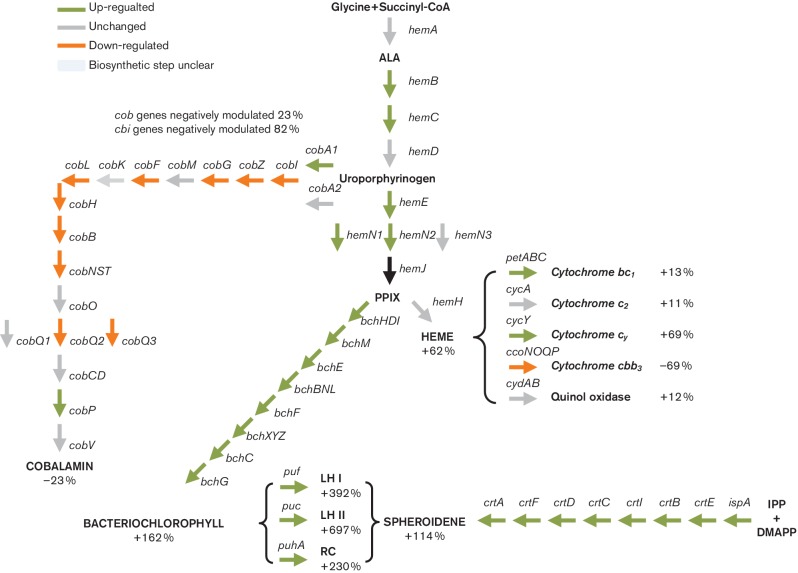
Tetrapyrrole and carotenoid biosynthetic pathways. Arrows indicate biosynthetic steps, with green and orange being positively and negatively differentially expressed, respectively. Grey arrows show steps that are not differentially expressed. Per cent changes are taken from DESeq2 normalized counts. Abbreviations; ALA, alpha-aminolevulinic acid; PPIX, protoporphyrin IX; IPP, isopentenyl diphosphate; DMA-PP, dimethylallyl diphosphate; LH, light harvesting; RC, reaction center.

Production of haem and cobalamin are obligatory under both aerobic and photosynthetic states, while bacteriochlorophyll is only synthesized under photosynthetic conditions [50]. All three branches share common intermediates from δ−aminolevulinic acid to uroporphyrinogen, at which point the cobalamin branch diverges with haem and bacteriochlorophyll continuing to share common intermediates up to protoporphyrin IX (Fig. 4) [29, 51]. Overall *hem* transcription accounted for 0.85 % of all transcripts during photosynthesis and 0.57 % during aerobiosis, and that of *bch* transcription accounted for 2.7 % under photosynthesis and 1.2 % under aerobiosis (Figs 1 and S5). The total increase in transcription of the haem pathway was 61.9 % during photosynthesis as compared to 162 % increase of the bacteriochlorophyll (Fig. 3). Of the genes that were differentially expressed in both the haem and bacteriochlorophyll pathways, 100 % of these genes were up-regulated during photosynthesis (Figs 2 and 4).

As expected, transcripts for genes involved in the bacteriochlorophyll biosynthesis branch of the tetrapyrrole pathway (*bch* genes) all increased during photosynthesis (Figs 3, 4 and S5, Table S4). However, *bch* genes were also expressed to a high degree during aerobic conditions where very little bacteriochlorophyll is synthesized (Fig. 1). This suggests that aerobically grown cell are in a state of translational readiness in anticipation of growth changes to an anaerobic environment. It has been proposed that Mg-proto IX methyl ester oxidative cyclase encoded by *bchE* (the third committed step of the bacteriochlorophyll pathway) has an oxygen labile FeS centre [39, 51–53]. Likewise, it has also been shown that dark protochlorophyllide reductase, which is the next step of the pathway, also has oxygen labile iron sulfur centres [54, 55]. Thus, it appears that oxygen regulation of the bacteriochlorophyll branch involves significant post-transcription regulation.

While all *bch* genes showed anaerobic increases in expression, there were several *hem* genes in the common δ−aminolevulinic acid to protoporphyrin IX trunk of the haem/bacteriochlorophyll pathways that did not show this response (Figs 4 and S5, Table S5). Interestingly, *hemA* encoding the first dedicated enzyme of the pathway, δ−aminolevulinic acid synthase, only showed a modest perhaps insignificant increase in expression. This is contrasted by the second gene in the pathway, *hemB*, which exhibited the highest fold increase among the *hem* genes (Table S5). This result is congruent with a prior study which indicated that the rate-limiting step of the haem pathway likely involves δ−aminolevulinic acid dehydratase encoded by *hemB* and not δ−aminolevulinic acid synthase encoded by *hemA* [56].

The entire cobalamin branch of the tetrapyrrole pathway is enzymatically consuming with a total of 37 annotated genes. Cobalamin biosynthesis genes were down-regulated (on average 45 %) during photosynthesis (Figs 4 and S5), with *cob* and *cbi* genes in this branch representing 0.72 % of aerobic transcripts and 0.38 % photosynthetic transcripts (Fig. 1). Presumably the cobalamin branch is attenuated as the haem and bacteriochlorophyll pathways are ramped up, so as to divert more uroporphyrinogen towards protoporphyrin IX for production of haem and bacteriochlorophyll (Fig. 4). Also of note is that Mg-proto IX methyl ester oxidative cyclase encoded by *bchE* has been proposed to require cobalamin, indicating that enough cobalamin must be synthesized anaerobically to fulfil this role even with a photosynthetic reduction in *cob* transcription [51, 57, 58].

### Electron transport and ATP synthase

**Fig. 5.**
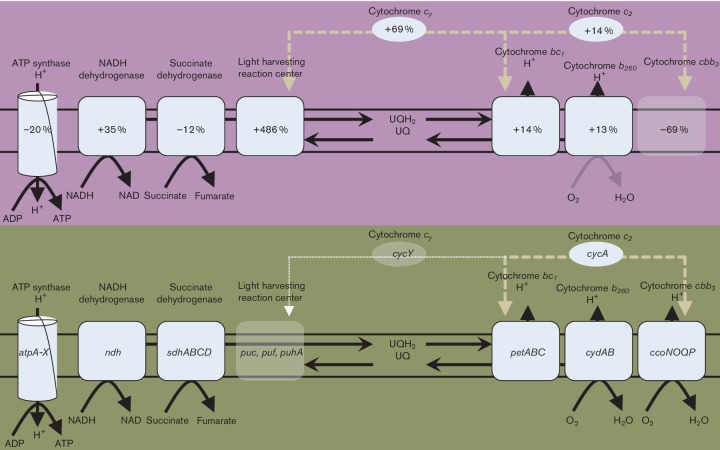
Diagramatic representation of the respiratory network in *R. capsulatus*. Photosynthesis has a purple background and aerobiosis has a green background. Transcriptional change during photosynthesis is shown inside individual complexes.

The *R. capsulatus* genome encodes two cytochrome oxidases, cytochrome *bd* oxidase (also called ubiquinone oxidase, obtaining electrons directly from ubiquinone) encoded by the *cydAB* operon, and cytochrome *cbb_3_* (also called cytochrome *c* oxidase, obtaining electrons from cytochrome *c_2_*) encoded by the *ccoNOQP* operon (Fig. 5) [26, 59–61]. Expression of the *cydAB* operon was largely unchanged between aerobic and anaerobic photosynthetic growth modes (Table S5). This cytochrome oxidase is proposed to have a high affinity for oxygen with a low turnover rate, which would be useful during photosynthesis to consume trace oxygen [62]. In contrast, the *ccoNOQP* operon is thought to encode a high turnover/low affinity oxidase useful for respiration under high oxygen tension conditions. Expression of the latter oxidase increased threefold to fourfold higher under aerobic than under photosynthetic growth conditions (Table S5).

Electrons are shuttled from the cytochrome *bc_1_* complex encoded by the *pet* operon either to the reaction centre via cytochrome *c_y_* encoded by *cycY* or from cytochrome *bc_1_* to cytochrome *cbb_3_* via a soluble cytochrome *c_2_* encoded by *cycA* (Fig. 5) [63, 64]. Cytochrome *bc_1_* expression remained relatively static, which is not surprising as this cytochrome is required for both photosynthesis and aerobic respiration. In regard to *cycY* expression that shuttles electrons to the photosystem, there was a 1.6-fold increase in expression during photosynthesis (Table S5). Regarding respiratory electron transport that utilizes periplasmic (non-membrane associated) cytochrome *c_2_*, there are two *cycA* homologs encoded by the *R. capsulatus* genome, *cycA1*, which was expressed at a high level with 0.2 % of the total transcript levels derived from this gene, and *cycA2*, which was expressed at a 36-fold lower level (Table S5). *cycA1* expression did not change expression levels, while *cycA2* expression was 3.5-fold higher during aerobiosis (Table S5). Given that CycA2 expression was extremely low, it may not be a major component of the respiratory of photosynthesis electron transport chain. An orthologue of *cycA2* (*exaB*) in *P. aeruginosa* forms a divergently transcribed pair with *exaA2* (quinoprotein ethanol dehydrogenase) used for oxidation of ethanol. A similar function of CycA2 could exist in *R. capsulatus*, but this has not yet been tested [65].

*R. capsulatus* is also capable of utilizing the alternative terminal electron acceptor trimethylamine *N*-oxide (TMAO). TMAO is reduced to trimethylamine by a reductase encoded by *torA* and *torC* that obtains electrons from ubiquinone [66]. Despite the fact that the growth medium in our study did not contain TMAO or DMSO, which is also a substrate, the expression of *torA* and *torC* was 18- and 31- fold higher under photosynthesis, respectively (Table S5).

In these cells, both metabolic and photosynthetic reducing potential is often stored in the membrane-bound ubiquinone pool. Anaerobic formation of an intracytoplasmic membrane that houses the photosystem would require additional photosynthetic synthesis of ubiquinones to prevent dilution of this pool. Indeed, ubiquinone biosynthetic genes (*ubiA*, *ubiD, ubiG, ubiH* and *ubiX*) exhibited an approximately twofold increase in expression during photosynthesis (Table S5).

### Motility is attenuated during photosynthesis

The motility group includes genes that encode proteins needed in flagella assembly, chemotaxis, aerotaxis and gas vesicle production. Interestingly, all of the various motility processes were attenuated during photosynthesis (Table S5). In fact, out of all the pathways that were analysed, motility genes had the second greatest reduction in expression during photosynthetic growth relative to aerobic growth (Fig. S5). Overall, a 31.7 % decrease in motility gene expression during photosynthesis was second only to a 42.4 % reduction in iron-transport genes (Fig. 3). Since light is the main energy source during photosynthesis, these cells presumably shut down motility, as a need to actively search for energy is not necessary.

Within the motility group of genes, gas vesicle production genes were the most repressed during photosynthesis, with all 10 genes decreasing to a mean amount of −65 % (Table S6). This was followed by chemotaxis and flagella assembly proteins, which had mean decreasing photosynthetic transcript levels of 36 and 30 %, respectively.

Given the overall reduced transcription level of motility genes during photosynthetic growth, it is surprising to see that a chemotaxis sensory transducer encoded by *rcc01185* was actually induced 2.5-fold during photosynthesis. One possibility is that this receptor has a role in governing phototaxis during photosynthesis. This gene shares homology with several other *mcpA* (methyl accepting chemotaxis), all of which are repressed during photosynthesis.

### Involvement of RegA, FnrL and CrtJ in modulating the aerobic and anaerobic photosynthetic states

A large number of similar aerobic and anaerobic photosynthetic data sets were collected for a variety of strains that have in frame deletions of transcription factors that have known roles in regulating the synthesis of the *R. capsulatus* photosystem. We chose to analyse the effect of deletions of RegA, CrtJ and FnrL on transcription patterns in the aerobic and anaerobic states, since each of these transcription factors are redox regulated and have global transcription functions [12–14, 16, 67]. Specifically, RegA is a DNA-binding response regulator that binds to target promoters after phosphorylation by the membrane-bound histidine sensor kinase RegB [32, 68–71]. The kinase activity of RegB is high under conditions where the ubiquinone pool is reduced (such as during anaerobic photosynthetic growth), but inactive under aerobiosis as the ubiquinone pool becomes oxidized [72, 73]. There is also a cytoplasmic redox reactive Cys that inhibits RegB kinase activity when oxidized [74, 75]. Phosphorylated RegA is known to control anaerobic expression of numerous photosystem structural genes, such as the genes involved in tetrapyrrole biosynthesis, cytochrome synthesis, hydrogen utilization, nitrogen fixation and carbon fixation [15, 32, 62, 76, 77]. CrtJ is a transcription factor that has a redox reactive Cys that when oxidized, stimulates DNA binding [67, 78]. CrtJ controls expression of numerous photosynthesis genes (bacteriochlorophyll, carotenoids, light harvesting and reaction centre genes), as well as genes involved in haem and cytochrome biosynthesis and a few metabolic genes [33, 78, 79]. FnrL is a transcription factor similar to *Escherichia coli* FNR that contains a redox sensitive 4Fe–4S centre coordinated by four Cys residues [80]. Under reducing conditions, this FeS centre promotes dimerization of FnrL that stimulates DNA binding; however, under oxidizing conditions the FeS centre disassembles, leading to loss of DNA binding activity [81–83]. Transcriptome profiling of FnrL has shown that it controls expression of genes involved in photosynthesis, tetrapyrroles and numerous cytochromes and several metabolic pathways [13]. Collectively, these redox-responding transcription factors appear to control many physiological changes that occur when these cells transition from an aerobic to photosynthetic environment.

**Fig. 6.**
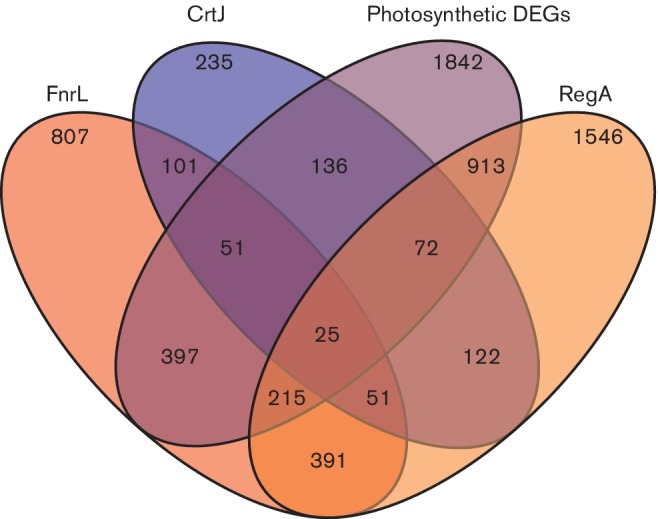
Common control of genes during photosynthesis by known photosynthetic regulators. DEGs for RegA (orange) FnrL (red) and CrtJ (blue) during photosynthesis, and photosynthetically DEGs (purple) are indicated.

Detailed transcriptome analyses of genes regulated by each of these redox regulators have recently been published [12–14]. We cross-referenced all of the 1842 DEGs in this data set with DEGs present in wild-type/regulatory mutant data sets. The dendrogram in Fig. 6, and the data in Table S7, show that these three regulators control the expression of more than 1000 genes. Expression of 50 % of the DEGs (913 of 1842 total genes) was affected by a deletion of RegA, while 31 % (575 out of 1842) were affected by FnrL, and just 7 % (136 out of 1842) by CrtJ. Numerous genes were also controlled by more than one redox-responding regulator, for example, 25 DEGs exhibited altered expression by deletions of FnrL, CrtJ and RegA (Table S7). A total of 51 common DEGs exhibited altered expression between CrtJ and FnrL, 72 common genes were affected between RegA and CrtJ, and 391 common genes were affected between RegA and FnrL. Thus, the RegA and FnrL regulons exhibit a considerable overlap in their gene targets. Interestingly, FnrL and CrtJ did not share much transcriptional expression overlap, having only two genes that were down-regulated and seven genes that were up-regulated for both.

**Fig. 7.**
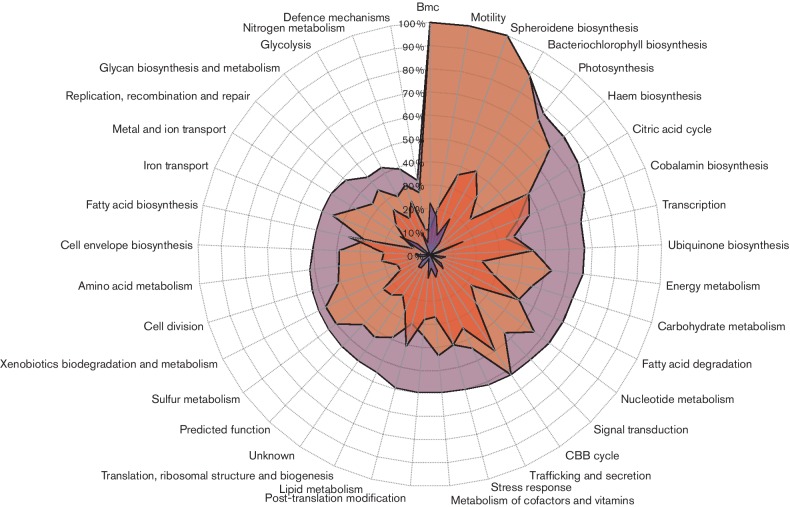
Involvement of RegA, FnrL and CrtJ in global functioning. Percentages are defined to be the number of genes within a pathway that are differentially expressed for RegA (orange), FnrL (red) and CrtJ (blue). The total control by these regulators of a particular pathway is shown in purple.

RegA was involved in controlling the expression of virtually all of the motility, carotenoid (spheroidene) and carboxysome biosynthesis (or bacterial micro-compartment, BMC) pathways (Fig. 7). FnrL and RegA together had major roles in controlling the expression of genes involved in carbon fixation, cobalamin biosynthesis, the citric acid cycle, fatty acid biosynthesis and degradation, ubiquinone biosynthesis, lipid metabolism carbohydrate metabolism, stress response and trafficking/secretion (Fig. 7). CrtJ, however, had a more limited role, exerting effects on photosynthetic expression of bacteriochlorophyll, carotenoids, the light harvesting II complex, carboxysome biosynthesis, motility and post-translational modification (Fig. 7).

**Fig. 8.**
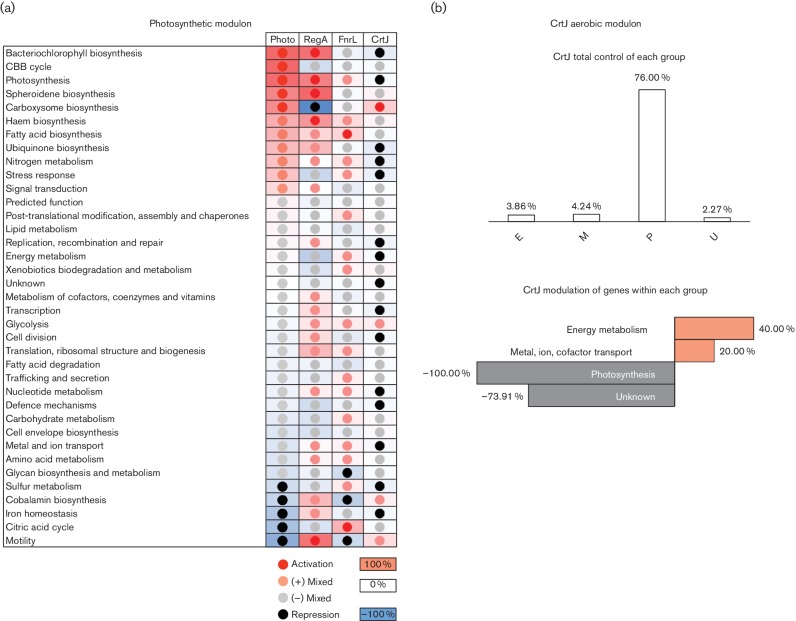
Global transcriptional trend during photosynthesis (a) and CrtJ during aerobiosis (b). (a) Coloured boxes represent the percentage of positively (red) and negatively (blue) DEGs as compared to the total number of genes for a given pathway. Coloured circles are normalized for individual columns to show the trend for photosynthesis, RegA, FnrL and CrtJ as being either activators (red), repressors (black) or mixed regulators (light red and light grey) for a given pathway. (b) CrtJ modulation during aerobiosis.

Genes targeted by FnrL did not exhibit large fold-changes in gene transcription, which was also observed previously (Table S7) [13]. Rather, FnrL appeared to act more as a moderator of transcription than as a pure repressor or activator. Overall, the gene modulation followed the global transcriptional trend, though not without exception to opposing global photosynthetic trends. For example, FnrL opposed the global trend by up-regulating the TCA cycle, which is among the most negatively regulated groups during photosynthetic growth (Fig. 8). Within most groups, FnrL was a mixed regulator not modulating an entire pathway either positively or negatively, but rather activation of one gene was often associated with repression of another. The best example of this included regulation of *bch* genes, where *bchC*, *bchE* and *bchF* were activated, but *bchM, bchJ, bchO, and bchD* were repressed. Also, FnrL appeared to have dominance over CrtJ and RegA in motility, since its modulation complemented that of the overall global trend, though its repression of motility genes was weaker than the activation of RegA alone.

There were several instances where a redox-responding regulator acted in opposition to the global transcriptional trend (Fig. 8). For example, motility, as measured by expression of flagellar (*fla*) genes, was reduced under photosynthesis. FnrL generally repressed anaerobic *fla* expression, which was congruent to the expression profile, while RegA and CrtJ activated the expression of *fla*. Similarly, RegA opposed the global trend of increased photosynthetic expression of enzymes involved in CBB biosynthesis.

### Concluding remarks

This study provides a global assessment of the differing transcription loads that occur in *R. capsulatus* during aerobic respiration versus photosynthetic growth states. The aim of this study was to provide a framework of global transcriptional trends against which specific gene knockouts could be referenced, by providing context for individual gene disruptions as they relate to natural changes between aerobic and photosynthetic states rather than interpreting them in isolation. The data in this study should help guide future hypothesis-driven research in metabolomics in photosynthetic bacteria. Here, we have shown that more than half of the genes (1834 out of 3493) encoded by *R. capsulatus* were observed to exhibit differences in expression in aerobic versus anaerobic photosynthetic growth states. Changes in expression in such a wide variety of genes highlight that there are significant changes in metabolism, energy production (photosynthesis versus respiratory electron transport), energy utilization and motility. We also have shown that transcription expression, in large part, is not an all or nothing event. For example, in the case of *bch* gene expression, *R. capsulatus* modulated expression in such a way that there appeared to be basal expression that is ready for translation in the event of a change in growth mode.

The global transcriptome trends observed here are broadly in agreement with analogous studies in *R. sphaeroides*, but unlike previous examples, we have delineated relative gene transcription ranges, as well as showing how major metabolic pathways are transcriptionally perturbed relative to branching pathways [7, 8]. Due to the large number of pathways that were affected by these different growth conditions, attention was paid to critical processes connected with photosynthesis and the involvement of the global regulators RegA, FnrL and CrtJ in perturbing expression of the annotated genome. It is evident that these global regulators have specialized niches of transcriptional modulation of specific pathways, with all three regulators perturbing the transcription of only 25 out of 1842 DEGs. Furthermore, the overall direction of regulation is unique to each regulator and need not necessarily coincide with the global transcriptional trend. Our studies also suggest that the transcriptional level of genes is modulatory rather than behaving as binary switches. As a result, a significant portion of regulatory events in *R. capsulatus* must exist on the protein and metabolite level. For example, the *fnrL* gene is constitutively transcribed during both aerobiosis and photosynthesis even though FnrL is inactive during aerobiosis.

## Data bibliography

1. Kumka JE, Schindel H, Fang M, Zappa S, and Bauer CE. RNA-seq sequences, National Center for Biotechnology Information Sequence Read Archive PRJNA357604 (2016).

## Supplementary Data

2119 Supplementary File 1
