# Supplementary material for: Transcriptomic analysis of aerobic respiratory and anaerobic photosynthetic states in Rhodobacter capsulatus and their modulation by global redox regulators RegA, FnrL and CrtJ
Source: Microb Genom. 2017 Jul 8;3(9):e000125. doi: 10.1099/mgen.0.000125 (PMC5643017; doi:10.1099/mgen.0.000125)
Supplement: Supplementary File 1 [file mgen-3-125-s001.pdf]

Table S1. qRT-PCR target gene primers

**Target**	**Sequence**
RCC02578-F	gaaatctgcgccaaacttgt
RCC02578-R	aaatgacgagcgaaagatcg
bchC-F	ttctatacggtgccggtcag
bchC-R	tcttcaggcagtcagggtct
bchF-F	gctcgtccttgtggtgagat
bchF-R	ggaaaacacgtcttcccaaa
ccoN-F	tggtttcgatcggcttttac
ccoN-R	ggaagtagagcatgccgaag
cycA2-F	gggctatccctattcgaagg
cycA2-R	ggccagatattcgacgatga
cycY-F	cacaagatcgatggcaagaa
cycY-R	cgggctgaccagatagatgt
feoB1-F	gcaaggatctgaccgaaaaa
feoB1-R	cagatacatcagcgcaagga
feoB2-F	gatcccggtctatacgctga
feoB2-R	ggatcttgtaatcgggcaga
hemA-F	gtcttctcctcggcctatatcgccaat
hemA-R	gaatagaccgattcgaaggcgatcag
hemB-F	aagacctatcagatgaaccccgccaac
hemB-R	tattcgcccgagacctgataggcatag
hemC-F	ggccttttcaccaaggaaatcgaagag
hemC-R	cgagcgaattgtatttcatcgaaacga
hemH-F	ggcaacaactatcgctcgatctggaac
hemH-R	atattgcggataaagcggcaggaacag
pucB-F	gcctgtcgctgaaagaagctgaagaaa
pucB-R	atggccgagaggatgtgcgcaacaag
puhA-F	gcaagaccgagatgaaggtt
puhA-R	tcttcttgcccgagttcagt
Reference Gene	
rpoZ-F	gtgacggttgaagattgcgttgac
rpoZ-R	gatctgggtctggttgctttcgat

Table S2. Pearson Correlation Coefficients (r) between different biological replicates

A) Aerobic Replicates

	**Aero-1**	**Aero-2**	**Aero-3**	**Aero-4**	**Aero-5**	**Aero-6**	**Aero-7**	**Aero-8**	**Aero-9**	**Aero-10**	**Aero-11**	**Aero-12**	**Aero-13**	**Aero-14**	**Aero-15**	**Aero-16**	**Aero-17**	**Aero-18**
**Aero-1**		0.98	0.95	0.97	0.93	0.96	0.92	0.97	0.90	0.96	0.89	0.98	0.86	0.96	0.89	0.94	0.84	0.91
**Aero-2**	0.98		0.97	0.98	0.96	0.99	0.96	0.99	0.95	0.98	0.93	0.99	0.83	0.88	0.96	0.92	0.83	0.89
**Aero-3**	0.95	0.97		0.95	0.97	0.96	0.97	0.96	0.95	0.96	0.94	0.96	0.83	0.89	0.86	0.90	0.83	0.90
**Aero-4**	0.97	0.98	0.95		0.94	0.96	0.92	1.00	0.93	0.94	0.88	1.00	0.85	0.80	0.97	0.94	0.84	0.90
**Aero-5**	0.93	0.96	0.97	0.94		0.98	0.98	0.94	0.97	0.97	0.98	0.95	0.89	0.94	0.92	0.85	0.87	0.83
**Aero-6**	0.96	0.99	0.96	0.96	0.98		0.98	0.97	0.96	0.99	0.97	0.97	0.81	0.86	0.94	0.88	0.89	0.86
**Aero-7**	0.92	0.96	0.97	0.92	0.98	0.98		0.93	0.97	0.98	0.99	0.93	0.80	0.83	0.93	0.86	0.89	0.85
**Aero-8**	0.97	0.99	0.96	1.00	0.94	0.97	0.93		0.93	0.95	0.89	1.00	0.85	0.90	0.88	0.94	0.84	0.90
**Aero-9**	0.90	0.95	0.95	0.93	0.97	0.96	0.97	0.93		0.94	0.95	0.94	0.84	0.87	0.89	0.85	0.80	0.82
**Aero-10**	0.96	0.98	0.96	0.94	0.97	0.99	0.98	0.95	0.94		0.96	0.96	0.87	0.85	0.94	0.89	0.80	0.87
**Aero-11**	0.89	0.93	0.94	0.88	0.98	0.97	0.99	0.89	0.95	0.96		0.90	0.86	0.87	0.89	0.80	0.83	0.80
**Aero-12**	0.98	0.99	0.96	1.00	0.95	0.97	0.93	1.00	0.94	0.96	0.90		0.85	0.82	0.88	0.93	0.84	0.90
**Aero-13**	0.86	0.83	0.83	0.85	0.89	0.81	0.80	0.85	0.84	0.87	0.86	0.85		0.95	0.97	0.89	0.89	0.85
**Aero-14**	0.96	0.88	0.89	0.80	0.94	0.86	0.83	0.90	0.87	0.85	0.87	0.82	0.95		0.95	0.85	0.80	0.93
**Aero-15**	0.89	0.96	0.86	0.97	0.92	0.94	0.93	0.88	0.89	0.94	0.89	0.88	0.97	0.95		0.81	0.85	0.89
**Aero-16**	0.94	0.92	0.90	0.94	0.85	0.88	0.86	0.94	0.85	0.89	0.80	0.93	0.89	0.85	0.81		0.94	0.97
**Aero-17**	0.84	0.83	0.83	0.84	0.87	0.89	0.89	0.84	0.80	0.80	0.83	0.84	0.89	0.80	0.85	0.94		0.96
**Aero-18**	0.91	0.89	0.90	0.90	0.83	0.86	0.85	0.90	0.82	0.87	0.80	0.90	0.85	0.93	0.89	0.97	0.96	

B. Photosynthetic Replicates

	Photo-1	Photo-2	Photo-3	Photo-4	Photo-5	Photo-6	Photo-7	Photo-8	Photo-9	Photo-10	Photo-11	Photo-12	Photo-13	Photo-14	Photo-15	Photo-16	Photo-17	Photo-18
**Photo-1**		0.97	0.96	0.98	0.93	0.98	0.98	0.99	0.93	0.97	0.97	0.98	0.96	0.97	0.98	0.93	0.91	0.95
**Photo-2**	0.97		0.98	0.98	0.90	0.99	0.96	0.98	0.92	0.99	0.93	0.98	0.98	0.97	0.97	0.92	0.90	0.93
**Photo-3**	0.96	0.98		0.97	0.88	0.98	0.97	0.96	0.89	0.97	0.94	0.96	0.97	0.95	0.96	0.92	0.91	0.93
**Photo-4**	0.98	0.98	0.97		0.91	0.99	0.97	0.99	0.90	0.96	0.96	1.00	0.99	0.97	0.98	0.93	0.92	0.94
**Photo-5**	0.93	0.90	0.88	0.91		0.91	0.95	0.93	0.99	0.90	0.95	0.92	0.90	0.95	0.95	0.89	0.87	0.91
**Photo-6**	0.98	0.99	0.98	0.99	0.91		0.97	0.99	0.91	0.99	0.96	0.99	0.99	0.98	0.98	0.94	0.92	0.95
**Photo-7**	0.98	0.96	0.97	0.97	0.95	0.97		0.98	0.95	0.96	0.98	0.97	0.96	0.98	0.99	0.94	0.92	0.96
**Photo-8**	0.99	0.98	0.96	0.99	0.93	0.99	0.98		0.92	0.98	0.97	0.99	0.98	0.98	0.99	0.94	0.92	0.95
**Photo-9**	0.93	0.92	0.89	0.90	0.99	0.91	0.95	0.92		0.92	0.93	0.91	0.89	0.95	0.95	0.87	0.85	0.89
**Photo-10**	0.97	0.99	0.97	0.96	0.90	0.99	0.96	0.98	0.92		0.93	0.96	0.96	0.98	0.97	0.91	0.88	0.92
**Photo-11**	0.97	0.93	0.94	0.96	0.95	0.96	0.98	0.97	0.93	0.93		0.96	0.95	0.97	0.98	0.96	0.94	0.98
**Photo-12**	0.98	0.98	0.96	1.00	0.92	0.99	0.97	0.99	0.91	0.96	0.96		0.99	0.97	0.98	0.93	0.92	0.95
**Photo-13**	0.96	0.98	0.97	0.99	0.90	0.99	0.96	0.98	0.89	0.96	0.95	0.99		0.96	0.97	0.93	0.93	0.95
**Photo-14**	0.97	0.97	0.95	0.97	0.95	0.98	0.98	0.98	0.95	0.98	0.97	0.97	0.96		1.00	0.94	0.92	0.96
**Photo-15**	0.98	0.97	0.96	0.98	0.95	0.98	0.99	0.99	0.95	0.97	0.98	0.98	0.97	1.00		0.95	0.92	0.96
**Photo-16**	0.93	0.92	0.92	0.93	0.89	0.94	0.94	0.94	0.87	0.91	0.96	0.93	0.93	0.94	0.95		0.98	0.99
**Photo-17**	0.91	0.90	0.91	0.92	0.87	0.92	0.92	0.92	0.85	0.88	0.94	0.92	0.93	0.92	0.92	0.98		0.97
**Photo-18**	0.95	0.93	0.93	0.94	0.91	0.95	0.96	0.95	0.89	0.92	0.98	0.95	0.95	0.96	0.96	0.99	0.97	

Table S3. Summarized results for validation of RNA-seq by qRT-PCR of dark aerobic and anaerobic photosynthetic states in *Rba. capsulatus*

ID	Gene Name	qRT-PCR Expression	P-Value	RNA-seq Expression	P-Value	Agreement
RCAP_rcc02530	*pucB*	18.6	0	8.57	0	Yes
RCAP_rcc00686	*bchC*	7.2	0	4.9	0	Yes
RCAP_rcc00659	*puhA*	4.1	0.001	3.23	0	Yes
RCAP_rcc00666	*bchF*	4	0	2.19	0	Yes
RCAP_rcc01173	*hemC*	1.8	0.007	2.73	0	Yes
RCAP_rcc02029	*feoB2*	1.7	0.046	1.14	0.009	Yes
RCAP_rcc01808	*hemB*	-	-	1.34	0.004	No
RCAP_rcc02501	*cycY*	-	-	1.66	0.002	No
RCAP_rcc01447	*hemA*	-	-	-	-	Yes
RCAP_rcc00195	*hemH*	-	-	-	-	Yes
RCAP_rcc00092	*feoB1*	-2.5	0.008	-2.83	0	Yes
RCAP_rcc01157	*ccoN*	-2.5	0.004	-2.93	0	Yes
RCAP_rcc01656	*cycA2*	-7.7	0.001	-3.5	0	Yes
RCAP_rcc02578	*Iron(III) Transporter*	-27.0	0.002	-14	0	Yes

rpoZ house keeping gene was used a reference
p-values and fold-changes for qRT-PCR were determined using REST® 2009
Fold-changes represent differences in transcripts in comparison of [photosynthetic / aerobic] states
Dashes (-) represent instances where no statistically significant ($p < 0.05$) differential expression was observed from three independent biological replicates

Table S4. Top most transcribed genes under anaerobic photosynthetic and dark aerobic conditions. Log2FC is defined as log2FC([photosynthetic expression]/[aerobic expression]).

Anaerobic photosynthetic

ID	log2FC	padj	Aero Counts	Photo Counts	% Photo	% Aero	Gene	Global Function
RCAP_rcc00259	0.53	0	211311	305332	4.15%	3.09%	*porin family protein*	Cell Envelope Biosynthesis
RCAP_rcc02530	3.1	0	22813	212493	2.89%	0.46%	*pucB*	Photosynthesis
RCAP_rcc00691	2.13	0	27214	126480	1.72%	0.49%	*pufB*	Photosynthesis
RCAP_rcc02531	3.45	0	10822	126140	1.71%	0.24%	*pucA*	Photosynthesis
RCAP_rcc00692	2.17	0	21128	100184	1.36%	0.38%	*pufA*	Photosynthesis
RCAP_rcc00693	2.37	0	13547	74399	1.01%	0.25%	*pufL*	Photosynthesis
RCAP_rcc02533	2.49	0	11763	69872	0.95%	0.22%	*pucDE*	Photosynthesis
RCAP_rcc00694	2.32	0	13131	68952	0.94%	0.24%	*pufM*	Photosynthesis
RCAP_rcc00296	-0.24	0.299	78928	66310	0.90%	1.11%	*fusA1*	Translation, ribosomal structure and biogenesis
RCAP_rcc00871	1.34	0	22634	59714	0.81%	0.37%	*hypothetical protein*	Unknown
RCAP_rcc00659	1.69	0	17102	56589	0.77%	0.28%	*puhA*	Photosynthesis
RCAP_rcc02478	-0.28	0.2856	69131	56473	0.77%	0.96%	*groL*	Post-translational Modification, Assembly and Chaperones
RCAP_rcc00289	-0.27	0.3289	61844	50638	0.69%	0.87%	*rplJ*	Translation, ribosomal structure and biogenesis
RCAP_rcc00686	2.3	0	9549	49433	0.67%	0.17%	*bchC*	Photosynthesis
RCAP_rcc03525	-0.57	0.0001	69719	46683	0.63%	0.96%	*flaA*	Motility
RCAP_rcc00224	0.66	0.0007	27557	44250	0.60%	0.41%	*dnaK*	Post-translational Modification, Assembly and Chaperones
RCAP_rcc00292	-0.28	0.1393	51581	42085	0.57%	0.72%	*rpoC*	Replication, Recombination and Repair
RCAP_rcc00297	-0.15	0.3943	45634	40883	0.56%	0.64%	*tuf2*	Translation, ribosomal structure and biogenesis
RCAP_rcc02769	0.12	0.5727	34149	37286	0.51%	0.49%	*petB*	Energy Metabolism
RCAP_rcc00147	-0.13	0.511	40136	36672	0.50%	0.57%	*tuf1*	Translation, ribosomal structure and biogenesis

Dark aerobic

ID	log2FC	padj	Aero Counts	Photo Counts	% Photo	% Aero	Gene	Global Function
RCAP_rcc00259	0.53	0	211311	305332	4.15%	3.09%	*porin family protein*	Cell Envelope Biosynthesis
RCAP_rcc00296	-0.24	0.299	78928	66310	0.90%	1.11%	*fusA1*	Translation, ribosomal structure and biogenesis
RCAP_rcc03525	-0.57	0.0001	69719	46683	0.63%	0.96%	*flaA*	Motility
RCAP_rcc01157	-1.55	0	69618	22672	0.31%	0.94%	*ccoN*	Energy Metabolism
RCAP_rcc02478	-0.28	0.2856	69131	56473	0.77%	0.96%	*groL*	Post-translational Modification, Assembly and Chaperones
RCAP_rcc01620	-2.81	0	66679	7709	0.10%	0.90%	*aldB*	Carbohydrate Metabolism
RCAP_rcc00289	-0.27	0.3289	61844	50638	0.69%	0.87%	*rplJ*	Translation, ribosomal structure and biogenesis
RCAP_rcc00292	-0.28	0.1393	51581	42085	0.57%	0.72%	*rpoC*	Replication, Recombination and Repair
RCAP_rcc01244	-1.74	0	48039	13658	0.19%	0.65%	*potD1*	Unknown
RCAP_rcc02973	-0.35	0.0691	47063	36622	0.50%	0.66%	*atpA*	Energy Metabolism
RCAP_rcc01125	-0.4	0.1174	46509	34547	0.47%	0.65%	*rpsA*	Translation, ribosomal structure and biogenesis
RCAP_rcc02971	-0.34	0.0386	46005	36077	0.49%	0.65%	*atpD*	Energy Metabolism
RCAP_rcc00297	-0.15	0.3943	45634	40883	0.56%	0.64%	*tuf2*	Translation, ribosomal structure and biogenesis
RCAP_rcc00291	-0.43	0.0233	43943	32301	0.44%	0.61%	*rpoB*	Replication, Recombination and Repair
RCAP_rcc00147	-0.13	0.511	40136	36672	0.50%	0.57%	*tuf1*	Translation, ribosomal structure and biogenesis
RCAP_rcc00284	-0.33	0.0085	35251	27871	0.38%	0.49%	*pnp*	Nucleotide Metabolism
RCAP_rcc00290	-0.27	0.2442	34317	28154	0.38%	0.48%	*rplL*	Translation, ribosomal structure and biogenesis
RCAP_rcc02769	0.12	0.5727	34149	37286	0.51%	0.49%	*petB*	Energy Metabolism
RCAP_rcc01674	0.06	0.6868	31065	32383	0.44%	0.44%	*glnA3*	Carbohydrate Metabolism
RCAP_rcc00846	-1.15	0	30290	13442	0.18%	0.41%	*dppA*	Unknown

Table S5. Differential expression as compared between photosynthetic vs aerobic states.

ID	log2FC	padj	Mean Aero Counts	Mean Photo Counts	% Aero	% Photo	95% CI - Aero	95% CI + Aero	95% CI - Photo	95% CI + Photo	Gene	Global Function	Pathway Name
RCAP_rcc00001	-0.91	0	3672	1913	0.05%	0.03%	2979	4161	1608	2218	*dnaA*	Replication, Recombination and Repair	Replication
RCAP_rcc00002	-0.39	0.0001	5237	3995	0.07%	0.05%	4619	5710	3724	4266	*dnaN*	Replication, Recombination and Repair	Replication
RCAP_rcc00003	-0.78	0	644	372	0.01%	0.01%	573	681	343	402	*recF*	Replication, Recombination and Repair	Repair
RCAP_rcc00004	-0.3	0.0449	6986	5662	0.10%	0.08%	5935	7934	5070	6254	*gyrB*	Replication, Recombination and Repair	Unknown
RCAP_rcc00005	-0.16	0.15	329	292	0.00%	0.00%	293	354	266	318	*ntaB*	Unknown	Unknown
RCAP_rcc00006	-0.91	0	2230	1160	0.03%	0.02%	1769	2569	979	1340	*motB*	Motility	Chemotaxis
RCAP_rcc00007	-0.12	0.3432	5901	5425	0.08%	0.07%	5273	6615	4958	5892	*flgE*	Motility	Flagellar Assembly
RCAP_rcc00008	-0.4	0.0001	4208	3177	0.06%	0.04%	3737	4593	2941	3414	*flgK*	Motility	Flagellar Assembly
RCAP_rcc00009	-0.48	0	2222	1585	0.03%	0.02%	1975	2430	1429	1740	*flgL*	Motility	Flagellar Assembly
RCAP_rcc00010	-0.31	0.005	1825	1469	0.03%	0.02%	1615	2007	1350	1587	*flgI*	Motility	Flagellar Assembly
RCAP_rcc00011	0.94	0	254	499	0.00%	0.01%	222	296	398	601	*hemolysin D*	Trafficking and Secretion	Secretion
RCAP_rcc00012	0.39	0.0086	122	161	0.00%	0.00%	105	140	148	175	*ABC transporter ATP-binding protein*	Defense Mechanisms	Unknown
RCAP_rcc00013	0.82	0.0115	338	638	0.00%	0.01%	200	464	407	869	*inner membrane transport permease*	Defense Mechanisms	Unknown
RCAP_rcc00014	1.3	NA	2927	9202	0.04%	0.12%	937	4757	4863	13541	*xylA*	Carbohydrate Metabolism	Pentose and glucuronate interconversions
RCAP_rcc00015	1.14	NA	1427	4135	0.02%	0.06%	300	2463	2163	6106	*xylB*	Carbohydrate Metabolism	Pentose and glucuronate interconversions
RCAP_rcc00016	1.17	NA	1507	4396	0.02%	0.06%	484	2439	1955	6836	*xylG*	Carbohydrate Metabolism	Xylose Transport
RCAP_rcc00017	1.21	NA	2425	7352	0.03%	0.10%	697	4015	3667	11037	*xylH*	Carbohydrate Metabolism	Xylose Transport
RCAP_rcc00018	0.97	NA	12116	29501	0.17%	0.40%	3088	20521	17960	41043	*xylF*	Carbohydrate Metabolism	Xylose Transport
RCAP_rcc00019	2.15	0	118	675	0.00%	0.01%	72	165	381	969	*xylR*	Signal Transduction	Transcription Regulator
RCAP_rcc00020	1.6	0	383	1299	0.01%	0.02%	267	508	879	1720	*FG-GAP repeat domain-containing protein*	Unknown	Unknown
RCAP_rcc00021	-0.7	0	2334	1439	0.03%	0.02%	2157	2416	1360	1518	*cytochrome c peroxidase*	Energy Metabolism	Aerobic/Anaerobic Respiration
RCAP_rcc00022	-0.72	0	3945	2384	0.05%	0.03%	3575	4178	2152	2616	*aldo/keto reductase family oxidoreductase*	Energy Metabolism	Aerobic/Anaerobic Respiration
RCAP_rcc00023	-0.81	0.0011	1430	788	0.02%	0.01%	816	1958	678	897	*ribonuclease BN*	Unknown	Unknown
RCAP_rcc00024	-0.93	0.0004	5241	2623	0.07%	0.04%	2949	7169	2057	3189	*gcdH*	Lipid Metabolism	Unknown
RCAP_rcc00025	-0.07	0.67	179	169	0.00%	0.00%	157	199	146	193	*LysR family transcriptional regulator*	Signal Transduction	Transcription Regulator
RCAP_rcc00026	-0.37	0.0274	3783	2906	0.05%	0.04%	3097	4337	2559	3253	*hypothetical protein*	Unknown	Unknown
RCAP_rcc00027	0	0.982	1458	1462	0.02%	0.02%	1269	1608	1270	1654	*surface antigen*	Cell Envelope Biosynthesis	Cell Wall Biosynthesis
RCAP_rcc00028	1.6	0	243	772	0.00%	0.01%	221	327	632	911	*idi1*	Photosynthesis	Terpenoid backbone biosynthesis
RCAP_rcc00029	0.61	0.0002	3584	5512	0.05%	0.07%	3158	4329	4734	6290	*secB*	Trafficking and Secretion	Secretion
RCAP_rcc00030	1.78	0	185	653	0.00%	0.01%	193	239	555	750	*fxsA*	Unknown	Unknown
RCAP_rcc00031	1.94	0	1904	7643	0.03%	0.10%	2087	2707	6481	8805	*import inner membrane translocase subunit Tim44*	Unknown	Unknown
RCAP_rcc00032	1.51	0	479	1392	0.01%	0.02%	496	586	1185	1598	*Smr protein/MutS2*	Unknown	Unknown
RCAP_rcc00033	0.56	0	1562	2311	0.02%	0.03%	1444	1755	2182	2440	*hypothetical protein*	Unknown	Unknown
RCAP_rcc00034	1.78	0	2037	7198	0.03%	0.10%	2307	2663	6158	8239	*hslU*	Post-translational Modification, Assembly and Chaperones	Unknown
RCAP_rcc00035	2.36	0	523	2853	0.01%	0.04%	602	771	2436	3271	*hslV*	Post-translational Modification, Assembly and Chaperones	Peptidase
RCAP_rcc00036	-0.08	0.5516	2997	2830	0.04%	0.04%	2725	3298	2523	3137	*trxA1*	Post-translational Modification, Assembly and Chaperones	Unknown
RCAP_rcc00037	-0.44	0	1994	1464	0.03%	0.02%	1826	2083	1327	1601	*UvrD/REP helicase*	Replication, Recombination and Repair	Unknown
RCAP_rcc00038	-0.31	0.0003	1403	1132	0.02%	0.02%	1270	1490	1060	1204	*addB*	Unknown	Unknown
RCAP_rcc00039	-0.47	0.0001	517	373	0.01%	0.01%	447	569	338	409	*nucleotidyltransferase*	Cell Envelope Biosynthesis	Cell Wall Biosynthesis
RCAP_rcc00040	-0.09	0.2769	273	256	0.00%	0.00%	250	289	243	269	*aminoglycoside phosphotransferase*	Unknown	Unknown
RCAP_rcc00041	0.09	0.5079	331	354	0.00%	0.00%	291	368	322	385	*hypothetical protein*	Unknown	Unknown
RCAP_rcc00042	0.39	0.0098	1801	2368	0.03%	0.03%	1577	2102	2220	2516	*PAS/PAC sensor domain-containing protein*	Motility	Aerotaxis
RCAP_rcc00043	-0.1	0.255	1896	1774	0.03%	0.02%	1748	2019	1671	1877	*regB*	Signal Transduction	Kinase/Phosphorelay
RCAP_rcc00044	0.42	0.0006	1430	1916	0.02%	0.03%	1270	1644	1760	2071	*senC*	Unknown	Unknown
RCAP_rcc00045	-0.24	0.0656	5386	4543	0.08%	0.06%	4729	6006	4068	5018	*regA1*	Signal Transduction	Transcription Regulator
RCAP_rcc00046	-0.52	0.0217	5261	3608	0.07%	0.05%	3926	6436	2881	4336	*hvrA*	Signal Transduction	Transcription Regulator
RCAP_rcc00047	-0.66	0.002	576	357	0.01%	0.00%	430	694	305	409	*hvrB*	Transcription	Unknown
RCAP_rcc00048	1.57	0	3256	10167	0.05%	0.14%	2927	3958	7664	12670	*metal dependent phosphohydrolase*	Unknown	Unknown
RCAP_rcc00049	-0.22	0.1596	13503	11542	0.19%	0.16%	11422	15667	10281	12804	*ahcY*	Post-translation	Cysteine and methionine metabolism
RCAP_rcc00050	-0.89	0	900	476	0.01%	0.01%	707	1050	411	540	*hypothetical protein*	Unknown	Unknown
RCAP_rcc00051	-0.15	0.5899	238	214	0.00%	0.00%	170	293	175	252	*enoyl-CoA hydratase/isomerase*	Xenobiotics Biodegradation and Metabolism	Caprolactam degradation
RCAP_rcc00052	-0.1	0.5005	8963	8365	0.12%	0.11%	7862	9922	7452	9278	*hypothetical protein*	Unknown	Unknown
RCAP_rcc00053	-0.5	0	1469	1032	0.02%	0.01%	1277	1588	922	1142	*U62 family peptidase*	Post-translational Modification, Assembly and Chaperones	Peptidase
RCAP_rcc00054	-0.54	0	724	496	0.01%	0.01%	622	795	466	526	*inositol monophosphatase*	Carbohydrate Metabolism	Unknown
RCAP_rcc00055	-0.33	0.1199	1109	875	0.02%	0.01%	868	1340	757	993	*transglycosylase, Slt family*	Cell Envelope Biosynthesis	Cell Wall Biosynthesis
RCAP_rcc00056	-0.09	0.7132	1715	1602	0.02%	0.02%	1332	2127	1411	1792	*flhA*	Motility	Flagellar Assembly
RCAP_rcc00057	-0.07	0.8132	517	492	0.01%	0.01%	400	647	422	562	*fliR*	Motility	Flagellar Assembly
RCAP_rcc00058	-0.24	0.3605	979	820	0.01%	0.01%	718	1243	705	935	*flhB*	Motility	Flagellar Assembly
RCAP_rcc00059	-0.28	0.1455	273	223	0.00%	0.00%	223	323	201	245	*hypothetical protein*	Unknown	Unknown
RCAP_rcc00060	-0.47	0.0118	2380	1702	0.03%	0.02%	1944	2769	1419	1985	*parB*	Cell Division	Chromosome Partitioning
RCAP_rcc00061	-0.3	0.1567	1371	1101	0.02%	0.01%	1096	1637	894	1309	*parA*	Cell Division	Chromosome Partitioning
RCAP_rcc00062	-0.71	0.006	1187	700	0.02%	0.01%	861	1468	523	877	*gidB*	Cell Envelope Biosynthesis	Cell Wall Biosynthesis
RCAP_rcc00063	-0.58	0.0137	4061	2668	0.06%	0.04%	3058	4945	2116	3220	*gidA*	Cell Division	Chromosome Partitioning
RCAP_rcc00064	-0.51	0.0227	1593	1097	0.02%	0.01%	1224	1903	888	1306	*trmE*	Unknown	Unknown
RCAP_rcc00065	-0.24	0.3671	4030	3386	0.06%	0.05%	2950	5032	2740	4032	*rho*	Transcription	Unknown
RCAP_rcc00066	-0.05	0.8958	1337	1293	0.02%	0.02%	942	1759	1022	1563	*hypothetical protein*	Unknown	Unknown
RCAP_rcc00067	0.16	0.4481	132	149	0.00%	0.00%	107	160	129	168	*Maf-like protein*	Cell Division	Chromosome Partitioning
RCAP_rcc00068	0.11	0.4123	274	298	0.00%	0.00%	241	310	274	321	*aroE*	Amino Acid Metabolism	Phenylalanine, tyrosine and tryptophan biosynthesis
RCAP_rcc00069	-0.17	0.1884	93	83	0.00%	0.00%	86	99	73	93	*coaE*	Metabolism of Cofactors, Coenzymes and Vitamins	Pantothenate and CoA biosynthesis
RCAP_rcc00070	-0.45	0.0089	311	226	0.00%	0.00%	254	358	197	255	*dnaQ*	Replication, Recombination and Repair	Replication
RCAP_rcc00071	0.21	0.3459	840	975	0.01%	0.01%	694	1036	841	1109	*nadA*	Metabolism of Cofactors, Coenzymes and Vitamins	Nicotinate and nicotinamide metabolism
RCAP_rcc00072	0.28	0.1329	327	398	0.00%	0.01%	274	392	345	451	*nadB*	Metabolism of Cofactors, Coenzymes and Vitamins	Nicotinate and nicotinamide metabolism
RCAP_rcc00073	0.06	0.706	333	348	0.00%	0.00%	297	373	306	390	*nadC*	Metabolism of Cofactors, Coenzymes and Vitamins	Nicotinate and nicotinamide metabolism
RCAP_rcc00074	-0.28	0.0856	1891	1548	0.03%	0.02%	1609	2151	1352	1743	*nudH*	Metabolism of Cofactors, Coenzymes and Vitamins	Folate biosynthesis
RCAP_rcc00075	-0.19	0.1522	8000	7014	0.11%	0.10%	7156	8857	6295	7732	*ctpA*	Cell Envelope Biosynthesis	Cell Wall Biosynthesis

RCAP_rcc00076	-0.2	0.0093	1373	1194	0.02%	0.02%	1260	1463	1143	1245	*M23 family peptidase*	Post-translational Modification, Assembly and Chaperones	Peptidase
RCAP_rcc00077	0.12	0.351	3152	3439	0.04%	0.05%	2811	3582	3148	3730	*gpmI*	Carbohydrate Metabolism	Glycolysis / Gluconeogenesis
RCAP_rcc00078	-0.42	0.0115	1204	890	0.02%	0.01%	1037	1357	748	1031	*hypothetical protein*	Unknown	Unknown
RCAP_rcc00079	-0.24	0.1915	8762	7390	0.12%	0.10%	7391	10145	6161	8619	*iojap-related protein*	Unknown	Unknown
RCAP_rcc00080	0.38	0.0458	165	216	0.00%	0.00%	139	199	181	251	*hypothetical protein*	Unknown	Unknown
RCAP_rcc00081	0.34	0.0028	2784	3524	0.04%	0.05%	2735	3100	3113	3935	*leuC*	Amino Acid Metabolism	Valine, leucine and isoleucine biosynthesis
RCAP_rcc00082	-0.25	0.2447	2254	1889	0.03%	0.03%	1805	2719	1594	2184	*leuD*	Amino Acid Metabolism	Valine, leucine and isoleucine biosynthesis
RCAP_rcc00083	0.18	0.3678	1718	1960	0.02%	0.03%	1415	2071	1629	2291	*hypothetical protein*	Unknown	Unknown
RCAP_rcc00084	0.72	0	177	292	0.00%	0.00%	163	202	262	322	*hypothetical protein*	Unknown	Unknown
RCAP_rcc00085	0.01	0.9607	4341	4364	0.06%	0.06%	3949	4792	3969	4759	*leuB*	Amino Acid Metabolism	Valine, leucine and isoleucine biosynthesis
RCAP_rcc00086	-0.39	0	1366	1042	0.02%	0.01%	1216	1459	971	1113	*hypothetical protein*	Unknown	Unknown
RCAP_rcc00087	-0.17	0.4759	88	77	0.00%	0.00%	73	100	58	96	*hypothetical protein*	Unknown	Unknown
RCAP_rcc00088	0.05	0.8869	19	19	0.00%	0.00%	15	22	14	25	*hypothetical protein*	Unknown	Unknown
RCAP_rcc00089	0	0.9946	166	165	0.00%	0.00%	149	184	120	210	*cation diffusion facilitator family transporter*	Metal and Ion Transport	Unknown
RCAP_rcc00090	-1.01	0.0012	199	91	0.00%	0.00%	149	242	51	131	*feoA1*	Metal, Ion, Cofactor Transport	Iron and Heme Transport
RCAP_rcc00091	-0.75	0.0241	139	78	0.00%	0.00%	99	173	44	111	*feoA2*	Metal, Ion, Cofactor Transport	Iron and Heme Transport
RCAP_rcc00092	-1.5	0	2219	738	0.03%	0.01%	1763	2515	474	1001	*feoB1*	Metal, Ion, Cofactor Transport	Iron and Heme Transport
RCAP_rcc00093	-1.62	0	58	15	0.00%	0.00%	32	79	11	20	*hypothetical protein*	Unknown	Unknown
RCAP_rcc00094	-1.73	0	66	18	0.00%	0.00%	46	80	12	23	*hmuV*	Metal, Ion, Cofactor Transport	Iron and Heme Transport
RCAP_rcc00095	-1.47	0	32	10	0.00%	0.00%	21	41	7	13	*hmuU*	Metal, Ion, Cofactor Transport	Iron and Heme Transport
RCAP_rcc00096	-2	NA	21	3	0.00%	0.00%	7	33	2	5	*hmuT*	Metal, Ion, Cofactor Transport	Iron and Heme Transport
RCAP_rcc00097	-2.38	0	102	17	0.00%	0.00%	71	124	13	22	*hmuS*	Metal, Ion, Cofactor Transport	Iron and Heme Transport
RCAP_rcc00098	-1.85	0	556	145	0.01%	0.00%	428	642	108	182	*hmuR*	Metal, Ion, Cofactor Transport	Iron and Heme Transport
RCAP_rcc00099	-2.24	0	249	46	0.00%	0.00%	175	299	32	61	*ABC transporter ATP-binding protein*	Metal, Ion, Cofactor Transport	Nickel Transport
RCAP_rcc00100	-1.92	0	505	106	0.01%	0.00%	289	673	68	145	*ABC transporter ATP-binding protein*	Amino Acid Metabolism	Amino Acid Transport
RCAP_rcc00101	-2.36	0	817	147	0.01%	0.00%	603	952	114	180	*ABC transporter permease*	Amino Acid Metabolism	Amino Acid Transport
RCAP_rcc00102	-2.08	0	680	125	0.01%	0.00%	343	951	77	173	*ABC transporter permease*	Metal, Ion, Cofactor Transport	Nickel Transport
RCAP_rcc00103	-2.67	0	4537	691	0.06%	0.01%	3727	4916	629	752	*ABC transporter periplasmic substrate-binding protein*	Metal, Ion, Cofactor Transport	Nickel Transport
RCAP_rcc00104	-0.75	0	540	317	0.01%	0.00%	468	593	269	365	*hypothetical protein*	Unknown	Unknown
RCAP_rcc00105	-1.07	0.0054	21	8	0.00%	0.00%	10	29	5	12	*fhuC1*	Metal, Ion, Cofactor Transport	Iron and Heme Transport
RCAP_rcc00106	-1.3	0	45	17	0.00%	0.00%	36	51	12	21	*fhuB1*	Metal, Ion, Cofactor Transport	Iron and Heme Transport
RCAP_rcc00107	-0.83	0.0018	44	23	0.00%	0.00%	31	54	15	32	*fhuB2*	Metal, Ion, Cofactor Transport	Iron and Heme Transport
RCAP_rcc00108	-1.46	0	54	17	0.00%	0.00%	27	77	11	24	*fhuD1*	Metal, Ion, Cofactor Transport	Iron and Heme Transport
RCAP_rcc00109	-0.3	0.2992	219	175	0.00%	0.00%	189	246	111	239	*hypothetical protein*	Unknown	Unknown
RCAP_rcc00110	-0.37	0.2878	55	41	0.00%	0.00%	41	65	21	61	*esterase*	Unknown	Unknown
RCAP_rcc00111	-0.3	0.0388	92	74	0.00%	0.00%	81	100	63	84	*fhuE*	Metal, Ion, Cofactor Transport	Iron and Heme Transport
RCAP_rcc00112	0.64	NA	45	77	0.00%	0.00%	33	57	15	139	*AraC family transcriptional regulator*	Signal Transduction	Transcription Regulator
RCAP_rcc00113	0.04	0.7483	1058	1086	0.02%	0.01%	983	1157	1005	1166	*rnd1*	Translation, ribosomal structure and biogenesis	Unknown
RCAP_rcc00114	0.1	0.5881	500	536	0.01%	0.01%	414	586	496	576	*kdsD*	Cell Envelope Biosynthesis	Cell Wall Biosynthesis
RCAP_rcc00115	-0.04	0.7036	243	235	0.00%	0.00%	224	258	216	254	*hypothetical protein*	Unknown	Unknown
RCAP_rcc00116	0.26	0.0023	373	447	0.01%	0.01%	353	406	415	480	*OstA family protein*	Predicted Function	Unknown
RCAP_rcc00117	-0.44	0.0004	688	505	0.01%	0.01%	581	773	469	540	*ABC transporter ATP-binding protein*	Predicted Function	Unknown
RCAP_rcc00118	0.75	0	13029	22084	0.19%	0.30%	11899	15330	19507	24660	*sigma 54 modulation protein/ribosomal protein S30EA*	Translation, ribosomal structure and biogenesis	Unknown
RCAP_rcc00119	0.44	0.0001	2877	3919	0.04%	0.05%	2647	3222	3552	4285	*ptsN*	Signal Transduction	Transcription Regulator
RCAP_rcc00120	0.01	0.9571	884	886	0.01%	0.01%	814	938	832	941	*hypothetical protein*	Unknown	Unknown
RCAP_rcc00121	-0.25	0.1423	1187	994	0.02%	0.01%	996	1356	887	1102	*family 14 glycosyl transferase*	Unknown	Unknown
RCAP_rcc00122	-0.2	0.351	708	613	0.01%	0.01%	525	862	542	683	*hypothetical protein*	Unknown	Unknown
RCAP_rcc00123	-0.47	0.0003	335	240	0.00%	0.00%	287	374	219	261	*hypothetical protein*	Unknown	Unknown
RCAP_rcc00124	-0.22	0.0459	2865	2455	0.04%	0.03%	2570	3135	2265	2645	*galE*	Carbohydrate Metabolism	Galactose Metabolism
RCAP_rcc00125	0.37	0.0035	5155	6678	0.08%	0.09%	4759	5950	5992	7364	*galU*	Carbohydrate Metabolism	Galactose Metabolism
RCAP_rcc00126	-0.26	0.003	1227	1022	0.02%	0.01%	1105	1319	959	1085	*kdsB*	Glycan Biosynthesis and Metabolism	Lipopolysaccharide biosynthesis
RCAP_rcc00127	0.03	0.8389	1767	1806	0.02%	0.02%	1524	2015	1712	1900	*cysQ*	Energy Metabolism	Sulfur metabolism
RCAP_rcc00128	-0.89	0	2417	1276	0.03%	0.02%	1904	2829	1082	1471	*ABC transporter permease*	Unknown	Unknown
RCAP_rcc00129	0.16	0.0043	1542	1730	0.02%	0.02%	1461	1630	1671	1789	*LacI family transcriptional regulator*	Signal Transduction	Transcription Regulator
RCAP_rcc00130	-0.25	0.1114	2143	1799	0.03%	0.02%	1852	2404	1526	2072	*radical SAM family protein*	Energy Metabolism	Unknown
RCAP_rcc00131	0	0.9973	334	334	0.00%	0.00%	236	427	258	410	*tRNA/rRNA cytosine-C5-methylase*	Translation, ribosomal structure and biogenesis	Unknown
RCAP_rcc00132	2.35	0	131	834	0.00%	0.01%	131	223	559	1109	*hypothetical protein*	Unknown	Unknown
RCAP_rcc00133	1.49	0	2021	5840	0.03%	0.08%	2202	2585	4958	6723	*hypothetical protein*	Unknown	Unknown
RCAP_rcc00134	1.44	0	12	39	0.00%	0.00%	10	19	25	53	*hypothetical protein*	Unknown	Unknown
RCAP_rcc00135	0.88	0.029	3	6	0.00%	0.00%	2	4	3	8	*lolD1*	Predicted Function	Unknown
RCAP_rcc00136	0.7	0.0013	133	222	0.00%	0.00%	105	186	186	258	*phoA*	Metabolism of Cofactors, Coenzymes and Vitamins	Folate biosynthesis
RCAP_rcc00137	0.59	0.0022	14	22	0.00%	0.00%	13	18	19	25	*hypothetical protein*	Unknown	Unknown
RCAP_rcc00138	0.42	0.0001	132	175	0.00%	0.00%	122	148	161	190	*dnaE2*	Replication, Recombination and Repair	Replication
RCAP_rcc00139	1.61	0	14	56	0.00%	0.00%	10	20	27	86	*nucleotidyltransferase/DNA polymerase involved in DNA repair*	Replication, Recombination and Repair	Repair
RCAP_rcc00140	0.12	0.5055	159	173	0.00%	0.00%	136	183	149	196	*lipoprotein*	Predicted Function	Unknown
RCAP_rcc00141	0.41	0.0088	2048	2744	0.03%	0.04%	1918	2335	2258	3230	*hbdA*	Carbohydrate Metabolism	Butanoate metabolism
RCAP_rcc00142	-1.02	0	1971	956	0.03%	0.01%	1611	2230	840	1071	*hypothetical protein*	Unknown	Unknown
RCAP_rcc00143	-0.26	0.1523	3964	3287	0.05%	0.04%	3170	4650	2976	3599	*etfA*	Energy Metabolism	Aerobic/Anaerobic Metabolism
RCAP_rcc00144	0.01	0.9525	4477	4512	0.06%	0.06%	3902	5133	4065	4959	*etfB*	Energy Metabolism	Aerobic/Anaerobic Respiration
RCAP_rcc00145	-0.28	0.0571	359	296	0.01%	0.00%	313	406	261	330	*short-chain dehydrogenase/reductase family oxidoreductase*	Lipid transport and metabolism	Unknown
RCAP_rcc00146	-0.04	0.8082	2052	1992	0.03%	0.03%	1792	2309	1760	2224	*parC*	Replication, Recombination and Repair	Unknown
RCAP_rcc00147	-0.13	0.511	40136	36672	0.57%	0.50%	33715	46792	32294	41050	*tufI*	Translation, ribosomal structure and biogenesis	Unknown
RCAP_rcc00148	0.02	0.9236	622	632	0.01%	0.01%	495	732	552	711	*CDA peptide synthetase III*	Lipid Metabolism	Fatty acid metabolism
RCAP_rcc00149	0.45	0.0001	195	267	0.00%	0.00%	181	223	245	289	*hypothetical protein*	Unknown	Unknown
RCAP_rcc00150	0.57	0.0248	94	143	0.00%	0.00%	71	116	106	180	*hypothetical protein*	Unknown	Unknown
RCAP_rcc00151	1.47	0.0003	169	592	0.00%	0.01%	63	313	293	892	*hemN1*	Metabolism of Cofactors, Coenzymes and Vitamins	Heme Biosynthesis
RCAP_rcc00152	-3.29	0	451	41	0.01%	0.00%	328	530	37	46	*peptide ABC transporter periplasmic peptide-binding protein*	Amino Acid Metabolism	Amino Acid Transport

RCAP_rcc00153	-3.09	0	208	22	0.00%	0.00%	156	240	18	25	*peptide ABC transporter permease*	Amino Acid Metabolism	Amino Acid Transport
RCAP_rcc00154	-1.96	0	77	17	0.00%	0.00%	45	103	14	21	*peptide ABC transporter permease*	Amino Acid Metabolism	Amino Acid Transport
RCAP_rcc00155	-1.95	0	151	32	0.00%	0.00%	79	208	24	41	*peptide ABC transporter ATP-binding protein*	Predicted Function	Nickel Transport
RCAP_rcc00156	1.03	0	2179	4527	0.03%	0.06%	1931	2696	4188	4866	*alsT*	Amino Acid Metabolism	Unknown
RCAP_rcc00157	0.26	0.001	1625	1954	0.02%	0.03%	1524	1741	1821	2087	*hypothetical protein*	Unknown	Unknown
RCAP_rcc00158	-0.39	0.0137	17074	12939	0.24%	0.18%	14612	19273	11160	14719	*M10 family peptidase*	Post-translational Modification, Assembly and Chaperones	Peptidase
RCAP_rcc00159	-0.12	0.4165	1927	1773	0.03%	0.02%	1684	2137	1576	1971	*hypothetical protein*	Unknown	Unknown
RCAP_rcc00160	-0.31	0.0002	424	342	0.01%	0.00%	388	443	319	364	*mtgA*	Nucleotide Metabolism	Purine metabolism
RCAP_rcc00161	-0.11	0.5167	6275	5792	0.09%	0.08%	5604	6891	4748	6836	*gltB*	Energy Metabolism	Nitrogen metabolism
RCAP_rcc00162	-0.18	0.3448	481	424	0.01%	0.01%	424	534	346	502	*hypothetical protein*	Unknown	Unknown
RCAP_rcc00163	-0.11	0.3031	1004	930	0.01%	0.01%	967	1040	829	1031	*gltD*	Energy Metabolism	Nitrogen metabolism
RCAP_rcc00164	-0.7	0	2331	1420	0.03%	0.02%	1924	2642	1271	1568	*uppP*	Glycan Biosynthesis and Metabolism	Peptidoglycan biosynthesis
RCAP_rcc00165	-0.03	0.7707	2275	2222	0.03%	0.03%	2096	2516	2066	2378	*NAD-dependent epimerase/dehydratase*	Carbohydrate Metabolism	Unknown
RCAP_rcc00166	-0.47	0.0354	4223	3004	0.06%	0.04%	3303	5092	2417	3590	*sulfotransferase*	Unknown	Unknown
RCAP_rcc00167	-0.14	0.2464	11067	10037	0.16%	0.14%	10104	12027	9146	10927	*family 2 glycosyl transferase*	Cell Envelope Biosynthesis	Cell Wall Biosynthesis
RCAP_rcc00168	0.34	0.0354	551	699	0.01%	0.01%	484	647	612	787	*ABC transporter polysaccharide inner membrane subunit*	Carbohydrate Metabolism	Unknown
RCAP_rcc00169	-0.2	0.2588	2898	2517	0.04%	0.03%	2406	3352	2211	2823	*kpsE1*	Cell Envelope Biosynthesis	Cell Wall Biosynthesis
RCAP_rcc00170	-0.28	0.1368	834	683	0.01%	0.01%	674	983	607	759	*kpsT1*	Carbohydrate Metabolism	Unknown
RCAP_rcc00171	-0.32	0.0998	72	57	0.00%	0.00%	61	81	46	67	*hypothetical protein*	Unknown	Unknown
RCAP_rcc00172	0.22	0.3929	483	567	0.01%	0.01%	366	593	474	661	*glycosyl transferase*	Cell Envelope Biosynthesis	Cell Wall Biosynthesis
RCAP_rcc00173	0	0.9984	2184	2186	0.03%	0.03%	2078	2331	1967	2406	*rfbA*	Cell Envelope Biosynthesis	Cell Wall Biosynthesis
RCAP_rcc00174	0.09	0.7632	472	506	0.01%	0.01%	331	601	396	615	*rfbD*	Cell Envelope Biosynthesis	Cell Wall Biosynthesis
RCAP_rcc00175	-0.02	0.874	842	828	0.01%	0.01%	741	924	773	883	*hypothetical protein*	Unknown	Unknown
RCAP_rcc00176	0.11	0.3949	2918	3148	0.04%	0.04%	2640	3221	2842	3454	*rfbB*	Cell Envelope Biosynthesis	Cell Wall Biosynthesis
RCAP_rcc00177	-0.18	0.2934	2089	1834	0.03%	0.02%	1792	2368	1561	2107	*rfbC*	Cell Envelope Biosynthesis	Cell Wall Biosynthesis
RCAP_rcc00178	-0.58	0.0016	679	447	0.01%	0.01%	546	784	382	511	*hemolysin-type calcium-binding repeat family protein*	Trafficking and Secretion	Secretion
RCAP_rcc00179	-0.37	0	1915	1477	0.03%	0.02%	1805	1965	1341	1612	*ilvA*	Amino Acid Metabolism	Valine, leucine and isoleucine biosynthesis
RCAP_rcc00180	-1.08	0	2357	1097	0.03%	0.01%	1940	2636	948	1246	*Hpt domain-containing protein*	Unknown	Unknown
RCAP_rcc00181	-0.45	0	1001	732	0.01%	0.01%	891	1078	665	799	*response regulator receiver domain/protein phosphatase 2C domain-co*	Signal Transduction	Transcription Regulator
RCAP_rcc00182	-0.72	0.0002	2054	1224	0.03%	0.02%	1673	2353	1024	1424	*hemolysin D*	Trafficking and Secretion	Secretion
RCAP_rcc00183	-0.28	0.0068	2010	1656	0.03%	0.02%	1774	2203	1567	1745	*ABC transporter ATP-binding/permease*	Defense Mechanisms	Unknown
RCAP_rcc00184	-0.76	0	1861	1092	0.03%	0.01%	1579	2056	974	1210	*outer membrane efflux protein*	Cell Envelope Biosynthesis	Cell Wall Biosynthesis
RCAP_rcc00185	-0.22	0.1115	17021	14557	0.24%	0.20%	14923	18972	12976	16137	*hypothetical protein*	Unknown	Unknown
RCAP_rcc00186	-0.29	0.0218	232	188	0.00%	0.00%	208	246	164	213	*NUDIX superfamily hydrolase*	Unknown	Unknown
RCAP_rcc00187	0.66	0	1519	2423	0.02%	0.03%	1425	1805	2149	2698	*hslO*	Post-translational Modification, Assembly and Chaperones	Unknown
RCAP_rcc00188	0.57	0.0755	140	215	0.00%	0.00%	100	182	157	274	*NUDIX superfamily hydrolase*	Replication, Recombination and Repair	Unknown
RCAP_rcc00189	0.19	0.1304	416	474	0.01%	0.01%	372	464	431	517	*cca*	Translation, ribosomal structure and biogenesis	Unknown
RCAP_rcc00190	-0.17	0.0016	2582	2298	0.04%	0.03%	2462	2691	2201	2394	*ABC transporter ATP-binding/permease*	Defense Mechanisms	Unknown
RCAP_rcc00191	-0.19	0.4126	360	313	0.00%	0.00%	275	429	263	363	*tRNA (Uracil-5-)-methyltransferase*	Translation, ribosomal structure and biogenesis	Unknown
RCAP_rcc00192	-0.34	0.0521	1079	845	0.02%	0.01%	887	1256	727	963	*ErfK/YbiS/YcfS/YnhG family protein*	Unknown	Unknown
RCAP_rcc00193	-0.29	0.213	535	434	0.01%	0.01%	409	668	379	489	*SCP-like extracellular protein*	Unknown	Unknown
RCAP_rcc00194	0.21	0.337	1505	1758	0.02%	0.02%	1252	1908	1522	1994	*ErfK/YbiS/YcfS/YnhG family protein/Tat domain-containing protein*	Unknown	Unknown
RCAP_rcc00195	0.25	0.1123	2117	2523	0.03%	0.03%	1906	2460	2199	2846	*hemH*	Metabolism of Cofactors, Coenzymes and Vitamins	Heme Biosynthesis
RCAP_rcc00196	0.7	0	702	1145	0.01%	0.02%	682	782	1040	1250	*hypothetical protein*	Unknown	Unknown
RCAP_rcc00197	-0.06	0.89	39	37	0.00%	0.00%	24	52	24	51	*comF*	Unknown	Unknown
RCAP_rcc00198	0.8	0	218	386	0.00%	0.01%	183	263	335	437	*grxC*	Post-translational Modification, Assembly and Chaperones	Unknown
RCAP_rcc00199	0.54	0	589	859	0.01%	0.01%	545	663	812	906	*carbon-nitrogen family hydrolase*	Predicted Function	Nitrogen metabolism
RCAP_rcc00200	0.65	0	246	388	0.00%	0.01%	237	275	337	439	*MarR family transcriptional regulator*	Signal Transduction	Transcription Regulator
RCAP_rcc00201	-0.29	0.1631	6512	5277	0.09%	0.07%	5104	7811	4383	6172	*adenine-specific DNA-methyltransferase*	Replication, Recombination and Repair	Unknown
RCAP_rcc00202	-0.47	0.0023	332	239	0.00%	0.00%	285	365	200	277	*rnhB*	Replication, Recombination and Repair	Unknown
RCAP_rcc00203	-0.48	NA	282	172	0.00%	0.00%	58	490	69	276	*hypothetical protein*	Unknown	Unknown
RCAP_rcc00204	0.35	0.1043	234	300	0.00%	0.00%	188	295	259	341	*hypothetical protein*	Unknown	Unknown
RCAP_rcc00205	-0.07	0.7082	767	731	0.01%	0.01%	660	876	631	830	*hypothetical protein*	Unknown	Unknown
RCAP_rcc00206	-0.57	0.026	2134	1399	0.03%	0.02%	1590	2659	1111	1686	*nspC*	Amino Acid Metabolism	Unknown
RCAP_rcc00207	-0.28	0.274	3372	2747	0.05%	0.04%	2673	4084	2134	3361	*lys1*	Amino Acid Metabolism	Unknown
RCAP_rcc00208	-0.13	0.5747	5248	4788	0.07%	0.07%	4239	6253	4085	5491	*argG*	Amino Acid Metabolism	Arginine and proline metabolism
RCAP_rcc00209	0.19	0.0863	401	459	0.01%	0.01%	366	438	415	503	*mogA*	Unknown	Unknown
RCAP_rcc00210	0.96	0	1106	2175	0.02%	0.03%	1091	1325	1855	2494	*ubiG*	Energy Metabolism	Biosynthesis of Ubiquinone
RCAP_rcc00211	0.64	0	1469	2300	0.02%	0.03%	1378	1615	2177	2423	*pip1*	Amino Acid Metabolism	Arginine and proline metabolism
RCAP_rcc00212	-0.29	0.1565	4046	3277	0.06%	0.04%	3298	4852	2744	3811	*hypothetical protein*	Unknown	Unknown
RCAP_rcc00213	-0.33	0.0421	8948	7067	0.13%	0.10%	7870	9990	5913	8221	*nusA*	Transcription	Unknown
RCAP_rcc00214	-0.02	0.9264	2253	2222	0.03%	0.03%	2029	2494	1804	2639	*hypothetical protein*	Unknown	Unknown
RCAP_rcc00215	0.03	0.8233	10539	10779	0.15%	0.15%	9735	11412	9400	12157	*infB*	Translation, ribosomal structure and biogenesis	Unknown
RCAP_rcc00216	-1.89	0	8404	2110	0.11%	0.03%	6266	9978	1687	2534	*hypothetical protein*	Unknown	Unknown
RCAP_rcc00217	-0.59	0	529	350	0.01%	0.00%	471	561	321	379	*nudG*	Metabolism of Cofactors, Coenzymes and Vitamins	Folate biosynthesis
RCAP_rcc00218	-0.35	0.0001	3490	2725	0.05%	0.04%	3169	3745	2549	2901	*argJ*	Amino Acid Metabolism	Arginine and proline metabolism
RCAP_rcc00219	-0.06	0.6252	2366	2278	0.03%	0.03%	2170	2592	2104	2452	*PpiC-type peptidyl-prolyl cis-trans isomerase*	Post-translational Modification, Assembly and Chaperones	Unknown
RCAP_rcc00220	0.37	0.0492	9391	12195	0.14%	0.17%	7946	11469	10646	13744	*secA*	Trafficking and Secretion	Secretion
RCAP_rcc00221	0.27	0.3655	135	165	0.00%	0.00%	94	174	119	210	*hypothetical protein*	Unknown	Unknown
RCAP_rcc00222	0.26	0.0792	498	599	0.01%	0.01%	435	569	548	649	*radC*	Replication, Recombination and Repair	Repair
RCAP_rcc00223	0.01	0.9664	5409	5435	0.08%	0.07%	4619	6257	5066	5805	*dnaJ*	Post-translational Modification, Assembly and Chaperones	Unknown
RCAP_rcc00224	0.66	0.0007	27557	44250	0.41%	0.60%	23333	35236	38403	50097	*dnaK*	Post-translational Modification, Assembly and Chaperones	Unknown
RCAP_rcc00225	-0.56	0	163	109	0.00%	0.00%	146	173	101	117	*alkB*	Replication, Recombination and Repair	Unknown
RCAP_rcc00226	-0.71	0.0112	172	101	0.00%	0.00%	112	223	76	126	*mutY*	Replication, Recombination and Repair	Unknown
RCAP_rcc00227	0.95	0.0014	387	797	0.01%	0.01%	280	510	535	1058	*hypothetical protein*	Unknown	Unknown
RCAP_rcc00228	-0.18	0.2719	3401	2995	0.05%	0.04%	2905	3933	2634	3355	*DSBA family oxidoreductase*	Post-translational Modification, Assembly and Chaperones	Unknown
RCAP_rcc00229	0.09	0.7595	508	542	0.01%	0.01%	383	636	469	615	*lpxK*	Glycan Biosynthesis and Metabolism	Lipopolysaccharide biosynthesis

RCAP_rcc00230	-0.22	0.6088	522	437	0.01%	0.01%	260	746	260	614	*kdtA1*	Energy Metabolism	Puromycin biosynthesis
RCAP_rcc00231	-0.01	0.9723	1328	1322	0.02%	0.02%	1111	1569	1138	1505	*hypothetical protein*	Unknown	Unknown
RCAP_rcc00232	0.39	0.0033	275	364	0.00%	0.00%	252	303	316	412	*xylose isomerase-like TIM barrel family protein*	Carbohydrate Metabolism	Unknown
RCAP_rcc00233	-0.11	0.573	1304	1209	0.02%	0.02%	1076	1507	1076	1343	*AFG1-like ATPase*	Unknown	Unknown
RCAP_rcc00234	1.23	0	201	497	0.00%	0.01%	174	242	317	676	*luciferase*	Energy Metabolism	Unknown
RCAP_rcc00235	0.44	0.001	721	985	0.01%	0.01%	654	833	887	1083	*folC*	Metabolism of Cofactors, Coenzymes and Vitamins	Folate biosynthesis
RCAP_rcc00236	0.84	0	1497	2732	0.02%	0.04%	1304	1881	2397	3067	*accD*	Energy Metabolism	Reductive carboxylate cycle (CO2 fixation)
RCAP_rcc00237	0.37	0.0593	179	234	0.00%	0.00%	147	216	207	261	*abortive infection protein family*	Unknown	Unknown
RCAP_rcc00238	-0.02	0.9558	178	176	0.00%	0.00%	132	215	142	210	*hypothetical protein*	Unknown	Unknown
RCAP_rcc00239	0.23	0.093	216	253	0.00%	0.00%	191	240	225	282	*ansA*	Amino Acid Metabolism	Unknown
RCAP_rcc00240	0.12	0.3206	4743	5149	0.07%	0.07%	4369	5231	4680	5617	*hypothetical protein*	Unknown	Unknown
RCAP_rcc00241	-0.04	0.7511	1574	1525	0.02%	0.02%	1463	1746	1351	1699	*radical SAM family protein*	Translation, ribosomal structure and biogenesis	Unknown
RCAP_rcc00242	-0.38	0.0023	762	584	0.01%	0.01%	673	835	517	650	*hypothetical protein*	Unknown	Unknown
RCAP_rcc00243	-0.22	0.1035	1959	1674	0.03%	0.02%	1732	2171	1502	1845	*dapD*	Amino Acid Metabolism	Lysine biosynthesis
RCAP_rcc00244	-0.1	0.7376	191	177	0.00%	0.00%	137	239	153	201	*hypothetical protein*	Unknown	Unknown
RCAP_rcc00245	-0.36	0.0033	633	492	0.01%	0.01%	567	677	437	546	*hypothetical protein*	Unknown	Unknown
RCAP_rcc00246	0.03	0.7697	1970	2015	0.03%	0.03%	1789	2186	1916	2114	*mltB*	Glycan Biosynthesis and Metabolism	Glycosaminoglycan degradation
RCAP_rcc00247	-1.01	0	390	192	0.01%	0.00%	315	442	179	204	*NnrU family protein*	Energy Metabolism	Nitrogen Metabolism
RCAP_rcc00248	-0.61	0.0105	568	362	0.01%	0.00%	399	711	317	407	*hypothetical protein*	Unknown	Unknown
RCAP_rcc00249	-0.05	0.7314	1932	1858	0.03%	0.03%	1664	2189	1665	2051	*hup1*	Energy Metabolism	Aerobic/Anaerobic Respiration
RCAP_rcc00250	-0.45	0.0004	3268	2384	0.05%	0.03%	2798	3685	2162	2607	*amn*	Nucleotide Metabolism	Purine metabolism
RCAP_rcc00251	-0.2	0.0635	3742	3242	0.05%	0.04%	3271	4135	3011	3472	*ade*	Nucleotide Metabolism	Purine metabolism
RCAP_rcc00252	-0.37	0.022	761	583	0.01%	0.01%	625	876	514	653	*NAD-dependent epimerase/dehydratase*	Carbohydrate Metabolism	Unknown
RCAP_rcc00253	-1.2	0	5925	2510	0.08%	0.03%	4692	6836	2149	2871	*major facilitator superfamily protein*	Carbohydrate Metabolism	Unknown
RCAP_rcc00254	-0.47	0.0096	206	146	0.00%	0.00%	170	228	118	175	*hypothetical protein*	Unknown	Unknown
RCAP_rcc00255	-0.56	0	601	405	0.01%	0.01%	521	653	360	450	*polB*	Replication, Recombination and Repair	Replication
RCAP_rcc00256	0.16	0.3432	513	573	0.01%	0.01%	438	586	513	633	*lipoprotein*	Predicted Function	Unknown
RCAP_rcc00257	-0.09	0.6034	6085	5702	0.09%	0.08%	5319	6926	5010	6394	*leuS*	Translation, ribosomal structure and biogenesis	Aminoacyl-tRNA biosynthesis
RCAP_rcc00258	0.27	0.01	1144	1381	0.02%	0.02%	1027	1297	1307	1456	*lipoprotein*	Predicted Function	Unknown
RCAP_rcc00259	0.53	0	211311	305332	3.09%	4.15%	206326	233999	282391	328274	*porin family protein*	Cell Envelope Biosynthesis	Cell Wall Biosynthesis
RCAP_rcc00260	0.49	0.0024	243	343	0.00%	0.00%	205	289	306	380	*alanine racemase domain-containing protein*	Unknown	Unknown
RCAP_rcc00261	-0.05	0.7878	145	140	0.00%	0.00%	123	166	128	153	*hypothetical protein*	Unknown	Unknown
RCAP_rcc00262	0.02	0.8753	502	508	0.01%	0.01%	468	531	450	566	*ribA*	Metabolism of Cofactors, Coenzymes and Vitamins	Riboflavin metabolism
RCAP_rcc00263	0.9	0	3415	6491	0.05%	0.09%	3117	4334	5520	7461	*winged helix family two component transcriptional regulator*	Signal Transduction	Transcription Regulator
RCAP_rcc00264	0.61	0	1365	2096	0.02%	0.03%	1262	1526	1983	2210	*hypothetical protein*	Unknown	Unknown
RCAP_rcc00265	-0.35	0.0245	702	547	0.01%	0.01%	628	759	444	651	*RmuC domain-containing protein*	Unknown	Unknown
RCAP_rcc00266	-0.38	0.0396	1009	770	0.01%	0.01%	809	1168	679	860	*mutL*	Replication, Recombination and Repair	Repair
RCAP_rcc00267	-0.37	0	2999	2318	0.04%	0.03%	2793	3164	2143	2493	*M16 family peptidase*	Post-translational Modification, Assembly and Chaperones	Peptidase
RCAP_rcc00268	-0.33	0.0025	2319	1842	0.03%	0.03%	2090	2536	1674	2010	*M16 family peptidase*	Post-translational Modification, Assembly and Chaperones	Peptidase
RCAP_rcc00269	0.29	0.1208	669	821	0.01%	0.01%	548	796	730	912	*lipoprotein*	Predicted Function	Unknown
RCAP_rcc00270	-0.09	0.6291	620	582	0.01%	0.01%	537	700	501	663	*lspA*	Cell Envelope Biosynthesis	Cell Wall Biosynthesis
RCAP_rcc00271	0.23	0.1222	1677	1972	0.02%	0.03%	1499	1908	1755	2189	*purH*	Metabolism of Cofactors, Coenzymes and Vitamins	One carbon pool by folate
RCAP_rcc00272	-0.23	0.0484	478	406	0.01%	0.01%	424	516	376	436	*heparinase II/III family protein*	Unknown	Unknown
RCAP_rcc00273	-0.34	0.129	227	177	0.00%	0.00%	165	278	154	201	*rsmB1*	Translation, ribosomal structure and biogenesis	Unknown
RCAP_rcc00274	0.67	0.0057	154	250	0.00%	0.00%	123	190	176	324	*hypothetical protein*	Unknown	Unknown
RCAP_rcc00275	0.2	0.3509	458	530	0.01%	0.01%	354	557	436	624	*dapB*	Amino Acid Metabolism	Unknown
RCAP_rcc00276	0.61	0	622	951	0.01%	0.01%	596	685	868	1034	*rbfA*	Translation, ribosomal structure and biogenesis	Unknown
RCAP_rcc00277	0.64	0.0001	361	569	0.01%	0.01%	322	426	487	651	*hypothetical protein*	Unknown	Unknown
RCAP_rcc00278	0.42	0.0255	470	635	0.01%	0.01%	389	572	558	713	*truB*	Nucleotide Metabolism	Pyrimidine metabolism
RCAP_rcc00279	-0.06	0.7497	165	157	0.00%	0.00%	141	182	137	177	*hypothetical protein*	Unknown	Unknown
RCAP_rcc00280	-0.01	0.9371	4182	4143	0.06%	0.06%	3693	4741	3733	4553	*hemolysin-type calcium-binding repeat family protein*	Trafficking and Secretion	Secretion
RCAP_rcc00281	-0.3	0.2362	6915	5532	0.10%	0.08%	5064	8629	4426	6638	*rpsO*	Translation, ribosomal structure and biogenesis	Unknown
RCAP_rcc00282	-0.42	0.0568	1376	1017	0.02%	0.01%	1048	1623	807	1227	*aldehyde dehydrogenase*	Carbohydrate Metabolism	Glycolysis / Gluconeogenesis
RCAP_rcc00283	3.03	0	202	2136	0.00%	0.03%	226	366	1417	2855	*hypothetical protein*	Unknown	Unknown
RCAP_rcc00284	-0.33	0.0085	35251	27871	0.49%	0.38%	30707	39426	25042	30700	*pnp*	Nucleotide Metabolism	Pyrimidine metabolism
RCAP_rcc00285	-0.43	0.1054	2895	2104	0.04%	0.03%	2021	3698	1697	2511	*secE*	Trafficking and Secretion	Secretion
RCAP_rcc00286	-0.67	0.0022	9088	5596	0.13%	0.08%	7181	10657	4416	6775	*nusG*	Transcription	Unknown
RCAP_rcc00287	-0.27	0.247	17321	14236	0.24%	0.19%	13999	20507	11013	17459	*rplK*	Translation, ribosomal structure and biogenesis	Unknown
RCAP_rcc00288	-0.27	0.2167	18595	15319	0.26%	0.21%	15421	21474	11903	18735	*rplA*	Translation, ribosomal structure and biogenesis	Unknown
RCAP_rcc00289	-0.27	0.3289	61844	50638	0.87%	0.69%	48285	75655	35876	65400	*rplJ*	Translation, ribosomal structure and biogenesis	Unknown
RCAP_rcc00290	-0.27	0.2442	34317	28154	0.48%	0.38%	27662	40886	21856	34452	*rplL*	Translation, ribosomal structure and biogenesis	Unknown
RCAP_rcc00291	-0.43	0.0233	43943	32301	0.61%	0.44%	36458	50818	26662	37941	*rpoB*	Replication, Recombination and Repair	Replication
RCAP_rcc00292	-0.28	0.1393	51581	42085	0.72%	0.57%	42837	60011	34656	49514	*rpoC*	Replication, Recombination and Repair	Replication
RCAP_rcc00293	-0.03	0.9157	943	926	0.01%	0.01%	756	1123	792	1060	*hypothetical protein*	Unknown	Unknown
RCAP_rcc00294	-0.08	0.7335	13190	12468	0.19%	0.17%	11317	15294	9962	14975	*rpsL*	Translation, ribosomal structure and biogenesis	Unknown
RCAP_rcc00295	-0.12	0.5064	8289	7626	0.12%	0.10%	7255	9337	6428	8823	*rpsG*	Translation, ribosomal structure and biogenesis	Unknown
RCAP_rcc00296	-0.24	0.299	78928	66310	1.11%	0.90%	64448	93470	51003	81618	*fusA1*	Translation, ribosomal structure and biogenesis	Unknown
RCAP_rcc00297	-0.15	0.3943	45634	40883	0.64%	0.56%	39054	52222	34911	46854	*tuf2*	Translation, ribosomal structure and biogenesis	Unknown
RCAP_rcc00298	-0.4	0.1079	13567	10088	0.19%	0.14%	10193	16720	7905	12272	*rpsJ*	Translation, ribosomal structure and biogenesis	Unknown
RCAP_rcc00299	0.06	0.7688	12993	13611	0.19%	0.18%	11553	14976	10875	16348	*rplC*	Translation, ribosomal structure and biogenesis	Unknown
RCAP_rcc00300	-0.01	0.9527	15836	15669	0.23%	0.21%	13908	18145	12217	19121	*rplD*	Translation, ribosomal structure and biogenesis	Unknown
RCAP_rcc00301	-0.43	0.1129	17154	12436	0.24%	0.17%	12444	21637	9167	15704	*rplW*	Translation, ribosomal structure and biogenesis	Unknown
RCAP_rcc00302	-0.32	0.1696	28353	22476	0.40%	0.31%	23102	33330	17346	27606	*rplB*	Translation, ribosomal structure and biogenesis	Unknown
RCAP_rcc00303	-0.49	0.0766	10928	7557	0.15%	0.10%	7750	13857	5585	9528	*rpsS*	Translation, ribosomal structure and biogenesis	Unknown
RCAP_rcc00304	-0.32	0.225	13490	10615	0.19%	0.14%	10425	16444	7810	13419	*rplV*	Translation, ribosomal structure and biogenesis	Unknown
RCAP_rcc00305	-0.24	0.27	18672	15663	0.26%	0.21%	15624	21644	12115	19212	*rpsC*	Translation, ribosomal structure and biogenesis	Unknown
RCAP_rcc00306	0.1	0.6674	6988	7497	0.10%	0.10%	5993	8238	5953	9041	*rplP*	Translation, ribosomal structure and biogenesis	Unknown

RCAP_rcc00307	0.36	0.1949	583	764	0.01%	0.01%	433	726	599	929	*hypothetical protein*	Unknown	Unknown
RCAP_rcc00308	-0.63	0.0114	5673	3571	0.08%	0.05%	4347	6814	2778	4363	*rpmC*	Translation, ribosomal structure and biogenesis	Unknown
RCAP_rcc00309	-0.63	0.0214	10132	6357	0.14%	0.09%	7440	12538	4747	7967	*rpsQ*	Translation, ribosomal structure and biogenesis	Unknown
RCAP_rcc00310	-0.3	0.2279	9056	7288	0.13%	0.10%	7551	10522	5523	9053	*rplN*	Translation, ribosomal structure and biogenesis	Unknown
RCAP_rcc00311	-0.17	0.4588	5829	5147	0.08%	0.07%	5081	6649	4019	6275	*rplX*	Translation, ribosomal structure and biogenesis	Unknown
RCAP_rcc00312	-0.39	0.1584	20514	15288	0.29%	0.21%	15558	25269	11141	19436	*rplE*	Translation, ribosomal structure and biogenesis	Unknown
RCAP_rcc00313	-0.48	0.1032	11137	7772	0.16%	0.11%	8068	14086	5437	10106	*rpsN*	Translation, ribosomal structure and biogenesis	Unknown
RCAP_rcc00314	-0.39	0.1651	14499	10856	0.20%	0.15%	11034	17974	7698	14015	*rpsH*	Translation, ribosomal structure and biogenesis	Unknown
RCAP_rcc00315	-0.27	0.3078	21471	17574	0.30%	0.24%	17005	26052	12914	22233	*rplF*	Translation, ribosomal structure and biogenesis	Unknown
RCAP_rcc00316	-0.4	0.1383	18843	14064	0.26%	0.19%	14180	23299	10510	17618	*rplR*	Translation, ribosomal structure and biogenesis	Unknown
RCAP_rcc00317	0.01	0.9582	13220	13337	0.19%	0.18%	11385	15320	10611	16064	*rpsE*	Translation, ribosomal structure and biogenesis	Unknown
RCAP_rcc00318	-0.18	0.4353	4150	3636	0.06%	0.05%	3489	4807	2782	4490	*rpmD*	Translation, ribosomal structure and biogenesis	Unknown
RCAP_rcc00319	-0.46	0.0185	1801	1295	0.02%	0.02%	1427	2100	1056	1535	*hypothetical protein*	Unknown	Unknown
RCAP_rcc00320	-1.06	0.0001	601	271	0.01%	0.00%	402	759	206	337	*hypothetical protein*	Unknown	Unknown
RCAP_rcc00321	-0.32	0.1342	9768	7741	0.14%	0.11%	7896	11543	6328	9155	*rplO*	Translation, ribosomal structure and biogenesis	Unknown
RCAP_rcc00322	-0.17	0.4408	10530	9320	0.15%	0.13%	8468	12660	7896	10744	*secY*	Trafficking and Secretion	Secretion
RCAP_rcc00323	0.15	0.2124	2585	2883	0.04%	0.04%	2410	2872	2588	3179	*adk*	Nucleotide Metabolism	Purine metabolism
RCAP_rcc00324	-0.22	0.328	11389	9721	0.16%	0.13%	9240	13696	8096	11347	*rpsM*	Translation, ribosomal structure and biogenesis	Unknown
RCAP_rcc00325	-0.05	0.7736	7042	6788	0.10%	0.09%	6309	7948	5859	7717	*rpsK*	Translation, ribosomal structure and biogenesis	Unknown
RCAP_rcc00326	-0.65	0.006	25771	16052	0.36%	0.22%	19747	30997	12663	19442	*rpoA*	Replication, Recombination and Repair	Replication
RCAP_rcc00327	-0.28	0.2241	8804	7189	0.12%	0.10%	7353	10184	5482	8895	*rplQ*	Translation, ribosomal structure and biogenesis	Unknown
RCAP_rcc00328	-0.32	0.0457	6152	4891	0.08%	0.07%	5235	6858	4192	5589	*LuxR family autoinducer-binding transcriptional regulator*	Signal Transduction	Transcription Regulator
RCAP_rcc00329	-1.06	0	3443	1620	0.05%	0.02%	2876	3801	1367	1874	*autoinducer synthesis protein*	Unknown	Unknown
RCAP_rcc00330	0.32	0	586	735	0.01%	0.01%	562	617	694	776	*ATPase AAA*	Replication, Recombination and Repair	Unknown
RCAP_rcc00331	-0.26	0.207	496	412	0.01%	0.01%	397	580	345	480	*crcB*	Cell Division	Chromosome Partitioning
RCAP_rcc00332	-0.13	0.1568	1004	915	0.01%	0.01%	930	1062	837	992	*rluC*	Nucleotide Metabolism	Pyrimidine metabolism
RCAP_rcc00333	-0.06	0.612	252	241	0.00%	0.00%	228	273	220	262	*gph1*	Carbohydrate Metabolism	Glyoxylate and dicarboxylate metabolism
RCAP_rcc00334	0	0.9985	237	236	0.00%	0.00%	173	292	193	280	*ATP12 chaperone protein family*	Post-translational Modification, Assembly and Chaperones	Unknown
RCAP_rcc00335	-0.41	0.0133	9735	7248	0.13%	0.10%	7864	11216	6306	8190	*bztA*	Amino Acid Metabolism	Amino Acid Transport
RCAP_rcc00336	-0.56	0.0027	1710	1145	0.02%	0.02%	1347	1999	972	1318	*bztB*	Amino Acid Metabolism	Amino Acid Transport
RCAP_rcc00337	-0.36	0.1133	793	612	0.01%	0.01%	623	932	448	776	*bztC*	Amino Acid Metabolism	Amino Acid Transport
RCAP_rcc00338	-0.26	0.2424	2280	1886	0.03%	0.03%	1818	2707	1509	2263	*bztD*	Amino Acid Metabolism	Amino Acid Transport
RCAP_rcc00339	-0.07	0.5842	1098	1045	0.02%	0.01%	950	1229	989	1101	*sixA*	Signal Transduction	Kinase/Phosphorelay
RCAP_rcc00340	-0.39	0.0025	233	176	0.00%	0.00%	205	253	155	197	*hypothetical protein*	Unknown	Unknown
RCAP_rcc00341	0.01	0.9644	1469	1478	0.02%	0.02%	1236	1707	1348	1608	*argB*	Amino Acid Metabolism	Arginine and proline metabolism
RCAP_rcc00342	1.93	0	809	3209	0.01%	0.04%	864	1103	2766	3651	*short-chain dehydrogenase/reductase family oxidoreductase*	Unknown	Unknown
RCAP_rcc00343	0.73	0	753	1249	0.01%	0.02%	741	849	1157	1342	*engB*	Unknown	Unknown
RCAP_rcc00344	-0.11	0.3747	566	523	0.01%	0.01%	504	620	478	568	*MOSC domain-containing protein*	Unknown	Unknown
RCAP_rcc00345	-0.29	0.2114	6126	4979	0.09%	0.07%	4716	7525	4264	5694	*oxaA*	Trafficking and Secretion	Trafficing
RCAP_rcc00346	0.17	0.0464	600	674	0.01%	0.01%	555	645	635	713	*diguanylate cyclase/phosphodiesterase*	Signal Transduction	Kinase/Phosphorelay
RCAP_rcc00347	-0.61	0	790	515	0.01%	0.01%	703	850	443	587	*PP-loop family ATPase*	Cell Division	Chromosome Partitioning
RCAP_rcc00348	-0.23	0.4505	120	101	0.00%	0.00%	82	152	80	122	*hypothetical protein*	Unknown	Unknown
RCAP_rcc00349	-0.2	0.66	376	317	0.01%	0.00%	146	584	217	416	*rnpA*	Translation, ribosomal structure and biogenesis	Unknown
RCAP_rcc00350	0.04	0.9209	681	704	0.01%	0.01%	331	1020	578	830	*rpmH*	Translation, ribosomal structure and biogenesis	Unknown
RCAP_rcc00351	1.03	0	1051	2155	0.02%	0.03%	1025	1204	1961	2349	*sensor histidine kinase*	Signal Transduction	Kinase/Phosphorelay
RCAP_rcc00352	0.52	0.0508	70	102	0.00%	0.00%	54	89	73	131	*asnA*	Energy Metabolism	Nitrogen metabolism
RCAP_rcc00353	1.63	0	438	1496	0.01%	0.02%	349	713	1171	1822	*hypothetical protein*	Unknown	Unknown
RCAP_rcc00354	0.42	0	829	1113	0.01%	0.02%	765	924	1048	1177	*pepT*	Amino Acid Metabolism	Unknown
RCAP_rcc00355	0.22	0.1558	2368	2770	0.03%	0.04%	2144	2654	2424	3116	*S1 RNA binding domain-containing protein*	Transcription	Unknown
RCAP_rcc00356	1.34	0	704	1794	0.01%	0.02%	702	804	1615	1973	*cyclic nucleotide-binding domain-/cystathionine beta-synthase domain-*	Signal Transduction	Kinase/Phosphorelay
RCAP_rcc00357	0.12	0.2416	1253	1367	0.02%	0.02%	1159	1392	1268	1465	*macB*	Defense Mechanisms	Unknown
RCAP_rcc00358	1.21	0	1138	2715	0.02%	0.04%	1079	1437	2178	3251	*macA*	Defense Mechanisms	Unknown
RCAP_rcc00359	-0.54	0.0108	74	49	0.00%	0.00%	55	88	40	59	*hypothetical protein*	Unknown	Unknown
RCAP_rcc00360	-0.23	0.026	631	538	0.01%	0.01%	571	680	494	582	*mipZ*	Cell Division	Chromosome Partitioning
RCAP_rcc00361	-0.3	0.1156	2802	2261	0.04%	0.03%	2461	3143	1832	2689	*rpmE*	Translation, ribosomal structure and biogenesis	Unknown
RCAP_rcc00362	-0.26	0.1822	9647	8030	0.14%	0.11%	8368	10966	6632	9428	*rplS*	Translation, ribosomal structure and biogenesis	Unknown
RCAP_rcc00363	0.23	0.2485	336	397	0.00%	0.01%	266	406	350	444	*hypothetical protein*	Unknown	Unknown
RCAP_rcc00364	-0.1	0.6348	123	114	0.00%	0.00%	103	140	95	134	*trmD*	Translation, ribosomal structure and biogenesis	Unknown
RCAP_rcc00365	-0.11	0.6232	238	219	0.00%	0.00%	199	274	172	267	*hypothetical protein*	Unknown	Unknown
RCAP_rcc00366	0.13	0.5175	396	433	0.01%	0.01%	352	449	348	519	*rimM*	Translation, ribosomal structure and biogenesis	Unknown
RCAP_rcc00367	-0.2	0.2991	4298	3713	0.06%	0.05%	3651	4947	3035	4391	*rpsP*	Translation, ribosomal structure and biogenesis	Unknown
RCAP_rcc00368	-0.7	0.0109	895	529	0.01%	0.01%	691	1079	381	678	*aroH*	Amino Acid Metabolism	Phenylalanine, tyrosine and tryptophan biosynthesis
RCAP_rcc00369	-0.46	0.0408	854	611	0.01%	0.01%	698	992	488	734	*GNAT family acetyltransferase*	Cell Division	Chromosome Partitioning
RCAP_rcc00370	-0.01	0.9567	1672	1658	0.02%	0.02%	1436	1928	1457	1860	*ffh*	Trafficking and Secretion	Trafficing
RCAP_rcc00371	1.21	0	318	742	0.00%	0.01%	297	380	681	803	*LysR family transcriptional regulator*	Signal Transduction	Transcription Regulator
RCAP_rcc00372	-0.06	0.7852	1326	1267	0.02%	0.02%	1115	1541	1063	1472	*hypothetical protein*	Unknown	Unknown
RCAP_rcc00373	0.34	0.0061	3169	4025	0.05%	0.05%	2872	3680	3748	4302	*mdoH*	Cell Envelope Biosynthesis	Cell Wall Biosynthesis
RCAP_rcc00374	1.57	0	418	1292	0.01%	0.02%	447	609	1103	1481	*hypothetical protein*	Unknown	Unknown
RCAP_rcc00375	0.05	0.8691	5998	6209	0.09%	0.08%	4684	7457	5365	7052	*mdoG1*	Metal and Ion Transport	Unknown
RCAP_rcc00376	0.04	0.9017	2284	2344	0.03%	0.03%	1704	2904	2013	2676	*divalent ion symporter*	Predicted Function	Replication
RCAP_rcc00377	-0.1	0.7389	746	692	0.01%	0.01%	482	967	519	864	*hemL*	Metabolism of Cofactors, Coenzymes and Vitamins	Heme Biosynthesis
RCAP_rcc00378	-0.53	0.0206	2767	1874	0.04%	0.03%	1690	3680	1565	2183	*glnA1*	Carbohydrate Metabolism	Glyoxylate and dicarboxylate metabolism
RCAP_rcc00379	-0.2	0.2625	1359	1176	0.02%	0.02%	1141	1580	1021	1331	*dapA*	Amino Acid Metabolism	Unknown
RCAP_rcc00380	-0.15	0.494	572	514	0.01%	0.01%	455	676	440	588	*transglycosylase, Slt family*	Glycan Biosynthesis and Metabolism	Glycosaminoglycan degradation
RCAP_rcc00381	-0.89	0	671	362	0.01%	0.00%	602	705	341	384	*hypothetical protein*	Unknown	Unknown
RCAP_rcc00382	-0.56	0	373	251	0.01%	0.00%	326	408	229	273	*hypothetical protein*	Unknown	Unknown
RCAP_rcc00383	0.83	0	241	431	0.00%	0.01%	226	266	381	482	*FAD dependent oxidoreductase*	Energy Metabolism	Unknown

RCAP_rcc00384	0.07	0.6424	3298	3459	0.05%	0.05%	3028	3733	3052	3865	*gshA*	Metabolism of Other Amino Acids	Glutathione metabolism
RCAP_rcc00385	1.07	0	87	190	0.00%	0.00%	71	106	138	242	*hypothetical protein*	Unknown	Unknown
RCAP_rcc00386	1.04	0	642	1327	0.01%	0.02%	595	755	1256	1397	*hypothetical protein*	Unknown	Unknown
RCAP_rcc00387	0.65	0	534	844	0.01%	0.01%	501	599	771	917	*ubiA*	Photosynthesis	Biosynthesis of Ubiquinone
RCAP_rcc00388	-0.35	0.0093	1316	1030	0.02%	0.01%	1090	1492	969	1092	*OmpA/MotB domain-containing protein*	Cell Envelope Biosynthesis	Cell Wall Biosynthesis
RCAP_rcc00389	-0.48	0.0001	337	242	0.00%	0.00%	298	374	219	264	*hypothetical protein*	Unknown	Unknown
RCAP_rcc00390	1.75	0	679	2455	0.01%	0.03%	620	957	1898	3012	*S49 family peptidase*	Post-translational Modification, Assembly and Chaperones	Peptidase
RCAP_rcc00391	1.17	NA	21	68	0.00%	0.00%	7	33	19	117	*hypothetical protein*	Unknown	Unknown
RCAP_rcc00392	1.26	NA	41	119	0.00%	0.00%	26	56	13	225	*hypothetical protein*	Unknown	Unknown
RCAP_rcc00393	0.3	0.0035	651	801	0.01%	0.01%	601	723	742	859	*uvrC*	Replication, Recombination and Repair	Repair
RCAP_rcc00394	-0.09	0.6994	101	94	0.00%	0.00%	78	119	79	110	*kamA*	Amino Acid Metabolism	Lysine degradation
RCAP_rcc00395	0.83	0	1679	3016	0.02%	0.04%	1500	2002	2737	3295	*pgsA*	Lipid Metabolism	Glycerophospholipid metabolism
RCAP_rcc00396	0.29	0	861	1055	0.01%	0.01%	817	909	1010	1100	*hypothetical protein*	Unknown	Unknown
RCAP_rcc00397	-0.14	0.1899	946	855	0.01%	0.01%	839	1038	805	904	*pyrC1*	Nucleotide Metabolism	Pyrimidine metabolism
RCAP_rcc00398	-0.11	0.5224	188	173	0.00%	0.00%	158	213	151	195	*hypothetical protein*	Unknown	Unknown
RCAP_rcc00399	-0.05	0.764	365	352	0.01%	0.00%	309	414	322	382	*hypothetical protein*	Unknown	Unknown
RCAP_rcc00400	-0.06	0.7342	1184	1132	0.02%	0.02%	994	1384	1003	1261	*pyrB*	Amino Acid Metabolism	Alanine, aspartate and glutamate metabolism
RCAP_rcc00401	0.36	0.012	177	228	0.00%	0.00%	153	197	197	258	*udgA*	Replication, Recombination and Repair	Phage Interaction
RCAP_rcc00402	0.16	0.4334	265	295	0.00%	0.00%	218	308	262	329	*hypothetical protein*	Unknown	Unknown
RCAP_rcc00403	1.94	0	414	1787	0.01%	0.02%	337	662	1293	2282	*hypothetical protein*	Unknown	Unknown
RCAP_rcc00404	1.33	0.0001	151	438	0.00%	0.01%	106	215	231	645	*Sua5/YciO/YrdC/YwlC family protein*	Translation, ribosomal structure and biogenesis	Unknown
RCAP_rcc00405	0.47	0.1483	888	1270	0.01%	0.02%	649	1255	952	1587	*acyl-CoA dehydrogenase domain-containing protein*	Lipid Metabolism	Unknown
RCAP_rcc00406	0.58	0.034	185	286	0.00%	0.00%	138	231	211	361	*metallo-beta-lactamase*	Unknown	Unknown
RCAP_rcc00407	0.08	0.6208	703	744	0.01%	0.01%	605	810	671	818	*lipoprotein*	Predicted Function	Unknown
RCAP_rcc00408	-0.95	0	124	64	0.00%	0.00%	106	136	59	69	*branched-chain amino acid transport family protein*	Unknown	Unknown
RCAP_rcc00409	-0.89	0	787	421	0.01%	0.01%	650	880	371	470	*AzlC family protein*	Amino Acid Metabolism	Unknown
RCAP_rcc00410	0.62	0.0001	101	156	0.00%	0.00%	87	115	135	178	*GNAT family acetyltransferase*	Cell Division	Chromosome Partitioning
RCAP_rcc00411	0.3	0.437	123	157	0.00%	0.00%	69	173	91	222	*S58 family peptidase*	Post-translational Modification, Assembly and Chaperones	Peptidase
RCAP_rcc00412	0.09	0.5409	639	683	0.01%	0.01%	543	731	638	728	*arylformamidase*	Lipid Metabolism	Unknown
RCAP_rcc00413	-0.59	0	6833	4519	0.09%	0.06%	5951	7479	3988	5050	*gabD*	Carbohydrate Metabolism	Butanoate metabolism
RCAP_rcc00414	-0.85	0	438	242	0.01%	0.00%	387	469	219	266	*hypothetical protein*	Unknown	Unknown
RCAP_rcc00415	1.11	0	1556	3398	0.02%	0.05%	1486	1791	2999	3797	*short-chain dehydrogenase/reductase family oxidoreductase*	Unknown	Unknown
RCAP_rcc00416	0.17	0.4954	298	337	0.00%	0.00%	183	405	294	380	*LysR family transcriptional regulator*	Signal Transduction	Transcription Regulator
RCAP_rcc00417	-2.96	NA	1689	99	0.02%	0.00%	-420	3632	86	113	*hypothetical protein*	Unknown	Unknown
RCAP_rcc00418	0.45	0.0501	237	328	0.00%	0.00%	147	322	277	379	*TetR family transcriptional regulator*	Signal Transduction	Transcription Regulator
RCAP_rcc00419	-0.74	0	375	223	0.01%	0.00%	296	440	202	245	*NADH:flavin oxidoreductase/NADH oxidase*	Energy Metabolism	Unknown
RCAP_rcc00420	1.81	0	112	406	0.00%	0.01%	116	157	356	456	*pykA1*	Carbohydrate Metabolism	Glycolysis / Gluconeogenesis
RCAP_rcc00421	-0.16	0.4408	6351	5672	0.09%	0.08%	5399	7411	4849	6495	*lysC*	Amino Acid Metabolism	Lysine biosynthesis
RCAP_rcc00422	-0.13	0.2941	3837	3511	0.05%	0.05%	3389	4243	3281	3741	*ptsP*	Signal Transduction	Kinase/Phosphorelay
RCAP_rcc00423	1.38	0	263	730	0.00%	0.01%	205	356	608	852	*hypothetical protein*	Unknown	Unknown
RCAP_rcc00424	1.26	0	630	1573	0.01%	0.02%	513	822	1333	1814	*hypothetical protein*	Unknown	Unknown
RCAP_rcc00425	1.01	0	279	575	0.00%	0.01%	237	345	481	669	*M48 family peptidase*	Post-translational Modification, Assembly and Chaperones	Peptidase
RCAP_rcc00426	0.29	0.0615	1045	1287	0.01%	0.02%	893	1208	1138	1435	*hypothetical protein*	Unknown	Unknown
RCAP_rcc00427	0.26	0.0094	771	922	0.01%	0.01%	721	841	843	1000	*panC*	Metabolism of Cofactors, Coenzymes and Vitamins	Pantothenate and CoA biosynthesis
RCAP_rcc00428	0.55	0	496	728	0.01%	0.01%	463	554	665	792	*panB*	Metabolism of Cofactors, Coenzymes and Vitamins	Pantothenate and CoA biosynthesis
RCAP_rcc00429	-0.07	0.504	227	216	0.00%	0.00%	204	250	203	229	*hypothetical protein*	Unknown	Unknown
RCAP_rcc00430	-0.79	0	1201	687	0.02%	0.01%	955	1374	638	737	*ErfK/YbiS/YcfS/YnhG family protein*	Unknown	Unknown
RCAP_rcc00431	-1.62	0	3957	1171	0.05%	0.02%	1663	5997	1039	1303	*creA*	Amino Acid Metabolism	Arginine and proline metabolism
RCAP_rcc00432	0.3	0.0041	7079	8727	0.10%	0.12%	6563	7945	8078	9376	*mdtB*	Metal and Ion Transport	Unknown
RCAP_rcc00433	0.8	0	1648	2887	0.02%	0.04%	1635	1866	2649	3125	*RND family efflux transporter subunit MFP*	Defense Mechanisms	Unknown
RCAP_rcc00434	0.8	0.0073	14	26	0.00%	0.00%	12	17	16	35	*hypothetical protein*	Unknown	Unknown
RCAP_rcc00435	1.82	0	349	1353	0.01%	0.02%	255	537	1152	1554	*hypothetical protein*	Unknown	Unknown
RCAP_rcc00436	1.48	0	616	1760	0.01%	0.02%	624	752	1503	2016	*cytB*	Energy Metabolism	Aerobic/Anaerobic Respiration
RCAP_rcc00437	0.27	0.2532	193	235	0.00%	0.00%	151	234	197	273	*alpha/beta fold family hydrolase*	Unknown	Unknown
RCAP_rcc00438	-0.22	0.3089	11334	9666	0.16%	0.13%	9201	13430	8027	11305	*glyA*	Carbohydrate Metabolism	Glyoxylate and dicarboxylate metabolism
RCAP_rcc00439	0.77	0	436	751	0.01%	0.01%	395	496	673	828	*ppnk*	Metabolism of Cofactors, Coenzymes and Vitamins	Nicotinate and nicotinamide metabolism
RCAP_rcc00440	-0.03	0.9356	241	236	0.00%	0.00%	178	295	193	280	*zraR*	Signal Transduction	Transcription Regulator
RCAP_rcc00441	0.05	0.6645	717	744	0.01%	0.01%	635	776	684	804	*sensor histidine kinase/response regulator receiver protein*	Signal Transduction	Transcription Regulator
RCAP_rcc00442	-0.17	0.2123	1017	899	0.01%	0.01%	917	1105	778	1021	*prpE*	Energy Metabolism	Reductive carboxylate cycle (CO2 fixation)
RCAP_rcc00443	-0.39	0.0013	1357	1033	0.02%	0.01%	1204	1469	930	1136	*maeB1*	Energy Metabolism	Carbon fixation in photosynthetic organisms
RCAP_rcc00444	0.89	0	127	238	0.00%	0.00%	111	147	197	279	*cdd*	Xenobiotics Biodegradation and Metabolism	Drug metabolism - other enzymes
RCAP_rcc00445	0.3	0.002	1066	1315	0.01%	0.02%	1004	1119	1174	1456	*deoA*	Xenobiotics Biodegradation and Metabolism	Drug metabolism - other enzymes
RCAP_rcc00446	0.02	0.9017	943	954	0.01%	0.01%	868	1006	868	1039	*deoB*	Carbohydrate Metabolism	Pentose phosphate pathway
RCAP_rcc00447	0.01	0.9602	3077	3093	0.04%	0.04%	2785	3416	2816	3371	*upp*	Nucleotide Metabolism	Pyrimidine metabolism
RCAP_rcc00448	0.22	0.545	865	1023	0.01%	0.01%	562	1141	718	1328	*sporulation domain-containing protein*	Unknown	Unknown
RCAP_rcc00449	0.4	0.1551	430	579	0.01%	0.01%	312	534	427	731	*hypothetical protein*	Unknown	Unknown
RCAP_rcc00450	-0.36	0.0033	406	316	0.01%	0.00%	356	442	287	346	*hypothetical protein*	Unknown	Unknown
RCAP_rcc00451	-0.66	0	187	117	0.00%	0.00%	166	198	107	127	*hypothetical protein*	Unknown	Unknown
RCAP_rcc00452	-0.58	0	614	412	0.01%	0.01%	562	644	376	448	*amino acid ABC transporter permease*	Amino Acid Metabolism	Amino Acid Transport
RCAP_rcc00453	-0.01	0.9403	1307	1297	0.02%	0.02%	1163	1451	1157	1437	*amino acid ABC transporter periplasmic amino acid-binding protein*	Unknown	Unknown
RCAP_rcc00454	-0.49	0	513	364	0.01%	0.00%	455	549	331	396	*moeB1*	Metabolism of Cofactors, Coenzymes and Vitamins	Unknown
RCAP_rcc00455	-0.59	0.0073	335	217	0.00%	0.00%	251	402	172	263	*dut*	Nucleotide Metabolism	Pyrimidine metabolism
RCAP_rcc00456	-0.71	0.0004	1375	822	0.02%	0.01%	1023	1652	701	943	*coaBC*	Metabolism of Cofactors, Coenzymes and Vitamins	Pantothenate and CoA biosynthesis
RCAP_rcc00457	-0.84	0.0139	902	465	0.01%	0.01%	587	1159	234	695	*hypothetical protein*	Unknown	Unknown
RCAP_rcc00458	-0.21	0.3228	5561	4765	0.08%	0.06%	4839	6481	3685	5844	*rpoH1*	Replication, Recombination and Repair	Replication
RCAP_rcc00459	-0.54	0.0136	618	417	0.01%	0.01%	435	772	344	491	*glutathione S-transferase*	Sulfur Metabolism	Glutathione metabolism
RCAP_rcc00460	-0.6	0	403	264	0.01%	0.00%	359	426	225	303	*comM*	Metal, Ion, Cofactor Transport	Magnesium Transport

RCAP_rcc00461	-0.07	0.8196	355	337	0.00%	0.00%	263	441	278	396	*est*	Lipid Metabolism	Unknown
RCAP_rcc00462	-0.44	0.0001	2482	1819	0.03%	0.02%	2123	2775	1690	1947	*gshB*	Sulfur Metabolism	Glutathione metabolism
RCAP_rcc00463	-0.22	0.1296	1713	1466	0.02%	0.02%	1449	1941	1333	1598	*hypothetical protein*	Unknown	Unknown
RCAP_rcc00464	0.41	0.0489	1393	1879	0.02%	0.03%	1106	1712	1625	2132	*tetrapyrrole methylase*	Unknown	Unknown
RCAP_rcc00465	0.32	0.2638	957	1212	0.01%	0.02%	681	1260	1022	1401	*extracellular ligand-binding receptor family protein*	Unknown	Unknown
RCAP_rcc00466	-0.57	0	3035	2035	0.04%	0.03%	2723	3239	1862	2208	*glnD*	Post-translational Modification, Assembly and Chaperones	Unknown
RCAP_rcc00467	-0.5	0	1314	925	0.02%	0.01%	1209	1377	868	982	*mviN*	Unknown	Unknown
RCAP_rcc00468	0.19	0.0027	1067	1215	0.02%	0.02%	1009	1135	1162	1268	*rhomboid family protein*	Unknown	Unknown
RCAP_rcc00469	-0.24	0.2886	2817	2367	0.04%	0.03%	2231	3357	1951	2783	*trpS*	Translation, ribosomal structure and biogenesis	Aminoacyl-tRNA biosynthesis
RCAP_rcc00470	-0.23	0.0497	430	366	0.01%	0.00%	389	451	323	409	*MATE efflux family protein*	Defense Mechanisms	Unknown
RCAP_rcc00471	-0.01	0.964	402	401	0.01%	0.01%	356	441	357	444	*thioesterase superfamily protein*	Secondary metabolites biosynthesis, transport, and catabolism	Unknown
RCAP_rcc00472	0.38	0.3984	126	173	0.00%	0.00%	61	185	108	238	*thioesterase superfamily protein*	Secondary metabolites biosynthesis, transport, and catabolism	Unknown
RCAP_rcc00473	0.51	0.001	359	516	0.01%	0.01%	309	423	469	564	*MerR family transcriptional regulator*	Signal Transduction	Transcription Regulator
RCAP_rcc00474	-0.42	0.145	1063	777	0.02%	0.01%	862	1341	537	1016	*MerR family transcriptional regulator*	Signal Transduction	Transcription Regulator
RCAP_rcc00475	-0.54	0.0002	2337	1592	0.03%	0.02%	2048	2645	1374	1811	*acyl-CoA dehydrogenase domain-containing protein*	Lipid Metabolism	Unknown
RCAP_rcc00476	0.34	0.0008	2392	3029	0.03%	0.04%	2219	2680	2781	3276	*hypothetical protein*	Unknown	Unknown
RCAP_rcc00477	-0.49	0	278	199	0.00%	0.00%	245	305	184	213	*ribonuclease T2 family protein*	Translation, ribosomal structure and biogenesis	Unknown
RCAP_rcc00478	-0.72	0	795	479	0.01%	0.01%	657	892	446	511	*alcohol dehydrogenase*	Energy Metabolism	Unknown
RCAP_rcc00479	-0.47	0	1069	772	0.01%	0.01%	957	1132	736	807	*COQ9 family ubiquinone biosynthesis protein*	Energy Metabolism	Biosynthesis of Ubiquinone
RCAP_rcc00480	-0.28	0.3163	4989	4038	0.07%	0.05%	3383	6479	3282	4794	*rpsU*	Translation, ribosomal structure and biogenesis	Unknown
RCAP_rcc00481	-0.02	0.9159	565	556	0.01%	0.01%	483	643	482	631	*mcpI*	Motility	Chemotaxis
RCAP_rcc00482	0.11	0.5956	89	96	0.00%	0.00%	77	103	80	112	*hypothetical protein*	Unknown	Unknown
RCAP_rcc00483	0.47	0.039	145	204	0.00%	0.00%	115	193	181	227	*AsnC/Lrp family transcriptional regulator*	Signal Transduction	Transcription Regulator
RCAP_rcc00484	-2.11	0	23022	4721	0.31%	0.06%	13080	31029	3773	5669	*ald*	Metabolism of Other Amino Acids	Taurine and hypotaurine metabolism
RCAP_rcc00485	-2.07	0	8424	1886	0.11%	0.03%	6457	9698	1541	2231	*gst*	Sulfur Metabolism	Glutathione metabolism
RCAP_rcc00486	-0.32	0	1032	826	0.01%	0.01%	959	1099	779	872	*hypothetical protein*	Unknown	Unknown
RCAP_rcc00487	-0.53	0	3166	2179	0.04%	0.03%	2921	3339	1960	2398	*suhB*	Signal Transduction	Transcription Regulator
RCAP_rcc00488	-0.75	0	2880	1692	0.04%	0.02%	2504	3156	1448	1935	*fsr*	Metal and Ion Transport	Unknown
RCAP_rcc00489	-0.16	0.4333	541	482	0.01%	0.01%	440	621	397	568	*LysR family transcriptional regulator*	Signal Transduction	Transcription Regulator
RCAP_rcc00490	-0.53	NA	6086	3875	0.09%	0.05%	645	11466	2416	5335	*metF*	Energy Metabolism	Reductive carboxylate cycle (CO2 fixation)
RCAP_rcc00491	-0.11	0.7314	142	130	0.00%	0.00%	89	193	93	167	*thioesterase superfamily protein*	Unknown	Unknown
RCAP_rcc00492	0.74	0.0001	592	1004	0.01%	0.01%	568	685	765	1243	*hypothetical protein*	Unknown	Unknown
RCAP_rcc00493	-1.71	0	9398	2715	0.13%	0.04%	8419	10017	1986	3444	*ldc*	Metabolism of Other Amino Acids	Glutathione metabolism
RCAP_rcc00494	-0.02	0.9063	110	110	0.00%	0.00%	97	120	100	120	*AsnC/Lrp family transcriptional regulator*	Signal Transduction	Transcription Regulator
RCAP_rcc00495	-0.41	0.0028	360	269	0.01%	0.00%	302	413	246	293	*glyoxalase/bleomycin resistance protein/dioxygenase*	Unknown	Unknown
RCAP_rcc00496	-0.14	0.5175	8346	7533	0.12%	0.10%	6629	10070	6509	8556	*valS*	Translation, ribosomal structure and biogenesis	Aminoacyl-tRNA biosynthesis
RCAP_rcc00497	-0.25	0.0117	635	532	0.01%	0.01%	565	689	501	564	*UbiA prenyltransferase*	Photosynthesis	Biosynthesis of Ubiquinone
RCAP_rcc00498	0.93	0	282	542	0.00%	0.01%	263	339	494	589	*transglycosylase, Slt family*	Glycan Biosynthesis and Metabolism	Glycosaminoglycan degradation
RCAP_rcc00499	-0.36	0.162	22316	17142	0.31%	0.23%	16605	27901	14359	19925	*hypothetical protein*	Unknown	Unknown
RCAP_rcc00500	-0.98	0	3622	1818	0.05%	0.02%	3056	4036	1695	1941	*hypothetical protein*	Unknown	Unknown
RCAP_rcc00501	-0.97	0	3850	1955	0.05%	0.03%	3211	4307	1888	2022	*cpaB*	Trafficking and Secretion	Trafficing
RCAP_rcc00502	-0.22	0.0272	6810	5846	0.10%	0.08%	6209	7371	5426	6267	*type II and III secretion system protein*	Trafficking and Secretion	Secretion
RCAP_rcc00503	-0.39	0.0004	1926	1465	0.03%	0.02%	1714	2106	1349	1581	*OmpA/MotB domain-containing protein*	Cell Envelope Biosynthesis	Cell Wall Biosynthesis
RCAP_rcc00504	-0.93	0	6722	3467	0.09%	0.05%	5512	7720	2951	3982	*PP-loop family ATPase*	Trafficking and Secretion	Trafficing
RCAP_rcc00505	-0.96	0	4069	2053	0.06%	0.03%	3395	4543	1778	2329	*type II secretion system protein E*	Trafficking and Secretion	Secretion
RCAP_rcc00506	-0.92	0	2360	1235	0.03%	0.02%	2025	2609	1102	1368	*type II secretion system protein*	Trafficking and Secretion	Secretion
RCAP_rcc00507	-0.85	0	1758	971	0.02%	0.01%	1553	1901	885	1057	*type II secretion system protein*	Trafficking and Secretion	Secretion
RCAP_rcc00508	-0.36	0.0002	543	422	0.01%	0.01%	483	588	397	446	*hypothetical protein*	Unknown	Unknown
RCAP_rcc00509	-0.08	0.5612	1497	1414	0.02%	0.02%	1314	1676	1294	1534	*hypothetical protein*	Unknown	Unknown
RCAP_rcc00510	-0.08	0.4708	433	410	0.01%	0.01%	395	463	379	441	*A24 family peptidase*	Post-translational Modification, Assembly and Chaperones	Peptidase
RCAP_rcc00511	-0.02	0.9496	345	339	0.00%	0.00%	236	446	249	430	*ispE*	Lipid	Terpenoid backbone biosynthesis
RCAP_rcc00512	-0.16	0.0549	2156	1932	0.03%	0.03%	1992	2294	1805	2059	*hypothetical protein*	Unknown	Unknown
RCAP_rcc00513	-0.3	0.0463	4643	3765	0.07%	0.05%	3958	5314	3391	4139	*etfD*	Energy Metabolism	Aerobic/Anaerobic Respiration
RCAP_rcc00514	-0.07	0.7865	2018	1919	0.03%	0.03%	1519	2512	1632	2206	*greA*	Transcription	Unknown
RCAP_rcc00515	0.29	0.1527	416	513	0.01%	0.01%	320	508	454	571	*hypothetical protein*	Unknown	Unknown
RCAP_rcc00516	2.19	0	1002	4842	0.02%	0.07%	1134	1443	3929	5755	*tpl*	Amino Acid Metabolism	Tryptophan metabolism
RCAP_rcc00517	0.86	0	196	360	0.00%	0.00%	179	223	318	402	*hypothetical protein*	Unknown	Unknown
RCAP_rcc00518	0.11	0.7632	1913	2079	0.03%	0.03%	1399	2727	1559	2600	*fadA*	Lipid Metabolism	Fatty acid metabolism
RCAP_rcc00519	0.24	0.4568	250	301	0.00%	0.00%	168	353	249	353	*cupin domain-containing protein*	Unknown	Unknown
RCAP_rcc00520	0.53	0.001	1122	1633	0.02%	0.02%	977	1353	1452	1813	*fadB*	Lipid Metabolism	Fatty acid metabolism
RCAP_rcc00521	0.4	0	2532	3356	0.04%	0.05%	2463	2766	3134	3577	*dcp*	Amino Acid Metabolism	Unknown
RCAP_rcc00522	-0.01	0.9573	371	367	0.01%	0.00%	309	429	330	405	*fieF*	Metal and Ion Transport	Unknown
RCAP_rcc00523	0.32	0.0638	271	339	0.00%	0.00%	228	316	302	376	*hypothetical protein*	Unknown	Unknown
RCAP_rcc00524	0.12	0.238	885	961	0.01%	0.01%	806	976	928	993	*gyaR1*	Carbohydrate Metabolism	Glyoxylate and dicarboxylate metabolism
RCAP_rcc00525	-0.98	0	4235	2096	0.06%	0.03%	3091	5179	1940	2251	*hypothetical protein*	Unknown	Unknown
RCAP_rcc00526	-0.35	0.1945	14037	10807	0.20%	0.15%	11539	16582	8449	13166	*dimethylglycine dehydrogenase*	Amino Acid Metabolism	Unknown
RCAP_rcc00527	0	0.9941	492	493	0.01%	0.01%	405	572	419	566	*family 25 glycosyl hydrolase*	Cell Envelope Biosynthesis	Cell Wall Biosynthesis
RCAP_rcc00528	0.54	0	293	427	0.00%	0.01%	274	317	380	474	*carboxymuconolactone decarboxylase*	Xenobiotics Biodegradation and Metabolism	Benzoate degradation via hydroxylation
RCAP_rcc00529	0.65	0.0045	464	746	0.01%	0.01%	358	578	598	894	*FAD dependent oxidoreductase*	Energy Metabolism	Unknown
RCAP_rcc00530	1.49	0	1107	3145	0.02%	0.04%	1120	1352	2895	3394	*hypothetical protein*	Unknown	Unknown
RCAP_rcc00531	-0.28	0.2268	7367	6018	0.10%	0.08%	5917	8771	5011	7025	*carB*	Amino Acid Metabolism	Alanine, aspartate and glutamate metabolism
RCAP_rcc00532	-0.4	0.0445	10487	7894	0.15%	0.11%	8497	12381	6764	9023	*aspS*	Translation, ribosomal structure and biogenesis	Aminoacyl-tRNA biosynthesis
RCAP_rcc00533	-0.03	0.82	1028	1006	0.01%	0.01%	949	1095	894	1118	*hypothetical protein*	Unknown	Unknown
RCAP_rcc00534	0.04	0.9123	351	363	0.00%	0.00%	236	461	292	433	*hypothetical protein*	Unknown	Unknown
RCAP_rcc00535	-0.36	0.1649	147	112	0.00%	0.00%	108	185	93	132	*hypothetical protein*	Unknown	Unknown
RCAP_rcc00536	-0.47	0	1010	729	0.01%	0.01%	882	1103	658	799	*GNAT family acetyltransferase*	Cell Division	Chromosome Partitioning
RCAP_rcc00537	-0.42	0.1776	1433	1044	0.02%	0.01%	946	1849	852	1236	*response regulator receiver protein*	Signal Transduction	Transcription Regulator

RCAP_rcc00538	0.01	0.9664	1023	1030	0.01%	0.01%	860	1210	921	1138	*methylmalonyl-CoA epimerase*	Carbohydrate Metabolism	Glyoxylate and dicarboxylate metabolism
RCAP_rcc00539	1.44	0	1021	2817	0.02%	0.04%	1056	1245	2513	3121	*hypothetical protein*	Unknown	Unknown
RCAP_rcc00540	2.02	0	277	1438	0.00%	0.02%	195	435	912	1964	*nitroreductase*	Energy Metabolism	Unknown
RCAP_rcc00541	0.52	0.0002	77	111	0.00%	0.00%	67	90	101	120	*hypothetical protein*	Unknown	Unknown
RCAP_rcc00542	1.39	0	467	1273	0.01%	0.02%	434	615	1039	1507	*hypothetical protein*	Unknown	Unknown
RCAP_rcc00543	1.91	0	2426	9610	0.04%	0.13%	2646	3426	7780	11440	*hypothetical protein*	Unknown	Unknown
RCAP_rcc00544	0.35	0.226	553	718	0.01%	0.01%	390	703	507	929	*aldehyde dehydrogenase*	Carbohydrate Metabolism	Glycolysis / Gluconeogenesis
RCAP_rcc00545	0.42	0.0134	319	428	0.00%	0.01%	270	367	365	492	*deoC*	Carbohydrate Metabolism	Pentose phosphate pathway
RCAP_rcc00546	-0.28	0.189	400	327	0.01%	0.00%	304	487	293	362	*hypothetical protein*	Unknown	Unknown
RCAP_rcc00547	0.34	0.0395	6147	7829	0.09%	0.11%	5495	7246	6656	9003	*glutaredoxin family protein*	Post-translational Modification, Assembly and Chaperones	Unknown
RCAP_rcc00548	0.57	0	2247	3353	0.03%	0.05%	2199	2562	3058	3648	*BolA family protein*	Signal Transduction	Kinase/Phosphorelay
RCAP_rcc00549	-0.15	0.2413	5774	5208	0.08%	0.07%	5190	6385	4789	5626	*purL*	Nucleotide Metabolism	Purine metabolism
RCAP_rcc00550	-0.55	0.0002	1981	1342	0.03%	0.02%	1799	2138	1108	1577	*LysR family transcriptional regulator*	Signal Transduction	Transcription Regulator
RCAP_rcc00551	0.35	0	1243	1588	0.02%	0.02%	1185	1344	1530	1646	*murI*	Metabolism of Other Amino Acids	D-Glutamine and D-glutamate metabolism
RCAP_rcc00552	0.1	0.1737	969	1039	0.01%	0.01%	909	1022	988	1090	*hypothetical protein*	Unknown	Unknown
RCAP_rcc00553	-0.29	0.0276	2324	1891	0.03%	0.03%	2049	2578	1682	2100	*argC*	Amino Acid Metabolism	Arginine and proline metabolism
RCAP_rcc00554	-0.47	0	919	662	0.01%	0.01%	834	976	620	704	*ccmE*	Energy Metabolism	Cytochrome Biogenesis
RCAP_rcc00555	-1.05	0	197	92	0.00%	0.00%	146	238	83	101	*hypothetical protein*	Unknown	Unknown
RCAP_rcc00556	-0.36	0.0582	197	153	0.00%	0.00%	157	230	132	174	*hypothetical protein*	Unknown	Unknown
RCAP_rcc00557	-0.39	0.0926	3542	2677	0.05%	0.04%	2766	4252	2254	3099	*metG*	Translation, ribosomal structure and biogenesis	Aminoacyl-tRNA biosynthesis
RCAP_rcc00558	0.62	0	1569	2422	0.02%	0.03%	1380	1821	2298	2546	*diguanylate cyclase/phosphodiesterase*	Signal Transduction	Kinase/Phosphorelay
RCAP_rcc00559	-1.19	NA	1443	589	0.02%	0.01%	742	2065	546	632	*pmtA*	Lipid Metabolism	Glycerophospholipid metabolism
RCAP_rcc00560	-0.77	0.0065	258	145	0.00%	0.00%	152	350	118	172	*mopB*	Signal Transduction	Transcription Regulator
RCAP_rcc00561	3.6	NA	12	292	0.00%	0.00%	28	42	183	401	*mopA*	Signal Transduction	Transcription Regulator
RCAP_rcc00562	2.96	NA	171	2981	0.01%	0.04%	278	531	1913	4048	*modA1*	Metal and Ion Transport	Unknown
RCAP_rcc00563	1.08	0	479	1038	0.01%	0.01%	452	577	834	1241	*modB1*	Metal and Ion Transport	Unknown
RCAP_rcc00564	0.71	0.0005	249	414	0.00%	0.01%	214	293	327	502	*modC1*	Metal and Ion Transport	Unknown
RCAP_rcc00565	0.56	0.1572	145	230	0.00%	0.00%	81	207	145	316	*modD*	Metabolism of Cofactors, Coenzymes and Vitamins	Unknown
RCAP_rcc00566	0.21	0.1235	102	118	0.00%	0.00%	89	117	110	126	*nifB1*	Energy Metabolism	Nitrogen metabolism
RCAP_rcc00567	1.95	0	229	942	0.00%	0.01%	256	338	766	1118	*nifA1*	Signal Transduction	Transcription Regulator
RCAP_rcc00568	1.24	0	47	112	0.00%	0.00%	43	55	99	125	*rpoN*	Replication, Recombination and Repair	Replication
RCAP_rcc00569	1.3	0.0001	4	10	0.00%	0.00%	2	5	7	13	*nifU1*	Energy Metabolism	Nitrogen metabolism
RCAP_rcc00570	-0.18	0.4272	180	159	0.00%	0.00%	140	222	137	180	*nifK*	Energy Metabolism	Nitrogen metabolism
RCAP_rcc00571	-0.05	0.8377	224	216	0.00%	0.00%	178	270	183	250	*nifD*	Energy Metabolism	Nitrogen metabolism
RCAP_rcc00572	0.99	0	46	93	0.00%	0.00%	39	59	81	106	*nifH1*	Energy Metabolism	Nitrogen metabolism
RCAP_rcc00573	0.22	0.332	17	20	0.00%	0.00%	13	21	17	23	*fdxD*	Energy Metabolism	Aerobic/Anaerobic Respiration
RCAP_rcc00574	0.7	0.0753	21	38	0.00%	0.00%	11	31	20	56	*Crp/Fnr family transcriptional regulator*	Signal Transduction	Transcription Regulator
RCAP_rcc00575	3.15	0	35	336	0.00%	0.00%	39	53	284	389	*hypothetical protein*	Unknown	Unknown
RCAP_rcc00576	3.36	0	15	175	0.00%	0.00%	15	20	131	220	*cbbO*	Energy Metabolism	Carbon fixation in photosynthetic organisms
RCAP_rcc00577	3.62	0	20	253	0.00%	0.00%	23	28	220	286	*cbbQ*	Energy Metabolism	Carbon fixation in photosynthetic organisms
RCAP_rcc00578	4.34	0	27	614	0.00%	0.01%	35	48	504	725	*cbbS*	Energy Metabolism	Carbon fixation in photosynthetic organisms
RCAP_rcc00579	4.54	0	66	1697	0.00%	0.02%	103	127	1439	1954	*cbbL*	Energy Metabolism	Carbon fixation in photosynthetic organisms
RCAP_rcc00580	0.99	0	56	114	0.00%	0.00%	46	73	95	133	*cbbR1*	Signal Transduction	Transcription Regulator
RCAP_rcc00581	0.14	0.4771	179	197	0.00%	0.00%	145	210	173	220	*hypothetical protein*	Unknown	Unknown
RCAP_rcc00582	0.99	0	48	97	0.00%	0.00%	42	58	83	111	*hypothetical protein*	Unknown	Unknown
RCAP_rcc00583	1.38	0.0001	86	261	0.00%	0.00%	48	145	175	347	*TetR family transcriptional regulator*	Signal Transduction	Transcription Regulator
RCAP_rcc00584	3.63	0	62	1231	0.00%	0.02%	126	177	789	1674	*anfA*	Signal Transduction	Transcription Regulator
RCAP_rcc00585	0.04	0.8954	30	31	0.00%	0.00%	21	39	27	35	*anfH*	Energy Metabolism	Nitrogen metabolism
RCAP_rcc00586	-0.18	0.5557	90	78	0.00%	0.00%	58	117	66	91	*anfD*	Energy Metabolism	Nitrogen metabolism
RCAP_rcc00587	0.05	0.8976	9	9	0.00%	0.00%	6	12	7	11	*anfG*	Energy Metabolism	Nitrogen metabolism
RCAP_rcc00588	-0.15	0.5746	65	59	0.00%	0.00%	49	82	49	69	*anfK*	Energy Metabolism	Nitrogen metabolism
RCAP_rcc00589	0.22	0.4924	6	7	0.00%	0.00%	5	8	5	8	*anfO*	Energy Metabolism	Nitrogen metabolism
RCAP_rcc00590	0.33	0.192	6	8	0.00%	0.00%	5	7	6	9	*hypothetical protein*	Unknown	Unknown
RCAP_rcc00591	-0.37	0.0032	79	61	0.00%	0.00%	71	87	56	66	*flavin-nucleotide-binding protein*	Unknown	Unknown
RCAP_rcc00592	-0.25	0.1219	155	130	0.00%	0.00%	132	174	114	145	*hyi*	Carbohydrate Metabolism	Glyoxylate and dicarboxylate metabolism
RCAP_rcc00593	0.01	0.9383	206	208	0.00%	0.00%	185	226	192	224	*hypothetical protein*	Unknown	Unknown
RCAP_rcc00594	0.81	0	631	1126	0.01%	0.02%	558	778	1010	1242	*MoxR family ATPase*	Unknown	Unknown
RCAP_rcc00595	0.13	0.8026	98	110	0.00%	0.00%	45	143	52	167	*hypothetical protein*	Unknown	Unknown
RCAP_rcc00596	0.01	0.9642	859	867	0.01%	0.01%	642	1055	706	1027	*hypothetical protein*	Unknown	Unknown
RCAP_rcc00597	-0.19	0.3183	1095	959	0.02%	0.01%	900	1254	815	1103	*hypothetical protein*	Unknown	Unknown
RCAP_rcc00598	-0.51	0.2171	115	75	0.00%	0.00%	47	174	47	102	*M20 family peptidase*	Post-translational Modification, Assembly and Chaperones	Peptidase
RCAP_rcc00599	-0.49	0.0013	213	151	0.00%	0.00%	175	246	136	166	*hypothetical protein*	Unknown	Unknown
RCAP_rcc00600	-0.58	0.0002	3760	2486	0.05%	0.03%	3162	4203	2136	2835	*nrdJ1*	Metabolism of Other Amino Acids	Glutathione metabolism
RCAP_rcc00601	-0.56	0.1019	9151	5912	0.13%	0.08%	4379	13593	5311	6513	*mntH*	Metal and Ion Transport	Unknown
RCAP_rcc00602	0.79	0	693	1215	0.01%	0.02%	584	857	1119	1310	*mntR*	Signal Transduction	Transcription Regulator
RCAP_rcc00603	-0.11	0.6026	492	453	0.01%	0.01%	411	568	351	556	*alpha/beta fold family hydrolase*	Lipid Metabolism	Glycerophospholipid metabolism
RCAP_rcc00604	1.53	0	6937	21082	0.11%	0.29%	6497	9674	17010	25155	*TerC family integral membrane protein*	Metal and Ion Transport	Unknown
RCAP_rcc00605	0.47	0.0006	3585	5003	0.05%	0.07%	3258	4052	4293	5713	*kup*	Metal and Ion Transport	Unknown
RCAP_rcc00606	-0.2	0.3974	998	861	0.01%	0.01%	760	1242	714	1009	*lipoprotein*	Predicted Function	Unknown
RCAP_rcc00607	-1.36	0	28105	9690	0.38%	0.13%	20102	33879	6326	13054	*cspA1*	Unknown	Unknown
RCAP_rcc00608	-1.27	0.0001	2467	914	0.03%	0.01%	1600	3133	607	1222	*hypothetical protein*	Unknown	Unknown
RCAP_rcc00609	-1.25	0.0027	345	118	0.00%	0.00%	158	500	47	188	*hypothetical protein*	Unknown	Unknown
RCAP_rcc00610	-1.16	0.0088	334	117	0.00%	0.00%	213	425	29	205	*hypothetical protein*	Unknown	Unknown
RCAP_rcc00611	0.4	0.0585	239	318	0.00%	0.00%	194	282	253	384	*nikR*	Signal Transduction	Transcription Regulator
RCAP_rcc00612	0.68	0.0016	100	164	0.00%	0.00%	83	122	135	192	*hypothetical protein*	Unknown	Unknown
RCAP_rcc00613	0.89	0	311	582	0.00%	0.01%	293	360	485	680	*TetR family transcriptional regulator*	Signal Transduction	Transcription Regulator
RCAP_rcc00614	0.72	0.0062	156	266	0.00%	0.00%	112	204	192	340	*RND efflux system outer membrane lipoprotein*	Cell Envelope Biosynthesis	Cell Wall Biosynthesis

RCAP_rcc00615	-0.13	0.5315	437	399	0.01%	0.01%	335	540	360	438	*acrA*	Defense Mechanisms	Unknown
RCAP_rcc00616	-0.33	0.0127	3522	2783	0.05%	0.04%	3163	3884	2476	3089	*acrB*	Metal and Ion Transport	Unknown
RCAP_rcc00617	0.88	0	292	551	0.00%	0.01%	252	354	442	660	*hemolysin D*	Trafficking and Secretion	Secretion
RCAP_rcc00618	0.34	0.0015	646	817	0.01%	0.01%	603	694	730	903	*ABC transporter ATP-binding/permease*	Defense Mechanisms	Unknown
RCAP_rcc00619	0.16	0.2225	419	469	0.01%	0.01%	382	468	427	511	*ABC transporter permease*	Defense Mechanisms	Unknown
RCAP_rcc00620	-0.39	0.0132	1045	796	0.01%	0.01%	861	1206	727	864	*response regulator receiver modulated diguanylate cyclase/phosphodies*	Signal Transduction	Transcription Regulator
RCAP_rcc00621	-0.58	0.0243	500	325	0.01%	0.00%	354	619	253	397	*signal transduction histidine kinase*	Signal Transduction	Kinase/Phosphorelay
RCAP_rcc00622	0.57	0.1329	533	846	0.01%	0.01%	305	754	560	1133	*moeA*	Metabolism of Cofactors, Coenzymes and Vitamins	Unknown
RCAP_rcc00623	-0.44	0.0001	152	111	0.00%	0.00%	138	161	99	124	*RpiR family transcriptional regulator*	Signal Transduction	Transcription Regulator
RCAP_rcc00624	-0.72	0	1902	1143	0.03%	0.02%	1681	2075	963	1324	*amino acid ABC transporter periplasmic amino acid-binding protein*	Amino Acid Metabolism	Amino Acid Transport
RCAP_rcc00625	-0.55	0	357	244	0.00%	0.00%	333	371	214	274	*amino acid ABC transporter permease*	Amino Acid Metabolism	Amino Acid Transport
RCAP_rcc00626	-0.56	0	188	126	0.00%	0.00%	160	208	112	141	*amino acid ABC transporter permease*	Amino Acid Metabolism	Amino Acid Transport
RCAP_rcc00627	-0.59	0	374	246	0.01%	0.00%	327	413	216	277	*amino acid ABC transporter ATP-binding protein*	Amino Acid Metabolism	Amino Acid Transport
RCAP_rcc00628	-0.63	0	546	353	0.01%	0.00%	506	573	319	387	*menC*	Secondary metabolites biosynthesis, transport, and catabolism	Unknown
RCAP_rcc00629	-0.36	0.1702	127	98	0.00%	0.00%	94	155	80	115	*GNAT family acetyltransferase*	Cell Division	Chromosome Partitioning
RCAP_rcc00630	-0.12	0.634	631	577	0.01%	0.01%	499	740	420	733	*ice nucleation protein repeat family protein*	Unknown	Unknown
RCAP_rcc00631	1.5	0	69	211	0.00%	0.00%	61	96	149	273	*hypothetical protein*	Unknown	Unknown
RCAP_rcc00632	0.26	0.1406	66	80	0.00%	0.00%	58	75	65	94	*hypothetical protein*	Unknown	Unknown
RCAP_rcc00633	0.03	0.9409	69	70	0.00%	0.00%	46	89	50	90	*ABC transporter permease*	Defense Mechanisms	Unknown
RCAP_rcc00634	0.31	0.5241	18	24	0.00%	0.00%	9	26	14	34	*ABC transporter ATP-binding protein*	Defense Mechanisms	Unknown
RCAP_rcc00635	0.19	0.352	237	271	0.00%	0.00%	189	287	238	304	*von Willebrand factor type A domain-containing protein*	Signal Transduction	Transcription Regulator
RCAP_rcc00636	0.27	0.47	30	37	0.00%	0.00%	18	41	27	47	*hypothetical protein*	Unknown	Unknown
RCAP_rcc00637	0.19	0.2727	313	357	0.00%	0.00%	260	370	327	388	*hypothetical protein*	Unknown	Unknown
RCAP_rcc00638	0.19	0.2695	470	537	0.01%	0.01%	390	552	479	595	*srfB*	Unknown	Unknown
RCAP_rcc00639	0.15	0.5371	59	65	0.00%	0.00%	45	69	53	78	*hypothetical protein*	Unknown	Unknown
RCAP_rcc00640	0.44	0.1803	62	86	0.00%	0.00%	40	82	61	111	*S1/S6 family peptidase*	Post-translational Modification, Assembly and Chaperones	Peptidase
RCAP_rcc00641	0.3	0.078	112	139	0.00%	0.00%	94	130	122	155	*hypothetical protein*	Unknown	Unknown
RCAP_rcc00642	0.42	0.0638	312	421	0.00%	0.01%	241	380	338	505	*peptidoglycan binding domain-containing protein*	Unknown	Unknown
RCAP_rcc00643	-1.73	0	2161	637	0.03%	0.01%	1817	2334	555	719	*diguanylate cyclase/phosphodiesterase*	Signal Transduction	Kinase/Phosphorelay
RCAP_rcc00644	-0.77	0.0003	455	260	0.01%	0.00%	350	544	212	308	*mcpX*	Motility	Chemotaxis
RCAP_rcc00645	0.57	0.0035	411	618	0.01%	0.01%	343	489	503	732	*diguanylate cyclase/phosphodiesterase*	Signal Transduction	Kinase/Phosphorelay
RCAP_rcc00646	0.32	0.0255	182	229	0.00%	0.00%	157	208	204	254	*GNAT family acetyltransferase*	Cell Division	Chromosome Partitioning
RCAP_rcc00647	-0.04	0.8957	105	101	0.00%	0.00%	75	132	86	117	*kdtA2*	Energy Metabolism	Puromycin biosynthesis
RCAP_rcc00648	0.03	0.9319	140	143	0.00%	0.00%	89	184	99	186	*kdtA3*	Energy Metabolism	Puromycin biosynthesis
RCAP_rcc00649	0.25	0.3358	190	227	0.00%	0.00%	146	235	202	253	*dgkA*	Signal Transduction	Transcription Regulator
RCAP_rcc00650	-0.65	0	826	522	0.01%	0.01%	767	872	450	594	*sulfatase*	Unknown	Unknown
RCAP_rcc00651	0.21	0.4333	63	74	0.00%	0.00%	47	77	55	93	*winged helix family two component transcriptional regulator*	Signal Transduction	Transcription Regulator
RCAP_rcc00652	0.06	0.8321	168	175	0.00%	0.00%	127	201	139	211	*sensor histidine kinase*	Signal Transduction	Kinase/Phosphorelay
RCAP_rcc00653	-0.77	0	1016	590	0.01%	0.01%	894	1102	519	662	*ABC transporter*	Unknown	Unknown
RCAP_rcc00654	0.63	0.0228	175	280	0.00%	0.00%	128	221	202	357	*hypothetical protein*	Unknown	Unknown
RCAP_rcc00655	1.41	0	215	590	0.00%	0.01%	217	289	498	683	*hypothetical protein*	Unknown	Unknown
RCAP_rcc00656	0.91	0	992	1892	0.01%	0.03%	878	1202	1714	2071	*hypothetical protein*	Unknown	Unknown
RCAP_rcc00657	0.88	0	587	1110	0.01%	0.02%	474	746	976	1244	*hypothetical protein*	Unknown	Unknown
RCAP_rcc00658	1.16	0	1409	3253	0.02%	0.04%	1170	1909	3000	3506	*hypothetical protein*	Unknown	Unknown
RCAP_rcc00659	1.69	0	17102	56589	0.28%	0.77%	17641	22834	49462	63715	*puhA*	Photosynthesis	Light Harvesting Machinery
RCAP_rcc00660	1.2	0	5004	11599	0.08%	0.16%	4665	6176	10921	12277	*pucC1*	Photosynthesis	Light Harvesting Machinery
RCAP_rcc00661	0.94	0	2172	4256	0.03%	0.06%	1726	2862	4052	4461	*bchM*	Photosynthesis	Biosynthesis of Bacteriochlorophyll
RCAP_rcc00662	0.88	0	4695	8806	0.07%	0.12%	3744	6128	8182	9431	*bchL*	Photosynthesis	Biosynthesis of Bacteriochlorophyll
RCAP_rcc00663	0.95	0	12796	25128	0.19%	0.34%	11186	16048	23168	27088	*bchH*	Photosynthesis	Biosynthesis of Bacteriochlorophyll
RCAP_rcc00664	0.99	0	3986	8030	0.06%	0.11%	3658	4853	7319	8742	*bchB*	Photosynthesis	Biosynthesis of Bacteriochlorophyll
RCAP_rcc00665	1.21	0	3057	7178	0.05%	0.10%	2864	3679	6471	7885	*bchN*	Photosynthesis	Biosynthesis of Bacteriochlorophyll
RCAP_rcc00666	1.13	0	3359	7618	0.05%	0.10%	2898	4300	6230	9005	*bchF*	Photosynthesis	Biosynthesis of Bacteriochlorophyll
RCAP_rcc00667	0.52	0.0012	4622	6700	0.07%	0.09%	3861	5589	6047	7352	*ppaA*	Signal Transduction	Transcription Regulator
RCAP_rcc00668	0.11	0.3465	4302	4653	0.06%	0.06%	3809	4770	4343	4963	*ppsR*	Signal Transduction	Transcription Regulator
RCAP_rcc00669	1.09	0	15477	33649	0.23%	0.46%	14273	19069	29076	38221	*bchE*	Photosynthesis	Biosynthesis of Bacteriochlorophyll
RCAP_rcc00670	1.09	0	1485	3206	0.02%	0.04%	1276	1855	3042	3369	*bchJ*	Photosynthesis	Biosynthesis of Bacteriochlorophyll
RCAP_rcc00671	1.01	0	2074	4235	0.03%	0.06%	1939	2480	3750	4719	*bchG*	Photosynthesis	Biosynthesis of Bacteriochlorophyll
RCAP_rcc00672	1	0	971	1958	0.01%	0.03%	856	1149	1831	2085	*PUCC family protein*	Photosynthesis	Light Harvesting Machinery
RCAP_rcc00673	1.12	0	3706	8168	0.06%	0.11%	3487	4520	7276	9060	*bchP*	Photosynthesis	Biosynthesis of Bacteriochlorophyll
RCAP_rcc00674	1.18	0	1239	2842	0.02%	0.04%	1157	1568	2608	3076	*idi2*	Photosynthesis	Terpenoid backbone biosynthesis
RCAP_rcc00675	0.94	0	357	699	0.01%	0.01%	310	427	586	812	*bchO*	Photosynthesis	Biosynthesis of Bacteriochlorophyll
RCAP_rcc00676	1.8	0	1012	3781	0.02%	0.05%	863	1365	3183	4378	*bchD*	Photosynthesis	Biosynthesis of Bacteriochlorophyll
RCAP_rcc00677	1.87	0	2278	8462	0.04%	0.11%	2502	2878	7546	9378	*bchI*	Photosynthesis	Biosynthesis of Bacteriochlorophyll
RCAP_rcc00678	2.29	0	919	4648	0.02%	0.06%	1024	1316	4100	5195	*crtA*	Photosynthesis	Biosynthesis of Spheroidene
RCAP_rcc00679	0.93	0	12136	23406	0.18%	0.32%	11519	14641	20267	26545	*crtI*	Photosynthesis	Biosynthesis of Spheroidene
RCAP_rcc00680	0.83	0.0026	1454	2718	0.02%	0.04%	996	2015	2320	3116	*crtB*	Photosynthesis	Biosynthesis of Spheroidene
RCAP_rcc00681	1.56	0	1274	3867	0.02%	0.05%	1241	1634	3333	4402	*tspO*	Signal Transduction	Kinase/Phosphorelay
RCAP_rcc00682	0.98	0	844	1678	0.01%	0.02%	780	996	1535	1820	*crtC*	Photosynthesis	Biosynthesis of Spheroidene
RCAP_rcc00683	0.99	0	636	1275	0.01%	0.02%	600	720	1172	1378	*crtD*	Photosynthesis	Biosynthesis of Spheroidene
RCAP_rcc00684	1.29	0	1745	4331	0.03%	0.06%	1595	2152	4064	4597	*crtE*	Photosynthesis	Biosynthesis of Spheroidene
RCAP_rcc00685	0.86	0	1731	3181	0.03%	0.04%	1585	2043	2842	3520	*crtF*	Photosynthesis	Biosynthesis of Spheroidene
RCAP_rcc00686	2.3	0	9549	49433	0.17%	0.67%	9861	13630	44817	54050	*bchC*	Photosynthesis	Biosynthesis of Bacteriochlorophyll
RCAP_rcc00687	1.44	0	4136	11570	0.06%	0.16%	3775	5364	10193	12947	*bchX*	Photosynthesis	Biosynthesis of Bacteriochlorophyll
RCAP_rcc00688	1.37	0	2420	6400	0.04%	0.09%	2104	3103	6047	6753	*bchY*	Photosynthesis	Biosynthesis of Bacteriochlorophyll
RCAP_rcc00689	1.32	0	4527	11517	0.07%	0.16%	4160	5622	10227	12808	*bchZ*	Photosynthesis	Biosynthesis of Bacteriochlorophyll
RCAP_rcc00690	1.86	0	323	1210	0.01%	0.02%	310	427	1116	1305	*pufQ*	Photosynthesis	Light Harvesting Machinery
RCAP_rcc00691	2.13	0	27214	126480	0.49%	1.72%	28796	40357	108511	144448	*pufB*	Photosynthesis	Light Harvesting Machinery

RCAP_rcc00692	2.17	0	21128	100184	0.38%	1.36%	22960	31131	85768	114600	*pufA*	Photosynthesis	Light Harvesting Machinery
RCAP_rcc00693	2.37	0	13547	74399	0.25%	1.01%	15256	20242	63700	85098	*pufL*	Photosynthesis	Light Harvesting Machinery
RCAP_rcc00694	2.32	0	13131	68952	0.24%	0.94%	14870	19604	60418	77486	*pufM*	Photosynthesis	Light Harvesting Machinery
RCAP_rcc00695	2.24	0	3319	16464	0.06%	0.22%	3764	5005	14429	18498	*pufX*	Photosynthesis	Light Harvesting Machinery
RCAP_rcc00696	2.13	0	1332	6201	0.02%	0.08%	1265	2007	5607	6796	*dxs1*	Photosynthesis	Terpenoid backbone biosynthesis
RCAP_rcc00697	-1.01	0	1126	547	0.02%	0.01%	942	1244	440	654	*hypothetical protein*	Unknown	Unknown
RCAP_rcc00698	0.38	0.2921	356	481	0.01%	0.01%	243	478	264	698	*hypothetical protein*	Unknown	Unknown
RCAP_rcc00699	0.84	0.0003	458	846	0.01%	0.01%	411	566	565	1128	*rpoE*	Replication, Recombination and Repair	Replication
RCAP_rcc00700	1.01	0.0001	369	784	0.01%	0.01%	298	479	540	1028	*hypothetical protein*	Unknown	Unknown
RCAP_rcc00701	-0.29	0.071	1049	856	0.01%	0.01%	898	1209	752	960	*bsaA1*	Sulfur Metabolism	Glutathione metabolism
RCAP_rcc00702	-0.27	0.3608	64	53	0.00%	0.00%	43	85	46	59	*hypothetical protein*	Unknown	Unknown
RCAP_rcc00703	-0.48	0.0053	1094	778	0.02%	0.01%	882	1277	656	900	*deoxyribodipyrimidine photolyase-related protein*	Unknown	Unknown
RCAP_rcc00704	0.02	0.9371	2002	2031	0.03%	0.03%	1420	2602	1866	2196	*oppC1*	Metal, Ion, Cofactor Transport	Nickel Transport
RCAP_rcc00705	-0.08	0.7298	3504	3300	0.05%	0.04%	2527	4518	2890	3709	*oppB1*	Metal, Ion, Cofactor Transport	Nickel Transport
RCAP_rcc00706	0.1	0.7241	25354	27243	0.36%	0.37%	17127	34511	23590	30897	*oppA1*	Unknown	Unknown
RCAP_rcc00707	0.33	0.0842	1589	2011	0.02%	0.03%	1171	2038	1872	2150	*oppF*	Amino Acid Metabolism	Amino Acid Transport
RCAP_rcc00708	0.63	0.0009	1798	2827	0.03%	0.04%	1378	2262	2432	3221	*oppD1*	Amino Acid Metabolism	Amino Acid Transport
RCAP_rcc00709	1.04	0	559	1177	0.01%	0.02%	473	666	972	1381	*FAD dependent oxidoreductase*	Energy Metabolism	Unknown
RCAP_rcc00710	1.14	0	2640	5903	0.04%	0.08%	2342	3195	5268	6539	*hipO*	Amino Acid Metabolism	Phenylalanine metabolism
RCAP_rcc00711	1.53	0	1646	4903	0.03%	0.07%	1545	2182	4476	5330	*universal stress family protein*	Stress Response	Unknown
RCAP_rcc00712	0.19	0.3216	1038	1186	0.01%	0.02%	901	1185	968	1404	*moaA1*	Metabolism of Cofactors, Coenzymes and Vitamins	Unknown
RCAP_rcc00713	0.75	0.0004	411	708	0.01%	0.01%	323	515	593	824	*kynU*	Amino Acid Metabolism	Tryptophan metabolism
RCAP_rcc00714	0.65	0.0711	117	195	0.00%	0.00%	74	157	139	252	*hypothetical protein*	Unknown	Unknown
RCAP_rcc00715	0.33	0.0738	3714	4695	0.05%	0.06%	3271	4352	4057	5333	*pntA*	Metabolism of Cofactors, Coenzymes and Vitamins	Nicotinate and nicotinamide metabolism
RCAP_rcc00716	0.11	0.538	5368	5793	0.08%	0.08%	4701	6212	5133	6454	*pntB*	Metabolism of Cofactors, Coenzymes and Vitamins	Nicotinate and nicotinamide metabolism
RCAP_rcc00717	0	0.9981	239	238	0.00%	0.00%	217	258	204	273	*hypothetical protein*	Unknown	Unknown
RCAP_rcc00718	0.15	0.2489	10294	11456	0.15%	0.16%	9309	11597	10389	12522	*mdh*	Carbohydrate Metabolism	TCA Cycle
RCAP_rcc00719	0.15	0.2878	115	127	0.00%	0.00%	99	128	113	141	*hypothetical protein*	Unknown	Unknown
RCAP_rcc00720	-0.15	0.5728	8113	7292	0.11%	0.10%	6017	10150	6339	8246	*sucC*	Carbohydrate Metabolism	TCA Cycle
RCAP_rcc00721	-0.43	0.0093	6345	4673	0.09%	0.06%	5273	7266	4278	5068	*sucD*	Carbohydrate Metabolism	TCA Cycle
RCAP_rcc00722	-0.27	0.3654	599	488	0.01%	0.01%	391	786	385	591	*lipoprotein*	Predicted Function	Unknown
RCAP_rcc00723	-1.19	0	538	233	0.01%	0.00%	445	598	214	251	*lipoprotein*	Predicted Function	Unknown
RCAP_rcc00724	-1.52	0	27478	9168	0.37%	0.12%	21791	31528	7557	10780	*sucA*	Carbohydrate Metabolism	TCA Cycle
RCAP_rcc00725	-1.61	0	7901	2500	0.11%	0.03%	6614	8708	2112	2888	*sucB*	Carbohydrate Metabolism	TCA Cycle
RCAP_rcc00726	-1.55	0	8317	2766	0.11%	0.04%	7052	9078	2331	3200	*lpdA1*	Carbohydrate Metabolism	TCA Cycle
RCAP_rcc00727	-1.28	0	852	342	0.01%	0.00%	709	943	285	399	*citE1*	Carbohydrate Metabolism	Glyoxylate and dicarboxylate metabolism
RCAP_rcc00728	-0.01	0.968	2210	2197	0.03%	0.03%	1835	2633	1923	2472	*NnrU family protein*	Energy Metabolism	Nitrogen Metabolism
RCAP_rcc00729	0.35	0.092	288	373	0.00%	0.01%	240	357	336	410	*hypothetical protein*	Unknown	Unknown
RCAP_rcc00730	-0.21	0.0143	2496	2151	0.03%	0.03%	2270	2684	2019	2282	*maoC*	Carbohydrate Metabolism	Glyoxylate and dicarboxylate metabolism
RCAP_rcc00731	0.22	0.2102	2933	3430	0.04%	0.05%	2430	3512	3054	3806	*sdhC*	Carbohydrate Metabolism	TCA Cycle
RCAP_rcc00732	-0.06	0.7653	3034	2914	0.04%	0.04%	2530	3577	2616	3213	*sdhD*	Carbohydrate Metabolism	TCA Cycle
RCAP_rcc00733	-0.26	0.0028	11393	9514	0.16%	0.13%	10420	12213	8977	10052	*sdhA*	Carbohydrate Metabolism	TCA Cycle
RCAP_rcc00734	-0.14	0.7237	224	201	0.00%	0.00%	134	304	138	264	*lipoprotein*	Predicted Function	Unknown
RCAP_rcc00735	-0.04	0.9151	568	551	0.01%	0.01%	395	711	372	731	*hypothetical protein*	Unknown	Unknown
RCAP_rcc00736	-0.38	0.0287	5779	4422	0.08%	0.06%	4666	6832	3901	4943	*sdhB*	Carbohydrate Metabolism	TCA Cycle
RCAP_rcc00737	1.69	0	359	1306	0.01%	0.02%	293	555	939	1674	*hypothetical protein*	Unknown	Unknown
RCAP_rcc00738	0.09	0.5458	549	587	0.01%	0.01%	472	625	528	646	*hypothetical protein*	Unknown	Unknown
RCAP_rcc00739	0.3	0.094	455	562	0.01%	0.01%	381	519	470	654	*ArsR family transcriptional regulator*	Signal Transduction	Transcription Regulator
RCAP_rcc00740	-0.46	0.0691	2439	1743	0.03%	0.02%	1678	3093	1388	2098	*atpI*	Unknown	Unknown
RCAP_rcc00741	-0.25	0.2819	8191	6852	0.12%	0.09%	6506	9908	5500	8205	*atpB*	Energy Metabolism	Methane metabolism
RCAP_rcc00742	-0.1	0.5758	9736	9096	0.14%	0.12%	8059	11267	8117	10075	*atpE*	Energy Metabolism	Methane metabolism
RCAP_rcc00743	-0.12	0.3608	20247	18573	0.28%	0.25%	18237	22280	16717	20429	*atpX*	Energy Metabolism	Methane metabolism
RCAP_rcc00744	-0.3	0.0314	17441	14158	0.24%	0.19%	15135	19404	12879	15436	*atpF*	Energy Metabolism	Methane metabolism
RCAP_rcc00745	0.39	0.0106	4032	5316	0.06%	0.07%	3576	4873	4676	5956	*phaZ*	Lipid Metabolism	Unknown
RCAP_rcc00746	-0.26	0.2007	3885	3221	0.05%	0.04%	3160	4602	2671	3770	*phbC*	Carbohydrate Metabolism	Polyhydroxybutyrate
RCAP_rcc00747	1.13	0.0001	9180	21588	0.15%	0.29%	7777	14045	16277	26898	*hypothetical protein*	Unknown	Unknown
RCAP_rcc00748	-0.15	0.4114	2304	2063	0.03%	0.03%	1863	2746	1790	2337	*phaR*	Carbohydrate Metabolism	Polyhydroxybutyrate
RCAP_rcc00749	0.59	0.0354	311	484	0.00%	0.01%	223	406	355	613	*LysR family transcriptional regulator*	Signal Transduction	Transcription Regulator
RCAP_rcc00750	-0.85	0	4624	2528	0.06%	0.03%	3962	5146	2177	2879	*pyrG*	Nucleotide Metabolism	Pyrimidine metabolism
RCAP_rcc00751	0.22	0.348	429	505	0.01%	0.01%	312	543	453	556	*secG*	Trafficking and Secretion	Secretion
RCAP_rcc00752	-0.13	0.471	3070	2796	0.04%	0.04%	2617	3526	2478	3113	*purA*	Amino Acid Metabolism	Alanine, aspartate and glutamate metabolism
RCAP_rcc00753	-0.18	0.5036	154	134	0.00%	0.00%	111	189	105	164	*hypothetical protein*	Unknown	Unknown
RCAP_rcc00754	-0.09	0.5674	2067	1942	0.03%	0.03%	1815	2279	1687	2198	*lipoprotein*	Predicted Function	Unknown
RCAP_rcc00755	0.61	0.0001	241	370	0.00%	0.01%	221	261	302	439	*thiN*	Metabolism of Cofactors, Coenzymes and Vitamins	Thiamine metabolism
RCAP_rcc00756	-0.11	0.498	481	445	0.01%	0.01%	412	543	398	493	*dme family transporter*	Predicted Function	Replication
RCAP_rcc00757	0.29	0.1741	445	547	0.01%	0.01%	341	553	487	607	*alpha/beta fold family hydrolase*	Photosynthesis	Tropane, piperidine and pyridine alkaloid biosynthesis
RCAP_rcc00758	0.12	0.6099	162	176	0.00%	0.00%	126	193	141	211	*alpha/beta fold family hydrolase*	Unknown	Unknown
RCAP_rcc00759	-0.17	0.1108	2272	2021	0.03%	0.03%	2022	2498	1912	2131	*mcpB*	Motility	Chemotaxis
RCAP_rcc00760	-0.84	0	1347	749	0.02%	0.01%	1174	1446	685	812	*mcpA1*	Motility	Chemotaxis
RCAP_rcc00761	0.14	0.57	565	625	0.01%	0.01%	418	704	534	716	*hoxH*	Energy Metabolism	Methane metabolism
RCAP_rcc00762	0.04	0.9086	167	172	0.00%	0.00%	103	225	139	206	*hoxW*	Post-translational Modification, Assembly and Chaperones	Unknown
RCAP_rcc00763	0.53	0	641	931	0.01%	0.01%	577	720	854	1008	*hupT*	Energy Metabolism	Aerobic/Anaerobic Respiration
RCAP_rcc00764	0.22	0.3432	252	295	0.00%	0.00%	199	299	241	350	*hupU*	Energy Metabolism	Aerobic/Anaerobic Respiration
RCAP_rcc00765	0.02	0.9549	214	216	0.00%	0.00%	164	253	164	269	*hupV*	Energy Metabolism	Aerobic/Anaerobic Respiration
RCAP_rcc00766	-0.22	0.1036	248	212	0.00%	0.00%	219	270	185	239	*hypF*	Energy Metabolism	Aerobic/Anaerobic Respiration
RCAP_rcc00767	-1.76	0	2632	735	0.04%	0.01%	1951	3143	638	832	*hupA*	Energy Metabolism	Aerobic/Anaerobic Respiration
RCAP_rcc00768	-2.35	0	7530	1360	0.10%	0.02%	5702	8758	1133	1586	*hupB*	Energy Metabolism	Aerobic/Anaerobic Respiration

RCAP_rcc00769	-2.56	0	2870	439	0.04%	0.01%	2075	3422	350	528	*hupC*	Energy Metabolism	Aerobic/Anaerobic Respiration
RCAP_rcc00770	-0.96	0	804	409	0.01%	0.01%	733	849	356	463	*hupD*	Energy Metabolism	Aerobic/Anaerobic Respiration
RCAP_rcc00771	-0.75	0.0383	60	33	0.00%	0.00%	34	81	22	44	*hupF*	Energy Metabolism	Aerobic/Anaerobic Respiration
RCAP_rcc00772	-1.37	0	361	138	0.00%	0.00%	310	392	124	153	*hupG*	Energy Metabolism	Aerobic/Anaerobic Respiration
RCAP_rcc00773	-1.29	0	909	369	0.01%	0.01%	770	993	335	402	*hupH*	Energy Metabolism	Aerobic/Anaerobic Respiration
RCAP_rcc00774	-1.53	0	1297	441	0.02%	0.01%	1104	1402	380	502	*hupJ*	Energy Metabolism	Aerobic/Anaerobic Respiration
RCAP_rcc00775	-1.08	0.0034	129	54	0.00%	0.00%	67	180	36	71	*hupK*	Energy Metabolism	Aerobic/Anaerobic Respiration
RCAP_rcc00776	-1.03	0	505	247	0.01%	0.00%	452	527	228	265	*hypA*	Energy Metabolism	Aerobic/Anaerobic Respiration
RCAP_rcc00777	-0.98	0	1378	695	0.02%	0.01%	1251	1449	629	762	*hypB*	Energy Metabolism	Aerobic/Anaerobic Respiration
RCAP_rcc00778	-1.04	0	984	475	0.01%	0.01%	884	1029	440	511	*hupR*	Signal Transduction	Transcription Regulator
RCAP_rcc00779	-1	0	298	148	0.00%	0.00%	261	320	134	162	*hypC*	Energy Metabolism	Aerobic/Anaerobic Respiration
RCAP_rcc00780	-1.02	0	997	489	0.01%	0.01%	881	1062	447	532	*hypD*	Energy Metabolism	Aerobic/Anaerobic Respiration
RCAP_rcc00781	-0.69	0.0107	145	87	0.00%	0.00%	97	183	65	108	*hypE*	Energy Metabolism	Aerobic/Anaerobic Respiration
RCAP_rcc00782	0.06	0.2738	1804	1880	0.03%	0.03%	1736	1869	1803	1958	*cheR1*	Motility	Chemotaxis
RCAP_rcc00783	0.25	0.2698	551	657	0.01%	0.01%	443	657	554	761	*diguanylate cyclase/phosphodiesterase*	Signal Transduction	Kinase/Phosphorelay
RCAP_rcc00784	-1.02	0	129	61	0.00%	0.00%	86	163	48	73	*hypothetical protein*	Unknown	Unknown
RCAP_rcc00785	-1.09	0	1400	642	0.02%	0.01%	1068	1660	552	733	*sqr*	Energy Metabolism	Unknown
RCAP_rcc00786	0.24	0.0615	8437	9993	0.12%	0.14%	7622	9632	8988	10999	*basic membrane lipoprotein family*	Predicted Function	Unknown
RCAP_rcc00787	0.04	0.908	568	586	0.01%	0.01%	386	729	450	722	*monosacharide ABC transporter permease*	Carbohydrate Metabolism	Aerobic/Anaerobic Respiration
RCAP_rcc00788	-0.25	0.0067	1496	1257	0.02%	0.02%	1352	1616	1182	1332	*monosacharide ABC transporter permease*	Carbohydrate Metabolism	Aerobic/Anaerobic Respiration
RCAP_rcc00789	0.02	0.8951	1218	1238	0.02%	0.02%	1039	1373	1128	1348	*monosacharide ABC transporter ATP-binding protein*	Carbohydrate Metabolism	Aerobic/Anaerobic Respiration
RCAP_rcc00790	-0.34	0.0881	303	237	0.00%	0.00%	236	361	197	277	*xdhC*	Post-translational Modification, Assembly and Chaperones	Unknown
RCAP_rcc00791	-0.09	0.6654	1816	1700	0.03%	0.02%	1414	2200	1469	1931	*xdhB*	Nucleotide Metabolism	Purine metabolism
RCAP_rcc00792	0.37	0.1311	1215	1590	0.02%	0.02%	828	1599	1331	1849	*xdhA*	Nucleotide Metabolism	Unknown
RCAP_rcc00793	-0.43	0.0001	4937	3648	0.07%	0.05%	4338	5352	3353	3943	*dnaE1*	Replication, Recombination and Repair	Replication
RCAP_rcc00794	0.15	0.4382	167	186	0.00%	0.00%	136	196	161	211	*SlyX family protein*	Unknown	Unknown
RCAP_rcc00795	0.26	0.1423	1376	1654	0.02%	0.02%	1193	1610	1498	1811	*hisS*	Translation, ribosomal structure and biogenesis	Aminoacyl-tRNA biosynthesis
RCAP_rcc00796	-0.13	0.4441	559	509	0.01%	0.01%	477	633	461	558	*hisZ*	Signal Transduction	Transcription Regulator
RCAP_rcc00797	0.04	0.843	614	630	0.01%	0.01%	531	697	560	700	*hisG*	Amino Acid Metabolism	Histidine metabolism
RCAP_rcc00798	-0.08	0.7296	393	372	0.01%	0.01%	316	466	323	420	*D-isomer specific 2-hydroxyacid dehydrogenase*	Carbohydrate Metabolism	Glyoxylate and dicarboxylate metabolism
RCAP_rcc00799	-0.53	0.0075	1430	980	0.02%	0.01%	1163	1669	817	1143	*mrdB*	Cell Envelope Biosynthesis	Cell Wall Biosynthesis
RCAP_rcc00800	-0.2	0.0505	831	724	0.01%	0.01%	751	898	671	776	*mrdA*	Cell Envelope Biosynthesis	Cell Wall Biosynthesis
RCAP_rcc00801	-0.28	0.0916	171	141	0.00%	0.00%	143	195	126	155	*hypothetical protein*	Unknown	Unknown
RCAP_rcc00802	-0.16	0.3554	645	576	0.01%	0.01%	547	745	523	630	*mreC*	Cell Envelope Biosynthesis	Cell Wall Biosynthesis
RCAP_rcc00803	0.08	0.557	1796	1902	0.03%	0.03%	1612	2045	1745	2059	*mreB*	Cell Division	Chromosome Partitioning
RCAP_rcc00804	1.22	0	375	899	0.01%	0.01%	375	470	743	1056	*nhaA*	Metal and Ion Transport	Unknown
RCAP_rcc00805	0.87	0	106	195	0.00%	0.00%	97	120	176	214	*GNAT family acetyltransferase*	Cell Division	Chromosome Partitioning
RCAP_rcc00806	0.84	0	382	694	0.01%	0.01%	335	461	630	759	*heat shock protein DnaJ domain-containing protein*	Stress Response	Unknown
RCAP_rcc00807	0.23	0.0262	329	386	0.00%	0.01%	299	364	360	411	*endonuclease/exonuclease/phosphatase*	Unknown	Unknown
RCAP_rcc00808	1.97	0	334	1503	0.01%	0.02%	299	481	875	2130	*hypothetical protein*	Unknown	Unknown
RCAP_rcc00809	-0.33	0.0044	3205	2542	0.04%	0.03%	2794	3505	2319	2765	*ppx*	Nucleotide Metabolism	Purine metabolism
RCAP_rcc00810	-0.25	0.1201	5768	4814	0.08%	0.07%	4783	6791	4276	5353	*ppk*	Energy Metabolism	Oxidative phosphorylation
RCAP_rcc00811	0.58	0.0444	117	180	0.00%	0.00%	87	148	126	233	*hda*	Replication, Recombination and Repair	Unknown
RCAP_rcc00812	1.14	0	768	1707	0.01%	0.02%	822	876	1535	1879	*hypothetical protein*	Unknown	Unknown
RCAP_rcc00813	-0.17	0.4647	112	100	0.00%	0.00%	86	136	86	114	*hypothetical protein*	Unknown	Unknown
RCAP_rcc00814	0.8	0.0026	43	77	0.00%	0.00%	33	54	57	98	*LysR family transcriptional regulator*	Signal Transduction	Transcription Regulator
RCAP_rcc00815	0.04	0.8984	22	23	0.00%	0.00%	17	27	18	27	*DoxX family protein*	Unknown	Unknown
RCAP_rcc00816	-0.03	0.8747	507	498	0.01%	0.01%	442	563	456	541	*extradiol ring-cleavage dioxygenase subunit B*	Unknown	Unknown
RCAP_rcc00817	0.66	0.0553	298	499	0.00%	0.01%	193	398	373	625	*ldh*	Carbohydrate Metabolism	Glycolysis / Gluconeogenesis
RCAP_rcc00818	0.35	0.0007	1714	2184	0.02%	0.03%	1571	1933	2056	2312	*ftsW*	Cell Division	Chromosome Partitioning
RCAP_rcc00819	0.31	0.2484	374	471	0.01%	0.01%	282	471	397	545	*murG*	Glycan Biosynthesis and Metabolism	Peptidoglycan biosynthesis
RCAP_rcc00820	-0.23	0.0408	1849	1572	0.03%	0.02%	1673	2020	1426	1718	*murC*	Glycan Biosynthesis and Metabolism	Peptidoglycan biosynthesis
RCAP_rcc00821	-0.31	0.1239	68	54	0.00%	0.00%	53	82	46	61	*hypothetical protein*	Unknown	Unknown
RCAP_rcc00822	-0.02	0.9103	409	405	0.01%	0.01%	361	453	379	430	*murB*	Cell Envelope Biosynthesis	Cell Wall Biosynthesis
RCAP_rcc00823	0.35	0	1855	2369	0.03%	0.03%	1715	2034	2273	2465	*ddl*	Glycan Biosynthesis and Metabolism	Peptidoglycan biosynthesis
RCAP_rcc00824	-0.01	0.9708	677	672	0.01%	0.01%	536	806	547	798	*ftsQ*	Cell Envelope Biosynthesis	Cell Wall Biosynthesis
RCAP_rcc00825	-0.36	0.0017	3764	2928	0.05%	0.04%	3296	4151	2714	3141	*ftsA*	Cell Division	Chromosome Partitioning
RCAP_rcc00826	-0.27	0.0227	14120	11706	0.20%	0.16%	12390	15665	10781	12630	*ftsZ*	Unknown	Unknown
RCAP_rcc00827	0.75	0	1695	2879	0.03%	0.04%	1543	2032	2561	3197	*lpxC*	Xenobiotics Biodegradation and Metabolism	Caprolactam degradation
RCAP_rcc00828	-0.4	0.019	3477	2608	0.05%	0.04%	2857	4028	2244	2972	*comL*	Cell Envelope Biosynthesis	Cell Wall Biosynthesis
RCAP_rcc00829	-0.32	0.0625	1105	878	0.02%	0.01%	902	1246	743	1013	*recN*	Replication, Recombination and Repair	Recombination
RCAP_rcc00830	0.16	0.3091	91	101	0.00%	0.00%	79	102	89	114	*hypothetical protein*	Unknown	Unknown
RCAP_rcc00831	0.48	0.0001	560	784	0.01%	0.01%	494	641	733	835	*dld*	Carbohydrate Metabolism	Pyruvate metabolism
RCAP_rcc00832	0.19	0.2338	2828	3237	0.04%	0.04%	2487	3288	3001	3474	*pta*	Energy Metabolism	Reductive carboxylate cycle (CO2 fixation)
RCAP_rcc00833	0.2	0.1498	1939	2237	0.03%	0.03%	1741	2211	2027	2448	*ackA1*	Energy Metabolism	Reductive carboxylate cycle (CO2 fixation)
RCAP_rcc00834	-0.3	0.062	2010	1621	0.03%	0.02%	1681	2271	1424	1818	*pepP*	Amino Acid Metabolism	Unknown
RCAP_rcc00835	-0.46	0	7401	5379	0.10%	0.07%	6702	7860	5073	5684	*cobT*	Metabolism of Cofactors, Coenzymes and Vitamins	Cobalamin Biosynthesis
RCAP_rcc00836	-0.28	0.082	9837	8037	0.14%	0.11%	8298	11312	6982	9092	*cobS*	Metabolism of Cofactors, Coenzymes and Vitamins	Cobalamin Biosynthesis
RCAP_rcc00837	0.53	0.0759	117	174	0.00%	0.00%	72	167	148	201	*hypothetical protein*	Unknown	Unknown
RCAP_rcc00838	-0.02	0.9535	1217	1203	0.02%	0.02%	910	1550	1041	1364	*DnaJ domain-containing protein*	Post-translational Modification, Assembly and Chaperones	Unknown
RCAP_rcc00839	-0.3	0	3025	2457	0.04%	0.03%	2851	3155	2323	2590	*glcB*	Carbohydrate Metabolism	Glyoxylate and dicarboxylate metabolism
RCAP_rcc00840	0.29	0.0024	1624	1989	0.02%	0.03%	1494	1781	1841	2138	*pepN*	Metabolism of Other Amino Acids	Glutathione metabolism
RCAP_rcc00841	-0.33	0.1143	7418	5832	0.10%	0.08%	5793	8928	4938	6727	*gatB*	Translation, ribosomal structure and biogenesis	Aminoacyl-tRNA biosynthesis
RCAP_rcc00842	0.35	0.2405	1181	1538	0.02%	0.02%	866	1527	1201	1874	*hypothetical protein*	Unknown	Unknown
RCAP_rcc00843	-0.07	0.6261	295	281	0.00%	0.00%	263	324	257	306	*hypothetical protein*	Unknown	Unknown
RCAP_rcc00844	0.33	0.15	363	462	0.01%	0.01%	293	432	370	553	*hypothetical protein*	Unknown	Unknown
RCAP_rcc00845	0	0.9984	2502	2503	0.04%	0.03%	2103	3002	2141	2866	*hypothetical protein*	Unknown	Unknown

RCAP_rcc00846	-1.15	0	30290	13442	0.41%	0.18%	26213	32839	11562	15323	*dppA*	Unknown	Unknown
RCAP_rcc00847	-0.71	0	2801	1706	0.04%	0.02%	2497	2980	1528	1884	*dppB*	Metal, Ion, Cofactor Transport	Nickel Transport
RCAP_rcc00848	-0.43	0.0054	1152	850	0.02%	0.01%	953	1311	762	938	*dppC*	Metal, Ion, Cofactor Transport	Nickel Transport
RCAP_rcc00849	-0.37	0.0013	1795	1390	0.03%	0.02%	1625	1943	1251	1530	*dppD*	Predicted Function	Nickel Transport
RCAP_rcc00850	-0.2	0.1782	1056	915	0.01%	0.01%	907	1196	822	1007	*dppF*	Amino Acid Metabolism	Amino Acid Transport
RCAP_rcc00851	-0.29	0.1074	1658	1350	0.02%	0.02%	1365	1921	1203	1497	*membrane dipeptidase*	Cell Envelope Biosynthesis	Cell Wall Biosynthesis
RCAP_rcc00852	0	0.9734	3619	3610	0.05%	0.05%	3352	3918	3388	3833	*srmB*	Replication, Recombination and Repair	Unknown
RCAP_rcc00853	0.54	0.0089	100	146	0.00%	0.00%	81	118	119	173	*hypothetical protein*	Unknown	Unknown
RCAP_rcc00854	-0.23	0.0955	5186	4404	0.07%	0.06%	4494	5775	3933	4874	*argS*	Translation, ribosomal structure and biogenesis	Aminoacyl-tRNA biosynthesis
RCAP_rcc00855	0.29	0.3949	687	861	0.01%	0.01%	467	887	621	1101	*sporulation domain-containing protein*	Unknown	Unknown
RCAP_rcc00856	-0.25	0.1285	415	346	0.01%	0.00%	346	472	304	389	*nagZ*	Glycan Biosynthesis and Metabolism	Glycosphingolipid biosynthesis - ganglio series
RCAP_rcc00857	-0.75	0	567	332	0.01%	0.00%	461	647	281	383	*scpA*	Unknown	Unknown
RCAP_rcc00858	-0.45	0.0003	370	270	0.01%	0.00%	322	406	243	296	*scpB*	Transcription	Unknown
RCAP_rcc00859	-0.41	0.0054	79	59	0.00%	0.00%	68	90	52	66	*hypothetical protein*	Unknown	Unknown
RCAP_rcc00860	-0.66	0.0249	160	97	0.00%	0.00%	100	209	72	121	*enoyl-CoA hydratase/isomerase*	Xenobiotics Biodegradation and Metabolism	Caprolactam degradation
RCAP_rcc00861	-0.01	0.9771	84	84	0.00%	0.00%	62	112	68	99	*thioesterase*	Secondary metabolites biosynthesis, transport, and catabolism	Unknown
RCAP_rcc00862	1.18	0.0001	53	130	0.00%	0.00%	37	71	94	166	*divalent anion:Na+ symporter*	Metal and Ion Transport	Replication
RCAP_rcc00863	-0.14	0.5753	6846	6195	0.10%	0.08%	5546	8135	5073	7316	*rplM*	Translation, ribosomal structure and biogenesis	Unknown
RCAP_rcc00864	-0.14	0.5341	3414	3085	0.05%	0.04%	2719	4067	2661	3508	*rpsI*	Translation, ribosomal structure and biogenesis	Unknown
RCAP_rcc00865	0.44	0	1040	1410	0.01%	0.02%	969	1124	1336	1485	*hemolysin-type calcium-binding repeat family protein*	Trafficking and Secretion	Secretion
RCAP_rcc00866	0.14	0.6674	72	79	0.00%	0.00%	49	93	63	96	*argO*	Unknown	Unknown
RCAP_rcc00867	0.72	0.0761	9	16	0.00%	0.00%	5	12	6	25	*LysR family transcriptional regulator*	Signal Transduction	Transcription Regulator
RCAP_rcc00868	-1.86	0	489	128	0.01%	0.00%	380	568	112	144	*hypothetical protein*	Unknown	Unknown
RCAP_rcc00869	-3.01	0	12630	1447	0.17%	0.02%	10226	13932	1217	1677	*adhC*	Carbohydrate Metabolism	Glycolysis / Gluconeogenesis
RCAP_rcc00870	-2.66	0	4411	662	0.06%	0.01%	3480	4970	611	713	*fghA*	Sulfur Metabolism	Glutathione metabolism
RCAP_rcc00871	1.34	0	22634	59714	0.37%	0.81%	22416	30368	47857	71571	*hypothetical protein*	Unknown	Unknown
RCAP_rcc00872	0.34	0.0424	353	452	0.00%	0.01%	296	407	380	524	*alcohol dehydrogenase*	Energy Metabolism	Unknown
RCAP_rcc00873	-0.04	0.8976	3011	2930	0.04%	0.04%	2306	3709	2357	3503	*XRE family transcriptional regulator*	Signal Transduction	Transcription Regulator
RCAP_rcc00874	0.67	0.0255	123	205	0.00%	0.00%	88	161	162	247	*inositol monophosphatase*	Amino Acid Metabolism	Histidine metabolism
RCAP_rcc00875	-0.02	0.8666	452	447	0.01%	0.01%	421	474	421	473	*atzB*	Metabolism of Cofactors, Coenzymes and Vitamins	Riboflavin metabolism
RCAP_rcc00876	-0.1	0.3998	1051	977	0.01%	0.01%	947	1127	868	1085	*guaD*	Nucleotide Metabolism	Purine metabolism
RCAP_rcc00877	-0.16	0.1584	1476	1317	0.02%	0.02%	1279	1627	1234	1400	*mgtE*	Metal, Ion, Cofactor Transport	Magnesium Transport
RCAP_rcc00878	0.01	0.9791	61	61	0.00%	0.00%	30	88	33	89	*5-formyltetrahydrofolate cyclo-ligase*	Metabolism of Cofactors, Coenzymes and Vitamins	One carbon pool by folate
RCAP_rcc00879	-0.1	0.628	1057	986	0.01%	0.01%	917	1190	803	1169	*phnA*	Metal and Ion Transport	Unknown
RCAP_rcc00880	-0.75	0	968	567	0.01%	0.01%	791	1101	514	620	*CHAP domain-containing protein*	Unknown	Unknown
RCAP_rcc00881	-0.16	0.2655	1074	960	0.01%	0.01%	927	1181	854	1066	*metallophosphoesterase*	Unknown	Unknown
RCAP_rcc00882	-0.11	0.0927	1417	1312	0.02%	0.02%	1332	1481	1247	1377	*divalent ion symporter*	Metal and Ion Transport	Replication
RCAP_rcc00883	0.13	0.6571	168	185	0.00%	0.00%	116	216	153	217	*lipoprotein*	Predicted Function	Unknown
RCAP_rcc00884	-0.24	0.3984	3151	2639	0.04%	0.04%	2215	4026	2091	3188	*hypothetical protein*	Unknown	Unknown
RCAP_rcc00885	0.54	0.0259	34	50	0.00%	0.00%	27	42	38	62	*hypothetical protein*	Unknown	Unknown
RCAP_rcc00886	0.66	0.0217	32	53	0.00%	0.00%	22	41	39	66	*ABC transporter ATP-binding protein*	Defense Mechanisms	Unknown
RCAP_rcc00887	0.75	0.003	84	147	0.00%	0.00%	59	109	113	180	*hypothetical protein*	Unknown	Unknown
RCAP_rcc00888	1.08	0.0002	7	16	0.00%	0.00%	6	9	11	22	*hypothetical protein*	Unknown	Unknown
RCAP_rcc00889	0.89	0.0027	33	65	0.00%	0.00%	24	42	42	88	*NosL family protein*	Unknown	Unknown
RCAP_rcc00890	0.93	0.0001	172	340	0.00%	0.00%	135	210	237	443	*hypothetical protein*	Unknown	Unknown
RCAP_rcc00891	1.44	0.0015	79	303	0.00%	0.00%	41	115	134	472	*hypothetical protein*	Unknown	Unknown
RCAP_rcc00892	-0.06	0.6924	887	848	0.01%	0.01%	749	1003	765	931	*hypothetical protein*	Unknown	Unknown
RCAP_rcc00893	-0.61	0	1956	1274	0.03%	0.02%	1783	2106	1100	1449	*proV1*	Amino Acid Metabolism	Amino Acid Transport
RCAP_rcc00894	-0.77	0	3113	1801	0.04%	0.02%	2615	3505	1491	2111	*proW1*	Unknown	Unknown
RCAP_rcc00895	-0.33	0.1452	8864	6998	0.12%	0.10%	6960	10664	5631	8364	*proX1*	Amino Acid Metabolism	Amino Acid Transport
RCAP_rcc00896	0.05	0.8577	437	452	0.01%	0.01%	334	538	397	508	*betI*	Signal Transduction	Transcription Regulator
RCAP_rcc00897	0.87	0.0001	1190	2249	0.02%	0.03%	1000	1547	2004	2494	*betB*	Amino Acid Metabolism	Glycine, serine and threonine metabolism
RCAP_rcc00898	0.87	0	1160	2168	0.02%	0.03%	1048	1489	1870	2466	*betA*	Amino Acid Metabolism	Glycine, serine and threonine metabolism
RCAP_rcc00899	0.36	0.0099	426	550	0.01%	0.01%	375	477	481	618	*nuclease*	Replication, Recombination and Repair	Unknown
RCAP_rcc00900	0.14	0.1669	280	309	0.00%	0.00%	254	305	286	332	*hypothetical protein*	Unknown	Unknown
RCAP_rcc00901	1.42	0	2983	8470	0.05%	0.12%	2294	4232	7454	9485	*hypothetical protein*	Unknown	Unknown
RCAP_rcc00902	0.11	0.5524	858	926	0.01%	0.01%	735	986	797	1054	*XRE family transcriptional regulator*	Signal Transduction	Transcription Regulator
RCAP_rcc00903	-0.39	0.2531	152	112	0.00%	0.00%	100	195	84	140	*hypothetical protein*	Unknown	Unknown
RCAP_rcc00904	-0.42	0.1054	745	546	0.01%	0.01%	529	933	457	635	*major facilitator superfamily protein*	Metal and Ion Transport	Unknown
RCAP_rcc00905	-0.07	0.757	361	343	0.01%	0.00%	286	439	296	390	*hypothetical protein*	Unknown	Unknown
RCAP_rcc00906	-0.4	0.041	979	737	0.01%	0.01%	810	1117	576	897	*pccB*	Carbohydrate Metabolism	Glyoxylate and dicarboxylate metabolism
RCAP_rcc00907	-0.15	0.5292	70	63	0.00%	0.00%	60	76	44	81	*hypothetical protein*	Unknown	Unknown
RCAP_rcc00908	-0.2	0.2512	16294	14090	0.23%	0.19%	13041	19168	12785	15396	*lipoprotein*	Predicted Function	Unknown
RCAP_rcc00909	0.44	0.0009	761	1036	0.01%	0.01%	681	876	934	1138	*hypothetical protein*	Unknown	Unknown
RCAP_rcc00910	0.03	0.9533	986	1017	0.01%	0.01%	232	1670	434	1599	*hypothetical protein*	Unknown	Unknown
RCAP_rcc00911	0.08	0.5701	2373	2508	0.03%	0.03%	2195	2575	2152	2865	*pccA*	Carbohydrate Metabolism	Glyoxylate and dicarboxylate metabolism
RCAP_rcc00912	-0.04	0.7372	1728	1680	0.02%	0.02%	1645	1804	1473	1888	*bhbA*	Carbohydrate Metabolism	Glyoxylate and dicarboxylate metabolism
RCAP_rcc00913	-0.56	0.0003	8262	5554	0.12%	0.08%	7172	9272	4749	6359	*bfr*	Metal, Ion, Cofactor Transport	Iron and Heme Transport
RCAP_rcc00914	-0.82	0.0085	508	270	0.01%	0.00%	387	636	139	402	*hypothetical protein*	Unknown	Unknown
RCAP_rcc00915	0.61	0	1232	1892	0.02%	0.03%	1200	1347	1757	2026	*psd*	Lipid Metabolism	Glycerophospholipid metabolism
RCAP_rcc00916	0.43	0.0002	1638	2216	0.02%	0.03%	1508	1851	2023	2409	*pssA*	Lipid Metabolism	Glycerophospholipid metabolism
RCAP_rcc00917	-0.61	0.0004	290	187	0.00%	0.00%	230	341	170	205	*hypothetical protein*	Unknown	Unknown
RCAP_rcc00918	-0.55	0.0204	71	47	0.00%	0.00%	56	83	37	57	*N-acetylmuramoyl-L-alanine amidase*	Cell Envelope Biosynthesis	Cell Wall Biosynthesis
RCAP_rcc00919	0.76	0.0028	53	93	0.00%	0.00%	42	68	70	115	*hypothetical protein*	Unknown	Unknown
RCAP_rcc00920	1.11	0	52	114	0.00%	0.00%	45	63	91	137	*hypothetical protein*	Unknown	Unknown
RCAP_rcc00921	0.66	0.0452	7	11	0.00%	0.00%	4	10	8	14	*NlpC/P60 family phage cell wall peptidase*	Replication, Recombination and Repair	Phage Interaction
RCAP_rcc00922	0.58	0.0106	13	19	0.00%	0.00%	11	15	15	23	*hypothetical protein*	Unknown	Unknown

RCAP_rcc00923	0.39	0.1673	5	7	0.00%	0.00%	4	7	6	8	*hypothetical protein*	Unknown	Unknown
RCAP_rcc00924	0.61	0.0165	6	10	0.00%	0.00%	5	8	8	12	*hypothetical protein*	Unknown	Unknown
RCAP_rcc00925	0.56	0.0135	19	29	0.00%	0.00%	16	21	22	36	*hypothetical protein*	Unknown	Unknown
RCAP_rcc00926	-0.03	0.9445	6	6	0.00%	0.00%	4	8	4	7	*hypothetical protein*	Unknown	Unknown
RCAP_rcc00927	-0.02	0.9318	15	15	0.00%	0.00%	12	18	13	17	*hypothetical protein*	Unknown	Unknown
RCAP_rcc00928	-0.72	0.0001	42	25	0.00%	0.00%	35	49	21	28	*hypothetical protein*	Unknown	Unknown
RCAP_rcc00929	-0.4	0.0006	100	75	0.00%	0.00%	89	109	69	81	*hypothetical protein*	Unknown	Unknown
RCAP_rcc00930	-0.26	0.2937	3982	3292	0.06%	0.04%	2947	4955	2689	3894	*hypothetical protein*	Unknown	Unknown
RCAP_rcc00931	0.6	0.0007	13	19	0.00%	0.00%	11	15	17	22	*hypothetical protein*	Unknown	Unknown
RCAP_rcc00932	1.19	0.005	1	3	0.00%	0.00%	1	1	2	4	*hypothetical protein*	Unknown	Unknown
RCAP_rcc00933	1	0.0163	1	3	0.00%	0.00%	1	2	2	5	*hypothetical protein*	Unknown	Unknown
RCAP_rcc00934	0.72	0	24	40	0.00%	0.00%	22	29	34	46	*U35 family peptidase*	Post-translational Modification, Assembly and Chaperones	Peptidase
RCAP_rcc00935	0.11	0.551	1279	1381	0.02%	0.02%	1096	1508	1224	1538	*hypothetical protein*	Unknown	Unknown
RCAP_rcc00936	-0.29	0.0531	659	539	0.01%	0.01%	531	783	509	569	*lambda family phage portal protein*	Replication, Recombination and Repair	Phage Interaction
RCAP_rcc00937	0.46	0.1709	4	6	0.00%	0.00%	3	6	5	7	*hypothetical protein*	Unknown	Unknown
RCAP_rcc00938	0.63	0.0004	28	43	0.00%	0.00%	23	34	39	48	*phage terminase large subunit*	Replication, Recombination and Repair	Phage Interaction
RCAP_rcc00939	0.02	0.9682	5	5	0.00%	0.00%	3	6	3	6	*hypothetical protein*	Unknown	Unknown
RCAP_rcc00940	-0.22	0.4121	11	9	0.00%	0.00%	8	14	8	11	*hypothetical protein*	Unknown	Unknown
RCAP_rcc00941	-0.13	0.202	385	352	0.01%	0.00%	354	412	321	383	*hypothetical protein*	Unknown	Unknown
RCAP_rcc00942	1.27	0	19	49	0.00%	0.00%	15	27	37	61	*hypothetical protein*	Unknown	Unknown
RCAP_rcc00943	1.26	0.0001	9	25	0.00%	0.00%	7	15	18	31	*cytosine-N(4)-specific DNA-methyltransferase*	Replication, Recombination and Repair	Unknown
RCAP_rcc00944	1.04	0.001	5	10	0.00%	0.00%	4	6	7	13	*ParB domain-containing protein nuclease*	Transcription	Unknown
RCAP_rcc00945	0.26	0.4062	4	5	0.00%	0.00%	3	5	4	6	*hypothetical protein*	Unknown	Unknown
RCAP_rcc00946	1.35	0	13	35	0.00%	0.00%	11	19	28	42	*MarR family transcriptional regulator*	Signal Transduction	Transcription Regulator
RCAP_rcc00947	1.28	0.0003	2	6	0.00%	0.00%	1	4	5	7	*hypothetical protein*	Unknown	Unknown
RCAP_rcc00948	1.1	0.0006	3	8	0.00%	0.00%	2	5	6	9	*hypothetical protein*	Unknown	Unknown
RCAP_rcc00949	-0.56	0.0118	910	607	0.01%	0.01%	706	1094	499	714	*hypothetical protein*	Unknown	Unknown
RCAP_rcc00950	-0.51	0.0003	136	95	0.00%	0.00%	118	154	86	104	*phage integrase*	Replication, Recombination and Repair	Phage Interaction
RCAP_rcc00951	-0.7	0.0035	7387	4414	0.10%	0.06%	5375	9103	3618	5211	*hsdR1*	Defense Mechanisms	Unknown
RCAP_rcc00952	-0.66	0.0013	2654	1644	0.04%	0.02%	2079	3141	1370	1917	*hsdM1*	Defense Mechanisms	Unknown
RCAP_rcc00953	-0.68	0.0017	1558	950	0.02%	0.01%	1222	1851	772	1127	*hypothetical protein*	Unknown	Unknown
RCAP_rcc00954	-0.69	0.0024	2599	1573	0.04%	0.02%	1985	3125	1274	1873	*hsdS1*	Defense Mechanisms	Unknown
RCAP_rcc00955	-0.71	0.0014	3572	2135	0.05%	0.03%	2774	4252	1710	2560	*SMC protein, N-terminal domain-containing protein*	Unknown	Unknown
RCAP_rcc00956	-0.89	0.0002	3093	1607	0.04%	0.02%	2275	3779	1274	1940	*hypothetical protein*	Unknown	Unknown
RCAP_rcc00957	-0.89	0.0002	356	185	0.00%	0.00%	258	435	147	223	*hypothetical protein*	Unknown	Unknown
RCAP_rcc00960	-0.59	0.0045	163	107	0.00%	0.00%	126	193	90	124	*IS111A/IS1328/IS1533 family transposase/IS116/IS110/IS902 family tra*	Replication, Recombination and Repair	Recombination
RCAP_rcc00961	0.07	0.6274	419	440	0.01%	0.01%	374	469	401	479	*hypothetical protein*	Unknown	Unknown
RCAP_rcc00962	0.2	0.5646	82	95	0.00%	0.00%	51	110	64	125	*hypothetical protein*	Unknown	Unknown
RCAP_rcc00963	0.05	0.7132	1544	1598	0.02%	0.02%	1422	1681	1439	1757	*hypothetical protein*	Unknown	Unknown
RCAP_rcc00964	0.46	0.0082	174	242	0.00%	0.00%	149	204	202	281	*phage tail fiber protein*	Replication, Recombination and Repair	Phage Interaction
RCAP_rcc00965	0.6	0.0463	22	35	0.00%	0.00%	17	29	24	46	*phage tail assembly protein*	Replication, Recombination and Repair	Phage Interaction
RCAP_rcc00966	0.83	0.0003	22	42	0.00%	0.00%	17	29	35	48	*hypothetical protein*	Unknown	Unknown
RCAP_rcc00967	0.88	0.0075	4	8	0.00%	0.00%	3	5	5	10	*hypothetical protein*	Unknown	Unknown
RCAP_rcc00968	0.05	0.8713	265	276	0.00%	0.00%	194	337	199	353	*hypothetical protein*	Unknown	Unknown
RCAP_rcc00969	0.09	0.7653	34	37	0.00%	0.00%	25	44	26	48	*hypothetical protein*	Unknown	Unknown
RCAP_rcc00970	-0.03	0.9284	116	114	0.00%	0.00%	87	145	79	148	*hypothetical protein*	Unknown	Unknown
RCAP_rcc00971	-0.02	0.9525	226	224	0.00%	0.00%	172	279	162	285	*hypothetical protein*	Unknown	Unknown
RCAP_rcc00972	0.1	0.8106	7	7	0.00%	0.00%	4	9	5	10	*hypothetical protein*	Unknown	Unknown
RCAP_rcc00973	0.4	0.1701	51	70	0.00%	0.00%	41	64	48	93	*hypothetical protein*	Unknown	Unknown
RCAP_rcc00974	0.29	0.3873	24	30	0.00%	0.00%	17	30	20	39	*hypothetical protein*	Unknown	Unknown
RCAP_rcc00975	0.01	0.9749	77	78	0.00%	0.00%	58	97	54	102	*cyclic nucleotide-binding domain-containing protein*	Unknown	Unknown
RCAP_rcc00976	-0.08	0.8133	130	123	0.00%	0.00%	98	159	87	160	*hypothetical protein*	Unknown	Unknown
RCAP_rcc00977	-0.23	0.4648	685	578	0.01%	0.01%	491	867	366	791	*hypothetical protein*	Unknown	Unknown
RCAP_rcc00978	0.29	0.394	57	71	0.00%	0.00%	42	73	48	94	*hypothetical protein*	Unknown	Unknown
RCAP_rcc00979	0.12	0.6644	334	365	0.00%	0.00%	270	401	262	468	*hypothetical protein*	Unknown	Unknown
RCAP_rcc00980	-0.09	0.7358	107	101	0.00%	0.00%	83	132	80	122	*phage virion morphogenesis protein*	Replication, Recombination and Repair	Phage Interaction
RCAP_rcc00981	0.53	0.0096	255	372	0.00%	0.01%	213	319	322	423	*phage head morphogenesis protein*	Replication, Recombination and Repair	Phage Interaction
RCAP_rcc00982	0.24	0.3112	103	124	0.00%	0.00%	86	121	89	158	*hypothetical protein*	Unknown	Unknown
RCAP_rcc00983	0.14	0.7006	35	39	0.00%	0.00%	23	47	28	49	*hypothetical protein*	Unknown	Unknown
RCAP_rcc00984	0.39	0.0564	59	78	0.00%	0.00%	47	74	69	87	*hypothetical protein*	Unknown	Unknown
RCAP_rcc00985	0.18	0.5185	142	163	0.00%	0.00%	110	176	112	214	*hypothetical protein*	Unknown	Unknown
RCAP_rcc00986	-0.13	0.7011	177	160	0.00%	0.00%	120	231	101	219	*hypothetical protein*	Unknown	Unknown
RCAP_rcc00987	-0.31	0.2588	74	59	0.00%	0.00%	57	89	41	78	*hypothetical protein*	Unknown	Unknown
RCAP_rcc00988	0.3	0.2638	93	116	0.00%	0.00%	73	113	80	152	*hypothetical protein*	Unknown	Unknown
RCAP_rcc00989	0.25	0.3992	232	280	0.00%	0.00%	167	300	187	374	*lysozyme*	Unknown	Unknown
RCAP_rcc00990	0.45	0.0005	172	236	0.00%	0.00%	153	188	207	266	*hypothetical protein*	Unknown	Unknown
RCAP_rcc00991	-0.3	0.122	51	42	0.00%	0.00%	43	58	32	51	*hypothetical protein*	Unknown	Unknown
RCAP_rcc00992	0.1	0.8806	1	1	0.00%	0.00%	0	1	0	1	*hypothetical protein*	Unknown	Unknown
RCAP_rcc00993	0.02	0.9235	22	23	0.00%	0.00%	19	25	19	27	*hypothetical protein*	Unknown	Unknown
RCAP_rcc00994	-0.18	0.3898	13	12	0.00%	0.00%	11	15	10	14	*hypothetical protein*	Unknown	Unknown
RCAP_rcc00995	-0.08	0.6226	120	115	0.00%	0.00%	107	135	101	129	*hypothetical protein*	Unknown	Unknown
RCAP_rcc00996	-0.09	0.6877	91	86	0.00%	0.00%	76	105	71	100	*hup2*	Energy Metabolism	Aerobic/Anaerobic Respiration
RCAP_rcc00997	-0.02	0.9527	30	30	0.00%	0.00%	28	34	23	38	*hypothetical protein*	Unknown	Unknown
RCAP_rcc00998	-0.31	0.17	108	87	0.00%	0.00%	85	126	66	107	*gam*	Unknown	Unknown
RCAP_rcc00999	0.05	0.8701	11	12	0.00%	0.00%	9	12	9	14	*hypothetical protein*	Unknown	Unknown
RCAP_rcc01000	0.29	0.1228	30	38	0.00%	0.00%	26	34	31	45	*hypothetical protein*	Unknown	Unknown
RCAP_rcc01001	0.29	0.1403	69	85	0.00%	0.00%	58	82	71	99	*bacteriophage DNA transposition B protein*	Replication, Recombination and Repair	Phage Interaction

RCAP_rcc01002	0.31	0.0709	215	269	0.00%	0.00%	178	255	243	295	*integrase catalytic subunit*	Replication, Recombination and Repair	Recombination
RCAP_rcc01003	0.03	0.906	33	35	0.00%	0.00%	28	39	28	42	*hypothetical protein*	Unknown	Unknown
RCAP_rcc01004	0.17	0.331	63	72	0.00%	0.00%	54	74	61	83	*ParB domain-containing protein nuclease*	Cell Division	Chromosome Partitioning
RCAP_rcc01005	0.49	0.2375	5	7	0.00%	0.00%	2	7	5	9	*hypothetical protein*	Unknown	Unknown
RCAP_rcc01006	0.01	0.9708	29	30	0.00%	0.00%	22	37	26	33	*hypothetical protein*	Unknown	Unknown
RCAP_rcc01007	0.44	0.1679	7	11	0.00%	0.00%	5	10	8	14	*hypothetical protein*	Unknown	Unknown
RCAP_rcc01008	0.35	0.2684	7	9	0.00%	0.00%	5	10	7	11	*lipoprotein*	Predicted Function	Unknown
RCAP_rcc01009	0.21	0.592	26	31	0.00%	0.00%	13	39	25	38	*hypothetical protein*	Unknown	Unknown
RCAP_rcc01010	-0.43	0.0385	2536	1859	0.04%	0.03%	1899	3086	1566	2152	*DNA binding protein*	Signal Transduction	Transcription Regulator
RCAP_rcc01011	-0.85	0	1951	1058	0.03%	0.01%	1532	2282	878	1238	*S24 family peptidase*	Post-translational Modification, Assembly and Chaperones	Peptidase
RCAP_rcc01012	0.44	0.0132	708	968	0.01%	0.01%	619	890	840	1096	*hypothetical protein*	Unknown	Unknown
RCAP_rcc01013	0.02	0.9284	280	284	0.00%	0.00%	239	333	244	325	*hypothetical protein*	Unknown	Unknown
RCAP_rcc01014	-0.24	0.121	461	389	0.01%	0.01%	397	522	346	432	*hypothetical protein*	Unknown	Unknown
RCAP_rcc01015	-0.29	0.1106	152	124	0.00%	0.00%	126	179	108	140	*hypothetical protein*	Unknown	Unknown
RCAP_rcc01019	-0.21	0.5257	26	22	0.00%	0.00%	17	34	18	26	*PHP domain-containing protein*	Unknown	Unknown
RCAP_rcc01020	0.7	0	158	260	0.00%	0.00%	147	185	230	291	*diguanylate cyclase/phosphodiesterase*	Signal Transduction	Kinase/Phosphorelay
RCAP_rcc01021	0.53	0.0013	23	33	0.00%	0.00%	20	25	28	38	*ugpC*	Amino Acid Metabolism	Unknown
RCAP_rcc01022	-0.21	0.3843	47	41	0.00%	0.00%	36	58	35	46	*ugpE*	Metal and Ion Transport	Unknown
RCAP_rcc01023	-0.25	0.0691	59	48	0.00%	0.00%	51	65	43	54	*ugpA*	Metal and Ion Transport	Unknown
RCAP_rcc01024	-0.2	0.3404	68	59	0.00%	0.00%	56	81	51	68	*ugpB*	Carbohydrate Metabolism	Unknown
RCAP_rcc01025	-0.05	0.9272	61	59	0.00%	0.00%	35	85	40	78	*regA2*	Signal Transduction	Transcription Regulator
RCAP_rcc01026	0.22	0.4506	169	198	0.00%	0.00%	123	211	151	246	*sensor histidine kinase*	Signal Transduction	Kinase/Phosphorelay
RCAP_rcc01027	0.24	0.6232	68	84	0.00%	0.00%	36	96	48	119	*hypothetical protein*	Unknown	Unknown
RCAP_rcc01028	-1.67	0	144	40	0.00%	0.00%	74	203	31	49	*iron siderophore/cobalamin ABC transporter periplasmic iron sideroph*	Metal, Ion, Cofactor Transport	Iron and Heme Transport
RCAP_rcc01029	-1.46	0.0001	20	6	0.00%	0.00%	11	28	4	9	*iron siderophore/cobalamin ABC transporter permease*	Metal, Ion, Cofactor Transport	Iron and Heme Transport
RCAP_rcc01030	-0.9	0.0477	8	3	0.00%	0.00%	4	11	1	5	*iron siderophore/cobalamin ABC transporter permease*	Metal, Ion, Cofactor Transport	Iron and Heme Transport
RCAP_rcc01031	-1.52	0.0001	49	14	0.00%	0.00%	27	66	8	19	*iron siderophore/cobalamin ABC transporter ATP-binding protein*	Metal, Ion, Cofactor Transport	Iron and Heme Transport
RCAP_rcc01032	-2.15	0	473	98	0.01%	0.00%	337	566	83	113	*cbiO1*	Metabolism of Cofactors, Coenzymes and Vitamins	Cobalamin Biosynthesis
RCAP_rcc01033	-1.9	0	692	172	0.01%	0.00%	497	827	142	201	*cbiQ1*	Metabolism of Cofactors, Coenzymes and Vitamins	Cobalamin Biosynthesis
RCAP_rcc01034	-1.67	0	2695	825	0.04%	0.01%	2164	3002	716	933	*CbiM family cobalamin biosynthesis protein*	Metabolism of Cofactors, Coenzymes and Vitamins	Cobalamin Biosynthesis
RCAP_rcc01035	-1.72	NA	488	125	0.01%	0.00%	84	858	114	136	*hypothetical protein*	Unknown	Unknown
RCAP_rcc01036	-0.41	0.4577	4	2	0.00%	0.00%	0	7	1	4	*hypothetical protein*	Unknown	Unknown
RCAP_rcc01037	-0.13	0.8419	6	5	0.00%	0.00%	2	11	1	10	*hypothetical protein*	Unknown	Unknown
RCAP_rcc01038	-1.64	NA	777	211	0.01%	0.00%	181	1321	189	233	*hypothetical protein*	Unknown	Unknown
RCAP_rcc01039	-0.75	0.0409	90	49	0.00%	0.00%	43	130	31	67	*hypothetical protein*	Unknown	Unknown
RCAP_rcc01040	-0.6	0.1831	86	51	0.00%	0.00%	38	129	30	72	*hypothetical protein*	Unknown	Unknown
RCAP_rcc01041	-1.08	0	301	138	0.00%	0.00%	228	356	113	162	*hypothetical protein*	Unknown	Unknown
RCAP_rcc01042	-1.01	0.0021	574	261	0.01%	0.00%	329	774	182	341	*copA1*	Metal, Ion, Cofactor Transport	Copper Transport
RCAP_rcc01043	-0.83	0.0096	533	279	0.01%	0.00%	362	683	171	387	*hypothetical protein*	Unknown	Unknown
RCAP_rcc01044	-0.16	0.7042	98	86	0.00%	0.00%	64	124	37	135	*RimK-like ATP-grasp domain-containing protein*	Unknown	Unknown
RCAP_rcc01045	-0.45	0.0908	48	34	0.00%	0.00%	39	54	21	47	*iron siderophore/cobalamin ABC transporter ATP-binding protein*	Metal, Ion, Cofactor Transport	Iron and Heme Transport
RCAP_rcc01046	-1.99	0	111	26	0.00%	0.00%	87	125	20	33	*iron siderophore/cobalamin ABC transporter permease*	Metal, Ion, Cofactor Transport	Iron and Heme Transport
RCAP_rcc01047	-2.22	0	280	56	0.00%	0.00%	214	321	43	69	*iron siderophore/cobalamin ABC transporter periplasmic iron sideroph*	Metal, Ion, Cofactor Transport	Iron and Heme Transport
RCAP_rcc01048	-0.22	0.5347	38	32	0.00%	0.00%	30	45	16	47	*AraC family transcriptional regulator*	Signal Transduction	Transcription Regulator
RCAP_rcc01049	0.83	0	93	168	0.00%	0.00%	86	115	138	199	*TonB-dependent siderophore receptor*	Metal, Ion, Cofactor Transport	Iron and Heme Transport
RCAP_rcc01050	1.17	NA	5	14	0.00%	0.00%	3	7	4	24	*fes*	Metal and Ion Transport	Unknown
RCAP_rcc01051	-0.78	0	328	188	0.00%	0.00%	294	349	164	213	*gvpN*	Motility	Gas Vesicle
RCAP_rcc01052	-0.55	0.2175	15	9	0.00%	0.00%	8	21	4	14	*hypothetical protein*	Unknown	Unknown
RCAP_rcc01053	-1.5	0	131	45	0.00%	0.00%	107	145	38	51	*gvpO*	Motility	Gas Vesicle
RCAP_rcc01054	-1.13	0	119	54	0.00%	0.00%	98	132	47	61	*gvpJ*	Motility	Gas Vesicle
RCAP_rcc01055	-0.98	0.0129	71	31	0.00%	0.00%	33	103	17	45	*hypothetical protein*	Unknown	Unknown
RCAP_rcc01056	-1.44	0	186	63	0.00%	0.00%	130	229	48	79	*gas vesicle synthesis protein GvpL/GvpF*	Motility	Gas Vesicle
RCAP_rcc01057	-1.9	0	186	48	0.00%	0.00%	153	204	40	57	*gvpG*	Motility	Gas Vesicle
RCAP_rcc01058	-1.34	0.001	66	21	0.00%	0.00%	29	97	10	31	*gas vesicle synthesis protein GvpL/GvpF*	Motility	Gas Vesicle
RCAP_rcc01059	-1.08	0.0088	24	9	0.00%	0.00%	9	36	4	15	*gas vesicle synthesis protein GvpL/GvpF*	Motility	Gas Vesicle
RCAP_rcc01060	-1.7	0	48	14	0.00%	0.00%	39	53	11	17	*gas vesicle protein GvpA*	Motility	Gas Vesicle
RCAP_rcc01061	-0.88	0.0003	64	33	0.00%	0.00%	49	75	23	43	*hypothetical protein*	Unknown	Unknown
RCAP_rcc01062	-1.06	0	68	32	0.00%	0.00%	60	72	28	37	*gvpK*	Motility	Gas Vesicle
RCAP_rcc01063	-1.75	0	195	56	0.00%	0.00%	150	224	49	63	*pcl*	Lipid Metabolism	Unknown
RCAP_rcc01064	-1.44	0	579	206	0.01%	0.00%	437	688	176	236	*hypothetical protein*	Unknown	Unknown
RCAP_rcc01065	-1.54	0	217	69	0.00%	0.00%	136	280	54	83	*hypothetical protein*	Unknown	Unknown
RCAP_rcc01066	-1.28	0	676	267	0.01%	0.00%	499	813	220	314	*pyp*	Unknown	Unknown
RCAP_rcc01067	-1.26	0	1057	425	0.01%	0.01%	820	1238	352	497	*hypothetical protein*	Unknown	Unknown
RCAP_rcc01068	0.15	0.5409	158	177	0.00%	0.00%	119	196	144	210	*hypothetical protein*	Unknown	Unknown
RCAP_rcc01069	-0.08	0.8026	28	27	0.00%	0.00%	20	35	21	32	*hypothetical protein*	Unknown	Unknown
RCAP_rcc01070	-0.75	0.0013	41	23	0.00%	0.00%	32	48	18	29	*hypothetical protein*	Unknown	Unknown
RCAP_rcc01071	-1.15	0	65	27	0.00%	0.00%	45	80	20	35	*hypothetical protein*	Unknown	Unknown
RCAP_rcc01072	-0.78	0.0009	300	169	0.00%	0.00%	213	373	142	196	*hutH*	Amino Acid Metabolism	Histidine metabolism
RCAP_rcc01073	-1.57	0	1772	560	0.02%	0.01%	1298	2140	444	676	*gvpA*	Motility	Gas Vesicle
RCAP_rcc01074	-1.7	0	17	4	0.00%	0.00%	10	22	3	6	*hypothetical protein*	Unknown	Unknown
RCAP_rcc01075	-1.35	0	776	294	0.01%	0.00%	610	893	242	346	*methyl-accepting chemotaxis protein*	Motility	Chemotaxis
RCAP_rcc01076	-0.9	0	261	138	0.00%	0.00%	210	300	124	152	*hypothetical protein*	Unknown	Unknown
RCAP_rcc01077	1.37	0.0001	85	253	0.00%	0.00%	66	137	178	327	*hypothetical protein*	Unknown	Unknown
RCAP_rcc01078	0.95	0	139	270	0.00%	0.00%	141	157	250	291	*SH3 domain-containing protein*	Unknown	Unknown
RCAP_rcc01079	0.09	0.6701	40	42	0.00%	0.00%	35	47	36	49	*hypothetical protein*	Unknown	Unknown
RCAP_rcc01080	-0.06	0.7514	60	56	0.00%	0.00%	51	68	49	64	*hypothetical protein*	Unknown	Unknown
RCAP_rcc01081	0.02	0.9273	1079	1092	0.02%	0.01%	925	1236	994	1190	*group 1 glycosyl transferase*	Cell Envelope Biosynthesis	Cell Wall Biosynthesis

RCAP_rcc01082	-0.39	0.0453	1151	870	0.02%	0.01%	907	1355	768	971	*group 1 glycosyl transferase*	Cell Envelope Biosynthesis	Cell Wall Biosynthesis
RCAP_rcc01083	-0.58	0.0015	956	633	0.01%	0.01%	771	1115	559	708	*lspL1*	Carbohydrate Metabolism	Amino sugar and nucleotide sugar metabolism
RCAP_rcc01084	-0.37	0	405	311	0.01%	0.00%	365	432	290	331	*hypothetical protein*	Unknown	Unknown
RCAP_rcc01085	-0.89	0.0002	1416	736	0.02%	0.01%	988	1781	619	853	*hypothetical protein*	Unknown	Unknown
RCAP_rcc01086	-0.34	0.0014	505	396	0.01%	0.01%	461	536	355	437	*family 2 glycosyl transferase*	Cell Envelope Biosynthesis	Cell Wall Biosynthesis
RCAP_rcc01087	-0.37	0.0204	180	139	0.00%	0.00%	150	206	126	151	*pip2*	Amino Acid Metabolism	Arginine and proline metabolism
RCAP_rcc01088	0.11	0.4541	274	296	0.00%	0.00%	242	302	258	334	*LuxR family autoinducer-binding transcriptional regulator*	Signal Transduction	Transcription Regulator
RCAP_rcc01089	-0.33	0.05	900	715	0.01%	0.01%	778	1021	611	819	*gsiB*	Sulfur Metabolism	Glutathione metabolism
RCAP_rcc01090	-0.65	0	288	182	0.00%	0.00%	241	324	170	195	*gsiD*	Sulfur Metabolism	Glutathione metabolism
RCAP_rcc01091	-0.62	0	219	142	0.00%	0.00%	193	237	132	153	*gsiC*	Sulfur Metabolism	Glutathione metabolism
RCAP_rcc01092	-0.79	0	212	122	0.00%	0.00%	172	243	112	131	*gsiA*	Sulfur Metabolism	Glutathione metabolism
RCAP_rcc01093	-0.71	0	259	157	0.00%	0.00%	214	293	143	171	*pepA1*	Metabolism of Other Amino Acids	Glutathione metabolism
RCAP_rcc01094	-0.52	0	103	72	0.00%	0.00%	92	110	65	78	*flavin-nucleotide-binding protein*	Unknown	Unknown
RCAP_rcc01095	0.26	0.5468	50	62	0.00%	0.00%	31	67	31	93	*GntR family transcriptional regulator*	Signal Transduction	Transcription Regulator
RCAP_rcc01096	0.56	0.0012	1207	1797	0.02%	0.02%	989	1458	1572	2021	*abgB*	Amino Acid Metabolism	Amino Acid Transport
RCAP_rcc01097	-0.09	0.7484	162	151	0.00%	0.00%	117	203	129	174	*hypothetical protein*	Unknown	Unknown
RCAP_rcc01099	-0.13	0.6432	152	139	0.00%	0.00%	112	190	114	164	*IS4 family transposase*	Replication, Recombination and Repair	Recombination
RCAP_rcc01100	0.48	0.0051	52	74	0.00%	0.00%	43	64	66	82	*XRE family transcriptional regulator*	Signal Transduction	Transcription Regulator
RCAP_rcc01101	-0.08	0.6571	143	136	0.00%	0.00%	121	164	124	147	*HipA domain-containing protein*	Unknown	Unknown
RCAP_rcc01102	0.1	0.5178	40	43	0.00%	0.00%	36	45	38	47	*hypothetical protein*	Unknown	Unknown
RCAP_rcc01103	0.03	0.9037	15	15	0.00%	0.00%	12	17	13	18	*hypothetical protein*	Unknown	Unknown
RCAP_rcc01104	0.48	0.106	7	9	0.00%	0.00%	5	8	7	12	*hypothetical protein*	Unknown	Unknown
RCAP_rcc01105	0.35	0.18	6	7	0.00%	0.00%	4	7	6	9	*hypothetical protein*	Unknown	Unknown
RCAP_rcc01107	-0.05	0.7078	663	641	0.01%	0.01%	583	733	594	688	*hypothetical protein*	Unknown	Unknown
RCAP_rcc01108	-0.33	0.5066	85	63	0.00%	0.00%	28	135	23	104	*pyrroline-5-carboxylate reductase*	Amino Acid Metabolism	Arginine and proline metabolism
RCAP_rcc01109	-0.02	0.913	824	813	0.01%	0.01%	707	922	713	912	*rsbQ*	Signal Transduction	Transcription Regulator
RCAP_rcc01110	-0.43	0.2668	510	362	0.01%	0.00%	304	681	226	498	*diguanylate cyclase/phosphodiesterase*	Signal Transduction	Kinase/Phosphorelay
RCAP_rcc01111	0.03	0.9424	296	305	0.00%	0.00%	212	429	169	440	*hypothetical protein*	Unknown	Unknown
RCAP_rcc01112	-0.56	0.1015	361	234	0.01%	0.00%	284	455	120	348	*hypothetical protein*	Unknown	Unknown
RCAP_rcc01113	-0.47	0.01	652	466	0.01%	0.01%	531	751	404	529	*MiaB family RNA modification enzyme*	Translation, ribosomal structure and biogenesis	Unknown
RCAP_rcc01114	0.24	0.3835	1669	1991	0.02%	0.03%	1142	2228	1700	2282	*OmpA/MotB domain-containing protein*	Unknown	Unknown
RCAP_rcc01115	0.07	0.8419	1026	1085	0.01%	0.01%	674	1406	876	1295	*lipoprotein*	Predicted Function	Unknown
RCAP_rcc01116	-0.22	0.0908	3122	2684	0.04%	0.04%	2798	3417	2394	2974	*PhoH family protein*	Signal Transduction	Kinase/Phosphorelay
RCAP_rcc01117	-0.27	0.1831	1552	1278	0.02%	0.02%	1240	1817	1082	1474	*hypothetical protein*	Unknown	Unknown
RCAP_rcc01118	-0.18	0.144	2906	2554	0.04%	0.03%	2572	3227	2309	2798	*corC*	Metal, Ion, Cofactor Transport	Cobalt Transport
RCAP_rcc01119	-0.08	0.6424	1051	989	0.01%	0.01%	874	1205	884	1094	*lnt*	Cell Envelope Biosynthesis	Cell Wall Biosynthesis
RCAP_rcc01120	0.63	0.0001	7937	12451	0.11%	0.17%	7158	8905	10322	14580	*metK*	Amino Acid Metabolism	Cysteine and methionine metabolism
RCAP_rcc01121	-0.5	0.0038	374	261	0.01%	0.00%	312	426	224	298	*trmB*	Unknown	Unknown
RCAP_rcc01122	-0.19	0.4047	665	579	0.01%	0.01%	496	813	501	657	*aroA*	Amino Acid Metabolism	Phenylalanine, tyrosine and tryptophan biosynthesis
RCAP_rcc01123	-0.55	0.0007	179	121	0.00%	0.00%	144	204	109	132	*cmk*	Nucleotide Metabolism	Pyrimidine metabolism
RCAP_rcc01124	-0.4	0	1179	891	0.02%	0.01%	1057	1282	839	943	*nfnB*	Energy Metabolism	Unknown
RCAP_rcc01125	-0.4	0.1174	46509	34547	0.65%	0.47%	35175	57402	26631	42462	*rpsA*	Translation, ribosomal structure and biogenesis	Unknown
RCAP_rcc01126	0.29	0.1009	2381	2925	0.03%	0.04%	2042	2831	2512	3337	*ihfB*	Transcription	Unknown
RCAP_rcc01127	0.4	0.0026	2340	3097	0.03%	0.04%	2095	2698	2774	3420	*hypothetical protein*	Unknown	Unknown
RCAP_rcc01128	0.26	0.4562	400	487	0.01%	0.01%	270	512	372	602	*trpF*	Amino Acid Metabolism	Phenylalanine, tyrosine and tryptophan biosynthesis
RCAP_rcc01129	-0.4	0.0059	4147	3123	0.06%	0.04%	3586	4643	2727	3518	*trpB1*	Amino Acid Metabolism	Phenylalanine, tyrosine and tryptophan biosynthesis
RCAP_rcc01130	-0.79	0	396	226	0.01%	0.00%	332	436	195	257	*LuxR family transcriptional regulator*	Signal Transduction	Transcription Regulator
RCAP_rcc01131	0.67	0.0038	221	361	0.00%	0.00%	177	265	261	460	*MarR family transcriptional regulator*	Signal Transduction	Transcription Regulator
RCAP_rcc01132	0.74	0.0351	225	402	0.00%	0.01%	137	307	250	554	*galM*	Carbohydrate Metabolism	Glycolysis / Gluconeogenesis
RCAP_rcc01133	0.63	0.0005	41	65	0.00%	0.00%	34	50	57	73	*znuA*	Metal, Ion, Cofactor Transport	Zinc Transport
RCAP_rcc01134	0.74	0.0001	45	76	0.00%	0.00%	39	52	60	91	*Fur family transcriptional regulator*	Signal Transduction	Transcription Regulator
RCAP_rcc01135	0.58	0.0213	60	91	0.00%	0.00%	46	75	70	113	*znuC*	Metal, Ion, Cofactor Transport	Zinc Transport
RCAP_rcc01136	0.42	0.2412	120	166	0.00%	0.00%	79	158	119	213	*znuB*	Metal, Ion, Cofactor Transport	Zinc Transport
RCAP_rcc01137	0.66	0.014	499	818	0.01%	0.01%	356	645	655	981	*pfkB*	Carbohydrate Metabolism	Glycolysis / Gluconeogenesis
RCAP_rcc01138	-1.38	0	1514	558	0.02%	0.01%	1188	1774	445	670	*hypothetical protein*	Unknown	Unknown
RCAP_rcc01139	-1.71	0	5246	1545	0.07%	0.02%	4273	5898	1305	1784	*hypothetical protein*	Unknown	Unknown
RCAP_rcc01140	-0.99	0	659	327	0.01%	0.00%	572	714	287	368	*hypothetical protein*	Unknown	Unknown
RCAP_rcc01141	-0.11	0.4745	4812	4467	0.07%	0.06%	4309	5346	4006	4927	*gltX1*	Translation, ribosomal structure and biogenesis	Aminoacyl-tRNA biosynthesis
RCAP_rcc01142	-0.16	0.4632	2367	2103	0.03%	0.03%	1758	2904	1911	2296	*gcvT1*	Energy Metabolism	Nitrogen metabolism
RCAP_rcc01143	-0.53	0.0388	3351	2273	0.05%	0.03%	2338	4268	1914	2632	*gcvH*	Carbohydrate Metabolism	Glyoxylate and dicarboxylate metabolism
RCAP_rcc01144	-0.57	0.0029	14734	9748	0.20%	0.13%	11563	17421	8903	10594	*gcvP*	Amino Acid Metabolism	Glycine, serine and threonine metabolism
RCAP_rcc01145	-0.37	0.0216	2623	2014	0.04%	0.03%	2225	3005	1753	2275	*fabH1*	Lipid Metabolism	Biotin metabolism
RCAP_rcc01146	0.03	0.8119	537	549	0.01%	0.01%	487	577	497	600	*gyaR2*	Energy Metabolism	Methane metabolism
RCAP_rcc01147	0.21	0.0075	355	412	0.01%	0.01%	340	381	384	440	*csaA*	Unknown	Unknown
RCAP_rcc01148	0.41	0.0984	447	605	0.01%	0.01%	331	554	472	737	*proC*	Amino Acid Metabolism	Arginine and proline metabolism
RCAP_rcc01149	-0.52	0.0042	2339	1607	0.03%	0.02%	1846	2754	1376	1837	*hypothetical protein*	Unknown	Unknown
RCAP_rcc01150	0.15	0.1921	677	754	0.01%	0.01%	607	753	701	807	*aroC*	Amino Acid Metabolism	Phenylalanine, tyrosine and tryptophan biosynthesis
RCAP_rcc01151	-0.01	0.9606	376	375	0.01%	0.01%	339	409	341	409	*hypothetical protein*	Unknown	Unknown
RCAP_rcc01152	0	0.9846	426	426	0.01%	0.01%	383	470	385	467	*hisE*	Amino Acid Metabolism	Histidine metabolism
RCAP_rcc01153	0.34	0.0012	708	900	0.01%	0.01%	655	792	835	965	*hisF*	Amino Acid Metabolism	Histidine metabolism
RCAP_rcc01154	0.25	0.202	260	313	0.00%	0.00%	206	323	287	339	*hisA*	Amino Acid Metabolism	Histidine metabolism
RCAP_rcc01155	-0.81	0	973	543	0.01%	0.01%	726	1210	483	603	*hypothetical protein*	Unknown	Unknown
RCAP_rcc01156	1.67	0	2938	10186	0.05%	0.14%	2490	4053	7935	12437	*UspA domain-containing protein*	Stress Response	Unknown
RCAP_rcc01157	-1.55	0	69618	22672	0.94%	0.31%	53726	80481	18170	27173	*ccoN*	Energy Metabolism	Aerobic/Anaerobic Respiration
RCAP_rcc01158	-1.89	0	20124	5171	0.27%	0.07%	16551	22174	4257	6086	*ccoO*	Energy Metabolism	Aerobic/Anaerobic Respiration
RCAP_rcc01159	-2.07	0	5899	1307	0.08%	0.02%	4511	6813	1029	1585	*ccoQ*	Energy Metabolism	Aerobic/Anaerobic Respiration
RCAP_rcc01160	-1.83	0	20645	5665	0.28%	0.08%	18354	21401	4872	6458	*ccoP*	Energy Metabolism	Aerobic/Anaerobic Respiration

RCAP_rcc01161	-1.59	0	6551	2101	0.09%	0.03%	5240	7402	1788	2413	*ccoG*	Energy Metabolism	Aerobic/Anaerobic Respiration
RCAP_rcc01162	-0.87	0	905	492	0.01%	0.01%	819	945	456	528	*ccoH*	Energy Metabolism	Aerobic/Anaerobic Respiration
RCAP_rcc01163	-1.09	0	3130	1447	0.04%	0.02%	2589	3492	1344	1551	*ccoI*	Energy Metabolism	Aerobic/Anaerobic Respiration
RCAP_rcc01164	-1.04	0	465	220	0.01%	0.00%	356	553	181	259	*ccoS*	Energy Metabolism	Aerobic/Anaerobic Respiration
RCAP_rcc01165	0.14	0.1889	4119	4549	0.06%	0.06%	3738	4527	4229	4869	*dacC1*	Glycan Biosynthesis and Metabolism	Peptidoglycan biosynthesis
RCAP_rcc01166	0.22	0.0944	277	322	0.00%	0.00%	244	308	292	352	*HAD superfamily hydrolase*	Unknown	Unknown
RCAP_rcc01167	-0.31	0.0248	889	714	0.01%	0.01%	753	1003	642	787	*clpS*	Post-translational Modification, Assembly and Chaperones	Unknown
RCAP_rcc01168	-0.51	0.0043	670	465	0.01%	0.01%	535	768	393	538	*methyltransferase small domain-containing protein*	Translation, ribosomal structure and biogenesis	Unknown
RCAP_rcc01169	0.21	0.0745	757	878	0.01%	0.01%	678	849	806	949	*lipoprotein*	Predicted Function	Unknown
RCAP_rcc01170	-0.18	0.5896	8	7	0.00%	0.00%	6	11	6	9	*hypothetical protein*	Unknown	Unknown
RCAP_rcc01171	-1.05	0	3092	1447	0.04%	0.02%	2379	3638	1173	1721	*ATP-dependent RNA helicase DbpA*	Replication, Recombination and Repair	Unknown
RCAP_rcc01172	1.62	0	3477	10911	0.05%	0.15%	3470	4252	9687	12135	*hemE*	Metabolism of Cofactors, Coenzymes and Vitamins	Heme Biosynthesis
RCAP_rcc01173	1.45	0	2720	7518	0.04%	0.10%	2611	3235	6972	8065	*hemC*	Metabolism of Cofactors, Coenzymes and Vitamins	Heme Biosynthesis
RCAP_rcc01174	1.09	0	308	660	0.00%	0.01%	294	334	577	743	*D-2-hydroxyglutarate dehydrogenase*	Energy Metabolism	Unknown
RCAP_rcc01175	-0.39	0	280	212	0.00%	0.00%	263	290	204	221	*major facilitator superfamily protein*	Metal and Ion Transport	Unknown
RCAP_rcc01176	-0.27	0.3091	154	126	0.00%	0.00%	110	191	101	151	*GDSL-like lipase/acylhydrolase*	Xenobiotics Biodegradation and Metabolism	Bisphenol A degradation
RCAP_rcc01177	1.64	0	249	812	0.00%	0.01%	252	330	647	978	*ABC transporter ATP-binding protein*	Secondary metabolites biosynthesis, transport, and catabolism	Unknown
RCAP_rcc01178	0.77	0.0068	562	1000	0.01%	0.01%	420	737	691	1310	*hypothetical protein*	Unknown	Unknown
RCAP_rcc01179	-0.73	0.0001	1389	827	0.02%	0.01%	1092	1643	723	931	*transglycosylase, Slt family*	Cell Envelope Biosynthesis	Cell Wall Biosynthesis
RCAP_rcc01180	0.11	0.6683	324	350	0.00%	0.00%	251	391	280	419	*copA2*	Metal, Ion, Cofactor Transport	Copper Transport
RCAP_rcc01181	0.91	0	292	559	0.00%	0.01%	264	362	465	654	*hypothetical protein*	Unknown	Unknown
RCAP_rcc01182	0.3	0.0004	477	590	0.01%	0.01%	447	527	557	623	*hisH*	Nucleotide Metabolism	Purine metabolism
RCAP_rcc01183	0.13	0.5327	1016	1114	0.01%	0.02%	795	1265	1030	1197	*hisB*	Amino Acid Metabolism	Histidine metabolism
RCAP_rcc01184	0.93	0.0003	491	984	0.01%	0.01%	415	627	707	1261	*CsbD family protein*	Unknown	Unknown
RCAP_rcc01185	1.47	0	57	180	0.00%	0.00%	47	74	83	276	*methyl-accepting chemotaxis sensory transducer*	Motility	Chemotaxis
RCAP_rcc01186	0.99	0	56	112	0.00%	0.00%	51	71	97	126	*hypothetical protein*	Unknown	Unknown
RCAP_rcc01187	0.75	0.0279	4	6	0.00%	0.00%	2	5	4	8	*phnC*	Metal and Ion Transport	Unknown
RCAP_rcc01188	0.35	0.1012	66	85	0.00%	0.00%	53	81	74	95	*phnD*	Metal and Ion Transport	Unknown
RCAP_rcc01189	0.88	0.0013	7	13	0.00%	0.00%	5	9	9	16	*phnE1*	Metal and Ion Transport	Unknown
RCAP_rcc01190	1.2	0	24	58	0.00%	0.00%	19	33	47	69	*phnE2*	Metal and Ion Transport	Unknown
RCAP_rcc01191	1.23	0	38	97	0.00%	0.00%	23	57	70	124	*transferase hexapeptide repeat family protein*	Unknown	Unknown
RCAP_rcc01192	2.2	NA	17	128	0.00%	0.00%	10	27	34	221	*phnF*	Signal Transduction	Transcription Regulator
RCAP_rcc01193	0.79	0.1178	1	3	0.00%	0.00%	1	2	1	4	*phnG*	Metal and Ion Transport	Unknown
RCAP_rcc01194	0.62	0.0799	4	6	0.00%	0.00%	2	5	4	8	*phnH*	Metal and Ion Transport	Unknown
RCAP_rcc01195	0.6	0	114	175	0.00%	0.00%	103	132	154	197	*phnI*	Metal and Ion Transport	Unknown
RCAP_rcc01197	0.49	0.0027	17	24	0.00%	0.00%	16	20	21	27	*phnJ*	Metal and Ion Transport	Unknown
RCAP_rcc01198	0.1	0.7201	11	12	0.00%	0.00%	7	14	10	13	*phnK*	Metal and Ion Transport	Unknown
RCAP_rcc01199	0.34	0.4441	4	5	0.00%	0.00%	2	5	3	6	*phnL*	Metal and Ion Transport	Unknown
RCAP_rcc01200	0.58	NA	1	1	0.00%	0.00%	0	1	0	3	*phnN*	Carbohydrate Metabolism	Pentose phosphate pathway
RCAP_rcc01201	1.06	0.0004	7	15	0.00%	0.00%	5	9	9	20	*hypothetical protein*	Unknown	Unknown
RCAP_rcc01202	0.29	0.1012	19	23	0.00%	0.00%	17	22	20	26	*phnM*	Metal and Ion Transport	Unknown
RCAP_rcc01203	0.77	0	5471	9472	0.08%	0.13%	4863	6500	8427	10516	*pyc*	Carbohydrate Metabolism	TCA Cycle
RCAP_rcc01204	-0.08	0.426	848	800	0.01%	0.01%	765	909	743	858	*peptidoglycan binding domain-containing protein*	Unknown	Unknown
RCAP_rcc01205	-0.3	0.2449	1727	1384	0.02%	0.02%	1203	2231	1173	1595	*glyQ*	Translation, ribosomal structure and biogenesis	Aminoacyl-tRNA biosynthesis
RCAP_rcc01206	-0.11	0.7296	37	34	0.00%	0.00%	28	47	26	42	*hypothetical protein*	Unknown	Unknown
RCAP_rcc01207	-0.37	0.0739	5537	4250	0.08%	0.06%	4505	6485	3628	4871	*glyS*	Translation, ribosomal structure and biogenesis	Aminoacyl-tRNA biosynthesis
RCAP_rcc01208	0.2	0.0156	1618	1863	0.02%	0.03%	1526	1722	1723	2003	*ppdK*	Energy Metabolism	Carbon fixation in photosynthetic organisms
RCAP_rcc01209	-0.89	0	1597	854	0.02%	0.01%	1412	1689	765	943	*cell wall hydrolase, SleB*	Cell Envelope Biosynthesis	Cell Wall Biosynthesis
RCAP_rcc01210	0.19	0.1647	521	593	0.01%	0.01%	461	582	536	650	*folB*	Metabolism of Cofactors, Coenzymes and Vitamins	Folate biosynthesis
RCAP_rcc01211	-0.18	0.5492	321	281	0.00%	0.00%	218	409	216	347	*folP*	Metabolism of Cofactors, Coenzymes and Vitamins	Folate biosynthesis
RCAP_rcc01212	-0.09	0.2304	1708	1604	0.02%	0.02%	1611	1795	1509	1698	*glmM*	Carbohydrate Metabolism	Amino sugar and nucleotide sugar metabolism
RCAP_rcc01213	0.34	0.006	373	474	0.01%	0.01%	335	404	417	531	*hypothetical protein*	Unknown	Unknown
RCAP_rcc01214	0.44	0.0244	5496	7538	0.08%	0.10%	4299	6904	6716	8360	*ilvC*	Metabolism of Cofactors, Coenzymes and Vitamins	Pantothenate and CoA biosynthesis
RCAP_rcc01215	0.07	0.7389	68	72	0.00%	0.00%	55	80	63	80	*AsnC/Lrp family transcriptional regulator*	Signal Transduction	Transcription Regulator
RCAP_rcc01216	-0.25	0.1108	36	30	0.00%	0.00%	31	40	27	34	*AsnC/Lrp family transcriptional regulator*	Signal Transduction	Transcription Regulator
RCAP_rcc01217	2	0	30	126	0.00%	0.00%	28	39	98	155	*ureD*	Energy Metabolism	Urea Cycle
RCAP_rcc01218	1.82	0	15	54	0.00%	0.00%	15	21	47	62	*ureA*	Energy Metabolism	Urea Cycle
RCAP_rcc01219	1.45	0	17	47	0.00%	0.00%	16	21	40	54	*ureB*	Energy Metabolism	Urea Cycle
RCAP_rcc01220	0.96	0	285	558	0.00%	0.01%	267	330	498	618	*ureC*	Energy Metabolism	Urea Cycle
RCAP_rcc01221	0.16	0.6305	51	57	0.00%	0.00%	35	66	41	73	*hypothetical protein*	Unknown	Unknown
RCAP_rcc01222	0.83	0.0014	47	87	0.00%	0.00%	36	61	71	103	*ureE*	Energy Metabolism	Urea Cycle
RCAP_rcc01223	0.46	0	62	86	0.00%	0.00%	58	69	78	93	*ureF*	Energy Metabolism	Urea Cycle
RCAP_rcc01224	0.22	0.2567	37	43	0.00%	0.00%	30	43	37	48	*ureG*	Energy Metabolism	Urea Cycle
RCAP_rcc01225	0.26	0.634	1	1	0.00%	0.00%	0	2	1	2	*hypothetical protein*	Unknown	Unknown
RCAP_rcc01226	0.48	0.0227	44	62	0.00%	0.00%	33	56	52	71	*urtB*	Energy Metabolism	Urea Cycle
RCAP_rcc01227	0.88	0	14	26	0.00%	0.00%	12	17	21	30	*urtA*	Energy Metabolism	Urea Cycle
RCAP_rcc01228	0.52	0.0002	36	51	0.00%	0.00%	33	42	46	56	*urtC*	Energy Metabolism	Urea Cycle
RCAP_rcc01229	0.36	0.1091	11	14	0.00%	0.00%	9	14	12	16	*urtD*	Energy Metabolism	Urea Cycle
RCAP_rcc01230	-0.6	0.0016	218	142	0.00%	0.00%	178	256	122	161	*urtE*	Energy Metabolism	Urea Cycle
RCAP_rcc01231	1	0.001	648	1391	0.01%	0.02%	434	930	1042	1741	*UbiH/UbiF/VisC/COQ6 family ubiquinone biosynthesis hydroxylase*	Energy Metabolism	Biosynthesis of Ubiquinone
RCAP_rcc01232	2.16	0	139	653	0.00%	0.01%	144	200	570	737	*hypothetical protein*	Unknown	Unknown
RCAP_rcc01233	1.04	0	314	656	0.00%	0.01%	301	371	572	740	*hypothetical protein*	Unknown	Unknown
RCAP_rcc01234	-0.91	0	5040	2631	0.07%	0.04%	4296	5596	2102	3161	*hypothetical protein*	Unknown	Unknown
RCAP_rcc01235	0.19	0.3032	150	171	0.00%	0.00%	131	171	143	198	*pyrimidine 5'-nucleotidase*	Metabolism of Cofactors, Coenzymes and Vitamins	Vitamin B6 metabolism
RCAP_rcc01236	-0.77	0	884	507	0.01%	0.01%	707	1006	405	609	*GntR family transcriptional regulator*	Signal Transduction	Transcription Regulator
RCAP_rcc01237	-1.91	0	785	198	0.01%	0.00%	579	926	172	224	*family 2 glycosyl transferase*	Cell Envelope Biosynthesis	Cell Wall Biosynthesis
RCAP_rcc01238	-0.22	0.2068	2222	1894	0.03%	0.03%	1858	2591	1671	2116	*carA*	Amino Acid Metabolism	Alanine, aspartate and glutamate metabolism

RCAP_rcc01239	-0.11	0.3881	166	153	0.00%	0.00%	146	183	140	165	*glyoxalase/bleomycin resistance protein/dioxygenase*	Carbohydrate Metabolism	Pyruvate metabolism
RCAP_rcc01240	0.19	0.2783	13882	15834	0.20%	0.22%	11326	16713	14251	17417	*cycA1*	Energy Metabolism	Aerobic/Anaerobic Respiration
RCAP_rcc01241	1.1	0	178	404	0.00%	0.01%	123	239	312	495	*lipB*	Metabolism of Cofactors, Coenzymes and Vitamins	Unknown
RCAP_rcc01242	-0.54	0	660	451	0.01%	0.01%	559	738	421	480	*hemolysin-type calcium-binding repeat family protein*	Trafficking and Secretion	Secretion
RCAP_rcc01243	-0.87	0	12523	6672	0.17%	0.09%	9842	14593	5303	8042	*potA1*	Amino Acid Metabolism	Amino Acid Transport
RCAP_rcc01244	-1.74	0	48039	13658	0.65%	0.19%	40768	52226	10720	16596	*potD1*	Unknown	Unknown
RCAP_rcc01245	-1.44	0	7189	2557	0.10%	0.03%	6375	7595	2019	3096	*potB1*	Amino Acid Metabolism	Amino Acid Transport
RCAP_rcc01246	-1.38	0	7729	2869	0.11%	0.04%	6963	8109	2284	3453	*potI1*	Amino Acid Metabolism	Amino Acid Transport
RCAP_rcc01247	-0.73	0	335	199	0.00%	0.00%	290	362	177	222	*membrane transport family protein*	Unknown	Unknown
RCAP_rcc01248	-0.14	0.254	320	288	0.00%	0.00%	288	346	260	317	*metallo-beta-lactamase*	Unknown	Unknown
RCAP_rcc01249	-0.26	0.0119	482	403	0.01%	0.01%	430	526	375	431	*TatD-related deoxyribonuclease*	Trafficking and Secretion	Secretion
RCAP_rcc01250	-0.26	0.3228	236	195	0.00%	0.00%	174	288	159	231	*holB*	Replication, Recombination and Repair	Replication
RCAP_rcc01251	-0.21	0.2907	524	449	0.01%	0.01%	412	623	403	496	*tmk*	Nucleotide Metabolism	Pyrimidine metabolism
RCAP_rcc01252	-0.22	0.0259	2158	1848	0.03%	0.03%	1964	2334	1711	1985	*dacC2*	Glycan Biosynthesis and Metabolism	Peptidoglycan biosynthesis
RCAP_rcc01253	0.63	0.0026	454	715	0.01%	0.01%	364	557	581	850	*sporulation domain-containing protein*	Unknown	Unknown
RCAP_rcc01254	0.13	0.6017	513	563	0.01%	0.01%	382	643	492	635	*phage integrase*	Replication, Recombination and Repair	Phage Interaction
RCAP_rcc01255	-0.31	0.1368	52	41	0.00%	0.00%	42	59	33	49	*hypothetical protein*	Unknown	Unknown
RCAP_rcc01256	-0.21	0.2396	123	106	0.00%	0.00%	109	137	87	125	*hypothetical protein*	Unknown	Unknown
RCAP_rcc01257	-0.68	0.0048	149	91	0.00%	0.00%	116	178	65	116	*hypothetical protein*	Unknown	Unknown
RCAP_rcc01258	0.29	0.0488	41	50	0.00%	0.00%	37	46	44	56	*hypothetical protein*	Unknown	Unknown
RCAP_rcc01259	0.17	0.1601	43	48	0.00%	0.00%	39	49	46	51	*hypothetical protein*	Unknown	Unknown
RCAP_rcc01260	-0.46	0.0243	3420	2455	0.05%	0.03%	2669	4063	2052	2857	*mod*	Replication, Recombination and Repair	Unknown
RCAP_rcc01261	-0.49	0.0052	4242	3000	0.06%	0.04%	3492	4875	2571	3429	*res*	Defense Mechanisms	Unknown
RCAP_rcc01262	-0.29	0.1465	448	365	0.01%	0.00%	359	526	310	419	*recombinase*	Replication, Recombination and Repair	Recombination
RCAP_rcc01263	0.15	0.612	1312	1471	0.02%	0.02%	900	1726	1206	1737	*hypothetical protein*	Unknown	Unknown
RCAP_rcc01264	-0.43	0.1601	1501	1084	0.02%	0.01%	1009	1936	843	1325	*reverse transcriptase*	Unknown	Unknown
RCAP_rcc01267	0.14	0.5076	32	35	0.00%	0.00%	25	38	32	39	*hypothetical protein*	Unknown	Unknown
RCAP_rcc01268	2.03	0	36	161	0.00%	0.00%	43	61	120	201	*hypothetical protein*	Unknown	Unknown
RCAP_rcc01269	0.36	0.1038	136	176	0.00%	0.00%	100	167	145	208	*hypothetical protein*	Unknown	Unknown
RCAP_rcc01270	-0.59	0.0019	66	44	0.00%	0.00%	53	76	36	52	*XRE family transcriptional regulator*	Signal Transduction	Transcription Regulator
RCAP_rcc01271	0.25	0.0239	1297	1550	0.02%	0.02%	1160	1443	1434	1665	*hypothetical protein*	Unknown	Unknown
RCAP_rcc01272	-0.03	0.8776	1424	1392	0.02%	0.02%	1197	1623	1221	1563	*hypothetical protein*	Unknown	Unknown
RCAP_rcc01273	0.97	0	427	853	0.01%	0.01%	385	537	746	960	*hypothetical protein*	Unknown	Unknown
RCAP_rcc01274	0.48	0.0288	1604	2281	0.02%	0.03%	1339	2009	1885	2676	*RAMP family CRISPR-associated protein*	Replication, Recombination and Repair	Unknown
RCAP_rcc01275	0.68	0	56	90	0.00%	0.00%	47	65	78	102	*Cas2 family CRISPR-associated protein*	Unknown	Unknown
RCAP_rcc01276	0.12	0.6674	664	723	0.01%	0.01%	469	836	614	832	*Cas1 family CRISPR-associated protein*	Replication, Recombination and Repair	Unknown
RCAP_rcc01277	-0.12	0.5092	1107	1014	0.02%	0.01%	897	1282	884	1144	*RNA-directed DNA polymerase*	Replication, Recombination and Repair	Replication
RCAP_rcc01278	-0.32	0.0504	305	242	0.00%	0.00%	267	326	195	290	*hypothetical protein*	Unknown	Unknown
RCAP_rcc01279	-0.22	0.2786	127	108	0.00%	0.00%	103	146	88	128	*hypothetical protein*	Unknown	Unknown
RCAP_rcc01280	-0.17	0.2306	90	80	0.00%	0.00%	76	103	74	86	*exonuclease*	Unknown	Unknown
RCAP_rcc01281	-0.01	0.9424	50	50	0.00%	0.00%	43	57	43	57	*hypothetical protein*	Unknown	Unknown
RCAP_rcc01282	-0.43	0	115	85	0.00%	0.00%	105	123	78	92	*relaxase/mobilization nuclease domain-containing protein*	Unknown	Unknown
RCAP_rcc01283	-0.57	0.0002	36	24	0.00%	0.00%	31	40	21	27	*hypothetical protein*	Unknown	Unknown
RCAP_rcc01284	-0.62	0.0003	235	151	0.00%	0.00%	200	265	124	177	*hypothetical protein*	Unknown	Unknown
RCAP_rcc01285	0.24	0.0108	209	247	0.00%	0.00%	196	221	225	269	*GntR family transcriptional regulator*	Signal Transduction	Transcription Regulator
RCAP_rcc01286	0.38	0.3492	3055	4150	0.04%	0.06%	1412	4657	1989	6310	*mcrB*	Defense Mechanisms	Unknown
RCAP_rcc01287	0.15	NA	538	605	0.01%	0.01%	329	719	318	892	*mcrC*	Defense Mechanisms	Unknown
RCAP_rcc01288	1.39	0.0001	269	820	0.00%	0.01%	188	369	493	1146	*hypothetical protein*	Unknown	Unknown
RCAP_rcc01289	1.2	0.0004	135	346	0.00%	0.00%	91	179	177	515	*hypothetical protein*	Unknown	Unknown
RCAP_rcc01290	1.04	0.001	212	472	0.00%	0.01%	132	292	269	675	*UvrD/REP helicase*	Replication, Recombination and Repair	Unknown
RCAP_rcc01291	0.46	0.0301	1048	1465	0.01%	0.02%	802	1318	1203	1727	*kinetochore Spc7 domain-containing protein*	Unknown	Unknown
RCAP_rcc01292	-0.02	0.9349	486	478	0.01%	0.01%	363	598	386	571	*OmpA/MotB domain-containing protein*	Unknown	Unknown
RCAP_rcc01293	-0.18	0.4465	494	433	0.01%	0.01%	377	601	353	514	*hypothetical protein*	Unknown	Unknown
RCAP_rcc01294	0.29	0.0083	693	846	0.01%	0.01%	631	762	769	923	*SNF2 family helicase*	Replication, Recombination and Repair	Unknown
RCAP_rcc01298	0.48	0.0016	24	34	0.00%	0.00%	21	28	31	38	*SNF2 family helicase*	Unknown	Unknown
RCAP_rcc01299	1.02	0.004	157	353	0.00%	0.00%	98	219	208	499	*hypothetical protein*	Unknown	Unknown
RCAP_rcc01300	0.61	0.0836	738	1194	0.01%	0.02%	397	1076	637	1751	*Cas3 family CRISPR-associated helicase*	Replication, Recombination and Repair	Unknown
RCAP_rcc01301	0.73	0.0218	1087	1911	0.02%	0.03%	695	1544	1247	2575	*Cas5 family CRISPR-associated protein*	Replication, Recombination and Repair	Unknown
RCAP_rcc01302	0.39	NA	1336	1808	0.02%	0.02%	759	1899	1014	2602	*Csd1 family CRISPR-associated protein*	Replication, Recombination and Repair	Unknown
RCAP_rcc01303	0.21	NA	1527	1810	0.02%	0.02%	743	2261	876	2744	*Cas2 family CRISPR-associated protein*	Replication, Recombination and Repair	Unknown
RCAP_rcc01304	0.4	0.1861	199	268	0.00%	0.00%	131	260	172	363	*Cas4 family CRISPR-associated protein*	Replication, Recombination and Repair	Unknown
RCAP_rcc01305	0.77	0.0016	60	106	0.00%	0.00%	46	76	76	136	*Cas1 family CRISPR-associated protein*	Replication, Recombination and Repair	Unknown
RCAP_rcc01306	0.03	0.9339	177	182	0.00%	0.00%	100	248	112	252	*Cas2 family CRISPR-associated protein*	Replication, Recombination and Repair	Unknown
RCAP_rcc01307	0.62	0.0204	504	801	0.01%	0.01%	367	637	541	1061	*hypothetical protein*	Unknown	Unknown
RCAP_rcc01309	0.21	0.5635	126	148	0.00%	0.00%	76	175	91	205	*IS66 family transposase*	Replication, Recombination and Repair	Recombination
RCAP_rcc01310	-0.26	0.3976	169	138	0.00%	0.00%	118	222	102	174	*hypothetical protein*	Unknown	Unknown
RCAP_rcc01311	-0.49	0.0399	139	97	0.00%	0.00%	102	171	80	114	*hypothetical protein*	Unknown	Unknown
RCAP_rcc01312	-0.41	0.1469	973	716	0.01%	0.01%	662	1264	552	879	*hypothetical protein*	Unknown	Unknown
RCAP_rcc01313	0.96	0	452	915	0.01%	0.01%	357	615	744	1086	*hypothetical protein*	Unknown	Unknown
RCAP_rcc01314	-0.12	0.6051	3864	3545	0.05%	0.05%	3012	4683	3008	4082	*hypothetical protein*	Unknown	Unknown
RCAP_rcc01315	-0.58	0.0065	3712	2435	0.05%	0.03%	2814	4509	2053	2818	*hypothetical protein*	Unknown	Unknown
RCAP_rcc01316	-0.68	0.0009	1226	752	0.02%	0.01%	950	1443	633	872	*hypothetical protein*	Unknown	Unknown
RCAP_rcc01317	0.12	0.6787	108	118	0.00%	0.00%	89	124	81	154	*pirin domain-containing protein*	Unknown	Unknown
RCAP_rcc01318	0.59	0.0003	455	693	0.01%	0.01%	391	540	605	781	*outer membrane autotransporter barrel domain-containing protein*	Unknown	Unknown
RCAP_rcc01319	1.88	0	39	159	0.00%	0.00%	34	50	101	216	*hypothetical protein*	Unknown	Unknown
RCAP_rcc01320	-0.11	0.4641	398	370	0.01%	0.01%	337	453	336	404	*hypothetical protein*	Unknown	Unknown
RCAP_rcc01321	-0.67	0.0001	363	226	0.01%	0.00%	303	411	194	259	*GNAT family acetyltransferase*	Cell Division	Chromosome Partitioning

RCAP_rcc01323	-0.15	0.4988	340	306	0.00%	0.00%	271	408	262	351	*IS66 family transposase*	Replication, Recombination and Repair	Recombination
RCAP_rcc01324	-0.18	0.3608	1217	1069	0.02%	0.01%	968	1438	931	1208	*hsdR2*	Defense Mechanisms	Unknown
RCAP_rcc01325	-0.37	0.0031	566	436	0.01%	0.01%	496	618	392	479	*hsdM2*	Defense Mechanisms	Unknown
RCAP_rcc01326	-0.7	0.001	1464	884	0.02%	0.01%	1119	1756	731	1037	*hsdS2*	Defense Mechanisms	Unknown
RCAP_rcc01327	-0.7	0.0005	973	586	0.01%	0.01%	768	1141	483	690	*hypothetical protein*	Unknown	Unknown
RCAP_rcc01328	-0.11	0.6877	1632	1500	0.02%	0.02%	1186	2070	1200	1800	*hypothetical protein*	Unknown	Unknown
RCAP_rcc01329	-0.56	0.0259	1198	795	0.02%	0.01%	864	1493	635	954	*hypothetical protein*	Unknown	Unknown
RCAP_rcc01330	-0.49	0.084	28	19	0.00%	0.00%	20	35	16	23	*hypothetical protein*	Unknown	Unknown
RCAP_rcc01333	-0.18	0.4933	12	10	0.00%	0.00%	9	15	9	12	*IS66 family transposase*	Replication, Recombination and Repair	Recombination
RCAP_rcc01334	-0.32	0.0525	29	24	0.00%	0.00%	25	32	20	27	*hypothetical protein*	Unknown	Unknown
RCAP_rcc01335	-1.81	0	1139	292	0.02%	0.00%	625	1583	232	352	*Fis family GAF modulated sigma54 specific transcriptional regulator*	Signal Transduction	Transcription Regulator
RCAP_rcc01336	-2.56	NA	2008	169	0.03%	0.00%	-751	4622	97	241	*acxA*	Amino Acid Metabolism	Arginine and proline metabolism
RCAP_rcc01337	-2.19	NA	2135	316	0.03%	0.00%	-613	4738	237	395	*acxB*	Amino Acid Metabolism	Unknown
RCAP_rcc01338	-2.23	NA	482	73	0.01%	0.00%	-16	953	50	96	*acxC*	Secondary metabolites biosynthesis, transport, and catabolism	Unknown
RCAP_rcc01339	-0.64	NA	42	26	0.00%	0.00%	19	62	23	29	*hypothetical protein*	Unknown	Unknown
RCAP_rcc01340	0.23	0.6087	20	24	0.00%	0.00%	10	28	12	35	*LysR family transcriptional regulator*	Signal Transduction	Transcription Regulator
RCAP_rcc01341	1.07	0.0024	21	49	0.00%	0.00%	13	29	30	67	*hypothetical protein*	Unknown	Unknown
RCAP_rcc01342	-1.08	0	845	382	0.01%	0.01%	746	929	253	511	*iron siderophore/cobalamin ABC transporter periplasmic iron siderophc*	Metal, Ion, Cofactor Transport	Iron and Heme Transport
RCAP_rcc01343	-2.03	0	176	38	0.00%	0.00%	141	196	23	54	*iron siderophore/cobalamin ABC transporter permease*	Metal, Ion, Cofactor Transport	Iron and Heme Transport
RCAP_rcc01345	-0.44	0.0656	39	29	0.00%	0.00%	28	48	24	33	*hypothetical protein*	Unknown	Unknown
RCAP_rcc01347	0.6	0.0001	152	233	0.00%	0.00%	140	176	192	274	*pirin domain-containing protein*	Unknown	Unknown
RCAP_rcc01348	0.4	0.0004	67	88	0.00%	0.00%	62	74	79	96	*exonuclease*	Replication, Recombination and Repair	Replication
RCAP_rcc01349	0.74	0.0007	158	270	0.00%	0.00%	141	179	180	359	*pirin domain-containing protein*	Unknown	Unknown
RCAP_rcc01350	-0.4	0.0002	955	720	0.01%	0.01%	844	1040	666	774	*hypothetical protein*	Unknown	Unknown
RCAP_rcc01351	-0.24	0.3147	163	137	0.00%	0.00%	122	201	117	158	*hypothetical protein*	Unknown	Unknown
RCAP_rcc01352	-0.72	0	743	448	0.01%	0.01%	624	845	403	493	*cheY1*	Motility	Chemotaxis
RCAP_rcc01353	-0.97	0	2236	1130	0.03%	0.02%	1878	2491	1044	1216	*cheA1*	Motility	Chemotaxis
RCAP_rcc01354	-1.33	0	1099	432	0.02%	0.01%	959	1178	379	486	*cheW1*	Motility	Chemotaxis
RCAP_rcc01355	-1.1	0	11224	5152	0.15%	0.07%	9873	12133	4478	5827	*methyl-accepting chemotaxis sensory transducer*	Motility	Chemotaxis
RCAP_rcc01356	-1.12	0	929	420	0.01%	0.01%	758	1057	377	463	*chemotaxis protein CheW*	Motility	Chemotaxis
RCAP_rcc01357	-1.05	0	1135	542	0.02%	0.01%	985	1244	486	599	*cheR2*	Motility	Chemotaxis
RCAP_rcc01358	-1.19	0	737	316	0.01%	0.00%	561	871	288	344	*cheB1*	Signal Transduction	Transcription Regulator
RCAP_rcc01359	-0.1	0.6877	176	164	0.00%	0.00%	137	211	141	187	*hypothetical protein*	Unknown	Unknown
RCAP_rcc01360	-0.04	0.9035	72	69	0.00%	0.00%	55	86	45	94	*adenylate/guanylate cyclase/CHASE2 sensor domain-containing protei*	Nucleotide Metabolism	Purine metabolism
RCAP_rcc01361	0.31	0.4638	54	69	0.00%	0.00%	30	74	34	105	*Crp/Fnr family transcriptional regulator*	Signal Transduction	Transcription Regulator
RCAP_rcc01362	-0.23	0.1763	105	88	0.00%	0.00%	87	121	79	98	*hemolysin-type calcium-binding repeat family protein*	Trafficking and Secretion	Secretion
RCAP_rcc01363	0.01	0.9527	7993	8058	0.11%	0.11%	7094	9010	6801	9315	*leuA*	Carbohydrate Metabolism	Pyruvate metabolism
RCAP_rcc01364	-0.48	0	2010	1433	0.03%	0.02%	1851	2126	1353	1513	*signal transduction histidine kinase*	Signal Transduction	Kinase/Phosphorelay
RCAP_rcc01365	0.38	0.0025	543	712	0.01%	0.01%	497	608	637	788	*MORN repeat family protein*	Unknown	Unknown
RCAP_rcc01366	0.61	0	619	950	0.01%	0.01%	574	688	857	1044	*nadE*	Metabolism of Cofactors, Coenzymes and Vitamins	Nicotinate and nicotinamide metabolism
RCAP_rcc01367	-0.03	0.8691	225	220	0.00%	0.00%	205	246	189	250	*hypothetical protein*	Unknown	Unknown
RCAP_rcc01368	-0.02	0.9312	1370	1348	0.02%	0.02%	1167	1631	1085	1612	*antibiotic biosynthesis monooxygenase*	Unknown	Unknown
RCAP_rcc01369	-1.05	0	14317	6810	0.20%	0.09%	11609	16327	5902	7717	*ABC transporter periplasmic substrate-binding protein*	Metal and Ion Transport	Unknown
RCAP_rcc01370	-0.96	0	1842	941	0.03%	0.01%	1607	1990	839	1043	*ABC transporter permease*	Unknown	Unknown
RCAP_rcc01371	-1.15	0	2006	886	0.03%	0.01%	1695	2223	751	1022	*ABC transporter permease*	Amino Acid Metabolism	Amino Acid Transport
RCAP_rcc01372	-1.15	0	2042	911	0.03%	0.01%	1799	2170	814	1008	*ABC transporter ATP-binding protein*	Amino Acid Metabolism	Amino Acid Transport
RCAP_rcc01373	-0.96	0	382	193	0.01%	0.00%	314	428	173	213	*RpiR family transcriptional regulator*	Signal Transduction	Transcription Regulator
RCAP_rcc01374	-0.21	0.2369	346	299	0.00%	0.00%	291	393	258	341	*dctQ1*	Unknown	Unknown
RCAP_rcc01375	-0.37	0.0062	1287	990	0.02%	0.01%	1119	1427	878	1102	*dctM1*	Secondary metabolites biosynthesis, transport, and catabolism	Unknown
RCAP_rcc01376	-0.77	0.0001	5022	2874	0.07%	0.04%	4106	5764	2281	3467	*dctP1*	Unknown	Unknown
RCAP_rcc01377	-0.64	0.0007	2972	1872	0.04%	0.03%	2477	3364	1520	2224	*glnA2*	Carbohydrate Metabolism	Glyoxylate and dicarboxylate metabolism
RCAP_rcc01378	-0.44	0.0001	5184	3814	0.07%	0.05%	4661	5628	3431	4196	*aldehyde dehydrogenase*	Energy Metabolism	Limonene and pinene degradation
RCAP_rcc01379	-0.48	0.0002	6180	4409	0.09%	0.06%	5423	6836	3910	4907	*iron-containing alcohol dehydrogenase*	Xenobiotics Biodegradation and Metabolism	Drug metabolism - cytochrome P450
RCAP_rcc01380	-0.56	0	7193	4876	0.10%	0.07%	6551	7580	4483	5269	*hypothetical protein*	Unknown	Unknown
RCAP_rcc01381	-0.32	0.1326	892	707	0.01%	0.01%	684	1063	635	778	*lipoprotein*	Predicted Function	Unknown
RCAP_rcc01382	0.46	0.1474	8382	11892	0.12%	0.16%	5992	11426	9845	13939	*hypothetical protein*	Unknown	Unknown
RCAP_rcc01383	0.01	0.9475	2056	2074	0.03%	0.03%	1700	2394	1877	2272	*ugd*	Carbohydrate Metabolism	Pentose and glucuronate interconversions
RCAP_rcc01384	0.75	0	1617	2736	0.02%	0.04%	1504	1926	2440	3031	*uvrB*	Replication, Recombination and Repair	Repair
RCAP_rcc01385	0.33	0.0123	2054	2590	0.03%	0.04%	1912	2330	2258	2921	*NDUFA4 family NADH ubiquinone oxidoreductase subunit*	Energy Metabolism	Unknown
RCAP_rcc01386	-0.15	0.6085	22	19	0.00%	0.00%	16	26	15	24	*hypothetical protein*	Unknown	Unknown
RCAP_rcc01387	0.09	0.4926	117	124	0.00%	0.00%	104	127	111	137	*RpiR family transcriptional regulator*	Signal Transduction	Transcription Regulator
RCAP_rcc01388	-0.48	0.0442	450	318	0.01%	0.00%	358	542	247	389	*potD2*	Amino Acid Metabolism	Amino Acid Transport
RCAP_rcc01389	-0.62	0.0001	122	79	0.00%	0.00%	107	135	67	92	*potA2*	Amino Acid Metabolism	Amino Acid Transport
RCAP_rcc01390	-0.8	0.0002	266	149	0.00%	0.00%	206	315	121	177	*potB2*	Metal and Ion Transport	Unknown
RCAP_rcc01391	-0.53	0.0076	150	103	0.00%	0.00%	125	176	85	120	*potC1*	Amino Acid Metabolism	Amino Acid Transport
RCAP_rcc01392	-0.7	0.0002	107	65	0.00%	0.00%	84	129	56	75	*S58 family peptidase*	Post-translational Modification, Assembly and Chaperones	Peptidase
RCAP_rcc01393	-1.81	NA	140	34	0.00%	0.00%	59	211	30	39	*LuxR family two component transcriptional regulator*	Signal Transduction	Transcription Regulator
RCAP_rcc01394	-0.3	0.1672	16	12	0.00%	0.00%	13	19	10	14	*hypothetical protein*	Unknown	Unknown
RCAP_rcc01395	-0.25	0.1854	35	30	0.00%	0.00%	27	43	27	32	*pentapeptide repeat family protein*	Unknown	Unknown
RCAP_rcc01396	-0.86	0	209	112	0.00%	0.00%	148	263	99	126	*exaA1*	Carbohydrate Metabolism	Glycolysis / Gluconeogenesis
RCAP_rcc01397	-0.23	0.647	5	4	0.00%	0.00%	2	8	2	6	*metallo-beta-lactamase*	Unknown	Unknown
RCAP_rcc01398	-0.62	0.1122	7	4	0.00%	0.00%	2	11	3	5	*hypothetical protein*	Unknown	Unknown
RCAP_rcc01399	-0.12	0.6936	12	11	0.00%	0.00%	8	16	8	13	*rhodanese domain-containing protein*	Metal and Ion Transport	Unknown
RCAP_rcc01400	-1.17	NA	125	49	0.00%	0.00%	45	195	43	56	*signal transduction histidine kinase*	Signal Transduction	Kinase/Phosphorelay
RCAP_rcc01401	-0.21	0.0395	146	127	0.00%	0.00%	131	158	119	135	*AraC family transcriptional regulator*	Signal Transduction	Transcription Regulator
RCAP_rcc01402	-0.41	0.0014	3107	2326	0.04%	0.03%	2807	3357	2040	2611	*ABC transporter substrate-binding protein*	Unknown	Unknown
RCAP_rcc01403	-0.6	0	1343	884	0.02%	0.01%	1199	1438	810	959	*ABC transporter permease*	Metal and Ion Transport	Unknown

RCAP_rcc01404	-0.66	0	688	434	0.01%	0.01%	617	724	397	471	*ABC transporter ATP-binding protein*	Metal and Ion Transport	Unknown
RCAP_rcc01405	-0.26	0.0881	121	101	0.00%	0.00%	105	134	88	114	*hypothetical protein*	Unknown	Unknown
RCAP_rcc01406	-0.46	0.202	199	139	0.00%	0.00%	138	254	74	203	*phage lysozyme*	Replication, Recombination and Repair	Phage Interaction
RCAP_rcc01407	-0.08	0.8644	22	20	0.00%	0.00%	12	31	9	31	*hypothetical protein*	Unknown	Unknown
RCAP_rcc01408	-0.16	NA	30	26	0.00%	0.00%	10	49	9	44	*hypothetical protein*	Unknown	Unknown
RCAP_rcc01409	-0.25	0.5473	210	171	0.00%	0.00%	139	279	79	263	*hypothetical protein*	Unknown	Unknown
RCAP_rcc01410	0.11	0.8128	89	97	0.00%	0.00%	52	124	51	143	*hypothetical protein*	Unknown	Unknown
RCAP_rcc01412	0.51	0.1441	12	18	0.00%	0.00%	8	17	11	25	*hypothetical protein*	Unknown	Unknown
RCAP_rcc01413	0.62	0.0853	40	64	0.00%	0.00%	27	54	32	97	*hypothetical protein*	Unknown	Unknown
RCAP_rcc01414	0.62	0.033	10	15	0.00%	0.00%	8	12	10	21	*hypothetical protein*	Unknown	Unknown
RCAP_rcc01415	-0.26	0.3791	3458	2852	0.05%	0.04%	2344	4522	2241	3463	*serine/threonine-protein kinase*	Signal Transduction	Kinase/Phosphorelay
RCAP_rcc01416	0.01	0.947	271	273	0.00%	0.00%	237	307	251	295	*hypothetical protein*	Unknown	Unknown
RCAP_rcc01417	0.5	0.2418	6	8	0.00%	0.00%	3	8	5	11	*hypothetical protein*	Unknown	Unknown
RCAP_rcc01418	1.27	0	90	227	0.00%	0.00%	73	118	180	274	*hypothetical protein*	Unknown	Unknown
RCAP_rcc01419	1.16	0	73	170	0.00%	0.00%	69	97	133	208	*hypothetical protein*	Unknown	Unknown
RCAP_rcc01420	2.3	0	11	68	0.00%	0.00%	9	18	37	99	*S49 family peptidase*	Post-translational Modification, Assembly and Chaperones	Peptidase
RCAP_rcc01421	0.16	0.494	29	33	0.00%	0.00%	23	37	27	39	*fldA*	Energy Metabolism	Unknown
RCAP_rcc01422	1.25	0	10	25	0.00%	0.00%	9	14	21	29	*hypothetical protein*	Unknown	Unknown
RCAP_rcc01423	-0.41	0.0077	1694	1264	0.02%	0.02%	1460	1892	1076	1452	*hypothetical protein*	Unknown	Unknown
RCAP_rcc01424	0.41	0.1093	15	20	0.00%	0.00%	12	18	14	26	*hypothetical protein*	Unknown	Unknown
RCAP_rcc01425	-0.02	0.9432	72	70	0.00%	0.00%	52	90	62	78	*hypothetical protein*	Unknown	Unknown
RCAP_rcc01426	0.37	0.0663	25	33	0.00%	0.00%	22	29	25	40	*ABC transporter ATP-binding/permease*	Unknown	Unknown
RCAP_rcc01427	0.56	0.1394	10	15	0.00%	0.00%	6	13	10	20	*ABC transporter ATP-binding/permease*	Unknown	Unknown
RCAP_rcc01428	0.62	0.082	4	7	0.00%	0.00%	3	6	4	10	*major facilitator superfamily protein*	Unknown	Unknown
RCAP_rcc01429	-0.17	0.2398	70	62	0.00%	0.00%	60	78	56	68	*TonB-dependent receptor*	Metal and Ion Transport	Unknown
RCAP_rcc01430	0.91	0	23	44	0.00%	0.00%	20	27	36	52	*O-methyltransferase domain-containing protein*	Secondary metabolites biosynthesis, transport, and catabolism	Unknown
RCAP_rcc01431	0.58	0	50	74	0.00%	0.00%	46	57	65	82	*AraC family transcriptional regulator*	Signal Transduction	Transcription Regulator
RCAP_rcc01432	-0.41	0.0071	427	319	0.01%	0.00%	365	472	274	363	*AraC family transcriptional regulator*	Signal Transduction	Transcription Regulator
RCAP_rcc01433	-0.86	0	152	82	0.00%	0.00%	126	171	72	92	*TonB-dependent siderophore receptor*	Metal, Ion, Cofactor Transport	Iron and Heme Transport
RCAP_rcc01434	-0.57	0.0073	130	86	0.00%	0.00%	105	150	66	105	*fepB1*	Metal, Ion, Cofactor Transport	Iron and Heme Transport
RCAP_rcc01435	-0.62	0.1483	24	14	0.00%	0.00%	13	33	4	24	*fepD1*	Metal, Ion, Cofactor Transport	Iron and Heme Transport
RCAP_rcc01436	-0.94	0.0001	40	19	0.00%	0.00%	32	46	14	25	*fepG1*	Metal, Ion, Cofactor Transport	Iron and Heme Transport
RCAP_rcc01437	-1.13	0	35	15	0.00%	0.00%	26	41	10	19	*fepC1*	Metal, Ion, Cofactor Transport	Iron and Heme Transport
RCAP_rcc01438	-0.84	0.0169	25	13	0.00%	0.00%	14	34	8	17	*siderophore-interacting protein*	Metal, Ion, Cofactor Transport	Iron and Heme Transport
RCAP_rcc01439	-0.55	0.1175	223	145	0.00%	0.00%	131	298	94	197	*ABC transporter ATP-binding/permease*	Metal, Ion, Cofactor Transport	Iron and Heme Transport
RCAP_rcc01440	-1.46	0	191	65	0.00%	0.00%	130	235	51	79	*ABC transporter ATP-binding/permease*	Metal, Ion, Cofactor Transport	Iron and Heme Transport
RCAP_rcc01441	-1.07	0.0001	68	30	0.00%	0.00%	49	83	20	40	*fepC2*	Metal, Ion, Cofactor Transport	Iron and Heme Transport
RCAP_rcc01442	-0.68	0.069	31	17	0.00%	0.00%	21	38	5	29	*fepG2*	Metal, Ion, Cofactor Transport	Iron and Heme Transport
RCAP_rcc01443	-0.72	0.0579	23	12	0.00%	0.00%	16	28	3	22	*fepD2*	Metal, Ion, Cofactor Transport	Iron and Heme Transport
RCAP_rcc01444	-0.2	0.5654	91	77	0.00%	0.00%	72	106	33	122	*fepB2*	Metal, Ion, Cofactor Transport	Iron and Heme Transport
RCAP_rcc01445	0.24	0.3794	120	143	0.00%	0.00%	92	160	119	168	*tonB-dependent receptor*	Metal and Ion Transport	Unknown
RCAP_rcc01446	-0.23	0.0929	3470	2960	0.05%	0.04%	2933	3924	2742	3178	*M20 family peptidase*	Post-translational Modification, Assembly and Chaperones	Peptidase
RCAP_rcc01447	0.28	0.1399	10931	13357	0.16%	0.18%	9643	12653	11243	15471	*hemA*	Metabolism of Cofactors, Coenzymes and Vitamins	Heme Biosynthesis
RCAP_rcc01448	-0.21	0.0184	5019	4322	0.07%	0.06%	4538	5438	4039	4606	*hypothetical protein*	Unknown	Unknown
RCAP_rcc01449	0.5	0	3413	4828	0.05%	0.07%	3388	3689	4421	5236	*ispG*	Lipid	Terpenoid backbone biosynthesis
RCAP_rcc01450	-0.06	0.6252	1518	1452	0.02%	0.02%	1353	1690	1339	1564	*serS*	Translation, ribosomal structure and biogenesis	Aminoacyl-tRNA biosynthesis
RCAP_rcc01451	0.56	0.0007	33	49	0.00%	0.00%	28	40	44	55	*RND family efflux transporter subunit MFP*	Cell Envelope Biosynthesis	Cell Wall Biosynthesis
RCAP_rcc01452	0.02	0.901	317	320	0.00%	0.00%	290	348	305	336	*acriflavin resistance protein family*	Metal and Ion Transport	Unknown
RCAP_rcc01453	-0.08	0.7446	720	679	0.01%	0.01%	546	876	595	764	*ArsR family transcriptional regulator*	Signal Transduction	Transcription Regulator
RCAP_rcc01454	0.19	0.0118	417	473	0.01%	0.01%	401	436	438	508	*corA*	Metal, Ion, Cofactor Transport	Cobalt Transport
RCAP_rcc01455	1.65	0	196	687	0.00%	0.01%	156	271	474	900	*hypothetical protein*	Unknown	Unknown
RCAP_rcc01456	0.54	0.0727	257	386	0.00%	0.01%	185	333	308	464	*MarC family membrane protein*	Trafficking and Secretion	Trafficing
RCAP_rcc01457	0.14	0.6654	63	70	0.00%	0.00%	47	77	48	92	*DeoR family transcriptional regulator*	Signal Transduction	Transcription Regulator
RCAP_rcc01458	0.71	0	2081	3455	0.03%	0.05%	1869	2509	3031	3880	*tatA*	Trafficking and Secretion	Secretion
RCAP_rcc01459	0.85	0	862	1580	0.01%	0.02%	761	1045	1361	1799	*tatB*	Trafficking and Secretion	Secretion
RCAP_rcc01460	0.42	0.0003	3052	4102	0.04%	0.06%	2834	3422	3694	4511	*tatC*	Trafficking and Secretion	Secretion
RCAP_rcc01461	0.25	0.0702	530	631	0.01%	0.01%	467	596	563	700	*ATPase AAA*	Unknown	Unknown
RCAP_rcc01462	-1.19	0	23788	10162	0.32%	0.14%	19118	27017	8626	11698	*nrdJ2*	Metabolism of Other Amino Acids	Glutathione metabolism
RCAP_rcc01463	-1.38	0	5801	2138	0.08%	0.03%	4275	6969	1799	2477	*hypothetical protein*	Unknown	Unknown
RCAP_rcc01464	-1.09	0	2416	1111	0.03%	0.02%	1962	2733	967	1256	*hypothetical protein*	Unknown	Unknown
RCAP_rcc01465	-0.89	0	3531	1877	0.05%	0.03%	3022	3859	1618	2136	*hypothetical protein*	Unknown	Unknown
RCAP_rcc01466	-0.09	0.4646	1304	1230	0.02%	0.02%	1209	1397	1109	1351	*glutamate synthase domain-containing protein*	Energy Metabolism	Nitrogen metabolism
RCAP_rcc01467	0.66	0.0521	89	150	0.00%	0.00%	59	117	101	199	*gcvA*	Transcription	Unknown
RCAP_rcc01468	-2.62	NA	302	24	0.00%	0.00%	42	531	17	30	*FAD dependent oxidoreductase*	Energy Metabolism	Unknown
RCAP_rcc01469	-2.35	NA	342	46	0.00%	0.00%	55	599	39	53	*hypothetical protein*	Unknown	Unknown
RCAP_rcc01470	-2.43	NA	1140	157	0.02%	0.00%	209	1968	146	168	*L-aminoadipate-semialdehyde dehydrogenase*	Carbohydrate Metabolism	Glycolysis / Gluconeogenesis
RCAP_rcc01471	-2.02	NA	937	189	0.01%	0.00%	240	1553	180	199	*aspC1*	Energy Metabolism	Tropane, piperidine and pyridine alkaloid biosynthesis
RCAP_rcc01472	-0.52	NA	612	415	0.01%	0.01%	252	966	390	441	*aroQ*	Amino Acid Metabolism	Phenylalanine, tyrosine and tryptophan biosynthesis
RCAP_rcc01473	0.04	0.8615	104	106	0.00%	0.00%	82	127	96	117	*AraC family transcriptional regulator*	Signal Transduction	Transcription Regulator
RCAP_rcc01474	-0.9	0	103	54	0.00%	0.00%	78	124	47	62	*amino acid permease*	Amino Acid Metabolism	Unknown
RCAP_rcc01475	-0.69	0.0028	40	24	0.00%	0.00%	29	49	21	27	*ocd*	Amino Acid Metabolism	Arginine and proline metabolism
RCAP_rcc01476	-0.15	0.6753	8	7	0.00%	0.00%	5	11	5	8	*endoribonuclease L-PSP family protein*	Unknown	Unknown
RCAP_rcc01477	0.21	0.5051	9	10	0.00%	0.00%	7	11	7	13	*FAD dependent oxidoreductase*	Amino Acid Metabolism	Phenylalanine metabolism
RCAP_rcc01478	0.07	0.82	258	272	0.00%	0.00%	183	333	227	316	*CIA30 family NADH ubiquinone oxidoreductase subunit*	Energy Metabolism	Unknown
RCAP_rcc01479	-0.44	0.0539	49	35	0.00%	0.00%	38	57	28	43	*hypothetical protein*	Unknown	Unknown
RCAP_rcc01480	-0.33	0.04	339	268	0.00%	0.00%	301	377	223	314	*transmembrane pair family protein*	Unknown	Unknown
RCAP_rcc01481	-0.67	0	1038	652	0.01%	0.01%	906	1128	608	696	*murE*	Glycan Biosynthesis and Metabolism	Peptidoglycan biosynthesis

RCAP_rcc01482	-0.41	0.0001	1100	828	0.02%	0.01%	961	1197	766	890	*murF*	Glycan Biosynthesis and Metabolism	Peptidoglycan biosynthesis
RCAP_rcc01483	0.34	0.0032	2216	2816	0.03%	0.04%	2066	2442	2532	3100	*mraY*	Glycan Biosynthesis and Metabolism	Peptidoglycan biosynthesis
RCAP_rcc01484	1.11	0	1471	3203	0.02%	0.04%	1506	1694	2899	3506	*hypothetical protein*	Unknown	Unknown
RCAP_rcc01485	0.25	0.0006	1107	1321	0.02%	0.02%	1031	1195	1268	1374	*murD*	Glycan Biosynthesis and Metabolism	Peptidoglycan biosynthesis
RCAP_rcc01486	-0.16	0.1013	246	219	0.00%	0.00%	220	268	206	232	*hypothetical protein*	Unknown	Unknown
RCAP_rcc01487	-0.06	0.6433	115	111	0.00%	0.00%	103	128	100	122	*glutathione-dependent formaldehyde-activating enzyme family protein*	Sulfur Metabolism	Glutathione metabolism
RCAP_rcc01488	-0.17	0.333	1591	1414	0.02%	0.02%	1267	1900	1299	1529	*purM*	Nucleotide Metabolism	Purine metabolism
RCAP_rcc01489	-0.45	0	706	516	0.01%	0.01%	638	750	466	567	*purN*	Metabolism of Cofactors, Coenzymes and Vitamins	One carbon pool by folate
RCAP_rcc01490	-0.15	0.3295	4148	3718	0.06%	0.05%	3557	4685	3212	4224	*rnd2*	Translation, ribosomal structure and biogenesis	Unknown
RCAP_rcc01491	-0.33	0.0366	4023	3178	0.06%	0.04%	3274	4673	2884	3471	*uvrD*	Replication, Recombination and Repair	Repair
RCAP_rcc01492	-0.58	0.0135	315	206	0.00%	0.00%	202	422	184	228	*hypothetical protein*	Unknown	Unknown
RCAP_rcc01493	0.3	0.1659	5895	7297	0.09%	0.10%	4622	7682	6393	8201	*hypothetical protein*	Unknown	Unknown
RCAP_rcc01494	-0.21	0.1603	1987	1719	0.03%	0.02%	1735	2204	1530	1909	*mdeA*	Amino Acid Metabolism	Cysteine and methionine metabolism
RCAP_rcc01495	2.27	0	1937	10428	0.03%	0.14%	1724	2801	8138	12717	*fusA2*	Signal Transduction	Kinase/Phosphorelay
RCAP_rcc01496	0.12	0.2717	1202	1305	0.02%	0.02%	1095	1339	1229	1381	*lipoprotein*	Predicted Function	Unknown
RCAP_rcc01497	0.5	0.0001	258	367	0.00%	0.00%	232	300	341	392	*sufE*	Unknown	Unknown
RCAP_rcc01498	0.23	0.0177	458	538	0.01%	0.01%	419	493	492	584	*puuD*	Unknown	Unknown
RCAP_rcc01499	-0.1	0.6577	506	470	0.01%	0.01%	395	603	403	536	*DNA alkylation repair enzyme family protein*	Replication, Recombination and Repair	Repair
RCAP_rcc01500	-0.08	0.4341	2575	2442	0.04%	0.03%	2376	2772	2279	2605	*glmS*	Carbohydrate Metabolism	Amino sugar and nucleotide sugar metabolism
RCAP_rcc01501	0.14	0.2126	1261	1390	0.02%	0.02%	1126	1398	1341	1439	*glmU*	Carbohydrate Metabolism	Amino sugar and nucleotide sugar metabolism
RCAP_rcc01502	0.47	0.0906	177	250	0.00%	0.00%	119	228	189	312	*gph2*	Carbohydrate Metabolism	Glyoxylate and dicarboxylate metabolism
RCAP_rcc01503	0.02	0.8203	430	437	0.01%	0.01%	395	458	416	457	*tetA*	Metal and Ion Transport	Unknown
RCAP_rcc01504	-0.16	0.0945	428	380	0.01%	0.01%	395	450	346	415	*DegT/DnrJ/EryC1/StrS family aminotransferase*	Cell Envelope Biosynthesis	Cell Wall Biosynthesis
RCAP_rcc01505	-0.7	0.0001	156	95	0.00%	0.00%	127	178	82	107	*hypothetical protein*	Unknown	Unknown
RCAP_rcc01506	-0.28	0.0055	1347	1106	0.02%	0.02%	1211	1431	1003	1208	*signal transduction histidine kinase*	Signal Transduction	Kinase/Phosphorelay
RCAP_rcc01507	-0.23	0.0393	434	367	0.01%	0.00%	388	472	330	405	*hypothetical protein*	Unknown	Unknown
RCAP_rcc01508	0.42	0.0458	71	96	0.00%	0.00%	59	82	72	121	*LuxR family two component transcriptional regulator*	Signal Transduction	Transcription Regulator
RCAP_rcc01509	0.18	0.491	24	27	0.00%	0.00%	19	30	20	34	*hypothetical protein*	Unknown	Unknown
RCAP_rcc01510	1.22	0.0021	273	767	0.00%	0.01%	193	337	335	1199	*ivdH*	Lipid Metabolism	Unknown
RCAP_rcc01511	0.92	0.0533	76	181	0.00%	0.00%	37	112	59	303	*hypothetical protein*	Unknown	Unknown
RCAP_rcc01512	1.02	0.0028	424	950	0.01%	0.01%	331	494	488	1411	*mccB*	Amino Acid Metabolism	Valine, leucine and isoleucine degradation
RCAP_rcc01513	1.28	NA	123	345	0.00%	0.00%	96	145	155	535	*mccA*	Amino Acid Metabolism	Valine, leucine and isoleucine degradation
RCAP_rcc01514	1.06	0.0003	81	181	0.00%	0.00%	66	94	110	251	*glutathione S-transferase*	Sulfur Metabolism	Glutathione metabolism
RCAP_rcc01515	1.08	0.0067	57	140	0.00%	0.00%	34	75	68	212	*hmgL*	Carbohydrate Metabolism	Butanoate metabolism
RCAP_rcc01516	0.83	0.0126	69	130	0.00%	0.00%	50	84	75	186	*menB*	Amino Acid Metabolism	Valine, leucine and isoleucine degradation
RCAP_rcc01517	-0.87	0.0003	5654	2984	0.08%	0.04%	3682	7292	2494	3473	*nuoA*	Energy Metabolism	Aerobic/Anaerobic Respiration
RCAP_rcc01518	-0.56	0	2447	1659	0.03%	0.02%	2124	2669	1566	1751	*nuoB*	Energy Metabolism	Aerobic/Anaerobic Respiration
RCAP_rcc01519	-1.01	0	5923	2889	0.08%	0.04%	4824	6686	2600	3177	*nuoC*	Energy Metabolism	Aerobic/Anaerobic Respiration
RCAP_rcc01520	-1.28	0	11885	4783	0.16%	0.06%	9613	13414	4159	5408	*nuoD*	Energy Metabolism	Aerobic/Anaerobic Respiration
RCAP_rcc01521	-1.43	0	11848	4289	0.16%	0.06%	9455	13416	3637	4941	*nuoE*	Energy Metabolism	Aerobic/Anaerobic Respiration
RCAP_rcc01522	-0.43	0.0055	4137	3061	0.06%	0.04%	3358	4779	2753	3370	*hypothetical protein*	Unknown	Unknown
RCAP_rcc01523	-1.16	0	8625	3830	0.12%	0.05%	7833	8921	3389	4270	*nuoF*	Energy Metabolism	Aerobic/Anaerobic Respiration
RCAP_rcc01524	-1.18	0	753	323	0.01%	0.00%	620	825	263	383	*hypothetical protein*	Unknown	Unknown
RCAP_rcc01525	-1.14	0	111	49	0.00%	0.00%	90	124	40	58	*hypothetical protein*	Unknown	Unknown
RCAP_rcc01526	-1.28	0	2925	1181	0.04%	0.02%	2561	3100	1000	1362	*hypothetical protein*	Unknown	Unknown
RCAP_rcc01527	-1.21	0	10243	4408	0.14%	0.06%	9534	10314	3954	4863	*nuoG*	Energy Metabolism	Aerobic/Anaerobic Respiration
RCAP_rcc01528	-1.1	0	665	304	0.01%	0.00%	532	752	266	342	*hypothetical protein*	Unknown	Unknown
RCAP_rcc01529	-1.44	0	7976	2868	0.11%	0.04%	6587	8844	2401	3335	*nuoH*	Energy Metabolism	Aerobic/Anaerobic Respiration
RCAP_rcc01530	-1.56	0	2759	900	0.04%	0.01%	2131	3194	745	1056	*hypothetical protein*	Unknown	Unknown
RCAP_rcc01531	-1.49	0	2780	975	0.04%	0.01%	2410	2953	841	1109	*nuoI*	Energy Metabolism	Aerobic/Anaerobic Respiration
RCAP_rcc01532	-1.57	0	2760	914	0.04%	0.01%	2362	2957	769	1059	*pcaC*	Xenobiotics Biodegradation and Metabolism	Benzoate degradation via hydroxylation
RCAP_rcc01533	-1.37	0	3749	1434	0.05%	0.02%	3358	3894	1245	1623	*nuoJ*	Energy Metabolism	Aerobic/Anaerobic Respiration
RCAP_rcc01534	-1.29	0	4537	1805	0.06%	0.02%	3720	5066	1467	2144	*nuoK*	Energy Metabolism	Aerobic/Anaerobic Respiration
RCAP_rcc01535	-1.34	0	20721	7999	0.28%	0.11%	17263	22872	6724	9275	*nuoL*	Energy Metabolism	Aerobic/Anaerobic Respiration
RCAP_rcc01536	-1.37	0	14495	5426	0.20%	0.07%	11443	16638	4455	6397	*nuoM*	Energy Metabolism	Aerobic/Anaerobic Respiration
RCAP_rcc01537	-1.19	0	8335	3625	0.11%	0.05%	7580	8617	3169	4081	*nuoN*	Energy Metabolism	Aerobic/Anaerobic Respiration
RCAP_rcc01538	-1.09	0	540	249	0.01%	0.00%	432	613	220	278	*birA1*	Metabolism of Cofactors, Coenzymes and Vitamins	Biotin metabolism
RCAP_rcc01539	-0.55	0.0026	1163	784	0.02%	0.01%	942	1332	652	917	*coaX*	Metabolism of Cofactors, Coenzymes and Vitamins	Pantothenate and CoA biosynthesis
RCAP_rcc01540	-0.27	0.0395	5579	4618	0.08%	0.06%	4983	6046	4064	5173	*metallo-beta-lactamase*	Unknown	Unknown
RCAP_rcc01541	1.75	0	1164	3999	0.02%	0.05%	1237	1541	3525	4473	*hypothetical protein*	Unknown	Unknown
RCAP_rcc01542	-0.31	0.0537	922	740	0.01%	0.01%	784	1042	640	839	*ilvH*	Metabolism of Cofactors, Coenzymes and Vitamins	Pantothenate and CoA biosynthesis
RCAP_rcc01543	-0.55	0.0079	3789	2553	0.05%	0.03%	2959	4477	2098	3008	*ilvI*	Metabolism of Cofactors, Coenzymes and Vitamins	Pantothenate and CoA biosynthesis
RCAP_rcc01544	0.3	0.2426	1927	2408	0.03%	0.03%	1431	2459	1960	2856	*dctQ2*	Secondary metabolites biosynthesis, transport, and catabolism	Unknown
RCAP_rcc01545	0.34	0.0555	3037	3873	0.04%	0.05%	2516	3621	3409	4336	*dctM2*	Secondary metabolites biosynthesis, transport, and catabolism	Unknown
RCAP_rcc01546	-0.37	0.0085	2782	2142	0.04%	0.03%	2416	3080	1842	2441	*AMP-dependent synthetase and ligase*	Energy Metabolism	Tropane, piperidine and pyridine alkaloid biosynthesis
RCAP_rcc01547	-0.15	0.4662	3020	2706	0.04%	0.04%	2432	3597	2315	3097	*prfB*	Translation, ribosomal structure and biogenesis	Unknown
RCAP_rcc01548	-0.35	0.0281	237	185	0.00%	0.00%	198	266	161	208	*shikimate kinase domain-containing protein*	Nucleotide Metabolism	Unknown
RCAP_rcc01549	-0.16	0.6577	1433	1270	0.02%	0.02%	795	2024	1024	1516	*hypothetical protein*	Unknown	Unknown
RCAP_rcc01550	-0.95	0	2286	1173	0.03%	0.02%	1990	2479	1078	1268	*M23 family peptidase*	Post-translational Modification, Assembly and Chaperones	Peptidase
RCAP_rcc01551	-0.27	0.1575	1479	1218	0.02%	0.02%	1220	1784	1034	1401	*bcp*	Metabolism of Other Amino Acids	Glutathione metabolism
RCAP_rcc01552	0.01	0.9623	1013	1020	0.01%	0.01%	828	1167	856	1184	*hypothetical protein*	Unknown	Unknown
RCAP_rcc01553	-0.62	0	634	411	0.01%	0.01%	579	658	384	438	*queA*	Lipid Metabolism	Steroid biosynthesis
RCAP_rcc01554	-0.13	0.1201	1180	1075	0.02%	0.01%	1094	1259	1005	1146	*major facilitator superfamily protein*	Carbohydrate Metabolism	Unknown
RCAP_rcc01555	0.81	0.0003	1101	1988	0.02%	0.03%	860	1525	1719	2258	*hypothetical protein*	Unknown	Unknown
RCAP_rcc01556	-0.76	0	6874	4031	0.09%	0.05%	5609	7873	3816	4245	*lpdA2*	Carbohydrate Metabolism	TCA Cycle
RCAP_rcc01557	-0.16	0.2886	111	99	0.00%	0.00%	97	126	88	109	*rhodanese domain-containing protein*	Metal and Ion Transport	Unknown
RCAP_rcc01558	0	0.9745	2816	2825	0.04%	0.04%	2519	3173	2570	3080	*uvrA*	Replication, Recombination and Repair	Repair

RCAP_rcc01559	0.43	0.2232	115	161	0.00%	0.00%	75	155	109	213	*type 11 family methyltransferase*	Unknown	Unknown
RCAP_rcc01560	0.99	0	318	645	0.00%	0.01%	292	415	562	728	*hypothetical protein*	Unknown	Unknown
RCAP_rcc01561	1.88	0	458	1749	0.01%	0.02%	482	604	1448	2050	*Crp/Fnr family transcriptional regulator*	Signal Transduction	Transcription Regulator
RCAP_rcc01562	0.63	0.0681	309	507	0.00%	0.01%	204	398	303	710	*mmsB*	Amino Acid Metabolism	Valine, leucine and isoleucine degradation
RCAP_rcc01563	0.75	0.0271	264	475	0.00%	0.01%	166	353	292	658	*3-hydroxyisobutyryl-CoA hydrolase*	Carbohydrate Metabolism	Propanoate metabolism
RCAP_rcc01564	0.44	0.1818	313	437	0.00%	0.01%	210	404	282	591	*isobutyryl-CoA dehydrogenase*	Amino Acid Metabolism	Valine, leucine and isoleucine degradation
RCAP_rcc01565	0.88	0.0004	1623	3112	0.02%	0.04%	1177	2214	2331	3894	*mmsA*	Carbohydrate Metabolism	Propanoate metabolism
RCAP_rcc01566	-0.03	0.8576	62	60	0.00%	0.00%	54	71	55	66	*LysR family transcriptional regulator*	Signal Transduction	Transcription Regulator
RCAP_rcc01567	0.64	0	317	497	0.00%	0.01%	293	361	454	541	*coaD*	Metabolism of Cofactors, Coenzymes and Vitamins	Pantothenate and CoA biosynthesis
RCAP_rcc01568	0.43	0.0524	48	65	0.00%	0.00%	38	59	55	76	*RES domain family protein*	Unknown	Unknown
RCAP_rcc01569	-0.17	0.2937	1821	1609	0.03%	0.02%	1574	2073	1432	1787	*purF*	Amino Acid Metabolism	Alanine, aspartate and glutamate metabolism
RCAP_rcc01570	0	0.9928	1454	1456	0.02%	0.02%	1352	1570	1334	1578	*cvpA*	Unknown	Unknown
RCAP_rcc01571	0.06	0.6225	725	753	0.01%	0.01%	658	771	684	823	*radA*	Post-translational Modification, Assembly and Chaperones	Unknown
RCAP_rcc01572	-0.51	0	890	621	0.01%	0.01%	777	970	571	672	*ABC transporter ATP-binding protein*	Secondary metabolites biosynthesis, transport, and catabolism	Unknown
RCAP_rcc01573	-0.16	0.1449	883	787	0.01%	0.01%	786	967	732	842	*ABC transporter permease*	Secondary metabolites biosynthesis, transport, and catabolism	Unknown
RCAP_rcc01574	0.1	0.5292	874	938	0.01%	0.01%	744	1006	863	1013	*alr*	Metabolism of Other Amino Acids	D-Alanine metabolism
RCAP_rcc01575	-0.34	0.0144	1824	1431	0.03%	0.02%	1587	2019	1261	1601	*dnaB*	Metabolism of Cofactors, Coenzymes and Vitamins	Folate biosynthesis
RCAP_rcc01576	-0.01	0.9671	1373	1367	0.02%	0.02%	1253	1515	1230	1505	*pyrE*	Xenobiotics Biodegradation and Metabolism	Drug metabolism - other enzymes
RCAP_rcc01577	0.11	0.2959	674	730	0.01%	0.01%	607	748	692	767	*pyrC2*	Nucleotide Metabolism	Pyrimidine metabolism
RCAP_rcc01578	0.17	0.1587	725	815	0.01%	0.01%	679	789	731	900	*lipoprotein*	Predicted Function	Unknown
RCAP_rcc01579	1.98	0	765	3584	0.01%	0.05%	541	1229	2341	4827	*hypothetical protein*	Unknown	Unknown
RCAP_rcc01580	1.82	0	625	2360	0.01%	0.03%	578	898	1787	2933	*hypothetical protein*	Unknown	Unknown
RCAP_rcc01581	0.84	0	888	1608	0.01%	0.02%	802	1053	1313	1903	*hypothetical protein*	Unknown	Unknown
RCAP_rcc01582	0.05	NA	152	156	0.00%	0.00%	136	164	115	197	*hypothetical protein*	Unknown	Unknown
RCAP_rcc01583	-0.37	0	936	721	0.01%	0.01%	834	1012	688	753	*hypothetical protein*	Unknown	Unknown
RCAP_rcc01584	-0.36	0.035	2587	2000	0.04%	0.03%	2157	2973	1714	2286	*hypothetical protein*	Unknown	Unknown
RCAP_rcc01585	-0.04	0.8691	515	499	0.01%	0.01%	403	612	424	575	*hypothetical protein*	Unknown	Unknown
RCAP_rcc01586	-0.4	0.0251	639	480	0.01%	0.01%	503	751	429	530	*GSCFA family protein*	Unknown	Unknown
RCAP_rcc01587	-0.41	0.0408	448	334	0.01%	0.00%	348	536	294	374	*hypothetical protein*	Unknown	Unknown
RCAP_rcc01588	-0.03	NA	114	111	0.00%	0.00%	29	189	28	194	*GSCFA family protein*	Unknown	Unknown
RCAP_rcc01589	-0.72	0	621	372	0.01%	0.01%	520	695	311	433	*ErfK/YbiS/YcfS/YnhG family protein*	Unknown	Unknown
RCAP_rcc01590	-0.26	0.0395	6122	5116	0.09%	0.07%	5696	6663	4501	5731	*infC*	Translation, ribosomal structure and biogenesis	Unknown
RCAP_rcc01591	-0.1	0.5942	3702	3447	0.05%	0.05%	3280	4200	2896	3998	*fpr*	Energy Metabolism	Aerobic/Anaerobic Respiration
RCAP_rcc01592	-0.18	0.5937	358	312	0.00%	0.00%	237	471	258	367	*hypothetical protein*	Unknown	Unknown
RCAP_rcc01593	-0.49	0.0052	1943	1369	0.03%	0.02%	1679	2164	1112	1625	*cysH*	Energy Metabolism	Sulfur metabolism
RCAP_rcc01594	-0.24	0.1202	2804	2367	0.04%	0.03%	2589	3008	1981	2753	*cysI*	Energy Metabolism	Sulfur metabolism
RCAP_rcc01595	0.02	0.9284	264	268	0.00%	0.00%	238	298	234	301	*hypothetical protein*	Unknown	Unknown
RCAP_rcc01596	0.77	0.01	411	739	0.01%	0.01%	301	560	504	975	*cobA1*	Metabolism of Cofactors, Coenzymes and Vitamins	Cobalamin Biosynthesis
RCAP_rcc01597	-0.24	0.0707	317	268	0.00%	0.00%	271	355	246	289	*AsnC/Lrp family transcriptional regulator*	Signal Transduction	Transcription Regulator
RCAP_rcc01598	0.24	0.3137	1170	1388	0.02%	0.02%	838	1524	1208	1568	*tpiA*	Carbohydrate Metabolism	Glycolysis / Gluconeogenesis
RCAP_rcc01599	0.63	0	577	904	0.01%	0.01%	514	676	784	1023	*iscA*	Energy Metabolism	Aerobic/Anaerobic Respiration
RCAP_rcc01600	-0.04	0.8457	2347	2283	0.03%	0.03%	2014	2653	1913	2653	*nemA*	Energy Metabolism	Unknown
RCAP_rcc01601	-0.26	0.1949	1106	915	0.02%	0.01%	902	1323	758	1072	*FeS assembly SUF system protein*	Unknown	Unknown
RCAP_rcc01602	-0.1	0.4549	395	368	0.01%	0.00%	343	449	338	397	*tgt*	Translation, ribosomal structure and biogenesis	Unknown
RCAP_rcc01603	0.1	0.5788	954	1025	0.01%	0.01%	838	1125	889	1161	*hemolysin-III family protein*	Trafficking and Secretion	Secretion
RCAP_rcc01604	-0.1	0.7687	151	139	0.00%	0.00%	96	201	114	164	*hypothetical protein*	Unknown	Unknown
RCAP_rcc01605	0.06	0.6788	601	628	0.01%	0.01%	522	680	586	670	*thiamine monophosphate synthase*	Metabolism of Cofactors, Coenzymes and Vitamins	Thiamine metabolism
RCAP_rcc01606	-0.03	0.8507	422	412	0.01%	0.01%	366	463	365	459	*tRNA/rRNA methyltransferase, SpoU family*	Translation, ribosomal structure and biogenesis	Unknown
RCAP_rcc01607	0.24	0.4766	28	33	0.00%	0.00%	20	34	20	47	*hypothetical protein*	Unknown	Unknown
RCAP_rcc01608	0.9	0	564	1072	0.01%	0.01%	483	689	921	1224	*cytochrome c oxidase assembly protein*	Energy Metabolism	Aerobic/Anaerobic Respiration
RCAP_rcc01609	0.25	0.0192	1682	2007	0.02%	0.03%	1516	1882	1880	2133	*cxp*	Amino Acid Metabolism	Unknown
RCAP_rcc01610	0.35	0.0035	482	617	0.01%	0.01%	445	531	544	691	*hypothetical protein*	Unknown	Unknown
RCAP_rcc01611	1.07	0	446	959	0.01%	0.01%	438	557	748	1170	*M48 family peptidase*	Post-translational Modification, Assembly and Chaperones	Peptidase
RCAP_rcc01612	-0.54	0	701	480	0.01%	0.01%	591	787	440	520	*hypothetical protein*	Unknown	Unknown
RCAP_rcc01613	-1.07	0	1574	714	0.02%	0.01%	1011	2034	612	815	*hypothetical protein*	Unknown	Unknown
RCAP_rcc01614	-0.14	0.5617	4122	3734	0.06%	0.05%	3530	4831	3095	4374	*metH4*	Amino Acid Metabolism	Unknown
RCAP_rcc01615	-0.08	0.8171	1395	1312	0.02%	0.02%	1053	1763	1055	1569	*methylenetetrahydrofolate reductase*	Energy Metabolism	Reductive carboxylate cycle (CO2 fixation)
RCAP_rcc01616	0.47	0.1091	219	313	0.00%	0.00%	158	290	245	381	*hypothetical protein*	Unknown	Unknown
RCAP_rcc01617	0.85	0	788	1449	0.01%	0.02%	664	1027	1206	1691	*gppA*	Nucleotide Metabolism	Purine metabolism
RCAP_rcc01618	0.18	0.136	773	879	0.01%	0.01%	708	857	797	960	*rrmJ*	Translation, ribosomal structure and biogenesis	Unknown
RCAP_rcc01619	-0.98	0.0082	973	442	0.01%	0.01%	482	1398	344	540	*Fis family transcriptional regulator*	Signal Transduction	Transcription Regulator
RCAP_rcc01620	-2.81	0	66679	7709	0.90%	0.10%	35919	91744	5858	9560	*aldB*	Carbohydrate Metabolism	Glycolysis / Gluconeogenesis
RCAP_rcc01621	-0.11	0.5788	632	584	0.01%	0.01%	539	736	481	687	*mcpC*	Motility	Chemotaxis
RCAP_rcc01622	0.28	0.1962	3908	4786	0.06%	0.06%	3336	4820	3891	5682	*hypothetical protein*	Unknown	Unknown
RCAP_rcc01623	-0.39	0.0064	1154	879	0.02%	0.01%	946	1319	820	939	*AraC family transcriptional regulator*	Signal Transduction	Transcription Regulator
RCAP_rcc01624	0.19	0.4926	75	86	0.00%	0.00%	56	93	64	108	*miaA*	Translation, ribosomal structure and biogenesis	Unknown
RCAP_rcc01625	0	0.997	2399	2401	0.03%	0.03%	2029	2806	2146	2655	*pyrH*	Xenobiotics Biodegradation and Metabolism	Drug metabolism - other enzymes
RCAP_rcc01626	0.1	0.4839	2619	2806	0.04%	0.04%	2367	2912	2520	3092	*frr*	Translation, ribosomal structure and biogenesis	Unknown
RCAP_rcc01627	0.08	0.545	812	861	0.01%	0.01%	709	897	783	939	*uppS*	Lipid transport and metabolism	Unknown
RCAP_rcc01628	0.22	0.1899	403	471	0.01%	0.01%	328	466	423	519	*cdsA1*	Signal Transduction	Transcription Regulator
RCAP_rcc01629	0.28	0.0215	1413	1719	0.02%	0.02%	1275	1589	1562	1876	*dxr*	Photosynthesis	Terpenoid backbone biosynthesis
RCAP_rcc01630	0.36	0	2544	3266	0.04%	0.04%	2426	2747	3099	3433	*M50 family peptidase*	Post-translational Modification, Assembly and Chaperones	Peptidase
RCAP_rcc01631	-0.55	0	903	612	0.01%	0.01%	752	1033	563	661	*hypothetical protein*	Unknown	Unknown
RCAP_rcc01632	-0.37	0.0019	15091	11651	0.21%	0.16%	13507	16430	10466	12836	*yaeT*	Cell Envelope Biosynthesis	Cell Wall Biosynthesis
RCAP_rcc01633	-0.06	0.8233	247	236	0.00%	0.00%	193	291	192	280	*outer membrane chaperone Skp family protein*	Cell Envelope Biosynthesis	Cell Wall Biosynthesis
RCAP_rcc01634	-0.39	0.0903	1602	1202	0.02%	0.02%	1181	1975	984	1421	*fabZ*	Lipid Metabolism	Unknown
RCAP_rcc01635	-0.32	0.0231	1224	975	0.02%	0.01%	1081	1330	854	1097	*lpxA*	Glycan Biosynthesis and Metabolism	Lipopolysaccharide biosynthesis

RCAP_rcc01636	-0.35	0.3011	217	166	0.00%	0.00%	132	287	126	206	*hypothetical protein*	Unknown	Unknown
RCAP_rcc01637	-0.64	0	438	279	0.01%	0.00%	385	469	243	316	*lpxB*	Glycan Biosynthesis and Metabolism	Lipopolysaccharide biosynthesis
RCAP_rcc01638	-0.45	0.0002	266	193	0.00%	0.00%	231	292	175	212	*LamB/YcsF family protein*	Unknown	Unknown
RCAP_rcc01639	-0.41	0.0073	91	68	0.00%	0.00%	77	101	59	76	*allophanate hydrolase subunit 2*	Xenobiotics Biodegradation and Metabolism	Atrazine degradation
RCAP_rcc01640	-0.29	0.2315	55	44	0.00%	0.00%	42	65	33	56	*allophanate hydrolase subunit 1*	Xenobiotics Biodegradation and Metabolism	Atrazine degradation
RCAP_rcc01641	-0.12	0.7171	132	120	0.00%	0.00%	91	165	86	154	*transglutaminase-like family protein*	Amino Acid Metabolism	Unknown
RCAP_rcc01642	0.17	0.2027	810	910	0.01%	0.01%	720	890	814	1006	*hypothetical protein*	Unknown	Unknown
RCAP_rcc01643	0.97	0	710	1403	0.01%	0.02%	682	835	1288	1518	*transglutaminase-like family protein*	Amino Acid Metabolism	Unknown
RCAP_rcc01644	-2.03	NA	414	77	0.01%	0.00%	103	689	69	86	*FMN-binding domain-containing protein*	Metabolism of Cofactors, Coenzymes and Vitamins	Unknown
RCAP_rcc01645	-2.11	0	249	47	0.00%	0.00%	103	372	35	58	*lipoprotein*	Predicted Function	Unknown
RCAP_rcc01646	-2.15	NA	609	101	0.01%	0.00%	201	962	74	129	*cytochrome c domain-containing protein*	Energy Metabolism	Aerobic/Anaerobic Respiration
RCAP_rcc01647	-2.43	NA	564	75	0.01%	0.00%	205	872	54	97	*ABC transporter periplasmic substrate-binding protein*	Metal and Ion Transport	Unknown
RCAP_rcc01648	-2.2	NA	227	38	0.00%	0.00%	76	360	29	47	*ABC transporter permease*	Metal and Ion Transport	Unknown
RCAP_rcc01649	-1.37	NA	59	18	0.00%	0.00%	19	94	14	23	*ABC transporter ATP-binding protein*	Metal and Ion Transport	Unknown
RCAP_rcc01650	-2.06	NA	711	128	0.01%	0.00%	142	1219	109	148	*ABC transporter permease*	Unknown	Unknown
RCAP_rcc01651	-2.27	NA	222	24	0.00%	0.00%	55	367	16	32	*ABC transporter ATP-binding protein*	Unknown	Unknown
RCAP_rcc01652	-2.77	NA	750	71	0.01%	0.00%	174	1255	50	92	*hypothetical protein*	Unknown	Unknown
RCAP_rcc01653	-2.67	NA	1240	129	0.02%	0.00%	272	2091	96	161	*YVTN beta-propeller repeat family protein*	Unknown	Unknown
RCAP_rcc01654	-2.56	NA	435	45	0.01%	0.00%	98	729	36	55	*hypothetical protein*	Unknown	Unknown
RCAP_rcc01655	-2.63	NA	330	33	0.00%	0.00%	58	570	26	40	*hypothetical protein*	Unknown	Unknown
RCAP_rcc01656	-1.81	NA	395	84	0.01%	0.00%	121	634	62	106	*cycA2*	Energy Metabolism	Aerobic/Anaerobic Respiration
RCAP_rcc01657	-2.31	NA	6999	1044	0.09%	0.01%	2122	11273	798	1290	*exaA2*	Carbohydrate Metabolism	Glycolysis / Gluconeogenesis
RCAP_rcc01658	-1.28	NA	357	132	0.00%	0.00%	151	539	117	147	*two component LuxR family transcriptional regulator*	Signal Transduction	Transcription Regulator
RCAP_rcc01659	-0.96	0	313	157	0.00%	0.00%	244	364	134	181	*hypothetical protein*	Unknown	Unknown
RCAP_rcc01660	-1.05	0	928	440	0.01%	0.01%	718	1081	400	481	*signal transduction histidine kinase*	Signal Transduction	Kinase/Phosphorelay
RCAP_rcc01661	0.29	0.0239	1269	1561	0.02%	0.02%	1206	1454	1382	1739	*trmU*	Unknown	Unknown
RCAP_rcc01662	-0.52	0.0069	2110	1450	0.03%	0.02%	1698	2495	1211	1689	*hypothetical protein*	Unknown	Unknown
RCAP_rcc01663	-0.49	0.011	16083	11331	0.22%	0.15%	12790	19089	9640	13023	*ctrA*	Signal Transduction	Transcription Regulator
RCAP_rcc01664	-0.43	0.0001	2266	1671	0.03%	0.02%	1993	2476	1545	1798	*ligA*	Replication, Recombination and Repair	Unknown
RCAP_rcc01665	-0.42	0.0002	555	413	0.01%	0.01%	485	605	377	448	*recG*	Metabolism of Cofactors, Coenzymes and Vitamins	Folate biosynthesis
RCAP_rcc01666	0.84	0.0023	515	967	0.01%	0.01%	387	686	634	1300	*hypothetical protein*	Unknown	Unknown
RCAP_rcc01667	-0.45	0.0073	655	475	0.01%	0.01%	530	750	426	524	*methyl-accepting chemotaxis sensory transducer*	Motility	Chemotaxis
RCAP_rcc01668	-0.28	NA	52	42	0.00%	0.00%	30	72	34	50	*hypothetical protein*	Unknown	Unknown
RCAP_rcc01669	0.15	0.2992	568	633	0.01%	0.01%	507	641	564	702	*hisI*	Amino Acid Metabolism	Histidine metabolism
RCAP_rcc01670	0.43	0.0028	95	127	0.00%	0.00%	86	102	108	147	*hypothetical protein*	Unknown	Unknown
RCAP_rcc01671	-0.23	0.1862	167	141	0.00%	0.00%	141	186	114	168	*carbohydrate kinase*	Carbohydrate Metabolism	Unknown
RCAP_rcc01672	-0.79	0	106	60	0.00%	0.00%	86	118	47	73	*YjeF-related family protein*	Unknown	Unknown
RCAP_rcc01673	0.2	0.2973	4030	4642	0.06%	0.06%	3441	4831	3886	5398	*glnB1*	Signal Transduction	Transcription Regulator
RCAP_rcc01674	0.06	0.6868	31065	32383	0.44%	0.44%	28089	34870	28998	35767	*glnA3*	Carbohydrate Metabolism	Glyoxylate and dicarboxylate metabolism
RCAP_rcc01675	0.49	0.004	768	1085	0.01%	0.01%	619	933	982	1188	*fabD*	Lipid Metabolism	Fatty acid biosynthesis
RCAP_rcc01676	0.54	0	1406	2049	0.02%	0.03%	1313	1592	1901	2196	*fabG*	Lipid Metabolism	Biotin metabolism
RCAP_rcc01677	-0.16	0.5076	640	571	0.01%	0.01%	504	777	459	683	*hypothetical protein*	Unknown	Unknown
RCAP_rcc01678	-0.42	0.0351	11491	8501	0.16%	0.12%	9095	13661	7097	9904	*acpP1*	Lipid Metabolism	Unknown
RCAP_rcc01679	0.58	0.004	69	105	0.00%	0.00%	56	82	86	125	*ligT*	Unknown	Unknown
RCAP_rcc01680	0.4	0.0001	2734	3609	0.04%	0.05%	2542	3049	3375	3844	*fabF1*	Lipid Metabolism	Biotin metabolism
RCAP_rcc01681	-0.22	0.0707	2838	2427	0.04%	0.03%	2507	3135	2236	2619	*aminodeoxychorismate lyase*	Unknown	Unknown
RCAP_rcc01682	-0.71	0.0019	45	27	0.00%	0.00%	34	54	22	32	*hypothetical protein*	Unknown	Unknown
RCAP_rcc01683	-0.2	0.42	109	94	0.00%	0.00%	90	125	68	119	*terminase-like family protein*	Unknown	Unknown
RCAP_rcc01684	-1.23	0	33	13	0.00%	0.00%	24	40	11	15	*HK97 family phage portal protein*	Replication, Recombination and Repair	Phage Interaction
RCAP_rcc01685	-2.05	0	5	1	0.00%	0.00%	3	6	0	1	*hypothetical protein*	Unknown	Unknown
RCAP_rcc01686	-1.07	0.0008	8	4	0.00%	0.00%	6	10	2	5	*phage prohead protease*	Replication, Recombination and Repair	Phage Interaction
RCAP_rcc01687	-1.59	0	168	54	0.00%	0.00%	133	194	48	60	*HK97 family phage major capsid protein*	Replication, Recombination and Repair	Phage Interaction
RCAP_rcc01688	-1.52	0	10	2	0.00%	0.00%	6	12	1	4	*hypothetical protein*	Unknown	Unknown
RCAP_rcc01689	0.2	0.7531	0	0	0.00%	0.00%	0	1	0	1	*hypothetical protein*	Unknown	Unknown
RCAP_rcc01690	-0.31	0.4159	4	3	0.00%	0.00%	2	5	2	4	*hypothetical protein*	Unknown	Unknown
RCAP_rcc01691	-1.69	0	19	5	0.00%	0.00%	12	25	4	6	*TP901-1 family phage major tail protein*	Replication, Recombination and Repair	Phage Interaction
RCAP_rcc01692	-0.54	0.3403	1	0	0.00%	0.00%	0	2	0	1	*hypothetical protein*	Unknown	Unknown
RCAP_rcc01693	0.13	0.7358	0	0	0.00%	0.00%	0	0	0	0	*hypothetical protein*	Unknown	Unknown
RCAP_rcc01694	-0.81	0.0094	5	3	0.00%	0.00%	4	6	2	3	*hypothetical protein*	Unknown	Unknown
RCAP_rcc01695	-0.79	0.0048	13	7	0.00%	0.00%	9	16	5	8	*hypothetical protein*	Unknown	Unknown
RCAP_rcc01696	-0.36	0.2093	14	10	0.00%	0.00%	11	17	7	13	*hypothetical protein*	Unknown	Unknown
RCAP_rcc01697	-0.48	0.2962	3	2	0.00%	0.00%	2	4	1	3	*NlpC/P60 family phage cell wall peptidase*	Replication, Recombination and Repair	Phage Interaction
RCAP_rcc01698	-0.24	0.135	90	75	0.00%	0.00%	77	102	65	85	*hypothetical protein*	Unknown	Unknown
RCAP_rcc01699	-0.55	0.1441	5	3	0.00%	0.00%	3	7	2	4	*hypothetical protein*	Unknown	Unknown
RCAP_rcc01700	0.1	0.4337	1724	1847	0.02%	0.03%	1571	1927	1674	2020	*cysE1*	Energy Metabolism	Sulfur metabolism
RCAP_rcc01701	0.14	0.4814	179	197	0.00%	0.00%	153	204	159	235	*phospholipid/glycerol acyltransferase*	Lipid Metabolism	Glycerophospholipid metabolism
RCAP_rcc01702	-0.37	0.0195	294	226	0.00%	0.00%	244	335	206	246	*cdsA2*	Signal Transduction	Transcription Regulator
RCAP_rcc01703	-1.45	0	12805	4561	0.17%	0.06%	10470	14336	3682	5439	*pdhC*	Carbohydrate Metabolism	TCA Cycle
RCAP_rcc01704	-1.42	0	13239	4789	0.18%	0.07%	10391	15195	4051	5528	*pdhB*	Carbohydrate Metabolism	TCA Cycle
RCAP_rcc01705	-1.13	0	6260	2767	0.09%	0.04%	4750	7403	2325	3209	*pdhA*	Carbohydrate Metabolism	TCA Cycle
RCAP_rcc01706	-0.17	0.4311	1260	1113	0.02%	0.02%	1026	1492	941	1286	*septum formation initiator*	Unknown	Unknown
RCAP_rcc01707	-0.02	0.9403	6296	6225	0.09%	0.08%	5414	7325	5418	7031	*fda*	Carbohydrate Metabolism	Glycolysis / Gluconeogenesis
RCAP_rcc01708	0.36	0.0028	6070	7804	0.09%	0.11%	5464	6840	7104	8505	*pgk*	Carbohydrate Metabolism	Glycolysis / Gluconeogenesis
RCAP_rcc01709	1.43	0	2424	6614	0.04%	0.09%	2635	2953	5942	7286	*ppiA*	Post-translational Modification, Assembly and Chaperones	Unknown
RCAP_rcc01710	0.77	0	2453	4202	0.04%	0.06%	2285	2910	3886	4518	*ppiB*	Post-translational Modification, Assembly and Chaperones	Unknown
RCAP_rcc01711	-0.16	0.4361	5120	4552	0.07%	0.06%	4087	6093	3902	5203	*tyrS*	Translation, ribosomal structure and biogenesis	Aminoacyl-tRNA biosynthesis
RCAP_rcc01712	0.04	0.9109	259	266	0.00%	0.00%	182	325	200	331	*anmK*	Cell Envelope Biosynthesis	Cell Wall Biosynthesis

RCAP_rcc01713	-0.6	0.0011	1500	974	0.02%	0.01%	1255	1716	791	1157	*lipoprotein*	Predicted Function	Unknown
RCAP_rcc01714	-0.26	0.3621	234	192	0.00%	0.00%	172	292	145	239	*hypothetical protein*	Unknown	Unknown
RCAP_rcc01715	0.03	0.8307	8953	9153	0.13%	0.12%	7800	10178	8584	9721	*eno*	Carbohydrate Metabolism	Glycolysis / Gluconeogenesis
RCAP_rcc01716	-0.35	0.001	1075	842	0.01%	0.01%	947	1185	793	891	*hypothetical protein*	Unknown	Unknown
RCAP_rcc01717	0.21	0.5129	1070	1253	0.01%	0.02%	756	1376	804	1702	*hypothetical protein*	Unknown	Unknown
RCAP_rcc01718	0.19	0.0627	692	789	0.01%	0.01%	666	755	714	864	*rihA*	Metabolism of Cofactors, Coenzymes and Vitamins	Nicotinate and nicotinamide metabolism
RCAP_rcc01719	0.12	0.35	1045	1138	0.01%	0.02%	964	1119	990	1287	*ABC transporter ATP-binding protein*	Unknown	Unknown
RCAP_rcc01720	0.24	0.231	5667	6752	0.08%	0.09%	4774	6770	5685	7820	*ndk*	Nucleotide Metabolism	Pyrimidine metabolism
RCAP_rcc01721	-0.64	0.0392	318	195	0.00%	0.00%	216	401	123	266	*TfoX domain-containing protein*	Unknown	Unknown
RCAP_rcc01722	0.24	0.1164	495	587	0.01%	0.01%	420	578	545	629	*BadM/Rrf2 family transcriptional regulator*	Signal Transduction	Transcription Regulator
RCAP_rcc01723	0.97	0.0013	3541	7419	0.05%	0.10%	2502	5086	5580	9259	*ccpA*	Energy Metabolism	Aerobic/Anaerobic Respiration
RCAP_rcc01724	-0.2	0.2577	762	660	0.01%	0.01%	628	879	570	751	*speB1*	Amino Acid Metabolism	Arginine and proline metabolism
RCAP_rcc01725	0.9	0	4211	7961	0.06%	0.11%	3925	5276	6956	8966	*hypothetical protein*	Unknown	Unknown
RCAP_rcc01726	-0.38	0.0203	2598	1980	0.04%	0.03%	2102	3057	1824	2136	*mcpH*	Motility	Chemotaxis
RCAP_rcc01727	-0.16	0.2158	912	813	0.01%	0.01%	792	1007	731	894	*pyrD1*	Nucleotide Metabolism	Unknown
RCAP_rcc01728	2.12	0	343	1608	0.01%	0.02%	304	433	1150	2066	*nifJ*	Energy Metabolism	Nitrogen metabolism
RCAP_rcc01729	1.61	0	609	2106	0.01%	0.03%	437	852	1547	2665	*pyridine nucleotide-disulfide oxidoreductase*	Sulfur Metabolism	Unknown
RCAP_rcc01730	1.25	0	1692	4045	0.03%	0.05%	1713	1949	3768	4322	*U32 family peptidase*	Post-translational Modification, Assembly and Chaperones	Peptidase
RCAP_rcc01731	1.23	0	1618	3834	0.02%	0.05%	1652	1886	3539	4128	*U32 family peptidase*	Post-translational Modification, Assembly and Chaperones	Peptidase
RCAP_rcc01732	1.69	0	568	1913	0.01%	0.03%	554	792	1689	2138	*hypothetical protein*	Unknown	Unknown
RCAP_rcc01733	0.97	0	848	1669	0.01%	0.02%	821	948	1502	1836	*ubiD*	Photosynthesis	Biosynthesis of Ubiquinone
RCAP_rcc01734	1.08	0	148	324	0.00%	0.00%	126	186	288	359	*ubiX*	Photosynthesis	Biosynthesis of Ubiquinone
RCAP_rcc01735	0.7	0	730	1193	0.01%	0.02%	674	838	1098	1288	*NnrS family protein*	Energy Metabolism	Nitrogen Metabolism
RCAP_rcc01736	-0.03	0.8473	452	442	0.01%	0.01%	390	502	393	492	*acyltransferase*	Unknown	Unknown
RCAP_rcc01737	0.72	0.0533	77	136	0.00%	0.00%	52	104	81	192	*oxyR*	Transcription	Unknown
RCAP_rcc01738	-1.6	0	9760	2937	0.13%	0.04%	7218	11600	1714	4161	*katG*	Energy Metabolism	Unknown
RCAP_rcc01739	0.57	0.0003	1704	2560	0.02%	0.03%	1554	1982	2168	2951	*hypothetical protein*	Unknown	Unknown
RCAP_rcc01740	-0.19	0.1949	1574	1379	0.02%	0.02%	1350	1744	1245	1514	*ABC transporter ATP-binding protein*	Unknown	Unknown
RCAP_rcc01741	0.02	0.8663	521	530	0.01%	0.01%	474	580	497	562	*hypothetical protein*	Unknown	Unknown
RCAP_rcc01742	0.47	0.0226	142	199	0.00%	0.00%	122	169	158	240	*mcr*	Lipid Metabolism	Primary bile acid biosynthesis
RCAP_rcc01743	0	0.9747	2229	2234	0.03%	0.03%	2100	2397	2114	2354	*DSBA family oxidoreductase*	Post-translational Modification, Assembly and Chaperones	Unknown
RCAP_rcc01744	0.61	0.0026	164	255	0.00%	0.00%	129	207	224	287	*class I aminotransferase*	Amino Acid Metabolism	Valine, leucine and isoleucine biosynthesis
RCAP_rcc01745	0.03	0.9066	436	444	0.01%	0.01%	359	509	389	499	*amiC*	Cell Envelope Biosynthesis	Cell Wall Biosynthesis
RCAP_rcc01746	-0.33	0.0797	88	70	0.00%	0.00%	75	99	58	81	*hypothetical protein*	Unknown	Unknown
RCAP_rcc01747	0.19	0.2754	394	452	0.01%	0.01%	346	452	382	521	*actP1*	Amino Acid Metabolism	Unknown
RCAP_rcc01748	0.07	0.7965	319	336	0.00%	0.00%	240	382	258	413	*rsmB2*	Translation, ribosomal structure and biogenesis	Unknown
RCAP_rcc01749	-0.42	0	4271	3181	0.06%	0.04%	3988	4427	3022	3341	*signal transduction histidine kinase*	Signal Transduction	Kinase/Phosphorelay
RCAP_rcc01750	0.31	0.0076	141	176	0.00%	0.00%	127	157	161	191	*hypothetical protein*	Unknown	Unknown
RCAP_rcc01751	0.04	0.7358	5208	5373	0.07%	0.07%	4710	5695	4830	5916	*recA*	Replication, Recombination and Repair	Recombination
RCAP_rcc01752	-0.11	0.498	7758	7192	0.11%	0.10%	6954	8609	6324	8061	*alaS*	Translation, ribosomal structure and biogenesis	Aminoacyl-tRNA biosynthesis
RCAP_rcc01753	-0.64	0.0004	819	516	0.01%	0.01%	671	936	438	595	*hypothetical protein*	Unknown	Unknown
RCAP_rcc01754	0.06	0.66	2492	2608	0.04%	0.04%	2204	2817	2397	2819	*class II glutamine amidotransferase*	Unknown	Unknown
RCAP_rcc01755	-0.22	0.1764	2110	1803	0.03%	0.02%	1766	2429	1599	2007	*cysS*	Translation, ribosomal structure and biogenesis	Aminoacyl-tRNA biosynthesis
RCAP_rcc01756	-0.26	0.0146	1993	1663	0.03%	0.02%	1829	2147	1511	1815	*2-isopropylmalate synthase/homocitrate synthase*	Amino Acid Metabolism	Valine, leucine and isoleucine biosynthesis
RCAP_rcc01757	-0.38	0.3076	83	61	0.00%	0.00%	44	117	34	88	*phytoene synthase*	Unknown	Unknown
RCAP_rcc01758	-0.26	0.0118	1848	1542	0.03%	0.02%	1648	2027	1447	1637	*mcpA2*	Motility	Chemotaxis
RCAP_rcc01759	-0.3	0.0039	784	636	0.01%	0.01%	704	847	587	685	*cheB2*	Signal Transduction	Transcription Regulator
RCAP_rcc01760	-0.34	0.0024	443	349	0.01%	0.00%	394	490	320	378	*cheD*	Motility	Chemotaxis
RCAP_rcc01761	-0.65	0	1951	1238	0.03%	0.02%	1739	2110	1081	1395	*hypothetical protein*	Unknown	Unknown
RCAP_rcc01762	-0.75	0	1703	1006	0.02%	0.01%	1502	1857	868	1143	*cheY2*	Motility	Chemotaxis
RCAP_rcc01763	-0.82	0	4485	2511	0.06%	0.03%	3873	4945	2172	2851	*cheR3*	Motility	Chemotaxis
RCAP_rcc01764	-0.61	0	988	645	0.01%	0.01%	849	1109	582	708	*cheW2*	Motility	Chemotaxis
RCAP_rcc01765	-0.52	0	8191	5700	0.11%	0.08%	7329	8846	5419	5981	*cheA2*	Motility	Chemotaxis
RCAP_rcc01766	-0.32	0.0028	1293	1033	0.02%	0.01%	1136	1435	977	1089	*cheY3*	Motility	Chemotaxis
RCAP_rcc01767	-0.17	0.2077	1353	1202	0.02%	0.02%	1215	1519	1078	1325	*cheX*	Motility	Chemotaxis
RCAP_rcc01768	-0.84	0	795	436	0.01%	0.01%	604	937	377	496	*glk*	Carbohydrate Metabolism	Glycolysis / Gluconeogenesis
RCAP_rcc01769	-1	0	2191	1083	0.03%	0.01%	1775	2498	989	1177	*bglA*	Energy Metabolism	Phenylpropanoid biosynthesis
RCAP_rcc01770	-0.4	0.0185	618	467	0.01%	0.01%	483	721	422	512	*LacI family transcriptional regulator*	Signal Transduction	Transcription Regulator
RCAP_rcc01771	-0.62	0.0015	16764	10763	0.23%	0.15%	13837	19153	8827	12700	*aglE*	Carbohydrate Metabolism	Unknown
RCAP_rcc01772	-0.99	0	4838	2375	0.07%	0.03%	3919	5521	1993	2757	*aglF*	Metal and Ion Transport	Unknown
RCAP_rcc01773	-1.12	0	5535	2468	0.08%	0.03%	4438	6343	1992	2943	*aglG*	Metal and Ion Transport	Unknown
RCAP_rcc01774	-1.05	0	9787	4598	0.13%	0.06%	8008	11143	3742	5454	*aglA*	Carbohydrate Metabolism	Galactose Metabolism
RCAP_rcc01775	-1.28	0	7558	3008	0.10%	0.04%	6047	8664	2509	3508	*aglK*	Amino Acid Metabolism	Amino Acid Transport
RCAP_rcc01776	-0.69	0	11102	6817	0.15%	0.09%	9584	12295	5802	7832	*trimethylamine methyltransferase*	Unknown	Unknown
RCAP_rcc01777	-0.33	0.1093	689	544	0.01%	0.01%	518	843	506	582	*dusA*	Translation, ribosomal structure and biogenesis	Unknown
RCAP_rcc01778	0.14	0.4293	123	136	0.00%	0.00%	108	136	114	157	*hypothetical protein*	Unknown	Unknown
RCAP_rcc01779	0.16	0.0772	509	568	0.01%	0.01%	467	545	531	605	*MazG family protein*	Nucleotide Metabolism	Pyrimidine metabolism
RCAP_rcc01780	0.66	0	2441	3861	0.04%	0.05%	2397	2735	3593	4129	*M20 family peptidase*	Post-translational Modification, Assembly and Chaperones	Peptidase
RCAP_rcc01781	-0.42	0.042	5222	3866	0.07%	0.05%	4159	6289	3238	4495	*yajC*	Trafficking and Secretion	Trafficing
RCAP_rcc01782	-0.21	0.0252	5714	4948	0.08%	0.07%	5326	6041	4557	5338	*secD*	Trafficking and Secretion	Secretion
RCAP_rcc01783	-0.52	0.0022	5792	3992	0.08%	0.05%	4817	6629	3457	4527	*secF*	Trafficking and Secretion	Secretion
RCAP_rcc01784	-0.32	0.0674	300	239	0.00%	0.00%	248	334	202	275	*hypothetical protein*	Unknown	Unknown
RCAP_rcc01785	-0.35	0.1368	304	235	0.00%	0.00%	229	362	191	279	*ccmA*	Energy Metabolism	Cytochrome Biogenesis
RCAP_rcc01786	-0.61	0	597	389	0.01%	0.01%	527	637	354	424	*ccmB*	Energy Metabolism	Cytochrome Biogenesis
RCAP_rcc01787	-0.05	0.7599	1830	1766	0.03%	0.02%	1564	2100	1614	1919	*ccmC*	Energy Metabolism	Cytochrome Biogenesis
RCAP_rcc01788	0.52	0.0097	82	120	0.00%	0.00%	69	100	104	135	*ccmD*	Energy Metabolism	Cytochrome Biogenesis
RCAP_rcc01789	-0.02	0.7784	1067	1050	0.01%	0.01%	1002	1125	995	1105	*ccmG*	Energy Metabolism	Cytochrome Biogenesis

RCAP_rcc01790	-0.17	0.4534	1171	1038	0.02%	0.01%	919	1416	884	1191	*hpt*	Xenobiotics Biodegradation and Metabolism	Drug metabolism - other enzymes
RCAP_rcc01791	-0.19	0.3851	227	198	0.00%	0.00%	176	274	172	224	*cyclase/dehydrase*	Lipid Metabolism	Unknown
RCAP_rcc01792	0.08	0.7389	246	260	0.00%	0.00%	199	300	224	296	*amt*	Metal and Ion Transport	Unknown
RCAP_rcc01793	-0.02	0.832	222	218	0.00%	0.00%	202	237	204	232	*cinA domain-containing protein*	Metabolism of Cofactors, Coenzymes and Vitamins	Nicotinate and nicotinamide metabolism
RCAP_rcc01794	0.65	0.0023	255	408	0.00%	0.01%	201	314	363	452	*pgpA*	Lipid Metabolism	Glycerophospholipid metabolism
RCAP_rcc01795	0.7	0	1615	2631	0.02%	0.04%	1563	1790	2511	2751	*ispDF*	Lipid	Terpenoid backbone biosynthesis
RCAP_rcc01796	1.68	0	203	703	0.00%	0.01%	173	281	543	862	*dusB*	Translation, ribosomal structure and biogenesis	Unknown
RCAP_rcc01797	0.36	0.0014	367	470	0.01%	0.01%	331	407	431	510	*ntrB*	Signal Transduction	Kinase/Phosphorelay
RCAP_rcc01798	0.13	0.1194	608	664	0.01%	0.01%	570	641	616	712	*ntrC*	Signal Transduction	Transcription Regulator
RCAP_rcc01799	0.36	0	989	1265	0.01%	0.02%	927	1078	1226	1304	*ntrY*	Signal Transduction	Kinase/Phosphorelay
RCAP_rcc01800	-0.02	0.8907	1274	1256	0.02%	0.02%	1140	1432	1147	1364	*ntrX*	Signal Transduction	Transcription Regulator
RCAP_rcc01801	-0.04	0.8869	3525	3435	0.05%	0.05%	2765	4304	2917	3953	*hfq*	Signal Transduction	Kinase/Phosphorelay
RCAP_rcc01802	-0.26	0.0173	3157	2632	0.04%	0.04%	2888	3326	2331	2932	*hflX*	Unknown	Unknown
RCAP_rcc01803	-0.07	0.6695	824	784	0.01%	0.01%	725	927	701	867	*quiP*	Unknown	Unknown
RCAP_rcc01804	-0.02	0.9064	296	291	0.00%	0.00%	248	335	262	320	*garR*	Carbohydrate Metabolism	Glyoxylate and dicarboxylate metabolism
RCAP_rcc01805	-0.47	0	1103	794	0.02%	0.01%	1007	1184	717	871	*ssb1*	Replication, Recombination and Repair	Unknown
RCAP_rcc01806	-0.4	0.1201	643	479	0.01%	0.01%	437	822	399	558	*transglycosylase, Slt family*	Cell Envelope Biosynthesis	Cell Wall Biosynthesis
RCAP_rcc01807	0.14	0.331	1281	1411	0.02%	0.02%	1110	1482	1328	1493	*hypothetical protein*	Unknown	Unknown
RCAP_rcc01808	0.42	0.004	2015	2703	0.03%	0.04%	1812	2301	2367	3038	*hemB*	Metabolism of Cofactors, Coenzymes and Vitamins	Heme Biosynthesis
RCAP_rcc01809	0.44	0	365	497	0.01%	0.01%	338	396	449	544	*hypothetical protein*	Unknown	Unknown
RCAP_rcc01810	-0.76	0	4689	2739	0.06%	0.04%	3913	5237	2395	3083	*mfd*	Replication, Recombination and Repair	Repair
RCAP_rcc01811	-0.45	0	1335	974	0.02%	0.01%	1213	1427	887	1062	*major facilitator superfamily protein*	Metal and Ion Transport	Unknown
RCAP_rcc01812	-0.13	0.2911	492	448	0.01%	0.01%	449	532	399	498	*DSBA family oxidoreductase*	Secondary metabolites biosynthesis, transport, and catabolism	Unknown
RCAP_rcc01813	0.11	0.4371	1029	1114	0.01%	0.02%	911	1145	987	1240	*AMP-dependent synthetase and ligase*	Energy Metabolism	Reductive carboxylate cycle (CO2 fixation)
RCAP_rcc01814	-0.02	0.9284	1102	1089	0.02%	0.01%	933	1296	949	1228	*hypothetical protein*	Unknown	Unknown
RCAP_rcc01815	0.03	0.8554	117	120	0.00%	0.00%	104	129	110	129	*ecnB*	Unknown	Unknown
RCAP_rcc01816	-0.28	0.1527	1372	1126	0.02%	0.02%	1097	1631	962	1290	*family 5 extracellular solute-binding protein*	Amino Acid Metabolism	Amino Acid Transport
RCAP_rcc01817	0.12	0.2304	994	1079	0.01%	0.01%	921	1094	1012	1146	*bdhA*	Carbohydrate Metabolism	Butanoate metabolism
RCAP_rcc01818	0.55	0	896	1312	0.01%	0.02%	900	1001	1211	1413	*hypothetical protein*	Unknown	Unknown
RCAP_rcc01819	-0.02	0.9681	266	262	0.00%	0.00%	141	375	169	355	*indigoidine synthase A like protein family*	Secondary metabolites biosynthesis, transport, and catabolism	Unknown
RCAP_rcc01820	0.22	0.4882	230	272	0.00%	0.00%	142	310	192	352	*carbohydrate/purine kinase*	Signal Transduction	Kinase/Phosphorelay
RCAP_rcc01821	-0.24	0.2732	11431	9599	0.16%	0.13%	9201	13544	7946	11251	*rpsB*	Translation, ribosomal structure and biogenesis	Unknown
RCAP_rcc01822	-0.31	0.1105	8412	6749	0.12%	0.09%	7168	9558	5607	7890	*tsf*	Translation, ribosomal structure and biogenesis	Unknown
RCAP_rcc01823	-0.47	0.0001	1520	1096	0.02%	0.01%	1289	1701	1024	1168	*LuxR family autoinducer-binding transcriptional regulator*	Signal Transduction	Transcription Regulator
RCAP_rcc01824	0.06	0.6695	1151	1201	0.02%	0.02%	1001	1309	1122	1280	*hypothetical protein*	Unknown	Unknown
RCAP_rcc01825	1.57	0	363	1100	0.01%	0.01%	384	446	952	1248	*HAD superfamily hydrolase*	Unknown	Unknown
RCAP_rcc01826	1.43	0	583	1622	0.01%	0.02%	560	737	1320	1924	*gph3*	Carbohydrate Metabolism	Glyoxylate and dicarboxylate metabolism
RCAP_rcc01827	1.55	0	541	1602	0.01%	0.02%	574	660	1423	1781	*AhpC/TSA family protein*	Post-translational Modification, Assembly and Chaperones	Unknown
RCAP_rcc01828	1.67	0	1095	3526	0.02%	0.05%	1154	1385	3183	3869	*rpe1*	Carbohydrate Metabolism	Pentose and glucuronate interconversions
RCAP_rcc01829	2.75	0	3190	22633	0.07%	0.31%	4372	5248	19002	26264	*cbbM*	Energy Metabolism	Carbon fixation in photosynthetic organisms
RCAP_rcc01830	2.82	0	2249	16797	0.05%	0.23%	3139	3700	13773	19821	*fba*	Carbohydrate Metabolism	Glycolysis / Gluconeogenesis
RCAP_rcc01831	2.64	0	1368	9082	0.03%	0.12%	1833	2226	7366	10797	*gap1*	Carbohydrate Metabolism	Glycolysis / Gluconeogenesis
RCAP_rcc01832	2.19	0	1298	6363	0.02%	0.09%	1460	2055	5331	7396	*tkt1*	Carbohydrate Metabolism	Pentose phosphate pathway
RCAP_rcc01833	2.14	0	1596	7586	0.03%	0.10%	1810	2404	6025	9146	*cbbP*	Energy Metabolism	Carbon fixation in photosynthetic organisms
RCAP_rcc01834	1.28	0	1289	3306	0.02%	0.04%	1120	1725	2605	4006	*fbp*	Carbohydrate Metabolism	Glycolysis / Gluconeogenesis
RCAP_rcc01835	0.76	0	189	324	0.00%	0.00%	165	222	277	371	*cbbR2*	Signal Transduction	Transcription Regulator
RCAP_rcc01836	-0.58	0	3179	2115	0.04%	0.03%	2699	3560	1888	2342	*qor*	Energy Metabolism	Unknown
RCAP_rcc01837	-0.02	0.9319	5937	5864	0.08%	0.08%	5101	6843	5085	6643	*pgm*	Carbohydrate Metabolism	Glycolysis / Gluconeogenesis
RCAP_rcc01838	-0.55	0.0014	2900	1961	0.04%	0.03%	2361	3325	1689	2233	*glgX*	Glycan Biosynthesis and Metabolism	Glycosaminoglycan degradation
RCAP_rcc01839	-0.27	0.0186	3239	2675	0.05%	0.04%	2934	3528	2406	2944	*glgA*	Carbohydrate Metabolism	Starch and sucrose metabolism
RCAP_rcc01840	-0.36	0.0492	5270	4082	0.07%	0.06%	4340	6154	3493	4671	*glgC*	Carbohydrate Metabolism	Amino sugar and nucleotide sugar metabolism
RCAP_rcc01841	-0.4	0.0111	5939	4463	0.08%	0.06%	5049	6719	3851	5076	*glgB*	Carbohydrate Metabolism	Starch and sucrose metabolism
RCAP_rcc01842	-0.26	0.119	10212	8490	0.14%	0.12%	8601	11729	7336	9643	*glgP*	Carbohydrate Metabolism	Starch and sucrose metabolism
RCAP_rcc01843	0.47	0.0001	2059	2859	0.03%	0.04%	1848	2355	2593	3124	*family 13 glycosyl hydrolase*	Carbohydrate Metabolism	Galactose Metabolism
RCAP_rcc01844	0.34	0.004	416	527	0.01%	0.01%	383	443	465	590	*sensor histidine kinase*	Signal Transduction	Kinase/Phosphorelay
RCAP_rcc01845	-0.31	0.0591	557	445	0.01%	0.01%	439	652	401	490	*hypothetical protein*	Unknown	Unknown
RCAP_rcc01846	-0.15	0.2232	440	396	0.01%	0.01%	386	485	360	431	*transferase hexapeptide repeat family protein*	Unknown	Unknown
RCAP_rcc01847	0.24	0.0395	350	416	0.00%	0.01%	314	393	386	446	*gmk*	Nucleotide Metabolism	Purine metabolism
RCAP_rcc01848	0.73	0	190	316	0.00%	0.00%	165	224	297	335	*yicC*	Unknown	Unknown
RCAP_rcc01849	-0.02	0.9445	2941	2898	0.04%	0.04%	2167	3724	2481	3314	*hypothetical protein*	Unknown	Unknown
RCAP_rcc01850	-0.44	0.0079	3941	2877	0.05%	0.04%	3338	4472	2521	3232	*aroF*	Amino Acid Metabolism	Phenylalanine, tyrosine and tryptophan biosynthesis
RCAP_rcc01851	-0.44	0.0031	1803	1321	0.02%	0.02%	1489	2046	1190	1453	*AraC family transcriptional regulator*	Signal Transduction	Transcription Regulator
RCAP_rcc01852	0.28	0.0385	14483	17650	0.21%	0.24%	12864	16669	15931	19369	*livK1*	Amino Acid Metabolism	Amino Acid Transport
RCAP_rcc01853	-0.68	0.0026	1742	1062	0.02%	0.01%	1348	2047	831	1293	*livG1*	Amino Acid Metabolism	Amino Acid Transport
RCAP_rcc01854	-0.49	0.0026	459	323	0.01%	0.00%	405	491	267	380	*livF1*	Amino Acid Metabolism	Amino Acid Transport
RCAP_rcc01855	-0.29	0.1141	164	133	0.00%	0.00%	142	183	109	158	*hypothetical protein*	Unknown	Unknown
RCAP_rcc01856	-0.2	0.2453	670	582	0.01%	0.01%	568	752	492	673	*livH1*	Amino Acid Metabolism	Amino Acid Transport
RCAP_rcc01857	-0.13	0.4687	1281	1172	0.02%	0.02%	1097	1435	997	1347	*livM1*	Amino Acid Metabolism	Amino Acid Transport
RCAP_rcc01858	-0.74	0.0003	8398	4925	0.12%	0.07%	6677	9765	4029	5820	*guaA1*	Xenobiotics Biodegradation and Metabolism	Drug metabolism - other enzymes
RCAP_rcc01859	-0.29	0.0134	1381	1128	0.02%	0.02%	1234	1505	1025	1231	*aroB*	Amino Acid Metabolism	Phenylalanine, tyrosine and tryptophan biosynthesis
RCAP_rcc01860	0.29	0.2638	156	193	0.00%	0.00%	116	199	164	221	*aroK*	Amino Acid Metabolism	Phenylalanine, tyrosine and tryptophan biosynthesis
RCAP_rcc01861	0.3	0.001	696	855	0.01%	0.01%	674	737	771	940	*hypothetical protein*	Unknown	Unknown
RCAP_rcc01862	0.1	0.7002	254	273	0.00%	0.00%	191	310	235	311	*xerD*	Replication, Recombination and Repair	Recombination
RCAP_rcc01863	-0.38	0.0002	1465	1124	0.02%	0.02%	1305	1589	1049	1199	*hypothetical protein*	Unknown	Unknown
RCAP_rcc01864	-0.25	0.0378	1391	1169	0.02%	0.02%	1235	1530	1058	1279	*lipA*	Metabolism of Cofactors, Coenzymes and Vitamins	Unknown
RCAP_rcc01865	-0.68	0.0025	40	24	0.00%	0.00%	34	44	17	31	*hypothetical protein*	Unknown	Unknown
RCAP_rcc01866	-0.19	0.5363	19	16	0.00%	0.00%	14	22	11	21	*hypothetical protein*	Unknown	Unknown

RCAP_rcc01867	0.29	0.0018	278	342	0.00%	0.00%	262	304	318	367	*hypothetical protein*	Unknown	Unknown
RCAP_rcc01868	0.26	0.4398	8	10	0.00%	0.00%	5	11	8	12	*hypothetical protein*	Unknown	Unknown
RCAP_rcc01869	0.12	0.394	2312	2510	0.03%	0.03%	1985	2673	2365	2654	*invasion associated locus B family protein*	Unknown	Unknown
RCAP_rcc01870	0.39	0.0111	672	885	0.01%	0.01%	578	774	787	983	*fabF2*	Lipid Metabolism	Biotin metabolism
RCAP_rcc01871	0.25	0.1944	594	710	0.01%	0.01%	480	727	630	790	*acpP2*	Unknown	Unknown
RCAP_rcc01872	0.48	0	1571	2202	0.02%	0.03%	1492	1732	2049	2355	*lpxD*	Cell Envelope Biosynthesis	Cell Wall Biosynthesis
RCAP_rcc01873	-0.49	0	2131	1507	0.03%	0.02%	1843	2353	1388	1626	*peptidoglycan-binding domain 1 protein*	Unknown	Unknown
RCAP_rcc01874	-0.63	0	4983	3205	0.07%	0.04%	4220	5628	2936	3474	*hypothetical protein*	Unknown	Unknown
RCAP_rcc01875	-1.29	0	2951	1188	0.04%	0.02%	2453	3264	1057	1318	*sufS1*	Metabolism of Cofactors, Coenzymes and Vitamins	Thiamine metabolism
RCAP_rcc01876	-1.31	0	1354	535	0.02%	0.01%	1113	1500	482	588	*hypothetical protein*	Unknown	Unknown
RCAP_rcc01877	-0.9	0	1156	601	0.02%	0.01%	806	1447	551	651	*hypothetical protein*	Unknown	Unknown
RCAP_rcc01878	-1.5	0	6438	2235	0.09%	0.03%	5228	7224	2051	2420	*sufD*	Post-translational Modification, Assembly and Chaperones	Unknown
RCAP_rcc01879	-1.42	0	6336	2314	0.09%	0.03%	5111	7138	2001	2626	*sufC*	Post-translational Modification, Assembly and Chaperones	Unknown
RCAP_rcc01880	-1.29	0	2260	890	0.03%	0.01%	1800	2574	705	1075	*hypothetical protein*	Unknown	Unknown
RCAP_rcc01881	-0.7	0.0023	11275	6786	0.16%	0.09%	8999	13303	5034	8537	*sufB*	Post-translational Modification, Assembly and Chaperones	Unknown
RCAP_rcc01882	0.11	0.5956	1083	1167	0.02%	0.02%	946	1237	952	1382	*iscS*	Metabolism of Cofactors, Coenzymes and Vitamins	Thiamine metabolism
RCAP_rcc01883	0.01	0.9765	669	673	0.01%	0.01%	498	826	591	754	*iscR*	Transcription	Unknown
RCAP_rcc01884	0.44	0.0156	1816	2496	0.03%	0.03%	1547	2228	2129	2863	*alpha/beta fold family hydrolase*	Unknown	Unknown
RCAP_rcc01885	0.64	0.0211	105	170	0.00%	0.00%	81	139	140	200	*hypothetical protein*	Unknown	Unknown
RCAP_rcc01886	-0.21	0.1072	636	549	0.01%	0.01%	564	703	495	603	*metal dependent phosphohydrolase*	Unknown	Unknown
RCAP_rcc01887	-0.33	0.0872	16685	13152	0.23%	0.18%	13082	19768	11178	15126	*icd*	Carbohydrate Metabolism	TCA Cycle
RCAP_rcc01888	-0.03	0.8779	196	191	0.00%	0.00%	181	220	154	227	*hypothetical protein*	Unknown	Unknown
RCAP_rcc01889	-0.18	0.346	182	161	0.00%	0.00%	152	212	136	185	*hypothetical protein*	Unknown	Unknown
RCAP_rcc01890	-0.15	0.6703	629	562	0.01%	0.01%	359	870	442	683	*hypothetical protein*	Unknown	Unknown
RCAP_rcc01891	-0.54	0.0369	846	567	0.01%	0.01%	621	1041	455	679	*emrE*	Metal and Ion Transport	Unknown
RCAP_rcc01892	-0.5	0.0002	7659	5396	0.11%	0.07%	7068	8056	4609	6184	*typA*	Signal Transduction	Kinase/Phosphorelay
RCAP_rcc01893	-0.35	0	2733	2151	0.04%	0.03%	2537	2880	2009	2292	*potG1*	Amino Acid Metabolism	Amino Acid Transport
RCAP_rcc01894	-0.38	0.0467	3077	2352	0.04%	0.03%	2493	3649	2035	2670	*potI2*	Metal and Ion Transport	Unknown
RCAP_rcc01895	-0.16	0.4344	2301	2054	0.03%	0.03%	1840	2748	1775	2334	*potH1*	Metal and Ion Transport	Unknown
RCAP_rcc01896	0.51	0.0003	495	710	0.01%	0.01%	431	583	649	770	*GntR family transcriptional regulator*	Signal Transduction	Transcription Regulator
RCAP_rcc01897	0.57	0.2193	607	1008	0.01%	0.01%	152	1038	478	1537	*hypothetical protein*	Unknown	Unknown
RCAP_rcc01898	-0.35	0.0011	917	714	0.01%	0.01%	819	979	646	782	*hypothetical protein*	Unknown	Unknown
RCAP_rcc01899	-0.23	0.1021	804	681	0.01%	0.01%	694	885	594	768	*hypothetical protein*	Unknown	Unknown
RCAP_rcc01900	-0.71	0.0002	15780	9476	0.22%	0.13%	11205	19758	8838	10114	*hemolysin-type calcium-binding repeat family protein*	Trafficking and Secretion	Secretion
RCAP_rcc01901	0.77	0	580	991	0.01%	0.01%	565	643	936	1046	*polyphosphate kinase 2 domain-containing protein*	Energy Metabolism	Oxidative phosphorylation
RCAP_rcc01902	0.3	0.0786	80	99	0.00%	0.00%	68	93	87	111	*TetR family transcriptional regulator*	Signal Transduction	Transcription Regulator
RCAP_rcc01903	0.2	0.3659	108	124	0.00%	0.00%	86	131	111	138	*lysine exporter protein (LYSE/YGGA)*	Unknown	Unknown
RCAP_rcc01904	0.53	0.0517	27	40	0.00%	0.00%	22	36	32	48	*AsnC/Lrp family transcriptional regulator*	Signal Transduction	Transcription Regulator
RCAP_rcc01905	-0.06	0.8443	376	360	0.01%	0.00%	280	485	300	420	*IS4 family transposase*	Replication, Recombination and Repair	Recombination
RCAP_rcc01906	0.55	0	344	504	0.00%	0.01%	333	374	465	544	*hypothetical protein*	Unknown	Unknown
RCAP_rcc01907	0.21	0.0104	1513	1749	0.02%	0.02%	1435	1615	1618	1880	*SmpA/OmlA domain-containing protein*	Translation, ribosomal structure and biogenesis	Unknown
RCAP_rcc01908	0.01	0.9664	145	146	0.00%	0.00%	111	176	122	170	*hypothetical protein*	Unknown	Unknown
RCAP_rcc01909	0.53	0.0056	1985	2900	0.03%	0.04%	1610	2532	2539	3262	*rpmF*	Translation, ribosomal structure and biogenesis	Unknown
RCAP_rcc01910	0.19	0.2398	1309	1503	0.02%	0.02%	1139	1488	1345	1662	*plsX*	Lipid Metabolism	Unknown
RCAP_rcc01911	-0.02	0.7965	2344	2314	0.03%	0.03%	2188	2457	2252	2377	*fabH2*	Lipid Metabolism	Biotin metabolism
RCAP_rcc01912	-0.15	0.2775	4107	3684	0.06%	0.05%	3634	4545	3253	4116	*ihfA*	Replication, Recombination and Repair	Unknown
RCAP_rcc01913	-0.36	0.0021	8245	6384	0.11%	0.09%	7111	9105	5866	6903	*MerR family transcriptional regulator*	Signal Transduction	Transcription Regulator
RCAP_rcc01914	0.13	0.3259	546	600	0.01%	0.01%	468	624	563	636	*dcd*	Nucleotide Metabolism	Pyrimidine metabolism
RCAP_rcc01915	-0.26	0.0191	2440	2028	0.03%	0.03%	2199	2636	1845	2211	*guaB*	Xenobiotics Biodegradation and Metabolism	Drug metabolism - other enzymes
RCAP_rcc01916	0.52	0.1066	188	281	0.00%	0.00%	113	258	212	350	*C4-dicarboxylate transporter/malic acid transport protein family*	Metal and Ion Transport	Unknown
RCAP_rcc01917	0.87	0.0512	6	12	0.00%	0.00%	3	8	3	20	*hypothetical protein*	Unknown	Unknown
RCAP_rcc01918	0.64	0.2363	3	7	0.00%	0.00%	1	5	2	11	*hypothetical protein*	Unknown	Unknown
RCAP_rcc01919	-0.52	0.0161	13181	9042	0.18%	0.12%	10672	15464	7284	10800	*membrane protein involved in aromatic hydrocarbon degradation*	Cell Envelope Biosynthesis	Cell Wall Biosynthesis
RCAP_rcc01920	-0.79	0	620	352	0.01%	0.00%	463	750	316	388	*hypothetical protein*	Unknown	Unknown
RCAP_rcc01921	-0.55	0.0018	1395	941	0.02%	0.01%	1161	1597	785	1097	*hypothetical protein*	Unknown	Unknown
RCAP_rcc01922	0.7	0	1422	2328	0.02%	0.03%	1395	1661	2021	2635	*metA*	Energy Metabolism	Sulfur metabolism
RCAP_rcc01923	0.43	0.0054	303	412	0.00%	0.01%	270	362	371	453	*phage integrase*	Replication, Recombination and Repair	Phage Interaction
RCAP_rcc01925	0.69	0	152	247	0.00%	0.00%	136	180	228	267	*hypothetical protein*	Unknown	Unknown
RCAP_rcc01926	0.45	0.0299	149	206	0.00%	0.00%	123	183	172	240	*rnhA1*	Unknown	Unknown
RCAP_rcc01927	0.75	0	81	139	0.00%	0.00%	68	99	121	156	*hypothetical protein*	Unknown	Unknown
RCAP_rcc01928	-0.02	0.9349	2661	2622	0.04%	0.04%	2125	3162	2258	2986	*hsdR3*	Defense Mechanisms	Unknown
RCAP_rcc01929	-0.37	0.2232	957	722	0.01%	0.01%	664	1228	542	901	*hsdS3*	Defense Mechanisms	Unknown
RCAP_rcc01930	0.14	0.5482	666	739	0.01%	0.01%	522	832	627	851	*hsdM3*	Defense Mechanisms	Unknown
RCAP_rcc01931	2.68	0	62	486	0.00%	0.01%	89	142	344	628	*resolvase*	Replication, Recombination and Repair	Unknown
RCAP_rcc01932	-0.01	0.9764	1588	1579	0.02%	0.02%	1213	1989	1422	1737	*family 4 glycosyl transferase*	Metabolism of Cofactors, Coenzymes and Vitamins	Pantothenate and CoA biosynthesis
RCAP_rcc01933	0.06	0.8482	108	113	0.00%	0.00%	77	139	82	143	*hypothetical protein*	Unknown	Unknown
RCAP_rcc01934	0.07	0.8445	59	62	0.00%	0.00%	39	76	45	78	*manB*	Carbohydrate Metabolism	Fructose and mannose metabolism
RCAP_rcc01935	-0.05	0.472	567	548	0.01%	0.01%	542	588	513	583	*xanB*	Carbohydrate Metabolism	Fructose and mannose metabolism
RCAP_rcc01936	-0.17	0.0897	316	280	0.00%	0.00%	294	333	252	308	*gne*	Carbohydrate Metabolism	Amino sugar and nucleotide sugar metabolism
RCAP_rcc01937	-0.2	0.5367	113	97	0.00%	0.00%	77	144	73	121	*hypothetical protein*	Unknown	Unknown
RCAP_rcc01938	-0.1	0.3495	361	337	0.01%	0.00%	329	393	313	360	*hypothetical protein*	Unknown	Unknown
RCAP_rcc01939	-0.14	0.4561	134	121	0.00%	0.00%	111	153	105	138	*polysaccharide biosynthesis protein family*	Cell Envelope Biosynthesis	Cell Wall Biosynthesis
RCAP_rcc01940	-0.18	0.2177	789	695	0.01%	0.01%	680	906	631	760	*hemolysin-type calcium-binding repeat family protein*	Trafficking and Secretion	Secretion
RCAP_rcc01941	0.44	0.0134	134	184	0.00%	0.00%	113	153	150	218	*FAD dependent oxidoreductase*	Energy Metabolism	Unknown
RCAP_rcc01942	-0.14	0.7219	79	70	0.00%	0.00%	42	111	43	98	*polysaccharide pyruvyl transferase*	Cell Envelope Biosynthesis	Cell Wall Biosynthesis
RCAP_rcc01943	0.08	0.832	59	62	0.00%	0.00%	35	78	40	85	*group 1 glycosyl transferase*	Cell Envelope Biosynthesis	Cell Wall Biosynthesis
RCAP_rcc01944	0.26	0.2523	208	251	0.00%	0.00%	163	254	217	284	*family 2 glycosyl transferase*	Cell Envelope Biosynthesis	Cell Wall Biosynthesis

RCAP_rcc01945	-0.26	0.1774	384	319	0.01%	0.00%	300	453	279	360	*group 1 glycosyl transferase*	Cell Envelope Biosynthesis	Cell Wall Biosynthesis
RCAP_rcc01946	-0.3	0.0001	626	507	0.01%	0.01%	572	659	476	537	*hypothetical protein*	Unknown	Unknown
RCAP_rcc01947	-0.32	0.2724	100	78	0.00%	0.00%	69	124	63	93	*twin-arginine translocation pathway signal domain-containing protein*	Unknown	Unknown
RCAP_rcc01948	-0.36	0	915	712	0.01%	0.01%	816	988	688	736	*lipopolysaccharide biosynthesis family protein*	Cell Envelope Biosynthesis	Cell Wall Biosynthesis
RCAP_rcc01949	-0.73	0.001	446	262	0.01%	0.00%	313	557	228	297	*undecaprenyl-phosphate galactosephosphotransferase*	Cell Envelope Biosynthesis	Cell Wall Biosynthesis
RCAP_rcc01950	0.14	0.0396	534	589	0.01%	0.01%	503	567	562	616	*polysaccharide biosynthesis/export family protein*	Cell Envelope Biosynthesis	Cell Wall Biosynthesis
RCAP_rcc01951	0.47	0.1832	80	116	0.00%	0.00%	50	110	82	149	*hypothetical protein*	Unknown	Unknown
RCAP_rcc01952	-0.4	0.0021	887	670	0.01%	0.01%	759	996	611	730	*lspL2*	Carbohydrate Metabolism	Amino sugar and nucleotide sugar metabolism
RCAP_rcc01953	-0.04	0.8775	182	177	0.00%	0.00%	156	201	130	225	*hypothetical protein*	Unknown	Unknown
RCAP_rcc01954	-0.31	0.0665	1406	1131	0.02%	0.02%	1155	1636	1018	1245	*hypothetical protein*	Unknown	Unknown
RCAP_rcc01955	0.22	0.3769	376	440	0.01%	0.01%	321	424	282	599	*hypothetical protein*	Unknown	Unknown
RCAP_rcc01956	-0.42	0.0001	414	308	0.01%	0.00%	357	456	292	324	*lipoprotein*	Predicted Function	Unknown
RCAP_rcc01957	-0.13	0.201	1026	939	0.01%	0.01%	925	1107	879	998	*hypothetical protein*	Unknown	Unknown
RCAP_rcc01958	-0.2	0.0512	1108	966	0.02%	0.01%	993	1195	897	1035	*wzc*	Signal Transduction	Kinase/Phosphorelay
RCAP_rcc01959	-0.02	0.9306	129	126	0.00%	0.00%	109	143	108	145	*wzb*	Unknown	Unknown
RCAP_rcc01960	-0.23	0.0801	1350	1149	0.02%	0.02%	1165	1474	1025	1274	*wza*	Cell Envelope Biosynthesis	Cell Wall Biosynthesis
RCAP_rcc01961	-0.23	0.2362	162	137	0.00%	0.00%	125	193	122	152	*gluQ*	Translation, ribosomal structure and biogenesis	Unknown
RCAP_rcc01962	-0.43	0.0001	1304	965	0.02%	0.01%	1153	1428	888	1041	*gid*	Translation, ribosomal structure and biogenesis	Unknown
RCAP_rcc01963	-0.82	0	1864	1039	0.03%	0.01%	1567	2086	900	1179	*hypothetical protein*	Unknown	Unknown
RCAP_rcc01964	-0.31	0.0194	4009	3223	0.06%	0.04%	3510	4459	2892	3554	*hypothetical protein*	Unknown	Unknown
RCAP_rcc01965	-0.33	0.0002	8050	6373	0.11%	0.09%	7447	8519	5878	6869	*gyrA*	Replication, Recombination and Repair	Unknown
RCAP_rcc01966	-0.11	0.5842	159	147	0.00%	0.00%	128	191	132	161	*disulfide bond formation protein, DsbB family*	Sulfur Metabolism	Replication
RCAP_rcc01967	-0.2	0.1081	452	393	0.01%	0.01%	402	495	354	432	*hypothetical protein*	Unknown	Unknown
RCAP_rcc01968	1.15	0	41	96	0.00%	0.00%	33	54	77	114	*zinc peptidase*	Metal and Ion Transport	Signaling and Trafficking
RCAP_rcc01969	-0.41	0.0755	38	29	0.00%	0.00%	30	44	23	34	*hypothetical protein*	Unknown	Unknown
RCAP_rcc01970	-0.62	0.046	14	8	0.00%	0.00%	10	16	5	11	*hypothetical protein*	Unknown	Unknown
RCAP_rcc01971	0.57	0.3134	0	1	0.00%	0.00%	0	1	0	1	*hypothetical protein*	Unknown	Unknown
RCAP_rcc01972	0.2	0.7089	1	1	0.00%	0.00%	1	2	1	2	*hypothetical protein*	Unknown	Unknown
RCAP_rcc01973	0.16	0.6725	3	4	0.00%	0.00%	2	4	3	5	*hypothetical protein*	Unknown	Unknown
RCAP_rcc01974	-0.27	0.3147	7	6	0.00%	0.00%	5	8	5	7	*hypothetical protein*	Unknown	Unknown
RCAP_rcc01975	-0.51	0.0247	2640	1816	0.04%	0.02%	1978	3194	1428	2203	*hypothetical protein*	Unknown	Unknown
RCAP_rcc01976	0.54	0.1414	3	5	0.00%	0.00%	2	4	3	6	*hypothetical protein*	Unknown	Unknown
RCAP_rcc01977	0.36	0.5315	1	1	0.00%	0.00%	0	1	0	1	*hypothetical protein*	Unknown	Unknown
RCAP_rcc01978	0.72	0.1747	0	0	0.00%	0.00%	0	0	0	1	*hypothetical protein*	Unknown	Unknown
RCAP_rcc01979	0.28	0.3289	8	10	0.00%	0.00%	5	10	8	11	*hypothetical protein*	Unknown	Unknown
RCAP_rcc01980	0.49	0.2418	2	3	0.00%	0.00%	1	3	2	4	*hypothetical protein*	Unknown	Unknown
RCAP_rcc01981	1.16	0.0029	2	5	0.00%	0.00%	1	3	3	6	*hypothetical protein*	Unknown	Unknown
RCAP_rcc01982	-0.51	0.1028	15	10	0.00%	0.00%	10	19	7	13	*HNH endonuclease*	Unknown	Unknown
RCAP_rcc01983	0	0.9948	8	8	0.00%	0.00%	6	10	7	9	*phage terminase small subunit*	Replication, Recombination and Repair	Phage Interaction
RCAP_rcc01984	0.33	0.0741	27	34	0.00%	0.00%	22	32	30	39	*phage terminase large subunit*	Replication, Recombination and Repair	Phage Interaction
RCAP_rcc01985	0.49	0.0605	6	9	0.00%	0.00%	5	8	7	11	*HK97 family phage portal protein*	Replication, Recombination and Repair	Phage Interaction
RCAP_rcc01986	0.79	0.1397	1	2	0.00%	0.00%	0	1	1	3	*S49 family peptidase*	Post-translational Modification, Assembly and Chaperones	Peptidase
RCAP_rcc01987	0.89	0	9	18	0.00%	0.00%	8	12	15	20	*HK97 family phage major capsid protein*	Replication, Recombination and Repair	Phage Interaction
RCAP_rcc01988	1.22	0.001	2	5	0.00%	0.00%	1	3	3	7	*hypothetical protein*	Unknown	Unknown
RCAP_rcc01989	2.62	NA	4	52	0.00%	0.00%	3	7	8	95	*hypothetical protein*	Unknown	Unknown
RCAP_rcc01990	1.86	0	2	12	0.00%	0.00%	1	5	5	20	*hypothetical protein*	Unknown	Unknown
RCAP_rcc01991	0.98	0.0702	0	1	0.00%	0.00%	0	0	0	1	*hypothetical protein*	Unknown	Unknown
RCAP_rcc01992	1.09	0.0047	3	8	0.00%	0.00%	2	5	5	12	*hypothetical protein*	Unknown	Unknown
RCAP_rcc01993	0.5	0.0051	12	17	0.00%	0.00%	10	14	15	19	*hypothetical protein*	Unknown	Unknown
RCAP_rcc01994	0.59	0.1777	3	5	0.00%	0.00%	2	5	3	7	*hypothetical protein*	Unknown	Unknown
RCAP_rcc01995	0.76	0.1671	1	2	0.00%	0.00%	0	1	0	3	*hypothetical protein*	Unknown	Unknown
RCAP_rcc01996	1.42	0	13	36	0.00%	0.00%	12	17	29	44	*phage membrane protein*	Replication, Recombination and Repair	Phage Interaction
RCAP_rcc01997	0.39	0.0791	12	15	0.00%	0.00%	10	14	12	19	*hypothetical protein*	Unknown	Unknown
RCAP_rcc01998	0.94	0.0077	4	8	0.00%	0.00%	3	5	5	11	*hypothetical protein*	Unknown	Unknown
RCAP_rcc01999	1.53	0.0033	0	3	0.00%	0.00%	0	1	1	5	*hypothetical protein*	Unknown	Unknown
RCAP_rcc02000	0.99	0.0301	1	4	0.00%	0.00%	1	2	2	6	*hypothetical protein*	Unknown	Unknown
RCAP_rcc02001	0.78	0.0766	7	14	0.00%	0.00%	4	11	5	23	*peptidoglycan-binding domain 1 protein*	Unknown	Unknown
RCAP_rcc02002	0.22	0.3501	41	48	0.00%	0.00%	31	49	39	58	*hypothetical protein*	Unknown	Unknown
RCAP_rcc02003	-0.01	0.9606	206	204	0.00%	0.00%	159	247	188	220	*dam*	Unknown	Unknown
RCAP_rcc02004	0.13	0.4913	527	579	0.01%	0.01%	409	643	522	636	*hypothetical protein*	Unknown	Unknown
RCAP_rcc02005	0.53	0.0187	3507	5142	0.05%	0.07%	2945	4343	4119	6164	*hypothetical protein*	Unknown	Unknown
RCAP_rcc02006	-0.1	0.6477	2998	2787	0.04%	0.04%	2442	3497	2359	3214	*hypothetical protein*	Unknown	Unknown
RCAP_rcc02007	0.19	0.3141	85	97	0.00%	0.00%	70	101	86	107	*phage integrase*	Replication, Recombination and Repair	Phage Interaction
RCAP_rcc02008	-0.45	0.0512	18914	13682	0.26%	0.19%	14873	22648	11072	16293	*tig*	Post-translational Modification, Assembly and Chaperones	Unknown
RCAP_rcc02009	0.05	0.8268	7925	8217	0.11%	0.11%	6886	9106	6582	9852	*rplI*	Translation, ribosomal structure and biogenesis	Unknown
RCAP_rcc02010	-0.01	0.9578	4169	4125	0.06%	0.06%	3567	4889	3118	5131	*rpsR*	Translation, ribosomal structure and biogenesis	Unknown
RCAP_rcc02011	-0.14	0.5912	8688	7817	0.12%	0.11%	6894	10529	5981	9654	*rpsF*	Translation, ribosomal structure and biogenesis	Unknown
RCAP_rcc02012	-0.26	0.4762	594	484	0.01%	0.01%	420	793	294	673	*hypothetical protein*	Unknown	Unknown
RCAP_rcc02013	0.62	0	374	578	0.01%	0.01%	347	425	542	613	*hypothetical protein*	Unknown	Unknown
RCAP_rcc02014	0.78	0	534	926	0.01%	0.01%	466	656	864	989	*hypothetical protein*	Unknown	Unknown
RCAP_rcc02015	0.93	0.0001	947	1877	0.01%	0.03%	690	1279	1600	2155	*aldH1*	Carbohydrate Metabolism	Glycolysis / Gluconeogenesis
RCAP_rcc02016	0.72	0.0001	2769	4645	0.04%	0.06%	2477	3247	3837	5453	*fumarylacetoacetate hydrolase*	Secondary metabolites biosynthesis, transport, and catabolism	Unknown
RCAP_rcc02017	-1.02	0.0001	1057	497	0.01%	0.01%	710	1352	368	627	*rbsA*	Unknown	Unknown
RCAP_rcc02018	-0.96	0.0001	1717	847	0.02%	0.01%	1278	2066	599	1095	*rbsC*	Carbohydrate Metabolism	Unknown
RCAP_rcc02019	-1.38	0	8739	3125	0.12%	0.04%	6224	10774	2339	3911	*rbsB*	Carbohydrate Metabolism	Unknown
RCAP_rcc02020	0.55	0	427	628	0.01%	0.01%	378	491	559	696	*HAD superfamily hydrolase*	Unknown	Unknown
RCAP_rcc02021	0.24	0.0508	231	274	0.00%	0.00%	212	252	241	306	*DeoR family transcriptional regulator*	Signal Transduction	Transcription Regulator

RCAP_rcc02022	-0.65	0.0255	7023	4310	0.10%	0.06%	6359	8027	2222	6398	*polyols ABC transporter periplasmic polyols-binding protein*	Unknown	Unknown
RCAP_rcc02023	-0.41	0.1923	366	269	0.01%	0.00%	317	438	141	398	*polyols ABC transporter permease*	Metal and Ion Transport	Unknown
RCAP_rcc02024	-0.47	0.0943	331	232	0.00%	0.00%	285	391	135	330	*polyols ABC transporter permease*	Metal and Ion Transport	Unknown
RCAP_rcc02025	-0.34	0.2114	374	289	0.01%	0.00%	336	426	146	433	*polyols ABC transporter ATP-binding protein*	Amino Acid Metabolism	Amino Acid Transport
RCAP_rcc02026	-0.25	NA	223	185	0.00%	0.00%	201	257	92	278	*polS*	Carbohydrate Metabolism	Fructose and mannose metabolism
RCAP_rcc02027	-0.22	NA	501	425	0.01%	0.01%	413	608	164	686	*mtlK*	Carbohydrate Metabolism	Fructose and mannose metabolism
RCAP_rcc02028	1.31	0	30	78	0.00%	0.00%	25	38	64	92	*FeoA family protein*	Metal, Ion, Cofactor Transport	Iron and Heme Transport
RCAP_rcc02029	0.19	0.0089	601	687	0.01%	0.01%	574	628	641	734	*feoB2*	Metal, Ion, Cofactor Transport	Iron and Heme Transport
RCAP_rcc02030	1.1	0	193	417	0.00%	0.01%	182	223	386	448	*allA*	Nucleotide Metabolism	Purine metabolism
RCAP_rcc02031	1	0	477	960	0.01%	0.01%	458	542	898	1022	*hypothetical protein*	Unknown	Unknown
RCAP_rcc02032	-1.14	0	665	299	0.01%	0.00%	577	709	269	329	*cobB*	Metabolism of Cofactors, Coenzymes and Vitamins	Cobalamin Biosynthesis
RCAP_rcc02033	-0.91	0.0009	597	302	0.01%	0.00%	403	743	218	386	*cobZ*	Metabolism of Cofactors, Coenzymes and Vitamins	Cobalamin Biosynthesis
RCAP_rcc02034	-2.41	0	772	142	0.01%	0.00%	645	832	131	153	*cbiO2*	Metabolism of Cofactors, Coenzymes and Vitamins	Cobalamin Biosynthesis
RCAP_rcc02035	-2.34	0	892	158	0.01%	0.00%	619	1088	134	182	*cbiQ2*	Metabolism of Cofactors, Coenzymes and Vitamins	Cobalamin Biosynthesis
RCAP_rcc02036	-2.75	0	1984	241	0.03%	0.00%	1164	2618	195	287	*cbiN*	Metabolism of Cofactors, Coenzymes and Vitamins	Cobalamin Biosynthesis
RCAP_rcc02037	-3.17	0	13131	1408	0.18%	0.02%	11486	13586	1233	1584	*CbiM family cobalamin biosynthesis protein*	Metabolism of Cofactors, Coenzymes and Vitamins	Cobalamin Biosynthesis
RCAP_rcc02038	-0.41	0.0031	460	345	0.01%	0.00%	420	496	297	394	*cobF*	Metabolism of Cofactors, Coenzymes and Vitamins	Cobalamin Biosynthesis
RCAP_rcc02039	-0.25	0.1658	194	162	0.00%	0.00%	159	223	138	185	*cobA2*	Metabolism of Cofactors, Coenzymes and Vitamins	Cobalamin Biosynthesis
RCAP_rcc02040	0	0.9949	274	274	0.00%	0.00%	213	331	234	313	*cobM*	Metabolism of Cofactors, Coenzymes and Vitamins	Cobalamin Biosynthesis
RCAP_rcc02041	-0.12	0.7469	85	78	0.00%	0.00%	55	112	55	100	*cbiG*	Metabolism of Cofactors, Coenzymes and Vitamins	Cobalamin Biosynthesis
RCAP_rcc02042	-0.6	0.0032	255	165	0.00%	0.00%	199	297	134	196	*cobL*	Metabolism of Cofactors, Coenzymes and Vitamins	Cobalamin Biosynthesis
RCAP_rcc02043	-0.4	0.0848	207	154	0.00%	0.00%	154	248	124	184	*cobK*	Metabolism of Cofactors, Coenzymes and Vitamins	Cobalamin Biosynthesis
RCAP_rcc02044	-0.44	0.0449	490	356	0.01%	0.00%	378	584	300	413	*cobJ*	Metabolism of Cofactors, Coenzymes and Vitamins	Cobalamin Biosynthesis
RCAP_rcc02045	-0.6	0	861	563	0.01%	0.01%	775	915	490	635	*cobI*	Metabolism of Cofactors, Coenzymes and Vitamins	Cobalamin Biosynthesis
RCAP_rcc02046	-0.63	0.0017	629	398	0.01%	0.01%	475	749	330	466	*cobH*	Metabolism of Cofactors, Coenzymes and Vitamins	Cobalamin Biosynthesis
RCAP_rcc02047	-0.71	0	2128	1286	0.03%	0.02%	1722	2422	1116	1457	*cobN*	Metabolism of Cofactors, Coenzymes and Vitamins	Cobalamin Biosynthesis
RCAP_rcc02048	-0.66	0	2362	1487	0.03%	0.02%	2018	2600	1307	1666	*cobW*	Metabolism of Cofactors, Coenzymes and Vitamins	Cobalamin Biosynthesis
RCAP_rcc02049	-0.33	0.2453	157	122	0.00%	0.00%	89	219	105	139	*hypothetical protein*	Unknown	Unknown
RCAP_rcc02050	0.16	0.3765	1175	1312	0.02%	0.02%	1020	1367	1154	1471	*cobO*	Metabolism of Cofactors, Coenzymes and Vitamins	Cobalamin Biosynthesis
RCAP_rcc02051	-0.08	0.6928	752	709	0.01%	0.01%	601	889	629	790	*cobQ1*	Metabolism of Cofactors, Coenzymes and Vitamins	Cobalamin Biosynthesis
RCAP_rcc02052	-0.23	0.0407	577	492	0.01%	0.01%	501	641	468	515	*bluB*	Metabolism of Cofactors, Coenzymes and Vitamins	Unknown
RCAP_rcc02053	0.08	0.8201	216	230	0.00%	0.00%	129	292	164	296	*cobC*	Metabolism of Cofactors, Coenzymes and Vitamins	Cobalamin Biosynthesis
RCAP_rcc02054	0.13	0.5569	262	287	0.00%	0.00%	205	315	251	323	*cobD*	Metabolism of Cofactors, Coenzymes and Vitamins	Cobalamin Biosynthesis
RCAP_rcc02055	0.35	0.4703	51	70	0.00%	0.00%	19	82	32	107	*hypothetical protein*	Unknown	Unknown
RCAP_rcc02056	0.28	0.5315	79	99	0.00%	0.00%	32	122	52	146	*phosphoglycerate mutase*	Metabolism of Cofactors, Coenzymes and Vitamins	Porphyrin and chlorophyll metabolism
RCAP_rcc02057	1	0	113	227	0.00%	0.00%	104	135	211	242	*cobP*	Metabolism of Cofactors, Coenzymes and Vitamins	Cobalamin Biosynthesis
RCAP_rcc02058	0.49	0.0116	583	828	0.01%	0.01%	458	713	708	949	*cobU*	Metabolism of Cofactors, Coenzymes and Vitamins	Cobalamin Biosynthesis
RCAP_rcc02059	0.55	0.2144	197	316	0.00%	0.00%	96	289	188	443	*cobV*	Metabolism of Cofactors, Coenzymes and Vitamins	Cobalamin Biosynthesis
RCAP_rcc02060	-0.17	0.1963	811	719	0.01%	0.01%	697	894	652	786	*hemolysin-type calcium-binding repeat family protein*	Trafficking and Secretion	Secretion
RCAP_rcc02061	0.82	0	168	302	0.00%	0.00%	146	206	262	342	*GNAT family acetyltransferase*	Cell Division	Chromosome Partitioning
RCAP_rcc02062	-0.79	0.0038	142	78	0.00%	0.00%	94	180	63	93	*Hsp70 family heat shock protein*	Stress Response	Unknown
RCAP_rcc02063	-1.77	0	1538	417	0.02%	0.01%	991	1955	370	465	*M10 family peptidase*	Post-translational Modification, Assembly and Chaperones	Peptidase
RCAP_rcc02064	0.76	0.0001	370	639	0.01%	0.01%	293	458	559	719	*zntA1*	Metal, Ion, Cofactor Transport	Zinc Transport
RCAP_rcc02065	-0.24	0.0938	1416	1199	0.02%	0.02%	1221	1580	1085	1312	*secretion ATP-binding protein, HlyB family*	Trafficking and Secretion	Secretion
RCAP_rcc02066	-0.18	0.6008	317	276	0.00%	0.00%	191	430	191	360	*hemolysin D*	Trafficking and Secretion	Secretion
RCAP_rcc02067	-0.15	0.2034	1084	979	0.02%	0.01%	973	1204	905	1053	*type 11 family methyltransferase*	Unknown	Unknown
RCAP_rcc02068	-0.12	0.6865	5174	4750	0.07%	0.06%	3654	6644	4204	5297	*ice nucleation protein repeat family protein*	Unknown	Unknown
RCAP_rcc02069	-0.16	0.5758	666	593	0.01%	0.01%	484	827	468	718	*hypothetical protein*	Unknown	Unknown
RCAP_rcc02070	-0.77	0	172	100	0.00%	0.00%	145	194	91	108	*ArsR family transcriptional regulator/protein tyrosine phosphatase*	Signal Transduction	Transcription Regulator
RCAP_rcc02071	-0.32	0.0246	224	179	0.00%	0.00%	194	255	161	196	*gap2*	Carbohydrate Metabolism	Glycolysis / Gluconeogenesis
RCAP_rcc02072	-0.23	0.1328	723	616	0.01%	0.01%	614	827	571	660	*nonfunctional major facilitator superfamily protein*	Unknown	Unknown
RCAP_rcc02074	-1.17	0	1834	800	0.03%	0.01%	1496	2066	743	857	*ferredoxin domain-containing protein*	Energy Metabolism	Aerobic/Anaerobic Respiration
RCAP_rcc02075	-0.4	0.0004	1108	839	0.02%	0.01%	970	1208	764	913	*PAS/PAC sensor domain-containing protein*	Motility	Aerotaxis
RCAP_rcc02076	0.05	0.6387	1066	1108	0.01%	0.02%	969	1163	1039	1177	*diguanylate cyclase/phosphodiesterase*	Signal Transduction	Kinase/Phosphorelay
RCAP_rcc02077	0.6	0.0009	385	593	0.01%	0.01%	333	469	516	669	*SET domain-containing protein*	Unknown	Unknown
RCAP_rcc02078	0.6	0.0122	285	444	0.00%	0.01%	228	368	385	502	*hypothetical protein*	Unknown	Unknown
RCAP_rcc02079	-0.39	0.0208	2872	2176	0.04%	0.03%	2367	3305	1910	2442	*parE*	Replication, Recombination and Repair	Unknown
RCAP_rcc02080	-0.41	0.0017	647	487	0.01%	0.01%	543	735	462	511	*HAD superfamily hydrolase*	Carbohydrate Metabolism	Unknown
RCAP_rcc02081	-0.68	0.0001	368	226	0.01%	0.00%	303	426	193	259	*MaoC family protein*	Carbohydrate Metabolism	Glyoxylate and dicarboxylate metabolism
RCAP_rcc02082	0.23	0.0104	705	825	0.01%	0.01%	655	762	765	884	*ribF*	Metabolism of Cofactors, Coenzymes and Vitamins	Riboflavin metabolism
RCAP_rcc02083	-0.42	0.0477	559	413	0.01%	0.01%	436	659	340	485	*hypothetical protein*	Unknown	Unknown
RCAP_rcc02084	-0.07	0.7159	678	643	0.01%	0.01%	553	793	584	702	*ltaE*	Amino Acid Metabolism	Glycine, serine and threonine metabolism
RCAP_rcc02085	-0.18	0.05	112	98	0.00%	0.00%	101	119	92	104	*phage integrase*	Replication, Recombination and Repair	Phage Interaction
RCAP_rcc02086	-0.27	0.6179	1	1	0.00%	0.00%	1	2	1	2	*hypothetical protein*	Unknown	Unknown
RCAP_rcc02088	-0.39	0.0728	46	35	0.00%	0.00%	36	55	29	40	*IS3 family transposase*	Replication, Recombination and Repair	Recombination
RCAP_rcc02095	-0.26	0.2687	39	32	0.00%	0.00%	30	47	26	39	*hypothetical protein*	Unknown	Unknown
RCAP_rcc02097	-0.46	0.0351	116	83	0.00%	0.00%	88	141	71	95	*IS111A/IS1328/IS1533 family transposase/IS116/IS110/IS902 family tra*	Replication, Recombination and Repair	Recombination
RCAP_rcc02100	-0.05	0.8785	47	45	0.00%	0.00%	35	57	36	54	*IS3/IS911 family transposase*	Replication, Recombination and Repair	Recombination
RCAP_rcc02103	0	0.9903	12	12	0.00%	0.00%	9	15	10	13	*IS3/IS911 family transposase*	Replication, Recombination and Repair	Recombination
RCAP_rcc02105	-0.82	0.0042	159	86	0.00%	0.00%	109	197	60	111	*IS3/IS911 family transposase*	Replication, Recombination and Repair	Recombination
RCAP_rcc02106	-0.46	0.0596	5288	3778	0.07%	0.05%	3846	6566	3003	4552	*NACHT domain-containing protein*	Unknown	Unknown
RCAP_rcc02107	-0.62	0	2038	1321	0.03%	0.02%	1715	2275	1209	1433	*hypothetical protein*	Unknown	Unknown
RCAP_rcc02108	-0.42	0.0378	824	610	0.01%	0.01%	644	980	520	700	*hypothetical protein*	Unknown	Unknown
RCAP_rcc02109	-0.18	0.5516	124	108	0.00%	0.00%	86	164	82	135	*hypothetical protein*	Unknown	Unknown
RCAP_rcc02110	-0.41	0.0409	638	475	0.01%	0.01%	508	790	397	553	*multicopper oxidase*	Secondary metabolites biosynthesis, transport, and catabolism	Unknown
RCAP_rcc02111	-0.27	0.4398	147	119	0.00%	0.00%	90	199	85	153	*hypothetical protein*	Unknown	Unknown
RCAP_rcc02112	-1.27	0	1254	492	0.02%	0.01%	730	1690	438	546	*glyoxalase/bleomycin resistance protein/dioxygenase*	Amino Acid Metabolism	Unknown

RCAP_rcc02113	-0.78	NA	9207	5159	0.13%	0.07%	5200	12866	4605	5713	*arcB1*	Amino Acid Metabolism	Arginine and proline metabolism
RCAP_rcc02114	-0.21	0.5995	4741	4018	0.07%	0.05%	2487	6976	3288	4747	*arcA*	Amino Acid Metabolism	Arginine and proline metabolism
RCAP_rcc02115	0.4	0.0043	53	71	0.00%	0.00%	46	61	62	80	*AsnC/Lrp family transcriptional regulator*	Signal Transduction	Transcription Regulator
RCAP_rcc02116	-1.72	0	494	141	0.01%	0.00%	376	570	106	177	*iron siderophore/cobalamin ABC transporter ATP-binding protein*	Metal, Ion, Cofactor Transport	Iron and Heme Transport
RCAP_rcc02117	-0.39	0.0048	1579	1202	0.02%	0.02%	1370	1750	1057	1347	*methyl-accepting chemotaxis protein*	Motility	Chemotaxis
RCAP_rcc02118	0.22	0.4384	432	510	0.01%	0.01%	313	553	413	606	*cat*	Unknown	Unknown
RCAP_rcc02119	0.4	0.1572	422	569	0.01%	0.01%	290	561	448	690	*type 12 family methyltransferase*	Unknown	Unknown
RCAP_rcc02120	0.76	0.003	184	322	0.00%	0.00%	148	231	220	423	*ABC transporter ATP-binding/periplasmic substrate-binding protein*	Metal and Ion Transport	Unknown
RCAP_rcc02121	0.55	0.046	181	272	0.00%	0.00%	130	229	208	335	*PAS domain/exonuclease domain-containing protein*	Replication, Recombination and Repair	Replication
RCAP_rcc02122	0.42	0.1764	53	73	0.00%	0.00%	37	69	55	92	*response regulator receiver protein*	Signal Transduction	Transcription Regulator
RCAP_rcc02123	0.44	0.0135	368	503	0.01%	0.01%	304	433	421	585	*cyclic nucleotide-binding domain-/cystathionine beta-synthase domain-*	Signal Transduction	Kinase/Phosphorelay
RCAP_rcc02124	0.17	0.4626	5221	5894	0.08%	0.08%	4649	6054	4646	7143	*actP2*	Unknown	Unknown
RCAP_rcc02125	-0.06	0.8449	1679	1609	0.02%	0.02%	1371	2015	1232	1987	*hypothetical protein*	Unknown	Unknown
RCAP_rcc02126	-0.14	0.6006	7385	6659	0.10%	0.09%	5635	9071	5192	8126	*acsA1*	Carbohydrate Metabolism	Glycolysis / Gluconeogenesis
RCAP_rcc02127	1.53	0	45	147	0.00%	0.00%	32	66	102	192	*phaG*	Metal and Ion Transport	Unknown
RCAP_rcc02128	1.68	0	76	255	0.00%	0.00%	80	111	215	296	*phaF*	Metal and Ion Transport	Unknown
RCAP_rcc02129	1.12	0	33	75	0.00%	0.00%	29	40	58	92	*phaE*	Metal and Ion Transport	Unknown
RCAP_rcc02130	1.14	0	270	620	0.00%	0.01%	222	344	482	759	*phaD*	Metal and Ion Transport	Unknown
RCAP_rcc02131	1	0	78	161	0.00%	0.00%	65	101	141	180	*phaC*	Metal and Ion Transport	Unknown
RCAP_rcc02132	1.49	0	850	2457	0.01%	0.03%	820	1078	2030	2883	*phaAB*	Metal and Ion Transport	Unknown
RCAP_rcc02133	1.37	0	147	392	0.00%	0.01%	133	184	345	438	*sbcD*	Replication, Recombination and Repair	Unknown
RCAP_rcc02134	0.35	0.071	129	166	0.00%	0.00%	111	146	130	202	*sbcC*	Unknown	Unknown
RCAP_rcc02135	-0.46	0.0533	379	271	0.01%	0.00%	268	470	222	320	*folD1*	Energy Metabolism	Reductive carboxylate cycle (CO2 fixation)
RCAP_rcc02136	0.11	0.731	113	123	0.00%	0.00%	81	140	87	159	*hypothetical protein*	Unknown	Unknown
RCAP_rcc02137	0.32	0.092	75	94	0.00%	0.00%	60	90	81	107	*hypothetical protein*	Unknown	Unknown
RCAP_rcc02138	0.47	0	390	541	0.01%	0.01%	347	443	508	575	*hypothetical protein*	Unknown	Unknown
RCAP_rcc02139	-0.02	0.9445	530	523	0.01%	0.01%	422	625	408	638	*methyl-accepting chemotaxis protein*	Motility	Chemotaxis
RCAP_rcc02140	-0.27	0.2611	198	163	0.00%	0.00%	135	251	136	190	*extracellular ligand-binding receptor*	Amino Acid Metabolism	Unknown
RCAP_rcc02141	-0.41	0.3161	51	37	0.00%	0.00%	26	72	20	53	*hypothetical protein*	Unknown	Unknown
RCAP_rcc02142	-0.28	0.0064	544	445	0.01%	0.01%	491	572	400	490	*BadM/Rrf2 family transcriptional regulator*	Signal Transduction	Transcription Regulator
RCAP_rcc02143	-0.29	0.2035	37	30	0.00%	0.00%	31	43	23	37	*hypothetical protein*	Unknown	Unknown
RCAP_rcc02144	0.03	0.9424	221	225	0.00%	0.00%	138	295	173	278	*ZapA family cell division protein*	Unknown	Unknown
RCAP_rcc02145	-0.09	0.7406	324	304	0.00%	0.00%	246	392	255	353	*hypothetical protein*	Unknown	Unknown
RCAP_rcc02146	0.09	0.4892	7259	7727	0.10%	0.10%	6536	8179	7141	8313	*tkt2*	Carbohydrate Metabolism	Pentose phosphate pathway
RCAP_rcc02147	-0.39	0.0582	489	369	0.01%	0.01%	376	584	322	415	*hypothetical protein*	Unknown	Unknown
RCAP_rcc02148	0.38	0.0596	378	496	0.01%	0.01%	315	440	399	594	*lipid A biosynthesis acyltransferase*	Cell Envelope Biosynthesis	Cell Wall Biosynthesis
RCAP_rcc02149	0.21	0.2034	723	840	0.01%	0.01%	632	817	700	980	*hypothetical protein*	Unknown	Unknown
RCAP_rcc02150	0.08	0.6116	13582	14371	0.19%	0.20%	11684	15374	12849	15894	*acnA*	Carbohydrate Metabolism	TCA Cycle
RCAP_rcc02151	-0.03	0.889	159	156	0.00%	0.00%	139	175	136	176	*methyl-accepting chemotaxis sensory transducer*	Motility	Chemotaxis
RCAP_rcc02152	0.35	0.0781	285	367	0.00%	0.00%	229	343	300	435	*TM2 domain-containing protein*	Unknown	Unknown
RCAP_rcc02153	-0.14	0.4382	174	158	0.00%	0.00%	149	202	143	173	*SCP-like extracellular protein family*	Unknown	Unknown
RCAP_rcc02154	2.29	0	936	4973	0.02%	0.07%	1093	1485	4055	5892	*TM2 domain-containing protein*	Unknown	Unknown
RCAP_rcc02155	0.43	0.0033	3313	4489	0.05%	0.06%	3181	3562	3604	5375	*hypothetical protein*	Unknown	Unknown
RCAP_rcc02156	0.52	0.0001	112	161	0.00%	0.00%	97	128	145	177	*hypothetical protein*	Unknown	Unknown
RCAP_rcc02157	-0.09	0.6787	3235	3025	0.05%	0.04%	2641	3839	2530	3519	*purB*	Amino Acid Metabolism	Alanine, aspartate and glutamate metabolism
RCAP_rcc02158	-0.75	0	1383	821	0.02%	0.01%	1224	1483	783	860	*fliG*	Motility	Flagellar Assembly
RCAP_rcc02159	-0.09	0.4926	88	82	0.00%	0.00%	79	96	73	91	*hypothetical protein*	Unknown	Unknown
RCAP_rcc02160	-0.39	0.0095	25469	19356	0.36%	0.26%	21589	29149	17109	21603	*gap3*	Carbohydrate Metabolism	Glycolysis / Gluconeogenesis
RCAP_rcc02161	1.63	0	43	141	0.00%	0.00%	40	54	109	173	*hypothetical protein*	Unknown	Unknown
RCAP_rcc02162	-0.24	0.0056	3518	2971	0.05%	0.04%	3363	3680	2711	3230	*mrcA*	Cell Envelope Biosynthesis	Cell Wall Biosynthesis
RCAP_rcc02163	-0.03	0.8419	1031	1010	0.01%	0.01%	982	1064	879	1141	*ccdA*	Energy Metabolism	Aerobic/Anaerobic Respiration
RCAP_rcc02164	-0.26	0.5719	30	24	0.00%	0.00%	17	41	14	34	*tusA*	Post-translational Modification, Assembly and Chaperones	Unknown
RCAP_rcc02165	-0.3	0.0163	14645	11892	0.20%	0.16%	13204	15623	10347	13437	*rne*	Unknown	Unknown
RCAP_rcc02166	-0.14	0.0495	1584	1437	0.02%	0.02%	1472	1657	1355	1518	*dctD*	Signal Transduction	Transcription Regulator
RCAP_rcc02167	-0.14	0.0862	1413	1282	0.02%	0.02%	1321	1460	1183	1381	*dctB*	Signal Transduction	Kinase/Phosphorelay
RCAP_rcc02168	-0.37	0.0025	1401	1084	0.02%	0.01%	1179	1572	1018	1150	*purQ*	Nucleotide Metabolism	Purine metabolism
RCAP_rcc02169	-0.03	0.8295	960	941	0.01%	0.01%	886	1043	843	1040	*purS*	Nucleotide Metabolism	Purine metabolism
RCAP_rcc02170	-0.19	0.3492	8375	7325	0.12%	0.10%	6950	9831	6144	8507	*purC*	Nucleotide Metabolism	Purine metabolism
RCAP_rcc02171	1.16	0	2535	5775	0.04%	0.08%	2506	3087	5020	6530	*hypothetical protein*	Unknown	Unknown
RCAP_rcc02172	-0.62	0.1298	727	435	0.01%	0.01%	303	1105	226	644	*hypothetical protein*	Unknown	Unknown
RCAP_rcc02173	-0.04	0.7986	1235	1203	0.02%	0.02%	1043	1420	1130	1275	*metH3*	Amino Acid Metabolism	Cysteine and methionine metabolism
RCAP_rcc02174	0.7	0	227	371	0.00%	0.01%	203	256	313	428	*radical SAM family protein*	Replication, Recombination and Repair	Unknown
RCAP_rcc02175	0.26	0.3409	4329	5246	0.06%	0.07%	3349	5470	4215	6277	*mtbC*	Amino Acid Metabolism	Unknown
RCAP_rcc02176	1.23	0	298	729	0.00%	0.01%	283	354	522	935	*hypothetical protein*	Unknown	Unknown
RCAP_rcc02177	1.52	0	561	1685	0.01%	0.02%	539	739	1272	2098	*hypothetical protein*	Unknown	Unknown
RCAP_rcc02178	1	0	104	215	0.00%	0.00%	95	127	153	276	*hypothetical protein*	Unknown	Unknown
RCAP_rcc02179	0.33	0.2362	72	92	0.00%	0.00%	51	94	76	107	*AzlC protein family*	Unknown	Unknown
RCAP_rcc02180	-0.42	0.2596	14	9	0.00%	0.00%	8	18	5	14	*hypothetical protein*	Unknown	Unknown
RCAP_rcc02181	-0.52	0.0076	523	361	0.01%	0.00%	431	582	288	434	*GntR family transcriptional regulator*	Signal Transduction	Transcription Regulator
RCAP_rcc02182	-0.8	0.0004	4698	2622	0.06%	0.04%	3873	5161	1814	3430	*class III aminotransferase*	Carbohydrate Metabolism	Propanoate metabolism
RCAP_rcc02183	-0.51	0.0171	371	257	0.01%	0.00%	293	426	202	311	*potG2*	Amino Acid Metabolism	Amino Acid Transport
RCAP_rcc02184	-1.07	0	332	154	0.00%	0.00%	269	372	122	186	*potH2*	Metal and Ion Transport	Unknown
RCAP_rcc02185	-1.2	0	512	217	0.01%	0.00%	414	576	173	261	*potI3*	Unknown	Unknown
RCAP_rcc02186	-2.24	0	5583	1066	0.07%	0.01%	4256	6417	733	1399	*potF*	Carbohydrate Metabolism	Unknown
RCAP_rcc02187	-2.63	0	1386	197	0.02%	0.00%	900	1746	145	249	*amidohydrolase*	Unknown	Unknown
RCAP_rcc02188	-0.03	0.9103	670	656	0.01%	0.01%	530	793	539	772	*diguanylate cyclase/phosphodiesterase*	Signal Transduction	Kinase/Phosphorelay
RCAP_rcc02189	-0.27	0.0671	1198	993	0.02%	0.01%	999	1383	920	1067	*hypothetical protein*	Unknown	Unknown

RCAP_rcc02190	0.43	0.2232	801	1117	0.01%	0.02%	504	1089	654	1580	*zntA2*	Metal, Ion, Cofactor Transport	Zinc Transport
RCAP_rcc02191	0.11	0.6268	100	108	0.00%	0.00%	81	114	84	131	*MerR family transcriptional regulator*	Signal Transduction	Transcription Regulator
RCAP_rcc02192	-0.04	0.6366	822	798	0.01%	0.01%	768	854	747	850	*major facilitator superfamily protein*	Unknown	Unknown
RCAP_rcc02193	0.94	0.0052	77	160	0.00%	0.00%	52	100	92	228	*DNA-3-methyladenine glycosylase II*	Replication, Recombination and Repair	Replication
RCAP_rcc02194	0.26	0.1413	444	533	0.01%	0.01%	371	516	481	585	*phospholipase/carboxylesterase*	Unknown	Unknown
RCAP_rcc02195	1.41	0	210	567	0.00%	0.01%	198	251	498	636	*HNH endonuclease*	Unknown	Unknown
RCAP_rcc02196	-0.1	0.488	321	299	0.00%	0.00%	278	352	267	330	*FkbM family methyltransferase*	Unknown	Unknown
RCAP_rcc02197	2.05	0	110	466	0.00%	0.01%	103	135	409	523	*two component AraC family transcriptional regulator*	Signal Transduction	Transcription Regulator
RCAP_rcc02198	1.52	0	378	1184	0.01%	0.02%	261	564	1081	1286	*histidine kinase*	Signal Transduction	Kinase/Phosphorelay
RCAP_rcc02199	0.68	0.0003	32	51	0.00%	0.00%	29	36	41	61	*pflA1*	Post-translational Modification, Assembly and Chaperones	Propanediol Metabolism
RCAP_rcc02200	1.27	0	6	14	0.00%	0.00%	5	7	11	18	*hypothetical protein*	Energy Metabolism	Propanediol Metabolism
RCAP_rcc02201	1.12	0	60	133	0.00%	0.00%	58	70	107	158	*adhE*	Energy Metabolism	Propanediol Metabolism
RCAP_rcc02202	1.52	0.0004	1	5	0.00%	0.00%	1	2	2	8	*hypothetical protein*	Energy Metabolism	Propanediol Metabolism
RCAP_rcc02203	1.67	0	2	9	0.00%	0.00%	2	3	7	11	*eutN*	Energy Metabolism	Propanediol Metabolism
RCAP_rcc02204	2.03	0	1	6	0.00%	0.00%	1	1	2	9	*hypothetical protein*	Energy Metabolism	Propanediol Metabolism
RCAP_rcc02205	1.88	0	3	18	0.00%	0.00%	2	6	9	27	*eutJ*	Energy Metabolism	Propanediol Metabolism
RCAP_rcc02206	1.64	0	13	44	0.00%	0.00%	13	17	32	56	*pduL*	Energy Metabolism	Propanediol Metabolism
RCAP_rcc02207	2.05	0	5	27	0.00%	0.00%	5	8	16	38	*pduA1*	Energy Metabolism	Propanediol Metabolism
RCAP_rcc02208	2.35	0	21	128	0.00%	0.00%	20	28	81	174	*pduB*	Energy Metabolism	Propanediol Metabolism
RCAP_rcc02209	1.52	0	16	49	0.00%	0.00%	16	21	36	62	*pduA2*	Energy Metabolism	Propanediol Metabolism
RCAP_rcc02210	1.83	0	14	55	0.00%	0.00%	13	17	36	74	*adh2*	Energy Metabolism	Propanediol Metabolism
RCAP_rcc02211	2.35	0	133	751	0.00%	0.01%	145	184	526	976	*pflD*	Carbohydrate Metabolism	Butanoate metabolism
RCAP_rcc02212	2.76	0	13	113	0.00%	0.00%	13	20	62	164	*ThiJ/PfpI family protein*	Unknown	Unknown
RCAP_rcc02213	2.83	0	7	64	0.00%	0.00%	9	12	39	88	*hypothetical protein*	Unknown	Unknown
RCAP_rcc02214	1.83	0	17	66	0.00%	0.00%	16	22	48	84	*ackA2*	Energy Metabolism	Reductive carboxylate cycle (CO2 fixation)
RCAP_rcc02215	-0.04	0.7604	382	372	0.01%	0.01%	342	416	334	409	*sulfotransferase*	Unknown	Unknown
RCAP_rcc02216	-1.17	0	215	93	0.00%	0.00%	185	230	80	107	*hypothetical protein*	Unknown	Unknown
RCAP_rcc02217	-0.27	0.04	284	234	0.00%	0.00%	241	314	214	254	*group 1 glycosyl transferase*	Unknown	Unknown
RCAP_rcc02218	2.97	0	6	82	0.00%	0.00%	8	14	51	113	*modB2*	Metal and Ion Transport	Unknown
RCAP_rcc02219	3.36	NA	17	302	0.00%	0.00%	32	42	190	414	*modA2*	Metal and Ion Transport	Unknown
RCAP_rcc02220	2.61	0	4	34	0.00%	0.00%	4	6	22	45	*modC2*	Metal and Ion Transport	Unknown
RCAP_rcc02221	2.15	0	6	38	0.00%	0.00%	5	9	23	52	*guaA2*	Xenobiotics Biodegradation and Metabolism	Drug metabolism - other enzymes
RCAP_rcc02222	0.48	0.0124	17	24	0.00%	0.00%	15	21	20	27	*ntaA*	Unknown	Unknown
RCAP_rcc02223	0.92	0.0003	7	13	0.00%	0.00%	6	9	10	16	*ABC transporter permease*	Metal and Ion Transport	Unknown
RCAP_rcc02224	1.16	0.0001	8	19	0.00%	0.00%	6	12	14	23	*ABC transporter substrate-binding protein*	Metal and Ion Transport	Unknown
RCAP_rcc02225	0.61	0.0219	9	14	0.00%	0.00%	7	11	10	18	*ABC transporter ATP-binding protein*	Metal and Ion Transport	Unknown
RCAP_rcc02226	0.79	0.0395	13	24	0.00%	0.00%	10	23	15	34	*hypothetical protein*	Unknown	Unknown
RCAP_rcc02227	-0.18	NA	17	14	0.00%	0.00%	11	29	5	23	*hypothetical protein*	Unknown	Unknown
RCAP_rcc02228	0.57	NA	28	52	0.00%	0.00%	12	64	17	87	*hypothetical protein*	Unknown	Unknown
RCAP_rcc02229	0.07	0.8953	4	4	0.00%	0.00%	2	6	2	5	*hypothetical protein*	Unknown	Unknown
RCAP_rcc02230	-0.02	0.9723	0	0	0.00%	0.00%	0	1	0	1	*hypothetical protein*	Unknown	Unknown
RCAP_rcc02231	-0.05	0.9319	0	0	0.00%	0.00%	0	0	0	0	*hypothetical protein*	Unknown	Unknown
RCAP_rcc02232	0.09	0.7186	11	11	0.00%	0.00%	10	13	9	14	*FAD dependent oxidoreductase*	Energy Metabolism	Unknown
RCAP_rcc02233	0.07	0.8852	5	5	0.00%	0.00%	3	7	3	6	*hypothetical protein*	Unknown	Unknown
RCAP_rcc02234	0.81	0.0702	2	4	0.00%	0.00%	1	3	2	6	*oxidoreductase/nitrogenase subunit 1*	Energy Metabolism	Nitrogen metabolism
RCAP_rcc02235	1.21	0.0129	1	4	0.00%	0.00%	1	2	1	6	*oxidoreductase/nitrogenase subunit 1*	Energy Metabolism	Nitrogen metabolism
RCAP_rcc02236	0.34	0.1435	8	10	0.00%	0.00%	6	9	9	11	*nifH2*	Energy Metabolism	Nitrogen metabolism
RCAP_rcc02237	-0.06	0.8086	46	44	0.00%	0.00%	38	55	37	51	*radical SAM family protein*	Unknown	Unknown
RCAP_rcc02238	0.19	0.2279	104	118	0.00%	0.00%	93	117	102	134	*xsc*	Metabolism of Other Amino Acids	Taurine and hypotaurine metabolism
RCAP_rcc02239	0.5	0.0033	115	164	0.00%	0.00%	100	131	137	191	*GntR family transcriptional regulator*	Signal Transduction	Transcription Regulator
RCAP_rcc02240	-0.03	0.9142	426	416	0.01%	0.01%	327	521	343	490	*tpa*	Metabolism of Other Amino Acids	Taurine and hypotaurine metabolism
RCAP_rcc02241	0.97	0	17	33	0.00%	0.00%	15	21	28	38	*tauA*	Metal and Ion Transport	Unknown
RCAP_rcc02242	0.32	0.4101	2	3	0.00%	0.00%	2	3	2	3	*tauB*	Metal and Ion Transport	Unknown
RCAP_rcc02243	0.29	0.1701	16	20	0.00%	0.00%	13	20	17	23	*tauC*	Metal and Ion Transport	Unknown
RCAP_rcc02244	0.25	0.2	26	30	0.00%	0.00%	22	30	25	36	*sufS2*	Metabolism of Cofactors, Coenzymes and Vitamins	Thiamine metabolism
RCAP_rcc02245	0.37	0.0243	20	25	0.00%	0.00%	18	23	22	29	*major membrane protein I*	Unknown	Unknown
RCAP_rcc02246	0.48	0.0221	16	22	0.00%	0.00%	14	19	18	27	*cysE2*	Energy Metabolism	Sulfur metabolism
RCAP_rcc02247	0.63	0.2354	3	5	0.00%	0.00%	1	4	1	9	*rhodanese domain-containing protein*	Metal and Ion Transport	Unknown
RCAP_rcc02248	0.03	0.876	387	395	0.01%	0.01%	352	435	346	444	*aldo/keto reductase family oxidoreductase*	Energy Metabolism	Unknown
RCAP_rcc02249	1.15	0	102	240	0.00%	0.00%	82	139	171	309	*AraC family transcriptional regulator*	Signal Transduction	Transcription Regulator
RCAP_rcc02250	0.44	0.0194	71	97	0.00%	0.00%	59	82	82	113	*hypothetical protein*	Unknown	Unknown
RCAP_rcc02251	-0.24	0.5349	4	3	0.00%	0.00%	3	5	2	4	*hypothetical protein*	Unknown	Unknown
RCAP_rcc02252	0.02	0.8722	35	36	0.00%	0.00%	33	38	32	39	*kdpA*	Metal and Ion Transport	Unknown
RCAP_rcc02253	0.23	0.2562	24	28	0.00%	0.00%	21	28	23	33	*kdpB*	Metal and Ion Transport	Unknown
RCAP_rcc02254	0.38	0.1209	8	10	0.00%	0.00%	7	9	8	12	*kdpC*	Metal and Ion Transport	Unknown
RCAP_rcc02255	0.36	0.0774	135	174	0.00%	0.00%	113	155	137	211	*kdpD*	Signal Transduction	Kinase/Phosphorelay
RCAP_rcc02256	0.4	0.041	97	129	0.00%	0.00%	79	115	108	151	*kdpE*	Signal Transduction	Transcription Regulator
RCAP_rcc02257	-0.63	0.023	108	67	0.00%	0.00%	71	137	50	85	*DegT/DnrJ/EryC1/StrS family aminotransferase*	Cell Envelope Biosynthesis	Cell Wall Biosynthesis
RCAP_rcc02258	-0.38	0.0206	975	743	0.01%	0.01%	786	1135	651	835	*transferase hexapeptide repeat domain-containing protein*	Unknown	Unknown
RCAP_rcc02259	-0.45	0.0009	95	69	0.00%	0.00%	81	105	62	75	*WbqC-like protein family*	Unknown	Unknown
RCAP_rcc02260	-0.93	0.0001	197	99	0.00%	0.00%	137	247	84	114	*type 11 family methyltransferase*	Unknown	Unknown
RCAP_rcc02261	-1.26	0	352	145	0.00%	0.00%	310	373	125	165	*hypothetical protein*	Unknown	Unknown
RCAP_rcc02262	-1.33	0	239	91	0.00%	0.00%	169	290	82	100	*hypothetical protein*	Unknown	Unknown
RCAP_rcc02263	-1.09	0	245	110	0.00%	0.00%	177	298	96	125	*hypothetical protein*	Unknown	Unknown
RCAP_rcc02264	-1.05	0.0014	144	63	0.00%	0.00%	85	192	49	78	*hypothetical protein*	Unknown	Unknown
RCAP_rcc02265	-1.8	0	259	72	0.00%	0.00%	217	280	61	84	*short-chain dehydrogenase/reductase family oxidoreductase*	Unknown	Unknown
RCAP_rcc02266	-1.63	0	271	85	0.00%	0.00%	231	295	67	102	*potC2*	Metal and Ion Transport	Unknown

RCAP_rcc02267	-1.73	0	541	153	0.01%	0.00%	448	595	112	194	*potB3*	Metal and Ion Transport	Unknown
RCAP_rcc02268	-2.05	0	1544	341	0.02%	0.00%	1259	1712	245	438	*potD3*	Amino Acid Metabolism	Amino Acid Transport
RCAP_rcc02269	-1.15	0	725	321	0.01%	0.00%	637	775	251	390	*potA3*	Amino Acid Metabolism	Amino Acid Transport
RCAP_rcc02270	0.64	0	134	209	0.00%	0.00%	122	153	184	234	*LuxR family transcriptional regulator*	Signal Transduction	Transcription Regulator
RCAP_rcc02271	-0.32	0.0241	55	43	0.00%	0.00%	48	58	38	49	*speB2*	Amino Acid Metabolism	Arginine and proline metabolism
RCAP_rcc02272	0.64	0.0121	23	38	0.00%	0.00%	19	30	31	44	*LysR family transcriptional regulator*	Signal Transduction	Transcription Regulator
RCAP_rcc02273	0.74	0.0028	19	33	0.00%	0.00%	14	23	25	41	*hypothetical protein*	Unknown	Unknown
RCAP_rcc02274	0.13	0.6305	34	38	0.00%	0.00%	26	41	30	45	*pip*	Amino Acid Metabolism	Arginine and proline metabolism
RCAP_rcc02275	0.12	0.4888	284	310	0.00%	0.00%	240	335	274	347	*oppA2*	Amino Acid Metabolism	Amino Acid Transport
RCAP_rcc02276	-0.05	0.7415	100	97	0.00%	0.00%	87	114	87	106	*oppB2*	Metal and Ion Transport	Unknown
RCAP_rcc02277	-0.17	0.0691	65	58	0.00%	0.00%	60	69	54	62	*oppC2*	Metal and Ion Transport	Unknown
RCAP_rcc02278	-0.01	0.9057	67	66	0.00%	0.00%	62	72	63	69	*oppD2*	Unknown	Unknown
RCAP_rcc02279	0.54	0.0811	5	7	0.00%	0.00%	4	7	6	9	*dmsC*	Energy Metabolism	Aerobic/Anaerobic Respiration
RCAP_rcc02280	0.19	0.4534	7	8	0.00%	0.00%	6	8	7	9	*dmsB*	Energy Metabolism	Aerobic/Anaerobic Respiration
RCAP_rcc02281	0.16	0.4318	75	84	0.00%	0.00%	67	90	69	99	*dmsA*	Energy Metabolism	Aerobic/Anaerobic Respiration
RCAP_rcc02282	0.97	0.0054	26	56	0.00%	0.00%	20	35	26	85	*hypothetical protein*	Unknown	Unknown
RCAP_rcc02283	1.42	0.0003	25	82	0.00%	0.00%	17	37	34	130	*hypothetical protein*	Unknown	Unknown
RCAP_rcc02284	2.29	0	21	157	0.00%	0.00%	16	36	77	238	*hypothetical protein*	Unknown	Unknown
RCAP_rcc02285	2.19	0	85	468	0.00%	0.01%	89	145	320	615	*hypothetical protein*	Unknown	Unknown
RCAP_rcc02286	2.02	0	28	139	0.00%	0.00%	23	38	74	204	*divalent ion symporter family*	Metal and Ion Transport	Replication
RCAP_rcc02287	-0.57	0.0461	629	412	0.01%	0.01%	402	832	352	471	*yieF*	Energy Metabolism	Unknown
RCAP_rcc02288	1.11	0	487	1112	0.01%	0.02%	433	633	745	1479	*hypothetical protein*	Unknown	Unknown
RCAP_rcc02289	0.68	0.0083	533	883	0.01%	0.01%	453	661	620	1145	*two-component response regulator receiver protein*	Signal Transduction	Transcription Regulator
RCAP_rcc02290	1.76	0	89	356	0.00%	0.00%	73	135	237	475	*hypothetical protein*	Unknown	Unknown
RCAP_rcc02291	1.24	0	240	601	0.00%	0.01%	216	299	419	782	*ECF family RNA polymerase sigma factor*	Transcription	Unknown
RCAP_rcc02292	-0.15	0.5635	297	266	0.00%	0.00%	250	335	184	347	*signal transduction histidine kinase*	Signal Transduction	Kinase/Phosphorelay
RCAP_rcc02293	-0.19	0.4186	229	199	0.00%	0.00%	206	254	150	248	*hypothetical protein*	Unknown	Unknown
RCAP_rcc02294	-1.56	0	998	326	0.01%	0.00%	811	1111	265	386	*signal transduction histidine kinase*	Signal Transduction	Kinase/Phosphorelay
RCAP_rcc02295	-0.42	0.0002	1103	820	0.02%	0.01%	979	1183	740	901	*proB*	Amino Acid Metabolism	Arginine and proline metabolism
RCAP_rcc02296	-0.38	0.018	2801	2138	0.04%	0.03%	2420	3161	1832	2444	*GTP-binding protein Obg/CgtA*	Unknown	Unknown
RCAP_rcc02297	1.4	0	197	573	0.00%	0.01%	153	272	427	719	*GNAT family acetyltransferase*	Cell Division	Chromosome Partitioning
RCAP_rcc02298	1.16	0	1292	2932	0.02%	0.04%	1275	1577	2608	3255	*GNAT family acetyltransferase*	Cell Division	Chromosome Partitioning
RCAP_rcc02299	-0.21	0.2949	6759	5831	0.09%	0.08%	5772	7704	4756	6906	*rpmA*	Translation, ribosomal structure and biogenesis	Unknown
RCAP_rcc02300	-0.25	0.1603	6436	5373	0.09%	0.07%	5361	7376	4531	6215	*rplU*	Translation, ribosomal structure and biogenesis	Unknown
RCAP_rcc02301	-0.51	0.0264	803	554	0.01%	0.01%	600	976	456	652	*hypothetical protein*	Unknown	Unknown
RCAP_rcc02302	-0.34	0.0035	2090	1641	0.03%	0.02%	1874	2253	1478	1803	*engA*	Unknown	Unknown
RCAP_rcc02303	-0.25	0.0488	1685	1413	0.02%	0.02%	1467	1837	1248	1579	*PQQ enzyme repeat family protein*	Unknown	Unknown
RCAP_rcc02304	-0.21	0.1506	1033	892	0.01%	0.01%	876	1159	801	982	*hypothetical protein*	Unknown	Unknown
RCAP_rcc02305	0.01	0.9132	2740	2763	0.04%	0.04%	2607	2897	2555	2971	*heme ABC transporter ATP-binding/permease*	Defense Mechanisms	Unknown
RCAP_rcc02306	0.13	0.6142	292	322	0.00%	0.00%	232	344	227	416	*LysM domain-containing protein*	Unknown	Unknown
RCAP_rcc02307	-0.48	0	177	127	0.00%	0.00%	154	192	114	139	*decarboxylase*	Unknown	Unknown
RCAP_rcc02308	-0.38	0.0097	206	157	0.00%	0.00%	172	231	137	177	*rarD*	Unknown	Unknown
RCAP_rcc02309	-2.25	NA	2821	352	0.04%	0.00%	563	4816	307	396	*tnaA*	Amino Acid Metabolism	Tryptophan metabolism
RCAP_rcc02310	-1.63	NA	46	12	0.00%	0.00%	3	85	8	15	*hypothetical protein*	Unknown	Unknown
RCAP_rcc02311	-0.7	0	20764	12642	0.29%	0.17%	16480	24437	10990	14295	*sodB*	Metal and Ion Transport	Unknown
RCAP_rcc02312	-0.28	0.0192	512	421	0.01%	0.01%	452	573	390	452	*hypothetical protein*	Unknown	Unknown
RCAP_rcc02313	-0.3	0.3078	753	603	0.01%	0.01%	489	996	481	726	*soxG*	Amino Acid Metabolism	Glycine, serine and threonine metabolism
RCAP_rcc02314	-0.49	0.0274	15499	10891	0.22%	0.15%	12525	18424	9095	12687	*soxA*	Amino Acid Metabolism	Glycine, serine and threonine metabolism
RCAP_rcc02315	-0.29	0.199	1071	868	0.02%	0.01%	892	1272	718	1018	*soxD*	Amino Acid Metabolism	Glycine, serine and threonine metabolism
RCAP_rcc02316	-0.14	0.5976	3811	3437	0.05%	0.05%	2935	4687	2806	4069	*soxB*	Amino Acid Metabolism	Glycine, serine and threonine metabolism
RCAP_rcc02317	-0.44	0.1906	460	328	0.01%	0.00%	270	620	230	426	*cycH*	Energy Metabolism	Aerobic/Anaerobic Respiration
RCAP_rcc02318	-0.32	0.3206	299	234	0.00%	0.00%	206	386	196	272	*yqgF*	Replication, Recombination and Repair	Unknown
RCAP_rcc02319	-0.6	0	270	178	0.00%	0.00%	247	281	164	191	*hypothetical protein*	Unknown	Unknown
RCAP_rcc02320	0.24	0.0741	314	371	0.00%	0.01%	277	361	342	400	*hypothetical protein*	Unknown	Unknown
RCAP_rcc02321	1.19	0	307	732	0.00%	0.01%	269	407	587	876	*hypothetical protein*	Unknown	Unknown
RCAP_rcc02322	-0.03	0.9172	3007	2949	0.04%	0.04%	2550	3489	2426	3473	*prfC*	Translation, ribosomal structure and biogenesis	Unknown
RCAP_rcc02323	-0.66	0.0012	1747	1086	0.02%	0.01%	1332	2123	951	1220	*hypothetical protein*	Unknown	Unknown
RCAP_rcc02324	0.01	0.9533	1229	1237	0.02%	0.02%	1128	1345	1137	1337	*dgt*	Nucleotide Metabolism	Purine metabolism
RCAP_rcc02325	1.16	0	807	1843	0.01%	0.03%	727	1041	1646	2040	*HesB/YadR/YfhF family protein*	Unknown	Unknown
RCAP_rcc02326	-0.12	0.6086	2078	1909	0.03%	0.03%	1641	2513	1605	2213	*phage integrase*	Replication, Recombination and Repair	Phage Interaction
RCAP_rcc02327	0.1	0.5995	46	49	0.00%	0.00%	37	54	44	53	*HK97 family phage portal protein*	Replication, Recombination and Repair	Phage Interaction
RCAP_rcc02328	0.98	0.0083	2	3	0.00%	0.00%	1	2	2	4	*hypothetical protein*	Unknown	Unknown
RCAP_rcc02329	0.38	0.037	22	28	0.00%	0.00%	18	26	25	31	*HK97 family phage major capsid protein*	Replication, Recombination and Repair	Phage Interaction
RCAP_rcc02330	0.57	0.0047	17	25	0.00%	0.00%	14	20	20	29	*hypothetical protein*	Unknown	Unknown
RCAP_rcc02331	1.15	0.0001	6	13	0.00%	0.00%	4	8	10	17	*HNH nuclease*	Defense Mechanisms	Unknown
RCAP_rcc02332	0.45	0.0853	23	32	0.00%	0.00%	16	30	27	37	*hypothetical protein*	Unknown	Unknown
RCAP_rcc02333	-0.36	0.1635	1828	1409	0.03%	0.02%	1333	2264	1135	1683	*hypothetical protein*	Unknown	Unknown
RCAP_rcc02334	-0.79	0.0024	120	67	0.00%	0.00%	90	143	51	83	*hypothetical protein*	Unknown	Unknown
RCAP_rcc02335	1.01	0.0284	2	4	0.00%	0.00%	1	3	2	7	*hypothetical protein*	Unknown	Unknown
RCAP_rcc02336	1.45	0	3	10	0.00%	0.00%	2	5	8	12	*hypothetical protein*	Unknown	Unknown
RCAP_rcc02337	0.98	0.001	4	9	0.00%	0.00%	3	5	7	11	*hypothetical protein*	Unknown	Unknown
RCAP_rcc02338	0.2	0.4647	42	49	0.00%	0.00%	31	54	40	58	*hypothetical protein*	Unknown	Unknown
RCAP_rcc02339	0.85	0.0004	279	521	0.00%	0.01%	203	371	436	605	*hypothetical protein*	Unknown	Unknown
RCAP_rcc02340	0.87	0.0014	6	10	0.00%	0.00%	4	7	8	13	*phage terminase small subunit*	Replication, Recombination and Repair	Phage Interaction
RCAP_rcc02341	0.56	0.0023	21	30	0.00%	0.00%	18	25	26	34	*phage terminase large subunit*	Replication, Recombination and Repair	Phage Interaction
RCAP_rcc02342	0.44	0.0023	33	44	0.00%	0.00%	30	38	40	49	*hypothetical protein*	Unknown	Unknown
RCAP_rcc02343	-0.1	0.82	2471	2278	0.04%	0.03%	1581	3640	1469	3087	*hypothetical protein*	Unknown	Unknown

RCAP_rcc02344	-0.28	0.4959	1380	1098	0.02%	0.01%	910	1961	495	1701	*hypothetical protein*	Unknown	Unknown
RCAP_rcc02345	-0.83	0.0015	1277	691	0.02%	0.01%	871	1606	551	830	*hypothetical protein*	Unknown	Unknown
RCAP_rcc02346	-0.54	0.0512	625	417	0.01%	0.01%	432	800	340	494	*hypothetical protein*	Unknown	Unknown
RCAP_rcc02347	0.16	0.3892	80	89	0.00%	0.00%	69	90	73	105	*hypothetical protein*	Unknown	Unknown
RCAP_rcc02348	-0.09	0.4123	557	525	0.01%	0.01%	496	606	496	553	*cold-shock DNA-binding domain-containing protein*	Transcription	Unknown
RCAP_rcc02349	0.27	0	756	917	0.01%	0.01%	718	808	872	961	*pdxH*	Metabolism of Cofactors, Coenzymes and Vitamins	Vitamin B6 metabolism
RCAP_rcc02350	0.03	0.9273	3831	3917	0.05%	0.05%	2829	4825	3174	4659	*fabI1*	Lipid Metabolism	Fatty acid biosynthesis
RCAP_rcc02351	-0.24	0.24	619	520	0.01%	0.01%	497	735	455	584	*LysE family transporter*	Unknown	Unknown
RCAP_rcc02352	-0.15	0.4655	1086	972	0.02%	0.01%	895	1269	829	1115	*gpt*	Nucleotide Metabolism	Purine metabolism
RCAP_rcc02353	0.28	0.037	312	380	0.00%	0.01%	271	350	342	418	*class I/II aminotransferase*	Amino Acid Metabolism	Unknown
RCAP_rcc02354	0.08	0.573	1962	2073	0.03%	0.03%	1727	2214	1918	2227	*ppiD*	Post-translational Modification, Assembly and Chaperones	Unknown
RCAP_rcc02355	-0.23	0.0083	2977	2528	0.04%	0.03%	2727	3189	2371	2684	*trpE*	Amino Acid Metabolism	Phenylalanine, tyrosine and tryptophan biosynthesis
RCAP_rcc02356	0.13	0.7396	171	188	0.00%	0.00%	98	237	112	264	*hypothetical protein*	Unknown	Unknown
RCAP_rcc02357	0.2	0.0741	677	780	0.01%	0.01%	605	761	735	825	*trpG*	Metabolism of Cofactors, Coenzymes and Vitamins	Folate biosynthesis
RCAP_rcc02358	0.13	0.4647	902	992	0.01%	0.01%	752	1039	872	1112	*trpD*	Amino Acid Metabolism	Phenylalanine, tyrosine and tryptophan biosynthesis
RCAP_rcc02359	-0.17	0.1581	824	731	0.01%	0.01%	740	902	662	799	*trpC*	Amino Acid Metabolism	Phenylalanine, tyrosine and tryptophan biosynthesis
RCAP_rcc02360	-0.44	0	841	619	0.01%	0.01%	760	903	575	664	*moaC1*	Metabolism of Cofactors, Coenzymes and Vitamins	Unknown
RCAP_rcc02361	0.24	0.1447	886	1049	0.01%	0.01%	754	1027	917	1181	*lexA*	Transcription	Unknown
RCAP_rcc02362	1.16	0.0002	102	249	0.00%	0.00%	77	135	162	337	*competence protein*	Unknown	Unknown
RCAP_rcc02363	-0.15	0.337	1688	1517	0.02%	0.02%	1405	1939	1388	1647	*gltX2*	Translation, ribosomal structure and biogenesis	Aminoacyl-tRNA biosynthesis
RCAP_rcc02364	-0.23	0.2076	11409	9685	0.16%	0.13%	9233	13291	8279	11092	*gltA*	Carbohydrate Metabolism	TCA Cycle
RCAP_rcc02365	-0.04	0.8453	396	386	0.01%	0.01%	342	451	346	425	*mdoG2*	Unknown	Unknown
RCAP_rcc02366	1.56	0	123	381	0.00%	0.01%	127	158	271	492	*NnrS family protein*	Metal and Ion Transport	Unknown
RCAP_rcc02367	2.73	0	153	1604	0.00%	0.02%	99	343	1102	2105	*calcium-binding protein*	Unknown	Unknown
RCAP_rcc02368	3.14	0	80	949	0.00%	0.01%	78	107	493	1404	*gph4*	Carbohydrate Metabolism	Starch and sucrose metabolism
RCAP_rcc02369	3.19	NA	230	2767	0.00%	0.04%	237	289	1469	4066	*araB*	Carbohydrate Metabolism	Pentose and glucuronate interconversions
RCAP_rcc02370	3.39	0	218	3093	0.00%	0.04%	221	298	1650	4536	*rbtD*	Carbohydrate Metabolism	Pentose and glucuronate interconversions
RCAP_rcc02371	3.28	NA	380	4894	0.01%	0.07%	397	493	2681	7106	*monosacharide ABC transporter permease*	Carbohydrate Metabolism	Aerobic/Anaerobic Respiration
RCAP_rcc02372	3.82	0	206	4201	0.00%	0.06%	217	294	2187	6216	*monosacharide ABC transporter ATP-binding protein*	Carbohydrate Metabolism	Aerobic/Anaerobic Respiration
RCAP_rcc02373	4.1	0	1350	36214	0.03%	0.49%	2092	2717	21140	51288	*monosacharide ABC transporter periplasmic monosacharide-binding p*	Carbohydrate Metabolism	Aerobic/Anaerobic Respiration
RCAP_rcc02374	3.12	0	96	914	0.00%	0.01%	98	130	738	1091	*LacI family transcriptional regulator*	Signal Transduction	Transcription Regulator
RCAP_rcc02375	-0.94	0.0066	330	157	0.00%	0.00%	179	456	99	214	*exbB*	Trafficking and Secretion	Trafficing
RCAP_rcc02376	-1.85	0	338	90	0.00%	0.00%	280	370	71	109	*exbD1*	Trafficking and Secretion	Trafficing
RCAP_rcc02377	-1.63	0	229	61	0.00%	0.00%	110	326	34	88	*tonB*	Cell Envelope Biosynthesis	Cell Wall Biosynthesis
RCAP_rcc02378	-0.84	NA	4	2	0.00%	0.00%	0	8	0	3	*hypothetical protein*	Unknown	Unknown
RCAP_rcc02379	-0.84	0	125	68	0.00%	0.00%	101	143	58	78	*CobW/HypB/UreG family cobalamin biosynthesis protein*	Metabolism of Cofactors, Coenzymes and Vitamins	Cobalamin Biosynthesis
RCAP_rcc02380	-0.19	0.0078	3599	3145	0.05%	0.04%	3330	3789	2967	3324	*ftsI*	Glycan Biosynthesis and Metabolism	Peptidoglycan biosynthesis
RCAP_rcc02381	0.03	0.824	480	492	0.01%	0.01%	441	525	444	540	*hypothetical protein*	Unknown	Unknown
RCAP_rcc02382	0.21	0.1701	347	401	0.00%	0.01%	296	399	370	433	*mraW*	Cell Envelope Biosynthesis	Cell Wall Biosynthesis
RCAP_rcc02383	-0.05	0.815	1551	1499	0.02%	0.02%	1289	1798	1354	1643	*mraZ*	Unknown	Unknown
RCAP_rcc02384	0.05	0.5967	746	773	0.01%	0.01%	693	802	727	818	*Mrp/NBP35 family protein*	Cell Division	Chromosome Partitioning
RCAP_rcc02385	1.99	0	1112	4843	0.02%	0.07%	1055	1687	4031	5654	*hypothetical protein*	Unknown	Unknown
RCAP_rcc02386	0.46	0.0001	428	592	0.01%	0.01%	390	485	530	655	*hypothetical protein*	Unknown	Unknown
RCAP_rcc02387	0.11	0.3379	656	709	0.01%	0.01%	597	743	661	758	*hypothetical protein*	Unknown	Unknown
RCAP_rcc02388	0.59	0	1231	1862	0.02%	0.03%	1195	1448	1625	2098	*ispZ*	Cell Division	Chromosome Partitioning
RCAP_rcc02389	0.65	0	865	1370	0.01%	0.02%	766	989	1221	1520	*ftsY*	Trafficking and Secretion	Trafficing
RCAP_rcc02390	3.7	0	372	5315	0.01%	0.07%	639	736	4330	6300	*alkane 1-monooxygenase*	Metabolism of Cofactors, Coenzymes and Vitamins	Retinol metabolism
RCAP_rcc02391	0.23	0.1715	375	441	0.01%	0.01%	315	443	402	479	*hypothetical protein*	Unknown	Unknown
RCAP_rcc02392	0.33	0.1039	397	505	0.01%	0.01%	316	470	413	596	*xseA*	Replication, Recombination and Repair	Replication
RCAP_rcc02393	-0.1	0.6442	428	399	0.01%	0.01%	332	519	362	436	*purD*	Nucleotide Metabolism	Purine metabolism
RCAP_rcc02394	-0.46	0.0678	888	632	0.01%	0.01%	636	1102	499	766	*sdaA*	Amino Acid Metabolism	Cysteine and methionine metabolism
RCAP_rcc02395	-0.09	0.8004	124	116	0.00%	0.00%	79	164	91	141	*hypothetical protein*	Unknown	Unknown
RCAP_rcc02396	-0.18	0.2485	2060	1815	0.03%	0.02%	1743	2353	1611	2019	*rpiA*	Carbohydrate Metabolism	Pentose phosphate pathway
RCAP_rcc02397	0.28	0.0085	569	693	0.01%	0.01%	516	630	644	743	*hypothetical protein*	Unknown	Unknown
RCAP_rcc02398	-0.21	0.0046	1530	1324	0.02%	0.02%	1425	1627	1256	1393	*gor*	Sulfur Metabolism	Glutathione metabolism
RCAP_rcc02399	0.91	0	4489	8602	0.07%	0.12%	4141	5588	7469	9734	*hflK*	Post-translational Modification, Assembly and Chaperones	Unknown
RCAP_rcc02400	0.58	0.0001	5164	7769	0.08%	0.11%	4941	6030	6652	8885	*hflC*	Post-translational Modification, Assembly and Chaperones	Unknown
RCAP_rcc02401	0.64	0.0022	79	125	0.00%	0.00%	63	97	102	148	*hypothetical protein*	Unknown	Unknown
RCAP_rcc02402	0.7	0	3993	6522	0.06%	0.09%	3990	4466	5901	7143	*degP*	Unknown	Unknown
RCAP_rcc02403	0.95	0.0065	32	69	0.00%	0.00%	20	46	42	96	*peptidoglycan-binding domain 1 protein*	Unknown	Unknown
RCAP_rcc02404	0.04	0.8295	434	447	0.01%	0.01%	348	518	407	488	*fdx*	Energy Metabolism	Aerobic/Anaerobic Respiration
RCAP_rcc02405	-0.45	0.1305	4611	3278	0.06%	0.04%	3331	5821	2502	4054	*purU*	Carbohydrate Metabolism	Glyoxylate and dicarboxylate metabolism
RCAP_rcc02406	-0.14	0.4293	2747	2485	0.04%	0.03%	2286	3115	2064	2905	*pgi*	Carbohydrate Metabolism	Glycolysis / Gluconeogenesis
RCAP_rcc02407	-0.07	0.8244	452	429	0.01%	0.01%	314	568	329	528	*pgl*	Carbohydrate Metabolism	Pentose phosphate pathway
RCAP_rcc02408	-0.25	0.1491	2673	2238	0.04%	0.03%	2195	3058	1872	2603	*zwf*	Carbohydrate Metabolism	Pentose phosphate pathway
RCAP_rcc02409	-0.46	0.0009	90	65	0.00%	0.00%	78	98	57	72	*hypothetical protein*	Unknown	Unknown
RCAP_rcc02410	-0.05	0.9062	221	213	0.00%	0.00%	130	305	170	256	*hypothetical protein*	Unknown	Unknown
RCAP_rcc02411	0.17	0.5746	2264	2569	0.03%	0.03%	1593	2916	1891	3246	*edd*	Carbohydrate Metabolism	Pentose phosphate pathway
RCAP_rcc02412	0.3	0.2093	823	1024	0.01%	0.01%	619	1035	844	1204	*eda*	Carbohydrate Metabolism	Glyoxylate and dicarboxylate metabolism
RCAP_rcc02413	0.48	0.0038	776	1093	0.01%	0.01%	750	915	898	1287	*hypothetical protein*	Unknown	Unknown
RCAP_rcc02414	0.49	0.0002	857	1205	0.01%	0.02%	771	946	1044	1367	*glnE*	Unknown	Unknown
RCAP_rcc02415	4.27	0	684	21435	0.02%	0.29%	1330	1885	15237	27633	*hypothetical protein*	Unknown	Unknown
RCAP_rcc02416	0.28	0.0489	2743	3332	0.04%	0.05%	2440	3163	2977	3687	*ate*	Post-translational Modification, Assembly and Chaperones	Unknown
RCAP_rcc02417	-0.33	0.0292	13624	10822	0.19%	0.15%	11712	15275	9477	12168	*dctP2*	Secondary metabolites biosynthesis, transport, and catabolism	Unknown
RCAP_rcc02418	0.2	0.0866	408	470	0.01%	0.01%	377	464	433	507	*short-chain dehydrogenase/reductase family oxidoreductase*	Lipid transport and metabolism	Unknown
RCAP_rcc02419	0.19	0.2287	184	211	0.00%	0.00%	172	212	182	240	*dihydroneopterin aldolase*	Unknown	Unknown
RCAP_rcc02420	0.34	0.2539	323	418	0.00%	0.01%	212	434	321	515	*hypothetical protein*	Unknown	Unknown

RCAP_rcc02421	0.68	0	1622	2615	0.02%	0.04%	1553	1772	2403	2828	*bglX*	Photosynthesis	Phenylpropanoid biosynthesis
RCAP_rcc02422	-0.1	0.3576	438	408	0.01%	0.01%	398	473	374	441	*hypothetical protein*	Unknown	Unknown
RCAP_rcc02423	0.58	0.0004	549	825	0.01%	0.01%	480	617	680	971	*ABC transporter ATP-binding protein/permease*	Unknown	Unknown
RCAP_rcc02424	0.05	0.9035	248	258	0.00%	0.00%	134	359	213	302	*short-chain dehydrogenase/reductase family oxidoreductase*	Metabolism of Cofactors, Coenzymes and Vitamins	Biotin metabolism
RCAP_rcc02425	0.34	0.1816	231	297	0.00%	0.00%	167	294	242	352	*surE*	Metabolism of Cofactors, Coenzymes and Vitamins	Nicotinate and nicotinamide metabolism
RCAP_rcc02426	0.27	0.1124	311	378	0.00%	0.01%	262	368	345	411	*pcm1*	Post-translational Modification, Assembly and Chaperones	Unknown
RCAP_rcc02427	0.28	0.166	365	445	0.01%	0.01%	299	429	380	510	*M23 family peptidase*	Post-translational Modification, Assembly and Chaperones	Peptidase
RCAP_rcc02428	0.14	0.0463	2469	2732	0.03%	0.04%	2328	2637	2593	2870	*TrkA domain transport protein*	Metal and Ion Transport	Unknown
RCAP_rcc02429	-0.01	0.9371	759	751	0.01%	0.01%	642	862	682	819	*xthA1*	Replication, Recombination and Repair	Unknown
RCAP_rcc02430	0.67	0.0081	559	920	0.01%	0.01%	399	722	772	1068	*nahG*	Xenobiotics Biodegradation and Metabolism	Naphthalene and anthracene degradation
RCAP_rcc02431	-0.05	0.7834	1165	1121	0.02%	0.02%	959	1387	1026	1216	*dksA1*	Signal Transduction	Kinase/Phosphorelay
RCAP_rcc02432	-0.17	0.25	1254	1113	0.02%	0.02%	1071	1401	989	1237	*ATPase AAA*	Unknown	Unknown
RCAP_rcc02433	-0.38	0.0042	470	360	0.01%	0.00%	401	522	328	393	*hypothetical protein*	Unknown	Unknown
RCAP_rcc02434	-0.4	0.0388	410	306	0.01%	0.00%	317	485	257	356	*cephalosporin hydroxylase*	Defense Mechanisms	Unknown
RCAP_rcc02435	-0.11	0.6805	783	726	0.01%	0.01%	595	953	633	818	*family 2 glycosyl transferase*	Unknown	Unknown
RCAP_rcc02436	-0.36	0.0182	530	412	0.01%	0.01%	449	606	363	460	*phosphopantetheine-binding domain-containing protein*	Unknown	Unknown
RCAP_rcc02437	0.23	0.3658	468	551	0.01%	0.01%	350	580	453	650	*cytochrome P450 family protein*	Energy Metabolism	Aerobic/Anaerobic Respiration
RCAP_rcc02438	-0.04	0.8776	315	305	0.00%	0.00%	247	369	233	377	*AMP-dependent synthetase and ligase*	Lipid Metabolism	Fatty acid metabolism
RCAP_rcc02439	0.27	0.0336	317	382	0.00%	0.01%	286	338	333	430	*hypothetical protein*	Unknown	Unknown
RCAP_rcc02440	-0.18	0.6907	241	207	0.00%	0.00%	146	397	99	316	*AsnC/Lrp family transcriptional regulator*	Signal Transduction	Transcription Regulator
RCAP_rcc02441	0.29	0.1046	104	127	0.00%	0.00%	84	124	117	137	*GntR family transcriptional regulator*	Signal Transduction	Transcription Regulator
RCAP_rcc02442	-0.38	0.0002	366	280	0.01%	0.00%	324	400	262	299	*livJ*	Amino Acid Metabolism	Amino Acid Transport
RCAP_rcc02443	-0.66	0	299	188	0.00%	0.00%	250	337	169	207	*livH2*	Amino Acid Metabolism	Amino Acid Transport
RCAP_rcc02444	-0.42	0.0085	95	70	0.00%	0.00%	82	108	61	80	*livM2*	Amino Acid Metabolism	Amino Acid Transport
RCAP_rcc02445	-0.38	0.008	77	59	0.00%	0.00%	66	88	53	65	*livG2*	Amino Acid Metabolism	Amino Acid Transport
RCAP_rcc02446	0.05	0.8119	57	59	0.00%	0.00%	47	68	52	66	*livF2*	Amino Acid Metabolism	Amino Acid Transport
RCAP_rcc02447	-0.14	0.5698	76	68	0.00%	0.00%	60	92	55	82	*hyuE*	Amino Acid Metabolism	Unknown
RCAP_rcc02448	-2.45	0	1269	211	0.02%	0.00%	815	1614	182	240	*aldH2*	Energy Metabolism	Limonene and pinene degradation
RCAP_rcc02449	-1.95	0	209	50	0.00%	0.00%	131	271	45	55	*potC3*	Metal and Ion Transport	Unknown
RCAP_rcc02450	-2.22	0	325	64	0.00%	0.00%	220	404	53	75	*potB4*	Metal and Ion Transport	Unknown
RCAP_rcc02451	-2.77	0	297	38	0.00%	0.00%	180	388	33	43	*potA4*	Amino Acid Metabolism	Amino Acid Transport
RCAP_rcc02452	-2.66	0	1694	229	0.02%	0.00%	952	2284	189	270	*potD4*	Amino Acid Metabolism	Amino Acid Transport
RCAP_rcc02453	-1.2	0.0001	92	36	0.00%	0.00%	61	116	22	51	*Fis family sigma54 specific transcriptional regulator*	Signal Transduction	Transcription Regulator
RCAP_rcc02454	-0.12	0.7687	61	55	0.00%	0.00%	35	84	36	75	*PAS domain-containing protein*	Unknown	Unknown
RCAP_rcc02455	0.49	0.1948	102	151	0.00%	0.00%	62	137	93	209	*FAD dependent oxidoreductase*	Amino Acid Metabolism	Unknown
RCAP_rcc02456	-0.16	0.2133	1135	1011	0.02%	0.01%	985	1268	936	1086	*lolE*	Cell Envelope Biosynthesis	Cell Wall Biosynthesis
RCAP_rcc02457	0.1	0.6661	249	267	0.00%	0.00%	200	302	242	293	*lolD2*	Defense Mechanisms	Unknown
RCAP_rcc02458	-0.17	0.2133	179	157	0.00%	0.00%	154	197	141	173	*ArsC family protein*	Metal and Ion Transport	Unknown
RCAP_rcc02459	0.43	0.0086	284	385	0.00%	0.01%	256	312	317	452	*XRE family transcriptional regulator*	Signal Transduction	Transcription Regulator
RCAP_rcc02460	1.86	0	4443	16913	0.07%	0.23%	4206	6214	14666	19160	*cspA2*	Transcription	Unknown
RCAP_rcc02461	0.16	0.4647	3850	4308	0.05%	0.06%	3221	4607	3699	4917	*thrS*	Translation, ribosomal structure and biogenesis	Aminoacyl-tRNA biosynthesis
RCAP_rcc02462	2.07	0	1716	7724	0.03%	0.10%	1820	2590	6383	9065	*hypothetical protein*	Unknown	Unknown
RCAP_rcc02463	2.59	0	225	1458	0.00%	0.02%	286	359	1163	1753	*hemolysin-type calcium-binding repeat family protein*	Trafficking and Secretion	Secretion
RCAP_rcc02464	0.36	0.0069	2346	3035	0.03%	0.04%	2122	2689	2709	3360	*proS*	Translation, ribosomal structure and biogenesis	Aminoacyl-tRNA biosynthesis
RCAP_rcc02465	-0.44	0.0185	3947	2882	0.05%	0.04%	3216	4607	2483	3281	*hypothetical protein*	Unknown	Unknown
RCAP_rcc02466	-0.09	0.513	107	101	0.00%	0.00%	94	119	91	110	*hypothetical protein*	Unknown	Unknown
RCAP_rcc02467	0.03	0.9159	171	174	0.00%	0.00%	135	201	143	206	*GntR family transcriptional regulator*	Signal Transduction	Transcription Regulator
RCAP_rcc02468	0.24	0.202	768	909	0.01%	0.01%	625	928	816	1002	*thioesterase superfamily protein*	Lipid Metabolism	Unknown
RCAP_rcc02469	0.15	0.5126	999	1116	0.01%	0.02%	771	1225	933	1300	*ErfK/YbiS/YcfS/YnhG family protein*	Unknown	Unknown
RCAP_rcc02470	-0.27	0.4518	532	432	0.01%	0.01%	334	704	318	545	*selU*	Unknown	Unknown
RCAP_rcc02471	-0.82	0	80	43	0.00%	0.00%	68	89	35	52	*hypothetical protein*	Unknown	Unknown
RCAP_rcc02472	-0.57	0.0043	53	35	0.00%	0.00%	42	60	29	41	*bsaA2*	Sulfur Metabolism	Glutathione metabolism
RCAP_rcc02473	0.23	0.2406	190	223	0.00%	0.00%	159	231	199	248	*TonB-dependent receptor plug domain-containing protein*	Metal and Ion Transport	Unknown
RCAP_rcc02474	-1.03	0	96	46	0.00%	0.00%	74	112	42	51	*hypothetical protein*	Unknown	Unknown
RCAP_rcc02475	-0.93	0	219	114	0.00%	0.00%	191	235	104	125	*hypothetical protein*	Unknown	Unknown
RCAP_rcc02476	-0.49	0.0151	3920	2755	0.05%	0.04%	3148	4655	2308	3202	*ppaC*	Energy Metabolism	Oxidative phosphorylation
RCAP_rcc02477	-0.13	0.6366	9550	8661	0.13%	0.12%	6686	12267	7890	9432	*groS*	Post-translational Modification, Assembly and Chaperones	Unknown
RCAP_rcc02478	-0.28	0.2856	69131	56473	0.96%	0.77%	49840	86872	49898	63048	*groL*	Post-translational Modification, Assembly and Chaperones	Unknown
RCAP_rcc02479	1.88	0	326	1341	0.01%	0.02%	270	462	1018	1664	*lipoprotein*	Predicted Function	Unknown
RCAP_rcc02480	-0.24	0.0707	1939	1635	0.03%	0.02%	1682	2175	1482	1788	*polA*	Replication, Recombination and Repair	Replication
RCAP_rcc02481	-0.44	0.008	320	234	0.00%	0.00%	264	372	200	267	*hypothetical protein*	Unknown	Unknown
RCAP_rcc02482	0.36	0.0002	1082	1396	0.02%	0.02%	1019	1189	1271	1521	*HIT family protein*	Nucleotide Metabolism	Pyrimidine metabolism
RCAP_rcc02483	-0.02	0.881	516	510	0.01%	0.01%	468	565	480	540	*family 14 glycosyl transferase*	Unknown	Unknown
RCAP_rcc02484	-0.32	0.1028	2448	1945	0.03%	0.03%	1908	2936	1667	2223	*hypothetical protein*	Unknown	Unknown
RCAP_rcc02485	0.25	0.3093	222	265	0.00%	0.00%	174	263	204	325	*type 12 family methyltransferase*	Unknown	Unknown
RCAP_rcc02486	0.52	0	1365	1957	0.02%	0.03%	1312	1471	1896	2019	*carbohydrate/purine kinase*	Carbohydrate Metabolism	Amino sugar and nucleotide sugar metabolism
RCAP_rcc02487	0.2	0.1303	435	499	0.01%	0.01%	388	472	444	554	*nth*	Replication, Recombination and Repair	Unknown
RCAP_rcc02488	-0.78	0.0011	1261	714	0.02%	0.01%	802	1672	638	790	*hypothetical protein*	Unknown	Unknown
RCAP_rcc02489	-0.96	0	166	83	0.00%	0.00%	134	186	66	101	*ada*	Replication, Recombination and Repair	Unknown
RCAP_rcc02490	0.42	0.0395	1706	2308	0.02%	0.03%	1315	2173	2042	2574	*OmpA family protein*	Cell Envelope Biosynthesis	Cell Wall Biosynthesis
RCAP_rcc02491	0.75	0	1103	1860	0.02%	0.03%	1081	1202	1731	1990	*GntR family transcriptional regulator*	Signal Transduction	Transcription Regulator
RCAP_rcc02492	0.38	0.1587	146	192	0.00%	0.00%	109	179	157	227	*hypothetical protein*	Unknown	Unknown
RCAP_rcc02493	0.2	0.2937	4008	4639	0.06%	0.06%	3260	4860	4104	5175	*Crp/Fnr family transcriptional regulator*	Signal Transduction	Transcription Regulator
RCAP_rcc02494	0.64	0	11161	17528	0.16%	0.24%	10580	12833	15935	19121	*hemN2*	Metabolism of Cofactors, Coenzymes and Vitamins	Heme Biosynthesis
RCAP_rcc02495	1.18	0	213	495	0.00%	0.01%	186	254	417	573	*GTPase, EngC family*	Unknown	Unknown
RCAP_rcc02496	-0.79	0	1130	650	0.02%	0.01%	1022	1181	600	700	*group 1 glycosyl transferase*	Cell Envelope Biosynthesis	Cell Wall Biosynthesis
RCAP_rcc02497	-0.58	0	2199	1467	0.03%	0.02%	1971	2355	1356	1578	*ABC transporter ATP-binding protein*	Unknown	Unknown

RCAP_rcc02498	-0.8	0	1560	890	0.02%	0.01%	1354	1699	795	985	*ABC transporter permease*	Metal and Ion Transport	Unknown
RCAP_rcc02499	-0.76	0	2587	1507	0.04%	0.02%	2114	2972	1328	1685	*ABC transporter permease*	Metal and Ion Transport	Unknown
RCAP_rcc02500	-0.39	0.0002	3863	2946	0.05%	0.04%	3493	4191	2694	3198	*ABC transporter periplasmic substrate-binding protein*	Amino Acid Metabolism	Amino Acid Transport
RCAP_rcc02501	0.73	0.0002	2970	5035	0.04%	0.07%	2555	3695	4144	5927	*cycY*	Energy Metabolism	Aerobic/Anaerobic Respiration
RCAP_rcc02502	-0.32	0.0006	1106	883	0.02%	0.01%	975	1198	835	930	*pheA*	Amino Acid Metabolism	Phenylalanine, tyrosine and tryptophan biosynthesis
RCAP_rcc02503	0.19	0.1996	296	339	0.00%	0.00%	252	341	309	369	*hypothetical protein*	Unknown	Unknown
RCAP_rcc02504	0.35	0.4303	43	58	0.00%	0.00%	17	66	30	85	*nudC*	Metabolism of Cofactors, Coenzymes and Vitamins	Nicotinate and nicotinamide metabolism
RCAP_rcc02505	0.72	0.0001	175	293	0.00%	0.00%	164	200	233	354	*tadA*	Xenobiotics Biodegradation and Metabolism	Atrazine degradation
RCAP_rcc02506	-0.95	0	2269	1139	0.03%	0.02%	1885	2568	872	1407	*rluB*	Nucleotide Metabolism	Pyrimidine metabolism
RCAP_rcc02507	1.56	0	142	439	0.00%	0.01%	125	182	329	549	*hypothetical protein*	Unknown	Unknown
RCAP_rcc02508	0.32	0.0012	212	267	0.00%	0.00%	196	234	247	287	*hypothetical protein*	Unknown	Unknown
RCAP_rcc02509	0.51	0.0015	807	1155	0.01%	0.02%	698	937	1000	1310	*TelA family toxic anion resistance protein*	Unknown	Unknown
RCAP_rcc02510	-0.12	0.337	456	419	0.01%	0.01%	405	499	379	458	*lipoprotein*	Predicted Function	Unknown
RCAP_rcc02511	-0.21	0.1366	1990	1722	0.03%	0.02%	1821	2159	1493	1952	*hypothetical protein*	Unknown	Unknown
RCAP_rcc02512	-0.21	0.2083	844	728	0.01%	0.01%	697	976	647	810	*hypothetical protein*	Unknown	Unknown
RCAP_rcc02513	-0.27	0.0192	945	785	0.01%	0.01%	865	1014	699	872	*cscK*	Carbohydrate Metabolism	Amino sugar and nucleotide sugar metabolism
RCAP_rcc02514	-0.57	0	419	282	0.01%	0.00%	385	438	250	313	*dtd*	Translation, ribosomal structure and biogenesis	Unknown
RCAP_rcc02515	-0.6	0.0213	5354	3431	0.07%	0.05%	4035	6592	2542	4320	*rhlE*	Replication, Recombination and Repair	Unknown
RCAP_rcc02516	0.12	0.6542	370	402	0.01%	0.01%	300	441	291	513	*tdk*	Xenobiotics Biodegradation and Metabolism	Drug metabolism - other enzymes
RCAP_rcc02517	1.68	0	77	296	0.00%	0.00%	47	117	154	438	*trxC*	Post-translational Modification, Assembly and Chaperones	Unknown
RCAP_rcc02518	0.06	0.6625	468	490	0.01%	0.01%	417	529	446	534	*type 12 family methyltransferase*	Unknown	Unknown
RCAP_rcc02519	-0.08	0.7622	702	659	0.01%	0.01%	538	831	493	825	*diguanylate cyclase/phosphodiesterase*	Signal Transduction	Kinase/Phosphorelay
RCAP_rcc02520	-0.05	0.9102	287	277	0.00%	0.00%	141	426	204	350	*integrin alpha repeat/hemolysin-type calcium-binding repeat family pro*	Trafficking and Secretion	Secretion
RCAP_rcc02521	-1.38	0	9635	3457	0.13%	0.05%	5202	13576	2878	4035	*pyrimidine ABC transporter periplasmic pyrimidine-binding protein*	Metal and Ion Transport	Unknown
RCAP_rcc02522	-0.67	NA	1018	608	0.01%	0.01%	397	1605	523	694	*pyrimidine ABC transporter permease*	Metal and Ion Transport	Unknown
RCAP_rcc02523	-0.53	NA	1517	1015	0.02%	0.01%	619	2370	866	1164	*pyrimidine ABC transporter permease*	Metal and Ion Transport	Unknown
RCAP_rcc02524	-0.41	0.2059	2729	1993	0.04%	0.03%	1218	4183	1642	2345	*pyrimidine ABC transporter ATP-binding protein*	Metal and Ion Transport	Unknown
RCAP_rcc02525	-0.3	0.4312	3053	2408	0.04%	0.03%	1085	4987	1912	2903	*dht*	Metabolism of Cofactors, Coenzymes and Vitamins	Pantothenate and CoA biosynthesis
RCAP_rcc02526	-0.02	0.9604	2453	2414	0.03%	0.03%	896	3969	2076	2752	*amaB*	Xenobiotics Biodegradation and Metabolism	Drug metabolism - other enzymes
RCAP_rcc02527	0.14	0.3373	1427	1576	0.02%	0.02%	1259	1602	1402	1751	*XRE family transcriptional regulator*	Signal Transduction	Transcription Regulator
RCAP_rcc02528	0.03	NA	2885	2962	0.04%	0.04%	-213	5930	1999	3925	*dihydropyrimidine dehydrogenase*	Metabolism of Cofactors, Coenzymes and Vitamins	Pantothenate and CoA biosynthesis
RCAP_rcc02529	0.43	NA	1263	1879	0.02%	0.03%	7	2493	1121	2636	*pyridine nucleotide-disulfide oxidoreductase*	Unknown	Unknown
RCAP_rcc02530	3.1	0	22813	212493	0.46%	2.89%	27752	37074	183290	241695	*pucB*	Photosynthesis	Light Harvesting Machinery
RCAP_rcc02531	3.45	0	10822	126140	0.24%	1.71%	15278	19572	112766	139513	*pucA*	Photosynthesis	Light Harvesting Machinery
RCAP_rcc02532	2.15	0	3730	17291	0.06%	0.23%	3821	5083	14998	19585	*pucC2*	Photosynthesis	Light Harvesting Machinery
RCAP_rcc02533	2.49	0	11763	69872	0.22%	0.95%	13357	17382	59798	79946	*pucDE*	Photosynthesis	Light Harvesting Machinery
RCAP_rcc02534	0.74	0.0014	27	46	0.00%	0.00%	20	36	40	52	*cysA*	Metal and Ion Transport	Sulfute
RCAP_rcc02535	0.26	0.3671	74	90	0.00%	0.00%	53	107	75	104	*cysW*	Metal and Ion Transport	Sulfute
RCAP_rcc02536	0.19	0.619	158	184	0.00%	0.00%	117	251	111	257	*cysT*	Metal and Ion Transport	Sulfute
RCAP_rcc02537	0.07	0.731	1172	1234	0.02%	0.02%	927	1424	1133	1335	*cysK1*	Energy Metabolism	Sulfur metabolism
RCAP_rcc02538	0.52	0.0003	3706	5374	0.05%	0.07%	3559	4223	4571	6176	*trpB2*	Amino Acid Metabolism	Phenylalanine, tyrosine and tryptophan biosynthesis
RCAP_rcc02539	-0.73	0	1209	728	0.02%	0.01%	1097	1259	679	776	*diguanylate cyclase/phosphodiesterase*	Signal Transduction	Kinase/Phosphorelay
RCAP_rcc02540	-0.22	0.0268	1760	1514	0.02%	0.02%	1598	1866	1384	1644	*diguanylate cyclase/phosphodiesterase*	Signal Transduction	Kinase/Phosphorelay
RCAP_rcc02541	-0.7	0.0305	408	237	0.01%	0.00%	219	589	182	291	*fruA*	Carbohydrate Metabolism	Fructose and mannose metabolism
RCAP_rcc02542	0.46	NA	20	31	0.00%	0.00%	8	31	6	55	*fruK*	Carbohydrate Metabolism	Fructose and mannose metabolism
RCAP_rcc02543	1.09	0.0048	109	268	0.00%	0.00%	63	163	116	420	*fruB*	Carbohydrate Metabolism	Fructose and mannose metabolism
RCAP_rcc02544	0.12	0.7116	176	193	0.00%	0.00%	122	226	147	239	*ABC transporter substrate-binding protein*	Carbohydrate Metabolism	Unknown
RCAP_rcc02545	-0.3	0.117	349	281	0.00%	0.00%	284	397	230	332	*sensor histidine kinase/response regulator receiver protein*	Signal Transduction	Transcription Regulator
RCAP_rcc02546	-0.44	0.0006	244	179	0.00%	0.00%	215	262	155	203	*winged helix family two component transcriptional regulator*	Signal Transduction	Transcription Regulator
RCAP_rcc02547	-0.57	0.0031	191	127	0.00%	0.00%	159	215	103	151	*hypothetical protein*	Unknown	Unknown
RCAP_rcc02548	-0.29	0.0752	1038	843	0.01%	0.01%	865	1187	741	945	*rng*	Unknown	Unknown
RCAP_rcc02549	-0.35	0.0132	753	588	0.01%	0.01%	660	834	508	668	*maf*	Cell Division	Chromosome Partitioning
RCAP_rcc02550	-0.29	0.225	1457	1178	0.02%	0.02%	1128	1796	948	1407	*infA*	Translation, ribosomal structure and biogenesis	Unknown
RCAP_rcc02551	-0.5	0.0002	720	506	0.01%	0.01%	653	764	432	581	*carbon-nitrogen family hydrolase*	Predicted Function	Nitrogen metabolism
RCAP_rcc02552	-0.05	0.8776	77	74	0.00%	0.00%	57	96	61	87	*GNAT family acetyltransferase*	Cell Division	Chromosome Partitioning
RCAP_rcc02553	0.01	0.9618	573	578	0.01%	0.01%	456	685	498	657	*hypothetical protein*	Unknown	Unknown
RCAP_rcc02554	0.98	0.0044	73	158	0.00%	0.00%	46	99	98	218	*NmrA family protein*	Unknown	Unknown
RCAP_rcc02555	-0.16	0.1891	124	111	0.00%	0.00%	109	138	104	118	*HxlR family transcriptional regulator*	Signal Transduction	Transcription Regulator
RCAP_rcc02556	-0.71	0.0008	782	467	0.01%	0.01%	593	938	388	546	*arsC*	Signal Transduction	Kinase/Phosphorelay
RCAP_rcc02557	-0.29	0.0036	456	372	0.01%	0.01%	422	483	338	407	*uncharacterized protein family UPF0262*	Unknown	Unknown
RCAP_rcc02558	0.11	0.6366	842	913	0.01%	0.01%	649	1030	816	1011	*hisD*	Amino Acid Metabolism	Histidine metabolism
RCAP_rcc02559	-0.01	0.9853	176	174	0.00%	0.00%	114	228	129	220	*GNAT family acetyltransferase*	Cell Division	Chromosome Partitioning
RCAP_rcc02560	0.05	0.7553	688	713	0.01%	0.01%	592	787	655	771	*hypothetical protein*	Unknown	Unknown
RCAP_rcc02561	-0.3	0.0091	2523	2051	0.04%	0.03%	2215	2794	1912	2190	*murA*	Glycan Biosynthesis and Metabolism	Peptidoglycan biosynthesis
RCAP_rcc02562	0.81	0.0383	23	44	0.00%	0.00%	12	33	24	64	*hypothetical protein*	Unknown	Unknown
RCAP_rcc02563	1.24	0	25	63	0.00%	0.00%	23	37	51	76	*hypothetical protein*	Unknown	Unknown
RCAP_rcc02564	1.56	0	17	60	0.00%	0.00%	12	31	42	79	*hypothetical protein*	Unknown	Unknown
RCAP_rcc02565	1.33	0.0001	546	1566	0.01%	0.02%	368	953	1191	1941	*hypothetical protein*	Unknown	Unknown
RCAP_rcc02566	1.07	0	2308	5044	0.04%	0.07%	1929	3113	4264	5825	*reverse transcriptase catalytic domain-containing protein*	Unknown	Unknown
RCAP_rcc02567	0.71	0	155	256	0.00%	0.00%	138	186	235	277	*resolvase*	Replication, Recombination and Repair	Unknown
RCAP_rcc02568	-0.07	0.7572	129	123	0.00%	0.00%	103	156	108	138	*hypothetical protein*	Unknown	Unknown
RCAP_rcc02569	0.18	0.1565	144	165	0.00%	0.00%	133	159	146	183	*hypothetical protein*	Unknown	Unknown
RCAP_rcc02570	-1.09	0.0005	39	17	0.00%	0.00%	25	51	12	22	*hypothetical protein*	Unknown	Unknown
RCAP_rcc02571	-0.33	0.1868	379	297	0.01%	0.00%	269	477	245	348	*hypothetical protein*	Unknown	Unknown
RCAP_rcc02572	0.03	0.8541	190	194	0.00%	0.00%	170	214	174	214	*hypothetical protein*	Unknown	Unknown
RCAP_rcc02573	0.03	0.9059	13	13	0.00%	0.00%	11	14	11	15	*hypothetical protein*	Unknown	Unknown
RCAP_rcc02574	0.51	0.0099	15	22	0.00%	0.00%	13	19	20	25	*hypothetical protein*	Unknown	Unknown

RCAP_rcc02575	0.5	0.0474	7	10	0.00%	0.00%	5	9	8	13	*hypothetical protein*	Unknown	Unknown
RCAP_rcc02576	-0.05	0.8753	87	84	0.00%	0.00%	68	107	67	102	*BRO family protein*	Transcription	Unknown
RCAP_rcc02577	-0.29	0.3289	1963	1576	0.03%	0.02%	1337	2521	1187	1965	*phage integrase*	Replication, Recombination and Repair	Phage Interaction
RCAP_rcc02578	-3.81	0	3563	201	0.05%	0.00%	2781	4011	124	279	*iron(III) ABC transporter periplasmic iron(III)-compound-binding prote*	Metal, Ion, Cofactor Transport	Iron and Heme Transport
RCAP_rcc02579	-2.85	0	921	104	0.01%	0.00%	633	1120	56	152	*Fe(III) ABC transporter permease*	Metal, Ion, Cofactor Transport	Iron and Heme Transport
RCAP_rcc02580	-0.56	0.0243	129	85	0.00%	0.00%	95	157	62	108	*metallo-beta-lactamase*	Unknown	Unknown
RCAP_rcc02581	0.44	0.0146	485	665	0.01%	0.01%	409	549	533	797	*sensor histidine kinase*	Signal Transduction	Kinase/Phosphorelay
RCAP_rcc02582	-0.01	0.9527	117	116	0.00%	0.00%	90	142	104	129	*hypothetical protein*	Unknown	Unknown
RCAP_rcc02583	0.38	0.0139	19610	25632	0.29%	0.35%	17513	23129	22184	29080	*lon*	Post-translational Modification, Assembly and Chaperones	Unknown
RCAP_rcc02584	0.4	0.0821	204	272	0.00%	0.00%	159	243	200	345	*hup3*	Energy Metabolism	Aerobic/Anaerobic Respiration
RCAP_rcc02585	0.43	0	2964	4003	0.04%	0.05%	2859	3197	3835	4171	*ndh*	Energy Metabolism	Oxidative phosphorylation
RCAP_rcc02586	0.4	0.2766	301	411	0.00%	0.01%	178	412	268	554	*hypothetical protein*	Unknown	Unknown
RCAP_rcc02587	-0.32	0.1899	1757	1393	0.02%	0.02%	1287	2191	1156	1630	*hypothetical protein*	Unknown	Unknown
RCAP_rcc02588	-0.05	0.8048	140	134	0.00%	0.00%	116	158	115	153	*hypothetical protein*	Unknown	Unknown
RCAP_rcc02589	1.43	0.0009	138	492	0.00%	0.01%	51	226	189	794	*fadJ*	Lipid Metabolism	Fatty acid metabolism
RCAP_rcc02590	1.68	0	939	3087	0.01%	0.04%	901	1107	2593	3581	*dksA2*	Signal Transduction	Kinase/Phosphorelay
RCAP_rcc02591	-0.14	0.2962	1053	955	0.01%	0.01%	925	1161	861	1048	*surface presentation of antigens protein family*	Motility	Flagellar Assembly
RCAP_rcc02592	-0.61	0	1169	762	0.02%	0.01%	1044	1245	706	819	*Na+/solute symporter / histidine kinase*	Signal Transduction	Kinase/Phosphorelay
RCAP_rcc02593	0.2	0.5851	29	34	0.00%	0.00%	19	38	25	42	*hypothetical protein*	Unknown	Unknown
RCAP_rcc02594	0.15	0.68	129	143	0.00%	0.00%	83	166	93	194	*response regulator receiver protein*	Signal Transduction	Transcription Regulator
RCAP_rcc02595	0.21	0.337	488	568	0.01%	0.01%	384	601	488	647	*XRE family transcriptional regulator*	Signal Transduction	Transcription Regulator
RCAP_rcc02596	0.46	0.1174	832	1180	0.01%	0.02%	649	1117	992	1368	*lipoprotein*	Predicted Function	Unknown
RCAP_rcc02597	-0.2	0.2047	1378	1198	0.02%	0.02%	1130	1601	1073	1323	*hypothetical protein*	Unknown	Unknown
RCAP_rcc02598	0.01	0.9745	9926	9984	0.14%	0.14%	6889	13109	8851	11117	*beta-alanine--pyruvate transaminase*	Carbohydrate Metabolism	Propanoate metabolism
RCAP_rcc02599	0.89	0.0002	200	386	0.00%	0.01%	139	275	333	438	*TetR family transcriptional regulator*	Signal Transduction	Transcription Regulator
RCAP_rcc02600	-1.56	0	883	276	0.01%	0.00%	500	1190	228	324	*hypothetical protein*	Unknown	Unknown
RCAP_rcc02601	0.79	0	1435	2529	0.02%	0.03%	1240	1818	2218	2841	*accB*	Lipid Metabolism	Unknown
RCAP_rcc02602	0.52	0	4166	5982	0.06%	0.08%	3969	4691	5472	6492	*accC*	Lipid Metabolism	Fatty acid biosynthesis
RCAP_rcc02603	0.2	0.1523	362	415	0.01%	0.01%	315	412	386	444	*hypothetical protein*	Unknown	Unknown
RCAP_rcc02604	-0.81	0	537	304	0.01%	0.00%	457	591	276	331	*aat*	Unknown	Unknown
RCAP_rcc02605	-0.69	0	206	127	0.00%	0.00%	178	223	114	139	*hypothetical protein*	Unknown	Unknown
RCAP_rcc02606	-0.58	0	1981	1317	0.03%	0.02%	1825	2089	1180	1453	*mammalian cell entry domain-containing protein*	Secondary metabolites biosynthesis, transport, and catabolism	Unknown
RCAP_rcc02607	-0.78	0	5091	2938	0.07%	0.04%	4334	5686	2543	3333	*NDUFA12 family NADH ubiquinone oxidoreductase subunit*	Energy Metabolism	Unknown
RCAP_rcc02608	-0.11	0.4722	20541	19006	0.29%	0.26%	18254	23035	16717	21296	*clpX*	Post-translational Modification, Assembly and Chaperones	Unknown
RCAP_rcc02609	0.81	0	4628	8191	0.07%	0.11%	4267	5484	7123	9259	*clpP*	Post-translational Modification, Assembly and Chaperones	Unknown
RCAP_rcc02610	-0.64	0	804	512	0.01%	0.01%	680	904	469	555	*hypothetical protein*	Unknown	Unknown
RCAP_rcc02611	-0.83	0	5099	2840	0.07%	0.04%	4287	5718	2644	3036	*mcpA3*	Motility	Chemotaxis
RCAP_rcc02612	0.07	0.623	183	193	0.00%	0.00%	163	198	169	217	*transthyretin family protein*	Unknown	Unknown
RCAP_rcc02613	0.24	0.0466	1686	1990	0.02%	0.03%	1510	1878	1816	2164	*chitin deacetylase*	Carbohydrate Metabolism	Unknown
RCAP_rcc02614	-0.03	0.8048	1846	1803	0.03%	0.02%	1671	2055	1636	1971	*cupin domain-containing protein*	Unknown	Unknown
RCAP_rcc02615	0.47	0.0863	177	251	0.00%	0.00%	137	214	164	338	*ttuD*	Carbohydrate Metabolism	Glyoxylate and dicarboxylate metabolism
RCAP_rcc02616	0.82	0	428	763	0.01%	0.01%	380	488	666	861	*hypothetical protein*	Unknown	Unknown
RCAP_rcc02617	0.18	0.3078	1365	1549	0.02%	0.02%	1222	1553	1322	1776	*ushA*	Metabolism of Cofactors, Coenzymes and Vitamins	Nicotinate and nicotinamide metabolism
RCAP_rcc02618	0.28	0.0682	336	409	0.00%	0.01%	292	376	357	461	*hypothetical protein*	Unknown	Unknown
RCAP_rcc02619	-0.01	0.9172	1099	1087	0.02%	0.01%	996	1162	992	1183	*pyrD2*	Nucleotide Metabolism	Unknown
RCAP_rcc02620	0.05	0.82	8874	9216	0.13%	0.13%	7853	10071	7507	10925	*acsA2*	Carbohydrate Metabolism	Glycolysis / Gluconeogenesis
RCAP_rcc02621	-0.52	0.0006	700	483	0.01%	0.01%	584	784	428	537	*sulP*	Metal and Ion Transport	Unknown
RCAP_rcc02622	-0.14	0.7595	64	57	0.00%	0.00%	35	91	40	74	*pyridoxamine 5'-phosphate oxidase*	Unknown	Unknown
RCAP_rcc02623	-1.39	0	264	97	0.00%	0.00%	201	313	87	108	*hypothetical protein*	Unknown	Unknown
RCAP_rcc02624	-0.11	0.6574	3913	3612	0.06%	0.05%	3105	4758	3009	4214	*lysS*	Translation, ribosomal structure and biogenesis	Aminoacyl-tRNA biosynthesis
RCAP_rcc02625	1.56	0	260	779	0.00%	0.01%	281	328	701	858	*hypothetical protein*	Unknown	Unknown
RCAP_rcc02626	-0.52	0.0016	586	403	0.01%	0.01%	468	696	357	450	*hypothetical protein*	Unknown	Unknown
RCAP_rcc02627	0.3	0.1635	288	358	0.00%	0.00%	231	340	286	429	*dacB*	Glycan Biosynthesis and Metabolism	Peptidoglycan biosynthesis
RCAP_rcc02628	0.36	0.198	122	159	0.00%	0.00%	87	153	123	195	*nadD*	Metabolism of Cofactors, Coenzymes and Vitamins	Nicotinate and nicotinamide metabolism
RCAP_rcc02629	-0.35	0.136	226	176	0.00%	0.00%	164	277	141	211	*diguanylate cyclase/phosphodiesterase*	Signal Transduction	Kinase/Phosphorelay
RCAP_rcc02630	-0.12	0.4114	355	327	0.00%	0.00%	304	405	305	349	*heme NO binding domain-containing protein*	Unknown	Unknown
RCAP_rcc02631	0.97	0	199	399	0.00%	0.01%	165	237	310	488	*hypothetical protein*	Unknown	Unknown
RCAP_rcc02632	1.3	0	33	84	0.00%	0.00%	27	41	62	107	*HAD superfamily hydrolase*	Carbohydrate Metabolism	Glyoxylate and dicarboxylate metabolism
RCAP_rcc02633	1.62	0	21	67	0.00%	0.00%	19	26	49	84	*hypothetical protein*	Unknown	Unknown
RCAP_rcc02634	-0.36	0.0003	4873	3799	0.07%	0.05%	4316	5290	3543	4055	*response regulator receiver modulated diguanylate cyclase/phosphodies*	Signal Transduction	Transcription Regulator
RCAP_rcc02635	0.9	0	307	592	0.00%	0.01%	269	387	453	730	*hypothetical protein*	Unknown	Unknown
RCAP_rcc02636	1.55	0	54	166	0.00%	0.00%	53	71	120	212	*hypothetical protein*	Unknown	Unknown
RCAP_rcc02637	1.84	0	127	540	0.00%	0.01%	105	185	305	774	*ECF family RNA polymerase sigma factor*	Transcription	Unknown
RCAP_rcc02638	1.73	0	313	1353	0.00%	0.02%	218	477	764	1941	*calcium-binding EF-hand domain-containing protein*	Unknown	Unknown
RCAP_rcc02639	-0.21	0.552	94	80	0.00%	0.00%	64	119	60	99	*NUDIX superfamily hydrolase*	Unknown	Unknown
RCAP_rcc02640	-0.24	0.6723	61	48	0.00%	0.00%	19	97	22	74	*lipocalin family protein*	Predicted Function	Unknown
RCAP_rcc02641	-0.06	0.8956	31	29	0.00%	0.00%	18	42	18	41	*lipoprotein*	Predicted Function	Unknown
RCAP_rcc02642	-0.34	0.0003	1163	918	0.02%	0.01%	1041	1261	872	964	*aldo/keto reductase family oxidoreductase*	Energy Metabolism	Unknown
RCAP_rcc02643	-0.56	0	112	76	0.00%	0.00%	96	123	70	82	*class IV aminotransferase*	Metabolism of Cofactors, Coenzymes and Vitamins	Folate biosynthesis
RCAP_rcc02644	-1.04	0	287	138	0.00%	0.00%	254	304	128	148	*pabB*	Metabolism of Cofactors, Coenzymes and Vitamins	Folate biosynthesis
RCAP_rcc02645	-1.1	0	2185	1010	0.03%	0.01%	1904	2334	893	1127	*frc*	Energy Metabolism	Unknown
RCAP_rcc02646	-1.05	0	1868	888	0.03%	0.01%	1518	2104	786	990	*hypothetical protein*	Unknown	Unknown
RCAP_rcc02647	0.28	0.2584	1928	2370	0.03%	0.03%	1407	2552	1971	2770	*putA*	Amino Acid Metabolism	Arginine and proline metabolism
RCAP_rcc02648	0.09	0.6931	136	146	0.00%	0.00%	111	164	123	168	*putR*	Transcription	Unknown
RCAP_rcc02649	0.39	0.0341	312	411	0.00%	0.01%	256	374	364	458	*selD*	Metabolism of Other Amino Acids	Selenoamino acid metabolism
RCAP_rcc02650	0.12	0.5767	434	472	0.01%	0.01%	341	523	425	518	*UspA domain-containing protein*	Stress Response	Unknown
RCAP_rcc02651	-1.44	0.0001	415	127	0.01%	0.00%	125	670	100	154	*hypothetical protein*	Unknown	Unknown

RCAP_rcc02652	0.2	0.0818	1609	1854	0.02%	0.03%	1487	1778	1679	2030	*RbsD/FucU transport protein family*	Carbohydrate Metabolism	Unknown
RCAP_rcc02653	0.1	0.7484	174	188	0.00%	0.00%	126	219	140	237	*glpK1*	Lipid Metabolism	Glycerolipid metabolism
RCAP_rcc02654	-0.46	0.0001	475	345	0.01%	0.00%	432	509	303	386	*tktB*	Carbohydrate Metabolism	Pentose phosphate pathway
RCAP_rcc02655	-0.5	0.0027	662	463	0.01%	0.01%	564	749	394	533	*tktA*	Carbohydrate Metabolism	Pentose phosphate pathway
RCAP_rcc02656	0.03	0.9037	608	621	0.01%	0.01%	490	730	537	704	*hypothetical protein*	Unknown	Unknown
RCAP_rcc02657	-0.7	0.0001	26120	15798	0.36%	0.21%	23003	28925	12626	18970	*monosacharide ABC transporter periplasmic monosacharide-binding pr*	Carbohydrate Metabolism	Aerobic/Anaerobic Respiration
RCAP_rcc02658	-0.48	0.0002	554	395	0.01%	0.01%	494	600	342	447	*lipoprotein*	Predicted Function	Unknown
RCAP_rcc02659	-0.71	0	1891	1150	0.03%	0.02%	1682	2041	989	1310	*monosacharide ABC transporter ATP-binding protein*	Carbohydrate Metabolism	Aerobic/Anaerobic Respiration
RCAP_rcc02660	-0.81	0	1768	999	0.02%	0.01%	1593	1889	855	1143	*monosacharide ABC transporter permease*	Carbohydrate Metabolism	Aerobic/Anaerobic Respiration
RCAP_rcc02661	-0.65	0.0001	957	602	0.01%	0.01%	814	1068	514	691	*DeoR family transcriptional regulator*	Signal Transduction	Transcription Regulator
RCAP_rcc02662	1.37	0	451	1191	0.01%	0.02%	404	529	1067	1316	*class II aldolase/adducin N-terminal domain-containing protein/short-c*	Unknown	Unknown
RCAP_rcc02663	2.01	0	188	778	0.00%	0.01%	188	226	667	890	*DeoR family transcriptional regulator*	Signal Transduction	Transcription Regulator
RCAP_rcc02664	1.84	0	246	901	0.00%	0.01%	236	306	824	978	*mtnA*	Amino Acid Metabolism	Cysteine and methionine metabolism
RCAP_rcc02665	2.15	0	222	1001	0.00%	0.01%	237	281	909	1093	*hemolysin-type calcium-binding repeat family protein*	Trafficking and Secretion	Secretion
RCAP_rcc02666	0.71	0	368	608	0.01%	0.01%	348	413	571	645	*alpha/beta fold family hydrolase*	Unknown	Unknown
RCAP_rcc02667	0.1	0.4168	2975	3190	0.04%	0.04%	2728	3243	2899	3482	*fabI2*	Lipid Metabolism	Fatty acid biosynthesis
RCAP_rcc02668	0.25	0.0746	4317	5136	0.06%	0.07%	3844	4934	4634	5639	*fabB*	Lipid Metabolism	Biotin metabolism
RCAP_rcc02669	0.75	0	1385	2351	0.02%	0.03%	1260	1633	2062	2640	*fabA*	Lipid Metabolism	Fatty acid biosynthesis
RCAP_rcc02670	0.18	0.3282	3004	3412	0.04%	0.05%	2700	3448	2855	3969	*Fur family transcriptional regulator*	Signal Transduction	Transcription Regulator
RCAP_rcc02671	-0.39	0.1672	2868	2145	0.04%	0.03%	1991	3696	1688	2601	*efp*	Translation, ribosomal structure and biogenesis	Unknown
RCAP_rcc02672	-0.01	0.9403	302	300	0.00%	0.00%	279	325	275	325	*lysU*	Translation, ribosomal structure and biogenesis	Aminoacyl-tRNA biosynthesis
RCAP_rcc02673	-0.23	0.1194	477	407	0.01%	0.01%	397	549	372	442	*hypothetical protein*	Unknown	Unknown
RCAP_rcc02674	-0.32	0.0046	625	500	0.01%	0.01%	553	691	457	544	*nylB*	Defense Mechanisms	Unknown
RCAP_rcc02675	-0.47	0.0553	3398	2412	0.05%	0.03%	2436	4282	2009	2816	*MarR family transcriptional regulator*	Signal Transduction	Transcription Regulator
RCAP_rcc02676	-0.19	0.4336	640	556	0.01%	0.01%	473	795	458	653	*hypothetical protein*	Unknown	Unknown
RCAP_rcc02677	-0.03	0.8151	1226	1202	0.02%	0.02%	1105	1360	1123	1281	*aspC2*	Photosynthesis	Tropane, piperidine and pyridine alkaloid biosynthesis
RCAP_rcc02678	-0.2	0.1104	846	733	0.01%	0.01%	729	939	672	794	*hypothetical protein*	Unknown	Unknown
RCAP_rcc02679	0.75	0	718	1214	0.01%	0.02%	668	844	1118	1311	*pyridine nucleotide-disulfide oxidoreductase*	Energy Metabolism	Unknown
RCAP_rcc02680	0.1	0.5558	3666	3947	0.05%	0.05%	3191	4136	3459	4436	*mdtK*	Defense Mechanisms	Unknown
RCAP_rcc02681	0.08	0.5443	1553	1639	0.02%	0.02%	1397	1744	1513	1766	*cytochrome b561 family protein*	Energy Metabolism	Aerobic/Anaerobic Respiration
RCAP_rcc02682	0.61	0	3243	4976	0.05%	0.07%	3018	3793	4500	5452	*cytochrome c'*	Energy Metabolism	Aerobic/Anaerobic Respiration
RCAP_rcc02683	1.27	0	216	539	0.00%	0.01%	194	271	419	658	*type 11 family methyltransferase*	Unknown	Unknown
RCAP_rcc02684	1.42	0	1181	3247	0.02%	0.04%	1090	1479	2834	3660	*polyphosphate kinase 2 domain-containing protein*	Energy Metabolism	Oxidative phosphorylation
RCAP_rcc02685	0.16	0.3167	786	878	0.01%	0.01%	670	916	797	959	*glutathione S-transferase*	Sulfur Metabolism	Glutathione metabolism
RCAP_rcc02686	-0.21	0.0441	960	831	0.01%	0.01%	873	1025	759	903	*prfA*	Translation, ribosomal structure and biogenesis	Unknown
RCAP_rcc02687	-0.16	0.3153	361	322	0.00%	0.00%	308	402	284	360	*hemK*	Metabolism of Cofactors, Coenzymes and Vitamins	Heme Biosynthesis
RCAP_rcc02688	0.36	0.0106	990	1281	0.01%	0.02%	901	1120	1105	1456	*hypothetical protein*	Unknown	Unknown
RCAP_rcc02689	-0.71	0	447	273	0.01%	0.00%	405	469	245	300	*ksgA*	Translation, ribosomal structure and biogenesis	Unknown
RCAP_rcc02690	-0.06	0.8773	453	434	0.01%	0.01%	309	568	321	548	*pdxA*	Metabolism of Cofactors, Coenzymes and Vitamins	Vitamin B6 metabolism
RCAP_rcc02691	0.55	0.0721	803	1215	0.01%	0.02%	537	1081	904	1526	*surA*	Post-translational Modification, Assembly and Chaperones	Unknown
RCAP_rcc02692	-0.01	0.9371	1226	1216	0.02%	0.02%	1114	1302	1109	1324	*organic solvent tolerance protein family*	Cell Envelope Biosynthesis	Cell Wall Biosynthesis
RCAP_rcc02693	0.23	0.5203	331	394	0.00%	0.01%	211	441	292	496	*permease YjgP/YjgQ family protein*	Predicted Function	Unknown
RCAP_rcc02694	-0.19	0.1936	701	614	0.01%	0.01%	586	797	583	645	*permease YjgP/YjgQ family protein*	Predicted Function	Unknown
RCAP_rcc02695	-0.14	0.5257	1846	1669	0.03%	0.02%	1381	2259	1456	1882	*pepA2*	Metabolism of Other Amino Acids	Glutathione metabolism
RCAP_rcc02696	-0.51	0.0033	285	198	0.00%	0.00%	228	332	177	220	*holC*	Replication, Recombination and Repair	Replication
RCAP_rcc02697	-0.59	0.0007	435	285	0.01%	0.00%	345	506	243	327	*thiB*	Metabolism of Cofactors, Coenzymes and Vitamins	Thiamine metabolism
RCAP_rcc02698	0.26	0.3498	55	66	0.00%	0.00%	44	67	43	89	*thiP*	Metabolism of Cofactors, Coenzymes and Vitamins	Thiamine metabolism
RCAP_rcc02699	1.09	0.0002	28	64	0.00%	0.00%	23	37	38	90	*thiQ*	Metabolism of Cofactors, Coenzymes and Vitamins	Thiamine metabolism
RCAP_rcc02700	0.3	0.1651	571	708	0.01%	0.01%	433	746	634	782	*lipoprotein*	Predicted Function	Unknown
RCAP_rcc02701	0.13	0.6197	353	389	0.00%	0.01%	247	463	319	459	*lipoprotein*	Predicted Function	Unknown
RCAP_rcc02702	0.2	0.2419	1466	1687	0.02%	0.02%	1346	1702	1396	1978	*cytochrome c/b561 family protein*	Energy Metabolism	Aerobic/Anaerobic Respiration
RCAP_rcc02703	-0.08	0.7472	231	218	0.00%	0.00%	169	289	198	239	*hypothetical protein*	Unknown	Unknown
RCAP_rcc02704	0.3	0.3929	244	308	0.00%	0.00%	168	311	216	399	*NLP/P60 family protein*	Cell Envelope Biosynthesis	Cell Wall Biosynthesis
RCAP_rcc02705	0.4	0.0477	850	1132	0.01%	0.02%	673	1011	914	1349	*pepA3*	Metabolism of Other Amino Acids	Glutathione metabolism
RCAP_rcc02706	-0.66	0.0146	157	95	0.00%	0.00%	98	202	71	120	*hypothetical protein*	Unknown	Unknown
RCAP_rcc02707	1.52	0	1320	3897	0.02%	0.05%	1375	1706	3269	4524	*cynT*	Energy Metabolism	Nitrogen metabolism
RCAP_rcc02708	0.64	0	1522	2385	0.02%	0.03%	1484	1740	2161	2610	*hypothetical protein*	Unknown	Unknown
RCAP_rcc02709	0.01	0.947	129	131	0.00%	0.00%	116	143	114	149	*hypothetical protein*	Unknown	Unknown
RCAP_rcc02710	0.19	0.4816	908	1043	0.01%	0.01%	674	1139	841	1245	*hypothetical protein*	Unknown	Unknown
RCAP_rcc02711	-0.62	0.0111	9190	5811	0.13%	0.08%	6772	11341	4592	7030	*asd*	Amino Acid Metabolism	Lysine biosynthesis
RCAP_rcc02712	-0.58	0	907	605	0.01%	0.01%	801	973	563	647	*major facilitator superfamily protein*	Carbohydrate Metabolism	Unknown
RCAP_rcc02713	0.37	0.0429	77	100	0.00%	0.00%	64	90	85	116	*LysE family transporter*	Amino Acid Metabolism	Unknown
RCAP_rcc02714	-0.8	0.0001	2383	1335	0.03%	0.02%	1907	2758	1099	1571	*hypothetical protein*	Unknown	Unknown
RCAP_rcc02715	-0.65	0.0023	7186	4477	0.10%	0.06%	5639	8544	3645	5310	*hypothetical protein*	Unknown	Unknown
RCAP_rcc02716	-0.85	0.0001	5836	3152	0.08%	0.04%	4494	6938	2562	3742	*hypothetical protein*	Unknown	Unknown
RCAP_rcc02717	-0.74	0.0007	9845	5759	0.14%	0.08%	7510	11785	4689	6829	*cytosine-specific DNA-methyltransferase*	Replication, Recombination and Repair	Unknown
RCAP_rcc02718	-0.45	0.0564	40	29	0.00%	0.00%	31	48	25	34	*hypothetical protein*	Unknown	Unknown
RCAP_rcc02719	-0.53	0.0018	35	24	0.00%	0.00%	30	39	21	28	*resolvase*	Replication, Recombination and Repair	Unknown
RCAP_rcc02720	-0.18	0.346	23	21	0.00%	0.00%	19	27	18	23	*hypothetical protein*	Unknown	Unknown
RCAP_rcc02721	-0.35	0.2363	7	5	0.00%	0.00%	5	9	4	7	*hypothetical protein*	Unknown	Unknown
RCAP_rcc02722	0.35	0.0892	428	552	0.01%	0.01%	344	530	480	624	*hypothetical protein*	Unknown	Unknown
RCAP_rcc02723	-0.39	0.0746	165	124	0.00%	0.00%	127	196	106	142	*hypothetical protein*	Unknown	Unknown
RCAP_rcc02724	-1.08	0.0008	65	28	0.00%	0.00%	35	93	22	35	*ECF family RNA polymerase sigma factor*	Unknown	Unknown
RCAP_rcc02725	-1.38	0	27	10	0.00%	0.00%	18	35	8	12	*hypothetical protein*	Unknown	Unknown
RCAP_rcc02726	-0.91	0.0213	16	8	0.00%	0.00%	9	24	5	10	*hypothetical protein*	Unknown	Unknown
RCAP_rcc02727	-1.13	0	41	18	0.00%	0.00%	29	51	16	20	*hypothetical protein*	Unknown	Unknown
RCAP_rcc02728	-1.28	0	59	23	0.00%	0.00%	39	76	20	26	*ATPase AAA*	Unknown	Unknown

RCAP_rcc02729	-1.09	0	46	21	0.00%	0.00%	31	57	18	23	*hypothetical protein*	Unknown	Unknown
RCAP_rcc02730	-0.73	0.0048	19	11	0.00%	0.00%	14	24	9	13	*hypothetical protein*	Unknown	Unknown
RCAP_rcc02732	-0.7	0.0445	6	4	0.00%	0.00%	4	8	2	5	*hypothetical protein*	Unknown	Unknown
RCAP_rcc02733	-0.56	0.0332	16	11	0.00%	0.00%	12	20	8	13	*hypothetical protein*	Unknown	Unknown
RCAP_rcc02734	-0.57	0.0051	35	23	0.00%	0.00%	26	44	21	26	*recD*	Replication, Recombination and Repair	Unknown
RCAP_rcc02735	-0.25	0.0217	69	57	0.00%	0.00%	62	76	54	61	*virulence-associated protein E*	Unknown	Unknown
RCAP_rcc02736	0.04	0.8848	116	119	0.00%	0.00%	90	140	103	135	*hypothetical protein*	Unknown	Unknown
RCAP_rcc02737	0.07	0.8409	5	5	0.00%	0.00%	4	7	4	7	*hypothetical protein*	Unknown	Unknown
RCAP_rcc02738	0.2	0.5491	7	8	0.00%	0.00%	5	9	6	9	*hypothetical protein*	Unknown	Unknown
RCAP_rcc02739	-0.42	0.0665	1172	863	0.02%	0.01%	878	1436	712	1014	*hypothetical protein*	Unknown	Unknown
RCAP_rcc02740	0.33	0.3177	12	15	0.00%	0.00%	9	16	12	19	*ParB domain-containing protein nuclease*	Replication, Recombination and Repair	Unknown
RCAP_rcc02741	0.84	0.0023	6	11	0.00%	0.00%	5	8	9	14	*adenine-specific DNA-methyltransferase*	Unknown	Unknown
RCAP_rcc02742	-0.08	0.78	30	28	0.00%	0.00%	23	36	21	35	*cytosine-specific DNA-methyltransferase*	Replication, Recombination and Repair	Unknown
RCAP_rcc02743	-0.49	0.0716	14	10	0.00%	0.00%	10	17	7	12	*hypothetical protein*	Unknown	Unknown
RCAP_rcc02744	0.26	0.3358	95	115	0.00%	0.00%	73	133	94	136	*cysP*	Metal and Ion Transport	Sulfute
RCAP_rcc02745	0.23	0.6232	107	131	0.00%	0.00%	51	157	72	190	*RND family efflux transporter subunit MFP*	Cell Envelope Biosynthesis	Cell Wall Biosynthesis
RCAP_rcc02746	-0.03	0.947	63	61	0.00%	0.00%	34	89	35	87	*RND family efflux transporter subunit MFP*	Cell Envelope Biosynthesis	Cell Wall Biosynthesis
RCAP_rcc02747	-0.36	0.0015	1543	1200	0.02%	0.02%	1338	1702	1109	1291	*acriflavin resistance protein family*	Metal and Ion Transport	Unknown
RCAP_rcc02748	1.06	NA	31	70	0.00%	0.00%	26	39	30	111	*hypothetical protein*	Unknown	Unknown
RCAP_rcc02749	-0.07	0.764	3885	3690	0.05%	0.05%	3043	4781	3142	4238	*rpmG*	Translation, ribosomal structure and biogenesis	Unknown
RCAP_rcc02750	1.55	0	1228	3810	0.02%	0.05%	1120	1760	3122	4497	*hypothetical protein*	Unknown	Unknown
RCAP_rcc02751	0.04	0.903	412	423	0.01%	0.01%	308	512	359	487	*N-acetylmuramoyl-L-alanine amidase, family 2*	Cell Envelope Biosynthesis	Cell Wall Biosynthesis
RCAP_rcc02752	-0.13	0.3017	810	737	0.01%	0.01%	718	892	680	795	*lipoprotein*	Predicted Function	Unknown
RCAP_rcc02753	-0.2	0.2895	4258	3703	0.06%	0.05%	3572	4927	3238	4168	*gatA*	Translation, ribosomal structure and biogenesis	Aminoacyl-tRNA biosynthesis
RCAP_rcc02754	0.34	0.0521	543	694	0.01%	0.01%	466	642	600	788	*gatC*	Translation, ribosomal structure and biogenesis	Aminoacyl-tRNA biosynthesis
RCAP_rcc02755	0.54	0.0039	345	509	0.01%	0.01%	288	426	435	583	*metallo-beta-lactamase*	Unknown	Unknown
RCAP_rcc02756	0.91	0	297	563	0.00%	0.01%	284	355	523	603	*hypothetical protein*	Unknown	Unknown
RCAP_rcc02757	-0.04	0.5014	1838	1785	0.03%	0.02%	1738	1913	1712	1859	*dnaX*	Replication, Recombination and Repair	Replication
RCAP_rcc02758	1.77	0	1087	3785	0.02%	0.05%	1284	1382	3288	4282	*hypothetical protein*	Unknown	Unknown
RCAP_rcc02759	1.62	0	207	689	0.00%	0.01%	183	284	510	869	*recR*	Replication, Recombination and Repair	Recombination
RCAP_rcc02760	0.77	0	140	240	0.00%	0.00%	131	163	214	267	*TfoX N-terminal domain-containing protein*	Transcription	Unknown
RCAP_rcc02761	0.39	0.0012	1019	1336	0.01%	0.02%	920	1163	1219	1454	*glutathione S-transferase*	Sulfur Metabolism	Glutathione metabolism
RCAP_rcc02762	0.33	0.0052	124	155	0.00%	0.00%	110	140	145	165	*4Fe-4S ferredoxin, iron-sulfur cluster binding protein*	Energy Metabolism	Aerobic/Anaerobic Respiration
RCAP_rcc02763	0.31	0.0563	762	950	0.01%	0.01%	656	878	828	1072	*hypothetical protein*	Unknown	Unknown
RCAP_rcc02764	1.05	0	3568	7609	0.05%	0.10%	3097	4660	6827	8392	*hypothetical protein*	Unknown	Unknown
RCAP_rcc02765	0.36	0.1174	4524	5895	0.06%	0.08%	3394	5684	5005	6784	*ilvE1*	Metabolism of Cofactors, Coenzymes and Vitamins	Pantothenate and CoA biosynthesis
RCAP_rcc02766	0.12	0.5581	276	301	0.00%	0.00%	220	324	256	346	*petP*	Signal Transduction	Transcription Regulator
RCAP_rcc02767	0.14	0.3323	237	261	0.00%	0.00%	203	265	240	281	*petR*	Signal Transduction	Transcription Regulator
RCAP_rcc02768	0.41	0	7000	9333	0.10%	0.13%	6819	7549	8491	10174	*petA*	Energy Metabolism	Aerobic/Anaerobic Respiration
RCAP_rcc02769	0.12	0.5727	34149	37286	0.49%	0.51%	28662	41329	31392	43180	*petB*	Energy Metabolism	Aerobic/Anaerobic Respiration
RCAP_rcc02770	0.19	0.0755	16634	19026	0.24%	0.26%	16036	18230	17116	20936	*petC*	Energy Metabolism	Aerobic/Anaerobic Respiration
RCAP_rcc02771	1.03	0	543	1112	0.01%	0.02%	524	617	1039	1184	*TetR family transcriptional regulator*	Signal Transduction	Transcription Regulator
RCAP_rcc02772	0.43	0.09	191	262	0.00%	0.00%	143	238	203	322	*hypothetical protein*	Unknown	Unknown
RCAP_rcc02773	0.15	0.1956	1749	1949	0.02%	0.03%	1549	1926	1786	2112	*hisP*	Amino Acid Metabolism	Amino Acid Transport
RCAP_rcc02774	0.59	0.0001	6613	10034	0.10%	0.14%	5950	7772	8672	11397	*hisJ*	Amino Acid Metabolism	Amino Acid Transport
RCAP_rcc02775	-0.12	0.4398	2470	2272	0.03%	0.03%	2129	2767	2020	2525	*hisQ*	Amino Acid Metabolism	Amino Acid Transport
RCAP_rcc02776	-0.27	0.1584	1899	1569	0.03%	0.02%	1561	2218	1340	1798	*hisM*	Amino Acid Metabolism	Amino Acid Transport
RCAP_rcc02777	0.11	0.486	5612	6085	0.08%	0.08%	4792	6482	5419	6751	*glnA4*	Carbohydrate Metabolism	Glyoxylate and dicarboxylate metabolism
RCAP_rcc02778	0.35	0.0028	1394	1785	0.02%	0.02%	1248	1544	1621	1950	*class I glutamine amidotransferase*	Xenobiotics Biodegradation and Metabolism	Drug metabolism - other enzymes
RCAP_rcc02779	0.19	0.4913	64	73	0.00%	0.00%	48	77	52	95	*hypothetical protein*	Unknown	Unknown
RCAP_rcc02780	-0.12	0.3987	2605	2393	0.04%	0.03%	2290	2863	2141	2646	*glnA5*	Carbohydrate Metabolism	Glyoxylate and dicarboxylate metabolism
RCAP_rcc02781	-0.25	0.1407	637	532	0.01%	0.01%	554	691	439	626	*FAD dependent oxidoreductase*	Energy Metabolism	Unknown
RCAP_rcc02782	-0.69	0	1213	748	0.02%	0.01%	1061	1345	685	811	*DegT/DnrJ/EryC1/StrS family aminotransferase*	Amino Acid Metabolism	Unknown
RCAP_rcc02783	1	0.0001	210	442	0.00%	0.01%	183	271	291	593	*CoA-binding domain-containing protein*	Unknown	Unknown
RCAP_rcc02784	0.56	0.0634	430	655	0.01%	0.01%	290	567	503	806	*TrmH family RNA methyltransferase*	Translation, ribosomal structure and biogenesis	Unknown
RCAP_rcc02785	0.36	0.0003	734	943	0.01%	0.01%	660	808	881	1004	*hypothetical protein*	Unknown	Unknown
RCAP_rcc02786	0.36	0.0352	231	299	0.00%	0.00%	190	276	272	325	*hemolysin-type calcium-binding repeat family protein*	Trafficking and Secretion	Secretion
RCAP_rcc02787	-0.1	0.4798	6247	5804	0.09%	0.08%	5559	6885	5130	6478	*metB*	Energy Metabolism	Sulfur metabolism
RCAP_rcc02788	-0.37	0.0023	761	586	0.01%	0.01%	650	844	531	640	*hypothetical protein*	Unknown	Unknown
RCAP_rcc02789	0.05	0.7483	883	912	0.01%	0.01%	790	967	799	1025	*kefC1*	Sulfur Metabolism	Glutathione metabolism
RCAP_rcc02790	-0.46	0.0142	11236	8100	0.16%	0.11%	9134	13326	6859	9342	*CarD family transcriptional regulator*	Signal Transduction	Transcription Regulator
RCAP_rcc02791	0.32	0.2114	5901	7446	0.08%	0.10%	4481	7597	6178	8714	*fdxA*	Energy Metabolism	Aerobic/Anaerobic Respiration
RCAP_rcc02792	-0.09	0.4787	96	89	0.00%	0.00%	87	105	80	99	*hslR*	Stress Response	Unknown
RCAP_rcc02793	-0.04	0.5364	7008	6828	0.10%	0.09%	6859	7243	6456	7199	*helicase domain-containing protein*	Unknown	Unknown
RCAP_rcc02794	0.38	0.0001	550	718	0.01%	0.01%	503	609	670	765	*hypothetical protein*	Unknown	Unknown
RCAP_rcc02795	-0.01	0.958	1650	1641	0.02%	0.02%	1471	1850	1472	1810	*sterol-binding domain-containing protein*	Unknown	Unknown
RCAP_rcc02796	0.18	0.6292	942	1083	0.01%	0.01%	617	1239	796	1371	*pldB*	Lipid Metabolism	Glycerophospholipid metabolism
RCAP_rcc02797	0.01	0.9694	1049	1053	0.01%	0.01%	935	1152	939	1167	*T1 family peptidase*	Post-translational Modification, Assembly and Chaperones	Peptidase
RCAP_rcc02798	0.44	0.0177	300	412	0.00%	0.01%	247	356	359	466	*transglutaminase domain-containing protein*	Amino Acid Metabolism	Unknown
RCAP_rcc02799	0.28	0.0065	672	816	0.01%	0.01%	616	741	754	878	*hypothetical protein*	Unknown	Unknown
RCAP_rcc02800	0.75	0	1434	2429	0.02%	0.03%	1293	1670	2078	2781	*hypothetical protein*	Unknown	Unknown
RCAP_rcc02801	1.12	0	4116	9067	0.06%	0.12%	4187	4921	8018	10115	*pepF*	Amino Acid Metabolism	Unknown
RCAP_rcc02802	0.58	0.0005	390	590	0.01%	0.01%	337	461	503	678	*membrane dipeptidase*	Cell Envelope Biosynthesis	Cell Wall Biosynthesis
RCAP_rcc02803	0.26	0.3608	433	526	0.01%	0.01%	311	549	396	656	*hypothetical protein*	Unknown	Unknown
RCAP_rcc02804	-0.09	0.6337	156	147	0.00%	0.00%	138	171	116	177	*hypothetical protein*	Unknown	Unknown
RCAP_rcc02805	-0.22	0.3446	310	265	0.00%	0.00%	230	373	219	311	*hypothetical protein*	Unknown	Unknown
RCAP_rcc02806	-0.21	0.2369	223	192	0.00%	0.00%	182	257	169	214	*hypothetical protein*	Unknown	Unknown

RCAP_rcc02807	0.05	0.7751	458	474	0.01%	0.01%	382	526	429	520	*lgt*	Glycan Biosynthesis and Metabolism	Glycosphingolipid biosynthesis - ganglio series
RCAP_rcc02808	1.56	0	1586	4836	0.03%	0.07%	1686	2082	3916	5755	*hypothetical protein*	Unknown	Unknown
RCAP_rcc02809	1.22	0	199	469	0.00%	0.01%	211	234	394	544	*hypothetical protein*	Unknown	Unknown
RCAP_rcc02810	0.87	0.0001	267	503	0.00%	0.01%	240	333	371	635	*rluD*	Nucleotide Metabolism	Pyrimidine metabolism
RCAP_rcc02811	1.38	0	3511	9375	0.06%	0.13%	3539	4603	8112	10638	*rpoH2*	Replication, Recombination and Repair	Replication
RCAP_rcc02812	0.5	0.001	1160	1650	0.02%	0.02%	1059	1332	1363	1936	*gfo*	Unknown	Unknown
RCAP_rcc02813	0.37	0.0001	799	1035	0.01%	0.01%	767	876	942	1129	*lrp*	Signal Transduction	Transcription Regulator
RCAP_rcc02814	1.74	0	1969	6749	0.03%	0.09%	2226	2615	5834	7663	*trxB*	Sulfur Metabolism	Selenoamino acid metabolism
RCAP_rcc02815	1.75	0	2036	7218	0.03%	0.10%	1891	2793	5904	8531	*lipoprotein*	Predicted Function	Unknown
RCAP_rcc02816	-0.33	0.1696	7146	5603	0.10%	0.08%	5557	8638	4486	6719	*sat*	Energy Metabolism	Sulfur metabolism
RCAP_rcc02817	0.68	0	673	1093	0.01%	0.01%	598	817	965	1221	*hypothetical protein*	Unknown	Unknown
RCAP_rcc02818	0.79	0	1804	3194	0.03%	0.04%	1520	2358	2825	3562	*ibpA*	Stress Response	Unknown
RCAP_rcc02819	0.33	0.0259	864	1089	0.01%	0.01%	779	1015	954	1225	*hypothetical protein*	Unknown	Unknown
RCAP_rcc02820	0.14	0.0935	896	987	0.01%	0.01%	837	956	923	1051	*purE*	Nucleotide Metabolism	Purine metabolism
RCAP_rcc02821	0.01	0.9272	1457	1469	0.02%	0.02%	1336	1582	1375	1564	*purK*	Nucleotide Metabolism	Purine metabolism
RCAP_rcc02822	-0.07	0.7077	329	312	0.00%	0.00%	293	347	254	370	*lipase*	Lipid Metabolism	Glycerolipid metabolism
RCAP_rcc02823	-0.13	0.4825	2739	2491	0.04%	0.03%	2303	3175	2180	2802	*prs*	Carbohydrate Metabolism	Pentose phosphate pathway
RCAP_rcc02824	0.24	0.1649	409	483	0.01%	0.01%	339	475	426	541	*DSBA family oxidoreductase*	Secondary metabolites biosynthesis, transport, and catabolism	Unknown
RCAP_rcc02825	0.06	0.5942	701	731	0.01%	0.01%	642	756	672	790	*alpha/beta fold family hydrolase*	Unknown	Unknown
RCAP_rcc02826	-0.64	0.0009	7064	4460	0.10%	0.06%	5696	8315	3736	5184	*fumC*	Carbohydrate Metabolism	TCA Cycle
RCAP_rcc02827	0.36	0.0578	1163	1509	0.02%	0.02%	942	1424	1373	1644	*hypothetical protein*	Unknown	Unknown
RCAP_rcc02828	0.19	0.2035	3322	3813	0.05%	0.05%	2867	3876	3417	4208	*hypothetical protein*	Unknown	Unknown
RCAP_rcc02829	1.31	0	566	1440	0.01%	0.02%	535	693	1184	1696	*hypothetical protein*	Unknown	Unknown
RCAP_rcc02830	-0.28	0.0009	1046	861	0.01%	0.01%	961	1114	802	920	*acyltransferase domain-containing protein*	Unknown	Unknown
RCAP_rcc02831	0.19	0.0409	463	531	0.01%	0.01%	429	510	500	562	*ptsO*	Carbohydrate Metabolism	Unknown
RCAP_rcc02832	0.16	0.0867	615	685	0.01%	0.01%	554	676	657	713	*PTS system, IIA component*	Carbohydrate Metabolism	Fructose and mannose metabolism
RCAP_rcc02833	1.28	0	1188	2939	0.02%	0.04%	1145	1547	2546	3333	*P-loop family ATPase*	Unknown	Unknown
RCAP_rcc02834	-0.7	0	87	52	0.00%	0.00%	76	92	42	62	*hprK*	Signal Transduction	Kinase/Phosphorelay
RCAP_rcc02835	-0.62	0	2811	1822	0.04%	0.02%	2495	3011	1664	1980	*chvG*	Signal Transduction	Kinase/Phosphorelay
RCAP_rcc02836	-0.3	0.0839	3152	2549	0.04%	0.03%	2654	3617	2182	2916	*chvI*	Signal Transduction	Transcription Regulator
RCAP_rcc02837	0.33	0.0543	6782	8594	0.10%	0.12%	5979	7977	7427	9762	*pckA*	Carbohydrate Metabolism	TCA Cycle
RCAP_rcc02838	-0.34	0.0001	2442	1926	0.03%	0.03%	2264	2525	1754	2099	*rnr*	Transcription	Unknown
RCAP_rcc02839	0.54	0.2562	34	56	0.00%	0.00%	12	55	22	89	*mobA*	Metabolism of Cofactors, Coenzymes and Vitamins	Unknown
RCAP_rcc02840	2.13	0	24	122	0.00%	0.00%	23	31	68	177	*moaC2*	Metabolism of Cofactors, Coenzymes and Vitamins	Unknown
RCAP_rcc02841	3.05	0	25	248	0.00%	0.00%	32	40	159	337	*moeB2*	Metabolism of Cofactors, Coenzymes and Vitamins	Unknown
RCAP_rcc02842	3.15	0	4	57	0.00%	0.00%	6	8	34	80	*moaD1*	Metabolism of Cofactors, Coenzymes and Vitamins	Unknown
RCAP_rcc02843	3.32	0	33	385	0.00%	0.01%	47	56	264	506	*moaA2*	Unknown	Unknown
RCAP_rcc02844	3.58	NA	4	89	0.00%	0.00%	4	7	38	139	*hypothetical protein*	Unknown	Unknown
RCAP_rcc02845	4.16	NA	212	4681	0.01%	0.06%	433	487	3076	6287	*torA*	Energy Metabolism	Methane metabolism
RCAP_rcc02846	4.23	NA	2	138	0.00%	0.00%	4	6	37	239	*torD*	Unknown	Unknown
RCAP_rcc02847	4.98	0	35	1487	0.00%	0.02%	101	112	854	2121	*torC*	Energy Metabolism	Aerobic/Anaerobic Respiration
RCAP_rcc02848	1.22	0	233	546	0.00%	0.01%	230	274	499	593	*torR*	Signal Transduction	Transcription Regulator
RCAP_rcc02849	0.66	0.013	1516	2470	0.02%	0.03%	1128	1936	2090	2850	*dorS*	Energy Metabolism	Aerobic/Anaerobic Respiration
RCAP_rcc02850	0.46	0.0081	238	331	0.00%	0.00%	193	281	296	366	*thiM*	Metabolism of Cofactors, Coenzymes and Vitamins	Thiamine metabolism
RCAP_rcc02851	0.58	0.1498	5	9	0.00%	0.00%	4	7	4	13	*thiE*	Metabolism of Cofactors, Coenzymes and Vitamins	Thiamine metabolism
RCAP_rcc02852	0.54	0.1761	7	10	0.00%	0.00%	4	10	7	14	*thiD*	Metabolism of Cofactors, Coenzymes and Vitamins	Thiamine metabolism
RCAP_rcc02853	0.73	0.0009	14	24	0.00%	0.00%	12	17	19	29	*ABC transporter periplasmic substrate-binding protein*	Metal and Ion Transport	Unknown
RCAP_rcc02854	0.89	0.0003	8	16	0.00%	0.00%	6	10	13	19	*ABC transporter ATP-binding protein*	Metal and Ion Transport	Unknown
RCAP_rcc02855	0.3	0.3243	19	23	0.00%	0.00%	13	24	17	30	*ABC transporter permease*	Metal and Ion Transport	Unknown
RCAP_rcc02856	0.02	0.9269	302	307	0.00%	0.00%	251	352	285	329	*PAS/PAC sensor domain-containing protein*	Motility	Aerotaxis
RCAP_rcc02857	-0.11	0.4913	1270	1178	0.02%	0.02%	1081	1432	1069	1288	*diguanylate cyclase/phosphodiesterase*	Signal Transduction	Kinase/Phosphorelay
RCAP_rcc02858	0.75	0.0092	145	255	0.00%	0.00%	102	185	171	339	*hypothetical protein*	Unknown	Unknown
RCAP_rcc02859	-0.04	0.8889	6524	6312	0.09%	0.09%	4784	8246	4880	7744	*acsA3*	Carbohydrate Metabolism	Glycolysis / Gluconeogenesis
RCAP_rcc02860	-0.06	0.8443	1671	1601	0.02%	0.02%	1368	2003	1218	1983	*hypothetical protein*	Unknown	Unknown
RCAP_rcc02861	0.15	0.525	5276	5870	0.08%	0.08%	4684	6111	4622	7118	*actP3*	Unknown	Unknown
RCAP_rcc02862	0.45	0.0114	364	503	0.01%	0.01%	299	427	421	585	*cyclic nucleotide-binding domain-/cystathionine beta-synthase domain-*	Signal Transduction	Kinase/Phosphorelay
RCAP_rcc02863	0.46	0.1557	51	72	0.00%	0.00%	35	67	54	91	*response regulator receiver protein*	Signal Transduction	Transcription Regulator
RCAP_rcc02864	0.8	0.0044	160	292	0.00%	0.00%	115	208	217	366	*PAS domain/exonuclease domain-containing protein*	Replication, Recombination and Repair	Replication
RCAP_rcc02865	3.62	0	80	1059	0.00%	0.01%	99	124	909	1209	*pflA2*	Post-translational Modification, Assembly and Chaperones	Unknown
RCAP_rcc02866	3.67	0	746	10198	0.02%	0.14%	1021	1303	9021	11374	*pflB*	Carbohydrate Metabolism	Butanoate metabolism
RCAP_rcc02867	-1.81	0	99	27	0.00%	0.00%	76	114	22	32	*hypothetical protein*	Unknown	Unknown
RCAP_rcc02868	-1.02	0	709	335	0.01%	0.00%	485	886	293	377	*S1/S6 family peptidase*	Post-translational Modification, Assembly and Chaperones	Peptidase
RCAP_rcc02869	1.19	0	175	415	0.00%	0.01%	141	220	353	478	*glcF*	Carbohydrate Metabolism	Glyoxylate and dicarboxylate metabolism
RCAP_rcc02870	1.42	0	67	199	0.00%	0.00%	42	93	124	275	*glcE*	Carbohydrate Metabolism	Glyoxylate and dicarboxylate metabolism
RCAP_rcc02871	1.35	0	481	1245	0.01%	0.02%	444	574	1107	1383	*glcD*	Carbohydrate Metabolism	Glyoxylate and dicarboxylate metabolism
RCAP_rcc02872	0.48	0.0011	245	345	0.00%	0.00%	209	289	315	374	*hypothetical protein*	Unknown	Unknown
RCAP_rcc02873	-0.74	0	1285	760	0.02%	0.01%	1052	1454	646	873	*kpsT2*	Carbohydrate Metabolism	Unknown
RCAP_rcc02874	-0.74	0	1805	1078	0.02%	0.01%	1617	1916	999	1157	*kpsE2*	Cell Envelope Biosynthesis	Cell Wall Biosynthesis
RCAP_rcc02875	-0.42	0	1121	840	0.02%	0.01%	1062	1148	796	884	*kdsA*	Glycan Biosynthesis and Metabolism	Lipopolysaccharide biosynthesis
RCAP_rcc02876	0.54	0	917	1346	0.01%	0.02%	841	1071	1184	1508	*DeoC/LacD family aldolase*	Carbohydrate Metabolism	Unknown
RCAP_rcc02877	0.03	0.9017	2214	2269	0.03%	0.03%	1906	2584	1771	2767	*monosacharide ABC transporter periplasmic monosacharide-binding p*	Carbohydrate Metabolism	Aerobic/Anaerobic Respiration
RCAP_rcc02878	-0.13	0.3581	257	235	0.00%	0.00%	229	282	206	264	*monosacharide ABC transporter permease*	Carbohydrate Metabolism	Aerobic/Anaerobic Respiration
RCAP_rcc02879	-0.06	0.7253	269	258	0.00%	0.00%	241	298	220	296	*monosacharide ABC transporter permease*	Carbohydrate Metabolism	Aerobic/Anaerobic Respiration
RCAP_rcc02880	0.09	0.5574	242	259	0.00%	0.00%	217	267	225	294	*monosacharide ABC transporter ATP-binding protein*	Carbohydrate Metabolism	Aerobic/Anaerobic Respiration
RCAP_rcc02881	0.29	0.5266	17	22	0.00%	0.00%	8	26	10	34	*rpe2*	Energy Metabolism	Carbon fixation in photosynthetic organisms
RCAP_rcc02882	0.36	0.0719	73	95	0.00%	0.00%	64	85	78	113	*antibiotic biosynthesis monooxygenase*	Unknown	Unknown
RCAP_rcc02883	0.1	0.638	283	305	0.00%	0.00%	225	337	259	350	*DeoR family transcriptional regulator*	Signal Transduction	Transcription Regulator

RCAP_rcc02884	-0.05	0.7503	348	338	0.00%	0.00%	316	372	296	379	*FGGY family carbohydrate kinase*	Carbohydrate Metabolism	Unknown
RCAP_rcc02885	-0.62	0	580	377	0.01%	0.01%	528	608	347	408	*dimethylglycine dehydrogenase*	Amino Acid Metabolism	Unknown
RCAP_rcc02886	1.14	0.0005	23	55	0.00%	0.00%	16	30	36	73	*XRE family transcriptional regulator*	Signal Transduction	Transcription Regulator
RCAP_rcc02887	-0.5	0.0012	1167	819	0.02%	0.01%	944	1350	757	881	*methyl-accepting chemotaxis sensory transducer*	Motility	Chemotaxis
RCAP_rcc02888	-0.38	0.329	61	45	0.00%	0.00%	40	77	16	73	*hypothetical protein*	Unknown	Unknown
RCAP_rcc02889	0.11	0.7986	457	497	0.01%	0.01%	317	581	174	821	*hypothetical protein*	Unknown	Unknown
RCAP_rcc02890	-0.62	0.232	889	481	0.01%	0.01%	497	1221	25	937	*hypothetical protein*	Unknown	Unknown
RCAP_rcc02891	-0.92	0.0702	3408	1337	0.05%	0.02%	1822	4723	131	2544	*hypothetical protein*	Unknown	Unknown
RCAP_rcc02892	-1.25	0.0037	8051	2660	0.11%	0.04%	5015	10400	1146	4173	*hypothetical protein*	Unknown	Unknown
RCAP_rcc02893	-1.29	0.0007	21434	7342	0.29%	0.10%	13889	27214	4423	10260	*cspA3*	Transcription	Unknown
RCAP_rcc02894	-0.66	0.1672	10	5	0.00%	0.00%	3	15	2	8	*mdtE*	Cell Envelope Biosynthesis	Cell Wall Biosynthesis
RCAP_rcc02895	-0.57	0	98	65	0.00%	0.00%	90	102	58	71	*mdtF*	Unknown	Unknown
RCAP_rcc02896	0.5	0.0017	34	48	0.00%	0.00%	29	40	44	53	*baeR*	Signal Transduction	Transcription Regulator
RCAP_rcc02897	-0.13	0.4984	41	37	0.00%	0.00%	37	42	30	44	*sensor histidine kinase*	Signal Transduction	Kinase/Phosphorelay
RCAP_rcc02898	0.22	0.3492	160	188	0.00%	0.00%	125	197	159	218	*ABC transporter ATP-binding/permease*	Unknown	Unknown
RCAP_rcc02899	-0.27	0.0716	40	33	0.00%	0.00%	34	45	30	36	*hypothetical protein*	Unknown	Unknown
RCAP_rcc02900	-0.55	0.0097	51	34	0.00%	0.00%	40	59	27	41	*IS3 family transposase*	Replication, Recombination and Repair	Recombination
RCAP_rcc02903	0.6	0.0046	17	26	0.00%	0.00%	15	22	22	30	*hypothetical protein*	Unknown	Unknown
RCAP_rcc02904	0.14	0.7784	2	2	0.00%	0.00%	1	3	1	3	*hypothetical protein*	Unknown	Unknown
RCAP_rcc02905	-0.02	0.9701	4	4	0.00%	0.00%	3	6	3	6	*hypothetical protein*	Unknown	Unknown
RCAP_rcc02906	0.06	0.8651	19	20	0.00%	0.00%	14	24	15	24	*hypothetical protein*	Unknown	Unknown
RCAP_rcc02907	0.3	0.0571	46	56	0.00%	0.00%	43	49	45	66	*hypothetical protein*	Unknown	Unknown
RCAP_rcc02908	1.42	0	3	9	0.00%	0.00%	3	4	7	11	*hypothetical protein*	Unknown	Unknown
RCAP_rcc02909	0.27	0.2701	10	12	0.00%	0.00%	9	12	9	15	*hypothetical protein*	Unknown	Unknown
RCAP_rcc02910	0.6	0.1936	3	5	0.00%	0.00%	2	4	2	8	*hypothetical protein*	Unknown	Unknown
RCAP_rcc02911	-0.18	0.2416	48	42	0.00%	0.00%	40	54	38	45	*hypothetical protein*	Unknown	Unknown
RCAP_rcc02912	0.49	0.2756	2	2	0.00%	0.00%	1	3	1	3	*hypothetical protein*	Unknown	Unknown
RCAP_rcc02913	0.21	0.3111	10	11	0.00%	0.00%	9	12	10	12	*hypothetical protein*	Unknown	Unknown
RCAP_rcc02914	0.36	0.0293	23	30	0.00%	0.00%	21	27	26	34	*hypothetical protein*	Unknown	Unknown
RCAP_rcc02915	-0.19	0.5899	4	3	0.00%	0.00%	3	4	2	3	*hypothetical protein*	Unknown	Unknown
RCAP_rcc02916	0.87	0.0659	1	3	0.00%	0.00%	0	2	1	4	*hypothetical protein*	Unknown	Unknown
RCAP_rcc02917	0.72	0.0317	3	5	0.00%	0.00%	2	4	4	7	*hypothetical protein*	Unknown	Unknown
RCAP_rcc02918	0.27	0.4698	4	4	0.00%	0.00%	3	5	3	6	*hypothetical protein*	Unknown	Unknown
RCAP_rcc02919	0.61	0	25	37	0.00%	0.00%	22	29	33	42	*hypothetical protein*	Unknown	Unknown
RCAP_rcc02920	1.07	0.0039	4	8	0.00%	0.00%	3	5	5	11	*hypothetical protein*	Unknown	Unknown
RCAP_rcc02921	1.25	0	30	75	0.00%	0.00%	25	38	53	97	*hypothetical protein*	Unknown	Unknown
RCAP_rcc02922	1.66	0	193	638	0.00%	0.01%	187	267	534	742	*phage virion morphogenesis protein*	Replication, Recombination and Repair	Phage Interaction
RCAP_rcc02923	1.86	0	670	2625	0.01%	0.04%	652	971	2079	3171	*hypothetical protein*	Unknown	Unknown
RCAP_rcc02924	-0.12	0.6892	18	16	0.00%	0.00%	12	22	13	19	*hypothetical protein*	Unknown	Unknown
RCAP_rcc02925	0.16	0.6232	5	6	0.00%	0.00%	4	7	5	7	*hypothetical protein*	Unknown	Unknown
RCAP_rcc02926	-0.35	0.2315	21	16	0.00%	0.00%	15	28	13	19	*hypothetical protein*	Unknown	Unknown
RCAP_rcc02927	-0.49	0.0423	21	15	0.00%	0.00%	15	26	13	17	*hypothetical protein*	Unknown	Unknown
RCAP_rcc02928	-0.18	0.437	504	443	0.01%	0.01%	388	609	365	521	*XRE family transcriptional regulator*	Signal Transduction	Transcription Regulator
RCAP_rcc02929	-0.79	0.0008	4594	2564	0.06%	0.03%	3341	5653	2047	3081	*hypothetical protein*	Unknown	Unknown
RCAP_rcc02930	-0.59	0.0146	1634	1059	0.02%	0.01%	1181	2049	867	1252	*hypothetical protein*	Unknown	Unknown
RCAP_rcc02931	-0.19	0.5544	16	14	0.00%	0.00%	11	21	10	18	*hypothetical protein*	Unknown	Unknown
RCAP_rcc02932	0.55	0.0268	24	36	0.00%	0.00%	19	31	29	42	*pinE*	Replication, Recombination and Repair	Unknown
RCAP_rcc02933	0.09	0.7514	202	217	0.00%	0.00%	146	263	183	251	*hypothetical protein*	Unknown	Unknown
RCAP_rcc02934	-0.4	0.104	262	195	0.00%	0.00%	192	326	162	228	*phage integrase*	Replication, Recombination and Repair	Phage Interaction
RCAP_rcc02935	0.43	0.0023	41	54	0.00%	0.00%	35	47	49	60	*hypothetical protein*	Unknown	Unknown
RCAP_rcc02936	-0.61	0.0058	7060	4522	0.10%	0.06%	5503	8375	3590	5453	*hypothetical protein*	Unknown	Unknown
RCAP_rcc02937	-1.05	0	3029	1397	0.04%	0.02%	2236	3655	1067	1728	*hypothetical protein*	Unknown	Unknown
RCAP_rcc02938	-1.12	0	594	267	0.01%	0.00%	476	676	231	303	*hypothetical protein*	Unknown	Unknown
RCAP_rcc02939	0.08	0.7553	2609	2756	0.04%	0.04%	1976	3236	2359	3153	*hypothetical protein*	Unknown	Unknown
RCAP_rcc02940	0.54	0.0384	14	21	0.00%	0.00%	10	18	17	26	*hypothetical protein*	Unknown	Unknown
RCAP_rcc02941	0.88	0.0884	1	2	0.00%	0.00%	0	2	1	4	*hypothetical protein*	Unknown	Unknown
RCAP_rcc02942	0.43	0.1731	5	6	0.00%	0.00%	3	6	5	7	*hypothetical protein*	Unknown	Unknown
RCAP_rcc02943	0.36	0.2257	9	11	0.00%	0.00%	6	11	8	14	*hypothetical protein*	Unknown	Unknown
RCAP_rcc02944	0.75	0.0027	4	7	0.00%	0.00%	4	5	6	8	*hypothetical protein*	Unknown	Unknown
RCAP_rcc02945	1.14	0.0001	10	23	0.00%	0.00%	8	14	16	30	*hypothetical protein*	Unknown	Unknown
RCAP_rcc02946	0.19	0.5349	9	10	0.00%	0.00%	6	11	8	12	*hypothetical protein*	Unknown	Unknown
RCAP_rcc02947	-0.1	0.4984	133	124	0.00%	0.00%	118	150	113	136	*hypothetical protein*	Unknown	Unknown
RCAP_rcc02948	0.26	0.2745	40	48	0.00%	0.00%	29	50	41	55	*hypothetical protein*	Unknown	Unknown
RCAP_rcc02949	0.24	0.2331	200	237	0.00%	0.00%	171	239	203	271	*hypothetical protein*	Unknown	Unknown
RCAP_rcc02950	-0.07	0.746	61	58	0.00%	0.00%	49	73	53	63	*hypothetical protein*	Unknown	Unknown
RCAP_rcc02951	-0.9	0.0007	61	31	0.00%	0.00%	41	77	25	37	*excisionase, DNA-binding domain-containing protein*	Unknown	Unknown
RCAP_rcc02952	-0.31	0.0454	122	99	0.00%	0.00%	101	140	91	107	*hypothetical protein*	Unknown	Unknown
RCAP_rcc02953	0.28	0.0583	396	482	0.01%	0.01%	343	464	440	523	*phage integrase*	Replication, Recombination and Repair	Phage Interaction
RCAP_rcc02954	-0.24	0.0007	4603	3888	0.06%	0.05%	4351	4795	3634	4141	*mechanosensitive ion channel family protein*	Unknown	Unknown
RCAP_rcc02955	0.15	0.1814	1496	1661	0.02%	0.02%	1352	1671	1556	1765	*cysK2*	Energy Metabolism	Sulfur metabolism
RCAP_rcc02956	0.18	0.394	362	411	0.01%	0.01%	297	420	331	490	*nudF*	Nucleotide Metabolism	Purine metabolism
RCAP_rcc02957	-0.16	0.2032	845	755	0.01%	0.01%	761	939	689	821	*hypothetical protein*	Unknown	Unknown
RCAP_rcc02958	1.63	0	268	919	0.00%	0.01%	231	366	530	1308	*phrB*	Replication, Recombination and Repair	Unknown
RCAP_rcc02959	0.09	0.7044	8830	9396	0.13%	0.13%	7391	10437	7746	11047	*potD5*	Amino Acid Metabolism	Amino Acid Transport
RCAP_rcc02960	-0.03	0.8899	1904	1865	0.03%	0.03%	1575	2221	1622	2107	*neuB*	Carbohydrate Metabolism	Amino sugar and nucleotide sugar metabolism
RCAP_rcc02961	0.02	0.8852	608	617	0.01%	0.01%	543	670	564	671	*family 25 glycosyl transferase*	Cell Envelope Biosynthesis	Cell Wall Biosynthesis
RCAP_rcc02962	-0.17	0.1896	526	469	0.01%	0.01%	464	581	429	508	*rhamnan synthesis protein F family*	Cell Envelope Biosynthesis	Cell Wall Biosynthesis

RCAP_rcc02963	0.16	0.2967	551	615	0.01%	0.01%	482	626	555	675	*hypothetical protein*	Unknown	Unknown
RCAP_rcc02964	-0.24	0.1285	769	648	0.01%	0.01%	668	856	561	734	*webC*	Carbohydrate Metabolism	Amino sugar and nucleotide sugar metabolism
RCAP_rcc02965	-0.41	0	872	655	0.01%	0.01%	795	928	596	713	*oxidoreductase family NAD-binding Rossmann fold protein*	Unknown	Unknown
RCAP_rcc02966	-0.32	0.0002	1019	814	0.01%	0.01%	920	1103	768	860	*short-chain dehydrogenase/reductase family oxidoreductase*	Metabolism of Cofactors, Coenzymes and Vitamins	Biotin metabolism
RCAP_rcc02967	-0.52	0	880	609	0.01%	0.01%	792	954	539	680	*cytidylyltransferase*	Carbohydrate Metabolism	Amino sugar and nucleotide sugar metabolism
RCAP_rcc02968	-0.17	0.2363	281	248	0.00%	0.00%	240	315	223	273	*hypothetical protein*	Unknown	Unknown
RCAP_rcc02969	0.17	0.1565	574	644	0.01%	0.01%	519	623	586	702	*hypothetical protein*	Unknown	Unknown
RCAP_rcc02970	-0.03	0.9063	3751	3658	0.05%	0.05%	2822	4661	3134	4182	*atpC*	Energy Metabolism	Methane metabolism
RCAP_rcc02971	-0.34	0.0386	46005	36077	0.65%	0.49%	39590	52335	31292	40861	*atpD*	Energy Metabolism	Methane metabolism
RCAP_rcc02972	-0.5	0.0107	24928	17448	0.35%	0.24%	20259	29273	14750	20146	*atpG*	Energy Metabolism	Methane metabolism
RCAP_rcc02973	-0.35	0.0691	47063	36622	0.66%	0.50%	38845	55154	31354	41890	*atpA*	Energy Metabolism	Methane metabolism
RCAP_rcc02974	-0.22	0.2459	3970	3385	0.06%	0.05%	3157	4740	3054	3716	*atpH*	Energy Metabolism	Methane metabolism
RCAP_rcc02975	0.3	0.1575	384	475	0.01%	0.01%	309	451	386	564	*hypothetical protein*	Unknown	Unknown
RCAP_rcc02976	0.56	0	574	855	0.01%	0.01%	507	670	800	910	*gloB*	Sulfur Metabolism	Glutathione metabolism
RCAP_rcc02977	0.66	0	15897	25295	0.24%	0.34%	15380	18545	22031	28560	*clpA*	Post-translational Modification, Assembly and Chaperones	Unknown
RCAP_rcc02978	0.11	0.6049	58	62	0.00%	0.00%	50	66	50	75	*M23 family peptidase*	Post-translational Modification, Assembly and Chaperones	Peptidase
RCAP_rcc02979	-0.15	0.4811	9302	8367	0.13%	0.11%	8151	10481	6770	9964	*rpsD*	Translation, ribosomal structure and biogenesis	Unknown
RCAP_rcc02980	-2.07	0	953	203	0.01%	0.00%	517	1311	183	222	*hypothetical protein*	Unknown	Unknown
RCAP_rcc02981	0.11	0.3835	3253	3504	0.05%	0.05%	2952	3573	3213	3796	*hisC1*	Amino Acid Metabolism	Histidine metabolism
RCAP_rcc02982	-0.43	0.0002	1650	1216	0.02%	0.02%	1426	1807	1119	1313	*tyrA*	Amino Acid Metabolism	Phenylalanine, tyrosine and tryptophan biosynthesis
RCAP_rcc02983	0.27	0.4306	110	135	0.00%	0.00%	74	144	109	160	*extensin family protein*	Unknown	Unknown
RCAP_rcc02984	0.5	0.236	59	90	0.00%	0.00%	30	86	49	132	*hypothetical protein*	Unknown	Unknown
RCAP_rcc02985	0.72	0.0001	36	60	0.00%	0.00%	29	45	54	66	*chaC*	Metal and Ion Transport	Unknown
RCAP_rcc02986	0.11	0.5284	594	644	0.01%	0.01%	498	680	548	739	*motA/TolQ/ExbB proton channel family protein*	Motility	Chemotaxis
RCAP_rcc02987	-0.27	0.0058	1511	1250	0.02%	0.02%	1342	1640	1172	1327	*OmpA/MotB domain-containing protein*	Motility	Chemotaxis
RCAP_rcc02988	0.15	0.5111	844	939	0.01%	0.01%	698	971	720	1159	*hypothetical protein*	Unknown	Unknown
RCAP_rcc02989	-0.42	0.1262	301	221	0.00%	0.00%	213	370	163	278	*hypothetical protein*	Unknown	Unknown
RCAP_rcc02990	0.27	0.3054	123	150	0.00%	0.00%	90	154	128	172	*YbaK/EbsC family protein*	Unknown	Unknown
RCAP_rcc02991	-0.06	0.8666	181	172	0.00%	0.00%	126	236	121	224	*hadH*	Unknown	Unknown
RCAP_rcc02992	-0.11	0.7284	292	269	0.00%	0.00%	210	362	186	352	*atoB1*	Carbohydrate Metabolism	Glyoxylate and dicarboxylate metabolism
RCAP_rcc02993	-0.13	0.3509	4732	4303	0.07%	0.06%	4139	5332	3879	4728	*ilvD*	Metabolism of Cofactors, Coenzymes and Vitamins	Pantothenate and CoA biosynthesis
RCAP_rcc02994	1.55	0	341	1064	0.01%	0.01%	325	434	725	1402	*exoD*	Unknown	Unknown
RCAP_rcc02995	0.96	0.0045	44	93	0.00%	0.00%	29	59	62	124	*hypothetical protein*	Unknown	Unknown
RCAP_rcc02996	0.82	0.0689	12	25	0.00%	0.00%	6	18	11	39	*hypothetical protein*	Unknown	Unknown
RCAP_rcc02997	0.01	0.9533	313	313	0.00%	0.00%	287	333	295	332	*phospholipid/glycerol acyltransferase*	Lipid Metabolism	Glycerophospholipid metabolism
RCAP_rcc02998	0.02	0.9103	482	488	0.01%	0.01%	425	529	445	530	*hypothetical protein*	Unknown	Unknown
RCAP_rcc02999	-0.26	0.1522	301	250	0.00%	0.00%	245	353	217	282	*hypothetical protein*	Unknown	Unknown
RCAP_rcc03000	-0.4	0.0001	860	652	0.01%	0.01%	776	924	593	710	*hypothetical protein*	Unknown	Unknown
RCAP_rcc03001	-0.13	0.348	1573	1440	0.02%	0.02%	1345	1784	1376	1504	*proA*	Amino Acid Metabolism	Arginine and proline metabolism
RCAP_rcc03002	-0.24	0.1991	735	621	0.01%	0.01%	606	849	541	701	*hypothetical protein*	Unknown	Unknown
RCAP_rcc03003	-0.59	0.0069	121	79	0.00%	0.00%	92	144	63	95	*hypothetical protein*	Unknown	Unknown
RCAP_rcc03004	-0.44	0.0246	619	452	0.01%	0.01%	470	739	375	528	*DeoR family transcriptional regulator*	Signal Transduction	Transcription Regulator
RCAP_rcc03005	0.54	0.0019	1554	2279	0.02%	0.03%	1297	1878	2004	2554	*diguanylate cyclase/phosphodiesterase*	Signal Transduction	Kinase/Phosphorelay
RCAP_rcc03006	0.57	0.1091	9	14	0.00%	0.00%	5	13	9	19	*apbE*	Metabolism of Cofactors, Coenzymes and Vitamins	Thiamine metabolism
RCAP_rcc03007	-0.59	0.0561	48	31	0.00%	0.00%	25	69	26	35	*cysJ*	Energy Metabolism	Unknown
RCAP_rcc03008	-1.33	0	106	38	0.00%	0.00%	53	154	32	44	*hypothetical protein*	Unknown	Unknown
RCAP_rcc03009	-2.12	NA	70	11	0.00%	0.00%	22	113	8	14	*hypothetical protein*	Unknown	Unknown
RCAP_rcc03010	-1.15	0.0042	397	149	0.01%	0.00%	114	663	112	186	*hypothetical protein*	Unknown	Unknown
RCAP_rcc03011	0.1	0.723	228	245	0.00%	0.00%	148	308	216	274	*winged helix family two component transcriptional regulator*	Signal Transduction	Transcription Regulator
RCAP_rcc03012	0.97	0	180	355	0.00%	0.00%	161	210	323	388	*signal transduction histidine kinase*	Signal Transduction	Kinase/Phosphorelay
RCAP_rcc03013	1.88	0	825	3091	0.01%	0.04%	903	1027	2768	3414	*DeoC/LacD family aldolase*	Carbohydrate Metabolism	Unknown
RCAP_rcc03014	-0.27	0.0452	2702	2234	0.04%	0.03%	2298	3073	2068	2399	*methyl-accepting chemotaxis sensory transducer*	Motility	Chemotaxis
RCAP_rcc03015	0.55	0	658	969	0.01%	0.01%	583	741	875	1064	*kefC2*	Sulfur Metabolism	Glutathione metabolism
RCAP_rcc03016	0.33	0.0407	191	241	0.00%	0.00%	166	216	211	272	*draT*	Energy Metabolism	Nitrogen metabolism
RCAP_rcc03017	0.3	0.1759	130	162	0.00%	0.00%	101	158	135	190	*draG*	Energy Metabolism	Nitrogen metabolism
RCAP_rcc03018	-0.36	0.3794	61	45	0.00%	0.00%	30	88	32	59	*hypothetical protein*	Unknown	Unknown
RCAP_rcc03022	-2.64	0	3950	573	0.05%	0.01%	2906	4635	457	689	*dctM3*	Carbohydrate Metabolism	Unknown
RCAP_rcc03023	-2.38	0	1888	327	0.03%	0.00%	1251	2354	263	390	*dctQ3*	Carbohydrate Metabolism	Unknown
RCAP_rcc03024	-2.31	0	23800	4305	0.32%	0.06%	18415	27119	3098	5512	*dctP3*	Carbohydrate Metabolism	Unknown
RCAP_rcc03025	-0.52	0.0083	424	292	0.01%	0.00%	335	493	250	333	*dctS3*	Signal Transduction	Kinase/Phosphorelay
RCAP_rcc03026	-0.55	0.0003	131	89	0.00%	0.00%	108	149	80	98	*dctR3*	Signal Transduction	Transcription Regulator
RCAP_rcc03027	0.94	0	207	398	0.00%	0.01%	192	230	349	447	*radical SAM family protein*	Unknown	Unknown
RCAP_rcc03028	0.3	0.2533	40	48	0.00%	0.00%	32	45	34	63	*hypothetical protein*	Unknown	Unknown
RCAP_rcc03029	-0.76	NA	899	500	0.01%	0.01%	461	1368	320	680	*hypothetical protein*	Unknown	Unknown
RCAP_rcc03030	-0.66	NA	1332	758	0.02%	0.01%	497	2286	283	1234	*YiaA/B two helix domain-containing family protein*	Unknown	Unknown
RCAP_rcc03031	0.03	NA	339	348	0.01%	0.00%	150	604	60	636	*PspA/IM30 family protein*	Signal Transduction	Unknown
RCAP_rcc03032	-0.34	0.1194	84	65	0.00%	0.00%	69	100	55	76	*TetR family transcriptional regulator*	Signal Transduction	Transcription Regulator
RCAP_rcc03033	-1.09	0.0028	28	11	0.00%	0.00%	13	41	8	15	*fdhE*	Carbohydrate Metabolism	Glyoxylate and dicarboxylate metabolism
RCAP_rcc03034	-1.97	0	445	107	0.01%	0.00%	362	494	88	126	*fdhD*	Energy Metabolism	Nitrogen metabolism
RCAP_rcc03035	-1.71	0	8524	2508	0.12%	0.03%	7555	8983	2029	2988	*fdhA*	Carbohydrate Metabolism	Glyoxylate and dicarboxylate metabolism
RCAP_rcc03036	-1.51	0	2625	899	0.04%	0.01%	2171	2924	755	1044	*fdhB*	Carbohydrate Metabolism	Glyoxylate and dicarboxylate metabolism
RCAP_rcc03037	-1.07	0	903	421	0.01%	0.01%	709	1047	363	479	*fdhC*	Carbohydrate Metabolism	Glyoxylate and dicarboxylate metabolism
RCAP_rcc03038	0.29	0.0003	497	609	0.01%	0.01%	465	536	569	649	*moaE*	Metabolism of Cofactors, Coenzymes and Vitamins	Unknown
RCAP_rcc03039	0.51	0.2225	63	97	0.00%	0.00%	32	94	58	137	*moaD2*	Metabolism of Cofactors, Coenzymes and Vitamins	Unknown
RCAP_rcc03040	-1.23	0	2140	844	0.03%	0.01%	1364	2767	653	1035	*hypothetical protein*	Unknown	Unknown
RCAP_rcc03041	-0.56	0.0003	1333	896	0.02%	0.01%	1109	1503	780	1013	*thyX*	Nucleotide Metabolism	Unknown
RCAP_rcc03042	0.16	0.1413	3514	3939	0.05%	0.05%	3202	3970	3678	4201	*lipoprotein*	Predicted Function	Unknown

RCAP_rcc03043	-0.37	0.0204	350	269	0.00%	0.00%	294	396	239	298	*hypothetical protein*	Unknown	Unknown
RCAP_rcc03044	0.69	0	1503	2451	0.02%	0.03%	1380	1775	2191	2711	*gloA*	Sulfur Metabolism	Glutathione metabolism
RCAP_rcc03045	0.37	0.0193	583	758	0.01%	0.01%	482	685	671	846	*hypothetical protein*	Unknown	Unknown
RCAP_rcc03046	0.05	0.8127	1106	1151	0.02%	0.02%	937	1286	977	1325	*engD*	Translation, ribosomal structure and biogenesis	Unknown
RCAP_rcc03047	-0.12	0.4556	490	450	0.01%	0.01%	405	578	421	479	*trpA*	Amino Acid Metabolism	Phenylalanine, tyrosine and tryptophan biosynthesis
RCAP_rcc03048	0.89	0	1920	3573	0.03%	0.05%	1865	2109	3228	3919	*lldD*	Carbohydrate Metabolism	Pyruvate metabolism
RCAP_rcc03049	-0.83	0.0016	863	466	0.01%	0.01%	567	1112	383	548	*major facilitator superfamily protein*	Carbohydrate Metabolism	Unknown
RCAP_rcc03050	-0.09	0.7553	11892	11109	0.17%	0.15%	9207	14666	8366	13851	*rplY*	Translation, ribosomal structure and biogenesis	Unknown
RCAP_rcc03051	-1.49	0	252	88	0.00%	0.00%	207	281	79	97	*pth*	Translation, ribosomal structure and biogenesis	Unknown
RCAP_rcc03052	-1.68	0	1451	435	0.02%	0.01%	1040	1748	409	461	*hypothetical protein*	Unknown	Unknown
RCAP_rcc03053	-1.28	0	195	74	0.00%	0.00%	128	248	60	88	*hypothetical protein*	Unknown	Unknown
RCAP_rcc03054	-0.29	0.1312	12125	9876	0.17%	0.13%	9765	14465	8587	11166	*rpoD*	Replication, Recombination and Repair	Replication
RCAP_rcc03055	0.78	0	1725	2979	0.03%	0.04%	1679	2029	2695	3264	*dnaG*	Replication, Recombination and Repair	Unknown
RCAP_rcc03056	-0.45	0	4983	3648	0.07%	0.05%	4521	5380	3324	3971	*hypothetical protein*	Unknown	Unknown
RCAP_rcc03057	-0.76	0	889	519	0.01%	0.01%	737	1000	462	576	*hypothetical protein*	Unknown	Unknown
RCAP_rcc03058	-1.02	0	1178	573	0.02%	0.01%	980	1315	510	636	*hypothetical protein*	Unknown	Unknown
RCAP_rcc03059	0.33	0.2077	158	200	0.00%	0.00%	125	186	146	255	*TetR family transcriptional regulator*	Signal Transduction	Transcription Regulator
RCAP_rcc03060	1.54	0	7	23	0.00%	0.00%	5	10	14	33	*lipoprotein*	Predicted Function	Unknown
RCAP_rcc03061	0.06	0.5168	2102	2199	0.03%	0.03%	1921	2269	2067	2330	*hom*	Amino Acid Metabolism	Lysine biosynthesis
RCAP_rcc03062	0.63	0	1292	2017	0.02%	0.03%	1170	1483	1842	2192	*glpX*	Carbohydrate Metabolism	Glycolysis / Gluconeogenesis
RCAP_rcc03063	0.42	0.0656	232	314	0.00%	0.00%	182	272	240	389	*recJ*	Replication, Recombination and Repair	Unknown
RCAP_rcc03064	0.49	0.1332	254	370	0.00%	0.01%	183	329	243	496	*HPP family/CBS domain-containing protein*	Signal Transduction	Kinase/Phosphorelay
RCAP_rcc03065	-1.48	0	635	199	0.01%	0.00%	386	829	109	288	*efeU*	Metal and Ion Transport	Unknown
RCAP_rcc03066	-3.03	0	1673	196	0.02%	0.00%	1398	1793	164	227	*dyp-type peroxidase*	Metal and Ion Transport	Unknown
RCAP_rcc03067	-4.14	0	4453	231	0.06%	0.00%	3557	4907	186	275	*hypothetical protein*	Unknown	Unknown
RCAP_rcc03068	-0.76	0	908	530	0.01%	0.01%	783	994	485	575	*cache sensor protein*	Signal Transduction	Kinase/Phosphorelay
RCAP_rcc03069	0.12	0.5153	185	201	0.00%	0.00%	151	217	182	220	*hypothetical protein*	Unknown	Unknown
RCAP_rcc03070	0.28	0.1652	325	397	0.00%	0.01%	256	399	346	448	*hypothetical protein*	Unknown	Unknown
RCAP_rcc03071	-0.6	0	837	550	0.01%	0.01%	727	919	522	577	*hypothetical protein*	Unknown	Unknown
RCAP_rcc03072	-0.03	0.8263	3024	2951	0.04%	0.04%	2840	3340	2566	3336	*hypothetical protein*	Unknown	Unknown
RCAP_rcc03073	-0.14	0.1243	1010	915	0.01%	0.01%	943	1066	844	986	*nusB*	Transcription	Unknown
RCAP_rcc03074	-0.01	0.9238	1500	1488	0.02%	0.02%	1356	1641	1386	1589	*ribH*	Metabolism of Cofactors, Coenzymes and Vitamins	Riboflavin metabolism
RCAP_rcc03075	-0.07	0.6751	5651	5363	0.08%	0.07%	4797	6524	4782	5943	*ribAB*	Metabolism of Cofactors, Coenzymes and Vitamins	Riboflavin metabolism
RCAP_rcc03076	0.35	0.0081	1024	1309	0.01%	0.02%	931	1159	1178	1441	*ribE*	Metabolism of Cofactors, Coenzymes and Vitamins	Riboflavin metabolism
RCAP_rcc03077	-0.36	0	316	246	0.00%	0.00%	290	331	232	261	*capsule polysaccharide modification protein family*	Cell Envelope Biosynthesis	Cell Wall Biosynthesis
RCAP_rcc03078	0.26	0.0052	2258	2709	0.03%	0.04%	2087	2510	2548	2870	*polysaccharide biosynthesis/export family protein*	Cell Envelope Biosynthesis	Cell Wall Biosynthesis
RCAP_rcc03079	-0.17	0.2577	373	331	0.01%	0.00%	316	421	297	364	*capsule polysaccharide biosynthesis protein family*	Cell Envelope Biosynthesis	Cell Wall Biosynthesis
RCAP_rcc03080	-0.01	0.9849	133	132	0.00%	0.00%	58	198	64	200	*ribD*	Metabolism of Cofactors, Coenzymes and Vitamins	Riboflavin metabolism
RCAP_rcc03081	-0.14	0.3869	1944	1763	0.03%	0.02%	1674	2204	1543	1983	*nrdR*	Signal Transduction	Transcription Regulator
RCAP_rcc03082	0.92	0	785	1491	0.01%	0.02%	767	894	1404	1578	*lipoprotein*	Predicted Function	Unknown
RCAP_rcc03083	-0.02	0.9284	322	317	0.00%	0.00%	273	387	257	377	*membrane bound YbgT-like protein*	Unknown	Unknown
RCAP_rcc03084	0.02	0.9356	2112	2140	0.03%	0.03%	1729	2605	1877	2403	*cydB*	Energy Metabolism	Aerobic/Anaerobic Respiration
RCAP_rcc03085	0.26	0.2131	3611	4339	0.05%	0.06%	3167	4338	3529	5149	*cydA*	Energy Metabolism	Aerobic/Anaerobic Respiration
RCAP_rcc03086	1.88	0	201	848	0.00%	0.01%	175	297	520	1176	*hypothetical protein*	Unknown	Unknown
RCAP_rcc03087	0.32	0.067	211	265	0.00%	0.00%	179	246	238	292	*GNAT family acetyltransferase*	Cell Division	Chromosome Partitioning
RCAP_rcc03088	0.72	0	146	244	0.00%	0.00%	136	171	221	266	*flavin reductase domain-containing protein*	Unknown	Unknown
RCAP_rcc03089	0.14	0.1675	888	979	0.01%	0.01%	801	969	916	1042	*group 1 glycosyl transferase*	Glycan Biosynthesis and Metabolism	N-Glycan biosynthesis
RCAP_rcc03090	0.99	0	307	618	0.00%	0.01%	280	359	521	716	*hypothetical protein*	Unknown	Unknown
RCAP_rcc03091	1.36	0	784	2051	0.01%	0.03%	748	973	1778	2324	*mtnP*	Amino Acid Metabolism	Cysteine and methionine metabolism
RCAP_rcc03092	1.18	0	819	1893	0.01%	0.03%	833	966	1591	2196	*apt*	Nucleotide Metabolism	Purine metabolism
RCAP_rcc03093	0.07	0.7078	239	250	0.00%	0.00%	202	269	215	284	*D-2-hydroxyglutarate dehydrogenase*	Energy Metabolism	Unknown
RCAP_rcc03094	-0.27	0.0916	367	303	0.01%	0.00%	305	414	267	339	*rimJ*	Translation, ribosomal structure and biogenesis	Unknown
RCAP_rcc03095	-0.69	0	2592	1587	0.04%	0.02%	2189	2889	1391	1784	*M16 family peptidase*	Post-translational Modification, Assembly and Chaperones	Peptidase
RCAP_rcc03096	-0.28	0.0741	2661	2179	0.04%	0.03%	2285	2987	1926	2432	*thrC*	Metabolism of Cofactors, Coenzymes and Vitamins	Vitamin B6 metabolism
RCAP_rcc03097	0.42	0.0274	616	835	0.01%	0.01%	499	743	720	950	*tldD*	Unknown	Unknown
RCAP_rcc03098	-0.15	0.4505	245	221	0.00%	0.00%	199	293	197	245	*drpA*	Replication, Recombination and Repair	Unknown
RCAP_rcc03099	0.21	0.2124	141	164	0.00%	0.00%	124	162	141	188	*hypothetical protein*	Unknown	Unknown
RCAP_rcc03100	-0.21	0.1175	3507	3021	0.05%	0.04%	3097	3862	2718	3325	*topA*	Replication, Recombination and Repair	Unknown
RCAP_rcc03101	-0.17	0.2268	1800	1592	0.03%	0.02%	1578	2020	1417	1768	*scoA*	Carbohydrate Metabolism	Butanoate metabolism
RCAP_rcc03102	-0.29	0.0474	2614	2127	0.04%	0.03%	2237	2962	1892	2362	*scoB*	Carbohydrate Metabolism	Butanoate metabolism
RCAP_rcc03103	-0.54	0.0244	305	204	0.00%	0.00%	221	366	161	248	*hypothetical protein*	Unknown	Unknown
RCAP_rcc03104	-0.56	0.0001	215	144	0.00%	0.00%	178	241	132	156	*FAD linked oxidase domain-containing protein*	Energy Metabolism	Unknown
RCAP_rcc03105	-0.56	0.0026	183	122	0.00%	0.00%	144	219	110	135	*short-chain dehydrogenase/reductase family oxidoreductase*	Lipid transport and metabolism	Unknown
RCAP_rcc03106	-0.19	0.5354	121	105	0.00%	0.00%	90	153	76	133	*enoyl-CoA hydratase/isomerase*	Xenobiotics Biodegradation and Metabolism	Caprolactam degradation
RCAP_rcc03107	-0.32	0.0019	520	416	0.01%	0.01%	458	559	387	445	*hisC2*	Amino Acid Metabolism	Histidine metabolism
RCAP_rcc03108	-0.57	0	996	667	0.01%	0.01%	905	1075	615	719	*hypothetical protein*	Unknown	Unknown
RCAP_rcc03109	-0.3	0.1963	1370	1099	0.02%	0.01%	1043	1673	969	1228	*serine--glyoxylate aminotransferase/alanine--glyoxylate aminotransfera*	Carbohydrate Metabolism	Glyoxylate and dicarboxylate metabolism
RCAP_rcc03110	0.33	0	847	1065	0.01%	0.01%	789	921	1027	1104	*ABC transporter ATP-binding/permease*	Defense Mechanisms	Unknown
RCAP_rcc03111	0.22	0.0903	410	479	0.01%	0.01%	362	464	432	526	*gcvT2*	Unknown	Unknown
RCAP_rcc03112	0.51	0.0001	2035	2910	0.03%	0.04%	1880	2374	2614	3205	*hypothetical protein*	Unknown	Unknown
RCAP_rcc03113	0.25	0.2201	1278	1525	0.02%	0.02%	1102	1445	1177	1872	*hypothetical protein*	Unknown	Unknown
RCAP_rcc03114	-0.15	0.1174	5728	5144	0.08%	0.07%	5076	6350	4964	5323	*TolC family type I secretion outer membrane protein*	Trafficking and Secretion	Secretion
RCAP_rcc03115	-0.38	0.0023	1615	1240	0.02%	0.02%	1410	1763	1110	1370	*pcm2*	Post-translational Modification, Assembly and Chaperones	Unknown
RCAP_rcc03116	-0.21	0.5619	19	16	0.00%	0.00%	13	25	10	22	*hypothetical protein*	Unknown	Unknown
RCAP_rcc03117	0.97	0.0153	7	16	0.00%	0.00%	5	10	8	24	*inositol monophosphatase*	Unknown	Unknown
RCAP_rcc03118	1.01	0	85	175	0.00%	0.00%	74	105	148	202	*hypothetical protein*	Unknown	Unknown
RCAP_rcc03119	0.69	0	431	703	0.01%	0.01%	397	510	615	790	*ion transport 2 family protein*	Metal and Ion Transport	Unknown

RCAP_rcc03120	-0.08	0.7389	9060	8554	0.13%	0.12%	7221	10991	7266	9842	*ABC transporter ATP-binding protein*	Unknown	Unknown
RCAP_rcc03121	-0.82	0.0059	36	19	0.00%	0.00%	24	46	15	24	*hypothetical protein*	Unknown	Unknown
RCAP_rcc03122	0.31	0.2937	866	1087	0.01%	0.01%	607	1165	831	1343	*FRG domain-containing protein*	Unknown	Unknown
RCAP_rcc03123	-0.48	0.0102	4597	3265	0.06%	0.04%	3773	5351	2781	3749	*lepA*	Cell Envelope Biosynthesis	Cell Wall Biosynthesis
RCAP_rcc03124	-1.46	NA	982	325	0.01%	0.00%	418	1481	277	374	*hypothetical protein*	Unknown	Unknown
RCAP_rcc03125	0.49	0	677	954	0.01%	0.01%	658	777	847	1060	*heavy metal transport/detoxification protein family*	Metal, Ion, Cofactor Transport	Copper Transport
RCAP_rcc03126	0.17	0.1618	423	477	0.01%	0.01%	371	470	436	517	*hypothetical protein*	Unknown	Unknown
RCAP_rcc03127	0.68	0.0109	133	220	0.00%	0.00%	95	173	171	268	*etp*	Signal Transduction	Kinase/Phosphorelay
RCAP_rcc03128	0.1	0.5682	212	227	0.00%	0.00%	180	240	196	258	*npdA*	Transcription	Unknown
RCAP_rcc03129	-0.61	0.0002	8426	5439	0.12%	0.07%	7262	9418	4568	6309	*rpmB*	Translation, ribosomal structure and biogenesis	Unknown
RCAP_rcc03130	-0.23	0.2225	385	327	0.01%	0.00%	308	442	281	373	*argK*	Amino Acid Metabolism	Unknown
RCAP_rcc03131	0.62	0.0056	420	660	0.01%	0.01%	339	552	562	758	*hypothetical protein*	Unknown	Unknown
RCAP_rcc03132	-0.14	0.454	643	582	0.01%	0.01%	541	725	485	680	*hrpB*	Replication, Recombination and Repair	Unknown
RCAP_rcc03133	-0.32	0.0005	304	243	0.00%	0.00%	286	317	218	269	*entB*	Secondary metabolites biosynthesis, transport, and catabolism	Unknown
RCAP_rcc03134	-0.3	0.0475	1947	1573	0.03%	0.02%	1737	2145	1338	1808	*argF*	Amino Acid Metabolism	Arginine and proline metabolism
RCAP_rcc03135	0.1	0.4384	1188	1270	0.02%	0.02%	1106	1324	1146	1394	*argD*	Amino Acid Metabolism	Arginine and proline metabolism
RCAP_rcc03136	-0.11	0.1213	3602	3337	0.05%	0.05%	3435	3769	3128	3546	*ccmF*	Energy Metabolism	Cytochrome Biogenesis
RCAP_rcc03137	-0.38	0.0519	424	322	0.01%	0.00%	340	495	277	367	*ccmH*	Energy Metabolism	Cytochrome Biogenesis
RCAP_rcc03138	-0.37	0.1393	375	285	0.01%	0.00%	282	453	214	357	*paaG*	Xenobiotics Biodegradation and Metabolism	Caprolactam degradation
RCAP_rcc03139	0.58	0	755	1130	0.01%	0.02%	745	809	1081	1178	*ABC transporter ATP-binding protein/permease*	Defense Mechanisms	Unknown
RCAP_rcc03140	-0.32	0.0209	6680	5328	0.09%	0.07%	5752	7706	4809	5848	*fadD*	Lipid Metabolism	Fatty acid metabolism
RCAP_rcc03141	-0.34	0.05	24032	18810	0.34%	0.26%	20724	27119	15273	22348	*cspD*	Transcription	Unknown
RCAP_rcc03142	-0.06	0.6159	1770	1699	0.02%	0.02%	1634	1912	1549	1849	*MiaB family RNA modification enzyme*	Translation, ribosomal structure and biogenesis	Unknown
RCAP_rcc03143	-0.24	0.3206	646	542	0.01%	0.01%	468	812	454	630	*hypothetical protein*	Unknown	Unknown
RCAP_rcc03144	0.29	0.0043	283	348	0.00%	0.00%	264	309	317	380	*GcrA cell cycle regulator*	Signal Transduction	Transcription Regulator
RCAP_rcc03145	-0.06	0.8862	452	434	0.01%	0.01%	262	628	347	521	*AsnC/Lrp family transcriptional regulator*	Signal Transduction	Transcription Regulator
RCAP_rcc03146	0.15	0.3225	4787	5315	0.07%	0.07%	4133	5611	4777	5853	*gabT1*	Carbohydrate Metabolism	Butanoate metabolism
RCAP_rcc03147	0.67	0.0089	99	162	0.00%	0.00%	71	126	117	207	*MerR family transcriptional regulator*	Signal Transduction	Transcription Regulator
RCAP_rcc03148	0.7	0.0092	164	275	0.00%	0.00%	125	216	233	317	*hypothetical protein*	Unknown	Unknown
RCAP_rcc03149	-0.28	0.0498	752	619	0.01%	0.01%	625	848	557	682	*S54 family peptidase*	Post-translational Modification, Assembly and Chaperones	Peptidase
RCAP_rcc03150	-0.3	0.0997	236	189	0.00%	0.00%	186	277	165	213	*alkJ*	Amino Acid Metabolism	Glycine, serine and threonine metabolism
RCAP_rcc03151	-0.17	0.1451	6984	6186	0.10%	0.08%	6327	7594	5602	6769	*pheT*	Translation, ribosomal structure and biogenesis	Aminoacyl-tRNA biosynthesis
RCAP_rcc03152	-0.75	0.0089	5470	3107	0.07%	0.04%	3556	7115	2465	3749	*hypothetical protein*	Unknown	Unknown
RCAP_rcc03153	-0.16	0.4756	2538	2257	0.04%	0.03%	1950	3107	1983	2530	*pheS*	Translation, ribosomal structure and biogenesis	Aminoacyl-tRNA biosynthesis
RCAP_rcc03154	0.12	0.4743	3776	4125	0.05%	0.06%	3416	4298	3459	4790	*rplT*	Translation, ribosomal structure and biogenesis	Unknown
RCAP_rcc03155	-0.12	0.6521	3984	3648	0.06%	0.05%	3138	4866	2829	4467	*rpmI*	Translation, ribosomal structure and biogenesis	Unknown
RCAP_rcc03156	-0.1	0.5942	437	407	0.01%	0.01%	365	489	345	468	*hypothetical protein*	Unknown	Unknown
RCAP_rcc03157	0.16	0.2634	2573	2884	0.04%	0.04%	2226	2867	2565	3202	*pykA2*	Carbohydrate Metabolism	Glycolysis / Gluconeogenesis
RCAP_rcc03158	1.42	0	196	533	0.00%	0.01%	190	235	453	613	*n-formylglutamate amidohydrolase*	Amino Acid Metabolism	Unknown
RCAP_rcc03159	0.89	0	2013	3803	0.03%	0.05%	1705	2476	3551	4055	*hemolysin-type calcium-binding repeat family protein*	Trafficking and Secretion	Secretion
RCAP_rcc03160	-0.24	0.4489	285	237	0.00%	0.00%	201	354	163	311	*hypothetical protein*	Unknown	Unknown
RCAP_rcc03161	-1.28	0	16563	6605	0.23%	0.09%	13152	19140	5690	7521	*dat*	Metabolism of Other Amino Acids	D-Alanine metabolism
RCAP_rcc03162	-1.52	0	9904	3263	0.13%	0.04%	6491	12605	2917	3609	*mandelate racemase/muconate lactonizing enzyme family protein*	Unknown	Unknown
RCAP_rcc03163	-1.2	0	23316	9738	0.32%	0.13%	16463	28891	8720	10756	*hypothetical protein*	Unknown	Unknown
RCAP_rcc03164	0.4	0.0979	136	182	0.00%	0.00%	118	167	126	239	*citE2*	Carbohydrate Metabolism	Glyoxylate and dicarboxylate metabolism
RCAP_rcc03165	1.15	0	2100	4704	0.03%	0.06%	2090	2503	4281	5126	*accA*	Energy Metabolism	Reductive carboxylate cycle (CO2 fixation)
RCAP_rcc03166	-0.24	0.0123	1511	1277	0.02%	0.02%	1388	1605	1179	1376	*phospholipid/glycerol acyltransferase*	Lipid Metabolism	Unknown
RCAP_rcc03167	0.38	0.207	273	363	0.00%	0.00%	192	352	269	456	*hypothetical protein*	Unknown	Unknown
RCAP_rcc03168	0.2	0.0163	830	955	0.01%	0.01%	780	886	885	1026	*ftsE*	Cell Division	Chromosome Partitioning
RCAP_rcc03169	0.53	0.046	495	732	0.01%	0.01%	357	613	514	949	*zinc finger domain-containing protein*	Metal, Ion, Cofactor Transport	Zinc Transport
RCAP_rcc03170	-0.27	0.0378	1687	1393	0.02%	0.02%	1448	1877	1253	1532	*hypothetical protein*	Unknown	Unknown
RCAP_rcc03171	-0.19	0.1677	1329	1167	0.02%	0.02%	1153	1487	1077	1257	*lysA*	Amino Acid Metabolism	Lysine biosynthesis
RCAP_rcc03172	-0.43	0.1005	72	52	0.00%	0.00%	51	87	38	66	*lipoprotein*	Predicted Function	Unknown
RCAP_rcc03173	-0.19	0.7184	22	18	0.00%	0.00%	7	36	8	29	*lipoprotein*	Predicted Function	Unknown
RCAP_rcc03174	-0.16	0.1317	2174	1946	0.03%	0.03%	2014	2323	1771	2121	*argH1*	Amino Acid Metabolism	Arginine and proline metabolism
RCAP_rcc03175	-0.23	0.0418	721	612	0.01%	0.01%	642	801	564	660	*dsbE*	Sulfur Metabolism	Unknown
RCAP_rcc03176	-1.27	0	2206	895	0.03%	0.01%	1757	2535	844	947	*PAS/PAC sensor domain-containing protein*	Motility	Aerotaxis
RCAP_rcc03177	-1.19	0	2193	944	0.03%	0.01%	1773	2493	857	1032	*EAL domain-containing protein*	Signal Transduction	Kinase/Phosphorelay
RCAP_rcc03178	-0.27	0.0442	4720	3909	0.07%	0.05%	4137	5236	3484	4335	*atoB2*	Carbohydrate Metabolism	Polyhydroxybutyrate
RCAP_rcc03179	-0.48	0	2067	1479	0.03%	0.02%	1845	2258	1343	1615	*phbB*	Carbohydrate Metabolism	Polyhydroxybutyrate
RCAP_rcc03180	-0.33	0.0674	2998	2364	0.04%	0.03%	2473	3641	2055	2672	*hypothetical protein*	Unknown	Unknown
RCAP_rcc03181	-0.36	0.0396	16358	12691	0.23%	0.17%	14039	18860	10786	14596	*hypothetical protein*	Unknown	Unknown
RCAP_rcc03182	-0.04	0.8263	137	132	0.00%	0.00%	116	153	114	151	*methyltransferase small domain-containing protein*	Unknown	Unknown
RCAP_rcc03183	-0.11	0.6268	166	153	0.00%	0.00%	131	197	131	176	*hypothetical protein*	Unknown	Unknown
RCAP_rcc03184	0.29	0.019	997	1225	0.01%	0.02%	904	1124	1128	1321	*ispB*	Lipid	Terpenoid backbone biosynthesis
RCAP_rcc03185	0.53	0.0562	137	202	0.00%	0.00%	100	168	136	268	*hypothetical protein*	Unknown	Unknown
RCAP_rcc03186	-0.84	0	3550	1963	0.05%	0.03%	3040	3925	1699	2227	*ATPase AAA*	Post-translational Modification, Assembly and Chaperones	Unknown
RCAP_rcc03187	1.35	0	661	1705	0.01%	0.02%	647	722	1475	1935	*AlgR/AgrA/LytR family transcriptional regulator*	Signal Transduction	Transcription Regulator
RCAP_rcc03188	-0.23	0.0666	2024	1728	0.03%	0.02%	1828	2214	1570	1886	*folD2*	Energy Metabolism	Reductive carboxylate cycle (CO2 fixation)
RCAP_rcc03189	0.05	0.8669	191	198	0.00%	0.00%	144	234	161	235	*chorismate mutase*	Amino Acid Metabolism	Unknown
RCAP_rcc03190	-0.09	0.6218	750	702	0.01%	0.01%	631	856	636	769	*fhs*	Energy Metabolism	Reductive carboxylate cycle (CO2 fixation)
RCAP_rcc03191	0.55	0	575	849	0.01%	0.01%	534	635	774	925	*hemolysin-type calcium-binding repeat family protein*	Trafficking and Secretion	Secretion
RCAP_rcc03192	0.63	0	16223	25369	0.24%	0.34%	15449	19129	22208	28530	*ftsH*	Post-translational Modification, Assembly and Chaperones	Unknown
RCAP_rcc03193	-0.29	0.1397	654	530	0.01%	0.01%	540	741	428	633	*tilS*	Cell Division	Chromosome Partitioning
RCAP_rcc03194	-0.15	0.5087	1050	941	0.01%	0.01%	805	1245	751	1130	*hypothetical protein*	Unknown	Unknown
RCAP_rcc03195	0.16	0.3523	3372	3777	0.05%	0.05%	2925	3983	3413	4142	*OmpA/MotB domain-containing protein*	Cell Envelope Biosynthesis	Cell Wall Biosynthesis
RCAP_rcc03196	-0.17	0.0656	5631	5002	0.08%	0.07%	5174	6082	4691	5314	*tolB*	Trafficking and Secretion	Secretion

RCAP_rcc03197	-0.26	0.0332	1482	1235	0.02%	0.02%	1298	1616	1118	1351	*tolA*	Trafficking and Secretion	Secretion
RCAP_rcc03198	-0.02	0.9102	1110	1097	0.02%	0.01%	977	1245	1010	1184	*exbD2*	Trafficking and Secretion	Trafficing
RCAP_rcc03199	0.31	0.0001	2687	3340	0.04%	0.05%	2545	2964	3145	3535	*tolQ*	Trafficking and Secretion	Secretion
RCAP_rcc03200	0.27	0.06	247	300	0.00%	0.00%	214	285	270	329	*ybgC*	Unknown	Unknown
RCAP_rcc03201	0.4	0.0002	343	454	0.00%	0.01%	312	376	412	497	*hypothetical protein*	Unknown	Unknown
RCAP_rcc03202	-0.33	0.0643	748	593	0.01%	0.01%	602	869	518	668	*glpK2*	Lipid Metabolism	Glycerolipid metabolism
RCAP_rcc03203	-0.18	0.2315	615	543	0.01%	0.01%	520	695	489	597	*glpD*	Lipid Metabolism	Glycerophospholipid metabolism
RCAP_rcc03204	-0.32	0.0401	4827	3851	0.07%	0.05%	4258	5358	3324	4379	*ileS*	Translation, ribosomal structure and biogenesis	Aminoacyl-tRNA biosynthesis
RCAP_rcc03205	0.01	0.9664	394	395	0.01%	0.01%	353	427	370	421	*hypothetical protein*	Unknown	Unknown
RCAP_rcc03206	0.07	0.6305	2260	2375	0.03%	0.03%	1984	2523	2142	2609	*pcs*	Lipid Metabolism	Glycerophospholipid metabolism
RCAP_rcc03207	-1.54	0	1987	661	0.03%	0.01%	1533	2300	594	728	*hypothetical protein*	Unknown	Unknown
RCAP_rcc03208	0.04	0.7819	1206	1239	0.02%	0.02%	1106	1303	1100	1378	*xerC*	Replication, Recombination and Repair	Recombination
RCAP_rcc03209	0.47	0	2571	3581	0.04%	0.05%	2459	2900	3307	3855	*hypothetical protein*	Unknown	Unknown
RCAP_rcc03210	-0.35	0.0297	5720	4449	0.08%	0.06%	4880	6533	3849	5049	*talC*	Carbohydrate Metabolism	Pentose phosphate pathway
RCAP_rcc03211	0.66	0	255	405	0.00%	0.01%	232	289	343	467	*priA*	Replication, Recombination and Repair	Unknown
RCAP_rcc03212	0.59	0.0007	813	1240	0.01%	0.02%	736	975	1030	1449	*prmA*	Translation, ribosomal structure and biogenesis	Unknown
RCAP_rcc03213	1.13	0.0002	478	1136	0.01%	0.02%	331	681	830	1442	*hypothetical protein*	Unknown	Unknown
RCAP_rcc03214	3.39	0	262	3905	0.01%	0.05%	276	497	2645	5165	*hypothetical protein*	Unknown	Unknown
RCAP_rcc03215	-1.18	0	20	9	0.00%	0.00%	15	25	7	10	*hypothetical protein*	Unknown	Unknown
RCAP_rcc03216	1.18	0	172	394	0.00%	0.01%	169	201	343	444	*ruvC*	Replication, Recombination and Repair	Unknown
RCAP_rcc03217	0.89	0	103	192	0.00%	0.00%	90	120	161	223	*ruvA*	Replication, Recombination and Repair	Unknown
RCAP_rcc03218	0.08	0.4397	299	314	0.00%	0.00%	277	312	288	340	*ruvB*	Replication, Recombination and Repair	Unknown
RCAP_rcc03219	0.13	0.3485	511	561	0.01%	0.01%	437	585	517	605	*globin family protein*	Unknown	Unknown
RCAP_rcc03220	1.17	0	424	971	0.01%	0.01%	406	497	826	1116	*hypothetical protein*	Unknown	Unknown
RCAP_rcc03221	2.02	0	530	2231	0.01%	0.03%	564	721	1898	2564	*hypothetical protein*	Unknown	Unknown
RCAP_rcc03222	-0.16	0.1199	244	217	0.00%	0.00%	219	259	200	234	*truA*	Nucleotide Metabolism	Pyrimidine metabolism
RCAP_rcc03223	0	0.9901	3395	3398	0.05%	0.05%	3149	3620	3296	3501	*capD*	Cell Envelope Biosynthesis	Cell Wall Biosynthesis
RCAP_rcc03224	-0.02	0.8967	172	169	0.00%	0.00%	159	187	158	180	*sugar transferase*	Cell Envelope Biosynthesis	Cell Wall Biosynthesis
RCAP_rcc03225	0.3	0.5347	31	40	0.00%	0.00%	10	49	16	64	*NAD-dependent epimerase/dehydratase*	Unknown	Unknown
RCAP_rcc03226	0.01	0.9135	1812	1825	0.03%	0.02%	1706	1901	1746	1903	*polysaccharide biosynthesis/export family protein*	Cell Envelope Biosynthesis	Cell Wall Biosynthesis
RCAP_rcc03227	0.33	0.0019	2390	3005	0.03%	0.04%	2204	2714	2815	3195	*ilvE2*	Metabolism of Cofactors, Coenzymes and Vitamins	Pantothenate and CoA biosynthesis
RCAP_rcc03228	-0.01	0.974	1980	1973	0.03%	0.03%	1733	2242	1746	2200	*universal stress family protein*	Stress Response	Unknown
RCAP_rcc03229	0.66	0	1810	2872	0.03%	0.04%	1796	2062	2545	3199	*NifU domain-containing protein*	Energy Metabolism	Nitrogen metabolism
RCAP_rcc03230	1.05	0.0111	47	116	0.00%	0.00%	29	67	38	193	*M22 family peptidase*	Post-translational Modification, Assembly and Chaperones	Peptidase
RCAP_rcc03231	0.23	NA	45	52	0.00%	0.00%	39	51	30	74	*rimI*	Unknown	Unknown
RCAP_rcc03232	0.61	0.0004	5029	7776	0.07%	0.11%	4364	6053	6677	8874	*basic membrane lipoprotein family*	Predicted Function	Unknown
RCAP_rcc03233	-0.15	0.2713	2555	2293	0.04%	0.03%	2225	2816	2057	2529	*monosacharide ABC transporter ATP-binding protein*	Carbohydrate Metabolism	Aerobic/Anaerobic Respiration
RCAP_rcc03234	-0.12	0.2405	1434	1317	0.02%	0.02%	1313	1542	1211	1423	*monosacharide ABC transporter permease*	Carbohydrate Metabolism	Aerobic/Anaerobic Respiration
RCAP_rcc03235	-0.03	0.8926	890	872	0.01%	0.01%	737	1033	780	964	*monosacharide ABC transporter permease*	Carbohydrate Metabolism	Aerobic/Anaerobic Respiration
RCAP_rcc03236	-0.26	0.002	786	656	0.01%	0.01%	730	829	605	707	*punA*	Metabolism of Cofactors, Coenzymes and Vitamins	Nicotinate and nicotinamide metabolism
RCAP_rcc03237	-0.36	0	973	758	0.01%	0.01%	898	1031	722	794	*hypothetical protein*	Unknown	Unknown
RCAP_rcc03238	-0.23	0	807	688	0.01%	0.01%	772	839	657	720	*hypothetical protein*	Unknown	Unknown
RCAP_rcc03239	-0.02	0.9268	651	642	0.01%	0.01%	536	769	565	720	*AsnC/Lrp family transcriptional regulator*	Signal Transduction	Transcription Regulator
RCAP_rcc03240	0.03	0.7604	716	732	0.01%	0.01%	656	756	672	792	*ATPase AAA*	Replication, Recombination and Repair	Unknown
RCAP_rcc03241	0.29	0.231	272	335	0.00%	0.00%	227	334	242	429	*mutB*	Carbohydrate Metabolism	Glyoxylate and dicarboxylate metabolism
RCAP_rcc03242	-0.64	0.0031	4063	2554	0.06%	0.03%	3176	4849	2068	3040	*hypothetical protein*	Unknown	Unknown
RCAP_rcc03243	-0.41	0.0268	2055	1538	0.03%	0.02%	1682	2394	1288	1787	*ccrA*	Carbohydrate Metabolism	Glyoxylate and dicarboxylate metabolism
RCAP_rcc03244	-1.02	0.008	4	1	0.00%	0.00%	2	5	1	2	*hypothetical protein*	Unknown	Unknown
RCAP_rcc03245	1.57	0	430	1380	0.01%	0.02%	335	565	1025	1736	*ROK family protein*	Transcription	Unknown
RCAP_rcc03246	1.73	0	61	236	0.00%	0.00%	45	84	147	326	*family 2 glycosyl transferase*	Unknown	Unknown
RCAP_rcc03247	2.03	0	279	1214	0.00%	0.02%	292	401	1010	1418	*ceramide glucosyltransferase*	Cell Envelope Biosynthesis	Cell Wall Biosynthesis
RCAP_rcc03248	1.66	0	94	313	0.00%	0.00%	97	128	242	385	*metallophosphoesterase*	Metabolism of Cofactors, Coenzymes and Vitamins	Folate biosynthesis
RCAP_rcc03249	0.05	0.8863	295	305	0.00%	0.00%	198	389	269	341	*BioY family protein*	Metabolism of Cofactors, Coenzymes and Vitamins	Biotin metabolism
RCAP_rcc03250	0.28	0.0633	145	175	0.00%	0.00%	125	167	158	192	*cbiQ3*	Metabolism of Cofactors, Coenzymes and Vitamins	Cobalamin Biosynthesis
RCAP_rcc03251	0.63	0.0012	148	233	0.00%	0.00%	115	180	208	258	*cbiO3*	Metabolism of Cofactors, Coenzymes and Vitamins	Cobalamin Biosynthesis
RCAP_rcc03252	0.12	0.2935	1462	1590	0.02%	0.02%	1329	1589	1439	1740	*cydD*	Energy Metabolism	Aerobic/Anaerobic Respiration
RCAP_rcc03253	0.12	0.766	837	924	0.01%	0.01%	509	1131	648	1201	*cydC*	Energy Metabolism	Aerobic/Anaerobic Respiration
RCAP_rcc03254	0.13	0.3468	454	497	0.01%	0.01%	395	507	449	545	*cytochrome P450 family protein*	Energy Metabolism	Aerobic/Anaerobic Respiration
RCAP_rcc03255	1.36	0	239	621	0.00%	0.01%	239	278	548	694	*Crp/Fnr family transcriptional regulator*	Signal Transduction	Transcription Regulator
RCAP_rcc03256	0.28	0.1905	636	780	0.01%	0.01%	545	713	575	985	*cytochrome P450 family protein*	Energy Metabolism	Aerobic/Anaerobic Respiration
RCAP_rcc03257	-1.92	0	788	195	0.01%	0.00%	523	995	175	215	*msrA1*	Post-translational Modification, Assembly and Chaperones	Unknown
RCAP_rcc03258	-1.69	0	2598	736	0.04%	0.01%	1658	3353	565	908	*cytochrome c biogenesis protein transmembrane region*	Energy Metabolism	Aerobic/Anaerobic Respiration
RCAP_rcc03259	-1.12	0	2523	1107	0.04%	0.02%	1736	3255	907	1308	*msrB1*	Post-translational Modification, Assembly and Chaperones	Unknown
RCAP_rcc03260	0.9	0	794	1492	0.01%	0.02%	756	899	1406	1577	*NAD-dependent epimerase/dehydratase*	Metabolism of Cofactors, Coenzymes and Vitamins	Porphyrin and chlorophyll metabolism
RCAP_rcc03261	1.52	0	2069	6053	0.03%	0.08%	2040	2663	5400	6706	*TOBE domain-containing protein*	Metabolism of Cofactors, Coenzymes and Vitamins	Unknown
RCAP_rcc03262	0.35	0.0587	51	65	0.00%	0.00%	42	61	56	74	*hypothetical protein*	Unknown	Unknown
RCAP_rcc03263	0.87	0.0007	4	8	0.00%	0.00%	3	6	7	9	*NifT/FixU family protein*	Energy Metabolism	Nitrogen metabolism
RCAP_rcc03264	0.72	0.0008	12	20	0.00%	0.00%	10	15	17	24	*NifZ family protein*	Energy Metabolism	Nitrogen metabolism
RCAP_rcc03265	0.77	0.0045	5	8	0.00%	0.00%	4	6	6	11	*LRV FeS4 cluster domain-containing protein*	Unknown	Unknown
RCAP_rcc03266	0.14	0.2646	104	115	0.00%	0.00%	91	117	107	122	*nifB2*	Energy Metabolism	Nitrogen metabolism
RCAP_rcc03267	1.51	0	160	470	0.00%	0.01%	159	213	390	549	*nifA2*	Signal Transduction	Transcription Regulator
RCAP_rcc03268	0.16	0.5286	10	11	0.00%	0.00%	8	12	9	13	*nifW*	Energy Metabolism	Nitrogen metabolism
RCAP_rcc03269	1.28	0.0001	4	9	0.00%	0.00%	3	5	7	12	*nifV*	Energy Metabolism	Nitrogen metabolism
RCAP_rcc03270	1.02	0	6	13	0.00%	0.00%	5	8	10	15	*nifS*	Metabolism of Cofactors, Coenzymes and Vitamins	Nitrogen metabolism
RCAP_rcc03271	1.21	0.0023	1	3	0.00%	0.00%	1	2	2	4	*nifU2*	Energy Metabolism	Nitrogen metabolism
RCAP_rcc03272	1.16	0.0012	2	5	0.00%	0.00%	1	3	4	7	*HesB/YadR/YfhF family protein*	Unknown	Unknown
RCAP_rcc03273	0.89	0.0187	2	4	0.00%	0.00%	1	3	3	5	*hypothetical protein*	Unknown	Unknown

RCAP_rcc03274	0.83	0.0165	3	5	0.00%	0.00%	2	3	3	6	*NifQ family protein*	Energy Metabolism	Nitrogen metabolism
RCAP_rcc03275	0.29	0.3114	11	14	0.00%	0.00%	8	15	12	17	*fdxB*	Energy Metabolism	Aerobic/Anaerobic Respiration
RCAP_rcc03276	0.48	0.0329	13	18	0.00%	0.00%	10	16	16	21	*hypothetical protein*	Unknown	Unknown
RCAP_rcc03277	0.53	0.0115	16	23	0.00%	0.00%	12	19	19	26	*hypothetical protein*	Unknown	Unknown
RCAP_rcc03278	0.4	0.1032	6	8	0.00%	0.00%	5	7	6	9	*nifX*	Energy Metabolism	Nitrogen metabolism
RCAP_rcc03279	0.8	0.0003	9	15	0.00%	0.00%	7	11	13	18	*nifN*	Energy Metabolism	Nitrogen metabolism
RCAP_rcc03280	0.42	0.0927	52	71	0.00%	0.00%	39	67	61	81	*nifE*	Energy Metabolism	Nitrogen metabolism
RCAP_rcc03281	-0.62	0.0055	3249	2072	0.05%	0.03%	2679	3850	1621	2523	*peroxiredoxin*	Post-translational Modification, Assembly and Chaperones	Unknown
RCAP_rcc03282	0.99	0	15	31	0.00%	0.00%	13	18	25	37	*rnfF*	Energy Metabolism	Nitrogen metabolism
RCAP_rcc03283	1.4	0.0018	2	6	0.00%	0.00%	1	3	3	9	*rseC*	Signal Transduction	Transcription Regulator
RCAP_rcc03284	1.61	0	13	42	0.00%	0.00%	12	19	34	51	*fdxN*	Energy Metabolism	Aerobic/Anaerobic Respiration
RCAP_rcc03285	1.22	0	7	16	0.00%	0.00%	6	9	13	19	*fdxC*	Energy Metabolism	Aerobic/Anaerobic Respiration
RCAP_rcc03286	0.8	0.0001	11	19	0.00%	0.00%	10	13	16	23	*norV*	Unknown	Unknown
RCAP_rcc03287	0.92	0.0018	5	9	0.00%	0.00%	3	7	8	10	*rnfA*	Energy Metabolism	Unknown
RCAP_rcc03288	0.47	0.1612	3	4	0.00%	0.00%	2	4	3	5	*rnfB*	Energy Metabolism	Unknown
RCAP_rcc03289	0.75	0.0001	12	20	0.00%	0.00%	10	15	17	23	*rnfC*	Energy Metabolism	Unknown
RCAP_rcc03290	0.32	0.1041	16	20	0.00%	0.00%	13	19	17	22	*rnfD*	Energy Metabolism	Unknown
RCAP_rcc03291	0.7	0.001	7	12	0.00%	0.00%	6	9	10	13	*rnfG*	Energy Metabolism	Unknown
RCAP_rcc03292	0.35	0.1079	12	16	0.00%	0.00%	10	15	14	18	*rnfE*	Energy Metabolism	Unknown
RCAP_rcc03293	-0.3	0.4567	7	6	0.00%	0.00%	4	10	4	7	*hypothetical protein*	Unknown	Unknown
RCAP_rcc03294	0.42	0.187	5	6	0.00%	0.00%	3	6	5	8	*fccB*	Energy Metabolism	Unknown
RCAP_rcc03295	-0.67	0	567	355	0.01%	0.00%	501	628	310	400	*hypothetical protein*	Unknown	Unknown
RCAP_rcc03296	0.63	0.0056	477	756	0.01%	0.01%	440	559	552	959	*betC*	Metal and Ion Transport	Unknown
RCAP_rcc03297	1.34	0	243	658	0.00%	0.01%	230	316	457	859	*glycine betaine/L-proline ABC transporter periplasmic glycine betaine/*	Amino Acid Metabolism	Amino Acid Transport
RCAP_rcc03298	0.67	0.0017	104	169	0.00%	0.00%	87	125	133	205	*LysR family transcriptional regulator*	Signal Transduction	Transcription Regulator
RCAP_rcc03299	0.3	0.4022	111	139	0.00%	0.00%	68	147	86	193	*hypothetical protein*	Unknown	Unknown
RCAP_rcc03300	-0.12	0.4032	419	383	0.01%	0.01%	368	448	332	434	*radical SAM family protein*	Unknown	Unknown
RCAP_rcc03301	-2.09	0	2638	611	0.04%	0.01%	2282	2779	558	663	*diguanylate cyclase/phosphodiesterase*	Signal Transduction	Kinase/Phosphorelay
RCAP_rcc03302	-0.35	0.0322	127	99	0.00%	0.00%	105	147	91	108	*GNAT family acetyltransferase*	Cell Division	Chromosome Partitioning
RCAP_rcc03303	0.11	0.3562	1006	1086	0.01%	0.01%	904	1112	1005	1166	*dapE*	Amino Acid Metabolism	Lysine biosynthesis
RCAP_rcc03304	0.3	0.0951	738	912	0.01%	0.01%	653	836	740	1084	*hypothetical protein*	Unknown	Unknown
RCAP_rcc03305	0.17	0.5381	727	822	0.01%	0.01%	565	873	598	1046	*hypothetical protein*	Unknown	Unknown
RCAP_rcc03306	-0.81	0	2117	1192	0.03%	0.02%	1703	2456	1060	1324	*acyl-CoA dehydrogenase, medium-chain specific*	Carbohydrate Metabolism	Glyoxylate and dicarboxylate metabolism
RCAP_rcc03307	-0.42	0.0016	110	81	0.00%	0.00%	93	124	75	87	*GNAT family acetyltransferase*	Cell Division	Chromosome Partitioning
RCAP_rcc03308	-0.26	0.0585	156	129	0.00%	0.00%	142	163	110	148	*recO*	Replication, Recombination and Repair	Recombination
RCAP_rcc03309	-0.76	0.0015	639	366	0.01%	0.00%	497	752	273	459	*hypothetical protein*	Unknown	Unknown
RCAP_rcc03310	-0.46	0.0004	2140	1548	0.03%	0.02%	1937	2291	1318	1779	*era*	Unknown	Unknown
RCAP_rcc03311	-0.28	0.0497	534	438	0.01%	0.01%	449	596	395	481	*rnc*	Transcription	Unknown
RCAP_rcc03312	-0.44	0.0149	3069	2236	0.04%	0.03%	2483	3543	1877	2596	*lepB*	Trafficking and Secretion	Trafficing
RCAP_rcc03313	-0.22	0.0918	332	284	0.00%	0.00%	287	370	257	311	*acpS*	Metabolism of Cofactors, Coenzymes and Vitamins	Pantothenate and CoA biosynthesis
RCAP_rcc03314	-0.03	0.8153	467	459	0.01%	0.01%	426	503	426	492	*hypothetical protein*	Unknown	Unknown
RCAP_rcc03315	0.11	0.5096	1165	1263	0.02%	0.02%	963	1368	1117	1409	*pdxJ*	Metabolism of Cofactors, Coenzymes and Vitamins	Vitamin B6 metabolism
RCAP_rcc03316	-0.63	0.0001	821	525	0.01%	0.01%	711	905	445	604	*hypothetical protein*	Unknown	Unknown
RCAP_rcc03317	-0.38	0.0357	6059	4608	0.08%	0.06%	4955	7018	3898	5318	*spoT*	Nucleotide Metabolism	Purine metabolism
RCAP_rcc03318	0.05	0.7783	3312	3422	0.05%	0.05%	3196	3630	2908	3936	*rpoZ*	Replication, Recombination and Repair	Replication
RCAP_rcc03319	-0.19	0.1949	260	227	0.00%	0.00%	215	298	206	248	*folK*	Metabolism of Cofactors, Coenzymes and Vitamins	Folate biosynthesis
RCAP_rcc03320	0	0.9849	2824	2816	0.04%	0.04%	2270	3390	2473	3159	*hypothetical protein*	Unknown	Unknown
RCAP_rcc03321	-0.02	0.8758	461	454	0.01%	0.01%	417	496	407	500	*rluA*	Nucleotide Metabolism	Pyrimidine metabolism
RCAP_rcc03322	0.15	0.5875	219	244	0.00%	0.00%	167	274	208	280	*GAF domain-containing protein*	Signal Transduction	Kinase/Phosphorelay
RCAP_rcc03323	-0.53	0.0001	765	526	0.01%	0.01%	650	860	479	573	*rsbV*	Unknown	Unknown
RCAP_rcc03324	-0.07	0.7236	820	783	0.01%	0.01%	678	972	744	823	*rsbW*	Signal Transduction	Kinase/Phosphorelay
RCAP_rcc03325	0.35	0.0644	1593	2049	0.02%	0.03%	1304	1952	1763	2335	*hemimethylated DNA-binding protein family*	Stress Response	Unknown
RCAP_rcc03326	0.74	0	1374	2328	0.02%	0.03%	1220	1723	2012	2644	*lipoprotein*	Predicted Function	Unknown
RCAP_rcc03327	0.56	0	1696	2514	0.03%	0.03%	1641	1979	2235	2793	*lolA*	Cell Envelope Biosynthesis	Cell Wall Biosynthesis
RCAP_rcc03328	-0.35	0.0548	10736	8373	0.15%	0.11%	8751	12623	7341	9406	*ftsK*	Cell Division	Chromosome Partitioning
RCAP_rcc03329	0.08	0.6099	2058	2170	0.03%	0.03%	1795	2354	1997	2343	*class I/II aminotransferase*	Amino Acid Metabolism	Lysine biosynthesis
RCAP_rcc03330	0.49	0.0024	344	488	0.00%	0.01%	303	389	407	569	*amidase*	Translation, ribosomal structure and biogenesis	Unknown
RCAP_rcc03331	0.1	0.538	1284	1376	0.02%	0.02%	1120	1452	1175	1577	*ubiF*	Photosynthesis	Biosynthesis of Ubiquinone
RCAP_rcc03332	0.02	0.9527	109	111	0.00%	0.00%	71	145	87	135	*hypothetical protein*	Unknown	Unknown
RCAP_rcc03333	0.1	0.5034	1723	1850	0.02%	0.03%	1498	2012	1688	2012	*S16 family peptidase*	Post-translational Modification, Assembly and Chaperones	Peptidase
RCAP_rcc03334	1.21	0	2018	4757	0.03%	0.06%	1958	2567	4156	5358	*trxA2*	Post-translational Modification, Assembly and Chaperones	Unknown
RCAP_rcc03335	-0.18	0.1656	1657	1463	0.02%	0.02%	1459	1827	1315	1611	*xthA2*	Replication, Recombination and Repair	Unknown
RCAP_rcc03336	0.38	0.0247	113	147	0.00%	0.00%	94	131	128	165	*XRE family transcriptional regulator*	Signal Transduction	Transcription Regulator
RCAP_rcc03337	-0.53	0.041	63	42	0.00%	0.00%	41	79	35	49	*aceB*	Carbohydrate Metabolism	Glyoxylate and dicarboxylate metabolism
RCAP_rcc03338	-0.18	0.1969	208	183	0.00%	0.00%	176	242	171	194	*aceA*	Carbohydrate Metabolism	Glyoxylate and dicarboxylate metabolism
RCAP_rcc03339	0.26	0.6393	33	43	0.00%	0.00%	7	58	14	72	*hypothetical protein*	Unknown	Unknown
RCAP_rcc03340	0.64	0.1203	50	84	0.00%	0.00%	23	74	42	126	*hypothetical protein*	Unknown	Unknown
RCAP_rcc03341	1.37	0	174	478	0.00%	0.01%	131	221	370	586	*hypothetical protein*	Unknown	Unknown
RCAP_rcc03342	-1.46	0	3729	1278	0.05%	0.02%	2955	4197	933	1623	*nrdD*	Nucleotide Metabolism	Pyrimidine metabolism
RCAP_rcc03343	-1.57	0	1097	348	0.01%	0.00%	844	1264	268	428	*hypothetical protein*	Unknown	Unknown
RCAP_rcc03344	-1.16	0.0018	409	158	0.01%	0.00%	196	586	89	227	*anaerobic ribonucleoside-triphosphate reductase activating protein*	Post-translational Modification, Assembly and Chaperones	Unknown
RCAP_rcc03345	-0.74	0	196	116	0.00%	0.00%	157	224	100	131	*XRE family transcriptional regulator*	Signal Transduction	Transcription Regulator
RCAP_rcc03346	1.49	0.0008	1	5	0.00%	0.00%	1	2	2	8	*thioesterase superfamily protein*	Lipid Metabolism	Unknown
RCAP_rcc03347	0.68	0.0004	17	27	0.00%	0.00%	15	21	23	32	*prpD*	Carbohydrate Metabolism	Propanoate metabolism
RCAP_rcc03348	0.01	0.9475	29	29	0.00%	0.00%	25	32	27	32	*prpC*	Carbohydrate Metabolism	Propanoate metabolism
RCAP_rcc03349	-1.72	0	187	54	0.00%	0.00%	137	223	46	62	*prpB*	Carbohydrate Metabolism	Propanoate metabolism
RCAP_rcc03350	-2.77	0	1720	214	0.02%	0.00%	1186	2096	168	260	*ferredoxin domain-containing protein oxidireductase*	Energy Metabolism	Aerobic/Anaerobic Respiration

RCAP_rcc03351	-2.6	0	3717	543	0.05%	0.01%	2769	4334	434	652	*rieske (2Fe-2S) domain-containing protein*	Xenobiotics Biodegradation and Metabolism	Fluorobenzoate degradation
RCAP_rcc03352	-2.77	NA	2831	299	0.04%	0.00%	-354	5765	264	335	*metH2*	Metabolism of Cofactors, Coenzymes and Vitamins	One carbon pool by folate
RCAP_rcc03353	-1.73	0	568	156	0.01%	0.00%	309	790	127	185	*metH1*	Metabolism of Cofactors, Coenzymes and Vitamins	One carbon pool by folate
RCAP_rcc03354	-2.1	0	799	175	0.01%	0.00%	622	904	138	211	*cobQ2*	Metabolism of Cofactors, Coenzymes and Vitamins	Cobalamin Biosynthesis
RCAP_rcc03355	-1.98	0	634	144	0.01%	0.00%	399	811	113	175	*cobQ3*	Metabolism of Cofactors, Coenzymes and Vitamins	Cobalamin Biosynthesis
RCAP_rcc03356	-0.57	0.1949	60	36	0.00%	0.00%	29	85	21	51	*hypothetical protein*	Unknown	Unknown
RCAP_rcc03357	-0.11	0.8296	48	43	0.00%	0.00%	20	72	17	70	*hypothetical protein*	Unknown	Unknown
RCAP_rcc03358	-0.36	0.0436	428	330	0.01%	0.00%	360	484	262	399	*TonB-dependent receptor*	Metal and Ion Transport	Unknown
RCAP_rcc03359	-0.17	0.5686	40	35	0.00%	0.00%	31	49	24	46	*iron siderophore/cobalamin ABC transporter periplasmic iron sideroph*	Metal, Ion, Cofactor Transport	Iron and Heme Transport
RCAP_rcc03360	-0.41	0.2134	58	42	0.00%	0.00%	36	76	29	55	*iron siderophore/cobalamin ABC transporter permease*	Metal, Ion, Cofactor Transport	Iron and Heme Transport
RCAP_rcc03361	-0.15	0.7538	21	18	0.00%	0.00%	10	30	12	25	*iron siderophore/cobalamin ABC transporter ATP-binding protein*	Metal, Ion, Cofactor Transport	Iron and Heme Transport
RCAP_rcc03362	-0.3	0.5682	24	18	0.00%	0.00%	10	36	9	28	*cbiZ*	Metabolism of Cofactors, Coenzymes and Vitamins	Cobalamin Biosynthesis
RCAP_rcc03363	0.37	0.2745	175	232	0.00%	0.00%	128	220	116	347	*bioB*	Metabolism of Cofactors, Coenzymes and Vitamins	Biotin metabolism
RCAP_rcc03364	0.72	0.0378	34	59	0.00%	0.00%	23	43	29	88	*bioF*	Metabolism of Cofactors, Coenzymes and Vitamins	Biotin metabolism
RCAP_rcc03365	0.84	0.0437	9	19	0.00%	0.00%	5	13	9	28	*bioD*	Metabolism of Cofactors, Coenzymes and Vitamins	Biotin metabolism
RCAP_rcc03366	0.64	0.0055	46	74	0.00%	0.00%	38	54	54	94	*bioA*	Metabolism of Cofactors, Coenzymes and Vitamins	Biotin metabolism
RCAP_rcc03367	0.62	0.0503	6	9	0.00%	0.00%	4	7	6	13	*hypothetical protein*	Unknown	Unknown
RCAP_rcc03368	0.51	0.2094	7	11	0.00%	0.00%	4	10	6	16	*type 11 family methyltransferase*	Metabolism of Cofactors, Coenzymes and Vitamins	Unknown
RCAP_rcc03369	0.52	0.0139	41	59	0.00%	0.00%	33	50	50	69	*GntR family transcriptional regulator*	Signal Transduction	Transcription Regulator
RCAP_rcc03370	0.36	0.279	6	8	0.00%	0.00%	4	8	5	10	*LysR family transcriptional regulator*	Signal Transduction	Transcription Regulator
RCAP_rcc03371	-0.3	0.003	286	231	0.00%	0.00%	252	311	216	247	*fabI*	Lipid Metabolism	Fatty acid biosynthesis
RCAP_rcc03372	-0.56	0	956	644	0.01%	0.01%	817	1063	606	682	*hypothetical protein*	Unknown	Unknown
RCAP_rcc03373	-0.36	0.1423	182	140	0.00%	0.00%	136	225	119	161	*ackA3*	Energy Metabolism	Reductive carboxylate cycle (CO2 fixation)
RCAP_rcc03374	-0.6	0	179	118	0.00%	0.00%	159	190	106	130	*hypothetical protein*	Unknown	Unknown
RCAP_rcc03375	-0.38	0.0014	65	49	0.00%	0.00%	60	70	43	55	*phnX*	Metabolism of Other Amino Acids	Phosphonate and phosphinate metabolism
RCAP_rcc03376	0.79	0.0036	102	184	0.00%	0.00%	72	134	142	225	*hypothetical protein*	Unknown	Unknown
RCAP_rcc03377	1.05	0	872	1872	0.01%	0.03%	726	1114	1532	2211	*hypothetical protein*	Unknown	Unknown
RCAP_rcc03378	-0.08	0.7226	554	523	0.01%	0.01%	437	658	457	588	*CDA peptide synthetase III*	Lipid Metabolism	Fatty acid metabolism
RCAP_rcc03379	-1.38	0	13002	4852	0.18%	0.07%	10998	14275	3936	5769	*hypothetical protein*	Unknown	Unknown
RCAP_rcc03380	0.46	0.0031	2425	3364	0.03%	0.05%	2120	2855	2963	3765	*smpB*	Post-translational Modification, Assembly and Chaperones	Unknown
RCAP_rcc03381	0.23	0.0588	2925	3429	0.04%	0.05%	2566	3337	3241	3617	*sseA*	Metal and Ion Transport	Unknown
RCAP_rcc03382	-0.06	0.5788	5797	5578	0.08%	0.08%	5418	6240	5194	5962	*tyrB*	Amino Acid Metabolism	Phenylalanine, tyrosine and tryptophan biosynthesis
RCAP_rcc03383	1.24	0.0002	128	340	0.00%	0.00%	91	178	181	498	*MarR family transcriptional regulator*	Signal Transduction	Transcription Regulator
RCAP_rcc03384	0.43	0.1769	303	421	0.00%	0.01%	205	418	296	547	*emrA*	Defense Mechanisms	Unknown
RCAP_rcc03385	0.03	0.9137	566	578	0.01%	0.01%	450	691	473	682	*emrB*	Metal and Ion Transport	Unknown
RCAP_rcc03386	1.37	0	340	892	0.01%	0.01%	341	396	734	1050	*amtB*	Metal and Ion Transport	Unknown
RCAP_rcc03387	2.05	0	175	799	0.00%	0.01%	164	248	521	1077	*glnB2*	Signal Transduction	Transcription Regulator
RCAP_rcc03388	0.31	0.0004	1519	1886	0.02%	0.03%	1394	1674	1796	1976	*1A family penicillin-binding protein*	Glycan Biosynthesis and Metabolism	Peptidoglycan biosynthesis
RCAP_rcc03389	0.37	0.0001	1576	2047	0.02%	0.03%	1461	1765	1925	2170	*toluene tolerance family protein*	Secondary metabolites biosynthesis, transport, and catabolism	Unknown
RCAP_rcc03390	0.78	0	1926	3319	0.03%	0.05%	1876	2206	2985	3654	*VacJ family lipoprotein*	Cell Envelope Biosynthesis	Cell Wall Biosynthesis
RCAP_rcc03391	-0.11	0.1085	1108	1020	0.02%	0.01%	1037	1147	960	1081	*type I secretion system ATPase*	Trafficking and Secretion	Secretion
RCAP_rcc03392	0.02	0.9527	567	573	0.01%	0.01%	449	679	507	640	*hemolysin D*	Trafficking and Secretion	Secretion
RCAP_rcc03393	0.47	0.0016	509	712	0.01%	0.01%	445	590	630	794	*lytic murein transglycosylase*	Glycan Biosynthesis and Metabolism	Glycosaminoglycan degradation
RCAP_rcc03394	0.51	0.0175	714	1036	0.01%	0.01%	579	890	881	1192	*hypothetical protein*	Unknown	Unknown
RCAP_rcc03395	-0.02	0.9064	689	678	0.01%	0.01%	591	781	621	735	*fmt*	Translation, ribosomal structure and biogenesis	Aminoacyl-tRNA biosynthesis
RCAP_rcc03396	0	0.9903	747	747	0.01%	0.01%	684	803	686	807	*def1*	Translation, ribosomal structure and biogenesis	Unknown
RCAP_rcc03397	0.07	0.7729	2220	2338	0.03%	0.03%	1822	2686	1933	2742	*def2*	Translation, ribosomal structure and biogenesis	Unknown
RCAP_rcc03398	0.48	0.0002	539	757	0.01%	0.01%	469	629	694	820	*malY*	Amino Acid Metabolism	Unknown
RCAP_rcc03399	0.83	0	282	510	0.00%	0.01%	258	352	441	579	*hypothetical protein*	Unknown	Unknown
RCAP_rcc03400	1.11	0	37	82	0.00%	0.00%	34	46	68	95	*hypothetical protein*	Unknown	Unknown
RCAP_rcc03401	1.67	0	1357	4457	0.02%	0.06%	1430	1732	3862	5053	*band 7 protein family*	Post-translational Modification, Assembly and Chaperones	Unknown
RCAP_rcc03402	0.9	0	395	748	0.01%	0.01%	371	461	644	852	*hypothetical protein*	Unknown	Unknown
RCAP_rcc03403	0.32	0.0905	1338	1684	0.02%	0.02%	1106	1639	1441	1928	*hypothetical protein*	Unknown	Unknown
RCAP_rcc03404	0.17	0.4324	1217	1372	0.02%	0.02%	982	1509	1195	1548	*pyrF*	Nucleotide Metabolism	Pyrimidine metabolism
RCAP_rcc03405	1.36	0	69	194	0.00%	0.00%	57	104	134	255	*hypothetical protein*	Unknown	Unknown
RCAP_rcc03406	1.44	0	4913	13792	0.08%	0.19%	4704	6574	11812	15771	*clpB*	Post-translational Modification, Assembly and Chaperones	Unknown
RCAP_rcc03407	0.73	0.0001	162	272	0.00%	0.00%	147	188	203	342	*dinB*	Replication, Recombination and Repair	Replication
RCAP_rcc03408	-0.09	0.5113	703	659	0.01%	0.01%	614	771	592	726	*hutG*	Carbohydrate Metabolism	Glyoxylate and dicarboxylate metabolism
RCAP_rcc03409	-1.14	0	7087	2992	0.10%	0.04%	4791	8976	2217	3766	*rpmJ*	Translation, ribosomal structure and biogenesis	Unknown
RCAP_rcc03410	-0.31	0.0062	293	234	0.00%	0.00%	263	316	210	258	*HAD superfamily hydrolase*	Unknown	Unknown
RCAP_rcc03411	0.12	0.6665	170	186	0.00%	0.00%	129	211	159	213	*methyltransferase*	Replication, Recombination and Repair	Unknown
RCAP_rcc03412	-0.18	0.0385	501	441	0.01%	0.01%	456	534	415	467	*thcD*	Lipid Metabolism	Fatty acid metabolism
RCAP_rcc03413	-0.41	0.1146	2390	1770	0.03%	0.02%	1685	3138	1534	2006	*peroxiredoxin*	Post-translational Modification, Assembly and Chaperones	Unknown
RCAP_rcc03414	-0.36	0.0029	929	722	0.01%	0.01%	802	1035	663	781	*hypothetical protein*	Unknown	Unknown
RCAP_rcc03415	-0.16	0.526	829	737	0.01%	0.01%	606	1013	598	877	*ugpQ*	Lipid Metabolism	Glycerophospholipid metabolism
RCAP_rcc03416	-0.21	0.0977	1630	1405	0.02%	0.02%	1395	1847	1309	1501	*endoribonuclease L-PSP family protein*	Unknown	Unknown
RCAP_rcc03417	0.17	0.3238	580	656	0.01%	0.01%	500	687	573	739	*hypothetical protein*	Unknown	Unknown
RCAP_rcc03418	-0.04	0.7243	917	892	0.01%	0.01%	826	983	830	954	*smc*	Cell Division	Chromosome Partitioning
RCAP_rcc03419	2	0	48	223	0.00%	0.00%	37	74	176	270	*LrgA family protein*	Unknown	Unknown
RCAP_rcc03420	1.79	0	152	545	0.00%	0.01%	144	192	456	633	*LrgB family protein*	Cell Envelope Biosynthesis	Cell Wall Biosynthesis
RCAP_rcc03421	-0.18	0.1796	729	641	0.01%	0.01%	646	797	569	713	*rnhA2*	Replication, Recombination and Repair	Unknown
RCAP_rcc03422	0.39	0	1104	1446	0.02%	0.02%	1037	1198	1339	1552	*LysE family transporter*	Unknown	Unknown
RCAP_rcc03423	1.19	0	2564	5916	0.04%	0.08%	2724	2947	5214	6618	*ispH*	Lipid	Terpenoid backbone biosynthesis
RCAP_rcc03424	-0.35	0.0018	755	590	0.01%	0.01%	670	820	539	641	*winged helix family two component transcriptional regulator*	Signal Transduction	Transcription Regulator
RCAP_rcc03425	0.21	0.1465	734	850	0.01%	0.01%	633	819	752	948	*sensor histidine kinase/response regulator receiver protein*	Signal Transduction	Transcription Regulator
RCAP_rcc03426	-2.16	0	5376	860	0.07%	0.01%	2570	7642	348	1371	*AMP-dependent synthetase and ligase*	Lipid Metabolism	Fatty acid metabolism
RCAP_rcc03427	-1.96	0	3369	593	0.04%	0.01%	1597	4800	222	964	*livG3*	Amino Acid Metabolism	Amino Acid Transport

RCAP_rcc03428	-1.71	0	4572	1053	0.06%	0.01%	2114	6572	442	1665	*livH3*	Amino Acid Metabolism	Amino Acid Transport
RCAP_rcc03429	0.44	0.0695	1923	2651	0.03%	0.04%	1405	2503	2061	3241	*hypothetical protein*	Unknown	Unknown
RCAP_rcc03430	-1.15	0.0001	4339	1811	0.06%	0.02%	2333	6027	1337	2285	*livM3*	Amino Acid Metabolism	Amino Acid Transport
RCAP_rcc03431	-1.74	0	11633	2786	0.16%	0.04%	5600	16526	1425	4147	*livK2*	Amino Acid Metabolism	Amino Acid Transport
RCAP_rcc03432	-1.41	0.0002	2831	882	0.04%	0.01%	1182	4214	463	1301	*livF3*	Amino Acid Metabolism	Amino Acid Transport
RCAP_rcc03433	-0.67	NA	821	480	0.01%	0.01%	336	1246	283	677	*paaK*	Amino Acid Metabolism	Phenylalanine metabolism
RCAP_rcc03434	0.34	0.0027	1309	1665	0.02%	0.02%	1177	1507	1543	1787	*map*	Translation, ribosomal structure and biogenesis	Unknown
RCAP_rcc03435	0.3	0.0001	471	579	0.01%	0.01%	445	517	554	604	*molybdopterin binding domain-containing protein*	Metabolism of Cofactors, Coenzymes and Vitamins	Unknown
RCAP_rcc03436	0.17	0.5447	75	85	0.00%	0.00%	54	93	65	105	*GNAT family acetyltransferase*	Cell Division	Chromosome Partitioning
RCAP_rcc03437	-0.56	0	417	282	0.01%	0.00%	378	436	261	303	*OmpA/MotB domain-containing protein*	Cell Envelope Biosynthesis	Cell Wall Biosynthesis
RCAP_rcc03438	-1.06	0	5111	2429	0.07%	0.03%	4363	5587	2202	2656	*LysR family transcriptional regulator*	Signal Transduction	Transcription Regulator
RCAP_rcc03439	0.15	0.5761	853	948	0.01%	0.01%	674	1076	787	1110	*fadH*	Lipid Metabolism	Fatty acid metabolism
RCAP_rcc03440	0.3	0.0184	480	591	0.01%	0.01%	428	545	550	632	*hypothetical protein*	Unknown	Unknown
RCAP_rcc03441	0.17	0.2638	1692	1913	0.02%	0.03%	1436	1917	1750	2075	*hypothetical protein*	Unknown	Unknown
RCAP_rcc03442	-0.07	0.6115	331	314	0.00%	0.00%	285	368	293	336	*lipoprotein*	Predicted Function	Unknown
RCAP_rcc03443	0.45	0.199	244	346	0.00%	0.00%	152	327	231	461	*BadF/BadG/BcrA/BcrD family ATPase*	Carbohydrate Metabolism	Amino sugar and nucleotide sugar metabolism
RCAP_rcc03444	0.14	0.6415	284	317	0.00%	0.00%	200	359	238	395	*nagA*	Carbohydrate Metabolism	Amino sugar and nucleotide sugar metabolism
RCAP_rcc03445	-0.29	0.1551	536	437	0.01%	0.01%	427	637	375	498	*serB*	Energy Metabolism	Methane metabolism
RCAP_rcc03446	0.31	0.047	2227	2769	0.03%	0.04%	2028	2531	2395	3142	*serC*	Metabolism of Cofactors, Coenzymes and Vitamins	Vitamin B6 metabolism
RCAP_rcc03447	-0.11	0.5617	3847	3557	0.05%	0.05%	3251	4495	3104	4010	*serA*	Energy Metabolism	Methane metabolism
RCAP_rcc03448	1.89	0	265	1018	0.00%	0.01%	290	363	835	1202	*serine/threonine-protein phosphatase*	Signal Transduction	Kinase/Phosphorelay
RCAP_rcc03449	0.63	0.0141	386	615	0.01%	0.01%	306	504	461	769	*atoB3*	Carbohydrate Metabolism	Glyoxylate and dicarboxylate metabolism
RCAP_rcc03450	-0.22	0.4037	358	304	0.00%	0.00%	263	442	236	372	*pbpC*	Glycan Biosynthesis and Metabolism	Peptidoglycan biosynthesis
RCAP_rcc03451	0.02	0.9424	6757	6830	0.09%	0.09%	5544	7937	6014	7645	*alpha-2-macroglobulin domain-containing protein*	Unknown	Unknown
RCAP_rcc03452	-0.54	0.0013	2169	1472	0.03%	0.02%	1729	2526	1301	1644	*sensor histidine kinase/response regulator receiver protein*	Signal Transduction	Transcription Regulator
RCAP_rcc03453	-0.15	0.1776	1313	1178	0.02%	0.02%	1174	1438	1085	1271	*recQ*	Replication, Recombination and Repair	Unknown
RCAP_rcc03454	0.22	0.3167	1582	1860	0.02%	0.03%	1245	1986	1550	2169	*hypothetical protein*	Unknown	Unknown
RCAP_rcc03455	-0.07	0.8473	364	346	0.01%	0.00%	269	496	254	438	*hypothetical protein*	Unknown	Unknown
RCAP_rcc03456	0.28	0.0053	671	818	0.01%	0.01%	616	749	757	880	*major facilitator superfamily protein*	Unknown	Unknown
RCAP_rcc03457	0.28	0.1963	284	347	0.00%	0.00%	229	341	282	411	*mepA*	Cell Envelope Biosynthesis	Cell Wall Biosynthesis
RCAP_rcc03458	-0.41	0.0001	366	275	0.01%	0.00%	327	398	252	298	*hypothetical protein*	Unknown	Unknown
RCAP_rcc03459	-0.8	0.0003	1614	905	0.02%	0.01%	1178	1983	728	1082	*hypothetical protein*	Unknown	Unknown
RCAP_rcc03460	0.3	0.1202	569	707	0.01%	0.01%	477	677	605	810	*xseB*	Replication, Recombination and Repair	Replication
RCAP_rcc03461	0.58	0.0003	499	756	0.01%	0.01%	406	607	691	821	*ispA*	Lipid	Terpenoid backbone biosynthesis
RCAP_rcc03462	-0.11	0.1727	2784	2577	0.04%	0.03%	2661	2892	2382	2771	*dxs2*	Photosynthesis	Terpenoid backbone biosynthesis
RCAP_rcc03463	0.05	0.7502	344	356	0.00%	0.00%	298	381	319	393	*arcB2*	Amino Acid Metabolism	Arginine and proline metabolism
RCAP_rcc03464	0.23	0.0079	257	303	0.00%	0.00%	248	277	275	330	*HAD superfamily hydrolase*	Xenobiotics Biodegradation and Metabolism	Tetrachloroethene degradation
RCAP_rcc03465	0.34	0.0175	308	392	0.00%	0.01%	274	343	337	446	*membrane transport family protein*	Unknown	Unknown
RCAP_rcc03466	1.06	0	632	1381	0.01%	0.02%	569	812	978	1785	*hypothetical protein*	Unknown	Unknown
RCAP_rcc03467	1.31	0.0064	413	1494	0.01%	0.02%	280	813	652	2336	*hypothetical protein*	Unknown	Unknown
RCAP_rcc03468	0.86	0.0155	1557	3086	0.02%	0.04%	941	2564	1912	4260	*pncA*	Metabolism of Cofactors, Coenzymes and Vitamins	Nicotinate and nicotinamide metabolism
RCAP_rcc03469	0.38	0.0099	1136	1491	0.02%	0.02%	980	1335	1361	1620	*pncB*	Metabolism of Cofactors, Coenzymes and Vitamins	Nicotinate and nicotinamide metabolism
RCAP_rcc03470	0.25	0.0561	984	1175	0.01%	0.02%	877	1104	1061	1288	*phosphate transporter*	Metal and Ion Transport	Unknown
RCAP_rcc03471	-0.19	0.1276	426	373	0.01%	0.01%	368	475	350	397	*NUDIX superfamily hydrolase*	Replication, Recombination and Repair	Unknown
RCAP_rcc03472	0.53	0.0468	282	418	0.00%	0.01%	212	349	301	535	*thrB*	Unknown	Unknown
RCAP_rcc03473	0.66	0	845	1341	0.01%	0.02%	809	930	1263	1419	*gabT2*	Carbohydrate Metabolism	Propanoate metabolism
RCAP_rcc03474	1.15	0	422	946	0.01%	0.01%	429	474	810	1081	*class I peptide chain release factor*	Translation, ribosomal structure and biogenesis	Unknown
RCAP_rcc03475	-0.58	0.0001	766	509	0.01%	0.01%	660	843	440	579	*MiaB family RNA modification enzyme*	Translation, ribosomal structure and biogenesis	Unknown
RCAP_rcc03476	-0.25	0.3017	297	247	0.00%	0.00%	216	366	200	293	*dapF*	Amino Acid Metabolism	Lysine biosynthesis
RCAP_rcc03477	-0.7	0.0001	657	397	0.01%	0.01%	535	775	330	465	*msrB2*	Post-translational Modification, Assembly and Chaperones	Unknown
RCAP_rcc03478	-0.73	0	1108	661	0.02%	0.01%	994	1202	570	752	*msrA2*	Post-translational Modification, Assembly and Chaperones	Unknown
RCAP_rcc03479	-0.62	0	1283	832	0.02%	0.01%	1102	1434	749	915	*fliP*	Motility	Flagellar Assembly
RCAP_rcc03480	0.03	0.8201	273	281	0.00%	0.00%	245	310	255	306	*fliN*	Motility	Flagellar Assembly
RCAP_rcc03481	-0.28	0.0399	295	242	0.00%	0.00%	256	337	224	261	*fliH*	Motility	Flagellar Assembly
RCAP_rcc03482	-0.31	0.007	2681	2154	0.04%	0.03%	2337	2992	2020	2289	*fliF*	Motility	Flagellar Assembly
RCAP_rcc03483	-0.11	0.4465	788	728	0.01%	0.01%	685	903	671	785	*fliL1*	Motility	Flagellar Assembly
RCAP_rcc03484	-0.08	0.5932	279	264	0.00%	0.00%	242	321	242	285	*hypothetical protein*	Unknown	Unknown
RCAP_rcc03485	-0.45	0.0126	872	634	0.01%	0.01%	698	1028	566	701	*hypothetical protein*	Unknown	Unknown
RCAP_rcc03486	-0.7	0.0002	2417	1461	0.03%	0.02%	1880	2881	1257	1666	*motA*	Motility	Chemotaxis
RCAP_rcc03487	-0.8	0	1909	1093	0.03%	0.01%	1685	2048	1045	1141	*hypothetical protein*	Unknown	Unknown
RCAP_rcc03488	0.34	0.0009	367	464	0.01%	0.01%	330	412	441	487	*hemS*	Metabolism of Cofactors, Coenzymes and Vitamins	Heme Biosynthesis
RCAP_rcc03489	-0.08	0.4318	809	764	0.01%	0.01%	741	869	706	822	*hemN3*	Metabolism of Cofactors, Coenzymes and Vitamins	Heme Biosynthesis
RCAP_rcc03490	0.03	0.8692	1370	1400	0.02%	0.02%	1220	1551	1225	1575	*endoribonuclease, L-PSP family*	Unknown	Unknown
RCAP_rcc03491	0.02	0.8847	1053	1070	0.01%	0.01%	972	1145	972	1168	*nucleoside-triphosphatase*	Metabolism of Cofactors, Coenzymes and Vitamins	Thiamine metabolism
RCAP_rcc03492	0.07	0.6846	1237	1298	0.02%	0.02%	1044	1429	1189	1407	*rph*	Translation, ribosomal structure and biogenesis	Unknown
RCAP_rcc03493	-0.01	0.9062	1459	1445	0.02%	0.02%	1327	1581	1344	1546	*hrcA*	Stress Response	Unknown
RCAP_rcc03494	0.21	0.0585	880	1023	0.01%	0.01%	777	995	952	1094	*grpE*	Post-translational Modification, Assembly and Chaperones	Unknown
RCAP_rcc03495	0.06	0.7036	1189	1239	0.02%	0.02%	1024	1353	1182	1296	*mutS*	Replication, Recombination and Repair	Repair
RCAP_rcc03496	-0.22	0.3161	6473	5503	0.09%	0.07%	5197	7715	4617	6388	*maeB2*	Energy Metabolism	Carbon fixation in photosynthetic organisms
RCAP_rcc03497	-0.32	0.0028	671	537	0.01%	0.01%	595	731	502	573	*rbsK*	Carbohydrate Metabolism	Pentose phosphate pathway
RCAP_rcc03498	-0.06	0.7273	957	914	0.01%	0.01%	784	1137	831	998	*phoB*	Signal Transduction	Transcription Regulator
RCAP_rcc03499	0.15	0.4864	1542	1712	0.02%	0.02%	1246	1916	1481	1943	*phoU*	Signal Transduction	Transcription Regulator
RCAP_rcc03500	0.16	0.4353	1321	1484	0.02%	0.02%	1044	1670	1301	1667	*pstB*	Metal and Ion Transport	Unknown
RCAP_rcc03501	0.43	0.0105	1266	1719	0.02%	0.02%	1066	1574	1523	1915	*pstA*	Metal and Ion Transport	Unknown
RCAP_rcc03502	0.32	0.0588	1380	1731	0.02%	0.02%	1133	1705	1554	1909	*pstC*	Metal and Ion Transport	Unknown
RCAP_rcc03503	0.25	0.352	2776	3329	0.04%	0.05%	1821	4012	2880	3777	*pstS*	Metal and Ion Transport	Unknown
RCAP_rcc03504	-0.05	0.9238	281	270	0.00%	0.00%	156	396	129	410	*cbiX*	Metabolism of Cofactors, Coenzymes and Vitamins	Cobalamin Biosynthesis

RCAP_rcc03505	-0.35	0.0156	1221	953	0.02%	0.01%	1019	1396	860	1046	*hypothetical protein*	Unknown	Unknown
RCAP_rcc03506	0.37	0.0477	221	287	0.00%	0.00%	186	265	254	321	*YCII-related domain-containing protein*	Unknown	Unknown
RCAP_rcc03507	0.16	0.3272	195	218	0.00%	0.00%	167	221	190	246	*gpsA*	Lipid Metabolism	Glycerophospholipid metabolism
RCAP_rcc03508	0.52	0.0531	180	264	0.00%	0.00%	124	231	203	326	*gcp*	Post-translational Modification, Assembly and Chaperones	Peptidase
RCAP_rcc03509	0.38	0.1649	188	248	0.00%	0.00%	134	235	196	300	*hemD*	Metabolism of Cofactors, Coenzymes and Vitamins	Heme Biosynthesis
RCAP_rcc03510	0.86	0.0005	911	1723	0.01%	0.02%	663	1219	1442	2004	*hypothetical protein*	Unknown	Unknown
RCAP_rcc03511	0.47	0	3785	5266	0.05%	0.07%	3729	4082	4837	5695	*HemY domain-containing protein*	Metabolism of Cofactors, Coenzymes and Vitamins	Heme Biosynthesis
RCAP_rcc03512	-0.47	0.0219	763	543	0.01%	0.01%	589	921	471	615	*fliL2*	Motility	Flagellar Assembly
RCAP_rcc03513	-0.27	0.0489	945	782	0.01%	0.01%	805	1078	731	833	*flgH*	Motility	Flagellar Assembly
RCAP_rcc03514	0.21	0.4124	192	223	0.00%	0.00%	150	238	187	260	*flgA*	Motility	Flagellar Assembly
RCAP_rcc03515	-0.17	0.3655	917	812	0.01%	0.01%	740	1098	750	874	*flgG*	Motility	Flagellar Assembly
RCAP_rcc03516	-0.06	0.8162	1329	1275	0.02%	0.02%	1056	1631	1142	1408	*flgF*	Motility	Flagellar Assembly
RCAP_rcc03517	-0.18	0.519	1119	977	0.02%	0.01%	821	1445	835	1119	*fliQ*	Motility	Flagellar Assembly
RCAP_rcc03518	0.17	0.3714	1195	1351	0.02%	0.02%	1011	1464	1189	1512	*fliE*	Motility	Flagellar Assembly
RCAP_rcc03519	-0.45	0.028	2179	1581	0.03%	0.02%	1694	2674	1381	1782	*flgC*	Motility	Flagellar Assembly
RCAP_rcc03520	-0.46	0.0004	1540	1117	0.02%	0.02%	1334	1733	1007	1226	*flgB*	Motility	Flagellar Assembly
RCAP_rcc03521	-0.79	0.0004	856	483	0.01%	0.01%	605	1057	417	548	*fliI*	Motility	Flagellar Assembly
RCAP_rcc03522	-1.13	0	2820	1259	0.04%	0.02%	2284	3185	1036	1482	*flagellar protein*	Motility	Flagellar Assembly
RCAP_rcc03523	-0.55	0.0003	2828	1919	0.04%	0.03%	2413	3184	1675	2163	*flbT*	Motility	Flagellar Assembly
RCAP_rcc03524	-0.89	0	6148	3257	0.08%	0.04%	4941	7108	2723	3792	*flagellar FlaF family protein*	Motility	Flagellar Assembly
RCAP_rcc03525	-0.57	0.0001	69719	46683	0.96%	0.63%	58667	78463	41920	51447	*flaA*	Motility	Flagellar Assembly
RCAP_rcc03526	-0.41	0.0004	1094	821	0.02%	0.01%	989	1199	734	908	*hypothetical protein*	Unknown	Unknown
RCAP_rcc03527	-0.76	0	1202	695	0.02%	0.01%	956	1415	589	802	*flgJ*	Motility	Flagellar Assembly
RCAP_rcc03528	0.34	0.1115	660	846	0.01%	0.01%	535	791	697	996	*hypothetical protein*	Unknown	Unknown
RCAP_rcc03529	-0.39	0.0216	2106	1599	0.03%	0.02%	1723	2457	1409	1789	*flgD*	Motility	Flagellar Assembly
RCAP_rcc03530	-0.15	0.1103	3294	2962	0.05%	0.04%	3066	3454	2708	3217	*ubiB*	Energy Metabolism	Biosynthesis of Ubiquinone
RCAP_rcc03531	-0.04	0.8824	4881	4738	0.07%	0.06%	3830	5994	3899	5577	*ubiE*	Energy Metabolism	Biosynthesis of Ubiquinone
RCAP_rcc03532	-0.18	0.0929	231	203	0.00%	0.00%	208	247	186	221	*mutM*	Replication, Recombination and Repair	Unknown
RCAP_rcc03533	0.17	0.2033	1193	1340	0.02%	0.02%	1095	1327	1196	1485	*paaF*	Xenobiotics Biodegradation and Metabolism	Caprolactam degradation
RCAP_rcc03534	-0.24	0.1596	2991	2523	0.04%	0.03%	2564	3410	2172	2874	*rpsT*	Translation, ribosomal structure and biogenesis	Unknown

Table S6. Percentage of total mRNA expression levels under anaerobic photosynthetic and dark aerobic growth.

Motility Group	Photo (%)	Aero (%)	Change (%)	Photo Activated	Photo Repressed	Total Genes
Gas vesicle production	0.01%	0.04%	-65%	0	10	10
Chemotaxis	0.59%	0.94%	-36%	1	25	26
Flagella assembly	1.16%	1.68%	-30%	0	18	18
Aerotaxis	0.06%	0.08%	-19%	1	2	3

Table S7. RNA-seq differential expression for FnrL, RegA and CrtJ from anaerobic photosynthecally grown cells defined as log2FC([WT]/[mutant])

**ID**	**FnrL log2FC**	**FnrL padj**	**RegA log2FC**	**RegA padj**	**CrtJ log2FC**	**CrtJ padj**	**Gene**	**Global Function**	**Pathway Name**
RCAP_rcc00001	0.274	0.085	0.525	0.000	-0.030	0.931	*dnaA*	Replication, Recombination and Repair	Replication
RCAP_rcc00002	0.247	0.116	-0.062	0.558	-0.053	0.837	*dnaN*	Replication, Recombination and Repair	Replication
RCAP_rcc00003	-0.360	0.132	0.090	0.642	0.095	0.809	*recF*	Replication, Recombination and Repair	Repair
RCAP_rcc00004	0.177	0.324	0.107	0.333	-0.125	0.622	*gyrB*	Replication, Recombination and Repair	Unknown
RCAP_rcc00005	-0.236	0.201	-0.050	0.777	0.155	0.667	*ntaB*	Unknown	Unknown
RCAP_rcc00006	-0.097	0.768	1.554	0.000	0.195	0.559	*motB*	Motility	Chemotaxis
RCAP_rcc00007	0.061	0.836	3.484	0.000	0.192	0.579	*flgE*	Motility	Flagellar Assembly
RCAP_rcc00008	-0.118	0.637	2.829	0.000	0.208	0.506	*flgK*	Motility	Flagellar Assembly
RCAP_rcc00009	-0.445	0.068	2.924	0.000	0.324	0.178	*flgL*	Motility	Flagellar Assembly
RCAP_rcc00010	-0.589	0.015	2.753	0.000	0.470	0.005	*flgI*	Motility	Flagellar Assembly
RCAP_rcc00011	-0.370	0.103	-0.722	0.000	0.325	0.356	*hemolysin D*	Trafficking and Secretion	Secretion
RCAP_rcc00012	-0.179	0.609	-0.403	0.281	-0.025	0.957	*ABC transporter ATP-binding protein*	Defense Mechanisms	Unknown
RCAP_rcc00013	-0.065	0.870	-0.404	NA	0.040	0.894	*inner membrane transport permease*	Defense Mechanisms	Unknown
RCAP_rcc00014	0.358	0.069	-0.066	0.814	0.068	0.863	*xylA*	Carbohydrate Metabolism	Pentose and glucuronate interconversions
RCAP_rcc00015	0.113	0.498	0.273	0.386	0.291	0.083	*xylB*	Carbohydrate Metabolism	Pentose and glucuronate interconversions
RCAP_rcc00016	0.397	0.069	0.348	0.281	0.048	0.910	*xylG*	Carbohydrate Metabolism	Xylose Transport
RCAP_rcc00017	0.277	0.133	0.467	0.229	0.168	0.601	*xylH*	Carbohydrate Metabolism	Xylose Transport
RCAP_rcc00018	-0.077	0.776	0.335	0.408	0.405	0.091	*xylF*	Carbohydrate Metabolism	Xylose Transport
RCAP_rcc00019	-0.220	0.224	0.455	0.073	0.396	0.008	*xylR*	Signal Transduction	Transcription Regulator
RCAP_rcc00020	-0.047	0.872	-0.032	0.913	0.085	0.700	*FG-GAP repeat domain-containing protein*	Unknown	Unknown
RCAP_rcc00021	-0.114	0.611	-1.155	0.000	0.234	0.241	*cytochrome c peroxidase*	Energy Metabolism	Aerobic/Anaerobic Respiration
RCAP_rcc00022	0.462	0.076	-1.022	0.000	-0.112	0.715	*aldo/keto reductase family oxidoreductase*	Energy Metabolism	Aerobic/Anaerobic Respiration
RCAP_rcc00023	-0.255	0.423	0.219	0.507	-0.361	0.046	*ribonuclease BN*	Unknown	Unknown
RCAP_rcc00024	-0.189	0.688	0.065	0.883	-0.159	0.562	*gcdH*	Lipid Metabolism	Unknown
RCAP_rcc00025	-0.149	0.631	-0.390	0.062	0.303	0.480	*LysR family transcriptional regulator*	Signal Transduction	Transcription Regulator
RCAP_rcc00026	-0.390	0.062	-0.703	0.000	0.534	0.005	*hypothetical protein*	Unknown	Unknown
RCAP_rcc00027	0.152	0.497	-0.283	0.028	0.134	0.482	*surface antigen*	Cell Envelope Biosynthesis	Cell Wall Biosynthesis
RCAP_rcc00028	0.342	0.127	2.885	0.000	-0.307	0.231	*idi1*	Photosynthesis	Terpenoid backbone biosynthesis
RCAP_rcc00029	0.682	0.003	0.395	0.002	-0.274	0.455	*secB*	Trafficking and Secretion	Secretion
RCAP_rcc00030	0.391	0.089	0.911	0.000	-0.578	0.009	*fxsA*	Unknown	Unknown
RCAP_rcc00031	0.063	0.836	0.271	0.067	0.073	0.877	*import inner membrane translocase subunit Tim44*	Unknown	Unknown
RCAP_rcc00032	0.139	0.494	0.606	0.002	-0.495	0.012	*Smr protein/MutS2*	Unknown	Unknown
RCAP_rcc00033	0.167	0.571	-0.040	0.856	-0.218	0.135	*hypothetical protein*	Unknown	Unknown
RCAP_rcc00034	0.442	0.015	0.209	0.278	-0.006	0.989	*hslU*	Post-translational Modification, Assembly and Chaperones	Unknown
RCAP_rcc00035	0.590	0.001	0.406	0.039	-0.125	0.701	*hslV*	Post-translational Modification, Assembly and Chaperones	Peptidase
RCAP_rcc00036	0.444	0.003	-0.120	0.358	-0.234	0.482	*trxA1*	Post-translational Modification, Assembly and Chaperones	Unknown
RCAP_rcc00037	-0.116	0.636	-0.100	0.656	0.260	0.358	*UvrD/REP helicase*	Replication, Recombination and Repair	Unknown
RCAP_rcc00038	-0.297	0.084	-0.128	0.438	0.342	0.173	*addB*	Unknown	Unknown
RCAP_rcc00039	0.122	0.613	0.115	0.556	0.032	0.928	*nucleotidyltransferase*	Cell Envelope Biosynthesis	Cell Wall Biosynthesis
RCAP_rcc00040	-0.312	0.137	-0.092	0.685	0.358	0.211	*aminoglycoside phosphotransferase*	Unknown	Unknown
RCAP_rcc00041	-0.365	0.087	0.384	0.111	0.277	0.294	*hypothetical protein*	Unknown	Unknown
RCAP_rcc00042	-0.245	0.109	2.758	0.000	0.083	0.758	*PAS/PAC sensor domain-containing protein*	Motility	Aerotaxis
RCAP_rcc00043	0.074	0.739	-0.809	0.000	0.017	0.948	*regB*	Signal Transduction	Kinase/Phosphorelay
RCAP_rcc00044	0.152	0.487	-0.002	0.993	-0.015	0.961	*senC*	Unknown	Unknown
RCAP_rcc00045	0.350	0.104	5.664	0.000	-0.192	0.577	*regA1*	Signal Transduction	Transcription Regulator
RCAP_rcc00046	0.446	0.031	0.203	0.240	-0.283	0.362	*hvrA*	Signal Transduction	Transcription Regulator
RCAP_rcc00047	0.109	0.613	0.445	0.001	-0.126	0.626	*hvrB*	Transcription	Unknown
RCAP_rcc00048	0.121	0.733	-0.963	0.001	-0.415	0.007	*metal dependent phosphohydrolase*	Unknown	Unknown
RCAP_rcc00049	0.542	0.002	0.214	0.205	-0.241	NA	*ahcY*	Post-translation	Cysteine and methionine metabolism
RCAP_rcc00050	0.939	0.001	-0.363	0.018	-0.307	0.291	*hypothetical protein*	Unknown	Unknown
RCAP_rcc00051	-0.322	0.310	-0.436	0.006	0.604	0.092	*enoyl-CoA hydratase/isomerase*	Xenobiotics Biodegradation and Metabolism	Caprolactam degradation
RCAP_rcc00052	0.509	0.039	-0.531	0.001	-0.149	0.633	*hypothetical protein*	Unknown	Unknown
RCAP_rcc00053	-0.106	0.635	-0.114	0.566	0.280	0.144	*U62 family peptidase*	Post-translational Modification, Assembly and Chaperones	Peptidase
RCAP_rcc00054	-0.420	0.059	0.143	0.478	0.393	0.190	*inositol monophosphatase*	Carbohydrate Metabolism	Unknown
RCAP_rcc00055	0.037	0.901	4.227	0.000	-0.073	0.866	*transglycosylase, Slt family*	Cell Envelope Biosynthesis	Cell Wall Biosynthesis
RCAP_rcc00056	-0.088	0.700	4.799	0.000	0.159	0.640	*flhA*	Motility	Flagellar Assembly
RCAP_rcc00057	-0.160	0.497	4.266	0.000	0.203	0.527	*fliR*	Motility	Flagellar Assembly
RCAP_rcc00058	-0.024	0.927	3.916	0.000	-0.039	0.931	*flhB*	Motility	Flagellar Assembly
RCAP_rcc00059	-0.426	0.021	1.830	0.000	0.198	0.477	*hypothetical protein*	Unknown	Unknown
RCAP_rcc00060	0.239	0.276	0.376	0.026	-0.160	0.542	*parB*	Cell Division	Chromosome Partitioning
RCAP_rcc00061	0.278	0.164	0.318	0.038	-0.245	0.266	*parA*	Cell Division	Chromosome Partitioning
RCAP_rcc00062	0.190	0.492	0.543	0.002	-0.123	0.692	*gidB*	Cell Envelope Biosynthesis	Cell Wall Biosynthesis
RCAP_rcc00063	0.160	0.467	0.419	0.024	-0.224	0.351	*gidA*	Cell Division	Chromosome Partitioning
RCAP_rcc00064	-0.107	0.520	0.639	0.003	-0.138	0.509	*trmE*	Unknown	Unknown
RCAP_rcc00065	0.420	0.121	0.495	0.002	-0.189	0.587	*rho*	Transcription	Unknown
RCAP_rcc00066	0.779	0.004	0.484	0.000	-0.285	0.474	*hypothetical protein*	Unknown	Unknown
RCAP_rcc00067	0.396	0.131	0.273	0.300	0.169	0.641	*Maf-like protein*	Cell Division	Chromosome Partitioning
RCAP_rcc00068	0.379	0.163	0.186	0.380	-0.060	0.890	*aroE*	Amino Acid Metabolism	Phenylalanine, tyrosine and tryptophan biosynthesis
RCAP_rcc00069	-0.017	0.963	0.430	0.043	0.050	0.916	*coaE*	Metabolism of Cofactors, Coenzymes and Vitamins	Pantothenate and CoA biosynthesis
RCAP_rcc00070	0.309	0.194	0.504	0.000	-0.058	0.871	*dnaQ*	Replication, Recombination and Repair	Replication
RCAP_rcc00071	0.245	0.401	0.633	0.000	-0.257	0.284	*nadA*	Metabolism of Cofactors, Coenzymes and Vitamins	Nicotinate and nicotinamide metabolism
RCAP_rcc00072	-0.930	0.002	0.511	0.000	0.363	0.270	*nadB*	Metabolism of Cofactors, Coenzymes and Vitamins	Nicotinate and nicotinamide metabolism
RCAP_rcc00073	-0.330	0.083	0.349	0.007	0.108	0.710	*nadC*	Metabolism of Cofactors, Coenzymes and Vitamins	Nicotinate and nicotinamide metabolism
RCAP_rcc00074	0.191	0.514	-0.678	0.000	-0.094	0.779	*nudH*	Metabolism of Cofactors, Coenzymes and Vitamins	Folate biosynthesis
RCAP_rcc00075	0.222	0.268	-0.144	0.170	-0.155	NA	*ctpA*	Cell Envelope Biosynthesis	Cell Wall Biosynthesis

RCAP_rcc00076	-0.020	0.942	-0.259	0.019	0.101	0.671	*M23 family peptidase*	Post-translational Modification, Assembly and Chaperones	Peptidase
RCAP_rcc00077	0.336	0.079	-0.195	0.234	-0.145	0.531	*gpmI*	Carbohydrate Metabolism	Glycolysis / Gluconeogenesis
RCAP_rcc00078	-0.288	0.289	0.337	0.361	-0.410	0.149	*hypothetical protein*	Unknown	Unknown
RCAP_rcc00079	0.257	0.322	-0.017	0.964	-0.428	0.033	*iojap-related protein*	Unknown	Unknown
RCAP_rcc00080	0.442	0.078	0.598	0.000	-0.779	0.000	*hypothetical protein*	Unknown	Unknown
RCAP_rcc00081	0.159	0.492	0.505	0.000	-0.323	0.041	*leuC*	Amino Acid Metabolism	Valine, leucine and isoleucine biosynthesis
RCAP_rcc00082	0.512	0.007	0.347	0.014	-0.367	0.195	*leuD*	Amino Acid Metabolism	Valine, leucine and isoleucine biosynthesis
RCAP_rcc00083	0.029	0.913	-0.332	0.014	-0.236	0.339	*hypothetical protein*	Unknown	Unknown
RCAP_rcc00084	-0.620	0.009	-0.677	0.000	0.122	0.719	*hypothetical protein*	Unknown	Unknown
RCAP_rcc00085	0.357	0.081	-0.177	0.351	-0.052	0.881	*leuB*	Amino Acid Metabolism	Valine, leucine and isoleucine biosynthesis
RCAP_rcc00086	-0.078	0.759	0.377	0.010	-0.107	0.655	*hypothetical protein*	Unknown	Unknown
RCAP_rcc00087	-0.564	0.124	1.129	0.000	-0.178	0.705	*hypothetical protein*	Unknown	Unknown
RCAP_rcc00088	-0.007	0.990	0.489	0.279	0.210	0.648	*hypothetical protein*	Unknown	Unknown
RCAP_rcc00089	0.108	0.706	0.721	0.005	-0.151	0.715	*cation diffusion facilitator family transporter*	Metal and Ion Transport	Unknown
RCAP_rcc00090	-0.192	0.648	1.161	0.000	0.033	0.960	*feoA1*	Metal, Ion, Cofactor Transport	Iron and Heme Transport
RCAP_rcc00091	-0.658	0.093	1.244	0.000	0.375	0.387	*feoA2*	Metal, Ion, Cofactor Transport	Iron and Heme Transport
RCAP_rcc00092	0.098	0.605	1.182	0.000	-0.328	0.113	*feoB1*	Metal, Ion, Cofactor Transport	Iron and Heme Transport
RCAP_rcc00093	-0.697	0.081	1.405	0.001	0.240	NA	*hypothetical protein*	Unknown	Unknown
RCAP_rcc00094	-0.737	0.069	1.263	0.001	0.005	0.991	*hmuV*	Metal, Ion, Cofactor Transport	Iron and Heme Transport
RCAP_rcc00095	0.074	NA	0.855	0.078	0.091	NA	*hmuU*	Metal, Ion, Cofactor Transport	Iron and Heme Transport
RCAP_rcc00096	0.113	NA	0.558	0.422	-0.036	NA	*hmuT*	Metal, Ion, Cofactor Transport	Iron and Heme Transport
RCAP_rcc00097	0.309	0.469	0.850	0.043	0.154	0.751	*hmuS*	Metal, Ion, Cofactor Transport	Iron and Heme Transport
RCAP_rcc00098	0.301	0.244	0.969	0.000	-0.401	0.279	*hmuR*	Metal, Ion, Cofactor Transport	Iron and Heme Transport
RCAP_rcc00099	-0.021	0.965	1.266	0.000	0.354	0.428	*ABC transporter ATP-binding protein*	Metal, Ion, Cofactor Transport	Nickel Transport
RCAP_rcc00100	-0.305	0.479	1.185	0.000	0.714	0.064	*ABC transporter ATP-binding protein*	Amino Acid Metabolism	Amino Acid Transport
RCAP_rcc00101	0.687	0.011	1.312	0.000	0.435	0.248	*ABC transporter permease*	Amino Acid Metabolism	Amino Acid Transport
RCAP_rcc00102	-0.266	0.520	1.177	0.000	0.923	0.007	*ABC transporter permease*	Metal, Ion, Cofactor Transport	Nickel Transport
RCAP_rcc00103	0.914	0.000	1.405	0.000	0.268	0.380	*ABC transporter periplasmic substrate-binding protein*	Metal, Ion, Cofactor Transport	Nickel Transport
RCAP_rcc00104	-0.061	0.840	-0.977	0.001	-0.007	0.989	*hypothetical protein*	Unknown	Unknown
RCAP_rcc00105	-0.197	NA	-0.311	0.592	0.248	NA	*fhuC1*	Metal, Ion, Cofactor Transport	Iron and Heme Transport
RCAP_rcc00106	-0.267	0.555	0.202	0.671	0.106	0.823	*fhuB1*	Metal, Ion, Cofactor Transport	Iron and Heme Transport
RCAP_rcc00107	0.069	NA	-0.003	0.996	-0.090	0.868	*fhuB2*	Metal, Ion, Cofactor Transport	Iron and Heme Transport
RCAP_rcc00108	0.166	0.730	-0.021	0.966	-0.028	0.965	*fhuD1*	Metal, Ion, Cofactor Transport	Iron and Heme Transport
RCAP_rcc00109	0.093	0.806	-0.014	0.970	-0.302	0.446	*hypothetical protein*	Unknown	Unknown
RCAP_rcc00110	0.012	0.981	0.153	0.795	-0.169	0.715	*esterase*	Unknown	Unknown
RCAP_rcc00111	0.324	0.298	0.561	0.023	-0.306	0.460	*fhuE*	Metal, Ion, Cofactor Transport	Iron and Heme Transport
RCAP_rcc00112	0.282	0.526	1.165	0.014	-0.690	0.073	*AraC family transcriptional regulator*	Signal Transduction	Transcription Regulator
RCAP_rcc00113	0.389	0.015	0.363	0.005	-0.307	0.157	*rnd1*	Translation, ribosomal structure and biogenesis	Unknown
RCAP_rcc00114	-0.504	0.035	-0.002	0.993	0.177	0.465	*kdsD*	Cell Envelope Biosynthesis	Cell Wall Biosynthesis
RCAP_rcc00115	0.076	0.817	-0.034	0.859	0.104	0.760	*hypothetical protein*	Unknown	Unknown
RCAP_rcc00116	-0.132	0.591	0.058	0.758	0.245	0.352	*OstA family protein*	Predicted Function	Unknown
RCAP_rcc00117	0.132	0.592	-0.097	0.647	-0.003	0.991	*ABC transporter ATP-binding protein*	Predicted Function	Unknown
RCAP_rcc00118	0.569	0.023	-1.244	0.000	-0.129	0.736	*sigma 54 modulation protein/ribosomal protein S30EA*	Translation, ribosomal structure and biogenesis	Unknown
RCAP_rcc00119	0.208	0.436	-0.564	0.000	-0.100	0.740	*ptsN*	Signal Transduction	Transcription Regulator
RCAP_rcc00120	-0.393	0.027	-0.301	0.033	0.333	0.150	*hypothetical protein*	Unknown	Unknown
RCAP_rcc00121	-0.152	0.371	-0.032	0.865	0.106	0.654	*family 14 glycosyl transferase*	Unknown	Unknown
RCAP_rcc00122	0.322	0.120	0.253	0.181	-0.112	0.748	*hypothetical protein*	Unknown	Unknown
RCAP_rcc00123	-0.062	0.833	0.386	0.063	-0.158	0.626	*hypothetical protein*	Unknown	Unknown
RCAP_rcc00124	0.316	0.264	-0.018	0.913	-0.103	0.602	*galE*	Carbohydrate Metabolism	Galactose Metabolism
RCAP_rcc00125	0.701	0.001	-0.027	0.896	-0.269	0.334	*galU*	Carbohydrate Metabolism	Galactose Metabolism
RCAP_rcc00126	-0.008	0.970	-0.287	0.045	0.173	0.349	*kdsB*	Glycan Biosynthesis and Metabolism	Lipopolysaccharide biosynthesis
RCAP_rcc00127	-0.013	0.956	-0.011	0.953	0.210	0.358	*cysQ*	Energy Metabolism	Sulfur metabolism
RCAP_rcc00128	0.447	0.045	0.040	0.791	-0.217	0.489	*ABC transporter permease*	Unknown	Unknown
RCAP_rcc00129	-0.065	0.763	0.386	0.005	0.133	0.471	*LacI family transcriptional regulator*	Signal Transduction	Transcription Regulator
RCAP_rcc00130	-0.258	0.181	0.139	0.525	-0.101	0.613	*radical SAM family protein*	Energy Metabolism	Unknown
RCAP_rcc00131	-0.601	0.082	0.395	0.014	0.306	0.501	*tRNA/rRNA cytosine-C5-methylase*	Translation, ribosomal structure and biogenesis	Unknown
RCAP_rcc00132	-0.140	0.747	0.403	0.136	0.164	0.731	*hypothetical protein*	Unknown	Unknown
RCAP_rcc00133	0.477	0.031	0.651	0.000	-0.333	0.293	*hypothetical protein*	Unknown	Unknown
RCAP_rcc00134	-0.434	0.312	0.028	0.956	-0.020	0.974	*hypothetical protein*	Unknown	Unknown
RCAP_rcc00135	-0.155	NA	-0.457	0.533	0.039	NA	*lolD1*	Predicted Function	Unknown
RCAP_rcc00136	0.297	0.267	0.290	0.384	-0.276	0.356	*phoA*	Metabolism of Cofactors, Coenzymes and Vitamins	Folate biosynthesis
RCAP_rcc00137	-0.198	0.640	-0.043	0.932	-0.193	0.673	*hypothetical protein*	Unknown	Unknown
RCAP_rcc00138	0.181	0.439	-0.073	0.731	-0.142	0.661	*dnaE2*	Replication, Recombination and Repair	Replication
RCAP_rcc00139	-0.366	0.404	-0.452	0.232	0.314	0.441	*nucleotidyltransferase/DNA polymerase involved in DNA repair*	Replication, Recombination and Repair	Repair
RCAP_rcc00140	-0.816	0.002	-0.061	0.845	0.114	0.814	*lipoprotein*	Predicted Function	Unknown
RCAP_rcc00141	-0.231	0.363	0.270	0.499	-0.316	0.258	*hbdA*	Carbohydrate Metabolism	Butanoate metabolism
RCAP_rcc00142	-0.354	0.245	2.440	0.000	0.263	0.383	*hypothetical protein*	Unknown	Unknown
RCAP_rcc00143	0.099	NA	-0.267	0.301	0.049	0.773	*etfA*	Energy Metabolism	Aerobic/Anaerobic Metabolism
RCAP_rcc00144	0.073	NA	0.041	0.891	0.073	0.716	*etfB*	Energy Metabolism	Aerobic/Anaerobic Respiration
RCAP_rcc00145	0.526	0.016	0.113	0.470	-0.259	0.393	*short-chain dehydrogenase/reductase family oxidoreductase*	Lipid transport and metabolism	Unknown
RCAP_rcc00146	-0.443	0.033	0.030	0.826	0.265	0.168	*parC*	Replication, Recombination and Repair	Unknown
RCAP_rcc00147	0.295	0.127	-0.162	0.427	-0.135	0.670	*tuf1*	Translation, ribosomal structure and biogenesis	Unknown
RCAP_rcc00148	-0.804	0.000	0.080	0.561	0.403	0.315	*CDA peptide synthetase III*	Lipid Metabolism	Fatty acid metabolism
RCAP_rcc00149	0.298	0.378	0.413	0.062	-0.131	0.637	*hypothetical protein*	Unknown	Unknown
RCAP_rcc00150	0.660	0.041	0.019	0.962	-0.184	0.701	*hypothetical protein*	Unknown	Unknown
RCAP_rcc00151	0.950	0.000	0.543	0.037	-0.241	0.524	*hemN1*	Metabolism of Cofactors, Coenzymes and Vitamins	Heme Biosynthesis
RCAP_rcc00152	0.082	0.844	1.592	0.000	-0.122	0.782	*peptide ABC transporter periplasmic peptide-binding protein*	Amino Acid Metabolism	Amino Acid Transport

RCAP_rcc00153	-0.219	0.603	1.020	0.004	-0.068	0.902	*peptide ABC transporter permease*	Amino Acid Metabolism	Amino Acid Transport
RCAP_rcc00154	-0.431	0.317	-0.071	0.872	0.174	NA	*peptide ABC transporter permease*	Amino Acid Metabolism	Amino Acid Transport
RCAP_rcc00155	-0.269	0.543	0.219	0.504	0.508	0.212	*peptide ABC transporter ATP-binding protein*	Predicted Function	Nickel Transport
RCAP_rcc00156	0.284	0.126	0.626	0.000	-0.160	0.572	*alsT*	Amino Acid Metabolism	Unknown
RCAP_rcc00157	0.264	0.161	-0.279	0.030	-0.004	0.990	*hypothetical protein*	Unknown	Unknown
RCAP_rcc00158	0.428	0.047	-0.827	0.000	-0.024	0.953	*M10 family peptidase*	Post-translational Modification, Assembly and Chaperones	Peptidase
RCAP_rcc00159	0.204	0.402	-0.620	0.000	0.033	0.899	*hypothetical protein*	Unknown	Unknown
RCAP_rcc00160	-0.415	0.148	0.429	0.004	0.272	0.356	*mtgA*	Nucleotide Metabolism	Purine metabolism
RCAP_rcc00161	0.076	0.729	0.297	0.130	-0.140	0.510	*gltB*	Energy Metabolism	Nitrogen metabolism
RCAP_rcc00162	0.011	0.961	0.424	0.109	-0.285	0.229	*hypothetical protein*	Unknown	Unknown
RCAP_rcc00163	-0.040	0.908	0.407	0.109	-0.080	0.713	*gltD*	Energy Metabolism	Nitrogen metabolism
RCAP_rcc00164	0.069	0.747	0.782	0.000	-0.169	0.514	*uppP*	Glycan Biosynthesis and Metabolism	Peptidoglycan biosynthesis
RCAP_rcc00165	0.035	0.891	0.987	0.000	-0.001	0.995	*NAD-dependent epimerase/dehydratase*	Carbohydrate Metabolism	Unknown
RCAP_rcc00166	0.301	0.199	-0.242	0.097	-0.176	0.529	*sulfotransferase*	Unknown	Unknown
RCAP_rcc00167	-0.043	0.795	-0.310	0.002	0.136	0.315	*family 2 glycosyl transferase*	Cell Envelope Biosynthesis	Cell Wall Biosynthesis
RCAP_rcc00168	0.308	0.150	-0.346	0.049	-0.147	0.633	*ABC transporter polysaccharide inner membrane subunit*	Carbohydrate Metabolism	Unknown
RCAP_rcc00169	0.145	0.377	-0.117	0.371	0.024	0.944	*kpsE1*	Cell Envelope Biosynthesis	Cell Wall Biosynthesis
RCAP_rcc00170	0.106	0.586	-0.165	0.430	-0.143	0.516	*kpsT1*	Carbohydrate Metabolism	Unknown
RCAP_rcc00171	-0.948	0.006	1.552	0.000	0.251	0.582	*hypothetical protein*	Unknown	Unknown
RCAP_rcc00172	-0.874	0.001	-0.492	0.000	0.529	0.081	*glycosyl transferase*	Cell Envelope Biosynthesis	Cell Wall Biosynthesis
RCAP_rcc00173	0.039	0.845	-0.410	0.000	0.090	0.714	*rfbA*	Cell Envelope Biosynthesis	Cell Wall Biosynthesis
RCAP_rcc00174	-0.623	0.007	-0.667	0.000	0.506	0.131	*rfbD*	Cell Envelope Biosynthesis	Cell Wall Biosynthesis
RCAP_rcc00175	-0.586	0.003	-0.328	0.052	0.464	0.036	*hypothetical protein*	Unknown	Unknown
RCAP_rcc00176	0.362	0.044	-0.218	0.038	-0.016	0.970	*rfbB*	Cell Envelope Biosynthesis	Cell Wall Biosynthesis
RCAP_rcc00177	0.612	0.002	-0.474	0.002	-0.135	0.703	*rfbC*	Cell Envelope Biosynthesis	Cell Wall Biosynthesis
RCAP_rcc00178	-0.561	0.099	-0.233	0.325	0.553	0.099	*hemolysin-type calcium-binding repeat family protein*	Trafficking and Secretion	Secretion
RCAP_rcc00179	-0.040	0.857	-0.011	0.950	0.016	0.948	*ilvA*	Amino Acid Metabolism	Valine, leucine and isoleucine biosynthesis
RCAP_rcc00180	-0.125	0.707	1.817	0.000	0.029	0.951	*Hpt domain-containing protein*	Unknown	Unknown
RCAP_rcc00181	-0.249	0.336	3.552	0.000	0.238	0.267	*response regulator receiver domain/protein phosphatase 2C domain-cont*	Signal Transduction	Transcription Regulator
RCAP_rcc00182	-0.135	0.607	0.260	0.258	-0.079	0.780	*hemolysin D*	Trafficking and Secretion	Secretion
RCAP_rcc00183	-0.335	0.040	0.038	0.842	0.234	0.240	*ABC transporter ATP-binding/permease*	Defense Mechanisms	Unknown
RCAP_rcc00184	-0.264	0.298	-0.231	0.311	0.104	0.617	*outer membrane efflux protein*	Cell Envelope Biosynthesis	Cell Wall Biosynthesis
RCAP_rcc00185	-0.022	0.942	-0.060	0.811	-0.087	0.760	*hypothetical protein*	Unknown	Unknown
RCAP_rcc00186	0.185	0.501	-0.231	0.270	-0.046	0.916	*NUDIX superfamily hydrolase*	Unknown	Unknown
RCAP_rcc00187	-0.358	0.170	0.121	0.345	0.030	0.957	*hslO*	Post-translational Modification, Assembly and Chaperones	Unknown
RCAP_rcc00188	-1.104	0.000	-0.047	0.835	0.295	0.519	*NUDIX superfamily hydrolase*	Replication, Recombination and Repair	Unknown
RCAP_rcc00189	-0.610	0.008	0.103	0.633	-0.096	0.760	*cca*	Translation, ribosomal structure and biogenesis	Unknown
RCAP_rcc00190	0.046	0.885	-0.125	0.334	-0.072	0.651	*ABC transporter ATP-binding/permease*	Defense Mechanisms	Unknown
RCAP_rcc00191	-0.760	0.006	0.319	0.009	0.390	0.313	*tRNA (Uracil-5-)-methyltransferase*	Translation, ribosomal structure and biogenesis	Unknown
RCAP_rcc00192	0.332	0.047	0.545	0.000	-0.392	0.090	*ErfK/YbiS/YcfS/YnhG family protein*	Unknown	Unknown
RCAP_rcc00193	0.642	0.055	0.128	0.647	-0.214	0.579	*SCP-like extracellular protein*	Unknown	Unknown
RCAP_rcc00194	0.875	0.002	-0.494	0.114	-0.287	0.367	*ErfK/YbiS/YcfS/YnhG family protein/Tat domain-containing protein*	Unknown	Unknown
RCAP_rcc00195	0.422	0.016	0.672	0.000	-0.255	0.346	*hemH*	Metabolism of Cofactors, Coenzymes and Vitamins	Heme Biosynthesis
RCAP_rcc00196	0.015	0.949	0.103	0.551	-0.061	0.855	*hypothetical protein*	Unknown	Unknown
RCAP_rcc00197	-0.405	0.328	2.831	0.000	0.112	0.827	*comF*	Unknown	Unknown
RCAP_rcc00198	-0.351	0.134	0.393	0.005	0.134	0.713	*grxC*	Post-translational Modification, Assembly and Chaperones	Unknown
RCAP_rcc00199	-0.250	0.218	0.350	0.016	-0.083	0.757	*carbon-nitrogen family hydrolase*	Predicted Function	Nitrogen metabolism
RCAP_rcc00200	-0.250	0.268	0.123	0.592	-0.204	0.512	*MarR family transcriptional regulator*	Signal Transduction	Transcription Regulator
RCAP_rcc00201	0.526	0.038	0.118	0.570	-0.304	0.264	*adenine-specific DNA-methyltransferase*	Replication, Recombination and Repair	Unknown
RCAP_rcc00202	-0.166	0.541	-0.310	0.318	0.040	0.916	*rnhB*	Replication, Recombination and Repair	Unknown
RCAP_rcc00203	0.315	0.479	-1.058	0.009	0.005	0.991	*hypothetical protein*	Unknown	Unknown
RCAP_rcc00204	-0.103	0.749	-0.387	0.129	-0.076	0.873	*hypothetical protein*	Unknown	Unknown
RCAP_rcc00205	-0.104	0.793	-0.187	0.545	-0.173	0.539	*hypothetical protein*	Unknown	Unknown
RCAP_rcc00206	0.385	0.131	0.868	0.000	-0.356	0.248	*nspC*	Amino Acid Metabolism	Unknown
RCAP_rcc00207	0.218	0.310	0.530	0.000	-0.213	0.391	*lys1*	Amino Acid Metabolism	Unknown
RCAP_rcc00208	0.361	0.201	-0.025	0.915	-0.153	0.641	*argG*	Amino Acid Metabolism	Arginine and proline metabolism
RCAP_rcc00209	-0.106	0.652	-0.447	0.000	0.074	0.802	*mogA*	Unknown	Unknown
RCAP_rcc00210	0.533	0.009	0.137	0.564	-0.463	0.066	*ubiG*	Energy Metabolism	Biosynthesis of Ubiquinone
RCAP_rcc00211	-0.038	0.899	-0.191	0.200	-0.104	0.607	*pip1*	Amino Acid Metabolism	Arginine and proline metabolism
RCAP_rcc00212	0.108	0.652	0.411	0.072	-0.538	0.046	*hypothetical protein*	Unknown	Unknown
RCAP_rcc00213	0.296	0.136	0.145	0.380	-0.187	0.291	*nusA*	Transcription	Unknown
RCAP_rcc00214	-0.208	0.181	0.229	0.074	0.056	0.781	*hypothetical protein*	Unknown	Unknown
RCAP_rcc00215	-0.168	0.405	0.086	0.639	0.211	0.324	*infB*	Translation, ribosomal structure and biogenesis	Unknown
RCAP_rcc00216	0.468	0.091	2.016	0.000	-0.028	0.964	*hypothetical protein*	Unknown	Unknown
RCAP_rcc00217	-0.158	0.643	-0.597	0.002	0.243	0.354	*nudG*	Metabolism of Cofactors, Coenzymes and Vitamins	Folate biosynthesis
RCAP_rcc00218	0.208	NA	-0.515	0.000	0.124	0.429	*argJ*	Amino Acid Metabolism	Arginine and proline metabolism
RCAP_rcc00219	-0.037	0.903	-0.175	0.229	0.269	0.136	*PpiC-type peptidyl-prolyl cis-trans isomerase*	Post-translational Modification, Assembly and Chaperones	Unknown
RCAP_rcc00220	0.359	0.022	0.111	0.502	-0.194	0.477	*secA*	Trafficking and Secretion	Secretion
RCAP_rcc00221	-0.191	0.564	-0.356	0.055	0.161	0.701	*hypothetical protein*	Unknown	Unknown
RCAP_rcc00222	-0.075	0.732	1.175	0.000	-0.060	0.839	*radC*	Replication, Recombination and Repair	Repair
RCAP_rcc00223	0.194	0.262	0.264	0.201	-0.011	0.972	*dnaJ*	Post-translational Modification, Assembly and Chaperones	Unknown
RCAP_rcc00224	0.667	0.000	0.206	0.414	-0.254	0.463	*dnaK*	Post-translational Modification, Assembly and Chaperones	Unknown
RCAP_rcc00225	-0.018	0.958	-0.540	0.022	-0.077	0.869	*alkB*	Replication, Recombination and Repair	Unknown
RCAP_rcc00226	-0.576	0.055	-0.037	0.895	0.147	0.750	*mutY*	Replication, Recombination and Repair	Unknown
RCAP_rcc00227	0.454	0.019	-0.230	0.325	0.053	0.916	*hypothetical protein*	Unknown	Unknown
RCAP_rcc00228	0.799	0.006	-0.269	0.075	-0.249	0.516	*DSBA family oxidoreductase*	Post-translational Modification, Assembly and Chaperones	Unknown
RCAP_rcc00229	-0.621	0.022	-0.268	0.088	0.450	0.153	*lpxK*	Glycan Biosynthesis and Metabolism	Lipopolysaccharide biosynthesis

RCAP_rcc00230	-0.446	0.102	-0.522	0.019	0.503	0.222	*kdtA1*	Energy Metabolism	Puromycin biosynthesis
RCAP_rcc00231	0.767	0.000	-0.310	0.002	-0.243	0.516	*hypothetical protein*	Unknown	Unknown
RCAP_rcc00232	0.162	0.469	-0.401	0.022	0.027	0.948	*xylose isomerase-like TIM barrel family protein*	Carbohydrate Metabolism	Unknown
RCAP_rcc00233	0.066	0.785	0.626	0.000	-0.148	0.538	*AFG1-like ATPase*	Unknown	Unknown
RCAP_rcc00234	0.020	0.952	0.441	0.075	-0.791	0.009	*luciferase*	Energy Metabolism	Unknown
RCAP_rcc00235	-0.352	0.225	0.221	0.127	0.284	0.174	*folC*	Metabolism of Cofactors, Coenzymes and Vitamins	Folate biosynthesis
RCAP_rcc00236	0.603	0.010	0.091	0.719	-0.229	0.522	*accD*	Energy Metabolism	Reductive carboxylate cycle (CO2 fixation)
RCAP_rcc00237	-0.220	0.559	0.405	0.023	0.520	0.066	*abortive infection protein family*	Unknown	Unknown
RCAP_rcc00238	-0.680	0.032	-0.029	0.910	0.572	0.157	*hypothetical protein*	Unknown	Unknown
RCAP_rcc00239	-0.245	0.310	-0.383	0.023	0.279	0.267	*ansA*	Amino Acid Metabolism	Unknown
RCAP_rcc00240	0.035	0.861	0.131	NA	0.012	0.975	*hypothetical protein*	Unknown	Unknown
RCAP_rcc00241	0.031	0.918	0.339	0.041	-0.291	0.203	*radical SAM family protein*	Translation, ribosomal structure and biogenesis	Unknown
RCAP_rcc00242	0.170	0.373	0.402	0.005	-0.168	0.447	*hypothetical protein*	Unknown	Unknown
RCAP_rcc00243	-0.021	0.952	0.195	0.305	0.147	0.548	*dapD*	Amino Acid Metabolism	Lysine biosynthesis
RCAP_rcc00244	-0.729	0.044	-0.024	0.902	0.765	0.004	*hypothetical protein*	Unknown	Unknown
RCAP_rcc00245	-0.133	0.537	0.156	0.184	0.162	0.526	*hypothetical protein*	Unknown	Unknown
RCAP_rcc00246	-0.284	0.127	-0.087	0.541	0.146	0.365	*mltB*	Glycan Biosynthesis and Metabolism	Glycosaminoglycan degradation
RCAP_rcc00247	-0.128	0.670	-0.739	0.001	0.063	0.888	*NnrU family protein*	Energy Metabolism	Nitrogen Metabolism
RCAP_rcc00248	-0.476	0.198	-0.593	0.000	0.502	0.117	*hypothetical protein*	Unknown	Unknown
RCAP_rcc00249	0.153	0.604	0.490	0.000	0.081	0.656	*hup1*	Energy Metabolism	Aerobic/Anaerobic Respiration
RCAP_rcc00250	0.214	0.398	-0.220	0.137	-0.349	0.018	*amn*	Nucleotide Metabolism	Purine metabolism
RCAP_rcc00251	-0.210	0.416	-0.313	0.111	-0.187	0.253	*ade*	Nucleotide Metabolism	Purine metabolism
RCAP_rcc00252	-0.055	0.836	-0.404	0.005	0.021	0.964	*NAD-dependent epimerase/dehydratase*	Carbohydrate Metabolism	Unknown
RCAP_rcc00253	0.229	0.156	-1.217	0.000	-0.012	0.978	*major facilitator superfamily protein*	Carbohydrate Metabolism	Unknown
RCAP_rcc00254	-0.422	0.089	0.259	0.357	0.276	0.506	*hypothetical protein*	Unknown	Unknown
RCAP_rcc00255	-0.500	0.025	0.034	0.896	0.226	0.541	*polB*	Replication, Recombination and Repair	Replication
RCAP_rcc00256	-0.891	0.000	0.080	0.674	0.399	0.178	*lipoprotein*	Predicted Function	Unknown
RCAP_rcc00257	0.188	0.329	0.161	0.260	-0.062	NA	*leuS*	Translation, ribosomal structure and biogenesis	Aminoacyl-tRNA biosynthesis
RCAP_rcc00258	-0.032	0.875	-0.270	0.086	0.195	0.264	*lipoprotein*	Predicted Function	Unknown
RCAP_rcc00259	0.128	0.513	-0.295	0.041	0.002	0.995	*porin family protein*	Cell Envelope Biosynthesis	Cell Wall Biosynthesis
RCAP_rcc00260	-0.116	0.702	-0.192	0.313	0.227	0.479	*alanine racemase domain-containing protein*	Unknown	Unknown
RCAP_rcc00261	-0.877	0.000	-0.225	0.223	0.411	0.225	*hypothetical protein*	Unknown	Unknown
RCAP_rcc00262	0.080	0.767	-0.196	0.230	0.001	0.995	*ribA*	Metabolism of Cofactors, Coenzymes and Vitamins	Riboflavin metabolism
RCAP_rcc00263	0.574	0.022	-0.116	0.452	-0.877	0.000	*winged helix family two component transcriptional regulator*	Signal Transduction	Transcription Regulator
RCAP_rcc00264	-0.137	0.574	-0.405	0.113	-0.121	0.596	*hypothetical protein*	Unknown	Unknown
RCAP_rcc00265	0.036	0.908	0.215	0.305	-0.543	0.154	*RmuC domain-containing protein*	Unknown	Unknown
RCAP_rcc00266	-0.474	0.063	-0.140	0.356	0.271	0.467	*mutL*	Replication, Recombination and Repair	Repair
RCAP_rcc00267	0.240	0.466	-0.428	0.001	-0.015	0.964	*M16 family peptidase*	Post-translational Modification, Assembly and Chaperones	Peptidase
RCAP_rcc00268	0.495	0.071	-0.088	0.592	-0.297	0.224	*M16 family peptidase*	Post-translational Modification, Assembly and Chaperones	Peptidase
RCAP_rcc00269	-0.527	0.039	-0.082	0.511	0.241	0.457	*lipoprotein*	Predicted Function	Unknown
RCAP_rcc00270	-0.311	0.125	0.178	0.141	0.193	0.300	*lspA*	Cell Envelope Biosynthesis	Cell Wall Biosynthesis
RCAP_rcc00271	0.121	0.692	-0.010	0.969	0.045	0.839	*purH*	Metabolism of Cofactors, Coenzymes and Vitamins	One carbon pool by folate
RCAP_rcc00272	-0.096	0.638	-0.007	0.971	0.057	0.819	*heparinase II/III family protein*	Unknown	Unknown
RCAP_rcc00273	-0.216	0.520	0.276	0.081	0.560	0.034	*rsmB1*	Translation, ribosomal structure and biogenesis	Unknown
RCAP_rcc00274	0.436	0.074	-0.169	0.545	-0.267	0.403	*hypothetical protein*	Unknown	Unknown
RCAP_rcc00275	0.026	0.926	-0.115	0.591	0.020	0.965	*dapB*	Amino Acid Metabolism	Unknown
RCAP_rcc00276	0.325	0.146	0.432	0.000	-0.290	0.240	*rbfA*	Translation, ribosomal structure and biogenesis	Unknown
RCAP_rcc00277	-0.323	0.374	0.742	0.000	-0.023	0.967	*hypothetical protein*	Unknown	Unknown
RCAP_rcc00278	-0.412	0.289	0.720	0.000	0.228	0.526	*truB*	Nucleotide Metabolism	Pyrimidine metabolism
RCAP_rcc00279	-0.208	0.538	0.650	0.000	0.078	0.809	*hypothetical protein*	Unknown	Unknown
RCAP_rcc00280	0.329	0.078	-0.030	0.845	-0.736	0.000	*hemolysin-type calcium-binding repeat family protein*	Trafficking and Secretion	Secretion
RCAP_rcc00281	0.598	0.002	0.324	0.168	-0.377	0.234	*rpsO*	Translation, ribosomal structure and biogenesis	Unknown
RCAP_rcc00282	0.118	0.601	0.063	0.839	0.028	0.927	*aldehyde dehydrogenase*	Carbohydrate Metabolism	Glycolysis / Gluconeogenesis
RCAP_rcc00283	-0.921	0.012	-1.665	0.000	0.675	NA	*hypothetical protein*	Unknown	Unknown
RCAP_rcc00284	0.437	0.012	-0.423	0.004	-0.203	0.354	*pnp*	Nucleotide Metabolism	Pyrimidine metabolism
RCAP_rcc00285	0.546	0.000	0.175	0.294	-0.267	0.408	*secE*	Trafficking and Secretion	Secretion
RCAP_rcc00286	0.211	0.282	0.258	0.186	-0.199	0.453	*nusG*	Transcription	Unknown
RCAP_rcc00287	0.279	0.181	0.260	0.230	-0.140	0.545	*rplK*	Translation, ribosomal structure and biogenesis	Unknown
RCAP_rcc00288	0.202	0.325	0.236	0.223	-0.209	0.441	*rplA*	Translation, ribosomal structure and biogenesis	Unknown
RCAP_rcc00289	0.195	0.387	0.464	0.004	-0.187	0.491	*rplJ*	Translation, ribosomal structure and biogenesis	Unknown
RCAP_rcc00290	0.164	0.470	0.373	0.025	-0.014	0.961	*rplL*	Translation, ribosomal structure and biogenesis	Unknown
RCAP_rcc00291	0.266	0.163	0.149	0.479	-0.182	0.506	*rpoB*	Replication, Recombination and Repair	Replication
RCAP_rcc00292	0.196	0.363	0.059	0.794	-0.131	0.569	*rpoC*	Replication, Recombination and Repair	Replication
RCAP_rcc00293	0.741	0.001	-0.134	0.528	-0.150	0.706	*hypothetical protein*	Unknown	Unknown
RCAP_rcc00294	0.359	0.099	0.355	0.058	-0.221	0.497	*rpsL*	Translation, ribosomal structure and biogenesis	Unknown
RCAP_rcc00295	0.205	0.389	0.269	0.198	-0.126	0.532	*rpsG*	Translation, ribosomal structure and biogenesis	Unknown
RCAP_rcc00296	0.262	0.193	0.285	0.124	-0.197	0.504	*fusA1*	Translation, ribosomal structure and biogenesis	Unknown
RCAP_rcc00297	0.212	0.269	0.041	0.857	-0.139	0.608	*tuf2*	Translation, ribosomal structure and biogenesis	Unknown
RCAP_rcc00298	0.568	0.009	0.405	0.046	-0.311	0.352	*rpsJ*	Translation, ribosomal structure and biogenesis	Unknown
RCAP_rcc00299	-0.318	0.279	0.359	0.059	0.153	0.421	*rplC*	Translation, ribosomal structure and biogenesis	Unknown
RCAP_rcc00300	-0.056	0.834	0.257	0.159	-0.074	0.767	*rplD*	Translation, ribosomal structure and biogenesis	Unknown
RCAP_rcc00301	0.428	0.026	0.316	0.065	-0.282	0.363	*rplW*	Translation, ribosomal structure and biogenesis	Unknown
RCAP_rcc00302	0.287	0.160	0.512	0.004	-0.176	0.512	*rplB*	Translation, ribosomal structure and biogenesis	Unknown
RCAP_rcc00303	0.481	0.033	0.396	0.059	-0.238	0.493	*rpsS*	Translation, ribosomal structure and biogenesis	Unknown
RCAP_rcc00304	0.336	0.104	0.393	0.031	-0.264	0.390	*rplV*	Translation, ribosomal structure and biogenesis	Unknown
RCAP_rcc00305	0.185	0.312	0.396	0.039	-0.133	0.579	*rpsC*	Translation, ribosomal structure and biogenesis	Unknown
RCAP_rcc00306	-0.772	0.006	0.384	0.037	0.509	0.044	*rplP*	Translation, ribosomal structure and biogenesis	Unknown

RCAP_rcc00307	-0.659	0.043	-0.547	0.000	0.465	0.156	*hypothetical protein*	Unknown	Unknown
RCAP_rcc00308	0.551	0.018	0.407	0.016	-0.263	0.479	*rpmC*	Translation, ribosomal structure and biogenesis	Unknown
RCAP_rcc00309	0.569	0.018	0.351	0.099	-0.262	0.462	*rpsQ*	Translation, ribosomal structure and biogenesis	Unknown
RCAP_rcc00310	0.342	0.170	0.486	0.007	-0.198	0.519	*rplN*	Translation, ribosomal structure and biogenesis	Unknown
RCAP_rcc00311	-0.036	0.913	0.472	0.016	0.024	0.930	*rplX*	Translation, ribosomal structure and biogenesis	Unknown
RCAP_rcc00312	0.416	0.052	0.391	0.042	-0.243	0.439	*rplE*	Translation, ribosomal structure and biogenesis	Unknown
RCAP_rcc00313	0.511	0.017	0.506	0.005	-0.279	0.388	*rpsN*	Translation, ribosomal structure and biogenesis	Unknown
RCAP_rcc00314	0.449	0.030	0.496	0.011	-0.260	0.430	*rpsH*	Translation, ribosomal structure and biogenesis	Unknown
RCAP_rcc00315	0.288	0.172	0.526	0.008	-0.202	0.508	*rplF*	Translation, ribosomal structure and biogenesis	Unknown
RCAP_rcc00316	0.432	0.044	0.555	0.006	-0.277	0.358	*rplR*	Translation, ribosomal structure and biogenesis	Unknown
RCAP_rcc00317	-0.370	0.095	0.621	0.004	0.253	0.138	*rpsE*	Translation, ribosomal structure and biogenesis	Unknown
RCAP_rcc00318	0.026	0.915	0.612	0.006	-0.131	0.605	*rpmD*	Translation, ribosomal structure and biogenesis	Unknown
RCAP_rcc00319	0.335	0.277	-0.343	0.217	-0.303	0.267	*hypothetical protein*	Unknown	Unknown
RCAP_rcc00320	-0.019	0.962	0.537	0.207	-0.396	0.114	*hypothetical protein*	Unknown	Unknown
RCAP_rcc00321	0.393	0.129	0.242	0.335	-0.214	0.501	*rplO*	Translation, ribosomal structure and biogenesis	Unknown
RCAP_rcc00322	0.418	0.020	0.185	0.226	-0.168	0.568	*secY*	Trafficking and Secretion	Secretion
RCAP_rcc00323	-0.300	0.198	0.280	0.025	0.252	0.102	*adk*	Nucleotide Metabolism	Purine metabolism
RCAP_rcc00324	0.435	0.017	0.389	0.060	-0.199	0.506	*rpsM*	Translation, ribosomal structure and biogenesis	Unknown
RCAP_rcc00325	-0.175	0.557	0.257	0.248	0.182	0.271	*rpsK*	Translation, ribosomal structure and biogenesis	Unknown
RCAP_rcc00326	0.388	0.042	0.235	0.222	-0.218	0.442	*rpoA*	Replication, Recombination and Repair	Replication
RCAP_rcc00327	0.044	0.828	0.323	0.072	-0.121	0.683	*rplQ*	Translation, ribosomal structure and biogenesis	Unknown
RCAP_rcc00328	-0.095	0.749	-0.160	0.612	-0.104	0.649	*LuxR family autoinducer-binding transcriptional regulator*	Signal Transduction	Transcription Regulator
RCAP_rcc00329	-0.345	0.260	-0.167	0.629	-0.092	0.713	*autoinducer synthesis protein*	Unknown	Unknown
RCAP_rcc00330	-0.048	0.823	0.024	0.882	0.239	0.264	*ATPase AAA*	Replication, Recombination and Repair	Unknown
RCAP_rcc00331	0.156	0.498	0.439	0.003	-0.173	0.556	*crcB*	Cell Division	Chromosome Partitioning
RCAP_rcc00332	-0.106	0.527	0.236	0.046	0.036	0.888	*rluC*	Nucleotide Metabolism	Pyrimidine metabolism
RCAP_rcc00333	-0.553	0.010	0.051	0.777	0.165	0.579	*gph1*	Carbohydrate Metabolism	Glyoxylate and dicarboxylate metabolism
RCAP_rcc00334	-0.690	0.019	-0.077	0.661	0.687	0.029	*ATP12 chaperone protein family*	Post-translational Modification, Assembly and Chaperones	Unknown
RCAP_rcc00335	0.431	0.104	0.866	0.000	-0.156	0.487	*bztA*	Amino Acid Metabolism	Amino Acid Transport
RCAP_rcc00336	0.411	0.178	1.578	0.000	-0.556	0.035	*bztB*	Amino Acid Metabolism	Amino Acid Transport
RCAP_rcc00337	0.259	0.208	1.394	0.000	-0.121	0.704	*bztC*	Amino Acid Metabolism	Amino Acid Transport
RCAP_rcc00338	0.571	0.019	0.975	0.000	-0.354	0.186	*bztD*	Amino Acid Metabolism	Amino Acid Transport
RCAP_rcc00339	0.109	0.637	0.637	0.000	-0.055	0.828	*sixA*	Signal Transduction	Kinase/Phosphorelay
RCAP_rcc00340	-0.193	0.497	-0.010	0.966	-0.007	0.989	*hypothetical protein*	Unknown	Unknown
RCAP_rcc00341	0.566	0.016	-0.126	0.606	-0.257	0.429	*argB*	Amino Acid Metabolism	Arginine and proline metabolism
RCAP_rcc00342	-0.060	0.800	-0.015	0.931	-0.326	0.211	*short-chain dehydrogenase/reductase family oxidoreductase*	Unknown	Unknown
RCAP_rcc00343	-0.110	0.601	0.050	0.781	-0.248	0.155	*engB*	Unknown	Unknown
RCAP_rcc00344	-0.243	0.276	-0.279	0.024	0.029	0.948	*MOSC domain-containing protein*	Unknown	Unknown
RCAP_rcc00345	0.403	0.033	-0.039	0.830	-0.187	0.510	*oxaA*	Trafficking and Secretion	Trafficing
RCAP_rcc00346	-0.453	0.050	1.760	0.000	0.461	0.003	*diguanylate cyclase/phosphodiesterase*	Signal Transduction	Kinase/Phosphorelay
RCAP_rcc00347	-0.327	0.131	0.390	0.010	0.271	0.151	*PP-loop family ATPase*	Cell Division	Chromosome Partitioning
RCAP_rcc00348	-0.854	0.022	0.201	0.315	0.551	0.176	*hypothetical protein*	Unknown	Unknown
RCAP_rcc00349	-0.585	0.151	-0.198	0.516	0.562	0.168	*rnpA*	Translation, ribosomal structure and biogenesis	Unknown
RCAP_rcc00350	-0.332	0.422	0.003	0.993	0.262	0.454	*rpmH*	Translation, ribosomal structure and biogenesis	Unknown
RCAP_rcc00351	-0.036	0.901	0.031	0.896	-0.372	0.002	*sensor histidine kinase*	Signal Transduction	Kinase/Phosphorelay
RCAP_rcc00352	-0.664	0.077	0.556	0.016	0.046	0.944	*asnA*	Energy Metabolism	Nitrogen metabolism
RCAP_rcc00353	1.203	0.000	1.426	0.000	-1.020	0.000	*hypothetical protein*	Unknown	Unknown
RCAP_rcc00354	0.463	0.005	-0.029	0.911	0.020	0.945	*pepT*	Amino Acid Metabolism	Unknown
RCAP_rcc00355	-0.071	0.782	0.741	0.000	0.045	0.808	*S1 RNA binding domain-containing protein*	Transcription	Unknown
RCAP_rcc00356	-0.023	0.910	1.675	0.000	0.036	0.848	*cyclic nucleotide-binding domain-/cystathionine beta-synthase domain-/u*	Signal Transduction	Kinase/Phosphorelay
RCAP_rcc00357	-0.012	0.974	-0.476	0.001	-0.564	0.004	*macB*	Defense Mechanisms	Unknown
RCAP_rcc00358	0.295	0.296	-0.087	0.731	-0.527	0.178	*macA*	Defense Mechanisms	Unknown
RCAP_rcc00359	-0.189	0.617	0.834	0.001	0.080	0.857	*hypothetical protein*	Unknown	Unknown
RCAP_rcc00360	0.330	0.308	0.871	0.000	-0.048	0.831	*mipZ*	Cell Division	Chromosome Partitioning
RCAP_rcc00361	-0.080	0.784	0.187	0.341	-0.010	0.975	*rpmE*	Translation, ribosomal structure and biogenesis	Unknown
RCAP_rcc00362	0.010	0.972	0.333	0.041	-0.050	0.874	*rplS*	Translation, ribosomal structure and biogenesis	Unknown
RCAP_rcc00363	0.401	0.122	-0.140	0.440	-0.271	0.267	*hypothetical protein*	Unknown	Unknown
RCAP_rcc00364	-0.712	0.011	0.180	0.452	0.202	0.607	*trmD*	Translation, ribosomal structure and biogenesis	Unknown
RCAP_rcc00365	-0.321	0.125	0.648	0.000	-0.007	0.986	*hypothetical protein*	Unknown	Unknown
RCAP_rcc00366	-0.278	0.284	0.669	0.000	0.321	0.157	*rimM*	Translation, ribosomal structure and biogenesis	Unknown
RCAP_rcc00367	0.133	0.538	0.450	0.004	-0.064	0.696	*rpsP*	Translation, ribosomal structure and biogenesis	Unknown
RCAP_rcc00368	-0.385	0.047	0.373	0.098	-0.124	0.575	*aroH*	Amino Acid Metabolism	Phenylalanine, tyrosine and tryptophan biosynthesis
RCAP_rcc00369	-0.393	0.084	0.345	0.024	-0.009	0.977	*GNAT family acetyltransferase*	Cell Division	Chromosome Partitioning
RCAP_rcc00370	-0.238	0.520	0.280	0.186	0.010	0.968	*ffh*	Trafficking and Secretion	Trafficing
RCAP_rcc00371	0.089	0.763	0.350	0.181	-0.396	0.055	*LysR family transcriptional regulator*	Signal Transduction	Transcription Regulator
RCAP_rcc00372	-0.174	0.433	0.339	0.058	-0.036	0.899	*hypothetical protein*	Unknown	Unknown
RCAP_rcc00373	-0.285	0.156	0.047	0.778	0.068	0.754	*mdoH*	Cell Envelope Biosynthesis	Cell Wall Biosynthesis
RCAP_rcc00374	-0.411	0.156	-0.210	0.218	-0.026	0.963	*hypothetical protein*	Unknown	Unknown
RCAP_rcc00375	0.464	0.032	-0.010	0.964	-0.114	0.729	*mdoG1*	Metal and Ion Transport	Unknown
RCAP_rcc00376	0.593	0.001	0.321	0.003	-0.365	0.231	*divalent ion symporter*	Predicted Function	Replication
RCAP_rcc00377	-0.712	0.011	-0.352	0.116	0.080	0.736	*hemL*	Metabolism of Cofactors, Coenzymes and Vitamins	Heme Biosynthesis
RCAP_rcc00378	-0.364	0.243	-0.291	0.059	0.070	0.760	*glnA1*	Carbohydrate Metabolism	Glyoxylate and dicarboxylate metabolism
RCAP_rcc00379	0.314	0.373	-0.054	0.854	0.014	0.975	*dapA*	Amino Acid Metabolism	Unknown
RCAP_rcc00380	-0.260	0.374	-0.202	0.302	0.426	0.188	*transglycosylase, Slt family*	Glycan Biosynthesis and Metabolism	Glycosaminoglycan degradation
RCAP_rcc00381	0.174	0.347	-0.126	0.650	-0.032	0.908	*hypothetical protein*	Unknown	Unknown
RCAP_rcc00382	0.142	0.647	-0.134	0.711	-0.026	0.944	*hypothetical protein*	Unknown	Unknown
RCAP_rcc00383	-0.314	0.240	1.842	0.000	0.082	0.768	*FAD dependent oxidoreductase*	Energy Metabolism	Unknown

RCAP_rcc00384	0.465	0.005	0.589	0.000	-0.544	0.000	*gshA*	Metabolism of Other Amino Acids	Glutathione metabolism
RCAP_rcc00385	-0.456	0.109	-0.207	0.432	0.457	0.258	*hypothetical protein*	Unknown	Unknown
RCAP_rcc00386	0.553	0.029	-0.170	0.275	-0.224	0.241	*hypothetical protein*	Unknown	Unknown
RCAP_rcc00387	0.117	0.600	-0.074	0.701	0.180	0.392	*ubiA*	Photosynthesis	Biosynthesis of Ubiquinone
RCAP_rcc00388	-0.326	0.109	-0.055	0.647	0.328	0.066	*OmpA/MotB domain-containing protein*	Cell Envelope Biosynthesis	Cell Wall Biosynthesis
RCAP_rcc00389	-0.480	0.029	-0.383	0.008	0.500	0.064	*hypothetical protein*	Unknown	Unknown
RCAP_rcc00390	-0.146	0.557	-0.103	0.525	-0.035	0.953	*S49 family peptidase*	Post-translational Modification, Assembly and Chaperones	Peptidase
RCAP_rcc00391	-0.003	NA	-0.630	0.074	0.385	0.286	*hypothetical protein*	Unknown	Unknown
RCAP_rcc00392	0.398	0.314	-0.584	0.086	-0.044	0.946	*hypothetical protein*	Unknown	Unknown
RCAP_rcc00393	0.038	0.863	0.299	0.081	-0.156	0.427	*uvrC*	Replication, Recombination and Repair	Repair
RCAP_rcc00394	-0.329	0.390	0.168	0.485	0.238	0.553	*kamA*	Amino Acid Metabolism	Lysine degradation
RCAP_rcc00395	0.513	0.009	0.212	0.227	-0.211	0.512	*pgsA*	Lipid Metabolism	Glycerophospholipid metabolism
RCAP_rcc00396	-0.026	0.908	-0.170	0.349	-0.069	0.721	*hypothetical protein*	Unknown	Unknown
RCAP_rcc00397	-0.121	0.504	-0.316	0.034	0.173	0.477	*pyrC1*	Nucleotide Metabolism	Pyrimidine metabolism
RCAP_rcc00398	-0.441	0.055	-0.314	0.039	0.092	0.772	*hypothetical protein*	Unknown	Unknown
RCAP_rcc00399	-0.087	0.757	-0.446	0.005	0.097	0.708	*hypothetical protein*	Unknown	Unknown
RCAP_rcc00400	0.564	0.011	-0.015	0.955	-0.206	0.453	*pyrB*	Amino Acid Metabolism	Alanine, aspartate and glutamate metabolism
RCAP_rcc00401	-0.145	0.597	0.088	0.611	-0.093	0.750	*udgA*	Replication, Recombination and Repair	Phage Interaction
RCAP_rcc00402	-0.466	0.168	0.228	0.286	0.516	0.129	*hypothetical protein*	Unknown	Unknown
RCAP_rcc00403	0.745	0.023	-0.407	0.104	-0.652	0.084	*hypothetical protein*	Unknown	Unknown
RCAP_rcc00404	-0.604	0.066	-0.106	0.613	-0.051	0.937	*Sua5/YciO/YrdC/YwlC family protein*	Translation, ribosomal structure and biogenesis	Unknown
RCAP_rcc00405	-0.630	0.032	-0.610	0.065	-0.024	0.948	*acyl-CoA dehydrogenase domain-containing protein*	Lipid Metabolism	Unknown
RCAP_rcc00406	-0.827	0.016	0.362	0.027	0.515	0.162	*metallo-beta-lactamase*	Unknown	Unknown
RCAP_rcc00407	0.096	0.685	0.096	0.630	-0.069	0.769	*lipoprotein*	Predicted Function	Unknown
RCAP_rcc00408	0.000	0.999	-0.633	0.022	-0.067	0.894	*branched-chain amino acid transport family protein*	Unknown	Unknown
RCAP_rcc00409	-0.242	0.466	-1.125	0.000	0.229	0.511	*AzlC family protein*	Amino Acid Metabolism	Unknown
RCAP_rcc00410	-0.384	0.154	-0.070	0.809	0.264	0.432	*GNAT family acetyltransferase*	Cell Division	Chromosome Partitioning
RCAP_rcc00411	-0.111	0.762	-0.469	0.027	0.410	0.273	*S58 family peptidase*	Post-translational Modification, Assembly and Chaperones	Peptidase
RCAP_rcc00412	-0.400	0.065	-0.233	0.242	0.303	0.211	*arylformamidase*	Lipid Metabolism	Unknown
RCAP_rcc00413	0.318	0.043	-0.201	0.205	-0.036	0.899	*gabD*	Carbohydrate Metabolism	Butanoate metabolism
RCAP_rcc00414	-0.165	0.476	0.241	0.223	-0.013	0.974	*hypothetical protein*	Unknown	Unknown
RCAP_rcc00415	0.089	0.637	-0.391	0.000	0.241	0.313	*short-chain dehydrogenase/reductase family oxidoreductase*	Unknown	Unknown
RCAP_rcc00416	0.227	0.298	-0.522	0.000	-0.026	0.940	*LysR family transcriptional regulator*	Signal Transduction	Transcription Regulator
RCAP_rcc00417	-0.420	0.168	1.099	0.000	0.022	0.972	*hypothetical protein*	Unknown	Unknown
RCAP_rcc00418	0.079	0.795	-0.063	0.751	0.075	0.845	*TetR family transcriptional regulator*	Signal Transduction	Transcription Regulator
RCAP_rcc00419	0.139	0.549	0.313	0.077	-0.218	0.493	*NADH:flavin oxidoreductase/NADH oxidase*	Energy Metabolism	Unknown
RCAP_rcc00420	-0.408	0.251	0.263	0.309	-0.208	0.482	*pykA1*	Carbohydrate Metabolism	Glycolysis / Gluconeogenesis
RCAP_rcc00421	0.173	0.582	-0.117	0.578	-0.036	0.930	*lysC*	Amino Acid Metabolism	Lysine biosynthesis
RCAP_rcc00422	-0.219	0.174	-0.394	0.000	0.103	0.549	*ptsP*	Signal Transduction	Kinase/Phosphorelay
RCAP_rcc00423	1.304	0.000	-2.493	0.000	-0.011	0.984	*hypothetical protein*	Unknown	Unknown
RCAP_rcc00424	1.814	0.000	-1.960	0.000	0.047	0.930	*hypothetical protein*	Unknown	Unknown
RCAP_rcc00425	0.170	0.527	-0.131	0.501	0.360	0.140	*M48 family peptidase*	Post-translational Modification, Assembly and Chaperones	Peptidase
RCAP_rcc00426	0.218	0.514	0.003	0.994	-0.354	0.251	*hypothetical protein*	Unknown	Unknown
RCAP_rcc00427	-0.208	0.326	0.112	0.438	-0.052	0.787	*panC*	Metabolism of Cofactors, Coenzymes and Vitamins	Pantothenate and CoA biosynthesis
RCAP_rcc00428	-0.138	0.565	0.044	0.794	0.075	0.819	*panB*	Metabolism of Cofactors, Coenzymes and Vitamins	Pantothenate and CoA biosynthesis
RCAP_rcc00429	0.451	0.050	-0.015	0.944	-0.264	0.286	*hypothetical protein*	Unknown	Unknown
RCAP_rcc00430	0.077	0.811	-0.063	0.795	-0.031	0.914	*ErfK/YbiS/YcfS/YnhG family protein*	Unknown	Unknown
RCAP_rcc00431	-0.267	0.264	0.391	0.256	0.074	0.791	*creA*	Amino Acid Metabolism	Arginine and proline metabolism
RCAP_rcc00432	0.123	0.560	-0.178	0.086	-0.045	0.886	*mdtB*	Metal and Ion Transport	Unknown
RCAP_rcc00433	0.263	0.085	-0.117	0.471	-0.207	0.269	*RND family efflux transporter subunit MFP*	Defense Mechanisms	Unknown
RCAP_rcc00434	-0.036	0.946	0.593	0.256	-0.149	0.748	*hypothetical protein*	Unknown	Unknown
RCAP_rcc00435	3.202	0.000	-0.409	0.353	-0.411	0.162	*hypothetical protein*	Unknown	Unknown
RCAP_rcc00436	0.713	0.000	1.238	0.000	-0.046	0.903	*cytB*	Energy Metabolism	Aerobic/Anaerobic Respiration
RCAP_rcc00437	-0.548	0.119	0.048	0.811	0.588	0.122	*alpha/beta fold family hydrolase*	Unknown	Unknown
RCAP_rcc00438	0.530	0.014	0.218	0.201	-0.185	0.595	*glyA*	Carbohydrate Metabolism	Glyoxylate and dicarboxylate metabolism
RCAP_rcc00439	-0.767	0.002	-0.022	0.961	0.497	0.139	*ppnk*	Metabolism of Cofactors, Coenzymes and Vitamins	Nicotinate and nicotinamide metabolism
RCAP_rcc00440	-0.809	0.003	-0.443	0.037	0.598	0.118	*zraR*	Signal Transduction	Transcription Regulator
RCAP_rcc00441	-0.273	0.205	-0.260	0.234	0.272	0.120	*sensor histidine kinase/response regulator receiver protein*	Signal Transduction	Transcription Regulator
RCAP_rcc00442	-0.299	0.147	0.470	0.109	0.009	0.974	*prpE*	Energy Metabolism	Reductive carboxylate cycle (CO2 fixation)
RCAP_rcc00443	-0.339	0.124	0.238	0.325	0.398	0.006	*maeB1*	Energy Metabolism	Carbon fixation in photosynthetic organisms
RCAP_rcc00444	-0.235	0.381	-0.187	0.455	-0.018	0.964	*cdd*	Xenobiotics Biodegradation and Metabolism	Drug metabolism - other enzymes
RCAP_rcc00445	0.051	0.867	-0.396	0.004	0.012	0.969	*deoA*	Xenobiotics Biodegradation and Metabolism	Drug metabolism - other enzymes
RCAP_rcc00446	-0.080	0.746	-0.437	0.008	0.272	0.142	*deoB*	Carbohydrate Metabolism	Pentose phosphate pathway
RCAP_rcc00447	0.497	0.011	-0.507	0.000	-0.140	0.605	*upp*	Nucleotide Metabolism	Pyrimidine metabolism
RCAP_rcc00448	-0.692	0.012	-0.542	0.000	0.498	0.150	*sporulation domain-containing protein*	Unknown	Unknown
RCAP_rcc00449	-0.791	0.000	-0.445	0.001	0.375	0.300	*hypothetical protein*	Unknown	Unknown
RCAP_rcc00450	-0.220	0.286	0.108	0.729	0.111	0.661	*hypothetical protein*	Unknown	Unknown
RCAP_rcc00451	-0.523	0.063	-0.029	0.936	0.111	0.766	*hypothetical protein*	Unknown	Unknown
RCAP_rcc00452	-0.328	0.095	-0.382	0.034	0.268	0.339	*amino acid ABC transporter permease*	Amino Acid Metabolism	Amino Acid Transport
RCAP_rcc00453	0.510	0.001	-0.265	0.066	-0.059	0.835	*amino acid ABC transporter periplasmic amino acid-binding protein*	Unknown	Unknown
RCAP_rcc00454	-0.099	0.643	-0.377	0.049	-0.101	0.704	*moeB1*	Metabolism of Cofactors, Coenzymes and Vitamins	Unknown
RCAP_rcc00455	-0.195	0.423	-0.488	0.012	0.250	0.493	*dut*	Nucleotide Metabolism	Pyrimidine metabolism
RCAP_rcc00456	-0.669	0.001	0.051	0.721	0.035	0.949	*coaBC*	Metabolism of Cofactors, Coenzymes and Vitamins	Pantothenate and CoA biosynthesis
RCAP_rcc00457	0.188	0.414	0.322	0.141	-0.097	0.855	*hypothetical protein*	Unknown	Unknown
RCAP_rcc00458	0.802	0.000	0.806	0.000	-0.662	0.080	*rpoH1*	Replication, Recombination and Repair	Replication
RCAP_rcc00459	0.366	0.136	-0.532	0.042	-0.147	0.608	*glutathione S-transferase*	Sulfur Metabolism	Glutathione metabolism
RCAP_rcc00460	0.155	0.613	-0.377	0.048	-0.026	0.955	*comM*	Metal, Ion, Cofactor Transport	Magnesium Transport

RCAP_rcc00461	-0.752	0.010	-0.574	0.002	0.650	0.059	*est*	Lipid Metabolism	Unknown
RCAP_rcc00462	0.319	0.154	-0.027	0.866	-0.182	0.547	*gshB*	Sulfur Metabolism	Glutathione metabolism
RCAP_rcc00463	0.181	0.322	1.373	0.000	0.041	0.900	*hypothetical protein*	Unknown	Unknown
RCAP_rcc00464	-0.564	0.022	0.984	0.000	0.531	0.030	*tetrapyrrole methylase*	Unknown	Unknown
RCAP_rcc00465	-0.529	0.108	-0.638	0.000	0.543	0.034	*extracellular ligand-binding receptor family protein*	Unknown	Unknown
RCAP_rcc00466	-0.044	0.845	-0.289	0.034	0.005	0.985	*glnD*	Post-translational Modification, Assembly and Chaperones	Unknown
RCAP_rcc00467	-0.149	0.535	-0.422	0.005	0.112	0.589	*mviN*	Unknown	Unknown
RCAP_rcc00468	0.167	0.438	-0.145	0.278	-0.058	0.826	*rhomboid family protein*	Unknown	Unknown
RCAP_rcc00469	0.316	0.252	0.229	0.281	-0.131	0.703	*trpS*	Translation, ribosomal structure and biogenesis	Aminoacyl-tRNA biosynthesis
RCAP_rcc00470	-0.372	0.052	0.181	0.323	0.340	0.275	*MATE efflux family protein*	Defense Mechanisms	Unknown
RCAP_rcc00471	0.288	0.177	-0.134	0.485	0.209	0.388	*thioesterase superfamily protein*	Secondary metabolites biosynthesis, transport, and catabolism	Unknown
RCAP_rcc00472	-0.644	0.110	-0.178	0.592	0.423	0.316	*thioesterase superfamily protein*	Secondary metabolites biosynthesis, transport, and catabolism	Unknown
RCAP_rcc00473	-0.550	0.009	-0.525	0.031	0.223	0.482	*MerR family transcriptional regulator*	Signal Transduction	Transcription Regulator
RCAP_rcc00474	-0.026	0.920	-0.347	0.194	-0.122	0.579	*MerR family transcriptional regulator*	Signal Transduction	Transcription Regulator
RCAP_rcc00475	0.003	0.990	-0.283	0.146	0.022	0.945	*acyl-CoA dehydrogenase domain-containing protein*	Lipid Metabolism	Unknown
RCAP_rcc00476	0.329	0.101	0.167	0.135	-0.370	0.026	*hypothetical protein*	Unknown	Unknown
RCAP_rcc00477	-0.322	0.120	0.407	0.004	-0.029	0.947	*ribonuclease T2 family protein*	Translation, ribosomal structure and biogenesis	Unknown
RCAP_rcc00478	-0.085	0.685	-0.090	0.643	0.127	0.559	*alcohol dehydrogenase*	Energy Metabolism	Unknown
RCAP_rcc00479	0.180	0.291	0.003	0.992	-0.083	0.652	*COQ9 family ubiquinone biosynthesis protein*	Energy Metabolism	Biosynthesis of Ubiquinone
RCAP_rcc00480	0.654	0.015	-0.461	0.000	-0.227	0.522	*rpsU*	Translation, ribosomal structure and biogenesis	Unknown
RCAP_rcc00481	-0.613	0.009	1.548	0.000	0.388	0.096	*mcpI*	Motility	Chemotaxis
RCAP_rcc00482	-0.421	0.258	1.916	0.000	0.170	0.686	*hypothetical protein*	Unknown	Unknown
RCAP_rcc00483	0.878	0.000	0.061	0.837	-0.371	0.188	*AsnC/Lrp family transcriptional regulator*	Signal Transduction	Transcription Regulator
RCAP_rcc00484	0.452	0.053	-1.873	0.000	0.264	0.137	*ald*	Metabolism of Other Amino Acids	Taurine and hypotaurine metabolism
RCAP_rcc00485	0.451	0.091	-0.622	0.000	-0.085	NA	*gst*	Sulfur Metabolism	Glutathione metabolism
RCAP_rcc00486	-0.215	0.432	1.529	0.000	0.126	0.622	*hypothetical protein*	Unknown	Unknown
RCAP_rcc00487	0.045	0.888	0.406	0.000	-0.026	0.944	*suhB*	Signal Transduction	Transcription Regulator
RCAP_rcc00488	0.239	0.377	-0.522	0.001	-0.029	0.948	*fsr*	Metal and Ion Transport	Unknown
RCAP_rcc00489	-0.621	0.036	0.324	0.366	-0.089	0.797	*LysR family transcriptional regulator*	Signal Transduction	Transcription Regulator
RCAP_rcc00490	-0.321	0.344	0.460	0.498	0.007	0.991	*metF*	Energy Metabolism	Reductive carboxylate cycle (CO2 fixation)
RCAP_rcc00491	-0.619	0.059	-0.195	0.623	0.209	0.633	*thioesterase superfamily protein*	Unknown	Unknown
RCAP_rcc00492	0.012	0.965	0.047	0.804	-0.272	0.549	*hypothetical protein*	Unknown	Unknown
RCAP_rcc00493	0.106	0.790	0.853	0.000	-0.299	0.034	*ldc*	Metabolism of Other Amino Acids	Glutathione metabolism
RCAP_rcc00494	0.205	0.494	0.379	0.122	0.066	0.875	*AsnC/Lrp family transcriptional regulator*	Signal Transduction	Transcription Regulator
RCAP_rcc00495	-0.187	0.443	-0.198	0.257	0.185	0.479	*glyoxalase/bleomycin resistance protein/dioxygenase*	Unknown	Unknown
RCAP_rcc00496	0.340	0.086	0.004	0.980	-0.223	0.453	*valS*	Translation, ribosomal structure and biogenesis	Aminoacyl-tRNA biosynthesis
RCAP_rcc00497	-0.430	0.044	-0.194	0.208	0.033	0.927	*UbiA prenyltransferase*	Photosynthesis	Biosynthesis of Ubiquinone
RCAP_rcc00498	-0.357	0.154	-0.282	0.153	-0.171	0.519	*transglycosylase, Slt family*	Glycan Biosynthesis and Metabolism	Glycosaminoglycan degradation
RCAP_rcc00499	-0.698	0.040	-0.591	0.000	0.638	0.050	*hypothetical protein*	Unknown	Unknown
RCAP_rcc00500	-0.483	0.035	-0.070	0.759	0.031	0.923	*hypothetical protein*	Unknown	Unknown
RCAP_rcc00501	-0.213	0.251	-0.168	0.240	0.134	0.529	*cpaB*	Trafficking and Secretion	Trafficing
RCAP_rcc00502	-0.025	0.900	-0.096	0.364	-0.060	0.716	*type II and III secretion system protein*	Trafficking and Secretion	Secretion
RCAP_rcc00503	-0.407	0.094	-0.135	0.487	0.186	0.395	*OmpA/MotB domain-containing protein*	Cell Envelope Biosynthesis	Cell Wall Biosynthesis
RCAP_rcc00504	0.431	0.069	-0.048	0.795	-0.340	0.133	*PP-loop family ATPase*	Trafficking and Secretion	Trafficing
RCAP_rcc00505	0.228	0.395	-0.243	NA	-0.116	0.661	*type II secretion system protein E*	Trafficking and Secretion	Secretion
RCAP_rcc00506	0.019	0.951	-0.192	0.408	0.121	0.530	*type II secretion system protein*	Trafficking and Secretion	Secretion
RCAP_rcc00507	-0.026	0.920	-0.231	0.214	0.109	0.572	*type II secretion system protein*	Trafficking and Secretion	Secretion
RCAP_rcc00508	0.165	0.451	0.091	0.629	-0.175	0.531	*hypothetical protein*	Unknown	Unknown
RCAP_rcc00509	-0.002	0.995	-0.010	0.961	-0.200	0.251	*hypothetical protein*	Unknown	Unknown
RCAP_rcc00510	-0.513	0.055	-0.269	0.060	0.380	0.095	*A24 family peptidase*	Post-translational Modification, Assembly and Chaperones	Peptidase
RCAP_rcc00511	-0.308	0.249	-0.580	0.002	0.481	0.144	*ispE*	Lipid	Terpenoid backbone biosynthesis
RCAP_rcc00512	0.081	0.754	-0.256	0.058	0.048	0.805	*hypothetical protein*	Unknown	Unknown
RCAP_rcc00513	0.242	0.144	-0.621	0.000	-0.088	0.651	*etfD*	Energy Metabolism	Aerobic/Anaerobic Respiration
RCAP_rcc00514	0.720	0.000	0.467	0.002	-0.259	0.446	*greA*	Transcription	Unknown
RCAP_rcc00515	-0.316	0.258	0.199	0.273	0.329	0.178	*hypothetical protein*	Unknown	Unknown
RCAP_rcc00516	0.488	0.007	3.067	0.000	-0.158	0.601	*tpl*	Amino Acid Metabolism	Tryptophan metabolism
RCAP_rcc00517	0.086	0.755	0.177	0.601	-0.158	0.625	*hypothetical protein*	Unknown	Unknown
RCAP_rcc00518	-0.380	0.207	-0.479	0.003	0.019	0.951	*fadA*	Lipid Metabolism	Fatty acid metabolism
RCAP_rcc00519	-0.797	0.016	-0.444	0.042	0.378	0.058	*cupin domain-containing protein*	Unknown	Unknown
RCAP_rcc00520	-0.596	0.009	-0.479	0.001	0.062	0.753	*fadB*	Lipid Metabolism	Fatty acid metabolism
RCAP_rcc00521	0.042	0.834	-0.065	0.685	0.040	0.874	*dcp*	Amino Acid Metabolism	Unknown
RCAP_rcc00522	-0.472	0.099	0.417	0.007	0.427	0.127	*fieF*	Metal and Ion Transport	Unknown
RCAP_rcc00523	-0.289	0.299	0.090	0.592	0.305	0.361	*hypothetical protein*	Unknown	Unknown
RCAP_rcc00524	0.359	0.033	-0.071	0.702	-0.042	0.860	*gyaR1*	Carbohydrate Metabolism	Glyoxylate and dicarboxylate metabolism
RCAP_rcc00525	-0.512	0.109	0.088	0.758	0.314	0.170	*hypothetical protein*	Unknown	Unknown
RCAP_rcc00526	0.085	0.685	-0.696	0.000	-0.013	0.969	*dimethylglycine dehydrogenase*	Amino Acid Metabolism	Unknown
RCAP_rcc00527	0.110	0.647	-0.177	0.305	0.041	0.887	*family 25 glycosyl hydrolase*	Cell Envelope Biosynthesis	Cell Wall Biosynthesis
RCAP_rcc00528	-0.108	0.735	0.245	0.189	-0.122	0.713	*carboxymuconolactone decarboxylase*	Xenobiotics Biodegradation and Metabolism	Benzoate degradation via hydroxylation
RCAP_rcc00529	-0.393	0.041	0.178	0.400	0.332	0.267	*FAD dependent oxidoreductase*	Energy Metabolism	Unknown
RCAP_rcc00530	0.269	0.345	0.670	0.001	-0.145	0.579	*hypothetical protein*	Unknown	Unknown
RCAP_rcc00531	0.072	0.773	0.376	0.006	-0.076	0.775	*carB*	Amino Acid Metabolism	Alanine, aspartate and glutamate metabolism
RCAP_rcc00532	0.335	0.099	0.173	0.194	-0.135	0.602	*aspS*	Translation, ribosomal structure and biogenesis	Aminoacyl-tRNA biosynthesis
RCAP_rcc00533	-0.187	0.474	0.167	0.305	0.018	0.968	*hypothetical protein*	Unknown	Unknown
RCAP_rcc00534	-0.752	0.019	-0.351	0.082	0.540	0.179	*hypothetical protein*	Unknown	Unknown
RCAP_rcc00535	-0.367	0.336	0.030	0.924	0.658	0.093	*hypothetical protein*	Unknown	Unknown
RCAP_rcc00536	-0.136	0.490	1.091	0.000	0.186	0.350	*GNAT family acetyltransferase*	Cell Division	Chromosome Partitioning
RCAP_rcc00537	-0.869	0.003	3.294	0.000	0.723	0.032	*response regulator receiver protein*	Signal Transduction	Transcription Regulator

RCAP_rcc00538	0.640	0.001	0.115	0.573	-0.274	0.295	*methylmalonyl-CoA epimerase*	Carbohydrate Metabolism	Glyoxylate and dicarboxylate metabolism
RCAP_rcc00539	0.270	0.320	-0.564	0.000	-0.081	0.738	*hypothetical protein*	Unknown	Unknown
RCAP_rcc00540	-0.512	0.059	-0.740	0.000	0.438	0.288	*nitroreductase*	Energy Metabolism	Unknown
RCAP_rcc00541	-0.592	0.040	0.275	0.156	0.396	0.237	*hypothetical protein*	Unknown	Unknown
RCAP_rcc00542	0.432	0.039	1.776	0.000	-0.328	0.295	*hypothetical protein*	Unknown	Unknown
RCAP_rcc00543	0.654	0.003	0.107	0.760	-0.210	0.588	*hypothetical protein*	Unknown	Unknown
RCAP_rcc00544	-0.364	0.073	-0.440	0.057	0.248	0.511	*aldehyde dehydrogenase*	Carbohydrate Metabolism	Glycolysis / Gluconeogenesis
RCAP_rcc00545	-0.155	0.489	-0.190	0.211	0.180	0.547	*deoC*	Carbohydrate Metabolism	Pentose phosphate pathway
RCAP_rcc00546	-0.717	0.028	0.273	0.296	0.446	0.171	*hypothetical protein*	Unknown	Unknown
RCAP_rcc00547	0.635	0.007	-0.356	0.002	-0.260	0.466	*glutaredoxin family protein*	Post-translational Modification, Assembly and Chaperones	Unknown
RCAP_rcc00548	-0.012	0.956	-0.137	0.328	-0.259	0.215	*BolA family protein*	Signal Transduction	Kinase/Phosphorelay
RCAP_rcc00549	0.064	0.816	-0.150	0.251	0.047	0.780	*purL*	Nucleotide Metabolism	Purine metabolism
RCAP_rcc00550	-0.287	0.444	1.778	0.000	0.099	0.736	*LysR family transcriptional regulator*	Signal Transduction	Transcription Regulator
RCAP_rcc00551	0.280	0.139	-0.660	0.000	-0.138	0.512	*murI*	Metabolism of Other Amino Acids	D-Glutamine and D-glutamate metabolism
RCAP_rcc00552	0.153	0.355	-0.170	0.173	-0.160	0.539	*hypothetical protein*	Unknown	Unknown
RCAP_rcc00553	0.526	0.004	0.137	0.313	-0.125	0.696	*argC*	Amino Acid Metabolism	Arginine and proline metabolism
RCAP_rcc00554	0.335	0.185	-0.379	0.001	-0.050	0.869	*ccmE*	Energy Metabolism	Cytochrome Biogenesis
RCAP_rcc00555	0.226	0.513	1.065	0.000	-0.234	0.549	*hypothetical protein*	Unknown	Unknown
RCAP_rcc00556	-0.830	0.007	0.266	0.138	0.398	0.270	*hypothetical protein*	Unknown	Unknown
RCAP_rcc00557	0.224	0.377	0.024	0.886	-0.137	0.605	*metG*	Translation, ribosomal structure and biogenesis	Aminoacyl-tRNA biosynthesis
RCAP_rcc00558	-0.353	0.047	0.253	0.191	0.272	0.070	*diguanylate cyclase/phosphodiesterase*	Signal Transduction	Kinase/Phosphorelay
RCAP_rcc00559	-0.419	0.084	0.289	0.059	0.233	0.332	*pmtA*	Lipid Metabolism	Glycerophospholipid metabolism
RCAP_rcc00560	-0.192	0.520	-0.192	0.411	-0.049	0.919	*mopB*	Signal Transduction	Transcription Regulator
RCAP_rcc00561	0.728	NA	-0.632	0.000	0.704	0.046	*mopA*	Signal Transduction	Transcription Regulator
RCAP_rcc00562	1.387	NA	-1.048	0.000	0.306	0.288	*modA1*	Metal and Ion Transport	Unknown
RCAP_rcc00563	1.124	0.000	-0.501	0.000	0.617	0.069	*modB1*	Metal and Ion Transport	Unknown
RCAP_rcc00564	0.504	0.009	-0.220	0.166	0.768	0.004	*modC1*	Metal and Ion Transport	Unknown
RCAP_rcc00565	-0.394	0.307	-0.702	0.001	0.714	0.060	*modD*	Metabolism of Cofactors, Coenzymes and Vitamins	Unknown
RCAP_rcc00566	-0.066	0.835	-0.131	0.531	0.152	0.673	*nifB1*	Energy Metabolism	Nitrogen metabolism
RCAP_rcc00567	-0.249	0.316	0.830	0.000	-0.533	0.170	*nifA1*	Signal Transduction	Transcription Regulator
RCAP_rcc00568	-0.027	0.948	0.996	0.001	-0.183	0.637	*rpoN*	Replication, Recombination and Repair	Replication
RCAP_rcc00569	-0.193	NA	0.448	0.501	-0.209	NA	*nifU1*	Energy Metabolism	Nitrogen metabolism
RCAP_rcc00570	0.447	0.064	-0.018	0.949	-0.500	0.042	*nifK*	Energy Metabolism	Nitrogen metabolism
RCAP_rcc00571	0.295	0.245	0.163	0.535	-0.511	0.043	*nifD*	Energy Metabolism	Nitrogen metabolism
RCAP_rcc00572	0.614	0.047	0.166	0.598	-0.712	0.003	*nifH1*	Energy Metabolism	Nitrogen metabolism
RCAP_rcc00573	0.334	0.396	0.125	0.807	0.128	0.794	*fdxD*	Energy Metabolism	Aerobic/Anaerobic Respiration
RCAP_rcc00574	-0.729	0.069	-0.961	0.002	0.111	0.809	*Crp/Fnr family transcriptional regulator*	Signal Transduction	Transcription Regulator
RCAP_rcc00575	0.517	0.020	-1.031	0.000	-0.370	0.251	*hypothetical protein*	Unknown	Unknown
RCAP_rcc00576	-0.690	0.002	-1.506	0.000	0.391	0.224	*cbbO*	Energy Metabolism	Carbon fixation in photosynthetic organisms
RCAP_rcc00577	-0.390	0.029	-1.431	0.000	0.175	0.578	*cbbQ*	Energy Metabolism	Carbon fixation in photosynthetic organisms
RCAP_rcc00578	0.524	0.023	-1.479	0.000	-0.053	0.919	*cbbS*	Energy Metabolism	Carbon fixation in photosynthetic organisms
RCAP_rcc00579	0.406	0.024	-1.334	0.000	-0.052	0.902	*cbbL*	Energy Metabolism	Carbon fixation in photosynthetic organisms
RCAP_rcc00580	-0.103	0.775	0.290	0.205	-0.197	0.651	*cbbR1*	Signal Transduction	Transcription Regulator
RCAP_rcc00581	0.335	0.167	-0.256	0.208	-0.244	0.448	*hypothetical protein*	Unknown	Unknown
RCAP_rcc00582	-0.323	0.349	-0.221	0.401	-0.089	0.844	*hypothetical protein*	Unknown	Unknown
RCAP_rcc00583	-0.415	0.299	-0.079	0.795	0.095	0.859	*TetR family transcriptional regulator*	Signal Transduction	Transcription Regulator
RCAP_rcc00584	2.996	0.000	-0.613	0.000	0.789	0.036	*anfA*	Signal Transduction	Transcription Regulator
RCAP_rcc00585	0.786	0.011	0.700	0.030	-0.006	0.991	*anfH*	Energy Metabolism	Nitrogen metabolism
RCAP_rcc00586	0.311	0.296	0.334	0.393	-0.400	0.192	*anfD*	Energy Metabolism	Nitrogen metabolism
RCAP_rcc00587	0.387	0.356	-0.230	0.719	-0.319	0.479	*anfG*	Energy Metabolism	Nitrogen metabolism
RCAP_rcc00588	0.619	0.044	0.245	0.402	-0.387	0.258	*anfK*	Energy Metabolism	Nitrogen metabolism
RCAP_rcc00589	-0.308	NA	-0.288	0.612	0.180	NA	*anfO*	Energy Metabolism	Nitrogen metabolism
RCAP_rcc00590	0.022	0.967	-0.320	0.460	0.200	NA	*hypothetical protein*	Unknown	Unknown
RCAP_rcc00591	-0.213	0.520	0.222	0.387	-0.275	0.482	*flavin-nucleotide-binding protein*	Unknown	Unknown
RCAP_rcc00592	-0.614	0.029	-0.294	0.106	0.219	0.626	*hyi*	Carbohydrate Metabolism	Glyoxylate and dicarboxylate metabolism
RCAP_rcc00593	-0.161	0.580	-0.114	0.479	0.292	0.327	*hypothetical protein*	Unknown	Unknown
RCAP_rcc00594	0.146	0.559	-0.376	0.016	0.059	0.802	*MoxR family ATPase*	Unknown	Unknown
RCAP_rcc00595	-0.556	0.172	-0.637	0.031	0.711	0.045	*hypothetical protein*	Unknown	Unknown
RCAP_rcc00596	-0.498	0.010	-0.403	0.012	0.480	0.076	*hypothetical protein*	Unknown	Unknown
RCAP_rcc00597	0.005	0.990	-0.611	0.000	0.430	0.140	*hypothetical protein*	Unknown	Unknown
RCAP_rcc00598	-0.711	0.072	-0.375	0.145	0.212	0.641	*M20 family peptidase*	Post-translational Modification, Assembly and Chaperones	Peptidase
RCAP_rcc00599	0.045	0.893	0.161	0.517	-0.034	0.946	*hypothetical protein*	Unknown	Unknown
RCAP_rcc00600	0.280	0.143	0.422	0.019	-0.167	0.371	*nrdJ1*	Metabolism of Other Amino Acids	Glutathione metabolism
RCAP_rcc00601	-0.237	0.344	-0.324	0.126	0.084	0.782	*mntH*	Metal and Ion Transport	Unknown
RCAP_rcc00602	0.145	0.589	-0.131	0.566	-0.541	0.001	*mntR*	Signal Transduction	Transcription Regulator
RCAP_rcc00603	-0.215	0.526	0.199	0.305	-0.266	0.504	*alpha/beta fold family hydrolase*	Lipid Metabolism	Glycerophospholipid metabolism
RCAP_rcc00604	0.323	0.402	0.103	0.740	-0.755	0.000	*TerC family integral membrane protein*	Metal and Ion Transport	Unknown
RCAP_rcc00605	-0.028	0.925	0.080	0.685	-0.108	0.589	*kup*	Metal and Ion Transport	Unknown
RCAP_rcc00606	0.877	0.001	0.188	0.521	-0.218	0.582	*lipoprotein*	Predicted Function	Unknown
RCAP_rcc00607	-0.825	0.002	-0.544	0.457	0.185	0.700	*cspA1*	Unknown	Unknown
RCAP_rcc00608	-2.033	0.000	-0.492	0.493	0.285	NA	*hypothetical protein*	Unknown	Unknown
RCAP_rcc00609	-1.632	0.000	-0.558	0.446	0.396	NA	*hypothetical protein*	Unknown	Unknown
RCAP_rcc00610	-0.667	NA	-0.131	0.872	0.191	0.565	*hypothetical protein*	Unknown	Unknown
RCAP_rcc00611	-0.122	0.635	-0.052	0.849	0.095	0.839	*nikR*	Signal Transduction	Transcription Regulator
RCAP_rcc00612	-0.695	0.028	0.232	0.264	-0.007	0.991	*hypothetical protein*	Unknown	Unknown
RCAP_rcc00613	0.158	0.433	0.489	0.000	-0.548	0.053	*TetR family transcriptional regulator*	Signal Transduction	Transcription Regulator
RCAP_rcc00614	-0.430	0.195	1.379	0.000	0.177	0.632	*RND efflux system outer membrane lipoprotein*	Cell Envelope Biosynthesis	Cell Wall Biosynthesis

RCAP_rcc00615	-0.597	0.004	1.072	0.000	0.329	0.285	*acrA*	Defense Mechanisms	Unknown
RCAP_rcc00616	-0.253	0.215	1.051	0.000	0.051	0.839	*acrB*	Metal and Ion Transport	Unknown
RCAP_rcc00617	-0.298	0.154	-0.649	0.000	0.281	0.354	*hemolysin D*	Trafficking and Secretion	Secretion
RCAP_rcc00618	-0.369	0.070	-0.897	0.000	0.091	0.738	*ABC transporter ATP-binding/permease*	Defense Mechanisms	Unknown
RCAP_rcc00619	-0.076	0.778	-0.809	0.000	0.074	0.760	*ABC transporter permease*	Defense Mechanisms	Unknown
RCAP_rcc00620	-0.704	0.005	3.434	0.000	0.696	0.001	*response regulator receiver modulated diguanylate cyclase/phosphodieste*	Signal Transduction	Transcription Regulator
RCAP_rcc00621	-0.825	0.008	1.959	0.000	0.653	0.064	*signal transduction histidine kinase*	Signal Transduction	Kinase/Phosphorelay
RCAP_rcc00622	-0.570	0.054	-1.098	0.000	0.783	0.023	*moeA*	Metabolism of Cofactors, Coenzymes and Vitamins	Unknown
RCAP_rcc00623	-0.293	0.260	0.205	0.316	0.169	0.612	*RpiR family transcriptional regulator*	Signal Transduction	Transcription Regulator
RCAP_rcc00624	0.349	0.159	0.487	0.012	-0.018	0.965	*amino acid ABC transporter periplasmic amino acid-binding protein*	Amino Acid Metabolism	Amino Acid Transport
RCAP_rcc00625	0.006	0.984	0.244	0.308	0.103	0.692	*amino acid ABC transporter permease*	Amino Acid Metabolism	Amino Acid Transport
RCAP_rcc00626	0.040	0.890	-0.170	0.459	-0.160	0.632	*amino acid ABC transporter permease*	Amino Acid Metabolism	Amino Acid Transport
RCAP_rcc00627	-0.039	0.879	-0.106	0.532	-0.144	0.516	*amino acid ABC transporter ATP-binding protein*	Amino Acid Metabolism	Amino Acid Transport
RCAP_rcc00628	-0.151	0.478	-0.002	0.993	0.070	0.779	*menC*	Secondary metabolites biosynthesis, transport, and catabolism	Unknown
RCAP_rcc00629	-0.748	0.020	-0.044	0.856	0.478	0.228	*GNAT family acetyltransferase*	Cell Division	Chromosome Partitioning
RCAP_rcc00630	-0.448	0.147	3.545	0.000	0.235	0.260	*ice nucleation protein repeat family protein*	Unknown	Unknown
RCAP_rcc00631	-0.085	0.836	-0.768	0.017	-0.278	0.458	*hypothetical protein*	Unknown	Unknown
RCAP_rcc00632	0.106	0.793	-0.928	0.001	-0.016	0.979	*hypothetical protein*	Unknown	Unknown
RCAP_rcc00633	-0.450	0.285	-1.119	0.000	0.706	0.055	*ABC transporter permease*	Defense Mechanisms	Unknown
RCAP_rcc00634	-0.501	NA	-0.880	0.019	0.455	NA	*ABC transporter ATP-binding protein*	Defense Mechanisms	Unknown
RCAP_rcc00635	-0.266	0.296	-1.062	0.000	0.409	0.140	*von Willebrand factor type A domain-containing protein*	Signal Transduction	Transcription Regulator
RCAP_rcc00636	-0.594	NA	-0.887	0.010	0.444	0.274	*hypothetical protein*	Unknown	Unknown
RCAP_rcc00637	-0.413	0.083	-0.997	0.000	0.273	0.389	*hypothetical protein*	Unknown	Unknown
RCAP_rcc00638	-0.248	0.275	-0.968	0.000	0.269	0.200	*srfB*	Unknown	Unknown
RCAP_rcc00639	-0.145	0.747	-0.937	0.005	0.159	0.731	*hypothetical protein*	Unknown	Unknown
RCAP_rcc00640	-0.392	0.369	-1.321	0.000	0.652	0.095	*S1/S6 family peptidase*	Post-translational Modification, Assembly and Chaperones	Peptidase
RCAP_rcc00641	-0.069	0.861	-0.592	0.010	0.270	0.388	*hypothetical protein*	Unknown	Unknown
RCAP_rcc00642	-0.631	0.012	-1.189	0.000	0.516	0.044	*peptidoglycan binding domain-containing protein*	Unknown	Unknown
RCAP_rcc00643	-0.337	0.120	-1.691	0.000	0.292	0.259	*diguanylate cyclase/phosphodiesterase*	Signal Transduction	Kinase/Phosphorelay
RCAP_rcc00644	-0.775	0.005	2.206	0.000	0.602	0.047	*mcpX*	Motility	Chemotaxis
RCAP_rcc00645	-0.611	0.004	0.218	0.228	0.739	0.000	*diguanylate cyclase/phosphodiesterase*	Signal Transduction	Kinase/Phosphorelay
RCAP_rcc00646	0.172	0.524	0.139	0.539	0.210	0.574	*GNAT family acetyltransferase*	Cell Division	Chromosome Partitioning
RCAP_rcc00647	-0.551	0.070	-0.360	0.088	0.384	0.316	*kdtA2*	Energy Metabolism	Puromycin biosynthesis
RCAP_rcc00648	-0.093	0.774	-0.306	0.263	0.385	0.259	*kdtA3*	Energy Metabolism	Puromycin biosynthesis
RCAP_rcc00649	-0.911	0.002	-0.771	0.000	0.595	0.059	*dgkA*	Signal Transduction	Transcription Regulator
RCAP_rcc00650	0.175	0.503	-0.911	0.001	-0.086	0.697	*sulfatase*	Unknown	Unknown
RCAP_rcc00651	-0.109	0.789	-0.857	0.000	0.605	0.124	*winged helix family two component transcriptional regulator*	Signal Transduction	Transcription Regulator
RCAP_rcc00652	-0.495	0.137	-0.694	0.000	0.412	0.266	*sensor histidine kinase*	Signal Transduction	Kinase/Phosphorelay
RCAP_rcc00653	0.255	0.268	0.157	0.369	-0.275	0.195	*ABC transporter*	Unknown	Unknown
RCAP_rcc00654	-0.259	0.381	-0.736	0.006	0.377	0.316	*hypothetical protein*	Unknown	Unknown
RCAP_rcc00655	-0.450	0.076	-0.719	0.011	0.109	0.705	*hypothetical protein*	Unknown	Unknown
RCAP_rcc00656	0.260	0.196	0.462	0.031	-0.171	0.596	*hypothetical protein*	Unknown	Unknown
RCAP_rcc00657	-0.318	0.181	0.002	0.993	0.442	0.048	*hypothetical protein*	Unknown	Unknown
RCAP_rcc00658	-0.647	0.004	0.339	0.097	0.251	0.231	*hypothetical protein*	Unknown	Unknown
RCAP_rcc00659	0.415	0.030	0.283	0.122	-0.155	0.622	*puhA*	Photosynthesis	Light Harvesting Machinery
RCAP_rcc00660	0.064	0.734	0.307	0.072	-0.015	0.960	*pucC1*	Photosynthesis	Light Harvesting Machinery
RCAP_rcc00661	-0.317	0.034	0.365	0.005	0.153	0.390	*bchM*	Photosynthesis	Biosynthesis of Bacteriochlorophyll
RCAP_rcc00662	-0.214	0.231	0.224	0.272	0.024	0.899	*bchL*	Photosynthesis	Biosynthesis of Bacteriochlorophyll
RCAP_rcc00663	-0.142	0.397	0.351	0.072	0.003	0.991	*bchH*	Photosynthesis	Biosynthesis of Bacteriochlorophyll
RCAP_rcc00664	-0.170	0.298	0.525	0.008	0.001	0.991	*bchB*	Photosynthesis	Biosynthesis of Bacteriochlorophyll
RCAP_rcc00665	-0.007	0.980	0.511	0.007	-0.195	0.341	*bchN*	Photosynthesis	Biosynthesis of Bacteriochlorophyll
RCAP_rcc00666	0.467	0.041	0.584	0.009	-0.384	0.229	*bchF*	Photosynthesis	Biosynthesis of Bacteriochlorophyll
RCAP_rcc00667	0.476	0.005	-0.320	0.247	-0.228	0.390	*ppaA*	Signal Transduction	Transcription Regulator
RCAP_rcc00668	-0.035	0.854	-0.299	0.160	1.366	0.000	*ppsR*	Signal Transduction	Transcription Regulator
RCAP_rcc00669	0.436	0.019	0.874	0.000	-0.350	0.248	*bchE*	Photosynthesis	Biosynthesis of Bacteriochlorophyll
RCAP_rcc00670	-0.479	0.001	0.939	0.000	0.127	0.566	*bchJ*	Photosynthesis	Biosynthesis of Bacteriochlorophyll
RCAP_rcc00671	-0.016	0.944	1.042	0.000	-0.193	0.382	*bchG*	Photosynthesis	Biosynthesis of Bacteriochlorophyll
RCAP_rcc00672	-0.242	0.140	0.837	0.000	0.064	0.725	*PUCC family protein*	Photosynthesis	Light Harvesting Machinery
RCAP_rcc00673	0.261	0.167	0.707	0.000	-0.233	0.317	*bchP*	Photosynthesis	Biosynthesis of Bacteriochlorophyll
RCAP_rcc00674	-0.175	0.255	0.654	0.000	-0.030	0.850	*idi2*	Photosynthesis	Terpenoid backbone biosynthesis
RCAP_rcc00675	-0.602	0.010	0.463	0.001	0.028	0.963	*bchO*	Photosynthesis	Biosynthesis of Bacteriochlorophyll
RCAP_rcc00676	-0.687	0.007	0.508	0.002	0.541	0.056	*bchD*	Photosynthesis	Biosynthesis of Bacteriochlorophyll
RCAP_rcc00677	0.099	0.564	0.812	0.000	0.008	0.977	*bchI*	Photosynthesis	Biosynthesis of Bacteriochlorophyll
RCAP_rcc00678	0.543	0.002	0.544	0.019	-0.253	0.370	*crtA*	Photosynthesis	Biosynthesis of Spheroidene
RCAP_rcc00679	0.383	0.026	1.277	0.000	-0.181	0.549	*crtI*	Photosynthesis	Biosynthesis of Spheroidene
RCAP_rcc00680	-0.705	0.004	0.999	0.000	0.613	0.048	*crtB*	Photosynthesis	Biosynthesis of Spheroidene
RCAP_rcc00681	0.375	0.044	0.927	0.000	-0.265	0.427	*tspO*	Signal Transduction	Kinase/Phosphorelay
RCAP_rcc00682	-0.143	0.430	0.676	0.000	0.102	0.562	*crtC*	Photosynthesis	Biosynthesis of Spheroidene
RCAP_rcc00683	0.175	0.323	0.630	0.000	-0.235	0.109	*crtD*	Photosynthesis	Biosynthesis of Spheroidene
RCAP_rcc00684	-0.137	0.582	1.345	0.000	0.055	0.839	*crtE*	Photosynthesis	Biosynthesis of Spheroidene
RCAP_rcc00685	-0.226	0.203	1.495	0.000	-0.063	0.651	*crtF*	Photosynthesis	Biosynthesis of Spheroidene
RCAP_rcc00686	0.480	0.030	0.714	0.000	-0.668	0.009	*bchC*	Photosynthesis	Biosynthesis of Bacteriochlorophyll
RCAP_rcc00687	-0.029	0.918	0.642	0.010	-0.428	0.003	*bchX*	Photosynthesis	Biosynthesis of Bacteriochlorophyll
RCAP_rcc00688	-0.163	0.358	0.598	0.002	-0.092	0.711	*bchY*	Photosynthesis	Biosynthesis of Bacteriochlorophyll
RCAP_rcc00689	0.077	0.723	0.744	0.001	-0.381	0.028	*bchZ*	Photosynthesis	Biosynthesis of Bacteriochlorophyll
RCAP_rcc00690	0.149	0.433	1.021	0.000	-0.227	0.261	*pufQ*	Photosynthesis	Light Harvesting Machinery
RCAP_rcc00691	0.618	0.003	1.032	0.000	-0.301	0.378	*pufB*	Photosynthesis	Light Harvesting Machinery

RCAP_rcc00692	0.581	0.004	1.000	0.000	-0.314	0.358	*pufA*	Photosynthesis	Light Harvesting Machinery
RCAP_rcc00693	0.420	0.039	1.184	0.000	-0.245	0.477	*pufL*	Photosynthesis	Light Harvesting Machinery
RCAP_rcc00694	0.357	0.078	1.173	0.000	-0.190	0.540	*pufM*	Photosynthesis	Light Harvesting Machinery
RCAP_rcc00695	0.218	0.239	1.171	0.000	-0.206	0.526	*pufX*	Photosynthesis	Light Harvesting Machinery
RCAP_rcc00696	-0.557	0.012	0.927	0.000	0.209	0.383	*dxs1*	Photosynthesis	Terpenoid backbone biosynthesis
RCAP_rcc00697	-0.782	0.001	-1.167	0.000	-0.226	0.576	*hypothetical protein*	Unknown	Unknown
RCAP_rcc00698	-0.783	0.008	0.006	0.981	-0.069	0.901	*hypothetical protein*	Unknown	Unknown
RCAP_rcc00699	0.098	0.702	0.399	0.007	-0.343	0.428	*rpoE*	Replication, Recombination and Repair	Replication
RCAP_rcc00700	-0.195	0.574	0.246	0.346	-0.397	0.356	*hypothetical protein*	Unknown	Unknown
RCAP_rcc00701	0.307	0.259	-0.577	0.002	-0.464	0.007	*bsaA1*	Sulfur Metabolism	Glutathione metabolism
RCAP_rcc00702	-0.594	0.076	-0.443	0.220	0.109	0.831	*hypothetical protein*	Unknown	Unknown
RCAP_rcc00703	-0.268	0.341	0.023	0.922	-0.397	0.273	*deoxyribodipyrimidine photolyase-related protein*	Unknown	Unknown
RCAP_rcc00704	-0.011	0.972	-0.116	0.559	0.037	0.883	*oppC1*	Metal, Ion, Cofactor Transport	Nickel Transport
RCAP_rcc00705	0.137	0.676	-0.030	0.902	-0.155	0.526	*oppB1*	Metal, Ion, Cofactor Transport	Nickel Transport
RCAP_rcc00706	0.375	0.168	-0.282	0.156	-0.097	0.751	*oppA1*	Unknown	Unknown
RCAP_rcc00707	0.052	0.878	0.354	0.061	-0.015	0.967	*oppF*	Amino Acid Metabolism	Amino Acid Transport
RCAP_rcc00708	0.072	0.836	0.610	0.000	-0.172	0.482	*oppD1*	Amino Acid Metabolism	Amino Acid Transport
RCAP_rcc00709	-0.154	0.455	-0.399	0.003	0.407	0.127	*FAD dependent oxidoreductase*	Energy Metabolism	Unknown
RCAP_rcc00710	0.033	0.911	-0.595	0.000	0.013	0.959	*hipO*	Amino Acid Metabolism	Phenylalanine metabolism
RCAP_rcc00711	0.437	0.126	-1.589	0.000	-0.101	0.716	*universal stress family protein*	Stress Response	Unknown
RCAP_rcc00712	-0.511	0.004	-1.019	0.000	0.002	0.995	*moaA1*	Metabolism of Cofactors, Coenzymes and Vitamins	Unknown
RCAP_rcc00713	-0.284	0.157	0.180	0.353	0.251	0.269	*kynU*	Amino Acid Metabolism	Tryptophan metabolism
RCAP_rcc00714	-0.882	0.008	-0.243	0.237	0.652	0.097	*hypothetical protein*	Unknown	Unknown
RCAP_rcc00715	0.262	0.303	-0.172	0.473	-0.167	0.467	*pntA*	Metabolism of Cofactors, Coenzymes and Vitamins	Nicotinate and nicotinamide metabolism
RCAP_rcc00716	0.205	0.297	-0.151	0.480	-0.187	0.356	*pntB*	Metabolism of Cofactors, Coenzymes and Vitamins	Nicotinate and nicotinamide metabolism
RCAP_rcc00717	-0.094	0.733	0.410	0.047	0.044	0.899	*hypothetical protein*	Unknown	Unknown
RCAP_rcc00718	0.058	0.838	-0.541	0.003	0.063	0.845	*mdh*	Carbohydrate Metabolism	TCA Cycle
RCAP_rcc00719	-0.194	0.483	-0.085	0.722	0.071	0.859	*hypothetical protein*	Unknown	Unknown
RCAP_rcc00720	0.566	0.012	-0.099	0.742	-0.189	0.562	*sucC*	Carbohydrate Metabolism	TCA Cycle
RCAP_rcc00721	0.151	0.496	-0.056	0.838	0.015	0.964	*sucD*	Carbohydrate Metabolism	TCA Cycle
RCAP_rcc00722	-0.709	0.015	-0.191	0.504	0.768	0.034	*lipoprotein*	Predicted Function	Unknown
RCAP_rcc00723	-0.291	0.361	0.203	0.491	-0.025	0.960	*lipoprotein*	Predicted Function	Unknown
RCAP_rcc00724	0.452	0.022	0.240	0.258	-0.137	0.673	*sucA*	Carbohydrate Metabolism	TCA Cycle
RCAP_rcc00725	0.277	0.172	0.078	0.733	-0.010	0.981	*sucB*	Carbohydrate Metabolism	TCA Cycle
RCAP_rcc00726	0.305	0.136	0.010	0.967	-0.002	0.992	*lpdA1*	Carbohydrate Metabolism	TCA Cycle
RCAP_rcc00727	0.020	0.944	0.251	0.243	-0.075	0.780	*citE1*	Carbohydrate Metabolism	Glyoxylate and dicarboxylate metabolism
RCAP_rcc00728	0.563	0.010	-0.572	0.000	-0.222	0.511	*NnrU family protein*	Energy Metabolism	Nitrogen Metabolism
RCAP_rcc00729	-0.930	0.000	-0.727	0.000	0.487	0.079	*hypothetical protein*	Unknown	Unknown
RCAP_rcc00730	0.249	0.201	-0.476	0.001	-0.051	0.814	*maoC*	Carbohydrate Metabolism	Glyoxylate and dicarboxylate metabolism
RCAP_rcc00731	0.612	0.009	-0.862	0.000	-0.296	0.354	*sdhC*	Carbohydrate Metabolism	TCA Cycle
RCAP_rcc00732	0.503	0.030	-0.957	0.000	-0.295	0.372	*sdhD*	Carbohydrate Metabolism	TCA Cycle
RCAP_rcc00733	0.054	0.757	-0.993	0.000	0.060	0.720	*sdhA*	Carbohydrate Metabolism	TCA Cycle
RCAP_rcc00734	-0.800	0.015	-0.997	0.000	0.548	0.159	*lipoprotein*	Predicted Function	Unknown
RCAP_rcc00735	-0.262	0.260	-0.990	0.000	0.318	0.354	*hypothetical protein*	Unknown	Unknown
RCAP_rcc00736	0.659	0.002	-1.166	0.000	-0.267	0.453	*sdhB*	Carbohydrate Metabolism	TCA Cycle
RCAP_rcc00737	0.986	0.001	-2.903	0.000	-1.038	0.000	*hypothetical protein*	Unknown	Unknown
RCAP_rcc00738	0.324	0.175	-0.039	0.894	-0.363	0.043	*hypothetical protein*	Unknown	Unknown
RCAP_rcc00739	-0.459	0.067	-0.351	0.303	0.105	0.719	*ArsR family transcriptional regulator*	Signal Transduction	Transcription Regulator
RCAP_rcc00740	0.532	0.008	0.289	0.046	-0.225	0.556	*atpI*	Unknown	Unknown
RCAP_rcc00741	0.424	0.167	-0.063	0.800	-0.082	0.845	*atpB*	Energy Metabolism	Methane metabolism
RCAP_rcc00742	0.240	0.199	-0.066	0.786	0.056	0.776	*atpE*	Energy Metabolism	Methane metabolism
RCAP_rcc00743	0.079	0.748	-0.166	0.408	0.037	0.914	*atpX*	Energy Metabolism	Methane metabolism
RCAP_rcc00744	0.301	0.097	-0.386	NA	-0.014	0.965	*atpF*	Energy Metabolism	Methane metabolism
RCAP_rcc00745	0.033	0.919	-0.030	0.886	-0.471	0.003	*phaZ*	Lipid Metabolism	Unknown
RCAP_rcc00746	0.637	0.015	0.121	0.690	-0.214	0.525	*phbC*	Carbohydrate Metabolism	Polyhydroxybutyrate
RCAP_rcc00747	1.924	0.000	-1.315	0.000	-0.772	0.036	*hypothetical protein*	Unknown	Unknown
RCAP_rcc00748	0.515	0.064	-0.379	0.083	-0.584	0.003	*phaR*	Carbohydrate Metabolism	Polyhydroxybutyrate
RCAP_rcc00749	-0.372	0.163	0.023	0.936	0.043	0.948	*LysR family transcriptional regulator*	Signal Transduction	Transcription Regulator
RCAP_rcc00750	0.189	0.497	0.366	0.013	-0.158	0.484	*pyrG*	Nucleotide Metabolism	Pyrimidine metabolism
RCAP_rcc00751	0.182	0.641	0.116	0.616	0.182	0.429	*secG*	Trafficking and Secretion	Secretion
RCAP_rcc00752	-0.021	0.958	0.461	0.002	0.110	0.574	*purA*	Amino Acid Metabolism	Alanine, aspartate and glutamate metabolism
RCAP_rcc00753	-0.039	0.911	0.273	0.045	0.479	0.248	*hypothetical protein*	Unknown	Unknown
RCAP_rcc00754	0.202	0.383	-0.622	0.000	0.059	0.819	*lipoprotein*	Predicted Function	Unknown
RCAP_rcc00755	-0.217	0.310	0.043	0.879	0.220	0.441	*thiN*	Metabolism of Cofactors, Coenzymes and Vitamins	Thiamine metabolism
RCAP_rcc00756	-0.314	0.185	0.230	0.251	0.025	0.950	*dme family transporter*	Predicted Function	Replication
RCAP_rcc00757	0.185	0.425	0.648	0.000	0.096	0.727	*alpha/beta fold family hydrolase*	Photosynthesis	Tropane, piperidine and pyridine alkaloid biosynthesis
RCAP_rcc00758	-0.182	0.547	0.392	0.094	0.225	0.574	*alpha/beta fold family hydrolase*	Unknown	Unknown
RCAP_rcc00759	-0.188	0.525	4.276	0.000	0.250	0.387	*mcpB*	Motility	Chemotaxis
RCAP_rcc00760	-0.240	0.241	3.761	0.000	0.231	0.285	*mcpA1*	Motility	Chemotaxis
RCAP_rcc00761	-1.163	0.000	-1.510	0.000	0.536	0.076	*hoxH*	Energy Metabolism	Methane metabolism
RCAP_rcc00762	-1.155	0.001	-1.741	0.000	0.667	0.059	*hoxW*	Post-translational Modification, Assembly and Chaperones	Unknown
RCAP_rcc00763	-0.421	0.031	-0.252	0.062	0.348	0.026	*hupT*	Energy Metabolism	Aerobic/Anaerobic Respiration
RCAP_rcc00764	-0.550	0.044	-0.415	0.004	0.246	0.526	*hupU*	Energy Metabolism	Aerobic/Anaerobic Respiration
RCAP_rcc00765	-0.448	0.082	-0.416	0.072	0.387	0.273	*hupV*	Energy Metabolism	Aerobic/Anaerobic Respiration
RCAP_rcc00766	-0.086	0.755	-0.464	0.034	-0.076	0.810	*hypF*	Energy Metabolism	Aerobic/Anaerobic Respiration
RCAP_rcc00767	0.309	0.249	-2.842	0.000	-0.154	0.686	*hupA*	Energy Metabolism	Aerobic/Anaerobic Respiration
RCAP_rcc00768	0.129	0.632	-3.196	0.000	0.027	0.957	*hupB*	Energy Metabolism	Aerobic/Anaerobic Respiration

RCAP_rcc00769	0.235	0.423	-3.399	0.000	0.012	NA	*hupC*	Energy Metabolism	Aerobic/Anaerobic Respiration
RCAP_rcc00770	-0.027	0.910	-1.592	0.000	-0.133	0.693	*hupD*	Energy Metabolism	Aerobic/Anaerobic Respiration
RCAP_rcc00771	-0.698	0.083	-2.051	0.000	0.339	0.429	*hupF*	Energy Metabolism	Aerobic/Anaerobic Respiration
RCAP_rcc00772	0.136	0.623	-1.954	0.000	0.131	0.720	*hupG*	Energy Metabolism	Aerobic/Anaerobic Respiration
RCAP_rcc00773	-0.142	0.538	-2.249	0.000	0.190	0.486	*hupH*	Energy Metabolism	Aerobic/Anaerobic Respiration
RCAP_rcc00774	-0.141	0.515	-1.926	0.000	0.082	0.781	*hupJ*	Energy Metabolism	Aerobic/Anaerobic Respiration
RCAP_rcc00775	-0.422	0.313	-2.221	0.000	0.471	0.191	*hupK*	Energy Metabolism	Aerobic/Anaerobic Respiration
RCAP_rcc00776	-0.210	0.408	-1.694	0.000	0.063	0.856	*hypA*	Energy Metabolism	Aerobic/Anaerobic Respiration
RCAP_rcc00777	-0.091	0.669	-1.750	0.000	0.082	0.762	*hypB*	Energy Metabolism	Aerobic/Anaerobic Respiration
RCAP_rcc00778	-0.371	0.053	-1.648	0.000	0.229	0.402	*hupR*	Signal Transduction	Transcription Regulator
RCAP_rcc00779	-0.269	0.284	-1.686	0.000	0.049	0.902	*hypC*	Energy Metabolism	Aerobic/Anaerobic Respiration
RCAP_rcc00780	-0.295	0.095	-1.622	0.000	0.269	0.224	*hypD*	Energy Metabolism	Aerobic/Anaerobic Respiration
RCAP_rcc00781	-0.219	0.569	-1.109	0.000	0.315	0.488	*hypE*	Energy Metabolism	Aerobic/Anaerobic Respiration
RCAP_rcc00782	-0.210	0.198	-0.766	0.000	0.117	0.531	*cheR1*	Motility	Chemotaxis
RCAP_rcc00783	-0.796	0.000	-1.063	0.000	0.480	0.102	*diguanylate cyclase/phosphodiesterase*	Signal Transduction	Kinase/Phosphorelay
RCAP_rcc00784	0.123	0.747	-0.742	0.019	-0.397	0.316	*hypothetical protein*	Unknown	Unknown
RCAP_rcc00785	-0.060	0.849	-0.594	0.002	-0.153	0.442	*sqr*	Energy Metabolism	Unknown
RCAP_rcc00786	0.166	0.310	-0.645	0.000	0.124	0.637	*basic membrane lipoprotein family*	Predicted Function	Unknown
RCAP_rcc00787	-0.606	0.004	-0.662	0.000	0.691	0.023	*monosacharide ABC transporter permease*	Carbohydrate Metabolism	Aerobic/Anaerobic Respiration
RCAP_rcc00788	0.131	0.514	-0.290	0.009	-0.062	0.802	*monosacharide ABC transporter permease*	Carbohydrate Metabolism	Aerobic/Anaerobic Respiration
RCAP_rcc00789	-0.384	0.026	-0.223	0.040	0.270	0.253	*monosacharide ABC transporter ATP-binding protein*	Carbohydrate Metabolism	Aerobic/Anaerobic Respiration
RCAP_rcc00790	-0.078	0.817	-1.375	0.000	0.217	0.406	*xdhC*	Post-translational Modification, Assembly and Chaperones	Unknown
RCAP_rcc00791	-0.024	0.940	-1.195	0.000	0.163	0.311	*xdhB*	Nucleotide Metabolism	Purine metabolism
RCAP_rcc00792	0.078	0.806	-0.977	0.000	0.179	0.384	*xdhA*	Nucleotide Metabolism	Unknown
RCAP_rcc00793	-0.049	0.860	0.056	0.777	0.022	0.947	*dnaE1*	Replication, Recombination and Repair	Replication
RCAP_rcc00794	-0.574	0.036	0.173	0.633	0.209	0.582	*SlyX family protein*	Unknown	Unknown
RCAP_rcc00795	0.032	0.929	0.256	0.162	0.012	0.960	*hisS*	Translation, ribosomal structure and biogenesis	Aminoacyl-tRNA biosynthesis
RCAP_rcc00796	-0.275	0.215	0.082	0.612	-0.078	0.730	*hisZ*	Signal Transduction	Transcription Regulator
RCAP_rcc00797	-0.736	0.000	0.201	0.248	0.334	0.211	*hisG*	Amino Acid Metabolism	Histidine metabolism
RCAP_rcc00798	-0.672	0.002	-0.025	0.922	0.519	0.074	*D-isomer specific 2-hydroxyacid dehydrogenase*	Carbohydrate Metabolism	Glyoxylate and dicarboxylate metabolism
RCAP_rcc00799	0.087	0.782	0.045	0.795	-0.101	0.738	*mrdB*	Cell Envelope Biosynthesis	Cell Wall Biosynthesis
RCAP_rcc00800	-0.182	0.405	0.150	0.296	0.160	0.477	*mrdA*	Cell Envelope Biosynthesis	Cell Wall Biosynthesis
RCAP_rcc00801	-0.116	0.699	0.122	0.669	-0.224	0.452	*hypothetical protein*	Unknown	Unknown
RCAP_rcc00802	-0.249	0.420	0.147	0.300	0.159	0.477	*mreC*	Cell Envelope Biosynthesis	Cell Wall Biosynthesis
RCAP_rcc00803	-0.171	0.599	0.259	0.147	0.120	0.664	*mreB*	Cell Division	Chromosome Partitioning
RCAP_rcc00804	0.080	0.749	0.609	0.004	-0.314	0.388	*nhaA*	Metal and Ion Transport	Unknown
RCAP_rcc00805	-0.260	0.303	0.101	0.688	0.203	0.482	*GNAT family acetyltransferase*	Cell Division	Chromosome Partitioning
RCAP_rcc00806	-0.897	0.000	-0.105	0.662	0.237	0.504	*heat shock protein DnaJ domain-containing protein*	Stress Response	Unknown
RCAP_rcc00807	-0.609	0.002	-0.108	0.662	0.121	0.738	*endonuclease/exonuclease/phosphatase*	Unknown	Unknown
RCAP_rcc00808	0.209	0.462	-0.733	0.001	-0.571	0.127	*hypothetical protein*	Unknown	Unknown
RCAP_rcc00809	-0.239	0.193	-0.293	0.003	0.091	0.769	*ppx*	Nucleotide Metabolism	Purine metabolism
RCAP_rcc00810	0.292	0.185	-0.260	0.041	-0.120	0.661	*ppk*	Energy Metabolism	Oxidative phosphorylation
RCAP_rcc00811	-0.456	0.163	-0.155	0.408	0.424	0.297	*hda*	Replication, Recombination and Repair	Unknown
RCAP_rcc00812	0.076	0.706	0.037	0.823	-0.396	0.050	*hypothetical protein*	Unknown	Unknown
RCAP_rcc00813	0.016	0.968	-0.556	0.032	0.333	0.388	*hypothetical protein*	Unknown	Unknown
RCAP_rcc00814	-0.480	0.184	0.087	0.777	0.184	0.701	*LysR family transcriptional regulator*	Signal Transduction	Transcription Regulator
RCAP_rcc00815	-0.184	0.698	0.396	0.351	0.170	0.691	*DoxX family protein*	Unknown	Unknown
RCAP_rcc00816	-0.483	0.016	0.360	0.020	0.203	0.410	*extradiol ring-cleavage dioxygenase subunit B*	Unknown	Unknown
RCAP_rcc00817	-0.812	0.008	0.233	0.436	0.729	0.047	*ldh*	Carbohydrate Metabolism	Glycolysis / Gluconeogenesis
RCAP_rcc00818	-0.561	0.010	0.039	0.758	0.215	0.251	*ftsW*	Cell Division	Chromosome Partitioning
RCAP_rcc00819	-0.547	0.081	0.019	0.932	0.434	0.212	*murG*	Glycan Biosynthesis and Metabolism	Peptidoglycan biosynthesis
RCAP_rcc00820	-0.101	0.670	0.238	0.094	-0.004	0.988	*murC*	Glycan Biosynthesis and Metabolism	Peptidoglycan biosynthesis
RCAP_rcc00821	-0.657	0.075	0.119	0.661	0.625	0.113	*hypothetical protein*	Unknown	Unknown
RCAP_rcc00822	0.012	0.968	0.166	0.235	0.072	0.779	*murB*	Cell Envelope Biosynthesis	Cell Wall Biosynthesis
RCAP_rcc00823	-0.323	0.077	0.193	0.136	0.048	0.822	*ddl*	Glycan Biosynthesis and Metabolism	Peptidoglycan biosynthesis
RCAP_rcc00824	-0.590	0.048	-0.217	0.040	0.409	0.267	*ftsQ*	Cell Envelope Biosynthesis	Cell Wall Biosynthesis
RCAP_rcc00825	-0.235	0.227	-0.183	0.064	0.132	0.429	*ftsA*	Cell Division	Chromosome Partitioning
RCAP_rcc00826	0.304	0.067	-0.160	NA	-0.145	0.607	*ftsZ*	Unknown	Unknown
RCAP_rcc00827	-0.140	0.552	0.228	0.267	0.141	0.699	*lpxC*	Xenobiotics Biodegradation and Metabolism	Caprolactam degradation
RCAP_rcc00828	0.356	0.058	0.254	0.135	-0.284	0.273	*comL*	Cell Envelope Biosynthesis	Cell Wall Biosynthesis
RCAP_rcc00829	-0.341	0.117	-0.065	0.659	0.295	0.248	*recN*	Replication, Recombination and Repair	Recombination
RCAP_rcc00830	-0.477	0.103	0.204	0.362	0.278	0.476	*hypothetical protein*	Unknown	Unknown
RCAP_rcc00831	-0.458	0.058	0.448	0.001	0.369	0.167	*dld*	Carbohydrate Metabolism	Pyruvate metabolism
RCAP_rcc00832	0.467	0.006	-0.569	0.005	-0.025	0.938	*pta*	Energy Metabolism	Reductive carboxylate cycle (CO2 fixation)
RCAP_rcc00833	-0.125	0.493	-0.598	0.000	0.213	0.267	*ackA1*	Energy Metabolism	Reductive carboxylate cycle (CO2 fixation)
RCAP_rcc00834	-0.324	0.080	-0.403	0.000	0.405	0.109	*pepP*	Amino Acid Metabolism	Unknown
RCAP_rcc00835	-0.211	0.265	-0.098	0.492	0.193	0.268	*cobT*	Metabolism of Cofactors, Coenzymes and Vitamins	Cobalamin Biosynthesis
RCAP_rcc00836	0.576	0.011	-0.032	0.834	-0.274	0.421	*cobS*	Metabolism of Cofactors, Coenzymes and Vitamins	Cobalamin Biosynthesis
RCAP_rcc00837	-0.251	0.538	-0.549	0.069	0.403	0.170	*hypothetical protein*	Unknown	Unknown
RCAP_rcc00838	0.726	0.000	0.375	0.085	-0.304	0.326	*DnaJ domain-containing protein*	Post-translational Modification, Assembly and Chaperones	Unknown
RCAP_rcc00839	0.227	NA	-0.020	0.886	0.028	0.888	*glcB*	Carbohydrate Metabolism	Glyoxylate and dicarboxylate metabolism
RCAP_rcc00840	-0.009	0.972	-0.432	0.003	0.132	0.344	*pepN*	Metabolism of Other Amino Acids	Glutathione metabolism
RCAP_rcc00841	0.398	0.024	0.159	0.229	-0.230	0.459	*gatB*	Translation, ribosomal structure and biogenesis	Aminoacyl-tRNA biosynthesis
RCAP_rcc00842	0.189	0.479	0.684	0.072	-0.209	0.578	*hypothetical protein*	Unknown	Unknown
RCAP_rcc00843	-0.330	0.301	0.834	0.000	0.141	0.591	*hypothetical protein*	Unknown	Unknown
RCAP_rcc00844	-0.481	0.099	2.174	0.000	0.768	0.007	*hypothetical protein*	Unknown	Unknown
RCAP_rcc00845	-0.078	0.723	1.801	0.000	0.011	0.984	*hypothetical protein*	Unknown	Unknown

RCAP_rcc00846	0.426	0.074	0.415	0.099	-0.094	0.713	*dppA*	Unknown	Unknown
RCAP_rcc00847	0.110	0.733	0.138	0.569	-0.028	0.921	*dppB*	Metal, Ion, Cofactor Transport	Nickel Transport
RCAP_rcc00848	-0.112	0.620	-0.029	0.913	0.237	0.366	*dppC*	Metal, Ion, Cofactor Transport	Nickel Transport
RCAP_rcc00849	-0.067	0.801	0.049	0.815	0.154	0.402	*dppD*	Predicted Function	Nickel Transport
RCAP_rcc00850	-0.138	0.543	-0.069	0.786	0.196	0.578	*dppF*	Amino Acid Metabolism	Amino Acid Transport
RCAP_rcc00851	-0.411	0.070	-0.289	0.051	0.365	0.223	*membrane dipeptidase*	Cell Envelope Biosynthesis	Cell Wall Biosynthesis
RCAP_rcc00852	-0.227	0.344	-0.126	0.521	0.221	0.140	*srmB*	Replication, Recombination and Repair	Unknown
RCAP_rcc00853	-0.297	0.272	-0.095	0.718	0.275	0.449	*hypothetical protein*	Unknown	Unknown
RCAP_rcc00854	0.315	0.055	0.274	0.010	-0.089	0.752	*argS*	Translation, ribosomal structure and biogenesis	Aminoacyl-tRNA biosynthesis
RCAP_rcc00855	-0.762	0.011	-0.010	0.955	0.614	NA	*sporulation domain-containing protein*	Unknown	Unknown
RCAP_rcc00856	-0.844	0.001	0.003	0.989	0.463	0.157	*nagZ*	Glycan Biosynthesis and Metabolism	Glycosphingolipid biosynthesis - ganglio series
RCAP_rcc00857	-0.255	0.354	0.140	0.613	-0.021	0.957	*scpA*	Unknown	Unknown
RCAP_rcc00858	-0.606	0.002	0.191	0.289	0.348	0.168	*scpB*	Transcription	Unknown
RCAP_rcc00859	-0.473	0.140	-0.113	0.698	0.188	0.667	*hypothetical protein*	Unknown	Unknown
RCAP_rcc00860	-0.482	0.126	-0.280	0.398	0.523	0.186	*enoyl-CoA hydratase/isomerase*	Xenobiotics Biodegradation and Metabolism	Caprolactam degradation
RCAP_rcc00861	-0.699	0.038	0.386	0.463	0.113	0.787	*thioesterase*	Secondary metabolites biosynthesis, transport, and catabolism	Unknown
RCAP_rcc00862	0.212	0.591	1.413	0.000	0.163	0.721	*divalent anion:Na+ symporter*	Metal and Ion Transport	Replication
RCAP_rcc00863	0.346	0.189	0.522	0.010	-0.155	0.636	*rplM*	Translation, ribosomal structure and biogenesis	Unknown
RCAP_rcc00864	-0.114	0.739	0.216	0.284	0.027	0.929	*rpsI*	Translation, ribosomal structure and biogenesis	Unknown
RCAP_rcc00865	0.059	0.793	-0.471	0.001	0.065	0.728	*hemolysin-type calcium-binding repeat family protein*	Trafficking and Secretion	Secretion
RCAP_rcc00866	-0.805	0.032	-0.312	0.156	0.362	0.387	*argO*	Unknown	Unknown
RCAP_rcc00867	0.071	NA	0.057	0.936	0.024	NA	*LysR family transcriptional regulator*	Signal Transduction	Transcription Regulator
RCAP_rcc00868	-0.226	0.485	0.365	0.145	-0.011	0.986	*hypothetical protein*	Unknown	Unknown
RCAP_rcc00869	0.164	0.443	0.984	0.000	-0.084	0.680	*adhC*	Carbohydrate Metabolism	Glycolysis / Gluconeogenesis
RCAP_rcc00870	0.210	0.310	0.807	0.000	-0.354	0.080	*fghA*	Sulfur Metabolism	Glutathione metabolism
RCAP_rcc00871	1.175	0.000	0.829	0.000	-0.354	0.347	*hypothetical protein*	Unknown	Unknown
RCAP_rcc00872	-0.072	0.733	-0.047	0.795	0.210	0.353	*alcohol dehydrogenase*	Energy Metabolism	Unknown
RCAP_rcc00873	0.468	0.031	0.177	0.287	-0.220	0.511	*XRE family transcriptional regulator*	Signal Transduction	Transcription Regulator
RCAP_rcc00874	-0.841	0.017	-0.317	0.061	0.703	0.064	*inositol monophosphatase*	Amino Acid Metabolism	Histidine metabolism
RCAP_rcc00875	-0.054	0.817	-0.348	0.031	0.280	0.168	*atzB*	Metabolism of Cofactors, Coenzymes and Vitamins	Riboflavin metabolism
RCAP_rcc00876	0.226	0.390	-0.482	0.001	0.017	0.951	*guaD*	Nucleotide Metabolism	Purine metabolism
RCAP_rcc00877	-0.011	0.963	-0.002	0.993	0.040	0.839	*mgtE*	Metal, Ion, Cofactor Transport	Magnesium Transport
RCAP_rcc00878	-0.836	0.032	-0.447	0.157	0.618	0.109	*5-formyltetrahydrofolate cyclo-ligase*	Metabolism of Cofactors, Coenzymes and Vitamins	One carbon pool by folate
RCAP_rcc00879	-0.106	0.691	0.659	0.000	-0.140	0.579	*phnA*	Metal and Ion Transport	Unknown
RCAP_rcc00880	0.869	0.002	-1.033	0.000	0.170	0.539	*CHAP domain-containing protein*	Unknown	Unknown
RCAP_rcc00881	-0.528	0.015	0.084	0.624	0.445	0.083	*metallophosphoesterase*	Unknown	Unknown
RCAP_rcc00882	-0.070	0.700	-0.075	0.566	0.128	0.526	*divalent ion symporter*	Metal and Ion Transport	Replication
RCAP_rcc00883	-0.319	0.282	-0.475	0.007	0.561	0.114	*lipoprotein*	Predicted Function	Unknown
RCAP_rcc00884	0.649	0.022	0.352	0.130	-0.233	0.553	*hypothetical protein*	Unknown	Unknown
RCAP_rcc00885	-0.143	0.746	-1.874	0.000	0.013	0.984	*hypothetical protein*	Unknown	Unknown
RCAP_rcc00886	-0.498	0.231	-2.465	0.000	0.379	0.385	*ABC transporter ATP-binding protein*	Defense Mechanisms	Unknown
RCAP_rcc00887	-0.171	0.648	-2.512	0.000	0.419	0.284	*hypothetical protein*	Unknown	Unknown
RCAP_rcc00888	0.201	NA	-2.371	0.000	0.069	0.896	*hypothetical protein*	Unknown	Unknown
RCAP_rcc00889	0.784	0.045	-2.808	0.000	0.452	0.278	*NosL family protein*	Unknown	Unknown
RCAP_rcc00890	1.379	0.000	-2.465	0.000	-0.134	0.683	*hypothetical protein*	Unknown	Unknown
RCAP_rcc00891	-0.250	0.577	-2.512	0.000	0.798	0.033	*hypothetical protein*	Unknown	Unknown
RCAP_rcc00892	-0.163	0.563	-1.254	0.000	0.042	0.877	*hypothetical protein*	Unknown	Unknown
RCAP_rcc00893	-0.043	0.877	-0.576	0.001	0.372	0.037	*proV1*	Amino Acid Metabolism	Amino Acid Transport
RCAP_rcc00894	0.292	0.260	-0.477	0.038	0.061	0.845	*proW1*	Unknown	Unknown
RCAP_rcc00895	0.552	0.036	-0.339	0.106	-0.157	0.658	*proX1*	Amino Acid Metabolism	Amino Acid Transport
RCAP_rcc00896	0.144	0.570	0.518	0.061	-0.014	0.969	*betI*	Signal Transduction	Transcription Regulator
RCAP_rcc00897	0.160	0.425	0.433	0.178	0.172	0.374	*betB*	Amino Acid Metabolism	Glycine, serine and threonine metabolism
RCAP_rcc00898	-0.069	0.763	0.518	0.051	0.241	0.157	*betA*	Amino Acid Metabolism	Glycine, serine and threonine metabolism
RCAP_rcc00899	-0.071	0.794	0.388	0.026	-0.262	0.241	*nuclease*	Replication, Recombination and Repair	Unknown
RCAP_rcc00900	0.330	0.304	-0.082	0.747	-0.204	0.449	*hypothetical protein*	Unknown	Unknown
RCAP_rcc00901	2.777	0.000	-1.378	0.000	-0.352	0.312	*hypothetical protein*	Unknown	Unknown
RCAP_rcc00902	-0.710	0.000	-0.021	0.936	0.188	0.504	*XRE family transcriptional regulator*	Signal Transduction	Transcription Regulator
RCAP_rcc00903	-0.994	0.009	-0.959	0.000	0.905	0.011	*hypothetical protein*	Unknown	Unknown
RCAP_rcc00904	-0.815	0.009	-1.364	0.000	0.528	0.072	*major facilitator superfamily protein*	Metal and Ion Transport	Unknown
RCAP_rcc00905	0.411	0.035	-0.044	0.810	-0.068	0.869	*hypothetical protein*	Unknown	Unknown
RCAP_rcc00906	0.106	0.747	-0.751	0.017	-0.005	0.991	*pccB*	Carbohydrate Metabolism	Glyoxylate and dicarboxylate metabolism
RCAP_rcc00907	-0.223	0.466	-0.702	0.008	0.112	0.777	*hypothetical protein*	Unknown	Unknown
RCAP_rcc00908	0.699	0.002	-0.514	0.000	-0.332	0.327	*lipoprotein*	Predicted Function	Unknown
RCAP_rcc00909	-0.602	0.025	0.059	0.730	0.509	0.079	*hypothetical protein*	Unknown	Unknown
RCAP_rcc00910	0.170	0.640	-0.886	0.000	0.376	0.382	*hypothetical protein*	Unknown	Unknown
RCAP_rcc00911	0.111	0.604	-0.289	0.143	-0.045	0.894	*pccA*	Carbohydrate Metabolism	Glyoxylate and dicarboxylate metabolism
RCAP_rcc00912	-0.183	0.344	-0.247	0.227	0.157	0.300	*bhbA*	Carbohydrate Metabolism	Glyoxylate and dicarboxylate metabolism
RCAP_rcc00913	0.555	0.022	-0.147	0.507	-0.233	0.482	*bfr*	Metal, Ion, Cofactor Transport	Iron and Heme Transport
RCAP_rcc00914	0.569	0.047	0.036	0.927	-0.678	0.072	*hypothetical protein*	Unknown	Unknown
RCAP_rcc00915	0.167	0.374	-0.260	0.112	0.082	0.642	*psd*	Lipid Metabolism	Glycerophospholipid metabolism
RCAP_rcc00916	0.265	0.154	-0.414	0.004	-0.102	0.757	*pssA*	Lipid Metabolism	Glycerophospholipid metabolism
RCAP_rcc00917	0.172	0.517	-0.267	0.337	-0.095	0.766	*hypothetical protein*	Unknown	Unknown
RCAP_rcc00918	-0.264	0.499	0.390	0.397	-0.030	0.964	*N-acetylmuramoyl-L-alanine amidase*	Cell Envelope Biosynthesis	Cell Wall Biosynthesis
RCAP_rcc00919	0.397	0.260	0.425	0.258	-0.246	0.594	*hypothetical protein*	Unknown	Unknown
RCAP_rcc00920	0.595	0.059	0.683	0.016	-0.180	0.704	*hypothetical protein*	Unknown	Unknown
RCAP_rcc00921	-0.156	NA	0.635	0.111	0.003	NA	*NlpC/P60 family phage cell wall peptidase*	Replication, Recombination and Repair	Phage Interaction
RCAP_rcc00922	0.089	0.836	0.290	0.515	0.053	0.931	*hypothetical protein*	Unknown	Unknown

RCAP_rcc00923	0.019	0.971	0.834	0.185	-0.423	0.314	*hypothetical protein*	Unknown	Unknown
RCAP_rcc00924	-0.054	0.911	0.856	0.056	-0.189	NA	*hypothetical protein*	Unknown	Unknown
RCAP_rcc00925	-0.373	0.343	0.431	0.187	0.126	0.791	*hypothetical protein*	Unknown	Unknown
RCAP_rcc00926	-0.130	NA	0.551	0.267	0.341	NA	*hypothetical protein*	Unknown	Unknown
RCAP_rcc00927	0.107	0.817	0.523	0.189	-0.167	0.730	*hypothetical protein*	Unknown	Unknown
RCAP_rcc00928	0.272	0.525	0.227	0.478	0.029	0.964	*hypothetical protein*	Unknown	Unknown
RCAP_rcc00929	-0.148	0.649	-0.303	0.309	-0.096	0.823	*hypothetical protein*	Unknown	Unknown
RCAP_rcc00930	0.331	0.297	-0.479	0.020	-0.161	0.655	*hypothetical protein*	Unknown	Unknown
RCAP_rcc00931	-0.583	0.157	0.127	0.807	0.191	0.689	*hypothetical protein*	Unknown	Unknown
RCAP_rcc00932	-0.151	NA	-0.042	0.961	0.055	NA	*hypothetical protein*	Unknown	Unknown
RCAP_rcc00933	-0.215	NA	-0.501	0.472	-0.038	NA	*hypothetical protein*	Unknown	Unknown
RCAP_rcc00934	0.487	0.167	0.274	0.463	-0.307	0.479	*U35 family peptidase*	Post-translational Modification, Assembly and Chaperones	Peptidase
RCAP_rcc00935	0.633	0.005	-0.023	0.930	-0.438	0.080	*hypothetical protein*	Unknown	Unknown
RCAP_rcc00936	0.562	0.024	0.087	0.748	-0.429	0.041	*lambda family phage portal protein*	Replication, Recombination and Repair	Phage Interaction
RCAP_rcc00937	0.559	0.174	0.754	0.221	-0.695	0.059	*hypothetical protein*	Unknown	Unknown
RCAP_rcc00938	1.453	0.000	0.027	0.960	-0.473	0.249	*phage terminase large subunit*	Replication, Recombination and Repair	Phage Interaction
RCAP_rcc00939	0.302	NA	1.191	0.059	-0.042	NA	*hypothetical protein*	Unknown	Unknown
RCAP_rcc00940	-0.115	0.815	-0.047	0.930	-0.007	0.991	*hypothetical protein*	Unknown	Unknown
RCAP_rcc00941	-0.057	0.809	-0.247	0.106	0.218	0.361	*hypothetical protein*	Unknown	Unknown
RCAP_rcc00942	-0.524	0.215	0.226	0.636	-0.052	0.934	*hypothetical protein*	Unknown	Unknown
RCAP_rcc00943	0.670	0.101	0.804	0.163	-0.478	0.251	*cytosine-N(4)-specific DNA-methyltransferase*	Replication, Recombination and Repair	Unknown
RCAP_rcc00944	0.069	NA	-0.036	0.962	-0.511	0.192	*ParB domain-containing protein nuclease*	Transcription	Unknown
RCAP_rcc00945	-0.144	NA	0.578	0.401	-0.393	NA	*hypothetical protein*	Unknown	Unknown
RCAP_rcc00946	0.292	0.508	0.224	0.662	-0.340	0.431	*MarR family transcriptional regulator*	Signal Transduction	Transcription Regulator
RCAP_rcc00947	-0.184	NA	-0.013	0.989	-0.035	NA	*hypothetical protein*	Unknown	Unknown
RCAP_rcc00948	-0.245	NA	0.524	0.432	-0.179	NA	*hypothetical protein*	Unknown	Unknown
RCAP_rcc00949	0.574	0.023	-0.308	0.022	-0.416	0.153	*hypothetical protein*	Unknown	Unknown
RCAP_rcc00950	-0.078	0.807	-0.232	0.296	-0.419	0.131	*phage integrase*	Replication, Recombination and Repair	Phage Interaction
RCAP_rcc00951	0.190	0.546	-0.559	0.003	-0.183	0.571	*hsdR1*	Defense Mechanisms	Unknown
RCAP_rcc00952	0.264	0.341	-0.367	0.068	-0.163	0.592	*hsdM1*	Defense Mechanisms	Unknown
RCAP_rcc00953	0.101	0.739	-0.315	0.183	-0.192	0.512	*hypothetical protein*	Unknown	Unknown
RCAP_rcc00954	0.227	0.405	-0.567	0.003	-0.171	0.526	*hsdS1*	Defense Mechanisms	Unknown
RCAP_rcc00955	0.269	0.326	-0.703	0.000	-0.134	0.641	*SMC protein, N-terminal domain-containing protein*	Unknown	Unknown
RCAP_rcc00956	0.300	0.283	-0.534	0.012	-0.171	0.566	*hypothetical protein*	Unknown	Unknown
RCAP_rcc00957	-0.113	0.728	-0.334	0.338	-0.308	0.353	*hypothetical protein*	Unknown	Unknown
RCAP_rcc00960	-0.034	0.926	0.056	0.867	-0.457	0.124	*IS111A/IS1328/IS1533 family transposase/IS116/IS110/IS902 family trans*	Replication, Recombination and Repair	Recombination
RCAP_rcc00961	-0.395	0.055	-0.156	0.338	0.340	0.091	*hypothetical protein*	Unknown	Unknown
RCAP_rcc00962	-0.195	0.585	-0.456	0.223	0.453	0.203	*hypothetical protein*	Unknown	Unknown
RCAP_rcc00963	0.292	0.177	-0.109	0.496	-0.061	0.846	*hypothetical protein*	Unknown	Unknown
RCAP_rcc00964	0.084	0.789	-1.150	0.000	0.010	0.984	*phage tail fiber protein*	Replication, Recombination and Repair	Phage Interaction
RCAP_rcc00965	0.018	0.971	-1.858	0.000	-0.300	0.504	*phage tail assembly protein*	Replication, Recombination and Repair	Phage Interaction
RCAP_rcc00966	0.281	0.531	-1.556	0.000	-0.128	0.778	*hypothetical protein*	Unknown	Unknown
RCAP_rcc00967	-0.123	NA	-1.539	0.008	0.021	NA	*hypothetical protein*	Unknown	Unknown
RCAP_rcc00968	0.245	0.464	-1.497	0.000	-0.248	0.531	*hypothetical protein*	Unknown	Unknown
RCAP_rcc00969	0.032	NA	-1.660	0.000	0.085	0.871	*hypothetical protein*	Unknown	Unknown
RCAP_rcc00970	0.472	0.261	-2.493	0.000	0.087	0.825	*hypothetical protein*	Unknown	Unknown
RCAP_rcc00971	0.342	0.283	-1.633	0.000	-0.067	0.890	*hypothetical protein*	Unknown	Unknown
RCAP_rcc00972	-0.162	NA	-2.118	0.000	0.204	NA	*hypothetical protein*	Unknown	Unknown
RCAP_rcc00973	-0.062	0.905	-2.147	0.000	0.289	0.451	*hypothetical protein*	Unknown	Unknown
RCAP_rcc00974	0.136	NA	-2.224	0.000	0.183	0.704	*hypothetical protein*	Unknown	Unknown
RCAP_rcc00975	0.858	0.022	-1.664	0.000	0.011	0.985	*cyclic nucleotide-binding domain-containing protein*	Unknown	Unknown
RCAP_rcc00976	0.091	0.850	-2.280	0.000	0.234	0.560	*hypothetical protein*	Unknown	Unknown
RCAP_rcc00977	0.669	0.043	-2.175	0.000	-0.022	0.966	*hypothetical protein*	Unknown	Unknown
RCAP_rcc00978	-0.099	NA	-2.148	0.000	0.684	0.050	*hypothetical protein*	Unknown	Unknown
RCAP_rcc00979	0.402	0.273	-2.311	0.000	0.063	0.888	*hypothetical protein*	Unknown	Unknown
RCAP_rcc00980	0.487	0.148	-1.042	0.000	0.150	0.718	*phage virion morphogenesis protein*	Replication, Recombination and Repair	Phage Interaction
RCAP_rcc00981	0.858	0.005	-0.400	0.056	-0.937	0.000	*phage head morphogenesis protein*	Replication, Recombination and Repair	Phage Interaction
RCAP_rcc00982	-0.176	0.647	-1.209	0.000	-0.097	0.818	*hypothetical protein*	Unknown	Unknown
RCAP_rcc00983	0.464	0.267	-1.253	0.004	-0.215	0.633	*hypothetical protein*	Unknown	Unknown
RCAP_rcc00984	0.584	0.082	-0.874	0.007	-0.788	0.003	*hypothetical protein*	Unknown	Unknown
RCAP_rcc00985	0.326	0.385	-1.550	0.000	-0.252	0.549	*hypothetical protein*	Unknown	Unknown
RCAP_rcc00986	0.507	0.157	-1.976	0.000	-0.115	0.808	*hypothetical protein*	Unknown	Unknown
RCAP_rcc00987	-0.009	0.984	-1.858	0.000	-0.042	0.944	*hypothetical protein*	Unknown	Unknown
RCAP_rcc00988	0.138	0.768	-2.043	0.000	0.129	0.750	*hypothetical protein*	Unknown	Unknown
RCAP_rcc00989	0.697	0.046	-1.956	0.000	-0.181	0.652	*lysozyme*	Unknown	Unknown
RCAP_rcc00990	0.064	0.841	-0.524	0.033	0.132	0.667	*hypothetical protein*	Unknown	Unknown
RCAP_rcc00991	0.740	0.024	-0.395	0.205	-0.201	0.648	*hypothetical protein*	Unknown	Unknown
RCAP_rcc00992	-0.211	NA	-0.346	0.648	0.001	NA	*hypothetical protein*	Unknown	Unknown
RCAP_rcc00993	0.010	0.984	-0.510	0.258	-0.190	0.689	*hypothetical protein*	Unknown	Unknown
RCAP_rcc00994	0.275	0.526	-0.521	0.269	-0.186	0.700	*hypothetical protein*	Unknown	Unknown
RCAP_rcc00995	0.612	0.014	-0.628	0.002	-0.178	0.612	*hypothetical protein*	Unknown	Unknown
RCAP_rcc00996	0.518	0.074	-0.528	0.051	-0.124	0.764	*hup2*	Energy Metabolism	Aerobic/Anaerobic Respiration
RCAP_rcc00997	0.125	0.756	-0.200	0.594	-0.018	0.977	*hypothetical protein*	Unknown	Unknown
RCAP_rcc00998	0.825	0.001	-0.450	0.086	-0.209	0.582	*gam*	Unknown	Unknown
RCAP_rcc00999	0.513	0.220	-0.356	0.472	0.124	0.801	*hypothetical protein*	Unknown	Unknown
RCAP_rcc01000	0.399	0.294	-0.423	0.359	-0.185	0.670	*hypothetical protein*	Unknown	Unknown
RCAP_rcc01001	0.565	0.031	-0.290	0.275	-0.397	0.197	*bacteriophage DNA transposition B protein*	Replication, Recombination and Repair	Phage Interaction

RCAP_rcc01002	0.384	0.056	-0.037	0.885	-0.347	0.178	*integrase catalytic subunit*	Replication, Recombination and Repair	Recombination
RCAP_rcc01003	0.428	0.253	-0.379	0.415	-0.393	0.347	*hypothetical protein*	Unknown	Unknown
RCAP_rcc01004	0.386	0.266	-0.411	0.206	-0.004	0.991	*ParB domain-containing protein nuclease*	Cell Division	Chromosome Partitioning
RCAP_rcc01005	-0.130	NA	-0.520	0.337	0.253	NA	*hypothetical protein*	Unknown	Unknown
RCAP_rcc01006	0.531	0.185	-0.814	0.034	0.127	0.791	*hypothetical protein*	Unknown	Unknown
RCAP_rcc01007	0.088	NA	-0.893	0.105	0.081	0.881	*hypothetical protein*	Unknown	Unknown
RCAP_rcc01008	0.225	NA	0.168	0.786	0.217	0.628	*lipoprotein*	Predicted Function	Unknown
RCAP_rcc01009	0.146	0.756	-0.850	0.014	0.153	0.750	*hypothetical protein*	Unknown	Unknown
RCAP_rcc01010	0.571	0.004	-0.107	0.672	-0.234	0.526	*DNA binding protein*	Signal Transduction	Transcription Regulator
RCAP_rcc01011	0.201	0.338	0.084	0.690	-0.175	0.504	*S24 family peptidase*	Post-translational Modification, Assembly and Chaperones	Peptidase
RCAP_rcc01012	0.708	0.001	-0.021	0.941	-0.790	0.000	*hypothetical protein*	Unknown	Unknown
RCAP_rcc01013	0.649	0.003	-0.043	0.810	-0.581	0.006	*hypothetical protein*	Unknown	Unknown
RCAP_rcc01014	0.459	0.026	-0.160	0.279	-0.357	0.076	*hypothetical protein*	Unknown	Unknown
RCAP_rcc01015	0.426	0.059	-0.187	0.337	-0.272	0.460	*hypothetical protein*	Unknown	Unknown
RCAP_rcc01019	-0.521	0.215	-1.009	0.006	0.499	0.220	*PHP domain-containing protein*	Unknown	Unknown
RCAP_rcc01020	-0.179	0.604	2.586	0.000	-0.173	0.538	*diguanylate cyclase/phosphodiesterase*	Signal Transduction	Kinase/Phosphorelay
RCAP_rcc01021	-0.626	0.078	0.425	0.338	-0.043	0.948	*ugpC*	Amino Acid Metabolism	Unknown
RCAP_rcc01022	0.058	0.880	0.535	0.077	-0.472	0.178	*ugpE*	Metal and Ion Transport	Unknown
RCAP_rcc01023	-0.262	0.431	-0.163	0.641	0.198	0.652	*ugpA*	Metal and Ion Transport	Unknown
RCAP_rcc01024	-0.228	0.540	0.410	0.179	-0.298	0.467	*ugpB*	Carbohydrate Metabolism	Unknown
RCAP_rcc01025	-0.541	0.198	-0.377	0.142	0.568	0.139	*regA2*	Signal Transduction	Transcription Regulator
RCAP_rcc01026	-0.420	0.277	-0.231	0.267	0.325	0.251	*sensor histidine kinase*	Signal Transduction	Kinase/Phosphorelay
RCAP_rcc01027	-0.713	0.079	-3.330	0.000	0.627	NA	*hypothetical protein*	Unknown	Unknown
RCAP_rcc01028	0.434	0.304	-2.474	0.000	-0.184	0.702	*iron siderophore/cobalamin ABC transporter periplasmic iron siderophor*	Metal, Ion, Cofactor Transport	Iron and Heme Transport
RCAP_rcc01029	-0.095	NA	-1.586	0.002	-0.300	NA	*iron siderophore/cobalamin ABC transporter permease*	Metal, Ion, Cofactor Transport	Iron and Heme Transport
RCAP_rcc01030	-0.286	NA	-1.438	0.011	0.083	NA	*iron siderophore/cobalamin ABC transporter permease*	Metal, Ion, Cofactor Transport	Iron and Heme Transport
RCAP_rcc01031	-0.569	NA	1.033	0.034	0.243	NA	*iron siderophore/cobalamin ABC transporter ATP-binding protein*	Metal, Ion, Cofactor Transport	Iron and Heme Transport
RCAP_rcc01032	-0.081	0.849	1.785	0.000	0.478	0.207	*cbiO1*	Metabolism of Cofactors, Coenzymes and Vitamins	Cobalamin Biosynthesis
RCAP_rcc01033	-0.298	0.420	1.464	0.000	0.948	0.001	*cbiQ1*	Metabolism of Cofactors, Coenzymes and Vitamins	Cobalamin Biosynthesis
RCAP_rcc01034	0.594	0.004	1.279	0.000	0.229	0.394	*CbiM family cobalamin biosynthesis protein*	Metabolism of Cofactors, Coenzymes and Vitamins	Cobalamin Biosynthesis
RCAP_rcc01035	0.419	0.101	-0.985	0.000	-0.443	0.151	*hypothetical protein*	Unknown	Unknown
RCAP_rcc01036	-0.171	NA	-1.238	0.062	0.045	NA	*hypothetical protein*	Unknown	Unknown
RCAP_rcc01037	0.055	NA	-0.362	0.576	0.138	NA	*hypothetical protein*	Unknown	Unknown
RCAP_rcc01038	0.657	0.036	-0.632	0.001	-0.728	0.000	*hypothetical protein*	Unknown	Unknown
RCAP_rcc01039	-0.426	0.310	1.016	0.019	0.153	0.752	*hypothetical protein*	Unknown	Unknown
RCAP_rcc01040	-0.551	0.175	0.767	0.051	0.544	0.170	*hypothetical protein*	Unknown	Unknown
RCAP_rcc01041	-0.411	0.133	1.001	0.000	0.136	0.746	*hypothetical protein*	Unknown	Unknown
RCAP_rcc01042	-0.623	0.057	0.919	0.000	0.316	0.468	*copA1*	Metal, Ion, Cofactor Transport	Copper Transport
RCAP_rcc01043	-0.316	0.416	-0.128	0.575	0.002	0.995	*hypothetical protein*	Unknown	Unknown
RCAP_rcc01044	-0.340	0.426	-0.793	0.007	0.177	0.712	*RimK-like ATP-grasp domain-containing protein*	Unknown	Unknown
RCAP_rcc01045	-0.234	0.602	-0.046	0.926	0.005	0.991	*iron siderophore/cobalamin ABC transporter ATP-binding protein*	Metal, Ion, Cofactor Transport	Iron and Heme Transport
RCAP_rcc01046	-0.096	0.837	0.355	0.336	0.074	0.894	*iron siderophore/cobalamin ABC transporter permease*	Metal, Ion, Cofactor Transport	Iron and Heme Transport
RCAP_rcc01047	-0.298	0.422	0.349	0.228	-0.030	0.964	*iron siderophore/cobalamin ABC transporter periplasmic iron siderophor*	Metal, Ion, Cofactor Transport	Iron and Heme Transport
RCAP_rcc01048	0.071	0.887	-0.267	0.592	-0.002	0.995	*AraC family transcriptional regulator*	Signal Transduction	Transcription Regulator
RCAP_rcc01049	-0.051	0.893	0.138	0.623	-0.238	0.607	*TonB-dependent siderophore receptor*	Metal, Ion, Cofactor Transport	Iron and Heme Transport
RCAP_rcc01050	0.107	NA	-0.050	0.953	-0.034	NA	*fes*	Metal and Ion Transport	Unknown
RCAP_rcc01051	-0.823	0.000	2.880	0.000	0.289	0.432	*gvpN*	Motility	Gas Vesicle
RCAP_rcc01052	-0.491	NA	1.501	0.006	0.121	NA	*hypothetical protein*	Unknown	Unknown
RCAP_rcc01053	-0.370	0.256	2.815	0.000	-0.132	0.787	*gvpO*	Motility	Gas Vesicle
RCAP_rcc01054	-0.946	0.001	2.613	0.000	0.228	0.613	*gvpJ*	Motility	Gas Vesicle
RCAP_rcc01055	-0.616	0.134	2.192	0.000	0.327	NA	*hypothetical protein*	Unknown	Unknown
RCAP_rcc01056	-1.209	0.000	2.555	0.000	0.194	0.683	*gas vesicle synthesis protein GvpL/GvpF*	Motility	Gas Vesicle
RCAP_rcc01057	-0.465	0.127	2.292	0.000	0.134	0.775	*gvpG*	Motility	Gas Vesicle
RCAP_rcc01058	-0.707	0.082	1.847	0.000	0.305	NA	*gas vesicle synthesis protein GvpL/GvpF*	Motility	Gas Vesicle
RCAP_rcc01059	-0.838	NA	1.133	0.036	0.193	NA	*gas vesicle synthesis protein GvpL/GvpF*	Motility	Gas Vesicle
RCAP_rcc01060	-0.091	0.838	1.696	0.002	-0.064	0.912	*gas vesicle protein GvpA*	Motility	Gas Vesicle
RCAP_rcc01061	-0.679	0.085	1.123	0.001	-0.028	0.965	*hypothetical protein*	Unknown	Unknown
RCAP_rcc01062	0.081	0.817	1.176	0.003	-0.015	0.982	*gvpK*	Motility	Gas Vesicle
RCAP_rcc01063	-0.742	0.008	2.098	0.000	0.410	0.267	*pcl*	Lipid Metabolism	Unknown
RCAP_rcc01064	-0.558	0.069	2.344	0.000	0.475	0.133	*hypothetical protein*	Unknown	Unknown
RCAP_rcc01065	-0.179	0.641	2.271	0.000	0.478	0.182	*hypothetical protein*	Unknown	Unknown
RCAP_rcc01066	0.074	0.836	2.383	0.000	0.422	0.215	*pyp*	Unknown	Unknown
RCAP_rcc01067	0.121	0.733	2.495	0.000	0.214	0.628	*hypothetical protein*	Unknown	Unknown
RCAP_rcc01068	0.398	0.258	-0.548	0.231	-0.550	0.062	*hypothetical protein*	Unknown	Unknown
RCAP_rcc01069	-0.179	0.682	-0.643	0.263	-0.637	0.084	*hypothetical protein*	Unknown	Unknown
RCAP_rcc01070	-0.819	0.036	2.264	0.000	0.220	0.616	*hypothetical protein*	Unknown	Unknown
RCAP_rcc01071	-0.419	0.279	1.847	0.000	0.546	0.180	*hypothetical protein*	Unknown	Unknown
RCAP_rcc01072	-0.881	0.005	3.090	0.000	0.628	0.072	*hutH*	Amino Acid Metabolism	Histidine metabolism
RCAP_rcc01073	0.098	0.747	3.201	0.000	0.133	0.780	*gvpA*	Motility	Gas Vesicle
RCAP_rcc01074	-0.239	0.589	1.966	0.001	0.219	NA	*hypothetical protein*	Unknown	Unknown
RCAP_rcc01075	0.093	0.767	2.538	0.000	0.296	0.455	*methyl-accepting chemotaxis protein*	Motility	Chemotaxis
RCAP_rcc01076	-0.473	0.082	3.216	0.000	0.435	0.171	*hypothetical protein*	Unknown	Unknown
RCAP_rcc01077	-0.403	0.348	0.282	0.401	-0.150	0.757	*hypothetical protein*	Unknown	Unknown
RCAP_rcc01078	0.139	0.632	-0.063	0.795	-0.300	0.306	*SH3 domain-containing protein*	Unknown	Unknown
RCAP_rcc01079	-0.230	0.589	2.239	0.000	-0.223	0.631	*hypothetical protein*	Unknown	Unknown
RCAP_rcc01080	-0.714	0.026	2.423	0.000	-0.114	0.815	*hypothetical protein*	Unknown	Unknown
RCAP_rcc01081	0.020	0.915	-0.074	0.707	-0.282	0.157	*group 1 glycosyl transferase*	Cell Envelope Biosynthesis	Cell Wall Biosynthesis

RCAP_rcc01082	-0.078	0.755	-0.056	0.845	-0.115	0.558	*group 1 glycosyl transferase*	Cell Envelope Biosynthesis	Cell Wall Biosynthesis
RCAP_rcc01083	-0.057	0.825	0.119	0.669	-0.077	0.705	*lspL1*	Carbohydrate Metabolism	Amino sugar and nucleotide sugar metabolism
RCAP_rcc01084	0.114	0.624	-0.319	0.005	0.317	0.225	*hypothetical protein*	Unknown	Unknown
RCAP_rcc01085	0.350	0.185	-0.402	0.043	-0.132	0.715	*hypothetical protein*	Unknown	Unknown
RCAP_rcc01086	-0.316	0.133	-0.267	0.046	0.224	0.465	*family 2 glycosyl transferase*	Cell Envelope Biosynthesis	Cell Wall Biosynthesis
RCAP_rcc01087	-0.422	0.164	0.093	0.801	-0.335	0.267	*pip2*	Amino Acid Metabolism	Arginine and proline metabolism
RCAP_rcc01088	0.391	0.082	-0.427	0.010	0.020	0.963	*LuxR family autoinducer-binding transcriptional regulator*	Signal Transduction	Transcription Regulator
RCAP_rcc01089	0.429	0.044	-0.712	0.000	0.052	0.902	*gsiB*	Sulfur Metabolism	Glutathione metabolism
RCAP_rcc01090	-0.115	0.670	-0.249	0.211	0.145	0.661	*gsiD*	Sulfur Metabolism	Glutathione metabolism
RCAP_rcc01091	0.099	0.717	-0.348	0.134	0.100	0.778	*gsiC*	Sulfur Metabolism	Glutathione metabolism
RCAP_rcc01092	-0.069	0.826	-0.402	0.105	0.075	0.852	*gsiA*	Sulfur Metabolism	Glutathione metabolism
RCAP_rcc01093	0.037	0.901	-0.495	0.005	0.351	0.241	*pepA1*	Metabolism of Other Amino Acids	Glutathione metabolism
RCAP_rcc01094	0.093	0.784	0.041	0.870	-0.188	0.611	*flavin-nucleotide-binding protein*	Unknown	Unknown
RCAP_rcc01095	-1.035	0.009	-0.353	0.253	0.248	0.545	*GntR family transcriptional regulator*	Signal Transduction	Transcription Regulator
RCAP_rcc01096	0.099	0.657	-1.678	0.000	0.052	0.845	*abgB*	Amino Acid Metabolism	Amino Acid Transport
RCAP_rcc01097	-0.582	0.106	0.280	0.133	0.426	0.205	*hypothetical protein*	Unknown	Unknown
RCAP_rcc01099	0.251	0.341	0.056	0.865	-0.387	0.284	*IS4 family transposase*	Replication, Recombination and Repair	Recombination
RCAP_rcc01100	-0.268	0.514	1.119	0.000	0.150	0.731	*XRE family transcriptional regulator*	Signal Transduction	Transcription Regulator
RCAP_rcc01101	0.177	0.597	0.636	0.000	-0.046	0.902	*HipA domain-containing protein*	Unknown	Unknown
RCAP_rcc01102	-0.060	0.885	0.572	0.018	0.417	0.305	*hypothetical protein*	Unknown	Unknown
RCAP_rcc01103	-0.044	0.926	0.063	0.915	-0.200	0.672	*hypothetical protein*	Unknown	Unknown
RCAP_rcc01104	-0.311	0.476	0.470	0.425	-0.046	0.923	*hypothetical protein*	Unknown	Unknown
RCAP_rcc01105	-0.103	0.835	0.084	0.893	0.095	NA	*hypothetical protein*	Unknown	Unknown
RCAP_rcc01107	0.252	0.385	0.056	0.831	-0.115	0.697	*hypothetical protein*	Unknown	Unknown
RCAP_rcc01108	-0.813	0.043	-0.364	0.412	0.322	0.477	*pyrroline-5-carboxylate reductase*	Amino Acid Metabolism	Arginine and proline metabolism
RCAP_rcc01109	0.105	0.655	-0.855	0.000	0.100	0.670	*rsbQ*	Signal Transduction	Transcription Regulator
RCAP_rcc01110	-0.802	0.015	-1.469	0.000	0.662	NA	*diguanylate cyclase/phosphodiesterase*	Signal Transduction	Kinase/Phosphorelay
RCAP_rcc01111	-0.146	0.749	-0.320	0.211	0.167	0.705	*hypothetical protein*	Unknown	Unknown
RCAP_rcc01112	0.941	0.005	-0.273	0.458	-0.510	0.216	*hypothetical protein*	Unknown	Unknown
RCAP_rcc01113	0.066	0.877	0.198	0.332	-0.013	0.974	*MiaB family RNA modification enzyme*	Translation, ribosomal structure and biogenesis	Unknown
RCAP_rcc01114	-0.654	0.006	-0.820	0.000	0.446	0.109	*OmpA/MotB domain-containing protein*	Unknown	Unknown
RCAP_rcc01115	-0.618	0.026	-1.075	0.000	0.771	0.016	*lipoprotein*	Predicted Function	Unknown
RCAP_rcc01116	0.059	0.849	-1.186	0.000	-0.081	0.736	*PhoH family protein*	Signal Transduction	Kinase/Phosphorelay
RCAP_rcc01117	0.106	0.705	0.100	0.601	-0.205	0.428	*hypothetical protein*	Unknown	Unknown
RCAP_rcc01118	-0.111	0.686	0.185	0.483	-0.328	0.007	*corC*	Metal, Ion, Cofactor Transport	Cobalt Transport
RCAP_rcc01119	-0.616	0.005	0.126	0.531	0.225	0.530	*lnt*	Cell Envelope Biosynthesis	Cell Wall Biosynthesis
RCAP_rcc01120	0.445	0.006	1.023	0.000	-0.069	0.781	*metK*	Amino Acid Metabolism	Cysteine and methionine metabolism
RCAP_rcc01121	0.026	0.957	0.737	0.000	-0.009	0.981	*trmB*	Unknown	Unknown
RCAP_rcc01122	-0.113	0.747	-0.150	0.420	0.470	0.110	*aroA*	Amino Acid Metabolism	Phenylalanine, tyrosine and tryptophan biosynthesis
RCAP_rcc01123	0.062	0.812	-0.068	0.722	0.155	0.626	*cmk*	Nucleotide Metabolism	Pyrimidine metabolism
RCAP_rcc01124	0.242	0.131	-0.123	0.394	-0.233	0.249	*nfnB*	Energy Metabolism	Unknown
RCAP_rcc01125	0.424	0.043	0.226	0.245	-0.286	0.367	*rpsA*	Translation, ribosomal structure and biogenesis	Unknown
RCAP_rcc01126	0.501	0.018	-0.078	0.609	-0.280	0.395	*ihfB*	Transcription	Unknown
RCAP_rcc01127	0.407	0.058	-0.020	0.896	-0.174	0.595	*hypothetical protein*	Unknown	Unknown
RCAP_rcc01128	-1.148	0.000	0.146	0.382	0.754	0.044	*trpF*	Amino Acid Metabolism	Phenylalanine, tyrosine and tryptophan biosynthesis
RCAP_rcc01129	0.323	0.050	0.377	0.001	-0.177	0.451	*trpB1*	Amino Acid Metabolism	Phenylalanine, tyrosine and tryptophan biosynthesis
RCAP_rcc01130	0.964	0.000	-1.702	0.000	0.108	0.746	*LuxR family transcriptional regulator*	Signal Transduction	Transcription Regulator
RCAP_rcc01131	0.073	0.792	-0.661	0.001	0.315	0.446	*MarR family transcriptional regulator*	Signal Transduction	Transcription Regulator
RCAP_rcc01132	-0.707	0.002	-0.362	0.075	0.521	0.183	*galM*	Carbohydrate Metabolism	Glycolysis / Gluconeogenesis
RCAP_rcc01133	0.069	0.874	0.557	0.018	0.204	0.625	*znuA*	Metal, Ion, Cofactor Transport	Zinc Transport
RCAP_rcc01134	-0.080	0.835	0.254	0.347	-0.151	0.721	*Fur family transcriptional regulator*	Signal Transduction	Transcription Regulator
RCAP_rcc01135	-0.473	0.220	-0.019	0.946	0.481	0.248	*znuC*	Metal, Ion, Cofactor Transport	Zinc Transport
RCAP_rcc01136	-0.993	0.007	-0.062	0.758	0.638	0.109	*znuB*	Metal, Ion, Cofactor Transport	Zinc Transport
RCAP_rcc01137	-0.467	0.124	0.071	0.803	0.557	0.081	*pfkB*	Carbohydrate Metabolism	Glycolysis / Gluconeogenesis
RCAP_rcc01138	-1.073	0.000	2.781	0.000	0.425	0.265	*hypothetical protein*	Unknown	Unknown
RCAP_rcc01139	-0.669	0.000	2.558	0.000	0.504	0.150	*hypothetical protein*	Unknown	Unknown
RCAP_rcc01140	-0.580	0.009	0.764	0.000	0.144	0.713	*hypothetical protein*	Unknown	Unknown
RCAP_rcc01141	0.151	0.531	0.090	0.584	-0.030	0.894	*gltX1*	Translation, ribosomal structure and biogenesis	Aminoacyl-tRNA biosynthesis
RCAP_rcc01142	-0.203	0.449	0.309	0.090	0.199	0.477	*gcvT1*	Energy Metabolism	Nitrogen metabolism
RCAP_rcc01143	0.446	0.125	0.165	0.430	-0.081	NA	*gcvH*	Carbohydrate Metabolism	Glyoxylate and dicarboxylate metabolism
RCAP_rcc01144	-0.043	0.888	0.137	0.482	0.140	0.620	*gcvP*	Amino Acid Metabolism	Glycine, serine and threonine metabolism
RCAP_rcc01145	0.374	0.016	-0.190	0.093	-0.203	0.453	*fabH1*	Lipid Metabolism	Biotin metabolism
RCAP_rcc01146	-0.261	0.173	-0.536	0.000	0.385	0.056	*gyaR2*	Energy Metabolism	Methane metabolism
RCAP_rcc01147	0.195	0.310	-0.253	0.178	-0.008	0.982	*csaA*	Unknown	Unknown
RCAP_rcc01148	-0.392	0.147	-0.206	0.431	0.546	0.101	*proC*	Amino Acid Metabolism	Arginine and proline metabolism
RCAP_rcc01149	0.410	0.116	0.314	0.256	-0.208	0.471	*hypothetical protein*	Unknown	Unknown
RCAP_rcc01150	-0.084	0.812	-0.079	0.651	-0.041	0.877	*aroC*	Amino Acid Metabolism	Phenylalanine, tyrosine and tryptophan biosynthesis
RCAP_rcc01151	-0.347	0.099	0.374	0.146	0.022	0.946	*hypothetical protein*	Unknown	Unknown
RCAP_rcc01152	0.062	0.838	0.010	0.952	0.007	0.984	*hisE*	Amino Acid Metabolism	Histidine metabolism
RCAP_rcc01153	-0.143	0.626	0.276	0.078	0.090	0.707	*hisF*	Amino Acid Metabolism	Histidine metabolism
RCAP_rcc01154	-0.096	0.806	0.113	0.721	0.026	0.958	*hisA*	Amino Acid Metabolism	Histidine metabolism
RCAP_rcc01155	0.266	0.316	-1.318	0.000	0.018	0.960	*hypothetical protein*	Unknown	Unknown
RCAP_rcc01156	2.384	0.000	-3.674	0.000	-0.074	0.871	*UspA domain-containing protein*	Stress Response	Unknown
RCAP_rcc01157	0.632	0.015	-3.554	0.000	-0.012	0.981	*ccoN*	Energy Metabolism	Aerobic/Anaerobic Respiration
RCAP_rcc01158	0.416	0.094	-3.556	0.000	0.186	0.566	*ccoO*	Energy Metabolism	Aerobic/Anaerobic Respiration
RCAP_rcc01159	0.492	0.075	-3.599	0.000	0.026	0.964	*ccoQ*	Energy Metabolism	Aerobic/Anaerobic Respiration
RCAP_rcc01160	0.348	0.156	-3.369	0.000	-0.017	0.961	*ccoP*	Energy Metabolism	Aerobic/Anaerobic Respiration

RCAP_rcc01161	0.399	0.117	-2.149	0.000	-0.176	0.628	*ccoG*	Energy Metabolism	Aerobic/Anaerobic Respiration
RCAP_rcc01162	0.359	0.146	-1.455	0.000	-0.050	0.888	*ccoH*	Energy Metabolism	Aerobic/Anaerobic Respiration
RCAP_rcc01163	-0.582	0.003	-1.423	0.000	0.404	0.066	*ccoI*	Energy Metabolism	Aerobic/Anaerobic Respiration
RCAP_rcc01164	0.438	0.102	-0.867	0.000	-0.147	0.619	*ccoS*	Energy Metabolism	Aerobic/Anaerobic Respiration
RCAP_rcc01165	-0.043	0.826	-0.073	0.598	-0.153	0.382	*dacC1*	Glycan Biosynthesis and Metabolism	Peptidoglycan biosynthesis
RCAP_rcc01166	-0.314	0.127	-0.276	0.114	0.237	0.301	*HAD superfamily hydrolase*	Unknown	Unknown
RCAP_rcc01167	0.460	0.061	-0.128	0.361	-0.409	0.100	*clpS*	Post-translational Modification, Assembly and Chaperones	Unknown
RCAP_rcc01168	-0.271	0.227	0.123	0.400	0.083	0.809	*methyltransferase small domain-containing protein*	Translation, ribosomal structure and biogenesis	Unknown
RCAP_rcc01169	0.633	0.004	-0.210	0.281	-0.330	0.254	*lipoprotein*	Predicted Function	Unknown
RCAP_rcc01170	-0.606	NA	1.253	0.040	-0.187	NA	*hypothetical protein*	Unknown	Unknown
RCAP_rcc01171	-0.389	0.239	0.387	0.002	0.225	0.408	*ATP-dependent RNA helicase DbpA*	Replication, Recombination and Repair	Unknown
RCAP_rcc01172	0.040	0.855	1.456	0.000	-0.070	0.652	*hemE*	Metabolism of Cofactors, Coenzymes and Vitamins	Heme Biosynthesis
RCAP_rcc01173	-0.013	0.966	0.926	0.000	0.116	0.558	*hemC*	Metabolism of Cofactors, Coenzymes and Vitamins	Heme Biosynthesis
RCAP_rcc01174	-0.067	0.751	0.836	0.000	0.116	0.665	*D-2-hydroxyglutarate dehydrogenase*	Energy Metabolism	Unknown
RCAP_rcc01175	-0.251	0.276	-0.138	0.431	0.286	0.279	*major facilitator superfamily protein*	Metal and Ion Transport	Unknown
RCAP_rcc01176	-0.275	0.395	-0.426	0.042	0.238	0.561	*GDSL-like lipase/acylhydrolase*	Xenobiotics Biodegradation and Metabolism	Bisphenol A degradation
RCAP_rcc01177	0.446	0.099	0.516	0.005	-0.678	0.046	*ABC transporter ATP-binding protein*	Secondary metabolites biosynthesis, transport, and catabolism	Unknown
RCAP_rcc01178	-0.373	0.176	-0.006	0.984	0.252	0.482	*hypothetical protein*	Unknown	Unknown
RCAP_rcc01179	0.874	0.000	-0.626	0.001	0.024	0.965	*transglycosylase, Slt family*	Cell Envelope Biosynthesis	Cell Wall Biosynthesis
RCAP_rcc01180	-0.490	0.039	-0.503	0.001	0.283	0.480	*copA2*	Metal, Ion, Cofactor Transport	Copper Transport
RCAP_rcc01181	0.382	0.071	-0.459	0.009	-0.784	0.000	*hypothetical protein*	Unknown	Unknown
RCAP_rcc01182	-0.064	0.817	-0.343	0.024	-0.241	0.244	*hisH*	Nucleotide Metabolism	Purine metabolism
RCAP_rcc01183	0.600	0.001	0.033	0.864	-0.325	0.225	*hisB*	Amino Acid Metabolism	Histidine metabolism
RCAP_rcc01184	0.700	0.006	-1.180	0.000	-0.877	0.000	*CsbD family protein*	Unknown	Unknown
RCAP_rcc01185	-0.401	0.273	1.506	0.000	-0.463	0.268	*methyl-accepting chemotaxis sensory transducer*	Motility	Chemotaxis
RCAP_rcc01186	-0.328	0.322	0.117	0.731	-0.299	0.441	*hypothetical protein*	Unknown	Unknown
RCAP_rcc01187	-0.091	NA	-0.350	0.650	-0.250	NA	*phnC*	Metal and Ion Transport	Unknown
RCAP_rcc01188	0.180	0.602	0.466	0.229	-0.631	0.046	*phnD*	Metal and Ion Transport	Unknown
RCAP_rcc01189	0.042	0.934	0.401	0.528	-0.090	0.866	*phnE1*	Metal and Ion Transport	Unknown
RCAP_rcc01190	-0.066	0.884	0.424	0.258	-0.827	0.012	*phnE2*	Metal and Ion Transport	Unknown
RCAP_rcc01191	0.163	0.702	0.157	0.698	-0.365	0.402	*transferase hexapeptide repeat family protein*	Unknown	Unknown
RCAP_rcc01192	-0.056	0.908	-0.677	0.148	0.048	0.932	*phnF*	Signal Transduction	Transcription Regulator
RCAP_rcc01193	-0.123	NA	-0.188	0.808	-0.065	NA	*phnG*	Metal and Ion Transport	Unknown
RCAP_rcc01194	-0.669	NA	0.608	0.371	-0.040	NA	*phnH*	Metal and Ion Transport	Unknown
RCAP_rcc01195	0.247	0.334	0.742	0.006	-0.155	0.629	*phnI*	Metal and Ion Transport	Unknown
RCAP_rcc01197	0.058	0.903	0.344	0.466	-0.300	0.504	*phnJ*	Metal and Ion Transport	Unknown
RCAP_rcc01198	0.125	0.789	0.031	0.964	-0.547	0.174	*phnK*	Metal and Ion Transport	Unknown
RCAP_rcc01199	0.055	NA	-0.219	0.767	0.265	NA	*phnL*	Metal and Ion Transport	Unknown
RCAP_rcc01200	#VALUE!	NA	-0.001	0.998	#VALUE!	NA	*phnN*	Carbohydrate Metabolism	Pentose phosphate pathway
RCAP_rcc01201	0.120	NA	-0.715	0.246	-0.225	0.605	*hypothetical protein*	Unknown	Unknown
RCAP_rcc01202	-0.027	0.956	-0.221	0.651	-0.458	0.272	*phnM*	Metal and Ion Transport	Unknown
RCAP_rcc01203	0.438	0.036	-0.669	0.001	-0.149	0.605	*pyc*	Carbohydrate Metabolism	TCA Cycle
RCAP_rcc01204	0.072	0.744	-0.002	0.993	-0.069	0.750	*peptidoglycan binding domain-containing protein*	Unknown	Unknown
RCAP_rcc01205	0.805	0.003	0.190	0.399	-0.317	0.298	*glyQ*	Translation, ribosomal structure and biogenesis	Aminoacyl-tRNA biosynthesis
RCAP_rcc01206	-0.387	0.323	-0.055	0.857	-0.049	0.936	*hypothetical protein*	Unknown	Unknown
RCAP_rcc01207	-0.012	0.952	0.175	0.202	0.007	0.981	*glyS*	Translation, ribosomal structure and biogenesis	Aminoacyl-tRNA biosynthesis
RCAP_rcc01208	-0.404	0.018	-0.471	0.000	0.212	0.168	*ppdK*	Energy Metabolism	Carbon fixation in photosynthetic organisms
RCAP_rcc01209	0.430	0.046	0.686	0.000	0.010	0.975	*cell wall hydrolase, SleB*	Cell Envelope Biosynthesis	Cell Wall Biosynthesis
RCAP_rcc01210	0.028	0.909	-0.060	0.744	-0.134	0.691	*folB*	Metabolism of Cofactors, Coenzymes and Vitamins	Folate biosynthesis
RCAP_rcc01211	-0.378	0.164	0.064	0.743	0.394	0.323	*folP*	Metabolism of Cofactors, Coenzymes and Vitamins	Folate biosynthesis
RCAP_rcc01212	-0.262	0.144	-0.171	0.259	0.249	0.209	*glmM*	Carbohydrate Metabolism	Amino sugar and nucleotide sugar metabolism
RCAP_rcc01213	0.252	0.232	-0.124	0.617	-0.157	0.589	*hypothetical protein*	Unknown	Unknown
RCAP_rcc01214	0.738	0.000	-0.945	0.000	-0.123	0.688	*ilvC*	Metabolism of Cofactors, Coenzymes and Vitamins	Pantothenate and CoA biosynthesis
RCAP_rcc01215	0.145	0.683	0.189	0.573	-0.254	0.535	*AsnC/Lrp family transcriptional regulator*	Signal Transduction	Transcription Regulator
RCAP_rcc01216	-0.203	0.589	0.054	0.874	0.027	0.967	*AsnC/Lrp family transcriptional regulator*	Signal Transduction	Transcription Regulator
RCAP_rcc01217	-0.730	0.032	-0.233	0.650	-0.034	0.958	*ureD*	Energy Metabolism	Urea Cycle
RCAP_rcc01218	-0.435	0.276	-1.039	0.001	-0.142	0.764	*ureA*	Energy Metabolism	Urea Cycle
RCAP_rcc01219	-0.052	0.912	-1.312	0.000	-0.163	0.715	*ureB*	Energy Metabolism	Urea Cycle
RCAP_rcc01220	0.271	0.230	-0.708	0.000	-0.738	0.000	*ureC*	Energy Metabolism	Urea Cycle
RCAP_rcc01221	-0.361	0.360	1.732	0.000	0.299	0.511	*hypothetical protein*	Unknown	Unknown
RCAP_rcc01222	-0.583	0.125	-0.885	0.000	0.280	0.529	*ureE*	Energy Metabolism	Urea Cycle
RCAP_rcc01223	-0.022	0.957	-0.783	0.000	-0.441	0.153	*ureF*	Energy Metabolism	Urea Cycle
RCAP_rcc01224	-0.678	0.046	-0.769	0.000	0.160	0.730	*ureG*	Energy Metabolism	Urea Cycle
RCAP_rcc01225	-0.052	NA	0.199	0.795	0.088	NA	*hypothetical protein*	Unknown	Unknown
RCAP_rcc01226	0.614	0.039	0.629	0.056	-0.609	0.059	*urtB*	Energy Metabolism	Urea Cycle
RCAP_rcc01227	0.269	0.538	0.773	0.043	-0.597	0.135	*urtA*	Energy Metabolism	Urea Cycle
RCAP_rcc01228	-0.306	0.390	0.742	0.001	-0.071	0.888	*urtC*	Energy Metabolism	Urea Cycle
RCAP_rcc01229	0.371	0.383	-0.192	0.762	-0.101	0.845	*urtD*	Energy Metabolism	Urea Cycle
RCAP_rcc01230	0.454	0.043	0.023	0.935	-0.479	0.034	*urtE*	Energy Metabolism	Urea Cycle
RCAP_rcc01231	0.049	0.890	0.154	NA	0.111	0.775	*UbiH/UbiF/VisC/COQ6 family ubiquinone biosynthesis hydroxylase*	Energy Metabolism	Biosynthesis of Ubiquinone
RCAP_rcc01232	-0.050	0.887	5.581	0.000	-0.009	0.985	*hypothetical protein*	Unknown	Unknown
RCAP_rcc01233	0.123	0.616	2.561	0.000	-0.414	0.114	*hypothetical protein*	Unknown	Unknown
RCAP_rcc01234	0.013	0.977	-0.584	0.401	-0.358	0.272	*hypothetical protein*	Unknown	Unknown
RCAP_rcc01235	-0.649	0.039	2.011	0.000	0.060	0.899	*pyrimidine 5'-nucleotidase*	Metabolism of Cofactors, Coenzymes and Vitamins	Vitamin B6 metabolism
RCAP_rcc01236	0.177	0.453	0.511	NA	0.035	0.914	*GntR family transcriptional regulator*	Signal Transduction	Transcription Regulator
RCAP_rcc01237	-0.160	0.447	0.791	0.001	-0.230	0.493	*family 2 glycosyl transferase*	Cell Envelope Biosynthesis	Cell Wall Biosynthesis
RCAP_rcc01238	0.378	0.233	0.097	0.642	-0.158	0.645	*carA*	Amino Acid Metabolism	Alanine, aspartate and glutamate metabolism

RCAP_rcc01239	0.336	0.271	-0.314	0.137	-0.417	0.152	*glyoxalase/bleomycin resistance protein/dioxygenase*	Carbohydrate Metabolism	Pyruvate metabolism
RCAP_rcc01240	0.398	0.047	-0.243	0.357	0.114	0.700	*cycA1*	Energy Metabolism	Aerobic/Anaerobic Respiration
RCAP_rcc01241	-0.128	0.692	-0.485	0.005	0.422	0.171	*lipB*	Metabolism of Cofactors, Coenzymes and Vitamins	Unknown
RCAP_rcc01242	-0.071	0.812	2.327	0.000	-0.104	0.633	*hemolysin-type calcium-binding repeat family protein*	Trafficking and Secretion	Secretion
RCAP_rcc01243	0.436	0.132	1.013	0.003	-0.140	0.648	*potA1*	Amino Acid Metabolism	Amino Acid Transport
RCAP_rcc01244	0.404	0.155	0.980	0.010	0.059	0.799	*potD1*	Unknown	Unknown
RCAP_rcc01245	0.266	0.344	0.978	0.016	0.113	0.556	*potB1*	Amino Acid Metabolism	Amino Acid Transport
RCAP_rcc01246	0.205	0.461	0.834	0.010	0.011	0.970	*potI1*	Amino Acid Metabolism	Amino Acid Transport
RCAP_rcc01247	-0.467	0.034	0.076	0.763	0.360	0.183	*membrane transport family protein*	Unknown	Unknown
RCAP_rcc01248	-0.195	0.398	-0.049	0.750	0.065	0.878	*metallo-beta-lactamase*	Unknown	Unknown
RCAP_rcc01249	0.436	0.028	0.102	0.594	-0.173	0.589	*TatD-related deoxyribonuclease*	Trafficking and Secretion	Secretion
RCAP_rcc01250	-0.865	0.001	-0.358	0.042	0.241	0.550	*holB*	Replication, Recombination and Repair	Replication
RCAP_rcc01251	-1.025	0.000	-0.395	0.050	0.666	0.045	*tmk*	Nucleotide Metabolism	Pyrimidine metabolism
RCAP_rcc01252	0.055	0.807	-0.496	0.001	0.155	0.350	*dacC2*	Glycan Biosynthesis and Metabolism	Peptidoglycan biosynthesis
RCAP_rcc01253	-0.095	0.733	-0.469	0.017	0.140	0.640	*sporulation domain-containing protein*	Unknown	Unknown
RCAP_rcc01254	0.608	0.025	-0.764	0.000	-0.262	0.499	*phage integrase*	Replication, Recombination and Repair	Phage Interaction
RCAP_rcc01255	0.026	0.950	-0.112	0.731	-0.318	0.457	*hypothetical protein*	Unknown	Unknown
RCAP_rcc01256	-0.146	0.589	0.217	0.359	-0.461	0.129	*hypothetical protein*	Unknown	Unknown
RCAP_rcc01257	0.183	0.494	0.080	0.758	-0.242	0.526	*hypothetical protein*	Unknown	Unknown
RCAP_rcc01258	0.072	0.843	0.400	0.257	-0.108	0.799	*hypothetical protein*	Unknown	Unknown
RCAP_rcc01259	-0.228	0.543	-0.110	0.795	-0.347	0.414	*hypothetical protein*	Unknown	Unknown
RCAP_rcc01260	0.325	0.232	0.069	0.732	-0.252	0.441	*mod*	Replication, Recombination and Repair	Unknown
RCAP_rcc01261	0.197	0.480	-0.030	0.883	-0.149	0.627	*res*	Defense Mechanisms	Unknown
RCAP_rcc01262	0.371	0.103	-0.021	0.911	-0.456	0.074	*recombinase*	Replication, Recombination and Repair	Recombination
RCAP_rcc01263	0.405	0.043	-0.374	0.096	-0.277	0.390	*hypothetical protein*	Unknown	Unknown
RCAP_rcc01264	0.364	0.095	-0.432	0.098	-0.275	0.338	*reverse transcriptase*	Unknown	Unknown
RCAP_rcc01267	-0.530	0.124	-0.337	0.418	0.305	0.504	*hypothetical protein*	Unknown	Unknown
RCAP_rcc01268	1.274	0.000	-0.587	0.102	-0.579	0.154	*hypothetical protein*	Unknown	Unknown
RCAP_rcc01269	-0.197	0.574	0.549	0.025	0.273	0.457	*hypothetical protein*	Unknown	Unknown
RCAP_rcc01270	0.301	0.453	0.884	0.000	0.142	0.766	*XRE family transcriptional regulator*	Signal Transduction	Transcription Regulator
RCAP_rcc01271	0.211	0.381	0.228	0.234	-0.149	0.616	*hypothetical protein*	Unknown	Unknown
RCAP_rcc01272	-0.177	0.509	0.774	0.001	0.144	0.411	*hypothetical protein*	Unknown	Unknown
RCAP_rcc01273	-0.098	0.651	0.970	0.000	0.317	0.168	*hypothetical protein*	Unknown	Unknown
RCAP_rcc01274	0.262	0.301	0.592	0.001	-0.019	NA	*RAMP family CRISPR-associated protein*	Replication, Recombination and Repair	Unknown
RCAP_rcc01275	0.171	0.651	0.373	0.252	-0.050	0.905	*Cas2 family CRISPR-associated protein*	Unknown	Unknown
RCAP_rcc01276	-0.481	0.029	0.589	0.006	0.350	0.267	*Cas1 family CRISPR-associated protein*	Replication, Recombination and Repair	Unknown
RCAP_rcc01277	0.413	0.150	-0.174	0.527	-0.274	0.375	*RNA-directed DNA polymerase*	Replication, Recombination and Repair	Replication
RCAP_rcc01278	-0.358	0.236	-0.148	0.669	0.296	0.403	*hypothetical protein*	Unknown	Unknown
RCAP_rcc01279	-0.466	0.193	0.091	0.845	-0.211	0.641	*hypothetical protein*	Unknown	Unknown
RCAP_rcc01280	0.319	0.279	-0.050	0.850	0.127	0.745	*exonuclease*	Unknown	Unknown
RCAP_rcc01281	-0.148	0.698	-0.015	0.967	-0.123	0.798	*hypothetical protein*	Unknown	Unknown
RCAP_rcc01282	0.077	0.823	-0.381	0.061	0.077	0.872	*relaxase/mobilization nuclease domain-containing protein*	Unknown	Unknown
RCAP_rcc01283	0.213	0.620	-0.743	0.027	-0.211	0.651	*hypothetical protein*	Unknown	Unknown
RCAP_rcc01284	0.084	0.781	-0.199	0.412	-0.343	0.218	*hypothetical protein*	Unknown	Unknown
RCAP_rcc01285	0.189	0.435	-0.010	0.970	0.003	0.991	*GntR family transcriptional regulator*	Signal Transduction	Transcription Regulator
RCAP_rcc01286	0.364	0.083	-0.079	0.608	-0.305	0.324	*mcrB*	Defense Mechanisms	Unknown
RCAP_rcc01287	-0.151	0.589	-0.207	0.206	0.335	0.226	*mcrC*	Defense Mechanisms	Unknown
RCAP_rcc01288	-0.041	0.907	-0.132	0.610	-0.419	0.301	*hypothetical protein*	Unknown	Unknown
RCAP_rcc01289	-0.469	0.154	0.547	0.001	0.277	0.406	*hypothetical protein*	Unknown	Unknown
RCAP_rcc01290	-0.404	0.121	0.562	0.000	0.350	0.251	*UvrD/REP helicase*	Replication, Recombination and Repair	Unknown
RCAP_rcc01291	0.073	0.730	0.066	0.758	-0.143	0.596	*kinetochore Spc7 domain-containing protein*	Unknown	Unknown
RCAP_rcc01292	-0.103	0.647	0.045	0.870	-0.112	0.662	*OmpA/MotB domain-containing protein*	Unknown	Unknown
RCAP_rcc01293	0.054	0.860	-0.037	0.899	-0.027	0.945	*hypothetical protein*	Unknown	Unknown
RCAP_rcc01294	-0.319	0.052	-0.179	0.246	0.107	0.641	*SNF2 family helicase*	Replication, Recombination and Repair	Unknown
RCAP_rcc01298	-0.244	0.498	1.175	0.000	0.069	0.899	*SNF2 family helicase*	Unknown	Unknown
RCAP_rcc01299	0.070	0.853	-0.481	0.039	-0.218	0.541	*hypothetical protein*	Unknown	Unknown
RCAP_rcc01300	-0.003	0.990	-0.545	0.001	0.039	0.890	*Cas3 family CRISPR-associated helicase*	Replication, Recombination and Repair	Unknown
RCAP_rcc01301	0.224	0.454	0.030	0.909	-0.747	0.006	*Cas5 family CRISPR-associated protein*	Replication, Recombination and Repair	Unknown
RCAP_rcc01302	0.090	0.702	-0.184	0.155	-0.126	0.673	*Csd1 family CRISPR-associated protein*	Replication, Recombination and Repair	Unknown
RCAP_rcc01303	0.477	0.044	-0.274	0.068	-0.044	NA	*Cas2 family CRISPR-associated protein*	Replication, Recombination and Repair	Unknown
RCAP_rcc01304	-0.194	0.486	-0.325	0.093	0.256	0.380	*Cas4 family CRISPR-associated protein*	Replication, Recombination and Repair	Unknown
RCAP_rcc01305	-0.430	0.188	-0.718	0.001	0.377	0.332	*Cas1 family CRISPR-associated protein*	Replication, Recombination and Repair	Unknown
RCAP_rcc01306	0.358	0.240	-0.390	0.163	-0.210	0.629	*Cas2 family CRISPR-associated protein*	Replication, Recombination and Repair	Unknown
RCAP_rcc01307	0.197	0.323	-0.976	0.000	0.284	0.132	*hypothetical protein*	Unknown	Unknown
RCAP_rcc01309	0.244	0.470	-0.217	0.612	-0.226	0.453	*IS66 family transposase*	Replication, Recombination and Repair	Recombination
RCAP_rcc01310	0.341	0.214	0.304	0.401	-0.796	0.002	*hypothetical protein*	Unknown	Unknown
RCAP_rcc01311	0.042	0.911	-0.006	0.992	-0.255	0.492	*hypothetical protein*	Unknown	Unknown
RCAP_rcc01312	0.453	0.035	-0.760	0.000	-0.200	0.579	*hypothetical protein*	Unknown	Unknown
RCAP_rcc01313	0.518	0.053	-0.362	0.112	-0.423	0.110	*hypothetical protein*	Unknown	Unknown
RCAP_rcc01314	0.244	0.401	-0.340	0.066	-0.215	0.516	*hypothetical protein*	Unknown	Unknown
RCAP_rcc01315	0.244	0.398	-0.435	0.013	-0.184	0.579	*hypothetical protein*	Unknown	Unknown
RCAP_rcc01316	0.172	0.573	-0.532	0.006	-0.185	0.572	*hypothetical protein*	Unknown	Unknown
RCAP_rcc01317	-0.192	0.572	-0.159	0.715	-0.909	0.003	*pirin domain-containing protein*	Unknown	Unknown
RCAP_rcc01318	0.181	0.497	-0.661	0.000	-0.368	0.080	*outer membrane autotransporter barrel domain-containing protein*	Unknown	Unknown
RCAP_rcc01319	0.454	0.216	-1.395	0.000	0.209	0.654	*hypothetical protein*	Unknown	Unknown
RCAP_rcc01320	-0.051	0.850	-0.488	0.026	0.112	0.699	*hypothetical protein*	Unknown	Unknown
RCAP_rcc01321	0.085	0.746	-0.362	0.096	-0.213	0.485	*GNAT family acetyltransferase*	Cell Division	Chromosome Partitioning

RCAP_rcc01323	0.277	0.285	-0.135	0.505	-0.269	0.316	*IS66 family transposase*	Replication, Recombination and Repair	Recombination
RCAP_rcc01324	0.293	0.235	-0.359	0.079	-0.194	0.498	*hsdR2*	Defense Mechanisms	Unknown
RCAP_rcc01325	-0.190	0.392	-0.067	0.727	0.037	0.896	*hsdM2*	Defense Mechanisms	Unknown
RCAP_rcc01326	0.235	0.377	-0.116	0.652	-0.200	0.514	*hsdS2*	Defense Mechanisms	Unknown
RCAP_rcc01327	0.129	0.647	-0.218	0.313	-0.296	0.281	*hypothetical protein*	Unknown	Unknown
RCAP_rcc01328	0.382	0.156	-0.493	0.003	-0.221	0.531	*hypothetical protein*	Unknown	Unknown
RCAP_rcc01329	0.300	0.322	-0.774	0.000	-0.158	0.637	*hypothetical protein*	Unknown	Unknown
RCAP_rcc01330	-0.193	0.631	-0.069	0.903	-0.322	0.480	*hypothetical protein*	Unknown	Unknown
RCAP_rcc01333	0.102	0.832	0.244	0.668	0.075	0.893	*IS66 family transposase*	Replication, Recombination and Repair	Recombination
RCAP_rcc01334	-0.245	0.556	-0.177	0.657	0.196	0.683	*hypothetical protein*	Unknown	Unknown
RCAP_rcc01335	0.307	0.184	-0.126	0.656	0.007	0.988	*Fis family GAF modulated sigma54 specific transcriptional regulator*	Signal Transduction	Transcription Regulator
RCAP_rcc01336	0.376	0.166	0.169	0.665	0.053	0.899	*acxA*	Amino Acid Metabolism	Arginine and proline metabolism
RCAP_rcc01337	0.266	0.203	0.144	0.592	-0.209	0.484	*acxB*	Amino Acid Metabolism	Unknown
RCAP_rcc01338	0.432	0.146	-0.043	0.913	-0.217	0.608	*acxC*	Secondary metabolites biosynthesis, transport, and catabolism	Unknown
RCAP_rcc01339	-0.468	0.247	-0.210	0.715	0.181	0.706	*hypothetical protein*	Unknown	Unknown
RCAP_rcc01340	-0.162	NA	0.067	0.882	0.018	0.978	*LysR family transcriptional regulator*	Signal Transduction	Transcription Regulator
RCAP_rcc01341	-0.368	0.386	0.490	0.068	-0.023	0.972	*hypothetical protein*	Unknown	Unknown
RCAP_rcc01342	0.041	0.912	0.156	0.540	-0.643	0.069	*iron siderophore/cobalamin ABC transporter periplasmic iron siderophor*	Metal, Ion, Cofactor Transport	Iron and Heme Transport
RCAP_rcc01343	-0.164	0.718	0.213	0.557	-0.258	0.556	*iron siderophore/cobalamin ABC transporter permease*	Metal, Ion, Cofactor Transport	Iron and Heme Transport
RCAP_rcc01345	0.460	0.210	0.137	0.778	-0.312	0.486	*hypothetical protein*	Unknown	Unknown
RCAP_rcc01347	0.037	0.897	-0.493	0.014	-0.707	0.001	*pirin domain-containing protein*	Unknown	Unknown
RCAP_rcc01348	-0.154	0.641	-0.212	0.428	-0.030	0.958	*exonuclease*	Replication, Recombination and Repair	Replication
RCAP_rcc01349	-0.368	0.174	-0.023	0.935	-0.501	0.189	*pirin domain-containing protein*	Unknown	Unknown
RCAP_rcc01350	-0.593	0.012	3.152	0.000	0.519	0.009	*hypothetical protein*	Unknown	Unknown
RCAP_rcc01351	-0.160	0.604	1.867	0.000	0.208	0.617	*hypothetical protein*	Unknown	Unknown
RCAP_rcc01352	-0.144	0.525	2.718	0.000	0.372	0.310	*cheY1*	Motility	Chemotaxis
RCAP_rcc01353	-0.436	0.043	2.793	0.000	0.416	0.214	*cheA1*	Motility	Chemotaxis
RCAP_rcc01354	-0.313	0.231	2.942	0.000	0.233	0.539	*cheW1*	Motility	Chemotaxis
RCAP_rcc01355	-0.186	0.486	2.940	0.000	0.324	0.398	*methyl-accepting chemotaxis sensory transducer*	Motility	Chemotaxis
RCAP_rcc01356	-0.483	0.059	2.727	0.000	0.762	0.002	*chemotaxis protein CheW*	Motility	Chemotaxis
RCAP_rcc01357	-0.499	0.053	2.806	0.000	0.635	0.039	*cheR2*	Motility	Chemotaxis
RCAP_rcc01358	-0.495	0.056	2.338	0.000	0.645	0.023	*cheB1*	Signal Transduction	Transcription Regulator
RCAP_rcc01359	-0.625	0.014	-0.433	0.035	0.296	0.356	*hypothetical protein*	Unknown	Unknown
RCAP_rcc01360	-0.604	0.111	-0.520	0.100	0.122	0.799	*adenylate/guanylate cyclase/CHASE2 sensor domain-containing protein*	Nucleotide Metabolism	Purine metabolism
RCAP_rcc01361	-0.071	0.882	-0.223	0.623	0.216	0.592	*Crp/Fnr family transcriptional regulator*	Signal Transduction	Transcription Regulator
RCAP_rcc01362	-0.504	0.083	0.657	0.002	0.198	0.661	*hemolysin-type calcium-binding repeat family protein*	Trafficking and Secretion	Secretion
RCAP_rcc01363	0.276	0.081	0.143	0.337	-0.191	0.446	*leuA*	Carbohydrate Metabolism	Pyruvate metabolism
RCAP_rcc01364	-0.129	0.503	0.340	0.002	-0.122	0.514	*signal transduction histidine kinase*	Signal Transduction	Kinase/Phosphorelay
RCAP_rcc01365	-0.308	0.195	-0.023	0.913	0.285	0.240	*MORN repeat family protein*	Unknown	Unknown
RCAP_rcc01366	-0.174	0.324	0.064	0.644	0.002	0.991	*nadE*	Metabolism of Cofactors, Coenzymes and Vitamins	Nicotinate and nicotinamide metabolism
RCAP_rcc01367	0.286	0.327	0.382	0.049	-0.120	0.689	*hypothetical protein*	Unknown	Unknown
RCAP_rcc01368	0.984	0.000	-0.472	0.077	-0.246	0.559	*antibiotic biosynthesis monooxygenase*	Unknown	Unknown
RCAP_rcc01369	0.055	0.822	0.030	0.921	0.295	0.002	*ABC transporter periplasmic substrate-binding protein*	Metal and Ion Transport	Unknown
RCAP_rcc01370	0.159	0.520	-0.208	0.369	0.009	0.981	*ABC transporter permease*	Unknown	Unknown
RCAP_rcc01371	0.217	0.369	-0.123	0.572	-0.040	0.900	*ABC transporter permease*	Amino Acid Metabolism	Amino Acid Transport
RCAP_rcc01372	0.155	0.541	-0.455	0.002	-0.049	0.864	*ABC transporter ATP-binding protein*	Amino Acid Metabolism	Amino Acid Transport
RCAP_rcc01373	-0.522	0.069	0.135	0.761	0.088	0.814	*RpiR family transcriptional regulator*	Signal Transduction	Transcription Regulator
RCAP_rcc01374	0.219	0.503	0.778	0.023	-0.078	0.794	*dctQ1*	Unknown	Unknown
RCAP_rcc01375	0.248	0.299	0.322	0.277	-0.037	0.881	*dctM1*	Secondary metabolites biosynthesis, transport, and catabolism	Unknown
RCAP_rcc01376	0.683	0.007	0.260	0.398	-0.155	0.651	*dctP1*	Unknown	Unknown
RCAP_rcc01377	0.448	0.068	-0.183	0.396	-0.139	0.661	*glnA2*	Carbohydrate Metabolism	Glyoxylate and dicarboxylate metabolism
RCAP_rcc01378	0.381	0.050	-0.837	0.000	0.034	0.899	*aldehyde dehydrogenase*	Energy Metabolism	Limonene and pinene degradation
RCAP_rcc01379	0.243	0.199	-0.822	0.000	0.022	0.943	*iron-containing alcohol dehydrogenase*	Xenobiotics Biodegradation and Metabolism	Drug metabolism - cytochrome P450
RCAP_rcc01380	0.012	0.958	-0.060	0.711	-0.065	0.760	*hypothetical protein*	Unknown	Unknown
RCAP_rcc01381	-0.800	0.001	-0.124	0.401	0.532	0.087	*lipoprotein*	Predicted Function	Unknown
RCAP_rcc01382	-0.937	0.010	-0.291	0.060	0.827	0.010	*hypothetical protein*	Unknown	Unknown
RCAP_rcc01383	0.258	0.303	-0.175	0.256	-0.184	0.540	*ugd*	Carbohydrate Metabolism	Pentose and glucuronate interconversions
RCAP_rcc01384	0.264	0.310	-0.360	0.002	-0.335	0.135	*uvrB*	Replication, Recombination and Repair	Repair
RCAP_rcc01385	-0.057	0.827	0.341	NA	-0.083	0.730	*NDUFA4 family NADH ubiquinone oxidoreductase subunit*	Energy Metabolism	Unknown
RCAP_rcc01386	-0.410	0.299	0.957	0.049	-0.276	0.545	*hypothetical protein*	Unknown	Unknown
RCAP_rcc01387	-0.084	0.800	-0.024	0.930	0.011	0.984	*RpiR family transcriptional regulator*	Signal Transduction	Transcription Regulator
RCAP_rcc01388	0.644	0.014	0.538	0.037	-0.227	0.524	*potD2*	Amino Acid Metabolism	Amino Acid Transport
RCAP_rcc01389	0.222	0.492	0.281	0.421	0.093	0.838	*potA2*	Amino Acid Metabolism	Amino Acid Transport
RCAP_rcc01390	0.212	0.480	0.271	0.374	-0.303	0.347	*potB2*	Metal and Ion Transport	Unknown
RCAP_rcc01391	0.257	0.391	0.119	0.752	-0.380	0.234	*potC1*	Amino Acid Metabolism	Amino Acid Transport
RCAP_rcc01392	0.012	0.973	0.240	0.464	-0.260	0.565	*S58 family peptidase*	Post-translational Modification, Assembly and Chaperones	Peptidase
RCAP_rcc01393	-0.814	0.034	1.000	0.017	0.037	0.954	*LuxR family two component transcriptional regulator*	Signal Transduction	Transcription Regulator
RCAP_rcc01394	0.304	0.497	0.349	0.527	-0.197	0.680	*hypothetical protein*	Unknown	Unknown
RCAP_rcc01395	0.170	0.690	-0.251	0.534	-0.098	0.844	*pentapeptide repeat family protein*	Unknown	Unknown
RCAP_rcc01396	0.173	0.526	0.121	0.621	-0.195	0.549	*exaA1*	Carbohydrate Metabolism	Glycolysis / Gluconeogenesis
RCAP_rcc01397	-0.094	NA	0.002	0.997	0.052	NA	*metallo-beta-lactamase*	Unknown	Unknown
RCAP_rcc01398	-0.180	NA	0.518	0.468	-0.145	NA	*hypothetical protein*	Unknown	Unknown
RCAP_rcc01399	-0.326	0.458	-0.088	0.865	-0.104	0.838	*rhodanese domain-containing protein*	Metal and Ion Transport	Unknown
RCAP_rcc01400	-0.424	0.230	0.274	0.371	0.108	0.833	*signal transduction histidine kinase*	Signal Transduction	Kinase/Phosphorelay
RCAP_rcc01401	-0.097	0.744	0.313	0.064	0.190	0.549	*AraC family transcriptional regulator*	Signal Transduction	Transcription Regulator
RCAP_rcc01402	0.261	0.112	-0.479	0.002	-0.147	0.592	*ABC transporter substrate-binding protein*	Unknown	Unknown
RCAP_rcc01403	0.126	0.539	-0.490	0.000	-0.098	0.617	*ABC transporter permease*	Metal and Ion Transport	Unknown

RCAP_rcc01404	-0.184	0.411	-0.336	0.004	0.102	0.769	*ABC transporter ATP-binding protein*	Metal and Ion Transport	Unknown
RCAP_rcc01405	-0.255	0.371	0.972	0.000	0.318	0.338	*hypothetical protein*	Unknown	Unknown
RCAP_rcc01406	-1.130	0.001	1.069	0.011	0.140	0.775	*phage lysozyme*	Replication, Recombination and Repair	Phage Interaction
RCAP_rcc01407	-0.643	0.108	0.909	0.103	0.381	NA	*hypothetical protein*	Unknown	Unknown
RCAP_rcc01408	-0.272	NA	1.035	0.022	0.324	NA	*hypothetical protein*	Unknown	Unknown
RCAP_rcc01409	-0.340	0.210	1.528	0.000	0.212	0.651	*hypothetical protein*	Unknown	Unknown
RCAP_rcc01410	-0.302	0.453	0.996	0.001	0.259	0.555	*hypothetical protein*	Unknown	Unknown
RCAP_rcc01412	0.524	0.207	0.980	0.028	-0.166	NA	*hypothetical protein*	Unknown	Unknown
RCAP_rcc01413	0.016	0.971	1.155	0.001	-0.419	0.267	*hypothetical protein*	Unknown	Unknown
RCAP_rcc01414	-0.102	0.835	0.296	0.587	-0.097	NA	*hypothetical protein*	Unknown	Unknown
RCAP_rcc01415	0.779	0.001	-0.607	0.000	-0.220	0.523	*serine/threonine-protein kinase*	Signal Transduction	Kinase/Phosphorelay
RCAP_rcc01416	-0.193	0.368	-0.596	0.000	0.140	0.607	*hypothetical protein*	Unknown	Unknown
RCAP_rcc01417	-0.180	NA	-0.177	0.801	0.110	NA	*hypothetical protein*	Unknown	Unknown
RCAP_rcc01418	0.047	0.915	0.357	0.357	-0.282	0.318	*hypothetical protein*	Unknown	Unknown
RCAP_rcc01419	0.172	0.635	0.284	0.430	-0.768	0.023	*hypothetical protein*	Unknown	Unknown
RCAP_rcc01420	0.391	0.371	0.464	0.392	-0.184	0.692	*S49 family peptidase*	Post-translational Modification, Assembly and Chaperones	Peptidase
RCAP_rcc01421	0.264	0.520	0.192	0.729	-0.264	0.537	*fldA*	Energy Metabolism	Unknown
RCAP_rcc01422	-0.053	0.906	-0.870	0.022	-0.221	0.621	*hypothetical protein*	Unknown	Unknown
RCAP_rcc01423	0.693	0.003	-1.331	0.000	-0.158	0.661	*hypothetical protein*	Unknown	Unknown
RCAP_rcc01424	-0.007	0.990	1.219	0.031	-0.252	0.572	*hypothetical protein*	Unknown	Unknown
RCAP_rcc01425	-0.294	0.479	1.149	0.000	0.125	0.787	*hypothetical protein*	Unknown	Unknown
RCAP_rcc01426	-0.184	0.674	0.614	0.103	-0.286	0.519	*ABC transporter ATP-binding/permease*	Unknown	Unknown
RCAP_rcc01427	0.220	NA	0.025	0.966	0.207	NA	*ABC transporter ATP-binding/permease*	Unknown	Unknown
RCAP_rcc01428	-0.482	NA	0.018	0.980	0.225	NA	*major facilitator superfamily protein*	Unknown	Unknown
RCAP_rcc01429	0.205	0.503	0.397	0.108	-0.143	0.713	*TonB-dependent receptor*	Metal and Ion Transport	Unknown
RCAP_rcc01430	-0.171	0.716	-0.270	0.406	0.303	0.492	*O-methyltransferase domain-containing protein*	Secondary metabolites biosynthesis, transport, and catabolism	Unknown
RCAP_rcc01431	-0.718	0.018	0.452	0.150	-0.266	0.541	*AraC family transcriptional regulator*	Signal Transduction	Transcription Regulator
RCAP_rcc01432	-0.495	0.034	0.700	0.078	-0.290	0.453	*AraC family transcriptional regulator*	Signal Transduction	Transcription Regulator
RCAP_rcc01433	0.135	0.640	0.646	0.060	-0.553	0.050	*TonB-dependent siderophore receptor*	Metal, Ion, Cofactor Transport	Iron and Heme Transport
RCAP_rcc01434	-0.590	0.119	0.827	0.000	0.245	0.578	*fepB1*	Metal, Ion, Cofactor Transport	Iron and Heme Transport
RCAP_rcc01435	-0.005	NA	0.428	0.401	0.042	NA	*fepD1*	Metal, Ion, Cofactor Transport	Iron and Heme Transport
RCAP_rcc01436	-0.172	0.718	-0.122	0.803	0.018	0.978	*fepG1*	Metal, Ion, Cofactor Transport	Iron and Heme Transport
RCAP_rcc01437	0.329	0.454	0.092	0.864	-0.029	NA	*fepC1*	Metal, Ion, Cofactor Transport	Iron and Heme Transport
RCAP_rcc01438	-0.269	0.550	0.390	0.418	0.342	NA	*siderophore-interacting protein*	Metal, Ion, Cofactor Transport	Iron and Heme Transport
RCAP_rcc01439	-0.331	0.339	-0.020	0.963	0.346	0.442	*ABC transporter ATP-binding/permease*	Metal, Ion, Cofactor Transport	Iron and Heme Transport
RCAP_rcc01440	-0.169	0.662	0.147	0.687	0.041	0.950	*ABC transporter ATP-binding/permease*	Metal, Ion, Cofactor Transport	Iron and Heme Transport
RCAP_rcc01441	-0.438	0.301	-0.156	0.739	-0.038	0.953	*fepC2*	Metal, Ion, Cofactor Transport	Iron and Heme Transport
RCAP_rcc01442	-0.004	NA	-0.896	0.094	-0.216	NA	*fepG2*	Metal, Ion, Cofactor Transport	Iron and Heme Transport
RCAP_rcc01443	0.248	NA	-1.084	0.088	-0.085	NA	*fepD2*	Metal, Ion, Cofactor Transport	Iron and Heme Transport
RCAP_rcc01444	0.256	0.527	-0.497	0.177	-0.245	0.579	*fepB2*	Metal, Ion, Cofactor Transport	Iron and Heme Transport
RCAP_rcc01445	0.510	0.136	0.524	0.076	-1.210	0.000	*tonB-dependent receptor*	Metal and Ion Transport	Unknown
RCAP_rcc01446	-0.231	0.167	-0.231	0.168	0.353	0.006	*M20 family peptidase*	Post-translational Modification, Assembly and Chaperones	Peptidase
RCAP_rcc01447	0.404	0.052	1.315	0.000	-0.295	0.267	*hemA*	Metabolism of Cofactors, Coenzymes and Vitamins	Heme Biosynthesis
RCAP_rcc01448	0.182	0.325	-0.113	0.627	-0.253	0.187	*hypothetical protein*	Unknown	Unknown
RCAP_rcc01449	0.114	0.601	-0.111	0.574	-0.055	0.827	*ispG*	Lipid	Terpenoid backbone biosynthesis
RCAP_rcc01450	-0.160	0.610	0.145	0.397	0.157	0.442	*serS*	Translation, ribosomal structure and biogenesis	Aminoacyl-tRNA biosynthesis
RCAP_rcc01451	-0.058	0.890	0.070	0.836	0.226	0.610	*RND family efflux transporter subunit MFP*	Cell Envelope Biosynthesis	Cell Wall Biosynthesis
RCAP_rcc01452	0.037	0.871	-0.158	0.351	-0.323	0.136	*acriflavin resistance protein family*	Metal and Ion Transport	Unknown
RCAP_rcc01453	-0.650	0.014	0.064	0.818	0.523	NA	*ArsR family transcriptional regulator*	Signal Transduction	Transcription Regulator
RCAP_rcc01454	0.019	0.942	-0.181	0.343	-0.184	0.464	*corA*	Metal, Ion, Cofactor Transport	Cobalt Transport
RCAP_rcc01455	-0.637	0.006	-0.274	0.124	-0.034	0.958	*hypothetical protein*	Unknown	Unknown
RCAP_rcc01456	-0.982	0.004	-0.054	0.765	0.737	0.036	*MarC family membrane protein*	Trafficking and Secretion	Trafficing
RCAP_rcc01457	-0.104	0.817	-0.442	0.151	0.301	0.504	*DeoR family transcriptional regulator*	Signal Transduction	Transcription Regulator
RCAP_rcc01458	0.680	0.004	-0.524	0.000	-0.304	0.382	*tatA*	Trafficking and Secretion	Secretion
RCAP_rcc01459	-0.106	0.612	-0.544	0.000	0.244	0.317	*tatB*	Trafficking and Secretion	Secretion
RCAP_rcc01460	0.330	0.093	-0.434	0.000	-0.144	0.632	*tatC*	Trafficking and Secretion	Secretion
RCAP_rcc01461	0.243	0.334	-0.732	0.000	0.026	0.948	*ATPase AAA*	Unknown	Unknown
RCAP_rcc01462	0.502	0.004	-0.304	0.050	-0.149	0.483	*nrdJ2*	Metabolism of Other Amino Acids	Glutathione metabolism
RCAP_rcc01463	0.587	0.010	-0.635	0.000	-0.146	0.626	*hypothetical protein*	Unknown	Unknown
RCAP_rcc01464	-0.006	0.981	-0.647	0.001	0.382	0.211	*hypothetical protein*	Unknown	Unknown
RCAP_rcc01465	0.202	0.377	0.420	0.001	-0.078	0.777	*hypothetical protein*	Unknown	Unknown
RCAP_rcc01466	0.252	0.195	-0.319	0.032	0.009	0.981	*glutamate synthase domain-containing protein*	Energy Metabolism	Nitrogen metabolism
RCAP_rcc01467	-0.360	0.352	-0.209	0.451	0.394	0.362	*gcvA*	Transcription	Unknown
RCAP_rcc01468	-0.087	NA	-0.546	0.225	0.036	0.953	*FAD dependent oxidoreductase*	Energy Metabolism	Unknown
RCAP_rcc01469	-0.334	0.376	-0.120	0.720	0.103	0.838	*hypothetical protein*	Unknown	Unknown
RCAP_rcc01470	-0.046	0.878	-0.134	0.553	0.265	0.403	*L-aminoadipate-semialdehyde dehydrogenase*	Carbohydrate Metabolism	Glycolysis / Gluconeogenesis
RCAP_rcc01471	-0.251	0.345	-0.150	0.505	-0.091	0.777	*aspC1*	Energy Metabolism	Tropane, piperidine and pyridine alkaloid biosynthesis
RCAP_rcc01472	0.007	0.978	-0.076	0.674	-0.202	0.383	*aroQ*	Amino Acid Metabolism	Phenylalanine, tyrosine and tryptophan biosynthesis
RCAP_rcc01473	0.462	0.073	0.144	0.560	-0.266	0.379	*AraC family transcriptional regulator*	Signal Transduction	Transcription Regulator
RCAP_rcc01474	-0.424	0.210	0.570	0.058	-0.204	0.632	*amino acid permease*	Amino Acid Metabolism	Unknown
RCAP_rcc01475	0.206	0.584	0.291	0.473	-0.073	0.893	*ocd*	Amino Acid Metabolism	Arginine and proline metabolism
RCAP_rcc01476	-0.420	NA	0.759	0.265	-0.366	NA	*endoribonuclease L-PSP family protein*	Unknown	Unknown
RCAP_rcc01477	-0.377	NA	0.085	0.893	0.174	0.683	*FAD dependent oxidoreductase*	Amino Acid Metabolism	Phenylalanine metabolism
RCAP_rcc01478	-0.509	0.069	-0.836	0.000	0.324	0.284	*CIA30 family NADH ubiquinone oxidoreductase subunit*	Energy Metabolism	Unknown
RCAP_rcc01479	-0.315	0.405	-0.123	0.763	0.438	0.300	*hypothetical protein*	Unknown	Unknown
RCAP_rcc01480	-0.877	0.001	0.126	0.711	0.119	0.782	*transmembrane pair family protein*	Unknown	Unknown
RCAP_rcc01481	0.016	0.956	-0.180	0.332	0.086	0.652	*murE*	Glycan Biosynthesis and Metabolism	Peptidoglycan biosynthesis

RCAP_rcc01482	-0.067	0.752	0.240	0.138	0.065	0.827	*murF*	Glycan Biosynthesis and Metabolism	Peptidoglycan biosynthesis
RCAP_rcc01483	0.174	0.310	0.682	0.000	-0.074	0.740	*mraY*	Glycan Biosynthesis and Metabolism	Peptidoglycan biosynthesis
RCAP_rcc01484	0.212	0.310	0.410	0.050	-0.089	0.748	*hypothetical protein*	Unknown	Unknown
RCAP_rcc01485	0.055	0.784	-0.324	0.024	-0.031	0.913	*murD*	Glycan Biosynthesis and Metabolism	Peptidoglycan biosynthesis
RCAP_rcc01486	-0.497	0.052	-0.121	0.535	-0.010	0.983	*hypothetical protein*	Unknown	Unknown
RCAP_rcc01487	-0.400	0.162	-0.209	0.413	0.256	0.480	*glutathione-dependent formaldehyde-activating enzyme family protein*	Sulfur Metabolism	Glutathione metabolism
RCAP_rcc01488	0.004	0.993	0.071	0.750	0.288	0.185	*purM*	Nucleotide Metabolism	Purine metabolism
RCAP_rcc01489	-0.352	0.150	-0.114	0.592	0.069	0.808	*purN*	Metabolism of Cofactors, Coenzymes and Vitamins	One carbon pool by folate
RCAP_rcc01490	0.194	0.483	-0.367	0.021	-0.242	0.268	*rnd2*	Translation, ribosomal structure and biogenesis	Unknown
RCAP_rcc01491	0.320	0.059	0.099	0.499	-0.121	0.611	*uvrD*	Replication, Recombination and Repair	Repair
RCAP_rcc01492	0.133	0.704	-0.651	0.002	-0.583	0.060	*hypothetical protein*	Unknown	Unknown
RCAP_rcc01493	0.216	0.437	0.021	0.894	-0.260	0.198	*hypothetical protein*	Unknown	Unknown
RCAP_rcc01494	0.219	0.358	0.131	0.478	-0.018	0.963	*mdeA*	Amino Acid Metabolism	Cysteine and methionine metabolism
RCAP_rcc01495	1.792	0.000	-2.667	0.000	0.225	0.522	*fusA2*	Signal Transduction	Kinase/Phosphorelay
RCAP_rcc01496	-0.446	0.040	-0.180	0.186	0.282	0.161	*lipoprotein*	Predicted Function	Unknown
RCAP_rcc01497	-0.213	0.381	0.227	0.065	0.188	0.484	*sufE*	Unknown	Unknown
RCAP_rcc01498	-0.116	0.569	0.319	0.036	-0.010	0.976	*puuD*	Unknown	Unknown
RCAP_rcc01499	-0.829	0.001	0.045	0.804	0.501	0.201	*DNA alkylation repair enzyme family protein*	Replication, Recombination and Repair	Repair
RCAP_rcc01500	-0.192	0.230	0.174	0.136	0.218	0.300	*glmS*	Carbohydrate Metabolism	Amino sugar and nucleotide sugar metabolism
RCAP_rcc01501	-0.062	0.749	0.325	0.001	0.002	0.991	*glmU*	Carbohydrate Metabolism	Amino sugar and nucleotide sugar metabolism
RCAP_rcc01502	-0.170	0.574	-0.483	0.019	0.341	0.285	*gph2*	Carbohydrate Metabolism	Glyoxylate and dicarboxylate metabolism
RCAP_rcc01503	-0.175	0.381	-0.246	0.169	0.117	0.601	*tetA*	Metal and Ion Transport	Unknown
RCAP_rcc01504	-0.129	0.591	-0.358	0.039	0.406	0.043	*DegT/DnrJ/EryC1/StrS family aminotransferase*	Cell Envelope Biosynthesis	Cell Wall Biosynthesis
RCAP_rcc01505	-0.143	0.594	0.240	0.554	-0.108	0.769	*hypothetical protein*	Unknown	Unknown
RCAP_rcc01506	-0.346	0.044	0.973	0.000	0.237	0.140	*signal transduction histidine kinase*	Signal Transduction	Kinase/Phosphorelay
RCAP_rcc01507	-0.139	0.520	1.981	0.000	0.012	0.975	*hypothetical protein*	Unknown	Unknown
RCAP_rcc01508	-0.228	0.518	-0.320	0.141	0.005	0.991	*LuxR family two component transcriptional regulator*	Signal Transduction	Transcription Regulator
RCAP_rcc01509	-1.074	0.006	-0.063	0.913	-0.256	0.571	*hypothetical protein*	Unknown	Unknown
RCAP_rcc01510	-1.055	0.000	0.568	0.064	0.013	0.968	*ivdH*	Lipid Metabolism	Unknown
RCAP_rcc01511	-1.455	0.000	-0.892	0.001	0.726	0.058	*hypothetical protein*	Unknown	Unknown
RCAP_rcc01512	-1.196	0.000	0.138	0.501	0.178	0.215	*mccB*	Amino Acid Metabolism	Valine, leucine and isoleucine degradation
RCAP_rcc01513	-1.343	0.000	0.084	0.774	0.468	0.073	*mccA*	Amino Acid Metabolism	Valine, leucine and isoleucine degradation
RCAP_rcc01514	-1.005	0.001	-0.491	0.063	0.365	0.182	*glutathione S-transferase*	Sulfur Metabolism	Glutathione metabolism
RCAP_rcc01515	-1.332	0.000	-1.014	0.000	0.636	0.095	*hmgL*	Carbohydrate Metabolism	Butanoate metabolism
RCAP_rcc01516	-1.208	0.000	-0.254	0.519	0.387	0.198	*menB*	Amino Acid Metabolism	Valine, leucine and isoleucine degradation
RCAP_rcc01517	0.500	0.030	-0.588	0.015	-0.055	0.899	*nuoA*	Energy Metabolism	Aerobic/Anaerobic Respiration
RCAP_rcc01518	-0.018	0.934	-0.501	0.041	0.253	0.110	*nuoB*	Energy Metabolism	Aerobic/Anaerobic Respiration
RCAP_rcc01519	0.091	0.683	-0.613	0.004	-0.025	0.953	*nuoC*	Energy Metabolism	Aerobic/Anaerobic Respiration
RCAP_rcc01520	0.032	0.881	-0.563	0.011	0.007	0.984	*nuoD*	Energy Metabolism	Aerobic/Anaerobic Respiration
RCAP_rcc01521	0.011	0.963	-0.686	0.000	-0.045	0.869	*nuoE*	Energy Metabolism	Aerobic/Anaerobic Respiration
RCAP_rcc01522	0.450	0.032	-0.474	0.000	-0.285	0.371	*hypothetical protein*	Unknown	Unknown
RCAP_rcc01523	-0.271	0.121	-0.496	0.005	0.154	0.268	*nuoF*	Energy Metabolism	Aerobic/Anaerobic Respiration
RCAP_rcc01524	-0.271	0.220	-0.691	0.000	0.241	NA	*hypothetical protein*	Unknown	Unknown
RCAP_rcc01525	-0.278	0.414	-0.453	0.148	0.372	0.367	*hypothetical protein*	Unknown	Unknown
RCAP_rcc01526	0.147	0.453	-0.513	0.014	-0.072	0.793	*hypothetical protein*	Unknown	Unknown
RCAP_rcc01527	0.012	0.957	-0.804	0.000	0.175	0.205	*nuoG*	Energy Metabolism	Aerobic/Anaerobic Respiration
RCAP_rcc01528	-0.741	0.000	-1.057	0.000	0.531	0.080	*hypothetical protein*	Unknown	Unknown
RCAP_rcc01529	0.319	0.101	-0.760	0.000	-0.156	0.607	*nuoH*	Energy Metabolism	Aerobic/Anaerobic Respiration
RCAP_rcc01530	0.406	0.075	-0.800	0.000	-0.088	0.782	*hypothetical protein*	Unknown	Unknown
RCAP_rcc01531	0.116	0.589	-0.804	0.000	0.046	0.877	*nuoI*	Energy Metabolism	Aerobic/Anaerobic Respiration
RCAP_rcc01532	0.174	0.489	-0.951	0.000	-0.115	0.633	*pcaC*	Xenobiotics Biodegradation and Metabolism	Benzoate degradation via hydroxylation
RCAP_rcc01533	0.118	0.591	-1.030	0.000	0.001	0.996	*nuoJ*	Energy Metabolism	Aerobic/Anaerobic Respiration
RCAP_rcc01534	0.220	0.350	-0.948	0.000	-0.117	0.736	*nuoK*	Energy Metabolism	Aerobic/Anaerobic Respiration
RCAP_rcc01535	0.279	0.206	-0.966	0.000	-0.088	0.764	*nuoL*	Energy Metabolism	Aerobic/Anaerobic Respiration
RCAP_rcc01536	0.370	0.146	-0.972	0.000	-0.096	0.753	*nuoM*	Energy Metabolism	Aerobic/Anaerobic Respiration
RCAP_rcc01537	-0.104	0.622	-0.939	0.000	0.098	0.576	*nuoN*	Energy Metabolism	Aerobic/Anaerobic Respiration
RCAP_rcc01538	-0.217	0.455	0.019	0.919	0.038	0.894	*birA1*	Metabolism of Cofactors, Coenzymes and Vitamins	Biotin metabolism
RCAP_rcc01539	0.336	0.052	0.441	0.003	-0.137	0.648	*coaX*	Metabolism of Cofactors, Coenzymes and Vitamins	Pantothenate and CoA biosynthesis
RCAP_rcc01540	0.041	0.828	0.142	0.281	-0.054	0.805	*metallo-beta-lactamase*	Unknown	Unknown
RCAP_rcc01541	0.276	0.088	0.344	0.069	-0.316	0.082	*hypothetical protein*	Unknown	Unknown
RCAP_rcc01542	0.320	0.080	0.973	0.000	-0.212	0.178	*ilvH*	Metabolism of Cofactors, Coenzymes and Vitamins	Pantothenate and CoA biosynthesis
RCAP_rcc01543	0.532	0.002	1.117	0.000	-0.259	0.358	*ilvI*	Metabolism of Cofactors, Coenzymes and Vitamins	Pantothenate and CoA biosynthesis
RCAP_rcc01544	0.669	0.001	0.012	0.960	-0.289	0.437	*dctQ2*	Secondary metabolites biosynthesis, transport, and catabolism	Unknown
RCAP_rcc01545	0.350	0.050	-0.181	0.243	-0.191	0.560	*dctM2*	Secondary metabolites biosynthesis, transport, and catabolism	Unknown
RCAP_rcc01546	-0.211	0.393	-0.723	0.055	0.200	0.252	*AMP-dependent synthetase and ligase*	Energy Metabolism	Tropane, piperidine and pyridine alkaloid biosynthesis
RCAP_rcc01547	0.370	0.186	0.376	0.024	-0.241	0.453	*prfB*	Translation, ribosomal structure and biogenesis	Unknown
RCAP_rcc01548	-0.813	0.005	0.451	0.033	0.371	0.229	*shikimate kinase domain-containing protein*	Nucleotide Metabolism	Unknown
RCAP_rcc01549	-0.009	0.981	-0.439	0.098	0.602	0.059	*hypothetical protein*	Unknown	Unknown
RCAP_rcc01550	0.362	0.093	-0.378	0.010	-0.055	0.831	*M23 family peptidase*	Post-translational Modification, Assembly and Chaperones	Peptidase
RCAP_rcc01551	0.189	0.498	-0.266	0.296	-0.312	0.267	*bcp*	Metabolism of Other Amino Acids	Glutathione metabolism
RCAP_rcc01552	-0.325	0.179	-0.306	0.010	0.367	0.200	*hypothetical protein*	Unknown	Unknown
RCAP_rcc01553	0.125	0.600	-0.134	0.366	0.121	0.611	*queA*	Lipid Metabolism	Steroid biosynthesis
RCAP_rcc01554	0.335	0.160	-0.019	0.919	-0.075	0.801	*major facilitator superfamily protein*	Carbohydrate Metabolism	Unknown
RCAP_rcc01555	-0.254	0.197	-0.896	0.000	-0.126	0.671	*hypothetical protein*	Unknown	Unknown
RCAP_rcc01556	-0.060	0.755	-0.065	0.815	0.148	0.479	*lpdA2*	Carbohydrate Metabolism	TCA Cycle
RCAP_rcc01557	-0.405	0.231	-1.074	0.000	-0.183	0.611	*rhodanese domain-containing protein*	Metal and Ion Transport	Unknown
RCAP_rcc01558	0.124	0.498	-0.481	0.000	-0.106	0.526	*uvrA*	Replication, Recombination and Repair	Repair

RCAP_rcc01559	-0.793	0.014	-0.763	0.000	0.411	0.341	*type 11 family methyltransferase*	Unknown	Unknown
RCAP_rcc01560	0.079	0.786	-0.917	0.000	-0.472	0.024	*hypothetical protein*	Unknown	Unknown
RCAP_rcc01561	-0.150	0.403	0.700	0.000	-0.418	0.033	*Crp/Fnr family transcriptional regulator*	Signal Transduction	Transcription Regulator
RCAP_rcc01562	0.067	0.755	0.412	0.162	0.136	0.516	*mmsB*	Amino Acid Metabolism	Valine, leucine and isoleucine degradation
RCAP_rcc01563	-0.333	0.206	0.694	0.054	0.357	0.031	*3-hydroxyisobutyryl-CoA hydrolase*	Carbohydrate Metabolism	Propanoate metabolism
RCAP_rcc01564	0.208	0.476	0.749	0.049	0.260	0.114	*isobutyryl-CoA dehydrogenase*	Amino Acid Metabolism	Valine, leucine and isoleucine degradation
RCAP_rcc01565	0.276	0.279	0.175	0.722	0.146	0.628	*mmsA*	Carbohydrate Metabolism	Propanoate metabolism
RCAP_rcc01566	0.571	0.043	0.279	0.292	-0.272	0.432	*LysR family transcriptional regulator*	Signal Transduction	Transcription Regulator
RCAP_rcc01567	0.308	0.283	0.595	0.002	-0.107	0.691	*coaD*	Metabolism of Cofactors, Coenzymes and Vitamins	Pantothenate and CoA biosynthesis
RCAP_rcc01568	-0.416	0.283	0.117	0.765	0.249	0.525	*RES domain family protein*	Unknown	Unknown
RCAP_rcc01569	0.013	0.969	-0.159	0.414	0.028	0.944	*purF*	Amino Acid Metabolism	Alanine, aspartate and glutamate metabolism
RCAP_rcc01570	0.136	0.561	0.153	0.329	0.022	0.953	*cvpA*	Unknown	Unknown
RCAP_rcc01571	-0.269	0.236	0.358	0.001	0.275	0.279	*radA*	Post-translational Modification, Assembly and Chaperones	Unknown
RCAP_rcc01572	0.137	0.584	0.006	0.971	-0.016	0.961	*ABC transporter ATP-binding protein*	Secondary metabolites biosynthesis, transport, and catabolism	Unknown
RCAP_rcc01573	-0.444	0.025	0.014	0.941	0.257	0.203	*ABC transporter permease*	Secondary metabolites biosynthesis, transport, and catabolism	Unknown
RCAP_rcc01574	0.076	0.783	0.016	0.938	0.020	0.944	*alr*	Metabolism of Other Amino Acids	D-Alanine metabolism
RCAP_rcc01575	0.053	0.819	0.016	0.944	-0.032	0.866	*dnaB*	Metabolism of Cofactors, Coenzymes and Vitamins	Folate biosynthesis
RCAP_rcc01576	0.124	0.611	0.176	0.313	0.059	0.782	*pyrE*	Xenobiotics Biodegradation and Metabolism	Drug metabolism - other enzymes
RCAP_rcc01577	-0.179	0.571	0.275	0.077	0.238	0.266	*pyrC2*	Nucleotide Metabolism	Pyrimidine metabolism
RCAP_rcc01578	0.613	0.016	0.269	0.244	0.211	0.346	*lipoprotein*	Predicted Function	Unknown
RCAP_rcc01579	0.267	0.389	-0.256	0.284	-0.014	0.982	*hypothetical protein*	Unknown	Unknown
RCAP_rcc01580	0.233	0.423	-0.031	0.894	-0.287	0.526	*hypothetical protein*	Unknown	Unknown
RCAP_rcc01581	0.398	0.172	-0.914	0.000	-0.419	0.138	*hypothetical protein*	Unknown	Unknown
RCAP_rcc01582	-0.040	0.906	-0.030	0.909	-0.297	0.488	*hypothetical protein*	Unknown	Unknown
RCAP_rcc01583	0.109	0.709	-0.097	0.487	-0.209	0.361	*hypothetical protein*	Unknown	Unknown
RCAP_rcc01584	0.611	0.014	-0.149	0.297	-0.305	0.356	*hypothetical protein*	Unknown	Unknown
RCAP_rcc01585	-0.599	0.009	-0.451	0.002	0.433	0.176	*hypothetical protein*	Unknown	Unknown
RCAP_rcc01586	-0.281	0.197	-0.586	0.000	0.331	0.186	*GSCFA family protein*	Unknown	Unknown
RCAP_rcc01587	-0.409	0.079	-0.381	0.012	0.305	0.269	*hypothetical protein*	Unknown	Unknown
RCAP_rcc01588	0.008	0.989	-0.591	0.038	0.008	0.989	*GSCFA family protein*	Unknown	Unknown
RCAP_rcc01589	1.625	0.000	-1.025	0.000	-0.257	0.363	*ErfK/YbiS/YcfS/YnhG family protein*	Unknown	Unknown
RCAP_rcc01590	0.136	0.604	0.215	0.121	-0.173	0.478	*infC*	Translation, ribosomal structure and biogenesis	Unknown
RCAP_rcc01591	0.444	0.065	0.320	0.195	-0.241	0.482	*fpr*	Energy Metabolism	Aerobic/Anaerobic Respiration
RCAP_rcc01592	-1.012	0.000	-0.894	0.000	0.495	0.209	*hypothetical protein*	Unknown	Unknown
RCAP_rcc01593	-0.214	0.273	-0.794	0.002	-0.194	0.267	*cysH*	Energy Metabolism	Sulfur metabolism
RCAP_rcc01594	-0.344	0.099	-0.746	0.012	-0.299	0.032	*cysI*	Energy Metabolism	Sulfur metabolism
RCAP_rcc01595	-0.154	0.603	-0.723	0.042	-0.156	0.635	*hypothetical protein*	Unknown	Unknown
RCAP_rcc01596	-0.362	0.242	-0.733	0.086	-0.082	0.882	*cobA1*	Metabolism of Cofactors, Coenzymes and Vitamins	Cobalamin Biosynthesis
RCAP_rcc01597	0.267	0.185	0.201	0.281	-0.100	0.716	*AsnC/Lrp family transcriptional regulator*	Signal Transduction	Transcription Regulator
RCAP_rcc01598	-0.049	0.886	-0.418	0.124	0.070	0.752	*tpiA*	Carbohydrate Metabolism	Glycolysis / Gluconeogenesis
RCAP_rcc01599	0.417	0.046	-0.486	0.008	-0.360	0.168	*iscA*	Energy Metabolism	Aerobic/Anaerobic Respiration
RCAP_rcc01600	0.196	0.296	-0.596	0.002	-0.235	0.267	*nemA*	Energy Metabolism	Unknown
RCAP_rcc01601	0.261	0.381	0.090	0.711	-0.409	0.273	*FeS assembly SUF system protein*	Unknown	Unknown
RCAP_rcc01602	-0.034	0.934	0.216	0.335	0.224	0.387	*tgt*	Translation, ribosomal structure and biogenesis	Unknown
RCAP_rcc01603	-0.756	0.001	-0.001	0.997	0.073	0.684	*hemolysin-III family protein*	Trafficking and Secretion	Secretion
RCAP_rcc01604	-0.565	0.147	0.220	0.233	0.580	0.138	*hypothetical protein*	Unknown	Unknown
RCAP_rcc01605	-0.001	0.998	0.220	0.147	-0.023	0.951	*thiamine monophosphate synthase*	Metabolism of Cofactors, Coenzymes and Vitamins	Thiamine metabolism
RCAP_rcc01606	-0.112	0.688	0.450	0.000	0.190	0.482	*tRNA/rRNA methyltransferase, SpoU family*	Translation, ribosomal structure and biogenesis	Unknown
RCAP_rcc01607	-0.535	0.204	0.092	0.850	0.334	0.427	*hypothetical protein*	Unknown	Unknown
RCAP_rcc01608	-0.612	0.007	-0.099	0.401	0.412	0.249	*cytochrome c oxidase assembly protein*	Energy Metabolism	Aerobic/Anaerobic Respiration
RCAP_rcc01609	0.183	0.470	-0.147	0.187	-0.165	0.487	*cxp*	Amino Acid Metabolism	Unknown
RCAP_rcc01610	-0.512	0.005	-0.047	0.767	-0.185	0.641	*hypothetical protein*	Unknown	Unknown
RCAP_rcc01611	0.050	0.849	-0.022	0.928	-0.742	0.013	*M48 family peptidase*	Post-translational Modification, Assembly and Chaperones	Peptidase
RCAP_rcc01612	-0.001	0.997	-0.418	0.000	0.030	0.908	*hypothetical protein*	Unknown	Unknown
RCAP_rcc01613	-0.184	0.558	-1.533	0.000	0.261	0.316	*hypothetical protein*	Unknown	Unknown
RCAP_rcc01614	-0.008	0.973	-0.495	0.000	0.023	0.939	*metH4*	Amino Acid Metabolism	Unknown
RCAP_rcc01615	0.302	0.189	-0.519	0.001	-0.048	0.881	*methylenetetrahydrofolate reductase*	Energy Metabolism	Reductive carboxylate cycle (CO2 fixation)
RCAP_rcc01616	0.523	0.082	-0.604	0.021	-0.302	0.301	*hypothetical protein*	Unknown	Unknown
RCAP_rcc01617	-0.180	0.470	0.513	0.000	-0.341	0.390	*gppA*	Nucleotide Metabolism	Purine metabolism
RCAP_rcc01618	-0.294	0.125	0.320	0.052	-0.230	0.266	*rrmJ*	Translation, ribosomal structure and biogenesis	Unknown
RCAP_rcc01619	-0.799	0.011	0.248	0.249	0.698	0.046	*Fis family transcriptional regulator*	Signal Transduction	Transcription Regulator
RCAP_rcc01620	0.351	0.289	0.264	0.740	0.124	0.770	*aldB*	Carbohydrate Metabolism	Glycolysis / Gluconeogenesis
RCAP_rcc01621	-0.707	0.003	1.332	0.000	-0.121	0.787	*mcpC*	Motility	Chemotaxis
RCAP_rcc01622	0.670	0.004	0.325	0.001	-0.620	0.007	*hypothetical protein*	Unknown	Unknown
RCAP_rcc01623	-0.263	0.109	-0.028	0.864	0.227	0.244	*AraC family transcriptional regulator*	Signal Transduction	Transcription Regulator
RCAP_rcc01624	-0.526	0.084	-0.261	0.276	0.237	0.556	*miaA*	Translation, ribosomal structure and biogenesis	Unknown
RCAP_rcc01625	0.544	0.012	0.022	0.909	-0.122	0.706	*pyrH*	Xenobiotics Biodegradation and Metabolism	Drug metabolism - other enzymes
RCAP_rcc01626	0.185	0.402	0.231	0.229	-0.018	0.964	*frr*	Translation, ribosomal structure and biogenesis	Unknown
RCAP_rcc01627	-0.455	0.044	0.163	0.206	0.423	0.119	*uppS*	Lipid transport and metabolism	Unknown
RCAP_rcc01628	-0.201	0.337	0.192	0.272	0.152	0.464	*cdsA1*	Signal Transduction	Transcription Regulator
RCAP_rcc01629	0.154	0.424	0.520	0.008	-0.169	0.462	*dxr*	Photosynthesis	Terpenoid backbone biosynthesis
RCAP_rcc01630	0.157	0.367	0.318	0.047	-0.191	0.153	*M50 family peptidase*	Post-translational Modification, Assembly and Chaperones	Peptidase
RCAP_rcc01631	-0.351	0.107	-0.407	0.121	0.056	0.885	*hypothetical protein*	Unknown	Unknown
RCAP_rcc01632	0.119	0.478	-0.015	0.913	-0.053	0.844	*yaeT*	Cell Envelope Biosynthesis	Cell Wall Biosynthesis
RCAP_rcc01633	-0.514	0.109	-0.477	0.003	0.398	0.315	*outer membrane chaperone Skp family protein*	Cell Envelope Biosynthesis	Cell Wall Biosynthesis
RCAP_rcc01634	0.767	0.000	0.317	0.039	-0.250	0.482	*fabZ*	Lipid Metabolism	Unknown
RCAP_rcc01635	-0.126	0.629	0.270	0.040	0.162	0.390	*lpxA*	Glycan Biosynthesis and Metabolism	Lipopolysaccharide biosynthesis

RCAP_rcc01636	-0.869	0.007	0.045	0.855	0.768	0.041	*hypothetical protein*	Unknown	Unknown
RCAP_rcc01637	-0.613	0.011	0.443	0.103	0.483	0.187	*lpxB*	Glycan Biosynthesis and Metabolism	Lipopolysaccharide biosynthesis
RCAP_rcc01638	-0.223	0.377	-0.200	0.252	0.254	0.430	*LamB/YcsF family protein*	Unknown	Unknown
RCAP_rcc01639	0.001	0.997	-0.100	0.719	0.058	0.908	*allophanate hydrolase subunit 2*	Xenobiotics Biodegradation and Metabolism	Atrazine degradation
RCAP_rcc01640	0.125	0.755	-0.113	0.788	-0.104	0.840	*allophanate hydrolase subunit 1*	Xenobiotics Biodegradation and Metabolism	Atrazine degradation
RCAP_rcc01641	-0.454	0.111	-0.503	0.009	0.269	0.556	*transglutaminase-like family protein*	Amino Acid Metabolism	Unknown
RCAP_rcc01642	-0.254	0.267	-0.197	0.270	0.264	0.241	*hypothetical protein*	Unknown	Unknown
RCAP_rcc01643	-0.215	0.387	-0.348	0.158	-0.228	0.284	*transglutaminase-like family protein*	Amino Acid Metabolism	Unknown
RCAP_rcc01644	-0.462	0.231	1.452	0.000	0.250	0.569	*FMN-binding domain-containing protein*	Metabolism of Cofactors, Coenzymes and Vitamins	Unknown
RCAP_rcc01645	0.112	0.793	1.984	0.000	-0.105	0.825	*lipoprotein*	Predicted Function	Unknown
RCAP_rcc01646	0.294	0.484	2.802	0.000	0.177	0.661	*cytochrome c domain-containing protein*	Energy Metabolism	Aerobic/Anaerobic Respiration
RCAP_rcc01647	0.533	0.180	2.785	0.000	-0.081	0.858	*ABC transporter periplasmic substrate-binding protein*	Metal and Ion Transport	Unknown
RCAP_rcc01648	-0.234	0.604	1.349	0.008	-0.148	0.761	*ABC transporter permease*	Metal and Ion Transport	Unknown
RCAP_rcc01649	0.141	NA	-0.071	0.920	-0.104	NA	*ABC transporter ATP-binding protein*	Metal and Ion Transport	Unknown
RCAP_rcc01650	0.218	0.511	1.170	0.002	-0.294	0.432	*ABC transporter permease*	Unknown	Unknown
RCAP_rcc01651	-0.100	NA	1.221	0.019	0.453	NA	*ABC transporter ATP-binding protein*	Unknown	Unknown
RCAP_rcc01652	0.699	0.068	2.594	0.000	0.360	0.358	*hypothetical protein*	Unknown	Unknown
RCAP_rcc01653	0.402	0.184	2.171	0.000	0.017	0.975	*YVTN beta-propeller repeat family protein*	Unknown	Unknown
RCAP_rcc01654	-0.151	0.749	1.902	0.000	0.068	0.899	*hypothetical protein*	Unknown	Unknown
RCAP_rcc01655	-0.173	NA	1.939	0.000	0.280	0.530	*hypothetical protein*	Unknown	Unknown
RCAP_rcc01656	0.481	0.249	1.304	0.000	0.466	0.194	*cycA2*	Energy Metabolism	Aerobic/Anaerobic Respiration
RCAP_rcc01657	0.273	0.267	1.826	0.000	-0.132	0.552	*exaA2*	Carbohydrate Metabolism	Glycolysis / Gluconeogenesis
RCAP_rcc01658	-0.024	0.946	0.436	0.163	0.167	0.633	*two component LuxR family transcriptional regulator*	Signal Transduction	Transcription Regulator
RCAP_rcc01659	-0.036	0.906	-0.665	0.010	0.130	0.691	*hypothetical protein*	Unknown	Unknown
RCAP_rcc01660	-0.052	0.865	-0.606	0.000	0.010	0.984	*signal transduction histidine kinase*	Signal Transduction	Kinase/Phosphorelay
RCAP_rcc01661	0.070	0.825	0.110	0.608	-0.184	0.490	*trmU*	Unknown	Unknown
RCAP_rcc01662	0.253	0.328	1.860	0.000	0.003	0.994	*hypothetical protein*	Unknown	Unknown
RCAP_rcc01663	0.504	0.019	0.915	0.000	-0.091	0.839	*ctrA*	Signal Transduction	Transcription Regulator
RCAP_rcc01664	-0.305	0.083	0.930	0.000	0.082	0.738	*ligA*	Replication, Recombination and Repair	Unknown
RCAP_rcc01665	-0.945	0.000	0.383	0.016	0.455	0.156	*recG*	Metabolism of Cofactors, Coenzymes and Vitamins	Folate biosynthesis
RCAP_rcc01666	0.584	0.013	-0.029	0.886	-0.813	0.000	*hypothetical protein*	Unknown	Unknown
RCAP_rcc01667	-0.874	0.000	1.775	0.000	0.691	0.001	*methyl-accepting chemotaxis sensory transducer*	Motility	Chemotaxis
RCAP_rcc01668	0.489	0.141	-0.301	0.359	-0.105	0.819	*hypothetical protein*	Unknown	Unknown
RCAP_rcc01669	0.366	0.063	0.046	0.746	-0.117	NA	*hisI*	Amino Acid Metabolism	Histidine metabolism
RCAP_rcc01670	-0.239	0.411	0.178	0.470	0.209	0.627	*hypothetical protein*	Unknown	Unknown
RCAP_rcc01671	-0.014	0.963	0.572	0.004	-0.005	0.991	*carbohydrate kinase*	Carbohydrate Metabolism	Unknown
RCAP_rcc01672	0.037	0.919	0.377	0.128	-0.264	0.536	*YjeF-related family protein*	Unknown	Unknown
RCAP_rcc01673	0.379	0.056	-0.390	0.001	-0.446	0.007	*glnB1*	Signal Transduction	Transcription Regulator
RCAP_rcc01674	0.599	0.001	-0.613	0.000	-0.204	0.538	*glnA3*	Carbohydrate Metabolism	Glyoxylate and dicarboxylate metabolism
RCAP_rcc01675	0.027	0.926	0.413	0.032	-0.063	0.734	*fabD*	Lipid Metabolism	Fatty acid biosynthesis
RCAP_rcc01676	-0.208	0.383	0.362	0.036	0.150	0.324	*fabG*	Lipid Metabolism	Biotin metabolism
RCAP_rcc01677	0.463	0.013	0.605	0.000	-0.124	0.738	*hypothetical protein*	Unknown	Unknown
RCAP_rcc01678	0.772	0.000	0.498	0.000	-0.340	0.367	*acpP1*	Lipid Metabolism	Unknown
RCAP_rcc01679	-0.401	0.224	-0.154	0.629	0.154	0.716	*ligT*	Unknown	Unknown
RCAP_rcc01680	0.086	0.712	-0.172	0.401	0.098	0.651	*fabF1*	Lipid Metabolism	Biotin metabolism
RCAP_rcc01681	-0.020	0.938	-0.317	0.013	0.042	0.802	*aminodeoxychorismate lyase*	Unknown	Unknown
RCAP_rcc01682	-0.475	0.245	0.396	0.410	0.031	0.963	*hypothetical protein*	Unknown	Unknown
RCAP_rcc01683	-0.330	0.362	1.083	0.000	-0.096	0.857	*terminase-like family protein*	Unknown	Unknown
RCAP_rcc01684	-0.090	0.850	3.212	0.000	0.014	0.983	*HK97 family phage portal protein*	Replication, Recombination and Repair	Phage Interaction
RCAP_rcc01685	-0.146	NA	2.335	0.000	-0.063	NA	*hypothetical protein*	Unknown	Unknown
RCAP_rcc01686	-0.398	NA	2.632	0.000	-0.032	NA	*phage prohead protease*	Replication, Recombination and Repair	Phage Interaction
RCAP_rcc01687	-0.705	0.043	3.392	0.000	-0.124	0.794	*HK97 family phage major capsid protein*	Replication, Recombination and Repair	Phage Interaction
RCAP_rcc01688	-0.578	NA	3.898	0.000	-0.020	NA	*hypothetical protein*	Unknown	Unknown
RCAP_rcc01689	0.045	NA	1.327	0.043	#VALUE!	NA	*hypothetical protein*	Unknown	Unknown
RCAP_rcc01690	-0.283	NA	2.789	0.000	0.047	NA	*hypothetical protein*	Unknown	Unknown
RCAP_rcc01691	-0.117	NA	3.312	0.000	0.023	NA	*TP901-1 family phage major tail protein*	Replication, Recombination and Repair	Phage Interaction
RCAP_rcc01692	0.094	NA	1.234	0.063	#VALUE!	NA	*hypothetical protein*	Unknown	Unknown
RCAP_rcc01693	#VALUE!	NA	#VALUE!	NA	#VALUE!	NA	*hypothetical protein*	Unknown	Unknown
RCAP_rcc01694	0.053	NA	2.358	0.000	-0.034	NA	*hypothetical protein*	Unknown	Unknown
RCAP_rcc01695	-0.424	0.324	3.431	0.000	-0.603	0.122	*hypothetical protein*	Unknown	Unknown
RCAP_rcc01696	-0.257	NA	2.788	0.000	-0.038	0.948	*hypothetical protein*	Unknown	Unknown
RCAP_rcc01697	-0.001	NA	1.701	0.004	-0.004	NA	*NlpC/P60 family phage cell wall peptidase*	Replication, Recombination and Repair	Phage Interaction
RCAP_rcc01698	-0.333	0.325	2.403	0.000	-0.168	0.710	*hypothetical protein*	Unknown	Unknown
RCAP_rcc01699	0.179	NA	1.214	0.026	0.074	NA	*hypothetical protein*	Unknown	Unknown
RCAP_rcc01700	0.151	0.579	-0.303	0.017	-0.477	0.000	*cysE1*	Energy Metabolism	Sulfur metabolism
RCAP_rcc01701	-0.292	0.360	-0.223	0.283	-0.118	0.785	*phospholipid/glycerol acyltransferase*	Lipid Metabolism	Glycerophospholipid metabolism
RCAP_rcc01702	0.032	0.934	-0.276	0.334	-0.131	0.637	*cdsA2*	Signal Transduction	Transcription Regulator
RCAP_rcc01703	0.141	0.456	0.913	0.000	0.098	0.794	*pdhC*	Carbohydrate Metabolism	TCA Cycle
RCAP_rcc01704	0.275	0.225	0.743	0.004	0.045	0.922	*pdhB*	Carbohydrate Metabolism	TCA Cycle
RCAP_rcc01705	0.518	0.019	0.486	0.128	0.005	0.991	*pdhA*	Carbohydrate Metabolism	TCA Cycle
RCAP_rcc01706	0.187	0.589	0.062	0.883	-0.355	0.141	*septum formation initiator*	Unknown	Unknown
RCAP_rcc01707	0.414	0.084	-0.845	0.000	-0.101	NA	*fda*	Carbohydrate Metabolism	Glycolysis / Gluconeogenesis
RCAP_rcc01708	0.070	0.784	0.214	0.146	0.192	0.236	*pgk*	Carbohydrate Metabolism	Glycolysis / Gluconeogenesis
RCAP_rcc01709	0.288	0.121	0.271	0.083	-0.197	0.520	*ppiA*	Post-translational Modification, Assembly and Chaperones	Unknown
RCAP_rcc01710	0.371	0.023	0.043	0.786	-0.182	0.588	*ppiB*	Post-translational Modification, Assembly and Chaperones	Unknown
RCAP_rcc01711	0.422	0.029	0.064	0.735	-0.174	0.593	*tyrS*	Translation, ribosomal structure and biogenesis	Aminoacyl-tRNA biosynthesis
RCAP_rcc01712	-0.461	0.079	-0.120	0.610	0.290	0.471	*anmK*	Cell Envelope Biosynthesis	Cell Wall Biosynthesis

RCAP_rcc01713	-0.264	0.441	-0.424	0.244	-0.562	0.062	*lipoprotein*	Predicted Function	Unknown
RCAP_rcc01714	-0.658	0.034	-0.230	0.436	0.290	0.526	*hypothetical protein*	Unknown	Unknown
RCAP_rcc01715	0.508	0.019	-0.150	0.440	-0.088	0.783	*eno*	Carbohydrate Metabolism	Glycolysis / Gluconeogenesis
RCAP_rcc01716	-0.290	0.337	-0.661	0.000	0.133	0.582	*hypothetical protein*	Unknown	Unknown
RCAP_rcc01717	-0.270	0.399	-0.727	0.002	-0.146	0.763	*hypothetical protein*	Unknown	Unknown
RCAP_rcc01718	-0.142	0.514	0.346	0.007	-0.137	0.651	*rihA*	Metabolism of Cofactors, Coenzymes and Vitamins	Nicotinate and nicotinamide metabolism
RCAP_rcc01719	-0.185	0.491	0.582	0.000	0.099	0.790	*ABC transporter ATP-binding protein*	Unknown	Unknown
RCAP_rcc01720	0.489	0.018	0.632	0.002	-0.213	0.549	*ndk*	Nucleotide Metabolism	Pyrimidine metabolism
RCAP_rcc01721	-0.245	0.466	-1.301	0.000	0.116	0.820	*TfoX domain-containing protein*	Unknown	Unknown
RCAP_rcc01722	-0.321	0.137	1.781	0.000	-0.163	0.526	*BadM/Rrf2 family transcriptional regulator*	Signal Transduction	Transcription Regulator
RCAP_rcc01723	1.930	0.000	0.213	0.678	0.024	0.972	*ccpA*	Energy Metabolism	Aerobic/Anaerobic Respiration
RCAP_rcc01724	0.409	0.082	-0.397	0.055	0.049	0.899	*speB1*	Amino Acid Metabolism	Arginine and proline metabolism
RCAP_rcc01725	0.498	0.001	0.459	0.028	-0.462	0.013	*hypothetical protein*	Unknown	Unknown
RCAP_rcc01726	0.086	0.745	1.870	0.000	0.125	0.729	*mcpH*	Motility	Chemotaxis
RCAP_rcc01727	0.174	0.382	-1.469	0.000	0.201	0.313	*pyrD1*	Nucleotide Metabolism	Unknown
RCAP_rcc01728	1.179	0.000	-2.925	0.000	0.186	0.516	*nifJ*	Energy Metabolism	Nitrogen metabolism
RCAP_rcc01729	1.432	0.000	-2.279	0.000	0.450	NA	*pyridine nucleotide-disulfide oxidoreductase*	Sulfur Metabolism	Unknown
RCAP_rcc01730	-0.224	0.329	0.270	0.132	0.296	0.076	*U32 family peptidase*	Post-translational Modification, Assembly and Chaperones	Peptidase
RCAP_rcc01731	0.198	0.324	0.375	0.073	-0.135	0.626	*U32 family peptidase*	Post-translational Modification, Assembly and Chaperones	Peptidase
RCAP_rcc01732	-0.354	0.201	0.428	0.031	0.113	0.728	*hypothetical protein*	Unknown	Unknown
RCAP_rcc01733	0.133	0.521	0.561	0.003	-0.125	0.589	*ubiD*	Photosynthesis	Biosynthesis of Ubiquinone
RCAP_rcc01734	-0.825	0.012	0.382	0.020	0.547	0.069	*ubiX*	Photosynthesis	Biosynthesis of Ubiquinone
RCAP_rcc01735	-0.205	0.440	0.686	0.000	0.201	0.358	*NnrS family protein*	Energy Metabolism	Nitrogen Metabolism
RCAP_rcc01736	0.058	0.845	0.119	0.464	-0.015	0.963	*acyltransferase*	Unknown	Unknown
RCAP_rcc01737	-1.132	0.001	-0.125	0.771	0.522	0.195	*oxyR*	Transcription	Unknown
RCAP_rcc01738	1.130	0.000	-1.414	0.000	-0.634	0.110	*katG*	Energy Metabolism	Unknown
RCAP_rcc01739	0.785	0.000	0.608	0.002	-0.326	0.343	*hypothetical protein*	Unknown	Unknown
RCAP_rcc01740	-0.027	0.907	0.367	0.001	0.052	0.750	*ABC transporter ATP-binding protein*	Unknown	Unknown
RCAP_rcc01741	-0.439	0.015	-0.051	0.794	0.240	0.291	*hypothetical protein*	Unknown	Unknown
RCAP_rcc01742	-0.710	0.009	-0.196	0.463	0.248	0.453	*mcr*	Lipid Metabolism	Primary bile acid biosynthesis
RCAP_rcc01743	0.439	0.012	0.244	0.137	0.019	0.933	*DSBA family oxidoreductase*	Post-translational Modification, Assembly and Chaperones	Unknown
RCAP_rcc01744	-0.071	0.866	-0.365	0.153	0.100	0.705	*class I aminotransferase*	Amino Acid Metabolism	Valine, leucine and isoleucine biosynthesis
RCAP_rcc01745	-0.400	0.086	-0.153	0.309	0.362	0.270	*amiC*	Cell Envelope Biosynthesis	Cell Wall Biosynthesis
RCAP_rcc01746	-0.311	0.421	0.042	0.909	0.081	0.869	*hypothetical protein*	Unknown	Unknown
RCAP_rcc01747	0.213	0.451	0.080	0.715	-0.211	0.377	*actP1*	Amino Acid Metabolism	Unknown
RCAP_rcc01748	-0.488	0.029	0.341	0.062	0.677	0.060	*rsmB2*	Translation, ribosomal structure and biogenesis	Unknown
RCAP_rcc01749	-0.043	0.826	0.587	0.000	0.051	0.845	*signal transduction histidine kinase*	Signal Transduction	Kinase/Phosphorelay
RCAP_rcc01750	-0.336	0.282	0.159	0.525	0.260	0.383	*hypothetical protein*	Unknown	Unknown
RCAP_rcc01751	0.275	0.086	0.237	0.032	-0.114	0.626	*recA*	Replication, Recombination and Repair	Recombination
RCAP_rcc01752	0.093	0.749	0.097	0.570	0.045	0.869	*alaS*	Translation, ribosomal structure and biogenesis	Aminoacyl-tRNA biosynthesis
RCAP_rcc01753	0.222	0.350	-0.046	0.729	-0.009	0.982	*hypothetical protein*	Unknown	Unknown
RCAP_rcc01754	0.336	0.229	-0.288	0.062	-0.149	0.583	*class II glutamine amidotransferase*	Unknown	Unknown
RCAP_rcc01755	0.235	0.207	-0.022	0.891	-0.188	0.504	*cysS*	Translation, ribosomal structure and biogenesis	Aminoacyl-tRNA biosynthesis
RCAP_rcc01756	-0.106	0.574	-0.149	0.253	0.073	0.679	*2-isopropylmalate synthase/homocitrate synthase*	Amino Acid Metabolism	Valine, leucine and isoleucine biosynthesis
RCAP_rcc01757	-0.099	0.806	-0.356	0.256	0.157	0.731	*phytoene synthase*	Unknown	Unknown
RCAP_rcc01758	-0.364	0.117	3.166	0.000	0.460	0.028	*mcpA2*	Motility	Chemotaxis
RCAP_rcc01759	-0.364	0.121	3.668	0.000	0.271	0.344	*cheB2*	Signal Transduction	Transcription Regulator
RCAP_rcc01760	-0.072	0.826	3.157	0.000	-0.023	0.953	*cheD*	Motility	Chemotaxis
RCAP_rcc01761	-0.253	NA	3.455	0.000	0.271	0.409	*hypothetical protein*	Unknown	Unknown
RCAP_rcc01762	0.005	0.990	3.659	0.000	0.098	0.799	*cheY2*	Motility	Chemotaxis
RCAP_rcc01763	-0.068	0.832	3.904	0.000	0.208	0.549	*cheR3*	Motility	Chemotaxis
RCAP_rcc01764	-0.315	0.296	3.816	0.000	0.555	0.006	*cheW2*	Motility	Chemotaxis
RCAP_rcc01765	-0.262	NA	3.917	0.000	0.365	0.176	*cheA2*	Motility	Chemotaxis
RCAP_rcc01766	-0.080	0.814	4.278	0.000	0.371	0.168	*cheY3*	Motility	Chemotaxis
RCAP_rcc01767	-0.214	0.509	4.357	0.000	0.372	0.265	*cheX*	Motility	Chemotaxis
RCAP_rcc01768	-0.160	0.457	-0.211	0.318	0.607	0.005	*glk*	Carbohydrate Metabolism	Glycolysis / Gluconeogenesis
RCAP_rcc01769	-0.166	0.438	-0.471	0.003	0.263	0.139	*bglA*	Energy Metabolism	Phenylpropanoid biosynthesis
RCAP_rcc01770	0.044	0.838	-0.211	0.245	0.159	0.591	*LacI family transcriptional regulator*	Signal Transduction	Transcription Regulator
RCAP_rcc01771	0.337	0.215	-1.062	0.000	0.062	0.895	*aglE*	Carbohydrate Metabolism	Unknown
RCAP_rcc01772	0.155	0.550	-1.151	0.000	0.308	0.249	*aglF*	Metal and Ion Transport	Unknown
RCAP_rcc01773	0.238	0.365	-1.177	0.000	0.183	0.601	*aglG*	Metal and Ion Transport	Unknown
RCAP_rcc01774	0.396	0.066	-1.361	0.000	0.104	0.785	*aglA*	Carbohydrate Metabolism	Galactose Metabolism
RCAP_rcc01775	0.257	0.276	-1.541	0.000	0.238	0.425	*aglK*	Amino Acid Metabolism	Amino Acid Transport
RCAP_rcc01776	0.084	0.647	-1.204	0.000	0.064	0.697	*trimethylamine methyltransferase*	Unknown	Unknown
RCAP_rcc01777	0.246	0.247	0.417	0.000	-0.075	0.754	*dusA*	Translation, ribosomal structure and biogenesis	Unknown
RCAP_rcc01778	-0.334	0.254	0.060	0.752	0.352	0.337	*hypothetical protein*	Unknown	Unknown
RCAP_rcc01779	0.001	0.997	-0.158	0.337	-0.157	0.558	*MazG family protein*	Nucleotide Metabolism	Pyrimidine metabolism
RCAP_rcc01780	-0.236	0.121	-0.555	0.000	0.015	0.949	*M20 family peptidase*	Post-translational Modification, Assembly and Chaperones	Peptidase
RCAP_rcc01781	0.871	0.000	0.502	0.012	-0.254	0.516	*yajC*	Trafficking and Secretion	Trafficing
RCAP_rcc01782	0.016	0.942	0.262	0.010	0.089	0.683	*secD*	Trafficking and Secretion	Secretion
RCAP_rcc01783	0.211	0.358	0.031	0.816	-0.036	0.907	*secF*	Trafficking and Secretion	Secretion
RCAP_rcc01784	-0.227	0.345	-0.068	0.746	0.134	0.754	*hypothetical protein*	Unknown	Unknown
RCAP_rcc01785	-0.619	0.043	0.094	0.674	0.485	0.231	*ccmA*	Energy Metabolism	Cytochrome Biogenesis
RCAP_rcc01786	-0.435	0.025	0.212	0.226	0.375	0.122	*ccmB*	Energy Metabolism	Cytochrome Biogenesis
RCAP_rcc01787	0.444	0.082	-0.259	0.035	-0.098	0.661	*ccmC*	Energy Metabolism	Cytochrome Biogenesis
RCAP_rcc01788	-0.492	0.164	-0.440	0.019	0.587	0.076	*ccmD*	Energy Metabolism	Cytochrome Biogenesis
RCAP_rcc01789	0.269	0.351	-0.399	0.001	0.034	0.906	*ccmG*	Energy Metabolism	Cytochrome Biogenesis

RCAP_rcc01790	0.429	0.015	0.327	0.042	-0.204	0.535	*hpt*	Xenobiotics Biodegradation and Metabolism	Drug metabolism - other enzymes
RCAP_rcc01791	0.608	0.037	0.648	0.004	-0.178	0.617	*cyclase/dehydrase*	Lipid Metabolism	Unknown
RCAP_rcc01792	0.164	0.405	0.440	0.013	-0.510	0.003	*amt*	Metal and Ion Transport	Unknown
RCAP_rcc01793	0.080	0.762	0.266	0.189	-0.148	0.589	*cinA domain-containing protein*	Metabolism of Cofactors, Coenzymes and Vitamins	Nicotinate and nicotinamide metabolism
RCAP_rcc01794	-0.721	0.009	0.199	0.210	0.405	0.232	*pgpA*	Lipid Metabolism	Glycerophospholipid metabolism
RCAP_rcc01795	0.166	0.371	0.295	0.060	-0.131	0.559	*ispDF*	Lipid	Terpenoid backbone biosynthesis
RCAP_rcc01796	-0.801	0.008	-0.028	0.896	0.083	0.877	*dusB*	Translation, ribosomal structure and biogenesis	Unknown
RCAP_rcc01797	-0.138	0.616	-0.047	0.834	-0.104	0.702	*ntrB*	Signal Transduction	Kinase/Phosphorelay
RCAP_rcc01798	-0.242	0.227	-0.391	0.002	0.318	0.112	*ntrC*	Signal Transduction	Transcription Regulator
RCAP_rcc01799	-0.384	0.053	-0.165	0.429	0.034	0.929	*ntrY*	Signal Transduction	Kinase/Phosphorelay
RCAP_rcc01800	0.161	0.345	-0.283	0.035	-0.530	0.000	*ntrX*	Signal Transduction	Transcription Regulator
RCAP_rcc01801	0.511	0.028	0.158	0.311	-0.314	0.315	*hfq*	Signal Transduction	Kinase/Phosphorelay
RCAP_rcc01802	-0.378	0.083	0.389	0.063	0.089	0.752	*hflX*	Unknown	Unknown
RCAP_rcc01803	-0.050	0.853	-0.045	0.826	0.092	0.582	*quiP*	Unknown	Unknown
RCAP_rcc01804	-0.217	0.410	-0.527	0.001	0.368	0.131	*garR*	Carbohydrate Metabolism	Glyoxylate and dicarboxylate metabolism
RCAP_rcc01805	0.095	0.735	0.230	0.078	-0.046	0.890	*ssb1*	Replication, Recombination and Repair	Unknown
RCAP_rcc01806	-0.175	0.607	-0.732	0.000	0.452	0.133	*transglycosylase, Slt family*	Cell Envelope Biosynthesis	Cell Wall Biosynthesis
RCAP_rcc01807	-0.133	0.588	-0.616	0.001	0.144	0.608	*hypothetical protein*	Unknown	Unknown
RCAP_rcc01808	0.300	0.186	0.460	0.009	-0.060	0.881	*hemB*	Metabolism of Cofactors, Coenzymes and Vitamins	Heme Biosynthesis
RCAP_rcc01809	-0.128	0.577	-0.128	0.587	0.349	0.237	*hypothetical protein*	Unknown	Unknown
RCAP_rcc01810	0.016	0.949	0.063	0.740	-0.057	0.782	*mfd*	Replication, Recombination and Repair	Repair
RCAP_rcc01811	0.372	0.046	0.165	0.302	-0.039	0.888	*major facilitator superfamily protein*	Metal and Ion Transport	Unknown
RCAP_rcc01812	-0.103	0.678	0.168	0.150	0.156	0.492	*DSBA family oxidoreductase*	Secondary metabolites biosynthesis, transport, and catabolism	Unknown
RCAP_rcc01813	-0.294	0.290	0.263	0.025	0.340	0.169	*AMP-dependent synthetase and ligase*	Energy Metabolism	Reductive carboxylate cycle (CO2 fixation)
RCAP_rcc01814	0.640	0.003	-0.172	0.196	-0.258	0.488	*hypothetical protein*	Unknown	Unknown
RCAP_rcc01815	0.087	0.767	-0.059	0.796	0.050	0.916	*ecnB*	Unknown	Unknown
RCAP_rcc01816	0.558	0.005	-0.303	0.063	-0.189	0.560	*family 5 extracellular solute-binding protein*	Amino Acid Metabolism	Amino Acid Transport
RCAP_rcc01817	0.363	0.031	-0.363	0.030	-0.174	0.316	*bdhA*	Carbohydrate Metabolism	Butanoate metabolism
RCAP_rcc01818	-0.150	0.413	-0.246	0.031	-0.153	0.539	*hypothetical protein*	Unknown	Unknown
RCAP_rcc01819	-0.534	0.080	-0.461	0.019	0.482	NA	*indigoidine synthase A like protein family*	Secondary metabolites biosynthesis, transport, and catabolism	Unknown
RCAP_rcc01820	-0.336	0.212	-0.348	0.019	0.372	0.330	*carbohydrate/purine kinase*	Signal Transduction	Kinase/Phosphorelay
RCAP_rcc01821	0.355	0.163	0.481	0.035	-0.191	0.539	*rpsB*	Translation, ribosomal structure and biogenesis	Unknown
RCAP_rcc01822	0.147	0.608	0.276	0.219	-0.091	NA	*tsf*	Translation, ribosomal structure and biogenesis	Unknown
RCAP_rcc01823	0.150	0.478	-0.092	0.638	0.017	0.947	*LuxR family autoinducer-binding transcriptional regulator*	Signal Transduction	Transcription Regulator
RCAP_rcc01824	0.131	0.458	-0.184	0.333	0.225	0.211	*hypothetical protein*	Unknown	Unknown
RCAP_rcc01825	-0.443	0.016	0.542	0.041	0.172	0.308	*HAD superfamily hydrolase*	Unknown	Unknown
RCAP_rcc01826	0.018	0.952	0.665	0.003	-0.095	0.730	*gph3*	Carbohydrate Metabolism	Glyoxylate and dicarboxylate metabolism
RCAP_rcc01827	-0.264	0.154	0.613	0.002	0.102	0.550	*AhpC/TSA family protein*	Post-translational Modification, Assembly and Chaperones	Unknown
RCAP_rcc01828	0.010	0.969	0.674	0.001	-0.039	0.829	*rpe1*	Carbohydrate Metabolism	Pentose and glucuronate interconversions
RCAP_rcc01829	0.128	0.560	0.921	0.000	-0.075	0.819	*cbbM*	Energy Metabolism	Carbon fixation in photosynthetic organisms
RCAP_rcc01830	0.139	0.558	0.964	0.000	-0.059	0.869	*fba*	Carbohydrate Metabolism	Glycolysis / Gluconeogenesis
RCAP_rcc01831	0.214	0.335	1.095	0.000	0.051	0.865	*gap1*	Carbohydrate Metabolism	Glycolysis / Gluconeogenesis
RCAP_rcc01832	-0.142	0.525	0.961	NA	0.262	0.023	*tkt1*	Carbohydrate Metabolism	Pentose phosphate pathway
RCAP_rcc01833	0.390	0.052	0.749	0.059	-0.099	0.780	*cbbP*	Energy Metabolism	Carbon fixation in photosynthetic organisms
RCAP_rcc01834	0.276	0.446	0.758	0.080	0.045	0.886	*fbp*	Carbohydrate Metabolism	Glycolysis / Gluconeogenesis
RCAP_rcc01835	-0.538	0.078	0.489	0.205	0.212	0.526	*cbbR2*	Signal Transduction	Transcription Regulator
RCAP_rcc01836	0.435	0.058	-0.470	0.011	-0.128	NA	*qor*	Energy Metabolism	Unknown
RCAP_rcc01837	0.477	0.057	-0.608	0.000	-0.119	0.722	*pgm*	Carbohydrate Metabolism	Glycolysis / Gluconeogenesis
RCAP_rcc01838	0.239	0.422	-0.514	0.008	-0.130	0.607	*glgX*	Glycan Biosynthesis and Metabolism	Glycosaminoglycan degradation
RCAP_rcc01839	0.179	0.466	-0.575	0.000	-0.002	0.991	*glgA*	Carbohydrate Metabolism	Starch and sucrose metabolism
RCAP_rcc01840	0.561	0.026	-0.572	0.000	-0.198	0.576	*glgC*	Carbohydrate Metabolism	Amino sugar and nucleotide sugar metabolism
RCAP_rcc01841	0.175	0.529	-0.252	0.045	-0.154	0.599	*glgB*	Carbohydrate Metabolism	Starch and sucrose metabolism
RCAP_rcc01842	0.241	0.336	0.154	0.244	-0.218	0.382	*glgP*	Carbohydrate Metabolism	Starch and sucrose metabolism
RCAP_rcc01843	0.243	0.419	-0.436	0.004	-0.247	0.434	*family 13 glycosyl hydrolase*	Carbohydrate Metabolism	Galactose Metabolism
RCAP_rcc01844	-0.192	0.312	-0.329	0.012	-0.016	0.964	*sensor histidine kinase*	Signal Transduction	Kinase/Phosphorelay
RCAP_rcc01845	0.017	0.951	-0.586	0.001	0.164	0.475	*hypothetical protein*	Unknown	Unknown
RCAP_rcc01846	0.160	0.477	0.086	0.601	-0.073	0.764	*transferase hexapeptide repeat family protein*	Unknown	Unknown
RCAP_rcc01847	-0.460	0.125	0.323	0.011	0.277	0.360	*gmk*	Nucleotide Metabolism	Purine metabolism
RCAP_rcc01848	0.236	0.298	-0.102	0.565	0.071	0.801	*yicC*	Unknown	Unknown
RCAP_rcc01849	-1.058	0.000	-0.192	0.338	0.493	0.059	*hypothetical protein*	Unknown	Unknown
RCAP_rcc01850	0.197	0.494	-0.253	0.141	-0.034	0.922	*aroF*	Amino Acid Metabolism	Phenylalanine, tyrosine and tryptophan biosynthesis
RCAP_rcc01851	0.234	0.402	0.122	0.486	-0.133	0.686	*AraC family transcriptional regulator*	Signal Transduction	Transcription Regulator
RCAP_rcc01852	0.090	0.730	-0.231	0.283	0.123	0.667	*livK1*	Amino Acid Metabolism	Amino Acid Transport
RCAP_rcc01853	0.356	0.134	0.816	0.000	-0.247	0.321	*livG1*	Amino Acid Metabolism	Amino Acid Transport
RCAP_rcc01854	0.224	0.304	0.756	0.000	0.039	0.905	*livF1*	Amino Acid Metabolism	Amino Acid Transport
RCAP_rcc01855	-0.106	0.725	0.770	0.000	0.270	0.345	*hypothetical protein*	Unknown	Unknown
RCAP_rcc01856	-0.015	0.941	0.455	0.001	0.209	0.421	*livH1*	Amino Acid Metabolism	Amino Acid Transport
RCAP_rcc01857	0.204	0.347	0.193	0.141	-0.066	0.816	*livM1*	Amino Acid Metabolism	Amino Acid Transport
RCAP_rcc01858	0.278	0.296	0.452	0.000	-0.156	0.630	*guaA1*	Xenobiotics Biodegradation and Metabolism	Drug metabolism - other enzymes
RCAP_rcc01859	0.065	0.770	-0.018	0.913	0.003	0.989	*aroB*	Amino Acid Metabolism	Phenylalanine, tyrosine and tryptophan biosynthesis
RCAP_rcc01860	-0.627	0.105	0.228	0.177	0.556	0.082	*aroK*	Amino Acid Metabolism	Phenylalanine, tyrosine and tryptophan biosynthesis
RCAP_rcc01861	0.042	0.875	0.139	0.299	-0.408	0.002	*hypothetical protein*	Unknown	Unknown
RCAP_rcc01862	-0.682	0.006	-0.006	0.972	0.409	0.250	*xerD*	Replication, Recombination and Repair	Recombination
RCAP_rcc01863	0.001	0.995	0.106	0.507	0.057	0.753	*hypothetical protein*	Unknown	Unknown
RCAP_rcc01864	0.396	0.031	-0.058	0.688	-0.217	0.380	*lipA*	Metabolism of Cofactors, Coenzymes and Vitamins	Unknown
RCAP_rcc01865	-0.127	0.790	2.697	0.000	-0.332	0.464	*hypothetical protein*	Unknown	Unknown
RCAP_rcc01866	-0.124	0.801	0.378	0.387	0.001	NA	*hypothetical protein*	Unknown	Unknown

RCAP_rcc01867	0.243	0.298	-0.639	0.000	0.052	0.881	*hypothetical protein*	Unknown	Unknown
RCAP_rcc01868	0.220	0.627	0.701	0.264	-0.246	0.589	*hypothetical protein*	Unknown	Unknown
RCAP_rcc01869	0.564	0.023	-0.155	0.464	-0.108	0.696	*invasion associated locus B family protein*	Unknown	Unknown
RCAP_rcc01870	-0.590	0.007	0.278	0.221	0.410	0.195	*fabF2*	Lipid Metabolism	Biotin metabolism
RCAP_rcc01871	0.465	0.057	0.002	0.993	-0.344	0.136	*acpP2*	Unknown	Unknown
RCAP_rcc01872	0.298	0.098	-0.091	0.556	-0.206	0.092	*lpxD*	Cell Envelope Biosynthesis	Cell Wall Biosynthesis
RCAP_rcc01873	0.450	0.012	-0.085	0.642	-0.132	0.625	*peptidoglycan-binding domain 1 protein*	Unknown	Unknown
RCAP_rcc01874	0.858	0.000	-0.728	0.001	-0.174	0.577	*hypothetical protein*	Unknown	Unknown
RCAP_rcc01875	0.044	0.878	-0.447	0.000	-0.089	0.757	*sufS1*	Metabolism of Cofactors, Coenzymes and Vitamins	Thiamine metabolism
RCAP_rcc01876	0.106	0.754	-0.418	0.004	-0.204	0.451	*hypothetical protein*	Unknown	Unknown
RCAP_rcc01877	-0.662	0.003	-0.385	0.001	0.463	0.080	*hypothetical protein*	Unknown	Unknown
RCAP_rcc01878	0.116	0.607	-0.235	0.057	-0.061	0.723	*sufD*	Post-translational Modification, Assembly and Chaperones	Unknown
RCAP_rcc01879	0.411	0.108	-0.054	0.721	-0.219	0.382	*sufC*	Post-translational Modification, Assembly and Chaperones	Unknown
RCAP_rcc01880	0.106	0.749	-0.027	0.906	-0.387	0.036	*hypothetical protein*	Unknown	Unknown
RCAP_rcc01881	0.439	0.124	-0.087	0.540	-0.606	0.000	*sufB*	Post-translational Modification, Assembly and Chaperones	Unknown
RCAP_rcc01882	-0.324	0.199	-0.215	NA	-0.394	0.192	*iscS*	Metabolism of Cofactors, Coenzymes and Vitamins	Thiamine metabolism
RCAP_rcc01883	0.603	0.000	-0.038	0.864	-0.112	0.708	*iscR*	Transcription	Unknown
RCAP_rcc01884	0.589	0.005	0.072	0.708	-0.215	0.552	*alpha/beta fold family hydrolase*	Unknown	Unknown
RCAP_rcc01885	-0.815	0.039	-0.052	0.767	0.592	0.131	*hypothetical protein*	Unknown	Unknown
RCAP_rcc01886	-0.371	0.193	0.309	0.148	0.276	0.209	*metal dependent phosphohydrolase*	Unknown	Unknown
RCAP_rcc01887	0.503	0.042	0.090	0.781	-0.238	0.484	*icd*	Carbohydrate Metabolism	TCA Cycle
RCAP_rcc01888	0.422	0.178	0.659	0.001	-0.256	0.558	*hypothetical protein*	Unknown	Unknown
RCAP_rcc01889	0.427	0.108	0.562	0.000	-0.306	0.357	*hypothetical protein*	Unknown	Unknown
RCAP_rcc01890	-0.412	0.074	-1.180	0.000	0.129	0.641	*hypothetical protein*	Unknown	Unknown
RCAP_rcc01891	0.011	0.969	-0.299	0.480	-0.346	0.151	*emrE*	Metal and Ion Transport	Unknown
RCAP_rcc01892	-0.101	0.751	0.550	0.000	0.015	0.959	*typA*	Signal Transduction	Kinase/Phosphorelay
RCAP_rcc01893	0.139	0.483	-0.181	0.136	0.061	0.797	*potG1*	Amino Acid Metabolism	Amino Acid Transport
RCAP_rcc01894	0.414	0.045	-0.036	0.795	-0.098	0.791	*potI2*	Metal and Ion Transport	Unknown
RCAP_rcc01895	0.591	0.018	0.219	0.144	-0.212	0.574	*potH1*	Metal and Ion Transport	Unknown
RCAP_rcc01896	-0.868	0.003	0.042	0.810	0.351	0.316	*GntR family transcriptional regulator*	Signal Transduction	Transcription Regulator
RCAP_rcc01897	-0.590	0.116	-2.676	0.000	0.538	0.093	*hypothetical protein*	Unknown	Unknown
RCAP_rcc01898	0.049	0.860	-0.640	0.000	-0.304	0.068	*hypothetical protein*	Unknown	Unknown
RCAP_rcc01899	0.037	0.887	-0.334	0.040	-0.010	0.977	*hypothetical protein*	Unknown	Unknown
RCAP_rcc01900	-0.232	0.232	-1.653	0.000	0.276	0.127	*hemolysin-type calcium-binding repeat family protein*	Trafficking and Secretion	Secretion
RCAP_rcc01901	0.224	0.316	0.596	0.000	-0.026	0.944	*polyphosphate kinase 2 domain-containing protein*	Energy Metabolism	Oxidative phosphorylation
RCAP_rcc01902	-0.594	0.042	0.382	0.071	0.057	0.900	*TetR family transcriptional regulator*	Signal Transduction	Transcription Regulator
RCAP_rcc01903	-0.373	0.220	0.332	0.104	0.329	0.381	*lysine exporter protein (LYSE/YGGA)*	Unknown	Unknown
RCAP_rcc01904	-0.256	0.527	0.373	0.412	0.007	0.991	*AsnC/Lrp family transcriptional regulator*	Signal Transduction	Transcription Regulator
RCAP_rcc01905	0.068	0.811	-0.008	0.983	-0.487	0.127	*IS4 family transposase*	Replication, Recombination and Repair	Recombination
RCAP_rcc01906	-0.263	0.286	0.660	0.000	-0.013	0.971	*hypothetical protein*	Unknown	Unknown
RCAP_rcc01907	0.194	0.289	0.207	0.296	-0.180	0.526	*SmpA/OmlA domain-containing protein*	Translation, ribosomal structure and biogenesis	Unknown
RCAP_rcc01908	0.434	0.068	0.039	0.865	0.012	0.981	*hypothetical protein*	Unknown	Unknown
RCAP_rcc01909	-0.119	0.779	0.240	0.182	0.378	0.038	*rpmF*	Translation, ribosomal structure and biogenesis	Unknown
RCAP_rcc01910	0.070	0.842	0.288	0.123	0.116	0.522	*plsX*	Lipid Metabolism	Unknown
RCAP_rcc01911	-0.074	0.706	0.213	0.069	0.157	0.271	*fabH2*	Lipid Metabolism	Biotin metabolism
RCAP_rcc01912	0.208	0.352	-0.490	0.000	-0.174	0.584	*ihfA*	Replication, Recombination and Repair	Unknown
RCAP_rcc01913	0.194	0.423	-0.460	0.000	0.010	0.970	*MerR family transcriptional regulator*	Signal Transduction	Transcription Regulator
RCAP_rcc01914	-0.409	0.056	-0.047	0.812	0.257	0.339	*dcd*	Nucleotide Metabolism	Pyrimidine metabolism
RCAP_rcc01915	0.035	0.910	-0.066	0.751	-0.078	0.771	*guaB*	Xenobiotics Biodegradation and Metabolism	Drug metabolism - other enzymes
RCAP_rcc01916	-0.255	0.422	-0.191	0.510	0.405	0.266	*C4-dicarboxylate transporter/malic acid transport protein family*	Metal and Ion Transport	Unknown
RCAP_rcc01917	-0.043	NA	2.152	0.000	0.072	NA	*hypothetical protein*	Unknown	Unknown
RCAP_rcc01918	-0.101	NA	2.091	0.001	0.023	NA	*hypothetical protein*	Unknown	Unknown
RCAP_rcc01919	0.488	0.030	0.528	0.001	-0.263	0.415	*membrane protein involved in aromatic hydrocarbon degradation*	Cell Envelope Biosynthesis	Cell Wall Biosynthesis
RCAP_rcc01920	-0.450	0.100	-0.947	0.000	0.374	0.167	*hypothetical protein*	Unknown	Unknown
RCAP_rcc01921	0.507	0.016	0.038	0.860	-0.304	0.330	*hypothetical protein*	Unknown	Unknown
RCAP_rcc01922	0.303	0.125	0.328	0.062	-0.419	0.068	*metA*	Energy Metabolism	Sulfur metabolism
RCAP_rcc01923	0.041	0.859	0.922	0.000	-0.474	0.079	*phage integrase*	Replication, Recombination and Repair	Phage Interaction
RCAP_rcc01925	0.437	0.055	0.838	0.000	-0.348	0.123	*hypothetical protein*	Unknown	Unknown
RCAP_rcc01926	0.812	0.002	0.668	0.000	-0.433	0.081	*rnhA1*	Unknown	Unknown
RCAP_rcc01927	0.198	0.520	0.759	0.000	0.173	0.628	*hypothetical protein*	Unknown	Unknown
RCAP_rcc01928	0.082	0.810	-0.172	0.502	-0.300	0.275	*hsdR3*	Defense Mechanisms	Unknown
RCAP_rcc01929	0.219	0.397	0.504	0.063	-0.175	0.562	*hsdS3*	Defense Mechanisms	Unknown
RCAP_rcc01930	0.487	0.009	0.932	0.000	-0.601	0.000	*hsdM3*	Defense Mechanisms	Unknown
RCAP_rcc01931	0.193	0.635	0.101	0.758	-0.249	0.582	*resolvase*	Replication, Recombination and Repair	Unknown
RCAP_rcc01932	0.217	0.520	-0.941	0.000	-0.309	0.233	*family 4 glycosyl transferase*	Metabolism of Cofactors, Coenzymes and Vitamins	Pantothenate and CoA biosynthesis
RCAP_rcc01933	-0.490	0.145	1.276	0.000	0.234	0.608	*hypothetical protein*	Unknown	Unknown
RCAP_rcc01934	0.075	0.869	-0.417	0.142	0.037	0.953	*manB*	Carbohydrate Metabolism	Fructose and mannose metabolism
RCAP_rcc01935	-0.112	0.617	-0.265	0.140	-0.025	0.936	*xanB*	Carbohydrate Metabolism	Fructose and mannose metabolism
RCAP_rcc01936	-0.027	0.926	-0.374	0.014	0.045	0.908	*gne*	Carbohydrate Metabolism	Amino sugar and nucleotide sugar metabolism
RCAP_rcc01937	-0.549	0.098	-0.299	0.196	0.409	0.333	*hypothetical protein*	Unknown	Unknown
RCAP_rcc01938	-0.241	0.286	-0.024	0.906	-0.080	0.786	*hypothetical protein*	Unknown	Unknown
RCAP_rcc01939	0.208	0.490	-0.268	0.140	0.390	0.270	*polysaccharide biosynthesis protein family*	Cell Envelope Biosynthesis	Cell Wall Biosynthesis
RCAP_rcc01940	0.667	0.012	-1.671	0.000	-0.095	0.689	*hemolysin-type calcium-binding repeat family protein*	Trafficking and Secretion	Secretion
RCAP_rcc01941	0.159	0.619	-0.206	0.401	0.012	0.981	*FAD dependent oxidoreductase*	Energy Metabolism	Unknown
RCAP_rcc01942	-0.355	0.329	-0.081	0.823	0.477	0.241	*polysaccharide pyruvyl transferase*	Cell Envelope Biosynthesis	Cell Wall Biosynthesis
RCAP_rcc01943	-0.241	0.531	0.283	0.313	0.190	0.693	*group 1 glycosyl transferase*	Cell Envelope Biosynthesis	Cell Wall Biosynthesis
RCAP_rcc01944	-0.508	0.154	0.183	0.279	0.322	0.430	*family 2 glycosyl transferase*	Cell Envelope Biosynthesis	Cell Wall Biosynthesis

RCAP_rcc01945	-0.126	0.682	0.130	0.417	0.365	0.295	*group 1 glycosyl transferase*	Cell Envelope Biosynthesis	Cell Wall Biosynthesis
RCAP_rcc01946	-0.386	0.059	0.117	0.464	0.130	0.632	*hypothetical protein*	Unknown	Unknown
RCAP_rcc01947	-0.750	0.031	0.060	0.800	0.573	0.157	*twin-arginine translocation pathway signal domain-containing protein*	Unknown	Unknown
RCAP_rcc01948	-0.378	0.058	-0.185	0.173	0.115	0.632	*lipopolysaccharide biosynthesis family protein*	Cell Envelope Biosynthesis	Cell Wall Biosynthesis
RCAP_rcc01949	-0.243	0.457	-1.734	0.000	0.503	NA	*undecaprenyl-phosphate galactosephosphotransferase*	Cell Envelope Biosynthesis	Cell Wall Biosynthesis
RCAP_rcc01950	-0.326	0.204	-0.013	0.938	-0.028	0.947	*polysaccharide biosynthesis/export family protein*	Cell Envelope Biosynthesis	Cell Wall Biosynthesis
RCAP_rcc01951	-0.567	0.139	-0.260	0.305	0.167	0.728	*hypothetical protein*	Unknown	Unknown
RCAP_rcc01952	0.071	0.731	0.405	0.007	-0.147	0.470	*lspL2*	Carbohydrate Metabolism	Amino sugar and nucleotide sugar metabolism
RCAP_rcc01953	0.181	0.496	0.574	0.075	-0.459	0.205	*hypothetical protein*	Unknown	Unknown
RCAP_rcc01954	-0.019	0.953	0.781	0.000	-0.009	0.981	*hypothetical protein*	Unknown	Unknown
RCAP_rcc01955	0.110	0.695	-0.747	0.000	0.349	0.368	*hypothetical protein*	Unknown	Unknown
RCAP_rcc01956	0.078	0.781	-0.354	0.007	0.117	0.710	*lipoprotein*	Predicted Function	Unknown
RCAP_rcc01957	0.061	0.784	-0.123	0.336	-0.036	0.900	*hypothetical protein*	Unknown	Unknown
RCAP_rcc01958	-0.123	0.507	0.426	0.001	0.205	0.192	*wzc*	Signal Transduction	Kinase/Phosphorelay
RCAP_rcc01959	-0.392	0.174	-0.169	0.359	0.132	0.729	*wzb*	Unknown	Unknown
RCAP_rcc01960	0.116	0.525	0.040	0.789	-0.165	0.493	*wza*	Cell Envelope Biosynthesis	Cell Wall Biosynthesis
RCAP_rcc01961	-1.105	0.000	0.364	0.135	0.106	0.833	*gluQ*	Translation, ribosomal structure and biogenesis	Unknown
RCAP_rcc01962	0.023	0.937	0.248	0.054	-0.027	0.930	*gid*	Translation, ribosomal structure and biogenesis	Unknown
RCAP_rcc01963	0.158	0.481	0.698	0.000	-0.016	0.974	*hypothetical protein*	Unknown	Unknown
RCAP_rcc01964	0.332	0.098	-0.167	0.434	-0.116	0.668	*hypothetical protein*	Unknown	Unknown
RCAP_rcc01965	-0.012	0.965	0.068	0.624	0.047	0.751	*gyrA*	Replication, Recombination and Repair	Unknown
RCAP_rcc01966	-0.256	0.456	0.045	0.855	0.325	0.352	*disulfide bond formation protein, DsbB family*	Sulfur Metabolism	Replication
RCAP_rcc01967	0.166	0.444	0.341	0.064	-0.138	0.679	*hypothetical protein*	Unknown	Unknown
RCAP_rcc01968	-0.319	0.388	-0.130	0.636	0.397	0.267	*zinc peptidase*	Metal and Ion Transport	Signaling and Trafficking
RCAP_rcc01969	-0.596	0.062	0.215	0.473	0.088	0.869	*hypothetical protein*	Unknown	Unknown
RCAP_rcc01970	-0.278	0.536	0.801	0.075	0.036	NA	*hypothetical protein*	Unknown	Unknown
RCAP_rcc01971	#VALUE!	NA	0.440	0.453	-0.028	NA	*hypothetical protein*	Unknown	Unknown
RCAP_rcc01972	0.000	NA	0.099	0.886	-0.030	NA	*hypothetical protein*	Unknown	Unknown
RCAP_rcc01973	-0.049	NA	0.572	0.393	0.183	NA	*hypothetical protein*	Unknown	Unknown
RCAP_rcc01974	-0.199	0.670	0.403	0.480	-0.365	NA	*hypothetical protein*	Unknown	Unknown
RCAP_rcc01975	0.205	0.552	-0.110	NA	-0.174	0.549	*hypothetical protein*	Unknown	Unknown
RCAP_rcc01976	-0.085	NA	0.955	0.114	0.003	NA	*hypothetical protein*	Unknown	Unknown
RCAP_rcc01977	-0.001	NA	-0.222	0.771	0.067	NA	*hypothetical protein*	Unknown	Unknown
RCAP_rcc01978	#VALUE!	NA	#VALUE!	NA	0.027	NA	*hypothetical protein*	Unknown	Unknown
RCAP_rcc01979	0.393	0.354	0.404	0.465	-0.230	0.601	*hypothetical protein*	Unknown	Unknown
RCAP_rcc01980	-0.164	NA	0.467	0.479	0.029	NA	*hypothetical protein*	Unknown	Unknown
RCAP_rcc01981	0.255	NA	-0.112	0.883	0.039	NA	*hypothetical protein*	Unknown	Unknown
RCAP_rcc01982	-0.460	NA	0.149	0.822	0.329	NA	*HNH endonuclease*	Unknown	Unknown
RCAP_rcc01983	0.109	NA	0.187	0.764	0.300	0.409	*phage terminase small subunit*	Replication, Recombination and Repair	Phage Interaction
RCAP_rcc01984	-0.387	0.270	0.061	0.886	-0.005	0.991	*phage terminase large subunit*	Replication, Recombination and Repair	Phage Interaction
RCAP_rcc01985	-0.005	0.993	0.243	0.628	0.200	NA	*HK97 family phage portal protein*	Replication, Recombination and Repair	Phage Interaction
RCAP_rcc01986	-0.171	NA	-0.216	0.789	-0.028	NA	*S49 family peptidase*	Post-translational Modification, Assembly and Chaperones	Peptidase
RCAP_rcc01987	0.633	0.107	0.332	0.405	-0.590	0.141	*HK97 family phage major capsid protein*	Replication, Recombination and Repair	Phage Interaction
RCAP_rcc01988	0.191	NA	0.106	0.868	0.011	NA	*hypothetical protein*	Unknown	Unknown
RCAP_rcc01989	-0.058	NA	-0.498	0.478	-0.061	0.899	*hypothetical protein*	Unknown	Unknown
RCAP_rcc01990	0.387	NA	0.000	1.000	-0.152	0.704	*hypothetical protein*	Unknown	Unknown
RCAP_rcc01991	-0.053	NA	0.303	0.679	-0.161	NA	*hypothetical protein*	Unknown	Unknown
RCAP_rcc01992	-0.211	NA	0.416	0.485	0.142	NA	*hypothetical protein*	Unknown	Unknown
RCAP_rcc01993	-0.160	0.730	-0.168	0.741	-0.327	0.453	*hypothetical protein*	Unknown	Unknown
RCAP_rcc01994	-0.238	NA	-0.033	0.959	-0.071	NA	*hypothetical protein*	Unknown	Unknown
RCAP_rcc01995	#VALUE!	NA	-0.058	0.939	#VALUE!	NA	*hypothetical protein*	Unknown	Unknown
RCAP_rcc01996	0.337	0.414	-0.007	0.989	-0.544	0.170	*phage membrane protein*	Replication, Recombination and Repair	Phage Interaction
RCAP_rcc01997	0.196	0.676	-0.427	0.364	-0.142	0.771	*hypothetical protein*	Unknown	Unknown
RCAP_rcc01998	-0.194	NA	0.004	0.995	0.051	NA	*hypothetical protein*	Unknown	Unknown
RCAP_rcc01999	-0.059	NA	-0.401	0.591	-0.027	NA	*hypothetical protein*	Unknown	Unknown
RCAP_rcc02000	-0.110	NA	-0.258	0.731	0.292	NA	*hypothetical protein*	Unknown	Unknown
RCAP_rcc02001	-0.198	NA	0.477	0.452	0.091	NA	*peptidoglycan-binding domain 1 protein*	Unknown	Unknown
RCAP_rcc02002	-0.858	0.014	1.359	0.000	0.124	0.787	*hypothetical protein*	Unknown	Unknown
RCAP_rcc02003	-0.342	0.238	0.699	0.027	0.226	0.529	*dam*	Unknown	Unknown
RCAP_rcc02004	-0.081	0.800	0.824	0.000	0.017	0.958	*hypothetical protein*	Unknown	Unknown
RCAP_rcc02005	0.267	0.303	0.721	0.000	-0.118	0.720	*hypothetical protein*	Unknown	Unknown
RCAP_rcc02006	0.001	0.997	-0.420	0.047	-0.189	0.411	*hypothetical protein*	Unknown	Unknown
RCAP_rcc02007	-0.562	0.046	-0.108	0.602	0.119	0.752	*phage integrase*	Replication, Recombination and Repair	Phage Interaction
RCAP_rcc02008	0.620	0.016	0.438	0.013	-0.247	0.437	*tig*	Post-translational Modification, Assembly and Chaperones	Unknown
RCAP_rcc02009	-0.175	0.524	0.378	0.064	0.217	0.235	*rplI*	Translation, ribosomal structure and biogenesis	Unknown
RCAP_rcc02010	0.090	0.732	0.362	0.094	-0.137	0.621	*rpsR*	Translation, ribosomal structure and biogenesis	Unknown
RCAP_rcc02011	0.275	0.174	0.352	0.113	-0.227	0.453	*rpsF*	Translation, ribosomal structure and biogenesis	Unknown
RCAP_rcc02012	0.531	0.012	0.395	0.093	-0.384	0.167	*hypothetical protein*	Unknown	Unknown
RCAP_rcc02013	-0.003	0.990	-0.381	0.001	0.021	0.949	*hypothetical protein*	Unknown	Unknown
RCAP_rcc02014	-0.117	0.534	-0.190	0.464	-0.202	0.549	*hypothetical protein*	Unknown	Unknown
RCAP_rcc02015	-0.554	0.015	3.431	0.000	0.349	0.117	*aldH1*	Carbohydrate Metabolism	Glycolysis / Gluconeogenesis
RCAP_rcc02016	0.420	0.041	3.798	0.000	-0.192	0.531	*fumarylacetoacetate hydrolase*	Secondary metabolites biosynthesis, transport, and catabolism	Unknown
RCAP_rcc02017	-0.202	0.560	0.475	0.232	0.043	0.900	*rbsA*	Unknown	Unknown
RCAP_rcc02018	-0.079	0.850	0.441	0.293	-0.152	0.538	*rbsC*	Carbohydrate Metabolism	Unknown
RCAP_rcc02019	0.097	0.806	0.632	0.150	-0.253	0.410	*rbsB*	Carbohydrate Metabolism	Unknown
RCAP_rcc02020	0.140	0.647	0.630	0.000	-0.281	0.198	*HAD superfamily hydrolase*	Unknown	Unknown
RCAP_rcc02021	-0.316	0.243	0.363	0.093	0.241	0.474	*DeoR family transcriptional regulator*	Signal Transduction	Transcription Regulator

RCAP_rcc02022	0.021	0.957	0.266	0.129	-0.134	0.715	*polyols ABC transporter periplasmic polyols-binding protein*	Unknown	Unknown
RCAP_rcc02023	-0.106	0.806	0.382	0.031	-0.224	0.526	*polyols ABC transporter permease*	Metal and Ion Transport	Unknown
RCAP_rcc02024	-0.055	0.890	0.302	0.120	0.051	0.905	*polyols ABC transporter permease*	Metal and Ion Transport	Unknown
RCAP_rcc02025	-0.185	0.597	0.203	0.308	-0.272	0.327	*polyols ABC transporter ATP-binding protein*	Amino Acid Metabolism	Amino Acid Transport
RCAP_rcc02026	-0.362	0.227	0.139	0.539	0.075	0.851	*polS*	Carbohydrate Metabolism	Fructose and mannose metabolism
RCAP_rcc02027	-0.716	0.003	0.148	0.507	0.425	0.079	*mtlK*	Carbohydrate Metabolism	Fructose and mannose metabolism
RCAP_rcc02028	-0.780	0.025	0.811	0.000	0.431	0.306	*FeoA family protein*	Metal, Ion, Cofactor Transport	Iron and Heme Transport
RCAP_rcc02029	-0.163	0.357	0.397	0.035	-0.081	0.715	*feoB2*	Metal, Ion, Cofactor Transport	Iron and Heme Transport
RCAP_rcc02030	-0.252	0.221	-0.037	0.872	0.092	0.715	*allA*	Nucleotide Metabolism	Purine metabolism
RCAP_rcc02031	0.056	0.862	-0.479	0.012	-0.056	0.835	*hypothetical protein*	Unknown	Unknown
RCAP_rcc02032	-0.234	0.249	0.001	0.994	0.273	0.253	*cobB*	Metabolism of Cofactors, Coenzymes and Vitamins	Cobalamin Biosynthesis
RCAP_rcc02033	-0.409	0.106	0.036	0.854	0.490	0.179	*cobZ*	Metabolism of Cofactors, Coenzymes and Vitamins	Cobalamin Biosynthesis
RCAP_rcc02034	-0.592	0.015	0.933	0.000	0.527	0.082	*cbiO2*	Metabolism of Cofactors, Coenzymes and Vitamins	Cobalamin Biosynthesis
RCAP_rcc02035	-1.159	0.001	0.240	0.337	0.760	0.038	*cbiQ2*	Metabolism of Cofactors, Coenzymes and Vitamins	Cobalamin Biosynthesis
RCAP_rcc02036	-0.884	0.018	-0.088	0.741	0.582	0.114	*cbiN*	Metabolism of Cofactors, Coenzymes and Vitamins	Cobalamin Biosynthesis
RCAP_rcc02037	-0.296	0.172	0.357	0.035	0.097	0.799	*CbiM family cobalamin biosynthesis protein*	Metabolism of Cofactors, Coenzymes and Vitamins	Cobalamin Biosynthesis
RCAP_rcc02038	-0.479	0.015	0.362	0.048	0.321	0.267	*cobF*	Metabolism of Cofactors, Coenzymes and Vitamins	Cobalamin Biosynthesis
RCAP_rcc02039	-0.232	0.443	0.341	0.061	0.200	0.628	*cobA2*	Metabolism of Cofactors, Coenzymes and Vitamins	Cobalamin Biosynthesis
RCAP_rcc02040	-0.590	0.049	0.349	0.073	0.395	0.229	*cobM*	Metabolism of Cofactors, Coenzymes and Vitamins	Cobalamin Biosynthesis
RCAP_rcc02041	-0.803	0.030	0.321	0.117	0.561	0.162	*cbiG*	Metabolism of Cofactors, Coenzymes and Vitamins	Cobalamin Biosynthesis
RCAP_rcc02042	-0.735	0.006	0.131	0.434	0.416	0.277	*cobL*	Metabolism of Cofactors, Coenzymes and Vitamins	Cobalamin Biosynthesis
RCAP_rcc02043	-0.599	0.019	0.203	0.188	0.247	0.549	*cobK*	Metabolism of Cofactors, Coenzymes and Vitamins	Cobalamin Biosynthesis
RCAP_rcc02044	-0.681	0.017	0.370	0.005	0.693	0.026	*cobJ*	Metabolism of Cofactors, Coenzymes and Vitamins	Cobalamin Biosynthesis
RCAP_rcc02045	-0.338	0.184	0.494	0.000	0.201	0.486	*cobI*	Metabolism of Cofactors, Coenzymes and Vitamins	Cobalamin Biosynthesis
RCAP_rcc02046	-0.510	0.070	0.269	0.115	0.383	0.272	*cobH*	Metabolism of Cofactors, Coenzymes and Vitamins	Cobalamin Biosynthesis
RCAP_rcc02047	-0.722	0.002	0.273	0.006	0.396	0.186	*cobN*	Metabolism of Cofactors, Coenzymes and Vitamins	Cobalamin Biosynthesis
RCAP_rcc02048	0.214	0.437	0.334	0.030	-0.059	0.727	*cobW*	Metabolism of Cofactors, Coenzymes and Vitamins	Cobalamin Biosynthesis
RCAP_rcc02049	0.005	0.991	0.585	0.010	0.174	0.605	*hypothetical protein*	Unknown	Unknown
RCAP_rcc02050	-0.059	0.783	-0.179	0.257	-0.174	0.432	*cobO*	Metabolism of Cofactors, Coenzymes and Vitamins	Cobalamin Biosynthesis
RCAP_rcc02051	-0.469	0.049	-0.046	0.763	0.369	0.192	*cobQ1*	Metabolism of Cofactors, Coenzymes and Vitamins	Cobalamin Biosynthesis
RCAP_rcc02052	-0.107	0.651	-0.195	0.160	0.298	0.216	*bluB*	Metabolism of Cofactors, Coenzymes and Vitamins	Unknown
RCAP_rcc02053	-0.500	0.039	-0.210	0.295	0.218	0.526	*cobC*	Metabolism of Cofactors, Coenzymes and Vitamins	Cobalamin Biosynthesis
RCAP_rcc02054	-0.269	0.272	-0.092	0.623	0.275	0.504	*cobD*	Metabolism of Cofactors, Coenzymes and Vitamins	Cobalamin Biosynthesis
RCAP_rcc02055	-0.424	0.322	0.004	0.993	0.411	0.324	*hypothetical protein*	Unknown	Unknown
RCAP_rcc02056	-0.054	0.910	0.235	0.486	0.244	0.574	*phosphoglycerate mutase*	Metabolism of Cofactors, Coenzymes and Vitamins	Porphyrin and chlorophyll metabolism
RCAP_rcc02057	0.387	0.079	0.498	0.002	-0.373	0.122	*cobP*	Metabolism of Cofactors, Coenzymes and Vitamins	Cobalamin Biosynthesis
RCAP_rcc02058	0.141	0.538	0.272	0.191	0.204	0.387	*cobU*	Metabolism of Cofactors, Coenzymes and Vitamins	Cobalamin Biosynthesis
RCAP_rcc02059	-0.945	0.015	-0.121	0.580	0.761	0.041	*cobV*	Metabolism of Cofactors, Coenzymes and Vitamins	Cobalamin Biosynthesis
RCAP_rcc02060	0.331	NA	-0.901	0.000	-0.014	0.963	*hemolysin-type calcium-binding repeat family protein*	Trafficking and Secretion	Secretion
RCAP_rcc02061	-0.310	0.235	-0.705	0.000	0.225	0.456	*GNAT family acetyltransferase*	Cell Division	Chromosome Partitioning
RCAP_rcc02062	-0.714	0.059	1.207	0.000	0.423	0.315	*Hsp70 family heat shock protein*	Stress Response	Unknown
RCAP_rcc02063	-0.466	0.150	3.561	0.000	0.696	0.000	*M10 family peptidase*	Post-translational Modification, Assembly and Chaperones	Peptidase
RCAP_rcc02064	-0.512	0.066	0.569	0.000	0.414	0.119	*zntA1*	Metal, Ion, Cofactor Transport	Zinc Transport
RCAP_rcc02065	-0.602	0.017	4.194	0.000	0.565	0.000	*secretion ATP-binding protein, HlyB family*	Trafficking and Secretion	Secretion
RCAP_rcc02066	-0.706	0.011	2.993	0.000	0.312	0.413	*hemolysin D*	Trafficking and Secretion	Secretion
RCAP_rcc02067	-0.169	0.534	4.245	0.000	0.120	0.738	*type 11 family methyltransferase*	Unknown	Unknown
RCAP_rcc02068	-0.735	0.039	3.646	0.000	0.879	0.000	*ice nucleation protein repeat family protein*	Unknown	Unknown
RCAP_rcc02069	-0.645	0.020	3.871	0.000	0.712	0.001	*hypothetical protein*	Unknown	Unknown
RCAP_rcc02070	-0.395	0.150	0.546	0.004	0.400	0.211	*ArsR family transcriptional regulator/protein tyrosine phosphatase*	Signal Transduction	Transcription Regulator
RCAP_rcc02071	-0.183	0.426	0.497	0.007	-0.106	0.738	*gap2*	Carbohydrate Metabolism	Glycolysis / Gluconeogenesis
RCAP_rcc02072	-0.454	0.018	0.660	0.000	-0.328	0.246	*nonfunctional major facilitator superfamily protein*	Unknown	Unknown
RCAP_rcc02074	0.019	0.925	-1.299	0.000	-0.006	0.986	*ferredoxin domain-containing protein*	Energy Metabolism	Aerobic/Anaerobic Respiration
RCAP_rcc02075	-0.458	0.011	1.911	0.000	0.366	0.082	*PAS/PAC sensor domain-containing protein*	Motility	Aerotaxis
RCAP_rcc02076	-0.235	0.163	1.000	0.000	0.311	0.142	*diguanylate cyclase/phosphodiesterase*	Signal Transduction	Kinase/Phosphorelay
RCAP_rcc02077	0.375	0.065	1.631	0.000	0.091	0.851	*SET domain-containing protein*	Unknown	Unknown
RCAP_rcc02078	0.487	0.043	1.555	0.000	0.116	0.811	*hypothetical protein*	Unknown	Unknown
RCAP_rcc02079	0.095	0.632	0.106	0.502	-0.166	0.555	*parE*	Replication, Recombination and Repair	Unknown
RCAP_rcc02080	-0.007	0.985	-0.493	0.010	-0.004	0.991	*HAD superfamily hydrolase*	Carbohydrate Metabolism	Unknown
RCAP_rcc02081	-0.393	0.157	0.269	0.167	-0.069	0.828	*MaoC family protein*	Carbohydrate Metabolism	Glyoxylate and dicarboxylate metabolism
RCAP_rcc02082	-0.365	0.094	0.241	0.170	0.333	0.178	*ribF*	Metabolism of Cofactors, Coenzymes and Vitamins	Riboflavin metabolism
RCAP_rcc02083	0.048	0.869	0.164	0.662	-0.342	0.231	*hypothetical protein*	Unknown	Unknown
RCAP_rcc02084	-0.766	0.002	0.134	0.302	0.504	0.079	*ltaE*	Amino Acid Metabolism	Glycine, serine and threonine metabolism
RCAP_rcc02085	-0.284	0.260	0.174	0.478	0.205	0.564	*phage integrase*	Replication, Recombination and Repair	Phage Interaction
RCAP_rcc02086	0.235	NA	0.268	0.688	-0.124	NA	*hypothetical protein*	Unknown	Unknown
RCAP_rcc02088	0.460	0.135	-0.069	0.866	-0.512	0.167	*IS3 family transposase*	Replication, Recombination and Repair	Recombination
RCAP_rcc02095	-0.004	0.993	0.467	0.289	-0.053	0.927	*hypothetical protein*	Unknown	Unknown
RCAP_rcc02097	0.240	0.453	0.291	0.314	-0.243	0.492	*IS111A/IS1328/IS1533 family transposase/IS116/IS110/IS902 family trans*	Replication, Recombination and Repair	Recombination
RCAP_rcc02100	0.442	0.181	0.272	0.546	-0.145	0.756	*IS3/IS911 family transposase*	Replication, Recombination and Repair	Recombination
RCAP_rcc02103	0.328	0.458	-0.081	0.893	-0.008	NA	*IS3/IS911 family transposase*	Replication, Recombination and Repair	Recombination
RCAP_rcc02105	-0.323	0.412	-0.420	0.475	-0.097	0.843	*IS3/IS911 family transposase*	Replication, Recombination and Repair	Recombination
RCAP_rcc02106	0.190	0.559	-0.531	0.014	-0.199	0.531	*NACHT domain-containing protein*	Unknown	Unknown
RCAP_rcc02107	0.004	0.990	-0.452	0.029	-0.066	0.762	*hypothetical protein*	Unknown	Unknown
RCAP_rcc02108	0.373	0.262	-0.313	NA	-0.310	0.273	*hypothetical protein*	Unknown	Unknown
RCAP_rcc02109	-0.051	0.892	-0.012	0.966	0.246	0.591	*hypothetical protein*	Unknown	Unknown
RCAP_rcc02110	-0.097	0.747	0.354	0.018	0.081	0.859	*multicopper oxidase*	Secondary metabolites biosynthesis, transport, and catabolism	Unknown
RCAP_rcc02111	-0.518	0.163	-0.305	0.367	0.866	0.009	*hypothetical protein*	Unknown	Unknown
RCAP_rcc02112	-0.176	0.615	-0.021	0.964	-0.081	0.750	*glyoxalase/bleomycin resistance protein/dioxygenase*	Amino Acid Metabolism	Unknown

RCAP_rcc02113	0.032	0.935	-0.322	0.429	-0.108	0.514	*arcB1*	Amino Acid Metabolism	Arginine and proline metabolism
RCAP_rcc02114	-0.601	0.107	-0.270	0.501	0.465	0.167	*arcA*	Amino Acid Metabolism	Arginine and proline metabolism
RCAP_rcc02115	-0.326	0.266	0.931	0.000	0.025	0.964	*AsnC/Lrp family transcriptional regulator*	Signal Transduction	Transcription Regulator
RCAP_rcc02116	-0.888	0.002	0.704	0.004	0.360	0.297	*iron siderophore/cobalamin ABC transporter ATP-binding protein*	Metal, Ion, Cofactor Transport	Iron and Heme Transport
RCAP_rcc02117	-0.361	0.052	0.931	0.000	0.353	0.266	*methyl-accepting chemotaxis protein*	Motility	Chemotaxis
RCAP_rcc02118	0.338	0.196	-1.669	0.000	-0.033	0.944	*cat*	Unknown	Unknown
RCAP_rcc02119	0.461	0.063	-1.289	0.000	-0.221	0.561	*type 12 family methyltransferase*	Unknown	Unknown
RCAP_rcc02120	0.043	0.908	-0.208	0.492	-0.244	0.574	*ABC transporter ATP-binding/periplasmic substrate-binding protein*	Metal and Ion Transport	Unknown
RCAP_rcc02121	-0.244	0.371	-0.839	0.001	0.394	0.347	*PAS domain/exonuclease domain-containing protein*	Replication, Recombination and Repair	Replication
RCAP_rcc02122	-0.175	0.612	1.026	0.051	0.416	0.295	*response regulator receiver protein*	Signal Transduction	Transcription Regulator
RCAP_rcc02123	0.166	0.565	1.822	0.000	0.411	0.244	*cyclic nucleotide-binding domain-/cystathionine beta-synthase domain-/u*	Signal Transduction	Kinase/Phosphorelay
RCAP_rcc02124	0.770	0.003	1.538	0.009	0.021	0.956	*actP2*	Unknown	Unknown
RCAP_rcc02125	0.953	0.001	1.937	0.001	-0.106	0.727	*hypothetical protein*	Unknown	Unknown
RCAP_rcc02126	0.596	0.010	1.867	0.000	0.039	0.896	*acsA1*	Carbohydrate Metabolism	Glycolysis / Gluconeogenesis
RCAP_rcc02127	-0.360	0.349	0.491	0.072	0.084	0.877	*phaG*	Metal and Ion Transport	Unknown
RCAP_rcc02128	-0.291	0.421	0.410	0.152	-0.412	0.280	*phaF*	Metal and Ion Transport	Unknown
RCAP_rcc02129	0.164	0.703	0.022	0.967	-0.038	0.948	*phaE*	Metal and Ion Transport	Unknown
RCAP_rcc02130	-0.503	0.025	0.252	0.322	0.497	0.021	*phaD*	Metal and Ion Transport	Unknown
RCAP_rcc02131	-0.018	0.962	0.645	0.026	-0.078	0.816	*phaC*	Metal and Ion Transport	Unknown
RCAP_rcc02132	0.543	0.038	0.516	0.002	-0.513	0.019	*phaAB*	Metal and Ion Transport	Unknown
RCAP_rcc02133	-0.541	0.064	0.075	0.707	0.234	0.315	*sbcD*	Replication, Recombination and Repair	Unknown
RCAP_rcc02134	-0.378	0.156	-0.495	0.005	0.298	0.327	*sbcC*	Unknown	Unknown
RCAP_rcc02135	-0.293	0.332	-0.072	0.775	0.212	0.556	*folD1*	Energy Metabolism	Reductive carboxylate cycle (CO2 fixation)
RCAP_rcc02136	-0.769	0.035	0.472	0.008	0.773	0.036	*hypothetical protein*	Unknown	Unknown
RCAP_rcc02137	0.081	0.823	0.048	0.883	0.160	0.672	*hypothetical protein*	Unknown	Unknown
RCAP_rcc02138	-0.345	0.181	0.257	0.248	0.019	0.951	*hypothetical protein*	Unknown	Unknown
RCAP_rcc02139	-0.663	0.012	0.917	0.000	0.286	0.244	*methyl-accepting chemotaxis protein*	Motility	Chemotaxis
RCAP_rcc02140	-0.339	0.230	0.988	0.000	0.296	0.362	*extracellular ligand-binding receptor*	Amino Acid Metabolism	Unknown
RCAP_rcc02141	-0.177	0.710	-2.871	0.000	0.348	0.338	*hypothetical protein*	Unknown	Unknown
RCAP_rcc02142	-0.274	0.207	0.424	0.043	0.078	0.807	*BadM/Rrf2 family transcriptional regulator*	Signal Transduction	Transcription Regulator
RCAP_rcc02143	-0.023	0.961	0.637	0.086	0.323	0.477	*hypothetical protein*	Unknown	Unknown
RCAP_rcc02144	-0.479	0.167	-0.050	0.863	0.395	NA	*ZapA family cell division protein*	Unknown	Unknown
RCAP_rcc02145	-0.568	0.085	0.121	NA	0.558	0.135	*hypothetical protein*	Unknown	Unknown
RCAP_rcc02146	0.065	0.825	-0.068	0.707	0.134	0.531	*tkt2*	Carbohydrate Metabolism	Pentose phosphate pathway
RCAP_rcc02147	-0.718	0.016	0.264	0.019	0.583	0.124	*hypothetical protein*	Unknown	Unknown
RCAP_rcc02148	-0.421	0.125	0.082	0.697	0.371	0.251	*lipid A biosynthesis acyltransferase*	Cell Envelope Biosynthesis	Cell Wall Biosynthesis
RCAP_rcc02149	0.029	0.927	-0.520	0.009	-0.016	0.965	*hypothetical protein*	Unknown	Unknown
RCAP_rcc02150	0.077	0.749	0.569	0.024	-0.043	0.881	*acnA*	Carbohydrate Metabolism	TCA Cycle
RCAP_rcc02151	-0.399	0.276	3.652	0.000	0.303	0.267	*methyl-accepting chemotaxis sensory transducer*	Motility	Chemotaxis
RCAP_rcc02152	0.341	0.207	1.152	0.000	-0.220	0.578	*TM2 domain-containing protein*	Unknown	Unknown
RCAP_rcc02153	-0.587	0.043	0.566	0.002	0.481	0.089	*SCP-like extracellular protein family*	Unknown	Unknown
RCAP_rcc02154	1.262	0.000	0.711	0.000	-0.999	0.000	*TM2 domain-containing protein*	Unknown	Unknown
RCAP_rcc02155	-0.344	0.043	-0.344	0.001	-0.199	0.626	*hypothetical protein*	Unknown	Unknown
RCAP_rcc02156	0.097	0.732	-0.130	0.578	0.050	0.899	*hypothetical protein*	Unknown	Unknown
RCAP_rcc02157	0.429	0.107	0.196	0.357	-0.226	0.487	*purB*	Amino Acid Metabolism	Alanine, aspartate and glutamate metabolism
RCAP_rcc02158	-0.136	0.588	2.586	0.000	0.392	0.024	*fliG*	Motility	Flagellar Assembly
RCAP_rcc02159	-0.282	0.305	0.582	0.077	-0.143	0.738	*hypothetical protein*	Unknown	Unknown
RCAP_rcc02160	0.517	0.008	-0.770	0.000	-0.154	0.641	*gap3*	Carbohydrate Metabolism	Glycolysis / Gluconeogenesis
RCAP_rcc02161	-0.532	0.169	-1.582	0.000	0.417	0.330	*hypothetical protein*	Unknown	Unknown
RCAP_rcc02162	0.039	0.888	-0.358	0.007	0.014	0.961	*mrcA*	Cell Envelope Biosynthesis	Cell Wall Biosynthesis
RCAP_rcc02163	0.201	NA	-0.353	0.088	-0.310	0.016	*ccdA*	Energy Metabolism	Aerobic/Anaerobic Respiration
RCAP_rcc02164	-0.504	NA	0.082	0.835	0.400	NA	*tusA*	Post-translational Modification, Assembly and Chaperones	Unknown
RCAP_rcc02165	0.142	0.520	-0.386	0.000	-0.015	0.958	*rne*	Unknown	Unknown
RCAP_rcc02166	0.148	0.543	-0.371	0.007	0.136	0.410	*dctD*	Signal Transduction	Transcription Regulator
RCAP_rcc02167	-0.152	0.436	-0.213	0.118	0.203	0.331	*dctB*	Signal Transduction	Kinase/Phosphorelay
RCAP_rcc02168	0.466	0.037	0.019	0.935	-0.101	0.730	*purQ*	Nucleotide Metabolism	Purine metabolism
RCAP_rcc02169	0.049	0.867	0.035	0.872	0.097	0.790	*purS*	Nucleotide Metabolism	Purine metabolism
RCAP_rcc02170	0.339	0.127	0.112	0.559	-0.207	0.526	*purC*	Nucleotide Metabolism	Purine metabolism
RCAP_rcc02171	0.304	0.171	0.428	0.006	-0.286	0.266	*hypothetical protein*	Unknown	Unknown
RCAP_rcc02172	-0.501	0.080	2.144	0.000	0.086	0.876	*hypothetical protein*	Unknown	Unknown
RCAP_rcc02173	0.284	0.272	-0.162	0.578	-0.112	0.651	*metH3*	Amino Acid Metabolism	Cysteine and methionine metabolism
RCAP_rcc02174	0.103	0.685	0.244	0.148	-0.054	0.896	*radical SAM family protein*	Replication, Recombination and Repair	Unknown
RCAP_rcc02175	0.491	0.027	-0.790	0.000	-0.108	0.752	*mtbC*	Amino Acid Metabolism	Unknown
RCAP_rcc02176	0.082	0.756	-0.346	0.111	-0.395	0.346	*hypothetical protein*	Unknown	Unknown
RCAP_rcc02177	0.410	NA	-0.136	0.607	-0.458	0.267	*hypothetical protein*	Unknown	Unknown
RCAP_rcc02178	0.474	0.119	-0.492	0.075	-0.494	0.142	*hypothetical protein*	Unknown	Unknown
RCAP_rcc02179	-0.205	0.557	-0.354	0.263	0.033	0.951	*AzlC protein family*	Unknown	Unknown
RCAP_rcc02180	-0.005	NA	-0.471	0.313	0.058	NA	*hypothetical protein*	Unknown	Unknown
RCAP_rcc02181	-0.241	0.423	-0.042	0.830	0.304	0.236	*GntR family transcriptional regulator*	Signal Transduction	Transcription Regulator
RCAP_rcc02182	0.428	0.010	-0.236	0.252	-0.069	0.827	*class III aminotransferase*	Carbohydrate Metabolism	Propanoate metabolism
RCAP_rcc02183	0.633	0.016	-0.660	0.006	-0.233	0.526	*potG2*	Amino Acid Metabolism	Amino Acid Transport
RCAP_rcc02184	0.481	0.155	-0.900	0.002	-0.324	0.277	*potH2*	Metal and Ion Transport	Unknown
RCAP_rcc02185	-0.075	0.827	-0.627	0.018	-0.081	0.815	*potI3*	Unknown	Unknown
RCAP_rcc02186	0.514	0.027	-0.591	0.002	-0.117	0.701	*potF*	Carbohydrate Metabolism	Unknown
RCAP_rcc02187	0.358	0.191	-0.371	0.238	0.197	0.556	*amidohydrolase*	Unknown	Unknown
RCAP_rcc02188	-0.246	0.235	-0.850	0.000	0.195	0.439	*diguanylate cyclase/phosphodiesterase*	Signal Transduction	Kinase/Phosphorelay
RCAP_rcc02189	-0.160	0.506	-1.022	0.000	-0.177	0.462	*hypothetical protein*	Unknown	Unknown

RCAP_rcc02190	-0.181	0.481	-1.794	0.000	-0.015	0.980	*zntA2*	Metal, Ion, Cofactor Transport	Zinc Transport
RCAP_rcc02191	-0.490	0.104	-0.472	0.064	-0.052	0.930	*MerR family transcriptional regulator*	Signal Transduction	Transcription Regulator
RCAP_rcc02192	-0.158	0.407	-0.276	0.076	0.152	0.518	*major facilitator superfamily protein*	Unknown	Unknown
RCAP_rcc02193	-0.982	0.000	-0.176	0.535	0.530	0.182	*DNA-3-methyladenine glycosylase II*	Replication, Recombination and Repair	Replication
RCAP_rcc02194	-0.715	0.001	-0.029	0.863	0.510	0.024	*phospholipase/carboxylesterase*	Unknown	Unknown
RCAP_rcc02195	0.188	0.638	-2.292	0.000	-0.161	0.637	*HNH endonuclease*	Unknown	Unknown
RCAP_rcc02196	-0.569	0.041	0.119	0.528	0.355	0.298	*FkbM family methyltransferase*	Unknown	Unknown
RCAP_rcc02197	0.918	0.000	-2.765	0.000	0.131	0.629	*two component AraC family transcriptional regulator*	Signal Transduction	Transcription Regulator
RCAP_rcc02198	1.589	0.000	-2.404	0.000	0.056	0.874	*histidine kinase*	Signal Transduction	Kinase/Phosphorelay
RCAP_rcc02199	0.085	0.843	-6.118	0.000	0.535	0.171	*pflA1*	Post-translational Modification, Assembly and Chaperones	Propanediol Metabolism
RCAP_rcc02200	0.332	0.448	-5.724	0.000	0.232	0.591	*hypothetical protein*	Energy Metabolism	Propanediol Metabolism
RCAP_rcc02201	0.095	0.799	-6.438	0.000	0.815	0.007	*adhE*	Energy Metabolism	Propanediol Metabolism
RCAP_rcc02202	-0.058	NA	-5.459	0.000	0.125	NA	*hypothetical protein*	Energy Metabolism	Propanediol Metabolism
RCAP_rcc02203	-0.253	NA	-6.061	0.000	0.408	NA	*eutN*	Energy Metabolism	Propanediol Metabolism
RCAP_rcc02204	0.082	NA	-5.849	0.000	0.198	NA	*hypothetical protein*	Energy Metabolism	Propanediol Metabolism
RCAP_rcc02205	-0.274	NA	-6.378	0.000	0.227	NA	*eutJ*	Energy Metabolism	Propanediol Metabolism
RCAP_rcc02206	-0.051	0.921	-6.721	0.000	0.679	0.071	*pduL*	Energy Metabolism	Propanediol Metabolism
RCAP_rcc02207	0.032	NA	-6.270	0.000	0.552	0.174	*pduA1*	Energy Metabolism	Propanediol Metabolism
RCAP_rcc02208	-0.438	0.306	-7.180	0.000	1.359	0.000	*pduB*	Energy Metabolism	Propanediol Metabolism
RCAP_rcc02209	0.454	0.253	-6.524	0.000	0.512	0.214	*pduA2*	Energy Metabolism	Propanediol Metabolism
RCAP_rcc02210	0.181	0.703	-6.236	0.000	0.681	0.062	*adh2*	Energy Metabolism	Propanediol Metabolism
RCAP_rcc02211	0.260	0.312	-7.773	0.000	1.009	0.000	*pflD*	Carbohydrate Metabolism	Butanoate metabolism
RCAP_rcc02212	0.315	0.470	-7.633	0.000	0.716	0.047	*ThiJ/PfpI family protein*	Unknown	Unknown
RCAP_rcc02213	-0.079	NA	-7.332	0.000	0.535	0.169	*hypothetical protein*	Unknown	Unknown
RCAP_rcc02214	-0.705	0.061	-6.330	0.000	0.493	0.218	*ackA2*	Energy Metabolism	Reductive carboxylate cycle (CO2 fixation)
RCAP_rcc02215	-0.134	0.531	-0.349	0.046	0.327	0.204	*sulfotransferase*	Unknown	Unknown
RCAP_rcc02216	-0.149	0.592	-0.216	0.365	-0.021	0.972	*hypothetical protein*	Unknown	Unknown
RCAP_rcc02217	-0.111	0.654	0.376	0.004	0.210	0.369	*group 1 glycosyl transferase*	Unknown	Unknown
RCAP_rcc02218	1.188	0.002	-0.827	0.001	0.784	0.033	*modB2*	Metal and Ion Transport	Unknown
RCAP_rcc02219	2.292	0.000	-0.690	0.008	0.875	0.008	*modA2*	Metal and Ion Transport	Unknown
RCAP_rcc02220	2.020	0.000	-0.296	0.232	0.810	0.019	*modC2*	Metal and Ion Transport	Unknown
RCAP_rcc02221	-0.005	NA	-0.868	0.003	0.668	0.079	*guaA2*	Xenobiotics Biodegradation and Metabolism	Drug metabolism - other enzymes
RCAP_rcc02222	-0.204	0.648	0.689	0.008	0.298	0.507	*ntaA*	Unknown	Unknown
RCAP_rcc02223	0.143	0.767	0.665	0.093	-0.007	NA	*ABC transporter permease*	Metal and Ion Transport	Unknown
RCAP_rcc02224	-0.253	0.577	0.538	0.074	0.072	0.894	*ABC transporter substrate-binding protein*	Metal and Ion Transport	Unknown
RCAP_rcc02225	0.506	0.234	0.325	0.437	-0.207	0.641	*ABC transporter ATP-binding protein*	Metal and Ion Transport	Unknown
RCAP_rcc02226	-0.362	0.405	0.672	0.252	0.049	0.922	*hypothetical protein*	Unknown	Unknown
RCAP_rcc02227	-0.240	0.597	0.886	0.124	-0.117	NA	*hypothetical protein*	Unknown	Unknown
RCAP_rcc02228	-0.740	NA	0.129	0.832	0.382	NA	*hypothetical protein*	Unknown	Unknown
RCAP_rcc02229	0.000	NA	-0.882	0.130	0.035	NA	*hypothetical protein*	Unknown	Unknown
RCAP_rcc02230	-0.053	NA	0.919	0.181	0.219	NA	*hypothetical protein*	Unknown	Unknown
RCAP_rcc02231	#VALUE!	NA	-0.445	0.488	#VALUE!	NA	*hypothetical protein*	Unknown	Unknown
RCAP_rcc02232	-0.395	0.364	-0.776	0.068	0.082	NA	*FAD dependent oxidoreductase*	Energy Metabolism	Unknown
RCAP_rcc02233	0.052	NA	-0.118	0.885	0.029	NA	*hypothetical protein*	Unknown	Unknown
RCAP_rcc02234	-0.047	NA	0.124	0.868	0.036	NA	*oxidoreductase/nitrogenase subunit 1*	Energy Metabolism	Nitrogen metabolism
RCAP_rcc02235	-0.259	NA	-0.269	0.733	0.064	NA	*oxidoreductase/nitrogenase subunit 1*	Energy Metabolism	Nitrogen metabolism
RCAP_rcc02236	-0.301	0.503	0.508	0.300	0.175	NA	*nifH2*	Energy Metabolism	Nitrogen metabolism
RCAP_rcc02237	0.060	0.861	0.323	0.283	-0.223	0.579	*radical SAM family protein*	Unknown	Unknown
RCAP_rcc02238	0.028	0.944	-0.372	0.066	-0.108	0.734	*xsc*	Metabolism of Other Amino Acids	Taurine and hypotaurine metabolism
RCAP_rcc02239	0.095	0.730	-0.586	0.011	0.190	0.561	*GntR family transcriptional regulator*	Signal Transduction	Transcription Regulator
RCAP_rcc02240	0.272	0.275	-0.221	0.239	0.394	0.127	*tpa*	Metabolism of Other Amino Acids	Taurine and hypotaurine metabolism
RCAP_rcc02241	-0.129	0.751	1.254	0.000	-0.139	0.771	*tauA*	Metal and Ion Transport	Unknown
RCAP_rcc02242	0.262	NA	1.020	0.054	0.031	NA	*tauB*	Metal and Ion Transport	Unknown
RCAP_rcc02243	-0.313	0.428	0.777	0.032	-0.106	0.839	*tauC*	Metal and Ion Transport	Unknown
RCAP_rcc02244	-0.136	0.738	0.412	0.173	-0.085	0.877	*sufS2*	Metabolism of Cofactors, Coenzymes and Vitamins	Thiamine metabolism
RCAP_rcc02245	-0.319	0.407	0.091	0.837	0.406	0.334	*major membrane protein I*	Unknown	Unknown
RCAP_rcc02246	-0.526	0.208	-0.503	0.198	-0.280	0.537	*cysE2*	Energy Metabolism	Sulfur metabolism
RCAP_rcc02247	-0.001	NA	-0.971	0.151	-0.035	NA	*rhodanese domain-containing protein*	Metal and Ion Transport	Unknown
RCAP_rcc02248	0.401	0.139	-2.281	0.000	-0.390	0.050	*aldo/keto reductase family oxidoreductase*	Energy Metabolism	Unknown
RCAP_rcc02249	-0.761	0.007	-0.221	0.430	-0.317	0.482	*AraC family transcriptional regulator*	Signal Transduction	Transcription Regulator
RCAP_rcc02250	-0.809	0.012	-0.165	0.583	0.380	0.354	*hypothetical protein*	Unknown	Unknown
RCAP_rcc02251	-0.187	NA	-0.323	0.674	0.029	NA	*hypothetical protein*	Unknown	Unknown
RCAP_rcc02252	-0.278	0.476	-0.397	0.182	0.410	0.316	*kdpA*	Metal and Ion Transport	Unknown
RCAP_rcc02253	0.197	0.659	-0.360	0.369	-0.217	0.632	*kdpB*	Metal and Ion Transport	Unknown
RCAP_rcc02254	0.110	0.823	0.292	0.526	-0.070	0.899	*kdpC*	Metal and Ion Transport	Unknown
RCAP_rcc02255	-0.318	0.218	-0.413	0.035	-0.151	0.702	*kdpD*	Signal Transduction	Kinase/Phosphorelay
RCAP_rcc02256	-0.201	0.568	-0.276	0.376	0.227	0.563	*kdpE*	Signal Transduction	Transcription Regulator
RCAP_rcc02257	-0.092	0.826	-0.346	0.244	0.139	0.764	*DegT/DnrJ/EryC1/StrS family aminotransferase*	Cell Envelope Biosynthesis	Cell Wall Biosynthesis
RCAP_rcc02258	0.299	0.268	-0.008	0.961	-0.260	0.346	*transferase hexapeptide repeat domain-containing protein*	Unknown	Unknown
RCAP_rcc02259	0.018	0.963	-0.213	0.389	0.221	0.603	*WbqC-like protein family*	Unknown	Unknown
RCAP_rcc02260	-0.503	0.117	0.555	0.014	0.385	0.326	*type 11 family methyltransferase*	Unknown	Unknown
RCAP_rcc02261	-0.076	0.810	0.608	0.013	-0.004	0.991	*hypothetical protein*	Unknown	Unknown
RCAP_rcc02262	-0.555	0.076	0.278	0.390	0.394	0.338	*hypothetical protein*	Unknown	Unknown
RCAP_rcc02263	-0.716	0.030	0.356	0.091	0.309	0.464	*hypothetical protein*	Unknown	Unknown
RCAP_rcc02264	-1.035	0.003	0.144	0.690	0.381	0.383	*hypothetical protein*	Unknown	Unknown
RCAP_rcc02265	-0.246	0.380	0.659	0.045	0.007	0.991	*short-chain dehydrogenase/reductase family oxidoreductase*	Unknown	Unknown
RCAP_rcc02266	0.186	0.548	0.549	0.027	-0.099	0.839	*potC2*	Metal and Ion Transport	Unknown

RCAP_rcc02267	0.304	0.242	1.138	0.000	-0.016	0.974	*potB3*	Metal and Ion Transport	Unknown
RCAP_rcc02268	0.550	0.019	1.130	0.002	0.050	0.888	*potD3*	Amino Acid Metabolism	Amino Acid Transport
RCAP_rcc02269	0.026	0.919	0.599	0.005	-0.329	0.275	*potA3*	Amino Acid Metabolism	Amino Acid Transport
RCAP_rcc02270	-0.251	0.285	0.052	0.864	0.005	0.991	*LuxR family transcriptional regulator*	Signal Transduction	Transcription Regulator
RCAP_rcc02271	-0.251	0.520	0.034	0.932	0.153	0.742	*speB2*	Amino Acid Metabolism	Arginine and proline metabolism
RCAP_rcc02272	-0.437	0.282	0.014	0.971	0.317	0.482	*LysR family transcriptional regulator*	Signal Transduction	Transcription Regulator
RCAP_rcc02273	-0.077	0.874	-3.637	0.000	0.205	0.664	*hypothetical protein*	Unknown	Unknown
RCAP_rcc02274	-0.673	0.085	-2.648	0.000	-0.050	0.939	*pip*	Amino Acid Metabolism	Arginine and proline metabolism
RCAP_rcc02275	0.368	0.102	-2.801	0.000	-0.712	0.000	*oppA2*	Amino Acid Metabolism	Amino Acid Transport
RCAP_rcc02276	0.381	0.241	-1.601	0.000	-0.267	0.434	*oppB2*	Metal and Ion Transport	Unknown
RCAP_rcc02277	-0.348	0.275	-1.578	0.000	0.135	0.747	*oppC2*	Metal and Ion Transport	Unknown
RCAP_rcc02278	-0.255	0.393	-1.401	0.000	-0.103	0.816	*oppD2*	Unknown	Unknown
RCAP_rcc02279	-0.337	NA	-0.366	0.556	0.233	NA	*dmsC*	Energy Metabolism	Aerobic/Anaerobic Respiration
RCAP_rcc02280	-0.261	0.563	0.406	0.463	-0.158	NA	*dmsB*	Energy Metabolism	Aerobic/Anaerobic Respiration
RCAP_rcc02281	-0.221	0.550	-0.023	0.951	-0.421	0.180	*dmsA*	Energy Metabolism	Aerobic/Anaerobic Respiration
RCAP_rcc02282	-0.431	NA	-1.585	0.003	-0.385	0.360	*hypothetical protein*	Unknown	Unknown
RCAP_rcc02283	-0.114	NA	-1.308	0.017	-0.337	0.440	*hypothetical protein*	Unknown	Unknown
RCAP_rcc02284	-0.199	NA	-0.454	0.436	0.104	0.821	*hypothetical protein*	Unknown	Unknown
RCAP_rcc02285	0.567	0.174	-1.495	0.001	-0.945	0.006	*hypothetical protein*	Unknown	Unknown
RCAP_rcc02286	0.068	NA	-1.070	0.002	-0.211	0.651	*divalent ion symporter family*	Metal and Ion Transport	Replication
RCAP_rcc02287	-0.231	0.423	-0.698	0.003	0.278	0.486	*yieF*	Energy Metabolism	Unknown
RCAP_rcc02288	0.571	0.028	-0.993	0.000	-0.956	0.000	*hypothetical protein*	Unknown	Unknown
RCAP_rcc02289	0.531	0.060	-1.567	0.000	-0.268	0.526	*two-component response regulator receiver protein*	Signal Transduction	Transcription Regulator
RCAP_rcc02290	-1.002	0.012	-1.087	0.000	0.117	0.811	*hypothetical protein*	Unknown	Unknown
RCAP_rcc02291	0.154	0.725	-1.393	0.000	-0.474	0.214	*ECF family RNA polymerase sigma factor*	Transcription	Unknown
RCAP_rcc02292	-0.299	0.368	-1.210	0.000	-0.118	0.797	*signal transduction histidine kinase*	Signal Transduction	Kinase/Phosphorelay
RCAP_rcc02293	-0.717	0.025	-1.013	0.003	-1.020	0.002	*hypothetical protein*	Unknown	Unknown
RCAP_rcc02294	-0.485	0.042	-1.417	0.000	0.118	0.750	*signal transduction histidine kinase*	Signal Transduction	Kinase/Phosphorelay
RCAP_rcc02295	0.017	0.940	-0.065	0.651	-0.109	0.632	*proB*	Amino Acid Metabolism	Arginine and proline metabolism
RCAP_rcc02296	0.133	0.544	0.261	0.071	-0.212	0.077	*GTP-binding protein Obg/CgtA*	Unknown	Unknown
RCAP_rcc02297	-0.959	0.009	1.270	0.000	0.310	0.497	*GNAT family acetyltransferase*	Cell Division	Chromosome Partitioning
RCAP_rcc02298	0.108	0.682	1.120	0.000	-0.108	0.778	*GNAT family acetyltransferase*	Cell Division	Chromosome Partitioning
RCAP_rcc02299	0.344	0.148	0.349	0.146	-0.247	0.454	*rpmA*	Translation, ribosomal structure and biogenesis	Unknown
RCAP_rcc02300	0.372	0.089	0.234	0.414	-0.133	0.590	*rplU*	Translation, ribosomal structure and biogenesis	Unknown
RCAP_rcc02301	0.504	0.005	0.442	0.019	-0.388	0.188	*hypothetical protein*	Unknown	Unknown
RCAP_rcc02302	-0.059	0.857	0.311	0.047	0.040	0.828	*engA*	Unknown	Unknown
RCAP_rcc02303	-0.142	0.437	-0.124	0.283	0.044	0.876	*PQQ enzyme repeat family protein*	Unknown	Unknown
RCAP_rcc02304	0.249	0.273	0.152	0.486	-0.261	0.269	*hypothetical protein*	Unknown	Unknown
RCAP_rcc02305	-0.074	0.698	0.144	0.184	0.065	0.686	*heme ABC transporter ATP-binding/permease*	Defense Mechanisms	Unknown
RCAP_rcc02306	-0.093	0.715	-0.487	0.100	0.237	0.442	*LysM domain-containing protein*	Unknown	Unknown
RCAP_rcc02307	0.429	0.089	-0.012	0.962	0.008	0.986	*decarboxylase*	Unknown	Unknown
RCAP_rcc02308	0.105	0.719	0.095	0.699	-0.021	0.963	*rarD*	Unknown	Unknown
RCAP_rcc02309	-0.266	0.218	0.272	0.302	-0.349	0.200	*tnaA*	Amino Acid Metabolism	Tryptophan metabolism
RCAP_rcc02310	0.488	0.221	-0.804	0.074	-0.037	NA	*hypothetical protein*	Unknown	Unknown
RCAP_rcc02311	0.722	0.006	-1.593	0.000	-0.315	0.344	*sodB*	Metal and Ion Transport	Unknown
RCAP_rcc02312	-0.321	0.186	0.112	0.488	0.259	0.301	*hypothetical protein*	Unknown	Unknown
RCAP_rcc02313	-0.554	0.013	-1.156	0.000	0.496	0.018	*soxG*	Amino Acid Metabolism	Glycine, serine and threonine metabolism
RCAP_rcc02314	0.064	0.774	-0.769	0.000	0.037	0.900	*soxA*	Amino Acid Metabolism	Glycine, serine and threonine metabolism
RCAP_rcc02315	-0.147	0.437	-0.656	0.000	0.069	0.801	*soxD*	Amino Acid Metabolism	Glycine, serine and threonine metabolism
RCAP_rcc02316	0.271	NA	-0.530	0.000	0.053	0.877	*soxB*	Amino Acid Metabolism	Glycine, serine and threonine metabolism
RCAP_rcc02317	-0.247	0.401	-0.734	0.000	0.338	0.388	*cycH*	Energy Metabolism	Aerobic/Anaerobic Respiration
RCAP_rcc02318	-0.731	0.040	-0.525	0.001	0.716	0.033	*yqgF*	Replication, Recombination and Repair	Unknown
RCAP_rcc02319	-0.021	0.942	-0.366	0.062	-0.076	0.826	*hypothetical protein*	Unknown	Unknown
RCAP_rcc02320	0.328	0.103	-0.142	0.418	-0.149	0.526	*hypothetical protein*	Unknown	Unknown
RCAP_rcc02321	1.120	0.000	-1.636	0.000	-0.565	0.130	*hypothetical protein*	Unknown	Unknown
RCAP_rcc02322	0.108	0.735	0.968	0.000	-0.088	0.727	*prfC*	Translation, ribosomal structure and biogenesis	Unknown
RCAP_rcc02323	0.341	0.103	-0.344	0.106	-0.432	0.063	*hypothetical protein*	Unknown	Unknown
RCAP_rcc02324	0.119	0.550	-0.146	0.243	-0.084	0.736	*dgt*	Nucleotide Metabolism	Purine metabolism
RCAP_rcc02325	0.574	0.008	-0.028	0.852	-0.195	0.358	*HesB/YadR/YfhF family protein*	Unknown	Unknown
RCAP_rcc02326	0.366	0.189	0.219	0.401	-0.274	0.340	*phage integrase*	Replication, Recombination and Repair	Phage Interaction
RCAP_rcc02327	0.281	0.392	0.498	0.137	0.153	0.728	*HK97 family phage portal protein*	Replication, Recombination and Repair	Phage Interaction
RCAP_rcc02328	0.038	NA	0.315	0.678	-0.172	NA	*hypothetical protein*	Unknown	Unknown
RCAP_rcc02329	-0.498	0.168	0.598	0.169	-0.305	0.504	*HK97 family phage major capsid protein*	Replication, Recombination and Repair	Phage Interaction
RCAP_rcc02330	-0.336	0.443	0.484	0.203	0.187	0.696	*hypothetical protein*	Unknown	Unknown
RCAP_rcc02331	0.366	0.404	0.134	0.859	-0.426	0.316	*HNH nuclease*	Defense Mechanisms	Unknown
RCAP_rcc02332	-0.034	0.940	0.846	0.064	-0.336	0.393	*hypothetical protein*	Unknown	Unknown
RCAP_rcc02333	0.279	0.399	-1.074	0.000	-0.140	0.716	*hypothetical protein*	Unknown	Unknown
RCAP_rcc02334	-0.453	0.198	-0.514	0.253	-0.142	0.760	*hypothetical protein*	Unknown	Unknown
RCAP_rcc02335	-0.171	NA	0.047	0.956	0.024	NA	*hypothetical protein*	Unknown	Unknown
RCAP_rcc02336	0.102	NA	-0.131	0.864	-0.148	0.738	*hypothetical protein*	Unknown	Unknown
RCAP_rcc02337	-0.322	NA	0.528	0.430	-0.252	NA	*hypothetical protein*	Unknown	Unknown
RCAP_rcc02338	-0.509	0.127	0.360	0.359	-0.080	0.887	*hypothetical protein*	Unknown	Unknown
RCAP_rcc02339	0.547	0.065	0.102	0.690	-0.386	0.248	*hypothetical protein*	Unknown	Unknown
RCAP_rcc02340	0.132	NA	0.210	0.760	-0.246	0.593	*phage terminase small subunit*	Replication, Recombination and Repair	Phage Interaction
RCAP_rcc02341	-0.153	0.722	-0.196	0.629	-0.091	0.869	*phage terminase large subunit*	Replication, Recombination and Repair	Phage Interaction
RCAP_rcc02342	-0.644	0.068	-0.103	0.791	-0.153	0.736	*hypothetical protein*	Unknown	Unknown
RCAP_rcc02343	0.350	0.064	0.144	0.506	-0.457	0.045	*hypothetical protein*	Unknown	Unknown

RCAP_rcc02344	0.469	0.050	-0.453	0.020	-0.296	0.133	*hypothetical protein*	Unknown	Unknown
RCAP_rcc02345	0.407	0.071	0.144	0.449	-0.216	0.549	*hypothetical protein*	Unknown	Unknown
RCAP_rcc02346	0.642	0.003	0.429	0.001	-0.219	0.595	*hypothetical protein*	Unknown	Unknown
RCAP_rcc02347	-0.303	0.289	0.480	0.071	0.163	0.696	*hypothetical protein*	Unknown	Unknown
RCAP_rcc02348	0.033	0.894	0.958	0.000	-0.142	0.561	*cold-shock DNA-binding domain-containing protein*	Transcription	Unknown
RCAP_rcc02349	-0.012	0.961	-0.352	0.007	0.009	0.975	*pdxH*	Metabolism of Cofactors, Coenzymes and Vitamins	Vitamin B6 metabolism
RCAP_rcc02350	0.615	0.006	0.278	0.165	-0.244	0.499	*fabI1*	Lipid Metabolism	Fatty acid biosynthesis
RCAP_rcc02351	0.064	0.838	0.182	0.245	-0.066	0.801	*LysE family transporter*	Unknown	Unknown
RCAP_rcc02352	0.376	0.061	0.009	0.966	-0.184	0.556	*gpt*	Nucleotide Metabolism	Purine metabolism
RCAP_rcc02353	-0.356	0.209	-0.044	0.817	0.137	0.558	*class I/II aminotransferase*	Amino Acid Metabolism	Unknown
RCAP_rcc02354	-0.414	0.132	0.041	0.752	0.265	0.189	*ppiD*	Post-translational Modification, Assembly and Chaperones	Unknown
RCAP_rcc02355	-0.249	0.087	-0.101	0.378	0.133	0.372	*trpE*	Amino Acid Metabolism	Phenylalanine, tyrosine and tryptophan biosynthesis
RCAP_rcc02356	-0.405	0.172	-0.716	0.000	-0.417	0.231	*hypothetical protein*	Unknown	Unknown
RCAP_rcc02357	0.642	0.002	0.169	0.380	-0.215	0.411	*trpG*	Metabolism of Cofactors, Coenzymes and Vitamins	Folate biosynthesis
RCAP_rcc02358	-0.276	0.279	-0.032	0.836	0.383	0.187	*trpD*	Amino Acid Metabolism	Phenylalanine, tyrosine and tryptophan biosynthesis
RCAP_rcc02359	-0.600	0.005	0.141	0.318	0.504	0.084	*trpC*	Amino Acid Metabolism	Phenylalanine, tyrosine and tryptophan biosynthesis
RCAP_rcc02360	-0.443	0.014	0.217	0.296	0.169	0.564	*moaC1*	Metabolism of Cofactors, Coenzymes and Vitamins	Unknown
RCAP_rcc02361	0.371	0.033	-0.059	0.746	-0.315	0.054	*lexA*	Transcription	Unknown
RCAP_rcc02362	-0.836	0.011	0.840	0.000	0.198	0.678	*competence protein*	Unknown	Unknown
RCAP_rcc02363	0.259	0.234	0.149	0.389	-0.103	0.736	*gltX2*	Translation, ribosomal structure and biogenesis	Aminoacyl-tRNA biosynthesis
RCAP_rcc02364	0.307	0.257	0.018	0.959	-0.091	0.771	*gltA*	Carbohydrate Metabolism	TCA Cycle
RCAP_rcc02365	-0.321	0.275	0.160	0.292	0.083	0.831	*mdoG2*	Unknown	Unknown
RCAP_rcc02366	0.143	0.638	0.045	0.874	-0.477	0.216	*NnrS family protein*	Metal and Ion Transport	Unknown
RCAP_rcc02367	0.060	0.884	0.033	0.952	-0.042	0.936	*calcium-binding protein*	Unknown	Unknown
RCAP_rcc02368	-0.151	0.544	-0.594	0.037	0.179	0.632	*gph4*	Carbohydrate Metabolism	Starch and sucrose metabolism
RCAP_rcc02369	0.338	0.168	-0.834	0.000	0.062	0.839	*araB*	Carbohydrate Metabolism	Pentose and glucuronate interconversions
RCAP_rcc02370	0.024	0.922	-0.721	0.000	0.265	0.201	*rbtD*	Carbohydrate Metabolism	Pentose and glucuronate interconversions
RCAP_rcc02371	0.309	0.169	-0.595	0.000	0.097	0.731	*monosacharide ABC transporter permease*	Carbohydrate Metabolism	Aerobic/Anaerobic Respiration
RCAP_rcc02372	0.161	0.563	-0.697	0.003	0.106	0.652	*monosacharide ABC transporter ATP-binding protein*	Carbohydrate Metabolism	Aerobic/Anaerobic Respiration
RCAP_rcc02373	0.503	0.035	-1.210	0.000	0.024	0.957	*monosacharide ABC transporter periplasmic monosacharide-binding pro*	Carbohydrate Metabolism	Aerobic/Anaerobic Respiration
RCAP_rcc02374	-0.639	0.001	-0.097	0.713	-0.039	0.946	*LacI family transcriptional regulator*	Signal Transduction	Transcription Regulator
RCAP_rcc02375	-0.497	0.226	0.116	0.606	0.303	0.506	*exbB*	Trafficking and Secretion	Trafficing
RCAP_rcc02376	0.189	0.581	-0.041	0.894	-0.323	0.445	*exbD1*	Trafficking and Secretion	Trafficing
RCAP_rcc02377	-0.442	0.303	0.021	0.951	0.630	0.113	*tonB*	Cell Envelope Biosynthesis	Cell Wall Biosynthesis
RCAP_rcc02378	#VALUE!	NA	0.110	0.894	#VALUE!	NA	*hypothetical protein*	Unknown	Unknown
RCAP_rcc02379	0.017	0.963	0.449	0.147	-0.684	0.016	*CobW/HypB/UreG family cobalamin biosynthesis protein*	Metabolism of Cofactors, Coenzymes and Vitamins	Cobalamin Biosynthesis
RCAP_rcc02380	0.013	0.956	0.205	0.115	0.004	0.989	*ftsI*	Glycan Biosynthesis and Metabolism	Peptidoglycan biosynthesis
RCAP_rcc02381	0.247	0.205	0.231	0.150	-0.122	0.689	*hypothetical protein*	Unknown	Unknown
RCAP_rcc02382	-0.258	0.243	0.569	0.000	0.170	0.480	*mraW*	Cell Envelope Biosynthesis	Cell Wall Biosynthesis
RCAP_rcc02383	-0.389	0.136	0.507	0.027	-0.120	0.579	*mraZ*	Unknown	Unknown
RCAP_rcc02384	-0.072	0.755	-0.144	0.488	0.075	0.736	*Mrp/NBP35 family protein*	Cell Division	Chromosome Partitioning
RCAP_rcc02385	0.498	0.069	-0.200	0.649	-0.910	0.000	*hypothetical protein*	Unknown	Unknown
RCAP_rcc02386	-0.165	0.454	0.154	0.357	-0.188	0.575	*hypothetical protein*	Unknown	Unknown
RCAP_rcc02387	-0.113	0.699	0.088	0.665	0.135	0.526	*hypothetical protein*	Unknown	Unknown
RCAP_rcc02388	0.454	0.033	-0.165	0.427	-0.586	0.002	*ispZ*	Cell Division	Chromosome Partitioning
RCAP_rcc02389	-0.093	0.698	-0.142	0.326	0.167	0.466	*ftsY*	Trafficking and Secretion	Trafficing
RCAP_rcc02390	0.455	0.042	5.003	0.000	-0.035	0.948	*alkane 1-monooxygenase*	Metabolism of Cofactors, Coenzymes and Vitamins	Retinol metabolism
RCAP_rcc02391	0.588	0.039	-0.463	0.008	-0.075	0.782	*hypothetical protein*	Unknown	Unknown
RCAP_rcc02392	-0.161	0.520	0.128	0.478	0.258	0.354	*xseA*	Replication, Recombination and Repair	Replication
RCAP_rcc02393	-0.307	0.417	0.250	0.163	0.343	0.117	*purD*	Nucleotide Metabolism	Purine metabolism
RCAP_rcc02394	-0.670	0.010	-0.774	0.001	0.471	0.157	*sdaA*	Amino Acid Metabolism	Cysteine and methionine metabolism
RCAP_rcc02395	-0.771	0.013	1.372	0.000	0.554	0.167	*hypothetical protein*	Unknown	Unknown
RCAP_rcc02396	0.522	0.025	0.200	0.241	-0.249	0.462	*rpiA*	Carbohydrate Metabolism	Pentose phosphate pathway
RCAP_rcc02397	0.100	0.652	0.287	0.066	-0.100	0.736	*hypothetical protein*	Unknown	Unknown
RCAP_rcc02398	0.225	0.174	0.174	0.226	-0.182	0.326	*gor*	Sulfur Metabolism	Glutathione metabolism
RCAP_rcc02399	0.217	0.254	0.342	0.022	-0.006	0.989	*hflK*	Post-translational Modification, Assembly and Chaperones	Unknown
RCAP_rcc02400	0.495	0.014	0.279	0.043	-0.140	0.693	*hflC*	Post-translational Modification, Assembly and Chaperones	Unknown
RCAP_rcc02401	0.236	0.511	0.041	0.909	0.047	0.926	*hypothetical protein*	Unknown	Unknown
RCAP_rcc02402	1.317	0.000	-0.262	0.309	-0.211	0.300	*degP*	Unknown	Unknown
RCAP_rcc02403	-0.387	0.324	-0.428	0.129	0.190	0.694	*peptidoglycan-binding domain 1 protein*	Unknown	Unknown
RCAP_rcc02404	0.248	0.425	0.013	0.959	0.054	0.857	*fdx*	Energy Metabolism	Aerobic/Anaerobic Respiration
RCAP_rcc02405	0.571	0.016	-0.027	NA	-0.220	0.506	*purU*	Carbohydrate Metabolism	Glyoxylate and dicarboxylate metabolism
RCAP_rcc02406	-0.346	0.053	0.528	0.006	0.245	0.030	*pgi*	Carbohydrate Metabolism	Glycolysis / Gluconeogenesis
RCAP_rcc02407	-0.613	0.019	0.767	0.010	0.457	0.072	*pgl*	Carbohydrate Metabolism	Pentose phosphate pathway
RCAP_rcc02408	-0.006	0.975	0.812	0.005	0.097	0.688	*zwf*	Carbohydrate Metabolism	Pentose phosphate pathway
RCAP_rcc02409	-0.257	0.462	0.268	0.291	0.138	0.701	*hypothetical protein*	Unknown	Unknown
RCAP_rcc02410	-0.646	0.103	0.137	0.592	0.747	0.020	*hypothetical protein*	Unknown	Unknown
RCAP_rcc02411	-0.388	0.103	1.045	0.001	0.121	0.608	*edd*	Carbohydrate Metabolism	Pentose phosphate pathway
RCAP_rcc02412	-0.388	0.137	0.826	0.000	0.278	0.309	*eda*	Carbohydrate Metabolism	Glyoxylate and dicarboxylate metabolism
RCAP_rcc02413	0.422	0.143	0.055	0.806	-0.964	0.000	*hypothetical protein*	Unknown	Unknown
RCAP_rcc02414	-0.059	0.785	0.243	0.083	0.045	0.831	*glnE*	Unknown	Unknown
RCAP_rcc02415	1.815	0.000	2.639	0.000	0.429	0.179	*hypothetical protein*	Unknown	Unknown
RCAP_rcc02416	0.067	0.824	0.138	0.616	-0.306	0.110	*ate*	Post-translational Modification, Assembly and Chaperones	Unknown
RCAP_rcc02417	0.590	0.007	0.573	0.034	-0.138	0.692	*dctP2*	Secondary metabolites biosynthesis, transport, and catabolism	Unknown
RCAP_rcc02418	-0.130	0.596	-0.024	0.920	0.079	0.839	*short-chain dehydrogenase/reductase family oxidoreductase*	Lipid transport and metabolism	Unknown
RCAP_rcc02419	-0.069	0.828	-0.195	0.292	0.025	0.964	*dihydroneopterin aldolase*	Unknown	Unknown
RCAP_rcc02420	-0.131	0.691	-0.305	0.059	0.352	0.358	*hypothetical protein*	Unknown	Unknown

RCAP_rcc02421	-0.260	0.228	-0.747	0.000	0.173	0.391	*bglX*	Photosynthesis	Phenylpropanoid biosynthesis
RCAP_rcc02422	-0.442	0.064	-0.959	0.000	0.359	0.169	*hypothetical protein*	Unknown	Unknown
RCAP_rcc02423	0.098	0.737	-0.651	0.000	0.102	0.673	*ABC transporter ATP-binding protein/permease*	Unknown	Unknown
RCAP_rcc02424	-0.426	0.282	-0.096	0.795	0.392	0.316	*short-chain dehydrogenase/reductase family oxidoreductase*	Metabolism of Cofactors, Coenzymes and Vitamins	Biotin metabolism
RCAP_rcc02425	-0.440	0.184	0.087	0.624	0.428	0.279	*surE*	Metabolism of Cofactors, Coenzymes and Vitamins	Nicotinate and nicotinamide metabolism
RCAP_rcc02426	-0.755	0.007	0.081	0.648	0.469	0.122	*pcm1*	Post-translational Modification, Assembly and Chaperones	Unknown
RCAP_rcc02427	-0.656	0.015	-0.058	0.753	0.525	0.056	*M23 family peptidase*	Post-translational Modification, Assembly and Chaperones	Peptidase
RCAP_rcc02428	-0.277	0.227	-0.642	0.000	0.223	0.120	*TrkA domain transport protein*	Metal and Ion Transport	Unknown
RCAP_rcc02429	-0.606	0.008	-0.519	0.000	0.352	0.135	*xthA1*	Replication, Recombination and Repair	Unknown
RCAP_rcc02430	-0.786	0.020	1.143	0.000	0.539	0.108	*nahG*	Xenobiotics Biodegradation and Metabolism	Naphthalene and anthracene degradation
RCAP_rcc02431	0.640	0.020	0.137	0.657	-0.195	0.530	*dksA1*	Signal Transduction	Kinase/Phosphorelay
RCAP_rcc02432	0.460	0.071	0.151	0.378	-0.173	0.589	*ATPase AAA*	Unknown	Unknown
RCAP_rcc02433	-0.380	0.082	-0.059	0.714	0.465	0.088	*hypothetical protein*	Unknown	Unknown
RCAP_rcc02434	0.029	0.910	-0.032	0.904	0.267	0.295	*cephalosporin hydroxylase*	Defense Mechanisms	Unknown
RCAP_rcc02435	-0.621	0.017	-0.027	0.864	0.710	0.001	*family 2 glycosyl transferase*	Unknown	Unknown
RCAP_rcc02436	0.380	0.142	-0.073	0.684	-0.060	0.877	*phosphopantetheine-binding domain-containing protein*	Unknown	Unknown
RCAP_rcc02437	-0.398	0.175	-0.157	0.462	0.514	0.013	*cytochrome P450 family protein*	Energy Metabolism	Aerobic/Anaerobic Respiration
RCAP_rcc02438	-0.237	0.325	-0.154	0.510	0.239	0.465	*AMP-dependent synthetase and ligase*	Lipid Metabolism	Fatty acid metabolism
RCAP_rcc02439	-0.295	0.269	0.070	0.678	0.209	0.483	*hypothetical protein*	Unknown	Unknown
RCAP_rcc02440	-0.553	0.094	0.446	0.096	0.074	0.869	*AsnC/Lrp family transcriptional regulator*	Signal Transduction	Transcription Regulator
RCAP_rcc02441	0.502	0.039	-0.027	0.927	-0.277	0.354	*GntR family transcriptional regulator*	Signal Transduction	Transcription Regulator
RCAP_rcc02442	0.007	0.977	0.512	0.033	-0.190	0.477	*livJ*	Amino Acid Metabolism	Amino Acid Transport
RCAP_rcc02443	-0.018	0.956	-0.180	0.533	-0.313	0.229	*livH2*	Amino Acid Metabolism	Amino Acid Transport
RCAP_rcc02444	-0.203	0.536	0.195	0.512	-0.224	0.514	*livM2*	Amino Acid Metabolism	Amino Acid Transport
RCAP_rcc02445	0.129	0.709	0.378	0.186	-0.034	0.947	*livG2*	Amino Acid Metabolism	Amino Acid Transport
RCAP_rcc02446	-0.426	0.270	0.307	0.332	0.126	0.787	*livF2*	Amino Acid Metabolism	Amino Acid Transport
RCAP_rcc02447	-0.405	0.256	0.396	0.158	-0.114	0.811	*hyuE*	Amino Acid Metabolism	Unknown
RCAP_rcc02448	0.514	0.011	-0.556	0.027	-0.280	0.231	*aldH2*	Energy Metabolism	Limonene and pinene degradation
RCAP_rcc02449	0.334	0.258	-0.878	0.036	-0.133	0.751	*potC3*	Metal and Ion Transport	Unknown
RCAP_rcc02450	0.364	0.163	-0.840	0.042	-0.459	0.169	*potB4*	Metal and Ion Transport	Unknown
RCAP_rcc02451	0.244	0.520	-1.052	0.003	-0.158	0.736	*potA4*	Amino Acid Metabolism	Amino Acid Transport
RCAP_rcc02452	0.489	0.081	-1.764	0.000	-0.064	0.892	*potD4*	Amino Acid Metabolism	Amino Acid Transport
RCAP_rcc02453	-0.187	0.637	-2.259	0.000	0.339	0.442	*Fis family sigma54 specific transcriptional regulator*	Signal Transduction	Transcription Regulator
RCAP_rcc02454	-0.600	0.087	-2.258	0.000	0.045	0.947	*PAS domain-containing protein*	Unknown	Unknown
RCAP_rcc02455	-0.665	0.067	-0.662	0.002	0.786	0.028	*FAD dependent oxidoreductase*	Amino Acid Metabolism	Unknown
RCAP_rcc02456	0.221	0.338	0.029	0.863	0.020	0.948	*lolE*	Cell Envelope Biosynthesis	Cell Wall Biosynthesis
RCAP_rcc02457	-0.789	0.003	0.002	0.993	0.508	0.154	*lolD2*	Defense Mechanisms	Unknown
RCAP_rcc02458	0.149	0.571	-0.207	0.318	-0.053	0.919	*ArsC family protein*	Metal and Ion Transport	Unknown
RCAP_rcc02459	-0.195	0.506	0.661	0.000	0.286	NA	*XRE family transcriptional regulator*	Signal Transduction	Transcription Regulator
RCAP_rcc02460	0.536	0.004	1.203	0.000	-0.349	0.170	*cspA2*	Transcription	Unknown
RCAP_rcc02461	0.125	0.579	0.155	0.431	-0.115	NA	*thrS*	Translation, ribosomal structure and biogenesis	Aminoacyl-tRNA biosynthesis
RCAP_rcc02462	1.477	0.000	-0.579	0.086	-0.410	0.082	*hypothetical protein*	Unknown	Unknown
RCAP_rcc02463	0.481	0.066	1.475	0.000	0.035	0.948	*hemolysin-type calcium-binding repeat family protein*	Trafficking and Secretion	Secretion
RCAP_rcc02464	0.503	0.031	-0.004	0.989	-0.139	0.680	*proS*	Translation, ribosomal structure and biogenesis	Aminoacyl-tRNA biosynthesis
RCAP_rcc02465	0.094	0.752	0.625	0.000	0.004	0.991	*hypothetical protein*	Unknown	Unknown
RCAP_rcc02466	-0.148	0.676	0.152	0.431	0.009	0.985	*hypothetical protein*	Unknown	Unknown
RCAP_rcc02467	-0.637	0.020	0.047	0.877	0.315	0.441	*GntR family transcriptional regulator*	Signal Transduction	Transcription Regulator
RCAP_rcc02468	0.672	0.000	-0.019	0.930	-0.210	0.539	*thioesterase superfamily protein*	Lipid Metabolism	Unknown
RCAP_rcc02469	-0.037	0.893	-0.225	0.182	0.379	0.112	*ErfK/YbiS/YcfS/YnhG family protein*	Unknown	Unknown
RCAP_rcc02470	-0.899	0.008	-0.200	0.291	0.660	0.058	*selU*	Unknown	Unknown
RCAP_rcc02471	-0.552	0.133	0.152	0.707	-0.039	0.951	*hypothetical protein*	Unknown	Unknown
RCAP_rcc02472	-0.328	0.385	0.403	0.313	-0.349	0.411	*bsaA2*	Sulfur Metabolism	Glutathione metabolism
RCAP_rcc02473	0.333	0.260	0.464	0.041	-0.604	0.033	*TonB-dependent receptor plug domain-containing protein*	Metal and Ion Transport	Unknown
RCAP_rcc02474	-0.218	0.583	0.901	0.000	-0.025	0.965	*hypothetical protein*	Unknown	Unknown
RCAP_rcc02475	0.034	0.925	0.662	0.000	-0.157	0.605	*hypothetical protein*	Unknown	Unknown
RCAP_rcc02476	0.698	0.006	0.140	0.497	-0.257	0.462	*ppaC*	Energy Metabolism	Oxidative phosphorylation
RCAP_rcc02477	0.901	0.000	0.330	0.317	0.002	0.995	*groS*	Post-translational Modification, Assembly and Chaperones	Unknown
RCAP_rcc02478	0.636	0.003	0.301	0.310	0.116	0.819	*groL*	Post-translational Modification, Assembly and Chaperones	Unknown
RCAP_rcc02479	0.514	0.059	-1.422	0.000	0.189	0.688	*lipoprotein*	Predicted Function	Unknown
RCAP_rcc02480	0.133	0.480	0.438	0.000	-0.079	0.759	*polA*	Replication, Recombination and Repair	Replication
RCAP_rcc02481	0.343	0.161	0.189	0.464	-0.224	0.526	*hypothetical protein*	Unknown	Unknown
RCAP_rcc02482	0.365	0.050	0.049	0.795	-0.080	0.803	*HIT family protein*	Nucleotide Metabolism	Pyrimidine metabolism
RCAP_rcc02483	-0.103	0.654	-0.110	0.509	0.091	0.734	*family 14 glycosyl transferase*	Unknown	Unknown
RCAP_rcc02484	0.428	0.054	-0.094	0.492	-0.314	0.285	*hypothetical protein*	Unknown	Unknown
RCAP_rcc02485	-0.561	0.025	0.128	0.406	0.555	0.131	*type 12 family methyltransferase*	Unknown	Unknown
RCAP_rcc02486	0.057	0.774	0.255	0.109	0.033	0.840	*carbohydrate/purine kinase*	Carbohydrate Metabolism	Amino sugar and nucleotide sugar metabolism
RCAP_rcc02487	-0.190	0.392	0.639	0.012	0.157	0.582	*nth*	Replication, Recombination and Repair	Unknown
RCAP_rcc02488	-0.474	0.169	2.579	0.000	0.263	0.383	*hypothetical protein*	Unknown	Unknown
RCAP_rcc02489	-0.466	0.100	0.835	0.017	0.470	0.196	*ada*	Replication, Recombination and Repair	Unknown
RCAP_rcc02490	0.618	0.011	-0.471	0.137	-0.051	0.836	*OmpA family protein*	Cell Envelope Biosynthesis	Cell Wall Biosynthesis
RCAP_rcc02491	0.183	0.249	-0.323	0.016	-0.149	0.382	*GntR family transcriptional regulator*	Signal Transduction	Transcription Regulator
RCAP_rcc02492	-0.821	0.036	-0.214	0.397	0.447	0.205	*hypothetical protein*	Unknown	Unknown
RCAP_rcc02493	5.580	0.000	-0.312	0.039	-0.280	0.316	*Crp/Fnr family transcriptional regulator*	Signal Transduction	Transcription Regulator
RCAP_rcc02494	0.318	0.084	0.359	0.015	-0.161	0.477	*hemN2*	Metabolism of Cofactors, Coenzymes and Vitamins	Heme Biosynthesis
RCAP_rcc02495	0.238	0.376	-0.432	0.012	-0.691	0.000	*GTPase, EngC family*	Unknown	Unknown
RCAP_rcc02496	0.054	0.878	-0.113	0.615	0.147	0.569	*group 1 glycosyl transferase*	Cell Envelope Biosynthesis	Cell Wall Biosynthesis
RCAP_rcc02497	-0.159	0.497	-0.225	0.126	0.271	0.070	*ABC transporter ATP-binding protein*	Unknown	Unknown

RCAP_rcc02498	0.247	0.347	-0.167	0.224	-0.027	0.936	*ABC transporter permease*	Metal and Ion Transport	Unknown
RCAP_rcc02499	0.398	0.154	-0.098	0.492	-0.132	0.628	*ABC transporter permease*	Metal and Ion Transport	Unknown
RCAP_rcc02500	0.359	0.253	-0.319	0.032	-0.013	0.977	*ABC transporter periplasmic substrate-binding protein*	Amino Acid Metabolism	Amino Acid Transport
RCAP_rcc02501	0.891	0.001	0.524	0.040	-0.292	0.453	*cycY*	Energy Metabolism	Aerobic/Anaerobic Respiration
RCAP_rcc02502	0.117	0.549	-0.026	0.839	-0.041	0.852	*pheA*	Amino Acid Metabolism	Phenylalanine, tyrosine and tryptophan biosynthesis
RCAP_rcc02503	-0.093	0.792	0.328	0.011	0.241	0.435	*hypothetical protein*	Unknown	Unknown
RCAP_rcc02504	0.007	0.990	-0.146	0.677	0.297	0.499	*nudC*	Metabolism of Cofactors, Coenzymes and Vitamins	Nicotinate and nicotinamide metabolism
RCAP_rcc02505	-0.268	0.328	-0.078	0.791	-0.169	0.701	*tadA*	Xenobiotics Biodegradation and Metabolism	Atrazine degradation
RCAP_rcc02506	-0.819	0.004	0.594	0.000	0.104	NA	*rluB*	Nucleotide Metabolism	Pyrimidine metabolism
RCAP_rcc02507	0.237	0.350	-0.650	0.002	-0.193	0.482	*hypothetical protein*	Unknown	Unknown
RCAP_rcc02508	-0.131	0.631	-0.848	0.000	-0.056	0.869	*hypothetical protein*	Unknown	Unknown
RCAP_rcc02509	-0.422	0.020	-0.636	0.000	0.386	0.094	*TelA family toxic anion resistance protein*	Unknown	Unknown
RCAP_rcc02510	-0.556	0.018	-0.596	0.003	0.311	0.193	*lipoprotein*	Predicted Function	Unknown
RCAP_rcc02511	0.196	0.395	-1.037	0.000	-0.005	0.991	*hypothetical protein*	Unknown	Unknown
RCAP_rcc02512	-0.531	0.003	-0.914	0.000	0.408	0.094	*hypothetical protein*	Unknown	Unknown
RCAP_rcc02513	-0.197	0.337	0.257	0.182	0.069	0.827	*cscK*	Carbohydrate Metabolism	Amino sugar and nucleotide sugar metabolism
RCAP_rcc02514	-0.184	0.402	-0.054	0.795	0.043	0.899	*dtd*	Translation, ribosomal structure and biogenesis	Unknown
RCAP_rcc02515	-0.308	0.329	0.171	0.688	-0.093	0.864	*rhlE*	Replication, Recombination and Repair	Unknown
RCAP_rcc02516	-0.473	0.151	0.616	0.000	0.172	0.693	*tdk*	Xenobiotics Biodegradation and Metabolism	Drug metabolism - other enzymes
RCAP_rcc02517	-0.207	0.628	0.501	0.012	0.174	0.706	*trxC*	Post-translational Modification, Assembly and Chaperones	Unknown
RCAP_rcc02518	0.128	0.652	-0.235	0.330	0.009	0.983	*type 12 family methyltransferase*	Unknown	Unknown
RCAP_rcc02519	-0.596	0.012	0.647	0.003	0.381	0.200	*diguanylate cyclase/phosphodiesterase*	Signal Transduction	Kinase/Phosphorelay
RCAP_rcc02520	-0.805	0.004	-1.489	0.000	0.439	0.284	*integrin alpha repeat/hemolysin-type calcium-binding repeat family prote*	Trafficking and Secretion	Secretion
RCAP_rcc02521	0.440	0.047	0.281	0.049	-0.015	0.964	*pyrimidine ABC transporter periplasmic pyrimidine-binding protein*	Metal and Ion Transport	Unknown
RCAP_rcc02522	-0.044	0.844	0.098	0.544	0.157	0.405	*pyrimidine ABC transporter permease*	Metal and Ion Transport	Unknown
RCAP_rcc02523	0.196	0.282	-0.025	0.868	0.172	0.442	*pyrimidine ABC transporter permease*	Metal and Ion Transport	Unknown
RCAP_rcc02524	0.403	0.078	0.132	0.325	-0.080	0.791	*pyrimidine ABC transporter ATP-binding protein*	Metal and Ion Transport	Unknown
RCAP_rcc02525	0.470	0.048	0.320	0.420	0.040	0.903	*dht*	Metabolism of Cofactors, Coenzymes and Vitamins	Pantothenate and CoA biosynthesis
RCAP_rcc02526	0.193	0.478	0.669	NA	0.095	0.633	*amaB*	Xenobiotics Biodegradation and Metabolism	Drug metabolism - other enzymes
RCAP_rcc02527	0.111	0.617	-0.069	0.721	-0.146	0.601	*XRE family transcriptional regulator*	Signal Transduction	Transcription Regulator
RCAP_rcc02528	0.342	NA	-0.577	0.012	0.088	0.738	*dihydropyrimidine dehydrogenase*	Metabolism of Cofactors, Coenzymes and Vitamins	Pantothenate and CoA biosynthesis
RCAP_rcc02529	0.203	0.430	-0.406	0.205	0.165	0.431	*pyridine nucleotide-disulfide oxidoreductase*	Unknown	Unknown
RCAP_rcc02530	0.595	0.003	2.397	0.000	-0.367	0.233	*pucB*	Photosynthesis	Light Harvesting Machinery
RCAP_rcc02531	0.078	0.739	2.476	0.000	-0.205	0.506	*pucA*	Photosynthesis	Light Harvesting Machinery
RCAP_rcc02532	-0.001	0.995	1.909	0.000	-0.093	0.701	*pucC2*	Photosynthesis	Light Harvesting Machinery
RCAP_rcc02533	0.630	0.002	1.503	0.000	-0.216	0.568	*pucDE*	Photosynthesis	Light Harvesting Machinery
RCAP_rcc02534	-0.625	0.070	-0.203	0.529	0.149	0.727	*cysA*	Metal and Ion Transport	Sulfute
RCAP_rcc02535	-0.320	0.279	0.133	0.756	-0.064	0.869	*cysW*	Metal and Ion Transport	Sulfute
RCAP_rcc02536	-0.093	0.812	0.164	0.699	-0.033	0.953	*cysT*	Metal and Ion Transport	Sulfute
RCAP_rcc02537	-0.496	0.050	-0.785	0.012	0.130	0.558	*cysK1*	Energy Metabolism	Sulfur metabolism
RCAP_rcc02538	0.344	0.054	0.397	0.004	-0.012	0.978	*trpB2*	Amino Acid Metabolism	Phenylalanine, tyrosine and tryptophan biosynthesis
RCAP_rcc02539	-0.328	0.173	2.855	0.000	0.258	0.228	*diguanylate cyclase/phosphodiesterase*	Signal Transduction	Kinase/Phosphorelay
RCAP_rcc02540	-0.398	0.058	0.710	0.000	0.322	0.108	*diguanylate cyclase/phosphodiesterase*	Signal Transduction	Kinase/Phosphorelay
RCAP_rcc02541	0.192	0.493	-0.790	0.000	-0.144	0.738	*fruA*	Carbohydrate Metabolism	Fructose and mannose metabolism
RCAP_rcc02542	-0.085	NA	-0.440	0.024	0.075	NA	*fruK*	Carbohydrate Metabolism	Fructose and mannose metabolism
RCAP_rcc02543	-0.096	0.806	-0.081	0.814	-0.036	0.955	*fruB*	Carbohydrate Metabolism	Fructose and mannose metabolism
RCAP_rcc02544	-0.578	0.043	-0.065	0.801	0.290	0.466	*ABC transporter substrate-binding protein*	Carbohydrate Metabolism	Unknown
RCAP_rcc02545	-0.101	0.709	-0.352	0.030	0.179	0.642	*sensor histidine kinase/response regulator receiver protein*	Signal Transduction	Transcription Regulator
RCAP_rcc02546	-0.851	0.001	0.460	0.082	0.403	0.272	*winged helix family two component transcriptional regulator*	Signal Transduction	Transcription Regulator
RCAP_rcc02547	-0.473	0.059	0.327	0.341	0.066	0.869	*hypothetical protein*	Unknown	Unknown
RCAP_rcc02548	-0.434	0.194	0.554	0.000	0.260	0.327	*rng*	Unknown	Unknown
RCAP_rcc02549	-0.350	0.290	0.838	0.000	0.183	0.426	*maf*	Cell Division	Chromosome Partitioning
RCAP_rcc02550	0.603	0.007	0.510	0.000	-0.281	0.381	*infA*	Translation, ribosomal structure and biogenesis	Unknown
RCAP_rcc02551	-0.265	0.282	0.003	0.991	0.250	0.310	*carbon-nitrogen family hydrolase*	Predicted Function	Nitrogen metabolism
RCAP_rcc02552	-0.592	0.129	0.161	0.527	0.375	0.383	*GNAT family acetyltransferase*	Cell Division	Chromosome Partitioning
RCAP_rcc02553	0.826	0.001	0.038	0.837	-0.206	0.498	*hypothetical protein*	Unknown	Unknown
RCAP_rcc02554	-0.604	0.069	-1.072	0.000	0.087	0.874	*NmrA family protein*	Unknown	Unknown
RCAP_rcc02555	-0.452	0.097	0.280	0.171	0.324	0.275	*HxlR family transcriptional regulator*	Signal Transduction	Transcription Regulator
RCAP_rcc02556	0.261	0.229	0.236	0.256	-0.176	0.547	*arsC*	Signal Transduction	Kinase/Phosphorelay
RCAP_rcc02557	-0.370	0.178	-0.098	0.484	0.460	0.122	*uncharacterized protein family UPF0262*	Unknown	Unknown
RCAP_rcc02558	-0.702	0.020	-0.028	0.864	0.455	0.104	*hisD*	Amino Acid Metabolism	Histidine metabolism
RCAP_rcc02559	-0.530	0.068	-0.655	0.003	0.447	0.276	*GNAT family acetyltransferase*	Cell Division	Chromosome Partitioning
RCAP_rcc02560	-0.462	0.041	-0.446	0.000	0.245	0.446	*hypothetical protein*	Unknown	Unknown
RCAP_rcc02561	0.011	0.956	-0.082	0.474	-0.108	0.527	*murA*	Glycan Biosynthesis and Metabolism	Peptidoglycan biosynthesis
RCAP_rcc02562	0.379	0.363	-0.443	0.225	-0.532	0.194	*hypothetical protein*	Unknown	Unknown
RCAP_rcc02563	0.553	0.146	0.686	0.157	-0.522	0.204	*hypothetical protein*	Unknown	Unknown
RCAP_rcc02564	0.529	0.199	-0.121	0.854	-0.345	0.425	*hypothetical protein*	Unknown	Unknown
RCAP_rcc02565	1.331	0.000	-0.064	0.855	-0.968	0.005	*hypothetical protein*	Unknown	Unknown
RCAP_rcc02566	0.636	0.055	-0.195	0.362	-0.761	0.000	*reverse transcriptase catalytic domain-containing protein*	Unknown	Unknown
RCAP_rcc02567	0.559	0.037	0.065	0.758	-0.873	0.000	*resolvase*	Replication, Recombination and Repair	Unknown
RCAP_rcc02568	0.765	0.003	-0.214	0.283	-0.595	0.046	*hypothetical protein*	Unknown	Unknown
RCAP_rcc02569	-0.039	0.889	-0.290	0.132	-0.036	0.930	*hypothetical protein*	Unknown	Unknown
RCAP_rcc02570	0.287	0.503	0.552	0.321	-0.112	0.828	*hypothetical protein*	Unknown	Unknown
RCAP_rcc02571	0.326	0.195	0.231	0.452	-0.235	0.476	*hypothetical protein*	Unknown	Unknown
RCAP_rcc02572	0.158	0.581	0.075	0.779	-0.329	0.270	*hypothetical protein*	Unknown	Unknown
RCAP_rcc02573	0.309	0.486	-0.827	0.127	-0.010	0.987	*hypothetical protein*	Unknown	Unknown
RCAP_rcc02574	-0.238	0.594	0.003	0.994	0.405	0.326	*hypothetical protein*	Unknown	Unknown

RCAP_rcc02575	-0.041	NA	0.160	0.808	0.143	NA	*hypothetical protein*	Unknown	Unknown
RCAP_rcc02576	0.090	0.815	0.208	0.540	-0.243	0.512	*BRO family protein*	Transcription	Unknown
RCAP_rcc02577	-0.011	0.975	0.423	0.204	-0.208	0.430	*phage integrase*	Replication, Recombination and Repair	Phage Interaction
RCAP_rcc02578	0.362	0.172	0.527	0.084	-0.352	0.401	*iron(III) ABC transporter periplasmic iron(III)-compound-binding protei*	Metal, Ion, Cofactor Transport	Iron and Heme Transport
RCAP_rcc02579	-0.649	0.080	0.433	0.182	0.126	0.799	*Fe(III) ABC transporter permease*	Metal, Ion, Cofactor Transport	Iron and Heme Transport
RCAP_rcc02580	-0.040	0.918	-0.239	0.473	0.051	0.927	*metallo-beta-lactamase*	Unknown	Unknown
RCAP_rcc02581	-0.206	0.268	-0.418	0.014	0.091	0.736	*sensor histidine kinase*	Signal Transduction	Kinase/Phosphorelay
RCAP_rcc02582	-0.102	0.732	-0.248	0.479	0.223	0.526	*hypothetical protein*	Unknown	Unknown
RCAP_rcc02583	0.429	0.105	-0.110	0.484	-0.316	0.176	*lon*	Post-translational Modification, Assembly and Chaperones	Unknown
RCAP_rcc02584	0.183	0.566	-0.508	0.009	-0.173	0.581	*hup3*	Energy Metabolism	Aerobic/Anaerobic Respiration
RCAP_rcc02585	-0.105	0.511	-0.419	0.000	0.063	0.689	*ndh*	Energy Metabolism	Oxidative phosphorylation
RCAP_rcc02586	-0.763	0.015	-0.400	0.039	0.621	0.117	*hypothetical protein*	Unknown	Unknown
RCAP_rcc02587	0.662	0.001	-0.009	0.962	-0.237	0.526	*hypothetical protein*	Unknown	Unknown
RCAP_rcc02588	-0.681	0.020	0.004	0.992	0.467	0.214	*hypothetical protein*	Unknown	Unknown
RCAP_rcc02589	-0.383	0.315	-1.579	0.000	0.304	0.506	*fadJ*	Lipid Metabolism	Fatty acid metabolism
RCAP_rcc02590	0.841	0.000	-1.457	0.000	-0.100	0.750	*dksA2*	Signal Transduction	Kinase/Phosphorelay
RCAP_rcc02591	-0.352	0.098	3.419	0.000	0.359	0.122	*surface presentation of antigens protein family*	Motility	Flagellar Assembly
RCAP_rcc02592	-0.167	0.467	-0.204	0.298	0.182	0.354	*Na+/solute symporter / histidine kinase*	Signal Transduction	Kinase/Phosphorelay
RCAP_rcc02593	-0.658	0.108	-0.291	0.438	0.680	0.070	*hypothetical protein*	Unknown	Unknown
RCAP_rcc02594	-0.302	0.373	-0.391	0.150	0.061	0.902	*response regulator receiver protein*	Signal Transduction	Transcription Regulator
RCAP_rcc02595	-0.443	0.098	-0.259	0.241	0.391	0.114	*XRE family transcriptional regulator*	Signal Transduction	Transcription Regulator
RCAP_rcc02596	-1.290	0.000	-0.304	0.204	0.718	0.009	*lipoprotein*	Predicted Function	Unknown
RCAP_rcc02597	-0.282	0.233	1.735	0.000	0.198	0.225	*hypothetical protein*	Unknown	Unknown
RCAP_rcc02598	0.199	0.351	0.282	NA	0.109	0.598	*beta-alanine--pyruvate transaminase*	Carbohydrate Metabolism	Propanoate metabolism
RCAP_rcc02599	-0.319	0.207	-0.054	0.737	0.469	0.040	*TetR family transcriptional regulator*	Signal Transduction	Transcription Regulator
RCAP_rcc02600	-0.143	0.647	1.402	0.000	0.010	0.984	*hypothetical protein*	Unknown	Unknown
RCAP_rcc02601	0.675	0.000	0.181	0.440	-0.357	0.264	*accB*	Lipid Metabolism	Unknown
RCAP_rcc02602	0.174	0.392	0.137	0.484	-0.017	0.949	*accC*	Lipid Metabolism	Fatty acid biosynthesis
RCAP_rcc02603	-0.481	0.028	0.124	0.537	0.436	0.050	*hypothetical protein*	Unknown	Unknown
RCAP_rcc02604	-0.152	0.582	-0.154	NA	-0.068	0.791	*aat*	Unknown	Unknown
RCAP_rcc02605	0.055	0.839	-0.358	0.099	0.012	0.981	*hypothetical protein*	Unknown	Unknown
RCAP_rcc02606	0.126	0.635	-0.235	0.174	-0.124	0.698	*mammalian cell entry domain-containing protein*	Secondary metabolites biosynthesis, transport, and catabolism	Unknown
RCAP_rcc02607	0.334	0.074	-0.105	0.556	-0.229	0.442	*NDUFA12 family NADH ubiquinone oxidoreductase subunit*	Energy Metabolism	Unknown
RCAP_rcc02608	0.233	0.271	-0.075	0.508	-0.114	NA	*clpX*	Post-translational Modification, Assembly and Chaperones	Unknown
RCAP_rcc02609	0.551	0.014	-0.445	0.000	-0.179	0.628	*clpP*	Post-translational Modification, Assembly and Chaperones	Unknown
RCAP_rcc02610	-0.006	0.982	3.313	0.000	0.243	0.487	*hypothetical protein*	Unknown	Unknown
RCAP_rcc02611	-0.711	0.000	3.296	0.000	0.576	0.001	*mcpA3*	Motility	Chemotaxis
RCAP_rcc02612	0.590	0.007	-0.078	0.801	-0.013	0.981	*transthyretin family protein*	Unknown	Unknown
RCAP_rcc02613	0.032	0.891	-0.125	0.313	0.104	0.526	*chitin deacetylase*	Carbohydrate Metabolism	Unknown
RCAP_rcc02614	0.122	0.542	-0.122	0.381	0.094	0.712	*cupin domain-containing protein*	Unknown	Unknown
RCAP_rcc02615	0.199	0.510	-0.968	0.000	-0.074	0.839	*ttuD*	Carbohydrate Metabolism	Glyoxylate and dicarboxylate metabolism
RCAP_rcc02616	-0.274	0.293	-0.517	0.037	0.250	0.201	*hypothetical protein*	Unknown	Unknown
RCAP_rcc02617	0.629	0.006	-1.358	0.000	0.024	0.953	*ushA*	Metabolism of Cofactors, Coenzymes and Vitamins	Nicotinate and nicotinamide metabolism
RCAP_rcc02618	-0.230	0.374	0.186	0.569	0.032	0.930	*hypothetical protein*	Unknown	Unknown
RCAP_rcc02619	-0.192	0.283	-0.208	0.113	0.231	0.344	*pyrD2*	Nucleotide Metabolism	Unknown
RCAP_rcc02620	0.348	0.094	1.875	0.000	-0.058	0.834	*acsA2*	Carbohydrate Metabolism	Glycolysis / Gluconeogenesis
RCAP_rcc02621	0.075	0.826	-0.390	0.079	0.443	0.118	*sulP*	Metal and Ion Transport	Unknown
RCAP_rcc02622	-0.668	0.101	0.355	0.154	0.431	0.311	*pyridoxamine 5'-phosphate oxidase*	Unknown	Unknown
RCAP_rcc02623	-0.384	0.235	2.924	0.000	-0.184	0.601	*hypothetical protein*	Unknown	Unknown
RCAP_rcc02624	0.316	0.218	0.057	0.770	-0.221	0.480	*lysS*	Translation, ribosomal structure and biogenesis	Aminoacyl-tRNA biosynthesis
RCAP_rcc02625	0.202	0.529	0.158	0.553	-0.153	0.611	*hypothetical protein*	Unknown	Unknown
RCAP_rcc02626	0.021	0.961	0.485	0.011	-0.714	0.001	*hypothetical protein*	Unknown	Unknown
RCAP_rcc02627	-0.582	0.018	-0.104	0.615	0.078	0.851	*dacB*	Glycan Biosynthesis and Metabolism	Peptidoglycan biosynthesis
RCAP_rcc02628	-0.654	0.064	0.218	0.306	0.365	0.312	*nadD*	Metabolism of Cofactors, Coenzymes and Vitamins	Nicotinate and nicotinamide metabolism
RCAP_rcc02629	-0.588	0.035	2.262	0.000	0.331	0.300	*diguanylate cyclase/phosphodiesterase*	Signal Transduction	Kinase/Phosphorelay
RCAP_rcc02630	-0.917	0.000	2.232	0.000	0.486	0.047	*heme NO binding domain-containing protein*	Unknown	Unknown
RCAP_rcc02631	-0.395	0.114	-0.563	0.001	0.186	0.579	*hypothetical protein*	Unknown	Unknown
RCAP_rcc02632	-0.428	0.264	-0.347	0.269	0.166	0.683	*HAD superfamily hydrolase*	Carbohydrate Metabolism	Glyoxylate and dicarboxylate metabolism
RCAP_rcc02633	0.087	0.855	-0.985	0.007	-0.494	0.171	*hypothetical protein*	Unknown	Unknown
RCAP_rcc02634	0.231	0.349	0.393	0.010	-0.190	0.389	*response regulator receiver modulated diguanylate cyclase/phosphodieste*	Signal Transduction	Transcription Regulator
RCAP_rcc02635	-0.204	0.531	-0.687	0.000	-0.447	0.189	*hypothetical protein*	Unknown	Unknown
RCAP_rcc02636	0.131	0.759	-0.290	0.325	-0.551	0.144	*hypothetical protein*	Unknown	Unknown
RCAP_rcc02637	0.609	0.059	-0.585	0.019	-0.173	0.716	*ECF family RNA polymerase sigma factor*	Transcription	Unknown
RCAP_rcc02638	0.496	0.189	-1.122	0.000	0.065	0.894	*calcium-binding EF-hand domain-containing protein*	Unknown	Unknown
RCAP_rcc02639	-1.235	0.001	-0.180	0.496	0.639	0.095	*NUDIX superfamily hydrolase*	Unknown	Unknown
RCAP_rcc02640	-0.271	0.534	-0.630	0.056	0.480	NA	*lipocalin family protein*	Predicted Function	Unknown
RCAP_rcc02641	-0.317	0.471	-0.068	0.837	0.289	0.477	*lipoprotein*	Predicted Function	Unknown
RCAP_rcc02642	0.314	0.237	0.031	0.833	-0.009	0.974	*aldo/keto reductase family oxidoreductase*	Energy Metabolism	Unknown
RCAP_rcc02643	-0.018	0.958	-0.097	0.732	0.197	0.592	*class IV aminotransferase*	Metabolism of Cofactors, Coenzymes and Vitamins	Folate biosynthesis
RCAP_rcc02644	0.009	0.975	-0.056	0.813	-0.068	0.874	*pabB*	Metabolism of Cofactors, Coenzymes and Vitamins	Folate biosynthesis
RCAP_rcc02645	-0.279	0.345	-0.701	0.000	0.033	0.900	*frc*	Energy Metabolism	Unknown
RCAP_rcc02646	0.062	0.784	-0.961	0.002	0.424	0.177	*hypothetical protein*	Unknown	Unknown
RCAP_rcc02647	-0.192	0.519	0.450	0.024	0.106	0.794	*putA*	Amino Acid Metabolism	Arginine and proline metabolism
RCAP_rcc02648	0.226	0.398	-0.057	0.874	-0.223	0.492	*putR*	Transcription	Unknown
RCAP_rcc02649	-0.391	0.282	-0.083	0.687	0.386	0.232	*selD*	Metabolism of Other Amino Acids	Selenoamino acid metabolism
RCAP_rcc02650	-0.364	0.302	0.084	NA	0.279	0.356	*UspA domain-containing protein*	Stress Response	Unknown
RCAP_rcc02651	-0.723	0.042	-0.131	0.619	0.567	0.153	*hypothetical protein*	Unknown	Unknown

RCAP_rcc02652	0.330	0.124	-1.329	0.000	-0.031	0.945	*RbsD/FucU transport protein family*	Carbohydrate Metabolism	Unknown
RCAP_rcc02653	-0.234	0.454	-1.823	0.000	0.426	0.161	*glpK1*	Lipid Metabolism	Glycerolipid metabolism
RCAP_rcc02654	-0.012	0.968	-2.012	0.000	0.293	0.248	*tktB*	Carbohydrate Metabolism	Pentose phosphate pathway
RCAP_rcc02655	0.361	0.107	-1.924	0.000	0.053	0.908	*tktA*	Carbohydrate Metabolism	Pentose phosphate pathway
RCAP_rcc02656	0.663	0.009	-1.899	0.000	0.008	0.989	*hypothetical protein*	Unknown	Unknown
RCAP_rcc02657	0.689	0.008	-2.603	0.000	0.157	0.703	*monosacharide ABC transporter periplasmic monosacharide-binding pro*	Carbohydrate Metabolism	Aerobic/Anaerobic Respiration
RCAP_rcc02658	0.103	0.749	-2.139	0.000	0.493	0.057	*lipoprotein*	Predicted Function	Unknown
RCAP_rcc02659	0.043	0.857	-2.248	0.000	0.375	0.155	*monosacharide ABC transporter ATP-binding protein*	Carbohydrate Metabolism	Aerobic/Anaerobic Respiration
RCAP_rcc02660	0.450	0.044	-2.241	0.000	0.242	0.366	*monosacharide ABC transporter permease*	Carbohydrate Metabolism	Aerobic/Anaerobic Respiration
RCAP_rcc02661	0.303	0.132	-0.655	0.000	-0.037	0.930	*DeoR family transcriptional regulator*	Signal Transduction	Transcription Regulator
RCAP_rcc02662	-0.351	0.171	0.665	0.000	0.234	0.351	*class II aldolase/adducin N-terminal domain-containing protein/short-ch*	Unknown	Unknown
RCAP_rcc02663	0.272	0.331	0.418	0.046	-0.393	0.036	*DeoR family transcriptional regulator*	Signal Transduction	Transcription Regulator
RCAP_rcc02664	-0.170	0.581	0.803	0.000	0.157	0.526	*mtnA*	Amino Acid Metabolism	Cysteine and methionine metabolism
RCAP_rcc02665	0.553	0.040	-1.543	0.000	-0.205	0.362	*hemolysin-type calcium-binding repeat family protein*	Trafficking and Secretion	Secretion
RCAP_rcc02666	0.097	0.678	-0.870	0.000	0.122	0.641	*alpha/beta fold family hydrolase*	Unknown	Unknown
RCAP_rcc02667	-0.171	0.312	0.283	0.082	0.026	0.904	*fabI2*	Lipid Metabolism	Fatty acid biosynthesis
RCAP_rcc02668	0.277	0.230	0.256	0.141	-0.131	0.608	*fabB*	Lipid Metabolism	Biotin metabolism
RCAP_rcc02669	0.388	0.082	0.290	0.201	-0.126	0.600	*fabA*	Lipid Metabolism	Fatty acid biosynthesis
RCAP_rcc02670	0.149	0.698	-0.353	0.417	-0.453	0.114	*Fur family transcriptional regulator*	Signal Transduction	Transcription Regulator
RCAP_rcc02671	0.824	0.010	0.342	0.098	-0.284	0.454	*efp*	Translation, ribosomal structure and biogenesis	Unknown
RCAP_rcc02672	-0.072	0.831	0.148	0.330	0.120	0.729	*lysU*	Translation, ribosomal structure and biogenesis	Aminoacyl-tRNA biosynthesis
RCAP_rcc02673	0.088	0.724	-0.373	0.100	0.094	0.779	*hypothetical protein*	Unknown	Unknown
RCAP_rcc02674	0.055	0.812	-0.071	0.714	-0.047	0.894	*nylB*	Defense Mechanisms	Unknown
RCAP_rcc02675	0.398	0.118	2.928	0.000	-0.013	0.983	*MarR family transcriptional regulator*	Signal Transduction	Transcription Regulator
RCAP_rcc02676	0.572	0.008	-0.212	0.164	-0.327	0.268	*hypothetical protein*	Unknown	Unknown
RCAP_rcc02677	0.409	0.074	-0.002	0.993	-0.184	0.482	*aspC2*	Photosynthesis	Tropane, piperidine and pyridine alkaloid biosynthesis
RCAP_rcc02678	0.139	0.519	0.073	0.765	-0.135	0.577	*hypothetical protein*	Unknown	Unknown
RCAP_rcc02679	-0.074	0.836	-0.433	0.005	-0.064	0.871	*pyridine nucleotide-disulfide oxidoreductase*	Energy Metabolism	Unknown
RCAP_rcc02680	-0.358	0.206	-0.241	0.529	-0.079	0.839	*mdtK*	Defense Mechanisms	Unknown
RCAP_rcc02681	0.773	0.009	-0.516	0.012	-0.306	0.267	*cytochrome b561 family protein*	Energy Metabolism	Aerobic/Anaerobic Respiration
RCAP_rcc02682	0.673	0.003	-0.448	0.087	-0.027	0.951	*cytochrome c'*	Energy Metabolism	Aerobic/Anaerobic Respiration
RCAP_rcc02683	-0.064	0.835	-1.140	0.000	-0.092	0.819	*type 11 family methyltransferase*	Unknown	Unknown
RCAP_rcc02684	1.171	0.000	-1.932	0.000	-0.074	0.830	*polyphosphate kinase 2 domain-containing protein*	Energy Metabolism	Oxidative phosphorylation
RCAP_rcc02685	0.307	0.169	-0.526	0.001	-0.017	0.964	*glutathione S-transferase*	Sulfur Metabolism	Glutathione metabolism
RCAP_rcc02686	-0.313	0.246	0.227	0.136	0.328	0.079	*prfA*	Translation, ribosomal structure and biogenesis	Unknown
RCAP_rcc02687	-0.238	0.425	0.046	0.739	0.382	0.270	*hemK*	Metabolism of Cofactors, Coenzymes and Vitamins	Heme Biosynthesis
RCAP_rcc02688	0.074	0.828	0.762	0.000	-0.181	0.477	*hypothetical protein*	Unknown	Unknown
RCAP_rcc02689	-0.229	0.350	0.242	0.130	0.003	0.994	*ksgA*	Translation, ribosomal structure and biogenesis	Unknown
RCAP_rcc02690	-0.559	0.054	-0.183	0.230	0.513	0.171	*pdxA*	Metabolism of Cofactors, Coenzymes and Vitamins	Vitamin B6 metabolism
RCAP_rcc02691	-0.766	0.007	-0.172	0.261	0.661	0.041	*surA*	Post-translational Modification, Assembly and Chaperones	Unknown
RCAP_rcc02692	-0.350	0.095	-0.104	0.425	0.313	0.254	*organic solvent tolerance protein family*	Cell Envelope Biosynthesis	Cell Wall Biosynthesis
RCAP_rcc02693	-0.896	0.004	-0.509	0.000	0.718	0.050	*permease YjgP/YjgQ family protein*	Predicted Function	Unknown
RCAP_rcc02694	0.313	0.066	0.139	0.286	0.089	0.757	*permease YjgP/YjgQ family protein*	Predicted Function	Unknown
RCAP_rcc02695	-0.177	0.312	-0.346	0.048	0.203	0.305	*pepA2*	Metabolism of Other Amino Acids	Glutathione metabolism
RCAP_rcc02696	-0.566	0.032	-0.357	0.059	0.449	0.157	*holC*	Replication, Recombination and Repair	Replication
RCAP_rcc02697	0.331	0.098	0.191	0.265	-0.180	0.512	*thiB*	Metabolism of Cofactors, Coenzymes and Vitamins	Thiamine metabolism
RCAP_rcc02698	0.008	0.985	-0.145	0.630	-0.223	0.628	*thiP*	Metabolism of Cofactors, Coenzymes and Vitamins	Thiamine metabolism
RCAP_rcc02699	-0.223	0.570	-0.060	0.881	-0.395	0.358	*thiQ*	Metabolism of Cofactors, Coenzymes and Vitamins	Thiamine metabolism
RCAP_rcc02700	0.328	0.256	-0.516	0.139	-0.051	0.899	*lipoprotein*	Predicted Function	Unknown
RCAP_rcc02701	-0.026	0.940	-0.560	0.027	-0.087	0.775	*lipoprotein*	Predicted Function	Unknown
RCAP_rcc02702	0.372	0.092	1.870	0.000	-0.637	0.001	*cytochrome c/b561 family protein*	Energy Metabolism	Aerobic/Anaerobic Respiration
RCAP_rcc02703	-0.582	0.015	-0.282	0.302	0.257	0.516	*hypothetical protein*	Unknown	Unknown
RCAP_rcc02704	-0.662	0.027	-0.142	0.516	0.540	0.186	*NLP/P60 family protein*	Cell Envelope Biosynthesis	Cell Wall Biosynthesis
RCAP_rcc02705	-0.041	0.857	-0.126	0.570	0.118	0.613	*pepA3*	Metabolism of Other Amino Acids	Glutathione metabolism
RCAP_rcc02706	-0.331	0.265	1.568	0.000	0.348	0.316	*hypothetical protein*	Unknown	Unknown
RCAP_rcc02707	0.503	0.009	0.404	0.028	-0.356	0.244	*cynT*	Energy Metabolism	Nitrogen metabolism
RCAP_rcc02708	-0.223	0.336	1.123	0.000	0.208	0.385	*hypothetical protein*	Unknown	Unknown
RCAP_rcc02709	1.098	0.000	-0.159	0.615	-0.034	0.947	*hypothetical protein*	Unknown	Unknown
RCAP_rcc02710	0.762	0.000	-0.081	0.721	0.288	0.087	*hypothetical protein*	Unknown	Unknown
RCAP_rcc02711	0.645	0.010	0.257	0.241	-0.358	0.273	*asd*	Amino Acid Metabolism	Lysine biosynthesis
RCAP_rcc02712	-0.031	0.910	0.143	0.486	0.243	0.370	*major facilitator superfamily protein*	Carbohydrate Metabolism	Unknown
RCAP_rcc02713	0.355	0.269	-0.428	0.098	0.264	0.427	*LysE family transporter*	Amino Acid Metabolism	Unknown
RCAP_rcc02714	0.061	0.856	-0.701	0.003	-0.185	0.455	*hypothetical protein*	Unknown	Unknown
RCAP_rcc02715	0.382	0.137	-0.811	0.000	-0.203	0.526	*hypothetical protein*	Unknown	Unknown
RCAP_rcc02716	0.318	0.194	-0.490	0.013	-0.178	0.574	*hypothetical protein*	Unknown	Unknown
RCAP_rcc02717	0.193	0.520	-0.332	0.187	-0.174	0.608	*cytosine-specific DNA-methyltransferase*	Replication, Recombination and Repair	Unknown
RCAP_rcc02718	-0.289	0.420	0.012	0.981	0.039	0.949	*hypothetical protein*	Unknown	Unknown
RCAP_rcc02719	-0.076	0.868	-0.209	0.557	-0.252	0.582	*resolvase*	Replication, Recombination and Repair	Unknown
RCAP_rcc02720	-0.316	0.409	-0.639	0.140	-0.134	0.782	*hypothetical protein*	Unknown	Unknown
RCAP_rcc02721	0.119	NA	-0.464	0.451	-0.147	NA	*hypothetical protein*	Unknown	Unknown
RCAP_rcc02722	0.228	0.329	-0.610	0.000	-0.131	0.722	*hypothetical protein*	Unknown	Unknown
RCAP_rcc02723	-0.111	0.732	-0.490	0.055	-0.266	0.480	*hypothetical protein*	Unknown	Unknown
RCAP_rcc02724	-0.377	0.371	-0.902	0.006	-0.022	0.974	*ECF family RNA polymerase sigma factor*	Unknown	Unknown
RCAP_rcc02725	0.578	0.165	-0.720	0.063	-0.342	0.388	*hypothetical protein*	Unknown	Unknown
RCAP_rcc02726	-0.112	NA	-1.382	0.001	0.168	NA	*hypothetical protein*	Unknown	Unknown
RCAP_rcc02727	0.095	0.832	-1.044	0.000	-0.397	0.356	*hypothetical protein*	Unknown	Unknown
RCAP_rcc02728	0.622	0.088	-1.016	0.000	0.087	0.874	*ATPase AAA*	Unknown	Unknown

RCAP_rcc02729	0.147	0.717	-0.502	0.072	0.038	0.953	*hypothetical protein*	Unknown	Unknown
RCAP_rcc02730	0.407	0.339	-0.545	0.241	-0.161	0.734	*hypothetical protein*	Unknown	Unknown
RCAP_rcc02732	0.160	NA	-0.658	0.243	-0.004	NA	*hypothetical protein*	Unknown	Unknown
RCAP_rcc02733	0.741	0.066	-0.691	0.089	-0.168	0.726	*hypothetical protein*	Unknown	Unknown
RCAP_rcc02734	0.124	0.755	-0.599	0.079	-0.244	0.586	*recD*	Replication, Recombination and Repair	Unknown
RCAP_rcc02735	-0.123	0.723	0.013	0.964	-0.060	0.899	*virulence-associated protein E*	Unknown	Unknown
RCAP_rcc02736	-0.420	0.172	0.368	0.527	-0.577	0.032	*hypothetical protein*	Unknown	Unknown
RCAP_rcc02737	0.554	NA	-0.062	0.940	-0.049	NA	*hypothetical protein*	Unknown	Unknown
RCAP_rcc02738	0.120	0.810	0.555	0.370	-0.110	NA	*hypothetical protein*	Unknown	Unknown
RCAP_rcc02739	0.396	0.081	-0.039	0.888	-0.260	0.477	*hypothetical protein*	Unknown	Unknown
RCAP_rcc02740	0.186	0.692	0.879	0.123	-0.535	0.179	*ParB domain-containing protein nuclease*	Replication, Recombination and Repair	Unknown
RCAP_rcc02741	-0.289	0.520	1.147	0.025	-0.190	NA	*adenine-specific DNA-methyltransferase*	Unknown	Unknown
RCAP_rcc02742	-0.583	0.150	1.178	0.000	0.197	0.667	*cytosine-specific DNA-methyltransferase*	Replication, Recombination and Repair	Unknown
RCAP_rcc02743	0.446	0.260	0.961	0.027	-0.385	0.373	*hypothetical protein*	Unknown	Unknown
RCAP_rcc02744	0.052	0.894	0.683	0.003	-0.194	0.538	*cysP*	Metal and Ion Transport	Sulfute
RCAP_rcc02745	-0.445	0.279	-0.910	0.001	0.938	0.008	*RND family efflux transporter subunit MFP*	Cell Envelope Biosynthesis	Cell Wall Biosynthesis
RCAP_rcc02746	-0.660	0.081	-0.671	0.015	0.664	0.087	*RND family efflux transporter subunit MFP*	Cell Envelope Biosynthesis	Cell Wall Biosynthesis
RCAP_rcc02747	-0.188	0.309	-0.682	0.000	0.195	0.222	*acriflavin resistance protein family*	Metal and Ion Transport	Unknown
RCAP_rcc02748	-0.024	0.959	0.536	0.137	-0.687	0.068	*hypothetical protein*	Unknown	Unknown
RCAP_rcc02749	0.921	0.000	0.362	0.006	-0.343	0.367	*rpmG*	Translation, ribosomal structure and biogenesis	Unknown
RCAP_rcc02750	0.455	0.076	-0.770	0.000	-0.168	0.619	*hypothetical protein*	Unknown	Unknown
RCAP_rcc02751	-1.199	0.000	-0.148	0.369	0.671	0.036	*N-acetylmuramoyl-L-alanine amidase, family 2*	Cell Envelope Biosynthesis	Cell Wall Biosynthesis
RCAP_rcc02752	-0.168	0.406	-0.231	0.073	0.125	0.511	*lipoprotein*	Predicted Function	Unknown
RCAP_rcc02753	0.268	0.223	0.027	0.874	-0.144	0.610	*gatA*	Translation, ribosomal structure and biogenesis	Aminoacyl-tRNA biosynthesis
RCAP_rcc02754	0.518	0.024	0.298	0.082	-0.182	0.639	*gatC*	Translation, ribosomal structure and biogenesis	Aminoacyl-tRNA biosynthesis
RCAP_rcc02755	0.786	0.001	-0.579	0.013	-0.283	0.454	*metallo-beta-lactamase*	Unknown	Unknown
RCAP_rcc02756	0.182	0.384	-0.778	0.027	-0.566	0.034	*hypothetical protein*	Unknown	Unknown
RCAP_rcc02757	-0.079	0.669	0.205	0.137	0.202	0.168	*dnaX*	Replication, Recombination and Repair	Replication
RCAP_rcc02758	-0.026	0.915	-0.077	0.770	-0.346	0.121	*hypothetical protein*	Unknown	Unknown
RCAP_rcc02759	-0.558	0.039	-0.092	0.672	0.020	0.974	*recR*	Replication, Recombination and Repair	Recombination
RCAP_rcc02760	-0.443	0.091	0.199	0.583	-0.168	0.582	*TfoX N-terminal domain-containing protein*	Transcription	Unknown
RCAP_rcc02761	0.201	0.258	-0.639	0.000	-0.055	0.865	*glutathione S-transferase*	Sulfur Metabolism	Glutathione metabolism
RCAP_rcc02762	-0.373	0.124	-0.291	0.088	-0.160	0.582	*4Fe-4S ferredoxin, iron-sulfur cluster binding protein*	Energy Metabolism	Aerobic/Anaerobic Respiration
RCAP_rcc02763	-0.457	0.170	-0.739	0.068	-0.087	0.839	*hypothetical protein*	Unknown	Unknown
RCAP_rcc02764	-0.291	0.260	-3.452	0.000	0.470	0.029	*hypothetical protein*	Unknown	Unknown
RCAP_rcc02765	0.694	0.003	0.648	0.001	-0.302	0.211	*ilvE1*	Metabolism of Cofactors, Coenzymes and Vitamins	Pantothenate and CoA biosynthesis
RCAP_rcc02766	0.336	0.239	-0.256	0.158	-0.205	0.537	*petP*	Signal Transduction	Transcription Regulator
RCAP_rcc02767	-0.584	0.010	-0.031	0.893	0.436	0.106	*petR*	Signal Transduction	Transcription Regulator
RCAP_rcc02768	0.059	0.811	0.324	0.054	0.086	0.600	*petA*	Energy Metabolism	Aerobic/Anaerobic Respiration
RCAP_rcc02769	0.402	0.046	0.282	0.109	-0.177	0.605	*petB*	Energy Metabolism	Aerobic/Anaerobic Respiration
RCAP_rcc02770	0.001	0.997	0.261	0.120	0.141	0.374	*petC*	Energy Metabolism	Aerobic/Anaerobic Respiration
RCAP_rcc02771	0.050	0.877	-0.269	0.094	-0.056	0.851	*TetR family transcriptional regulator*	Signal Transduction	Transcription Regulator
RCAP_rcc02772	-0.725	0.002	-0.033	0.932	0.564	0.048	*hypothetical protein*	Unknown	Unknown
RCAP_rcc02773	0.123	0.676	0.116	0.641	-0.143	0.574	*hisP*	Amino Acid Metabolism	Amino Acid Transport
RCAP_rcc02774	0.416	0.032	-0.102	0.698	0.065	0.807	*hisJ*	Amino Acid Metabolism	Amino Acid Transport
RCAP_rcc02775	0.231	0.310	0.236	0.091	-0.033	0.908	*hisQ*	Amino Acid Metabolism	Amino Acid Transport
RCAP_rcc02776	0.334	0.134	0.026	0.883	-0.101	0.775	*hisM*	Amino Acid Metabolism	Amino Acid Transport
RCAP_rcc02777	0.598	0.002	-0.187	0.162	-0.082	0.779	*glnA4*	Carbohydrate Metabolism	Glyoxylate and dicarboxylate metabolism
RCAP_rcc02778	0.609	0.007	-0.430	0.000	-0.146	0.538	*class I glutamine amidotransferase*	Xenobiotics Biodegradation and Metabolism	Drug metabolism - other enzymes
RCAP_rcc02779	0.307	0.299	-0.568	0.010	-0.176	0.697	*hypothetical protein*	Unknown	Unknown
RCAP_rcc02780	0.374	0.044	-0.113	0.281	-0.014	0.975	*glnA5*	Carbohydrate Metabolism	Glyoxylate and dicarboxylate metabolism
RCAP_rcc02781	0.051	0.812	-0.117	0.420	0.367	0.083	*FAD dependent oxidoreductase*	Energy Metabolism	Unknown
RCAP_rcc02782	0.409	0.121	-0.059	0.824	-0.220	0.167	*DegT/DnrJ/EryC1/StrS family aminotransferase*	Amino Acid Metabolism	Unknown
RCAP_rcc02783	-0.162	0.626	-0.036	0.883	-0.361	0.414	*CoA-binding domain-containing protein*	Unknown	Unknown
RCAP_rcc02784	-0.731	0.022	0.094	0.560	0.584	0.093	*TrmH family RNA methyltransferase*	Translation, ribosomal structure and biogenesis	Unknown
RCAP_rcc02785	-0.096	0.733	0.083	0.735	-0.002	0.995	*hypothetical protein*	Unknown	Unknown
RCAP_rcc02786	0.161	0.546	0.356	0.182	-0.122	0.644	*hemolysin-type calcium-binding repeat family protein*	Trafficking and Secretion	Secretion
RCAP_rcc02787	0.427	0.020	-0.294	0.135	0.038	0.914	*metB*	Energy Metabolism	Sulfur metabolism
RCAP_rcc02788	-0.052	0.820	0.011	0.953	0.180	0.346	*hypothetical protein*	Unknown	Unknown
RCAP_rcc02789	0.860	0.000	0.611	0.001	-0.167	0.540	*kefC1*	Sulfur Metabolism	Glutathione metabolism
RCAP_rcc02790	0.597	0.003	-0.644	0.000	-0.271	0.381	*CarD family transcriptional regulator*	Signal Transduction	Transcription Regulator
RCAP_rcc02791	0.917	0.000	0.065	0.835	-0.386	0.270	*fdxA*	Energy Metabolism	Aerobic/Anaerobic Respiration
RCAP_rcc02792	-0.183	0.540	0.075	0.767	0.262	0.482	*hslR*	Stress Response	Unknown
RCAP_rcc02793	-0.091	0.603	0.071	0.601	0.018	0.948	*helicase domain-containing protein*	Unknown	Unknown
RCAP_rcc02794	0.030	0.910	-0.127	0.560	-0.074	0.768	*hypothetical protein*	Unknown	Unknown
RCAP_rcc02795	0.061	0.787	-0.059	0.794	0.167	0.350	*sterol-binding domain-containing protein*	Unknown	Unknown
RCAP_rcc02796	-0.751	0.011	-0.183	0.474	0.696	0.050	*pldB*	Lipid Metabolism	Glycerophospholipid metabolism
RCAP_rcc02797	0.130	0.577	0.691	0.000	-0.169	0.382	*T1 family peptidase*	Post-translational Modification, Assembly and Chaperones	Peptidase
RCAP_rcc02798	-0.321	0.177	-0.797	0.000	0.205	0.504	*transglutaminase domain-containing protein*	Amino Acid Metabolism	Unknown
RCAP_rcc02799	-0.103	0.753	-0.562	0.000	0.086	0.696	*hypothetical protein*	Unknown	Unknown
RCAP_rcc02800	0.165	0.605	-0.229	0.167	-0.302	0.214	*hypothetical protein*	Unknown	Unknown
RCAP_rcc02801	0.477	0.008	0.124	0.276	-0.160	0.512	*pepF*	Amino Acid Metabolism	Unknown
RCAP_rcc02802	-0.544	0.009	-0.023	0.913	0.152	0.699	*membrane dipeptidase*	Cell Envelope Biosynthesis	Cell Wall Biosynthesis
RCAP_rcc02803	-0.386	0.134	-0.083	0.714	-0.159	0.708	*hypothetical protein*	Unknown	Unknown
RCAP_rcc02804	0.246	0.272	0.627	0.003	0.038	0.942	*hypothetical protein*	Unknown	Unknown
RCAP_rcc02805	-0.281	0.327	-0.023	0.913	0.364	0.154	*hypothetical protein*	Unknown	Unknown
RCAP_rcc02806	-0.391	0.168	-0.042	0.834	0.316	0.358	*hypothetical protein*	Unknown	Unknown

RCAP_rcc02807	0.249	0.211	-0.160	0.516	-0.167	0.467	*lgt*	Glycan Biosynthesis and Metabolism	Glycosphingolipid biosynthesis - ganglio series
RCAP_rcc02808	0.821	0.000	0.279	0.100	-0.948	0.000	*hypothetical protein*	Unknown	Unknown
RCAP_rcc02809	0.267	0.192	0.110	0.524	-0.314	0.307	*hypothetical protein*	Unknown	Unknown
RCAP_rcc02810	-0.032	0.936	0.391	0.019	-0.079	0.887	*rluD*	Nucleotide Metabolism	Pyrimidine metabolism
RCAP_rcc02811	0.550	0.050	-0.032	0.834	-0.693	0.000	*rpoH2*	Replication, Recombination and Repair	Replication
RCAP_rcc02812	0.142	0.542	0.068	0.684	-0.452	0.139	*gfo*	Unknown	Unknown
RCAP_rcc02813	-0.320	0.112	0.553	0.000	-0.116	0.704	*lrp*	Signal Transduction	Transcription Regulator
RCAP_rcc02814	0.159	0.513	0.318	0.057	-0.079	0.808	*trxB*	Sulfur Metabolism	Selenoamino acid metabolism
RCAP_rcc02815	0.759	0.000	2.171	0.000	-0.245	0.531	*lipoprotein*	Predicted Function	Unknown
RCAP_rcc02816	0.307	0.303	-0.357	0.159	-0.285	0.251	*sat*	Energy Metabolism	Sulfur metabolism
RCAP_rcc02817	0.608	0.020	-0.187	0.539	-0.328	0.205	*hypothetical protein*	Unknown	Unknown
RCAP_rcc02818	0.721	0.012	-0.150	0.570	-0.578	0.000	*ibpA*	Stress Response	Unknown
RCAP_rcc02819	0.135	0.572	-0.374	0.032	-0.510	0.005	*hypothetical protein*	Unknown	Unknown
RCAP_rcc02820	0.190	0.341	-0.042	0.841	-0.085	0.668	*purE*	Nucleotide Metabolism	Purine metabolism
RCAP_rcc02821	-0.172	0.526	-0.220	0.081	0.177	0.322	*purK*	Nucleotide Metabolism	Purine metabolism
RCAP_rcc02822	-0.230	0.312	-0.170	0.492	0.130	0.712	*lipase*	Lipid Metabolism	Glycerolipid metabolism
RCAP_rcc02823	0.289	0.253	0.144	0.468	-0.091	0.728	*prs*	Carbohydrate Metabolism	Pentose phosphate pathway
RCAP_rcc02824	0.649	0.009	-0.417	0.009	-0.213	0.516	*DSBA family oxidoreductase*	Secondary metabolites biosynthesis, transport, and catabolism	Unknown
RCAP_rcc02825	-0.197	0.296	-0.149	0.308	0.236	0.225	*alpha/beta fold family hydrolase*	Unknown	Unknown
RCAP_rcc02826	0.482	0.038	-0.003	0.992	-0.123	0.722	*fumC*	Carbohydrate Metabolism	TCA Cycle
RCAP_rcc02827	-0.345	0.102	-0.442	0.009	0.331	0.168	*hypothetical protein*	Unknown	Unknown
RCAP_rcc02828	0.586	0.025	-0.113	0.414	-0.360	0.251	*hypothetical protein*	Unknown	Unknown
RCAP_rcc02829	-0.705	0.002	0.126	0.452	-0.165	0.684	*hypothetical protein*	Unknown	Unknown
RCAP_rcc02830	-0.230	0.267	0.205	0.132	-0.137	0.549	*acyltransferase domain-containing protein*	Unknown	Unknown
RCAP_rcc02831	-0.319	0.122	0.050	0.864	0.054	0.829	*ptsO*	Carbohydrate Metabolism	Unknown
RCAP_rcc02832	0.154	0.591	-0.104	0.659	-0.172	0.446	*PTS system, IIA component*	Carbohydrate Metabolism	Fructose and mannose metabolism
RCAP_rcc02833	0.288	0.252	-0.257	0.037	-0.408	0.198	*P-loop family ATPase*	Unknown	Unknown
RCAP_rcc02834	-0.455	0.189	-0.399	0.243	0.239	0.596	*hprK*	Signal Transduction	Kinase/Phosphorelay
RCAP_rcc02835	-0.196	0.351	0.019	0.932	-0.020	0.945	*chvG*	Signal Transduction	Kinase/Phosphorelay
RCAP_rcc02836	0.167	0.466	0.083	0.476	-0.195	0.531	*chvI*	Signal Transduction	Transcription Regulator
RCAP_rcc02837	0.381	0.042	-0.907	0.000	-0.238	0.378	*pckA*	Carbohydrate Metabolism	TCA Cycle
RCAP_rcc02838	-0.243	0.181	-0.089	0.615	0.030	0.914	*rnr*	Transcription	Unknown
RCAP_rcc02839	-0.068	0.892	-0.854	0.024	0.090	0.869	*mobA*	Metabolism of Cofactors, Coenzymes and Vitamins	Unknown
RCAP_rcc02840	-0.261	0.538	-2.926	0.000	0.514	0.125	*moaC2*	Metabolism of Cofactors, Coenzymes and Vitamins	Unknown
RCAP_rcc02841	0.256	0.528	-3.884	0.000	0.349	0.190	*moeB2*	Metabolism of Cofactors, Coenzymes and Vitamins	Unknown
RCAP_rcc02842	-0.342	NA	-3.842	0.000	0.525	0.198	*moaD1*	Metabolism of Cofactors, Coenzymes and Vitamins	Unknown
RCAP_rcc02843	0.683	0.053	-3.730	0.000	0.132	0.607	*moaA2*	Unknown	Unknown
RCAP_rcc02844	0.104	NA	-3.690	0.000	0.401	0.354	*hypothetical protein*	Unknown	Unknown
RCAP_rcc02845	1.583	0.000	-4.914	0.000	0.126	0.752	*torA*	Energy Metabolism	Methane metabolism
RCAP_rcc02846	0.349	NA	-4.478	0.000	0.393	0.350	*torD*	Unknown	Unknown
RCAP_rcc02847	1.520	0.000	-4.800	0.000	0.237	0.455	*torC*	Energy Metabolism	Aerobic/Anaerobic Respiration
RCAP_rcc02848	-0.259	0.212	-0.772	0.000	0.109	0.678	*torR*	Signal Transduction	Transcription Regulator
RCAP_rcc02849	2.185	0.000	-1.448	0.000	0.315	0.487	*dorS*	Energy Metabolism	Aerobic/Anaerobic Respiration
RCAP_rcc02850	0.382	0.118	0.372	0.222	-0.056	0.874	*thiM*	Metabolism of Cofactors, Coenzymes and Vitamins	Thiamine metabolism
RCAP_rcc02851	0.261	NA	-0.575	0.384	-0.255	NA	*thiE*	Metabolism of Cofactors, Coenzymes and Vitamins	Thiamine metabolism
RCAP_rcc02852	-0.563	0.178	-0.223	0.627	0.105	NA	*thiD*	Metabolism of Cofactors, Coenzymes and Vitamins	Thiamine metabolism
RCAP_rcc02853	0.035	0.942	0.359	0.335	0.135	0.783	*ABC transporter periplasmic substrate-binding protein*	Metal and Ion Transport	Unknown
RCAP_rcc02854	0.276	0.532	0.859	0.066	-0.033	0.960	*ABC transporter ATP-binding protein*	Metal and Ion Transport	Unknown
RCAP_rcc02855	-0.365	0.395	1.246	0.002	0.071	0.899	*ABC transporter permease*	Metal and Ion Transport	Unknown
RCAP_rcc02856	-0.478	0.149	5.001	0.000	0.523	0.065	*PAS/PAC sensor domain-containing protein*	Motility	Aerotaxis
RCAP_rcc02857	-0.722	0.003	3.626	0.000	0.654	0.000	*diguanylate cyclase/phosphodiesterase*	Signal Transduction	Kinase/Phosphorelay
RCAP_rcc02858	-0.523	0.081	-0.373	0.146	0.336	0.326	*hypothetical protein*	Unknown	Unknown
RCAP_rcc02859	0.604	0.013	1.745	0.002	0.118	0.613	*acsA3*	Carbohydrate Metabolism	Glycolysis / Gluconeogenesis
RCAP_rcc02860	0.988	0.001	2.007	0.001	-0.120	0.708	*hypothetical protein*	Unknown	Unknown
RCAP_rcc02861	0.798	0.002	1.719	0.002	0.034	0.922	*actP3*	Unknown	Unknown
RCAP_rcc02862	0.120	0.720	1.621	0.000	0.442	0.200	*cyclic nucleotide-binding domain-/cystathionine beta-synthase domain-/u*	Signal Transduction	Kinase/Phosphorelay
RCAP_rcc02863	-0.155	0.687	0.872	0.087	0.399	0.356	*response regulator receiver protein*	Signal Transduction	Transcription Regulator
RCAP_rcc02864	-0.317	0.257	-1.088	0.000	0.407	NA	*PAS domain/exonuclease domain-containing protein*	Replication, Recombination and Repair	Replication
RCAP_rcc02865	-0.458	0.074	-2.227	0.000	0.473	0.009	*pflA2*	Post-translational Modification, Assembly and Chaperones	Unknown
RCAP_rcc02866	0.379	0.184	-1.955	0.000	0.102	0.752	*pflB*	Carbohydrate Metabolism	Butanoate metabolism
RCAP_rcc02867	0.093	0.812	0.172	0.750	0.166	0.730	*hypothetical protein*	Unknown	Unknown
RCAP_rcc02868	-0.942	0.000	-0.343	0.143	0.478	0.231	*S1/S6 family peptidase*	Post-translational Modification, Assembly and Chaperones	Peptidase
RCAP_rcc02869	-0.377	0.077	-1.540	0.000	0.337	0.288	*glcF*	Carbohydrate Metabolism	Glyoxylate and dicarboxylate metabolism
RCAP_rcc02870	0.060	0.794	-1.874	0.000	0.106	0.782	*glcE*	Carbohydrate Metabolism	Glyoxylate and dicarboxylate metabolism
RCAP_rcc02871	0.367	0.035	-1.372	0.000	-0.063	0.827	*glcD*	Carbohydrate Metabolism	Glyoxylate and dicarboxylate metabolism
RCAP_rcc02872	-0.251	0.394	-0.464	0.013	0.207	0.391	*hypothetical protein*	Unknown	Unknown
RCAP_rcc02873	0.260	0.360	-0.100	0.624	-0.223	0.471	*kpsT2*	Carbohydrate Metabolism	Unknown
RCAP_rcc02874	0.004	0.990	-0.847	0.000	0.009	0.979	*kpsE2*	Cell Envelope Biosynthesis	Cell Wall Biosynthesis
RCAP_rcc02875	-0.112	0.527	-0.089	0.495	0.162	0.334	*kdsA*	Glycan Biosynthesis and Metabolism	Lipopolysaccharide biosynthesis
RCAP_rcc02876	0.299	0.070	0.456	0.004	0.019	0.960	*DeoC/LacD family aldolase*	Carbohydrate Metabolism	Unknown
RCAP_rcc02877	0.756	0.000	0.102	0.567	-0.014	NA	*monosacharide ABC transporter periplasmic monosacharide-binding pro*	Carbohydrate Metabolism	Aerobic/Anaerobic Respiration
RCAP_rcc02878	0.377	0.118	0.076	0.758	-0.045	0.901	*monosacharide ABC transporter permease*	Carbohydrate Metabolism	Aerobic/Anaerobic Respiration
RCAP_rcc02879	0.311	0.344	0.137	0.507	0.136	0.697	*monosacharide ABC transporter permease*	Carbohydrate Metabolism	Aerobic/Anaerobic Respiration
RCAP_rcc02880	0.120	0.694	0.105	0.698	0.016	0.969	*monosacharide ABC transporter ATP-binding protein*	Carbohydrate Metabolism	Aerobic/Anaerobic Respiration
RCAP_rcc02881	0.373	0.394	-0.709	0.129	-0.002	NA	*rpe2*	Energy Metabolism	Carbon fixation in photosynthetic organisms
RCAP_rcc02882	0.809	0.002	0.277	0.399	-0.397	0.288	*antibiotic biosynthesis monooxygenase*	Unknown	Unknown
RCAP_rcc02883	0.588	0.014	0.421	0.014	-0.057	0.902	*DeoR family transcriptional regulator*	Signal Transduction	Transcription Regulator

RCAP_rcc02884	-0.088	0.732	-0.180	0.286	0.267	0.251	*FGGY family carbohydrate kinase*	Carbohydrate Metabolism	Unknown
RCAP_rcc02885	-0.061	0.806	0.647	0.000	0.045	0.899	*dimethylglycine dehydrogenase*	Amino Acid Metabolism	Unknown
RCAP_rcc02886	-0.489	0.209	-0.021	0.956	0.379	0.327	*XRE family transcriptional regulator*	Signal Transduction	Transcription Regulator
RCAP_rcc02887	-0.598	0.044	3.022	0.000	0.555	0.001	*methyl-accepting chemotaxis sensory transducer*	Motility	Chemotaxis
RCAP_rcc02888	-0.743	0.020	1.248	0.001	-0.145	0.767	*hypothetical protein*	Unknown	Unknown
RCAP_rcc02889	0.348	0.148	-0.286	0.060	0.006	0.988	*hypothetical protein*	Unknown	Unknown
RCAP_rcc02890	-1.590	0.000	-0.394	0.417	0.166	NA	*hypothetical protein*	Unknown	Unknown
RCAP_rcc02891	-0.837	NA	-0.412	0.539	0.163	NA	*hypothetical protein*	Unknown	Unknown
RCAP_rcc02892	-1.518	0.000	-0.668	NA	0.141	NA	*hypothetical protein*	Unknown	Unknown
RCAP_rcc02893	-0.733	0.002	-0.523	0.479	0.159	0.743	*cspA3*	Transcription	Unknown
RCAP_rcc02894	-0.433	0.303	0.394	0.559	0.054	NA	*mdtE*	Cell Envelope Biosynthesis	Cell Wall Biosynthesis
RCAP_rcc02895	-0.256	0.189	0.690	0.004	0.009	0.986	*mdtF*	Unknown	Unknown
RCAP_rcc02896	-0.716	0.016	-0.110	0.744	0.343	0.432	*baeR*	Signal Transduction	Transcription Regulator
RCAP_rcc02897	-0.193	0.478	-0.345	0.350	-0.212	0.639	*sensor histidine kinase*	Signal Transduction	Kinase/Phosphorelay
RCAP_rcc02898	-0.595	0.010	-0.381	0.034	0.254	0.529	*ABC transporter ATP-binding/permease*	Unknown	Unknown
RCAP_rcc02899	-0.421	0.229	1.017	0.008	-0.058	0.923	*hypothetical protein*	Unknown	Unknown
RCAP_rcc02900	0.512	0.125	-0.036	0.932	0.040	0.951	*IS3 family transposase*	Replication, Recombination and Repair	Recombination
RCAP_rcc02903	0.068	0.885	0.050	0.921	-0.108	0.833	*hypothetical protein*	Unknown	Unknown
RCAP_rcc02904	-0.003	NA	-0.644	0.369	-0.086	NA	*hypothetical protein*	Unknown	Unknown
RCAP_rcc02905	0.152	NA	-0.321	0.576	0.169	NA	*hypothetical protein*	Unknown	Unknown
RCAP_rcc02906	-0.468	0.273	-0.202	0.583	0.164	0.734	*hypothetical protein*	Unknown	Unknown
RCAP_rcc02907	-0.011	0.977	0.263	0.305	-0.383	0.293	*hypothetical protein*	Unknown	Unknown
RCAP_rcc02908	0.033	NA	-0.804	0.251	-0.624	0.105	*hypothetical protein*	Unknown	Unknown
RCAP_rcc02909	-0.039	0.941	0.288	0.597	-0.246	0.576	*hypothetical protein*	Unknown	Unknown
RCAP_rcc02910	-0.002	NA	-0.283	0.681	0.089	NA	*hypothetical protein*	Unknown	Unknown
RCAP_rcc02911	0.072	0.843	0.156	0.612	-0.392	0.325	*hypothetical protein*	Unknown	Unknown
RCAP_rcc02912	0.055	NA	0.621	0.371	0.079	NA	*hypothetical protein*	Unknown	Unknown
RCAP_rcc02913	0.327	0.463	-0.311	0.528	0.086	NA	*hypothetical protein*	Unknown	Unknown
RCAP_rcc02914	-0.220	0.597	-0.151	0.675	-0.110	0.826	*hypothetical protein*	Unknown	Unknown
RCAP_rcc02915	-0.148	NA	-0.159	0.838	-0.007	NA	*hypothetical protein*	Unknown	Unknown
RCAP_rcc02916	0.103	NA	-0.749	0.287	-0.006	NA	*hypothetical protein*	Unknown	Unknown
RCAP_rcc02917	-0.059	NA	-0.441	0.470	-0.029	NA	*hypothetical protein*	Unknown	Unknown
RCAP_rcc02918	0.228	NA	-0.303	0.670	-0.208	NA	*hypothetical protein*	Unknown	Unknown
RCAP_rcc02919	0.213	0.598	0.125	0.790	-0.400	0.295	*hypothetical protein*	Unknown	Unknown
RCAP_rcc02920	-0.109	NA	-0.400	0.478	0.259	NA	*hypothetical protein*	Unknown	Unknown
RCAP_rcc02921	0.679	0.032	0.112	0.823	-0.508	0.200	*hypothetical protein*	Unknown	Unknown
RCAP_rcc02922	0.383	0.213	-0.030	0.938	-0.483	0.157	*phage virion morphogenesis protein*	Replication, Recombination and Repair	Phage Interaction
RCAP_rcc02923	0.970	0.001	-0.001	0.997	-0.979	0.000	*hypothetical protein*	Unknown	Unknown
RCAP_rcc02924	0.934	0.017	0.535	0.385	-0.191	0.692	*hypothetical protein*	Unknown	Unknown
RCAP_rcc02925	0.345	0.428	0.390	0.570	0.126	NA	*hypothetical protein*	Unknown	Unknown
RCAP_rcc02926	0.468	0.254	0.623	0.245	-0.352	0.428	*hypothetical protein*	Unknown	Unknown
RCAP_rcc02927	-0.259	0.563	0.216	0.731	-0.213	0.644	*hypothetical protein*	Unknown	Unknown
RCAP_rcc02928	0.586	0.007	-0.071	0.731	-0.247	0.526	*XRE family transcriptional regulator*	Signal Transduction	Transcription Regulator
RCAP_rcc02929	0.429	0.181	-1.122	0.000	-0.167	0.626	*hypothetical protein*	Unknown	Unknown
RCAP_rcc02930	0.511	0.091	-0.948	0.000	-0.252	0.526	*hypothetical protein*	Unknown	Unknown
RCAP_rcc02931	0.326	0.447	0.298	0.553	-0.075	0.890	*hypothetical protein*	Unknown	Unknown
RCAP_rcc02932	0.760	0.038	0.395	0.387	-0.846	0.005	*pinE*	Replication, Recombination and Repair	Unknown
RCAP_rcc02933	0.522	0.044	0.043	0.842	-0.552	0.021	*hypothetical protein*	Unknown	Unknown
RCAP_rcc02934	0.251	0.379	0.337	0.205	-0.381	0.235	*phage integrase*	Replication, Recombination and Repair	Phage Interaction
RCAP_rcc02935	0.102	0.792	0.334	0.387	-0.190	0.655	*hypothetical protein*	Unknown	Unknown
RCAP_rcc02936	0.221	0.524	0.167	0.648	-0.225	0.512	*hypothetical protein*	Unknown	Unknown
RCAP_rcc02937	0.029	0.944	-0.045	0.913	-0.124	0.742	*hypothetical protein*	Unknown	Unknown
RCAP_rcc02938	-0.287	0.379	0.123	0.654	-0.147	0.591	*hypothetical protein*	Unknown	Unknown
RCAP_rcc02939	0.639	0.016	-0.042	0.812	-0.176	0.651	*hypothetical protein*	Unknown	Unknown
RCAP_rcc02940	-0.786	0.042	-0.331	0.466	0.000	0.999	*hypothetical protein*	Unknown	Unknown
RCAP_rcc02941	-0.053	NA	-0.036	0.964	-0.001	NA	*hypothetical protein*	Unknown	Unknown
RCAP_rcc02942	-0.149	NA	-0.144	0.833	-0.022	NA	*hypothetical protein*	Unknown	Unknown
RCAP_rcc02943	-0.006	NA	-0.351	0.505	-0.048	NA	*hypothetical protein*	Unknown	Unknown
RCAP_rcc02944	0.328	NA	0.067	0.922	-0.147	0.752	*hypothetical protein*	Unknown	Unknown
RCAP_rcc02945	-0.235	NA	-0.070	0.886	0.015	0.981	*hypothetical protein*	Unknown	Unknown
RCAP_rcc02946	0.028	0.958	-0.241	0.690	0.075	NA	*hypothetical protein*	Unknown	Unknown
RCAP_rcc02947	-0.196	0.529	-1.300	0.000	0.131	0.696	*hypothetical protein*	Unknown	Unknown
RCAP_rcc02948	-0.166	0.724	-1.245	0.000	0.469	0.266	*hypothetical protein*	Unknown	Unknown
RCAP_rcc02949	-0.101	0.754	-1.326	0.000	-0.255	0.312	*hypothetical protein*	Unknown	Unknown
RCAP_rcc02950	0.027	0.945	-0.554	0.061	-0.213	0.579	*hypothetical protein*	Unknown	Unknown
RCAP_rcc02951	0.171	0.667	-0.535	0.184	-0.221	0.626	*excisionase, DNA-binding domain-containing protein*	Unknown	Unknown
RCAP_rcc02952	0.272	0.317	-0.516	0.016	-0.145	0.661	*hypothetical protein*	Unknown	Unknown
RCAP_rcc02953	0.229	0.442	-0.081	0.648	-0.453	0.012	*phage integrase*	Replication, Recombination and Repair	Phage Interaction
RCAP_rcc02954	0.047	0.838	-0.155	0.205	0.115	0.520	*mechanosensitive ion channel family protein*	Unknown	Unknown
RCAP_rcc02955	-0.108	0.697	0.139	0.466	0.165	0.390	*cysK2*	Energy Metabolism	Sulfur metabolism
RCAP_rcc02956	-0.547	0.007	-0.218	0.144	-0.045	0.894	*nudF*	Nucleotide Metabolism	Purine metabolism
RCAP_rcc02957	-0.145	0.564	-0.305	0.112	-0.309	0.072	*hypothetical protein*	Unknown	Unknown
RCAP_rcc02958	-0.149	0.602	0.708	0.000	-0.053	0.927	*phrB*	Replication, Recombination and Repair	Unknown
RCAP_rcc02959	0.810	0.001	0.014	0.963	-0.059	NA	*potD5*	Amino Acid Metabolism	Amino Acid Transport
RCAP_rcc02960	0.359	0.099	-0.143	0.442	-0.162	0.621	*neuB*	Carbohydrate Metabolism	Amino sugar and nucleotide sugar metabolism
RCAP_rcc02961	-0.491	0.003	-0.212	0.225	0.039	0.886	*family 25 glycosyl transferase*	Cell Envelope Biosynthesis	Cell Wall Biosynthesis
RCAP_rcc02962	0.059	0.771	-0.059	0.661	-0.042	0.888	*rhamnan synthesis protein F family*	Cell Envelope Biosynthesis	Cell Wall Biosynthesis

RCAP_rcc02963	0.131	0.648	0.446	0.000	-0.110	0.697	*hypothetical protein*	Unknown	Unknown
RCAP_rcc02964	0.056	0.861	0.697	0.000	-0.096	0.728	*webC*	Carbohydrate Metabolism	Amino sugar and nucleotide sugar metabolism
RCAP_rcc02965	-0.369	0.047	-0.283	0.029	0.354	0.136	*oxidoreductase family NAD-binding Rossmann fold protein*	Unknown	Unknown
RCAP_rcc02966	0.312	0.082	-0.384	0.020	-0.055	0.809	*short-chain dehydrogenase/reductase family oxidoreductase*	Metabolism of Cofactors, Coenzymes and Vitamins	Biotin metabolism
RCAP_rcc02967	0.154	0.385	-0.061	0.695	0.033	0.900	*cytidylyltransferase*	Carbohydrate Metabolism	Amino sugar and nucleotide sugar metabolism
RCAP_rcc02968	-0.453	0.105	0.034	0.847	0.149	0.664	*hypothetical protein*	Unknown	Unknown
RCAP_rcc02969	-0.271	0.346	0.351	0.320	-0.297	0.264	*hypothetical protein*	Unknown	Unknown
RCAP_rcc02970	-0.642	0.009	-0.310	0.090	0.433	0.046	*atpC*	Energy Metabolism	Methane metabolism
RCAP_rcc02971	0.231	0.207	-0.299	0.085	-0.059	0.844	*atpD*	Energy Metabolism	Methane metabolism
RCAP_rcc02972	0.247	0.200	-0.308	0.032	-0.102	0.714	*atpG*	Energy Metabolism	Methane metabolism
RCAP_rcc02973	0.355	0.052	-0.252	0.091	-0.132	0.651	*atpA*	Energy Metabolism	Methane metabolism
RCAP_rcc02974	0.555	0.024	-0.121	0.659	-0.081	0.799	*atpH*	Energy Metabolism	Methane metabolism
RCAP_rcc02975	-0.377	0.111	-0.338	0.012	0.200	0.526	*hypothetical protein*	Unknown	Unknown
RCAP_rcc02976	-0.199	0.284	-0.231	0.279	0.033	0.919	*gloB*	Sulfur Metabolism	Glutathione metabolism
RCAP_rcc02977	-0.009	0.969	-0.177	0.101	0.106	0.699	*clpA*	Post-translational Modification, Assembly and Chaperones	Unknown
RCAP_rcc02978	0.269	0.449	-0.661	0.011	0.038	0.949	*M23 family peptidase*	Post-translational Modification, Assembly and Chaperones	Peptidase
RCAP_rcc02979	0.135	0.670	0.501	0.009	-0.080	0.811	*rpsD*	Translation, ribosomal structure and biogenesis	Unknown
RCAP_rcc02980	-0.421	0.247	2.199	0.000	0.418	0.134	*hypothetical protein*	Unknown	Unknown
RCAP_rcc02981	0.025	0.926	0.089	0.488	0.133	0.444	*hisC1*	Amino Acid Metabolism	Histidine metabolism
RCAP_rcc02982	0.124	0.512	0.061	0.690	0.006	0.984	*tyrA*	Amino Acid Metabolism	Phenylalanine, tyrosine and tryptophan biosynthesis
RCAP_rcc02983	-1.221	0.000	-0.291	0.045	0.595	0.118	*extensin family protein*	Unknown	Unknown
RCAP_rcc02984	-0.437	0.282	-0.384	0.037	0.507	0.198	*hypothetical protein*	Unknown	Unknown
RCAP_rcc02985	-0.391	0.311	0.313	0.117	0.194	0.664	*chaC*	Metal and Ion Transport	Unknown
RCAP_rcc02986	0.193	0.514	-0.645	0.000	0.007	0.984	*motA/TolQ/ExbB proton channel family protein*	Motility	Chemotaxis
RCAP_rcc02987	-0.267	0.280	-0.641	0.000	0.369	0.079	*OmpA/MotB domain-containing protein*	Motility	Chemotaxis
RCAP_rcc02988	-0.504	0.050	0.169	0.300	0.327	0.286	*hypothetical protein*	Unknown	Unknown
RCAP_rcc02989	-0.370	0.207	0.160	0.429	0.267	0.559	*hypothetical protein*	Unknown	Unknown
RCAP_rcc02990	-0.510	0.164	0.329	0.057	0.206	0.639	*YbaK/EbsC family protein*	Unknown	Unknown
RCAP_rcc02991	-0.700	0.024	-0.687	0.041	0.330	0.292	*hadH*	Unknown	Unknown
RCAP_rcc02992	-0.558	0.065	-0.877	0.003	0.333	0.232	*atoB1*	Carbohydrate Metabolism	Glyoxylate and dicarboxylate metabolism
RCAP_rcc02993	0.197	0.253	0.027	0.870	-0.123	0.605	*ilvD*	Metabolism of Cofactors, Coenzymes and Vitamins	Pantothenate and CoA biosynthesis
RCAP_rcc02994	0.103	0.709	0.347	0.030	-0.112	0.813	*exoD*	Unknown	Unknown
RCAP_rcc02995	-0.110	0.784	-0.536	0.044	0.227	0.626	*hypothetical protein*	Unknown	Unknown
RCAP_rcc02996	-0.204	NA	-0.510	0.249	0.242	NA	*hypothetical protein*	Unknown	Unknown
RCAP_rcc02997	-0.002	0.995	-0.622	0.000	-0.110	0.704	*phospholipid/glycerol acyltransferase*	Lipid Metabolism	Glycerophospholipid metabolism
RCAP_rcc02998	-0.087	0.736	-0.400	0.012	-0.228	0.229	*hypothetical protein*	Unknown	Unknown
RCAP_rcc02999	0.330	0.176	-0.134	0.547	-0.261	0.482	*hypothetical protein*	Unknown	Unknown
RCAP_rcc03000	0.267	0.235	1.821	0.000	-0.017	0.974	*hypothetical protein*	Unknown	Unknown
RCAP_rcc03001	0.411	0.032	0.004	0.987	-0.135	0.624	*proA*	Amino Acid Metabolism	Arginine and proline metabolism
RCAP_rcc03002	0.241	0.421	-0.125	NA	-0.151	0.649	*hypothetical protein*	Unknown	Unknown
RCAP_rcc03003	0.150	0.571	0.299	0.414	-0.264	0.519	*hypothetical protein*	Unknown	Unknown
RCAP_rcc03004	-0.125	0.697	-0.918	0.000	0.106	0.701	*DeoR family transcriptional regulator*	Signal Transduction	Transcription Regulator
RCAP_rcc03005	-0.560	0.018	-0.486	0.000	0.260	0.478	*diguanylate cyclase/phosphodiesterase*	Signal Transduction	Kinase/Phosphorelay
RCAP_rcc03006	-0.171	0.721	-0.527	0.298	-0.086	NA	*apbE*	Metabolism of Cofactors, Coenzymes and Vitamins	Thiamine metabolism
RCAP_rcc03007	0.012	0.976	-0.376	0.204	-0.010	0.989	*cysJ*	Energy Metabolism	Unknown
RCAP_rcc03008	0.844	0.001	-0.184	0.579	-0.328	0.442	*hypothetical protein*	Unknown	Unknown
RCAP_rcc03009	1.140	0.000	-0.249	0.678	-0.213	NA	*hypothetical protein*	Unknown	Unknown
RCAP_rcc03010	-0.194	0.579	-0.718	0.000	0.218	0.632	*hypothetical protein*	Unknown	Unknown
RCAP_rcc03011	0.217	0.525	-0.143	0.591	-0.138	0.725	*winged helix family two component transcriptional regulator*	Signal Transduction	Transcription Regulator
RCAP_rcc03012	-0.179	0.484	0.380	0.117	0.003	0.991	*signal transduction histidine kinase*	Signal Transduction	Kinase/Phosphorelay
RCAP_rcc03013	0.307	0.093	1.852	0.000	-0.132	0.553	*DeoC/LacD family aldolase*	Carbohydrate Metabolism	Unknown
RCAP_rcc03014	-0.021	0.949	4.309	0.000	0.238	0.477	*methyl-accepting chemotaxis sensory transducer*	Motility	Chemotaxis
RCAP_rcc03015	-0.723	0.002	1.252	0.000	0.364	0.171	*kefC2*	Sulfur Metabolism	Glutathione metabolism
RCAP_rcc03016	-0.095	0.763	1.306	0.000	-0.340	0.122	*draT*	Energy Metabolism	Nitrogen metabolism
RCAP_rcc03017	-0.466	0.094	-0.287	0.279	0.412	0.270	*draG*	Energy Metabolism	Nitrogen metabolism
RCAP_rcc03018	-0.608	0.139	0.508	0.129	0.300	0.451	*hypothetical protein*	Unknown	Unknown
RCAP_rcc03022	0.352	0.422	0.465	0.191	-0.396	0.199	*dctM3*	Carbohydrate Metabolism	Unknown
RCAP_rcc03023	0.114	0.817	0.547	0.129	-0.356	0.247	*dctQ3*	Carbohydrate Metabolism	Unknown
RCAP_rcc03024	0.568	0.169	0.893	0.004	-0.653	0.006	*dctP3*	Carbohydrate Metabolism	Unknown
RCAP_rcc03025	-0.633	0.050	-0.171	0.486	0.448	0.236	*dctS3*	Signal Transduction	Kinase/Phosphorelay
RCAP_rcc03026	-0.843	0.003	-0.057	0.810	0.420	0.269	*dctR3*	Signal Transduction	Transcription Regulator
RCAP_rcc03027	-0.184	0.486	0.244	0.192	-0.414	0.076	*radical SAM family protein*	Unknown	Unknown
RCAP_rcc03028	-0.146	0.725	-0.071	0.814	0.299	0.512	*hypothetical protein*	Unknown	Unknown
RCAP_rcc03029	0.336	0.238	0.189	0.266	-0.381	0.173	*hypothetical protein*	Unknown	Unknown
RCAP_rcc03030	0.299	0.341	0.066	0.787	-0.419	0.178	*YiaA/B two helix domain-containing family protein*	Unknown	Unknown
RCAP_rcc03031	-0.412	0.251	0.078	0.877	-0.275	0.546	*PspA/IM30 family protein*	Signal Transduction	Unknown
RCAP_rcc03032	0.156	0.688	-0.300	0.331	-0.206	0.637	*TetR family transcriptional regulator*	Signal Transduction	Transcription Regulator
RCAP_rcc03033	-0.331	NA	-1.267	0.015	0.141	NA	*fdhE*	Carbohydrate Metabolism	Glyoxylate and dicarboxylate metabolism
RCAP_rcc03034	-0.310	0.329	-1.798	0.000	-0.070	0.887	*fdhD*	Energy Metabolism	Nitrogen metabolism
RCAP_rcc03035	-0.283	0.132	-1.675	0.000	0.236	0.165	*fdhA*	Carbohydrate Metabolism	Glyoxylate and dicarboxylate metabolism
RCAP_rcc03036	-0.563	0.015	-1.264	0.000	0.382	0.259	*fdhB*	Carbohydrate Metabolism	Glyoxylate and dicarboxylate metabolism
RCAP_rcc03037	-0.795	0.002	-1.058	0.000	0.460	0.111	*fdhC*	Carbohydrate Metabolism	Glyoxylate and dicarboxylate metabolism
RCAP_rcc03038	0.228	0.377	-0.173	0.315	-0.051	0.859	*moaE*	Metabolism of Cofactors, Coenzymes and Vitamins	Unknown
RCAP_rcc03039	-0.164	0.683	-0.602	0.017	0.535	0.183	*moaD2*	Metabolism of Cofactors, Coenzymes and Vitamins	Unknown
RCAP_rcc03040	0.162	0.585	-0.068	0.863	-0.224	0.441	*hypothetical protein*	Unknown	Unknown
RCAP_rcc03041	0.330	0.085	0.403	0.004	-0.080	0.779	*thyX*	Nucleotide Metabolism	Unknown
RCAP_rcc03042	0.130	0.425	-0.419	0.000	-0.179	0.304	*lipoprotein*	Predicted Function	Unknown

RCAP_rcc03043	-0.318	0.226	-0.374	0.009	0.066	0.866	*hypothetical protein*	Unknown	Unknown
RCAP_rcc03044	0.158	0.601	0.221	0.343	0.178	0.504	*gloA*	Sulfur Metabolism	Glutathione metabolism
RCAP_rcc03045	-0.107	0.731	0.334	0.068	0.379	0.031	*hypothetical protein*	Unknown	Unknown
RCAP_rcc03046	-0.051	0.901	0.395	0.030	0.109	0.655	*engD*	Translation, ribosomal structure and biogenesis	Unknown
RCAP_rcc03047	0.567	0.054	0.355	0.054	-0.032	0.929	*trpA*	Amino Acid Metabolism	Phenylalanine, tyrosine and tryptophan biosynthesis
RCAP_rcc03048	0.499	0.002	-1.187	0.000	-0.027	0.944	*lldD*	Carbohydrate Metabolism	Pyruvate metabolism
RCAP_rcc03049	-0.737	0.012	-1.143	0.000	0.530	0.142	*major facilitator superfamily protein*	Carbohydrate Metabolism	Unknown
RCAP_rcc03050	0.384	0.080	0.438	0.043	-0.375	0.224	*rplY*	Translation, ribosomal structure and biogenesis	Unknown
RCAP_rcc03051	0.066	0.825	0.424	0.038	-0.323	0.268	*pth*	Translation, ribosomal structure and biogenesis	Unknown
RCAP_rcc03052	-0.681	0.004	1.360	0.000	0.246	0.362	*hypothetical protein*	Unknown	Unknown
RCAP_rcc03053	-0.594	0.032	0.541	0.099	-0.251	0.504	*hypothetical protein*	Unknown	Unknown
RCAP_rcc03054	0.350	0.070	0.376	0.002	-0.246	0.346	*rpoD*	Replication, Recombination and Repair	Replication
RCAP_rcc03055	0.087	0.690	0.420	0.003	-0.715	0.000	*dnaG*	Replication, Recombination and Repair	Unknown
RCAP_rcc03056	-0.016	0.956	-0.923	0.000	-0.003	0.991	*hypothetical protein*	Unknown	Unknown
RCAP_rcc03057	-0.295	0.213	-1.067	0.000	0.152	0.538	*hypothetical protein*	Unknown	Unknown
RCAP_rcc03058	0.051	0.877	-0.740	0.002	-0.157	0.526	*hypothetical protein*	Unknown	Unknown
RCAP_rcc03059	-0.937	0.000	0.129	0.691	0.256	0.467	*TetR family transcriptional regulator*	Signal Transduction	Transcription Regulator
RCAP_rcc03060	-0.564	0.176	-0.221	0.712	0.157	0.738	*lipoprotein*	Predicted Function	Unknown
RCAP_rcc03061	-0.019	0.951	0.193	0.096	0.147	0.428	*hom*	Amino Acid Metabolism	Lysine biosynthesis
RCAP_rcc03062	0.361	0.127	-0.810	0.000	0.141	0.346	*glpX*	Carbohydrate Metabolism	Glycolysis / Gluconeogenesis
RCAP_rcc03063	-0.179	0.422	-0.172	0.310	0.207	0.514	*recJ*	Replication, Recombination and Repair	Unknown
RCAP_rcc03064	-1.464	0.000	0.229	0.484	0.519	0.188	*HPP family/CBS domain-containing protein*	Signal Transduction	Kinase/Phosphorelay
RCAP_rcc03065	-0.146	0.734	-1.141	0.000	-0.044	0.948	*efeU*	Metal and Ion Transport	Unknown
RCAP_rcc03066	0.217	0.447	-1.459	0.000	-0.585	0.034	*dyp-type peroxidase*	Metal and Ion Transport	Unknown
RCAP_rcc03067	0.196	0.422	-1.387	0.000	-0.417	0.066	*hypothetical protein*	Unknown	Unknown
RCAP_rcc03068	-0.549	0.010	0.530	0.003	0.054	0.899	*cache sensor protein*	Signal Transduction	Kinase/Phosphorelay
RCAP_rcc03069	0.451	0.044	-0.010	0.964	-0.302	0.290	*hypothetical protein*	Unknown	Unknown
RCAP_rcc03070	0.201	0.432	0.389	0.132	-0.204	0.382	*hypothetical protein*	Unknown	Unknown
RCAP_rcc03071	-0.022	0.926	-0.437	0.017	0.056	0.815	*hypothetical protein*	Unknown	Unknown
RCAP_rcc03072	0.038	0.901	-0.703	0.001	-0.318	0.253	*hypothetical protein*	Unknown	Unknown
RCAP_rcc03073	-0.227	0.237	0.540	0.000	0.075	0.727	*nusB*	Transcription	Unknown
RCAP_rcc03074	-0.214	0.326	0.124	0.394	0.291	0.079	*ribH*	Metabolism of Cofactors, Coenzymes and Vitamins	Riboflavin metabolism
RCAP_rcc03075	0.431	0.012	0.215	0.151	-0.161	0.539	*ribAB*	Metabolism of Cofactors, Coenzymes and Vitamins	Riboflavin metabolism
RCAP_rcc03076	0.163	0.453	0.565	0.000	-0.060	0.839	*ribE*	Metabolism of Cofactors, Coenzymes and Vitamins	Riboflavin metabolism
RCAP_rcc03077	-0.094	0.716	-0.029	0.896	0.006	0.989	*capsule polysaccharide modification protein family*	Cell Envelope Biosynthesis	Cell Wall Biosynthesis
RCAP_rcc03078	0.093	0.662	-0.075	0.664	-0.149	0.480	*polysaccharide biosynthesis/export family protein*	Cell Envelope Biosynthesis	Cell Wall Biosynthesis
RCAP_rcc03079	-0.233	0.420	-0.092	0.601	0.326	0.300	*capsule polysaccharide biosynthesis protein family*	Cell Envelope Biosynthesis	Cell Wall Biosynthesis
RCAP_rcc03080	-0.489	0.235	-0.356	0.110	0.675	0.066	*ribD*	Metabolism of Cofactors, Coenzymes and Vitamins	Riboflavin metabolism
RCAP_rcc03081	0.054	0.849	-0.238	0.147	-0.287	0.174	*nrdR*	Signal Transduction	Transcription Regulator
RCAP_rcc03082	0.237	0.247	-0.538	0.000	-0.042	0.845	*lipoprotein*	Predicted Function	Unknown
RCAP_rcc03083	0.406	0.224	-0.217	0.415	-0.057	0.919	*membrane bound YbgT-like protein*	Unknown	Unknown
RCAP_rcc03084	0.225	0.490	-0.127	0.533	0.037	0.938	*cydB*	Energy Metabolism	Aerobic/Anaerobic Respiration
RCAP_rcc03085	0.394	0.224	-0.086	0.729	-0.169	0.660	*cydA*	Energy Metabolism	Aerobic/Anaerobic Respiration
RCAP_rcc03086	0.038	0.927	-0.015	0.963	0.083	0.869	*hypothetical protein*	Unknown	Unknown
RCAP_rcc03087	-0.046	0.908	0.327	0.085	0.095	0.710	*GNAT family acetyltransferase*	Cell Division	Chromosome Partitioning
RCAP_rcc03088	-0.044	0.901	0.231	0.281	0.158	0.617	*flavin reductase domain-containing protein*	Unknown	Unknown
RCAP_rcc03089	0.229	0.390	-0.238	0.183	-0.079	0.722	*group 1 glycosyl transferase*	Glycan Biosynthesis and Metabolism	N-Glycan biosynthesis
RCAP_rcc03090	-0.131	0.574	-0.053	0.801	-0.123	0.750	*hypothetical protein*	Unknown	Unknown
RCAP_rcc03091	0.053	NA	1.649	0.000	0.071	0.696	*mtnP*	Amino Acid Metabolism	Cysteine and methionine metabolism
RCAP_rcc03092	-0.002	0.995	1.484	0.000	-0.079	0.782	*apt*	Nucleotide Metabolism	Purine metabolism
RCAP_rcc03093	-0.318	0.225	-0.197	0.313	0.315	0.338	*D-2-hydroxyglutarate dehydrogenase*	Energy Metabolism	Unknown
RCAP_rcc03094	-0.490	0.025	-0.146	0.315	0.288	0.394	*rimJ*	Translation, ribosomal structure and biogenesis	Unknown
RCAP_rcc03095	0.052	0.793	0.186	0.087	-0.084	0.707	*M16 family peptidase*	Post-translational Modification, Assembly and Chaperones	Peptidase
RCAP_rcc03096	0.209	0.398	0.311	0.030	-0.050	0.881	*thrC*	Metabolism of Cofactors, Coenzymes and Vitamins	Vitamin B6 metabolism
RCAP_rcc03097	-0.512	0.052	-0.092	0.587	0.196	0.577	*tldD*	Unknown	Unknown
RCAP_rcc03098	0.151	0.589	1.516	0.000	0.031	0.951	*drpA*	Replication, Recombination and Repair	Unknown
RCAP_rcc03099	-0.331	0.324	-0.662	0.002	0.560	0.136	*hypothetical protein*	Unknown	Unknown
RCAP_rcc03100	0.093	0.710	0.324	0.001	-0.050	0.839	*topA*	Replication, Recombination and Repair	Unknown
RCAP_rcc03101	0.200	0.461	-0.152	0.405	-0.090	0.696	*scoA*	Carbohydrate Metabolism	Butanoate metabolism
RCAP_rcc03102	0.481	0.078	-0.473	0.001	-0.130	0.686	*scoB*	Carbohydrate Metabolism	Butanoate metabolism
RCAP_rcc03103	-0.082	0.817	-0.388	0.039	0.218	0.563	*hypothetical protein*	Unknown	Unknown
RCAP_rcc03104	-0.218	0.421	-0.537	0.005	0.142	0.667	*FAD linked oxidase domain-containing protein*	Energy Metabolism	Unknown
RCAP_rcc03105	-0.179	0.557	-0.905	0.000	0.341	0.270	*short-chain dehydrogenase/reductase family oxidoreductase*	Lipid transport and metabolism	Unknown
RCAP_rcc03106	-0.382	0.276	-0.364	0.426	0.005	0.991	*enoyl-CoA hydratase/isomerase*	Xenobiotics Biodegradation and Metabolism	Caprolactam degradation
RCAP_rcc03107	-0.330	0.185	0.133	0.466	0.151	0.510	*hisC2*	Amino Acid Metabolism	Histidine metabolism
RCAP_rcc03108	1.702	0.000	-0.313	0.224	-0.124	0.712	*hypothetical protein*	Unknown	Unknown
RCAP_rcc03109	-0.384	0.256	-0.825	0.000	0.471	0.170	*serine--glyoxylate aminotransferase/alanine--glyoxylate aminotransferas*	Carbohydrate Metabolism	Glyoxylate and dicarboxylate metabolism
RCAP_rcc03110	-0.140	0.463	-0.124	0.268	0.128	0.453	*ABC transporter ATP-binding/permease*	Defense Mechanisms	Unknown
RCAP_rcc03111	-0.154	0.451	-0.145	0.479	0.014	0.967	*gcvT2*	Unknown	Unknown
RCAP_rcc03112	0.173	0.577	0.171	0.401	-0.135	0.697	*hypothetical protein*	Unknown	Unknown
RCAP_rcc03113	-0.077	0.755	0.752	0.000	0.244	0.381	*hypothetical protein*	Unknown	Unknown
RCAP_rcc03114	0.226	0.211	0.168	0.315	-0.141	0.540	*TolC family type I secretion outer membrane protein*	Trafficking and Secretion	Secretion
RCAP_rcc03115	-0.097	0.698	0.139	0.650	0.259	0.109	*pcm2*	Post-translational Modification, Assembly and Chaperones	Unknown
RCAP_rcc03116	-0.390	0.367	1.372	0.001	0.086	NA	*hypothetical protein*	Unknown	Unknown
RCAP_rcc03117	-0.070	NA	0.537	0.337	0.400	NA	*inositol monophosphatase*	Unknown	Unknown
RCAP_rcc03118	-0.568	0.031	-0.179	0.472	0.072	0.888	*hypothetical protein*	Unknown	Unknown
RCAP_rcc03119	0.418	0.128	-0.163	0.413	-0.447	0.015	*ion transport 2 family protein*	Metal and Ion Transport	Unknown

RCAP_rcc03120	0.542	0.003	0.590	0.000	-0.238	0.446	*ABC transporter ATP-binding protein*	Unknown	Unknown
RCAP_rcc03121	-0.254	0.514	1.189	0.005	-0.357	0.426	*hypothetical protein*	Unknown	Unknown
RCAP_rcc03122	0.268	0.322	2.420	0.000	-0.030	0.955	*FRG domain-containing protein*	Unknown	Unknown
RCAP_rcc03123	0.240	0.409	0.331	0.020	-0.076	0.801	*lepA*	Cell Envelope Biosynthesis	Cell Wall Biosynthesis
RCAP_rcc03124	0.739	0.010	-0.913	0.000	-0.389	0.121	*hypothetical protein*	Unknown	Unknown
RCAP_rcc03125	-0.078	0.802	-0.352	0.014	0.243	0.442	*heavy metal transport/detoxification protein family*	Metal, Ion, Cofactor Transport	Copper Transport
RCAP_rcc03126	0.144	0.592	0.387	0.019	-0.033	0.911	*hypothetical protein*	Unknown	Unknown
RCAP_rcc03127	-0.512	0.116	0.140	0.592	0.180	0.651	*etp*	Signal Transduction	Kinase/Phosphorelay
RCAP_rcc03128	-0.247	0.308	-0.030	0.891	0.151	0.605	*npdA*	Transcription	Unknown
RCAP_rcc03129	0.425	0.076	0.317	0.070	-0.202	0.610	*rpmB*	Translation, ribosomal structure and biogenesis	Unknown
RCAP_rcc03130	0.095	0.690	0.200	0.208	0.278	0.198	*argK*	Amino Acid Metabolism	Unknown
RCAP_rcc03131	-0.017	0.956	0.042	0.830	0.035	0.900	*hypothetical protein*	Unknown	Unknown
RCAP_rcc03132	0.080	0.806	0.144	0.262	0.004	0.991	*hrpB*	Replication, Recombination and Repair	Unknown
RCAP_rcc03133	-0.187	0.384	0.427	0.002	0.014	0.974	*entB*	Secondary metabolites biosynthesis, transport, and catabolism	Unknown
RCAP_rcc03134	0.288	0.078	0.540	0.000	-0.395	0.019	*argF*	Amino Acid Metabolism	Arginine and proline metabolism
RCAP_rcc03135	-0.126	0.689	0.487	0.001	0.006	0.990	*argD*	Amino Acid Metabolism	Arginine and proline metabolism
RCAP_rcc03136	0.222	0.352	-0.670	0.000	-0.106	0.549	*ccmF*	Energy Metabolism	Cytochrome Biogenesis
RCAP_rcc03137	-0.468	0.084	-0.789	0.000	0.501	0.192	*ccmH*	Energy Metabolism	Cytochrome Biogenesis
RCAP_rcc03138	-0.414	0.189	-0.682	0.001	0.378	0.310	*paaG*	Xenobiotics Biodegradation and Metabolism	Caprolactam degradation
RCAP_rcc03139	-0.193	0.253	-0.228	0.086	0.010	0.974	*ABC transporter ATP-binding protein/permease*	Defense Mechanisms	Unknown
RCAP_rcc03140	-0.107	0.604	0.165	0.435	-0.036	0.845	*fadD*	Lipid Metabolism	Fatty acid metabolism
RCAP_rcc03141	0.350	0.066	0.474	0.094	-0.529	0.007	*cspD*	Transcription	Unknown
RCAP_rcc03142	0.062	0.775	-0.019	0.909	-0.004	0.989	*MiaB family RNA modification enzyme*	Translation, ribosomal structure and biogenesis	Unknown
RCAP_rcc03143	0.653	0.019	0.087	0.658	-0.361	0.290	*hypothetical protein*	Unknown	Unknown
RCAP_rcc03144	-0.118	0.690	-0.170	0.366	-0.208	0.517	*GcrA cell cycle regulator*	Signal Transduction	Transcription Regulator
RCAP_rcc03145	-0.583	0.068	-0.613	0.000	0.437	0.100	*AsnC/Lrp family transcriptional regulator*	Signal Transduction	Transcription Regulator
RCAP_rcc03146	-0.032	0.903	-0.473	0.014	0.297	0.055	*gabT1*	Carbohydrate Metabolism	Butanoate metabolism
RCAP_rcc03147	-0.927	0.001	-0.766	0.006	0.242	0.555	*MerR family transcriptional regulator*	Signal Transduction	Transcription Regulator
RCAP_rcc03148	-0.528	0.030	-0.150	0.559	0.187	0.667	*hypothetical protein*	Unknown	Unknown
RCAP_rcc03149	-0.098	0.781	-0.261	0.404	-0.023	0.948	*S54 family peptidase*	Post-translational Modification, Assembly and Chaperones	Peptidase
RCAP_rcc03150	-0.442	0.075	0.241	0.459	-0.019	0.972	*alkJ*	Amino Acid Metabolism	Glycine, serine and threonine metabolism
RCAP_rcc03151	-0.090	0.626	-0.115	0.452	0.115	0.390	*pheT*	Translation, ribosomal structure and biogenesis	Aminoacyl-tRNA biosynthesis
RCAP_rcc03152	0.408	0.080	-0.128	0.521	-0.218	0.510	*hypothetical protein*	Unknown	Unknown
RCAP_rcc03153	0.401	0.047	0.255	0.085	-0.217	0.482	*pheS*	Translation, ribosomal structure and biogenesis	Aminoacyl-tRNA biosynthesis
RCAP_rcc03154	-0.211	0.471	0.554	0.001	0.028	0.886	*rplT*	Translation, ribosomal structure and biogenesis	Unknown
RCAP_rcc03155	0.437	0.013	0.629	0.000	-0.376	0.224	*rpmI*	Translation, ribosomal structure and biogenesis	Unknown
RCAP_rcc03156	0.102	0.703	0.268	0.177	0.208	0.316	*hypothetical protein*	Unknown	Unknown
RCAP_rcc03157	-0.062	0.821	0.267	0.235	0.163	0.344	*pykA2*	Carbohydrate Metabolism	Glycolysis / Gluconeogenesis
RCAP_rcc03158	0.172	0.480	-0.105	0.608	-0.124	0.704	*n-formylglutamate amidohydrolase*	Amino Acid Metabolism	Unknown
RCAP_rcc03159	-0.526	0.004	-0.602	0.000	0.188	0.297	*hemolysin-type calcium-binding repeat family protein*	Trafficking and Secretion	Secretion
RCAP_rcc03160	-0.711	0.026	-0.613	0.003	0.561	0.140	*hypothetical protein*	Unknown	Unknown
RCAP_rcc03161	0.667	0.000	-0.308	0.017	-0.164	0.585	*dat*	Metabolism of Other Amino Acids	D-Alanine metabolism
RCAP_rcc03162	-0.278	0.276	-0.934	0.000	0.445	0.146	*mandelate racemase/muconate lactonizing enzyme family protein*	Unknown	Unknown
RCAP_rcc03163	0.219	0.352	-1.090	0.000	0.082	0.750	*hypothetical protein*	Unknown	Unknown
RCAP_rcc03164	0.401	0.145	0.297	0.484	-0.277	0.413	*citE2*	Carbohydrate Metabolism	Glyoxylate and dicarboxylate metabolism
RCAP_rcc03165	0.247	0.343	0.096	0.712	-0.282	0.197	*accA*	Energy Metabolism	Reductive carboxylate cycle (CO2 fixation)
RCAP_rcc03166	0.094	0.633	0.125	0.306	-0.033	0.894	*phospholipid/glycerol acyltransferase*	Lipid Metabolism	Unknown
RCAP_rcc03167	-0.588	0.050	-0.513	0.021	0.564	0.095	*hypothetical protein*	Unknown	Unknown
RCAP_rcc03168	-0.067	0.797	-0.033	0.852	-0.101	0.640	*ftsE*	Cell Division	Chromosome Partitioning
RCAP_rcc03169	-0.037	0.873	-0.683	0.001	0.110	0.705	*zinc finger domain-containing protein*	Metal, Ion, Cofactor Transport	Zinc Transport
RCAP_rcc03170	-0.175	0.538	-0.423	0.008	0.168	0.430	*hypothetical protein*	Unknown	Unknown
RCAP_rcc03171	0.174	0.419	0.243	0.060	0.006	0.985	*lysA*	Amino Acid Metabolism	Lysine biosynthesis
RCAP_rcc03172	-0.453	0.154	-0.811	0.004	0.346	0.406	*lipoprotein*	Predicted Function	Unknown
RCAP_rcc03173	-0.329	0.456	-0.336	0.256	0.086	NA	*lipoprotein*	Predicted Function	Unknown
RCAP_rcc03174	0.223	0.308	0.196	0.152	-0.094	0.658	*argH1*	Amino Acid Metabolism	Arginine and proline metabolism
RCAP_rcc03175	0.535	0.024	0.371	0.031	-0.288	0.295	*dsbE*	Sulfur Metabolism	Unknown
RCAP_rcc03176	-0.523	0.002	1.983	0.000	0.170	0.485	*PAS/PAC sensor domain-containing protein*	Motility	Aerotaxis
RCAP_rcc03177	0.132	0.663	3.110	0.000	0.045	0.919	*EAL domain-containing protein*	Signal Transduction	Kinase/Phosphorelay
RCAP_rcc03178	0.543	0.009	-0.305	0.319	-0.096	0.727	*atoB2*	Carbohydrate Metabolism	Polyhydroxybutyrate
RCAP_rcc03179	0.234	0.154	0.386	0.189	0.063	0.819	*phbB*	Carbohydrate Metabolism	Polyhydroxybutyrate
RCAP_rcc03180	0.055	0.814	-0.941	0.000	-0.056	0.888	*hypothetical protein*	Unknown	Unknown
RCAP_rcc03181	0.632	0.015	-1.514	0.000	-0.158	0.705	*hypothetical protein*	Unknown	Unknown
RCAP_rcc03182	-0.490	0.084	-0.098	0.753	0.491	0.211	*methyltransferase small domain-containing protein*	Unknown	Unknown
RCAP_rcc03183	0.372	0.172	-0.005	0.989	-0.236	0.526	*hypothetical protein*	Unknown	Unknown
RCAP_rcc03184	0.052	0.857	0.322	0.095	-0.074	0.782	*ispB*	Lipid	Terpenoid backbone biosynthesis
RCAP_rcc03185	-0.137	0.650	-0.800	0.000	0.136	0.734	*hypothetical protein*	Unknown	Unknown
RCAP_rcc03186	0.250	0.283	-0.305	0.123	-0.191	0.476	*ATPase AAA*	Post-translational Modification, Assembly and Chaperones	Unknown
RCAP_rcc03187	-0.085	0.810	-0.773	0.000	-0.032	0.919	*AlgR/AgrA/LytR family transcriptional regulator*	Signal Transduction	Transcription Regulator
RCAP_rcc03188	0.055	0.826	0.082	0.583	0.149	0.316	*folD2*	Energy Metabolism	Reductive carboxylate cycle (CO2 fixation)
RCAP_rcc03189	-0.402	0.157	-0.172	0.523	0.340	0.406	*chorismate mutase*	Amino Acid Metabolism	Unknown
RCAP_rcc03190	-0.105	0.730	0.096	0.682	0.082	0.703	*fhs*	Energy Metabolism	Reductive carboxylate cycle (CO2 fixation)
RCAP_rcc03191	0.186	0.282	-0.119	0.629	0.088	0.728	*hemolysin-type calcium-binding repeat family protein*	Trafficking and Secretion	Secretion
RCAP_rcc03192	0.465	0.002	0.399	0.000	-0.364	0.070	*ftsH*	Post-translational Modification, Assembly and Chaperones	Unknown
RCAP_rcc03193	-0.547	0.016	-0.002	0.993	0.281	0.445	*tilS*	Cell Division	Chromosome Partitioning
RCAP_rcc03194	-0.044	0.854	-0.420	0.046	0.080	0.732	*hypothetical protein*	Unknown	Unknown
RCAP_rcc03195	-0.605	0.027	-0.197	0.328	0.420	0.095	*OmpA/MotB domain-containing protein*	Cell Envelope Biosynthesis	Cell Wall Biosynthesis
RCAP_rcc03196	0.196	0.388	-0.162	0.456	-0.055	0.837	*tolB*	Trafficking and Secretion	Secretion

RCAP_rcc03197	-0.203	0.445	0.050	0.803	0.021	0.929	*tolA*	Trafficking and Secretion	Secretion
RCAP_rcc03198	-0.297	0.277	0.186	0.183	0.097	0.760	*exbD2*	Trafficking and Secretion	Trafficing
RCAP_rcc03199	0.249	0.317	0.098	0.574	-0.305	0.024	*tolQ*	Trafficking and Secretion	Secretion
RCAP_rcc03200	0.355	0.152	-0.185	0.363	-0.289	0.385	*ybgC*	Unknown	Unknown
RCAP_rcc03201	0.355	0.199	-0.065	0.742	-0.348	0.082	*hypothetical protein*	Unknown	Unknown
RCAP_rcc03202	-0.469	0.054	-0.803	0.000	0.383	0.095	*glpK2*	Lipid Metabolism	Glycerolipid metabolism
RCAP_rcc03203	-0.116	0.704	-0.604	0.004	0.107	0.621	*glpD*	Lipid Metabolism	Glycerophospholipid metabolism
RCAP_rcc03204	0.130	0.550	0.107	0.505	-0.033	0.888	*ileS*	Translation, ribosomal structure and biogenesis	Aminoacyl-tRNA biosynthesis
RCAP_rcc03205	-0.478	0.019	0.398	0.112	0.211	0.406	*hypothetical protein*	Unknown	Unknown
RCAP_rcc03206	0.083	0.751	0.295	0.199	-0.123	0.611	*pcs*	Lipid Metabolism	Glycerophospholipid metabolism
RCAP_rcc03207	0.534	0.013	0.371	0.025	0.124	0.747	*hypothetical protein*	Unknown	Unknown
RCAP_rcc03208	0.038	0.869	0.730	0.000	0.413	0.007	*xerC*	Replication, Recombination and Repair	Recombination
RCAP_rcc03209	0.061	0.789	1.089	0.000	0.159	0.605	*hypothetical protein*	Unknown	Unknown
RCAP_rcc03210	0.570	0.006	0.077	0.743	-0.219	0.549	*talC*	Carbohydrate Metabolism	Pentose phosphate pathway
RCAP_rcc03211	-0.467	0.041	-0.076	0.681	-0.111	0.819	*priA*	Replication, Recombination and Repair	Unknown
RCAP_rcc03212	0.561	0.003	0.284	0.021	-0.793	0.001	*prmA*	Translation, ribosomal structure and biogenesis	Unknown
RCAP_rcc03213	-0.174	0.591	-0.991	0.000	0.038	0.953	*hypothetical protein*	Unknown	Unknown
RCAP_rcc03214	0.129	0.709	-1.102	0.001	0.211	0.642	*hypothetical protein*	Unknown	Unknown
RCAP_rcc03215	0.287	0.511	0.636	0.249	-0.117	NA	*hypothetical protein*	Unknown	Unknown
RCAP_rcc03216	-0.275	0.160	-0.577	0.002	-0.288	0.172	*ruvC*	Replication, Recombination and Repair	Unknown
RCAP_rcc03217	-0.335	0.145	-0.748	0.000	-0.071	0.853	*ruvA*	Replication, Recombination and Repair	Unknown
RCAP_rcc03218	0.011	0.961	-0.276	0.050	0.158	0.570	*ruvB*	Replication, Recombination and Repair	Unknown
RCAP_rcc03219	-0.241	0.263	-0.122	NA	0.176	0.430	*globin family protein*	Unknown	Unknown
RCAP_rcc03220	-0.072	0.767	-0.188	0.260	0.268	0.168	*hypothetical protein*	Unknown	Unknown
RCAP_rcc03221	0.198	0.371	0.183	0.209	-0.481	0.058	*hypothetical protein*	Unknown	Unknown
RCAP_rcc03222	-0.150	0.635	0.363	0.012	0.146	0.651	*truA*	Nucleotide Metabolism	Pyrimidine metabolism
RCAP_rcc03223	-0.250	0.105	-0.642	0.000	0.122	0.477	*capD*	Cell Envelope Biosynthesis	Cell Wall Biosynthesis
RCAP_rcc03224	-0.188	0.422	-0.347	0.019	0.057	0.875	*sugar transferase*	Cell Envelope Biosynthesis	Cell Wall Biosynthesis
RCAP_rcc03225	0.015	0.975	-0.324	0.423	-0.145	0.754	*NAD-dependent epimerase/dehydratase*	Unknown	Unknown
RCAP_rcc03226	-0.037	0.842	-0.065	0.580	0.127	0.459	*polysaccharide biosynthesis/export family protein*	Cell Envelope Biosynthesis	Cell Wall Biosynthesis
RCAP_rcc03227	0.184	0.404	-0.557	0.000	-0.054	0.789	*ilvE2*	Metabolism of Cofactors, Coenzymes and Vitamins	Pantothenate and CoA biosynthesis
RCAP_rcc03228	0.055	0.831	-0.356	0.079	-0.207	0.426	*universal stress family protein*	Stress Response	Unknown
RCAP_rcc03229	0.096	0.676	-0.412	0.071	-0.343	0.137	*NifU domain-containing protein*	Energy Metabolism	Nitrogen metabolism
RCAP_rcc03230	-0.272	0.544	-0.022	0.935	0.195	NA	*M22 family peptidase*	Post-translational Modification, Assembly and Chaperones	Peptidase
RCAP_rcc03231	-0.584	0.141	0.479	0.036	-0.146	0.764	*rimI*	Unknown	Unknown
RCAP_rcc03232	0.653	0.003	-0.663	0.000	-0.072	0.845	*basic membrane lipoprotein family*	Predicted Function	Unknown
RCAP_rcc03233	-0.001	0.997	-0.358	0.002	0.058	0.823	*monosacharide ABC transporter ATP-binding protein*	Carbohydrate Metabolism	Aerobic/Anaerobic Respiration
RCAP_rcc03234	-0.084	NA	-0.334	0.015	0.142	0.526	*monosacharide ABC transporter permease*	Carbohydrate Metabolism	Aerobic/Anaerobic Respiration
RCAP_rcc03235	-0.491	0.026	-0.310	0.030	0.414	0.066	*monosacharide ABC transporter permease*	Carbohydrate Metabolism	Aerobic/Anaerobic Respiration
RCAP_rcc03236	-0.068	0.784	-0.538	0.000	0.198	0.267	*punA*	Metabolism of Cofactors, Coenzymes and Vitamins	Nicotinate and nicotinamide metabolism
RCAP_rcc03237	0.147	0.505	-0.264	0.126	-0.213	0.225	*hypothetical protein*	Unknown	Unknown
RCAP_rcc03238	0.020	0.933	-0.172	0.243	-0.042	0.881	*hypothetical protein*	Unknown	Unknown
RCAP_rcc03239	0.630	0.004	-0.238	0.185	-0.182	0.614	*AsnC/Lrp family transcriptional regulator*	Signal Transduction	Transcription Regulator
RCAP_rcc03240	-0.422	0.043	0.202	0.344	0.380	0.224	*ATPase AAA*	Replication, Recombination and Repair	Unknown
RCAP_rcc03241	0.463	0.026	0.371	0.331	-0.355	0.095	*mutB*	Carbohydrate Metabolism	Glyoxylate and dicarboxylate metabolism
RCAP_rcc03242	0.618	0.024	-0.194	0.420	-0.188	0.616	*hypothetical protein*	Unknown	Unknown
RCAP_rcc03243	0.485	0.031	0.096	0.767	-0.344	0.157	*ccrA*	Carbohydrate Metabolism	Glyoxylate and dicarboxylate metabolism
RCAP_rcc03244	0.131	NA	0.181	0.823	0.043	NA	*hypothetical protein*	Unknown	Unknown
RCAP_rcc03245	-0.903	0.000	-0.403	0.116	0.159	0.724	*ROK family protein*	Transcription	Unknown
RCAP_rcc03246	-0.736	0.021	-0.455	0.038	0.230	0.622	*family 2 glycosyl transferase*	Unknown	Unknown
RCAP_rcc03247	-0.082	0.811	-0.448	0.041	0.172	0.621	*ceramide glucosyltransferase*	Cell Envelope Biosynthesis	Cell Wall Biosynthesis
RCAP_rcc03248	0.703	0.010	0.727	0.002	-0.936	0.004	*metallophosphoesterase*	Metabolism of Cofactors, Coenzymes and Vitamins	Folate biosynthesis
RCAP_rcc03249	-0.625	0.054	-0.178	0.442	0.526	0.070	*BioY family protein*	Metabolism of Cofactors, Coenzymes and Vitamins	Biotin metabolism
RCAP_rcc03250	-0.368	0.219	0.488	0.001	0.226	0.493	*cbiQ3*	Metabolism of Cofactors, Coenzymes and Vitamins	Cobalamin Biosynthesis
RCAP_rcc03251	-0.730	0.029	0.326	0.015	0.350	0.293	*cbiO3*	Metabolism of Cofactors, Coenzymes and Vitamins	Cobalamin Biosynthesis
RCAP_rcc03252	0.065	0.747	-0.704	0.000	-0.076	0.722	*cydD*	Energy Metabolism	Aerobic/Anaerobic Respiration
RCAP_rcc03253	-0.710	0.030	-0.831	0.000	0.756	0.043	*cydC*	Energy Metabolism	Aerobic/Anaerobic Respiration
RCAP_rcc03254	-0.289	0.334	0.629	0.004	0.093	0.789	*cytochrome P450 family protein*	Energy Metabolism	Aerobic/Anaerobic Respiration
RCAP_rcc03255	-0.039	0.922	0.585	0.037	0.085	0.782	*Crp/Fnr family transcriptional regulator*	Signal Transduction	Transcription Regulator
RCAP_rcc03256	0.904	0.000	0.044	0.855	-0.173	0.466	*cytochrome P450 family protein*	Energy Metabolism	Aerobic/Anaerobic Respiration
RCAP_rcc03257	0.626	0.009	0.148	0.622	-0.097	0.789	*msrA1*	Post-translational Modification, Assembly and Chaperones	Unknown
RCAP_rcc03258	0.617	0.014	-0.023	0.949	-0.154	0.704	*cytochrome c biogenesis protein transmembrane region*	Energy Metabolism	Aerobic/Anaerobic Respiration
RCAP_rcc03259	0.328	0.120	0.316	0.100	-0.745	0.002	*msrB1*	Post-translational Modification, Assembly and Chaperones	Unknown
RCAP_rcc03260	0.247	0.256	0.234	0.139	-0.178	0.295	*NAD-dependent epimerase/dehydratase*	Metabolism of Cofactors, Coenzymes and Vitamins	Porphyrin and chlorophyll metabolism
RCAP_rcc03261	0.482	0.103	-1.793	0.000	-0.422	0.080	*TOBE domain-containing protein*	Metabolism of Cofactors, Coenzymes and Vitamins	Unknown
RCAP_rcc03262	0.274	0.374	0.314	0.263	-0.565	0.083	*hypothetical protein*	Unknown	Unknown
RCAP_rcc03263	-0.162	NA	-0.154	0.816	-0.261	0.559	*NifT/FixU family protein*	Energy Metabolism	Nitrogen metabolism
RCAP_rcc03264	0.756	0.040	-0.477	0.398	-0.289	0.506	*NifZ family protein*	Energy Metabolism	Nitrogen metabolism
RCAP_rcc03265	0.329	0.454	0.376	0.549	-0.295	0.451	*LRV FeS4 cluster domain-containing protein*	Unknown	Unknown
RCAP_rcc03266	0.127	0.690	-0.068	0.758	-0.015	0.974	*nifB2*	Energy Metabolism	Nitrogen metabolism
RCAP_rcc03267	-0.143	0.626	0.449	0.036	-0.194	0.635	*nifA2*	Signal Transduction	Transcription Regulator
RCAP_rcc03268	0.031	0.955	0.152	0.760	-0.336	0.457	*nifW*	Energy Metabolism	Nitrogen metabolism
RCAP_rcc03269	-0.312	NA	0.266	0.648	-0.157	NA	*nifV*	Energy Metabolism	Nitrogen metabolism
RCAP_rcc03270	0.128	NA	0.426	0.429	0.118	0.813	*nifS*	Metabolism of Cofactors, Coenzymes and Vitamins	Nitrogen metabolism
RCAP_rcc03271	0.095	NA	-0.032	0.967	-0.162	NA	*nifU2*	Energy Metabolism	Nitrogen metabolism
RCAP_rcc03272	-0.001	NA	-0.397	0.592	-0.168	NA	*HesB/YadR/YfhF family protein*	Unknown	Unknown
RCAP_rcc03273	0.217	NA	-0.394	0.592	0.004	NA	*hypothetical protein*	Unknown	Unknown

RCAP_rcc03274	-0.081	NA	-0.058	0.935	-0.092	NA	*NifQ family protein*	Energy Metabolism	Nitrogen metabolism
RCAP_rcc03275	0.565	0.154	-0.310	0.575	-0.288	0.526	*fdxB*	Energy Metabolism	Aerobic/Anaerobic Respiration
RCAP_rcc03276	0.677	0.078	-0.171	0.706	-0.304	0.504	*hypothetical protein*	Unknown	Unknown
RCAP_rcc03277	0.385	0.322	0.045	0.924	-0.653	0.087	*hypothetical protein*	Unknown	Unknown
RCAP_rcc03278	0.258	0.559	-0.407	0.438	-0.184	0.689	*nifX*	Energy Metabolism	Nitrogen metabolism
RCAP_rcc03279	-0.362	0.411	-0.384	0.421	0.042	0.949	*nifN*	Energy Metabolism	Nitrogen metabolism
RCAP_rcc03280	0.207	0.517	0.387	0.279	-0.517	0.066	*nifE*	Energy Metabolism	Nitrogen metabolism
RCAP_rcc03281	0.270	0.431	0.357	0.183	-0.293	0.429	*peroxiredoxin*	Post-translational Modification, Assembly and Chaperones	Unknown
RCAP_rcc03282	-0.323	0.433	0.102	0.795	-0.365	0.406	*rnfF*	Energy Metabolism	Nitrogen metabolism
RCAP_rcc03283	-0.182	NA	-0.506	0.463	0.035	NA	*rseC*	Signal Transduction	Transcription Regulator
RCAP_rcc03284	1.042	0.003	0.238	0.650	-0.602	0.064	*fdxN*	Energy Metabolism	Aerobic/Anaerobic Respiration
RCAP_rcc03285	0.877	0.027	-0.415	0.521	-0.671	0.079	*fdxC*	Energy Metabolism	Aerobic/Anaerobic Respiration
RCAP_rcc03286	0.189	0.682	-0.103	0.856	-0.431	0.295	*norV*	Unknown	Unknown
RCAP_rcc03287	-0.389	NA	0.387	0.601	-0.159	0.736	*rnfA*	Energy Metabolism	Unknown
RCAP_rcc03288	-0.101	NA	0.132	0.864	-0.204	NA	*rnfB*	Energy Metabolism	Unknown
RCAP_rcc03289	0.054	0.913	0.147	0.763	-0.375	0.391	*rnfC*	Energy Metabolism	Unknown
RCAP_rcc03290	-0.360	0.391	-0.247	0.592	-0.352	0.432	*rnfD*	Energy Metabolism	Unknown
RCAP_rcc03291	0.313	0.484	-0.585	0.280	-0.040	0.949	*rnfG*	Energy Metabolism	Unknown
RCAP_rcc03292	0.279	0.529	-0.282	0.569	-0.459	0.267	*rnfE*	Energy Metabolism	Unknown
RCAP_rcc03293	0.362	0.411	-0.168	0.814	-0.201	NA	*hypothetical protein*	Unknown	Unknown
RCAP_rcc03294	-0.090	NA	-0.642	0.264	-0.274	NA	*fccB*	Energy Metabolism	Unknown
RCAP_rcc03295	0.146	0.488	-0.277	0.203	0.265	0.395	*hypothetical protein*	Unknown	Unknown
RCAP_rcc03296	0.132	0.613	-0.014	0.963	-0.005	0.991	*betC*	Metal and Ion Transport	Unknown
RCAP_rcc03297	-0.131	0.589	-0.013	0.964	0.036	0.914	*glycine betaine/L-proline ABC transporter periplasmic glycine betaine/L-*	Amino Acid Metabolism	Amino Acid Transport
RCAP_rcc03298	-1.005	0.001	1.036	0.000	0.715	0.053	*LysR family transcriptional regulator*	Signal Transduction	Transcription Regulator
RCAP_rcc03299	-0.328	0.279	2.652	0.000	0.392	0.358	*hypothetical protein*	Unknown	Unknown
RCAP_rcc03300	-0.313	0.333	-0.003	0.993	0.078	0.820	*radical SAM family protein*	Unknown	Unknown
RCAP_rcc03301	-0.608	0.002	1.727	0.000	0.277	0.161	*diguanylate cyclase/phosphodiesterase*	Signal Transduction	Kinase/Phosphorelay
RCAP_rcc03302	-0.018	0.958	-0.320	0.168	-0.233	0.561	*GNAT family acetyltransferase*	Cell Division	Chromosome Partitioning
RCAP_rcc03303	0.261	0.123	-0.177	0.158	-0.113	0.550	*dapE*	Amino Acid Metabolism	Lysine biosynthesis
RCAP_rcc03304	1.260	0.000	-0.331	0.157	-0.476	0.001	*hypothetical protein*	Unknown	Unknown
RCAP_rcc03305	0.483	0.026	-0.466	0.040	0.131	0.700	*hypothetical protein*	Unknown	Unknown
RCAP_rcc03306	0.889	0.000	0.270	0.181	-0.310	0.078	*acyl-CoA dehydrogenase, medium-chain specific*	Carbohydrate Metabolism	Glyoxylate and dicarboxylate metabolism
RCAP_rcc03307	0.415	0.069	-0.089	0.776	0.239	0.540	*GNAT family acetyltransferase*	Cell Division	Chromosome Partitioning
RCAP_rcc03308	-0.225	0.511	0.406	0.101	-0.103	0.819	*recO*	Replication, Recombination and Repair	Recombination
RCAP_rcc03309	-0.090	0.767	0.508	0.083	-0.103	0.730	*hypothetical protein*	Unknown	Unknown
RCAP_rcc03310	-0.099	0.559	0.407	0.002	-0.045	0.831	*era*	Unknown	Unknown
RCAP_rcc03311	-0.463	0.039	0.221	0.243	0.059	0.874	*rnc*	Transcription	Unknown
RCAP_rcc03312	0.416	0.028	0.461	0.000	-0.221	0.492	*lepB*	Trafficking and Secretion	Trafficing
RCAP_rcc03313	-0.313	0.284	-0.046	0.814	0.282	0.337	*acpS*	Metabolism of Cofactors, Coenzymes and Vitamins	Pantothenate and CoA biosynthesis
RCAP_rcc03314	0.111	0.697	-0.326	0.013	-0.082	0.803	*hypothetical protein*	Unknown	Unknown
RCAP_rcc03315	0.101	0.732	-0.449	0.039	0.193	0.289	*pdxJ*	Metabolism of Cofactors, Coenzymes and Vitamins	Vitamin B6 metabolism
RCAP_rcc03316	-0.320	0.125	0.142	0.514	0.174	0.508	*hypothetical protein*	Unknown	Unknown
RCAP_rcc03317	0.197	0.189	0.256	0.109	-0.157	0.499	*spoT*	Nucleotide Metabolism	Purine metabolism
RCAP_rcc03318	-0.171	0.483	0.112	0.485	0.074	0.782	*rpoZ*	Replication, Recombination and Repair	Replication
RCAP_rcc03319	0.407	0.081	0.280	0.158	-0.190	0.563	*folK*	Metabolism of Cofactors, Coenzymes and Vitamins	Folate biosynthesis
RCAP_rcc03320	0.582	0.001	0.144	0.372	-0.376	0.153	*hypothetical protein*	Unknown	Unknown
RCAP_rcc03321	-0.180	0.531	0.425	0.006	0.030	0.919	*rluA*	Nucleotide Metabolism	Pyrimidine metabolism
RCAP_rcc03322	-0.668	0.019	-0.469	0.008	0.479	0.224	*GAF domain-containing protein*	Signal Transduction	Kinase/Phosphorelay
RCAP_rcc03323	-0.182	NA	4.352	0.000	0.082	0.830	*rsbV*	Unknown	Unknown
RCAP_rcc03324	-0.611	0.035	1.757	0.000	0.309	0.285	*rsbW*	Signal Transduction	Kinase/Phosphorelay
RCAP_rcc03325	0.714	0.003	-0.390	0.002	-0.433	0.136	*hemimethylated DNA-binding protein family*	Stress Response	Unknown
RCAP_rcc03326	0.739	0.001	-0.078	0.646	-0.609	0.009	*lipoprotein*	Predicted Function	Unknown
RCAP_rcc03327	0.579	0.060	-0.157	0.320	-0.715	0.000	*lolA*	Cell Envelope Biosynthesis	Cell Wall Biosynthesis
RCAP_rcc03328	0.179	0.458	0.099	0.544	-0.162	0.512	*ftsK*	Cell Division	Chromosome Partitioning
RCAP_rcc03329	-0.168	0.334	0.522	0.001	-0.074	0.745	*class I/II aminotransferase*	Amino Acid Metabolism	Lysine biosynthesis
RCAP_rcc03330	-0.169	0.396	-0.349	0.037	0.151	0.555	*amidase*	Translation, ribosomal structure and biogenesis	Unknown
RCAP_rcc03331	0.122	0.595	-0.146	0.336	-0.265	0.056	*ubiF*	Photosynthesis	Biosynthesis of Ubiquinone
RCAP_rcc03332	-0.702	0.025	-0.374	0.040	0.567	0.148	*hypothetical protein*	Unknown	Unknown
RCAP_rcc03333	-0.175	0.435	-0.415	0.000	-0.092	0.702	*S16 family peptidase*	Post-translational Modification, Assembly and Chaperones	Peptidase
RCAP_rcc03334	-0.050	0.817	-0.405	0.008	0.003	0.991	*trxA2*	Post-translational Modification, Assembly and Chaperones	Unknown
RCAP_rcc03335	0.352	0.171	-0.076	0.693	-0.253	0.394	*xthA2*	Replication, Recombination and Repair	Unknown
RCAP_rcc03336	-0.078	0.817	0.028	0.913	-0.428	0.133	*XRE family transcriptional regulator*	Signal Transduction	Transcription Regulator
RCAP_rcc03337	-0.172	0.651	0.304	0.270	-0.109	0.826	*aceB*	Carbohydrate Metabolism	Glyoxylate and dicarboxylate metabolism
RCAP_rcc03338	0.203	0.421	0.558	0.001	-0.478	0.109	*aceA*	Carbohydrate Metabolism	Glyoxylate and dicarboxylate metabolism
RCAP_rcc03339	-0.134	NA	1.611	0.000	0.358	0.393	*hypothetical protein*	Unknown	Unknown
RCAP_rcc03340	-0.178	0.684	2.061	0.000	0.045	0.946	*hypothetical protein*	Unknown	Unknown
RCAP_rcc03341	-0.554	0.031	2.041	0.000	0.172	NA	*hypothetical protein*	Unknown	Unknown
RCAP_rcc03342	0.646	0.043	1.277	0.000	-0.322	0.216	*nrdD*	Nucleotide Metabolism	Pyrimidine metabolism
RCAP_rcc03343	0.834	0.007	0.873	0.000	-0.357	0.140	*hypothetical protein*	Unknown	Unknown
RCAP_rcc03344	-0.067	0.883	0.634	0.023	0.265	0.560	*anaerobic ribonucleoside-triphosphate reductase activating protein*	Post-translational Modification, Assembly and Chaperones	Unknown
RCAP_rcc03345	-0.275	0.352	0.814	0.000	-0.039	0.948	*XRE family transcriptional regulator*	Signal Transduction	Transcription Regulator
RCAP_rcc03346	0.107	NA	0.876	0.186	-0.268	NA	*thioesterase superfamily protein*	Lipid Metabolism	Unknown
RCAP_rcc03347	0.028	0.956	0.740	0.025	-0.799	0.027	*prpD*	Carbohydrate Metabolism	Propanoate metabolism
RCAP_rcc03348	0.330	0.334	-0.229	0.556	0.145	0.752	*prpC*	Carbohydrate Metabolism	Propanoate metabolism
RCAP_rcc03349	-0.289	0.469	-0.077	0.836	-0.028	0.964	*prpB*	Carbohydrate Metabolism	Propanoate metabolism
RCAP_rcc03350	-0.390	0.228	-1.869	0.000	-0.046	0.946	*ferredoxin domain-containing protein oxidireductase*	Energy Metabolism	Aerobic/Anaerobic Respiration

RCAP_rcc03351	-0.046	0.901	-1.545	0.000	-0.025	0.960	*rieske (2Fe-2S) domain-containing protein*	Xenobiotics Biodegradation and Metabolism	Fluorobenzoate degradation
RCAP_rcc03352	0.036	0.911	0.509	0.166	-0.698	0.002	*metH2*	Metabolism of Cofactors, Coenzymes and Vitamins	One carbon pool by folate
RCAP_rcc03353	0.003	0.995	0.956	0.007	-0.626	0.059	*metH1*	Metabolism of Cofactors, Coenzymes and Vitamins	One carbon pool by folate
RCAP_rcc03354	-0.716	0.016	1.466	0.000	0.089	0.860	*cobQ2*	Metabolism of Cofactors, Coenzymes and Vitamins	Cobalamin Biosynthesis
RCAP_rcc03355	-0.840	0.023	1.304	0.000	0.175	0.704	*cobQ3*	Metabolism of Cofactors, Coenzymes and Vitamins	Cobalamin Biosynthesis
RCAP_rcc03356	-0.624	NA	1.424	0.000	0.201	0.522	*hypothetical protein*	Unknown	Unknown
RCAP_rcc03357	-0.514	NA	0.644	0.113	0.127	0.788	*hypothetical protein*	Unknown	Unknown
RCAP_rcc03358	0.263	0.410	2.709	0.000	-0.259	0.526	*TonB-dependent receptor*	Metal and Ion Transport	Unknown
RCAP_rcc03359	-0.247	0.584	2.550	0.000	0.018	0.978	*iron siderophore/cobalamin ABC transporter periplasmic iron siderophor*	Metal, Ion, Cofactor Transport	Iron and Heme Transport
RCAP_rcc03360	0.018	0.972	1.687	0.000	0.080	0.881	*iron siderophore/cobalamin ABC transporter permease*	Metal, Ion, Cofactor Transport	Iron and Heme Transport
RCAP_rcc03361	-0.403	NA	1.516	0.000	0.052	NA	*iron siderophore/cobalamin ABC transporter ATP-binding protein*	Metal, Ion, Cofactor Transport	Iron and Heme Transport
RCAP_rcc03362	-0.653	NA	1.740	0.000	0.285	NA	*cbiZ*	Metabolism of Cofactors, Coenzymes and Vitamins	Cobalamin Biosynthesis
RCAP_rcc03363	-0.649	0.032	-0.435	0.238	0.171	0.719	*bioB*	Metabolism of Cofactors, Coenzymes and Vitamins	Biotin metabolism
RCAP_rcc03364	-0.968	0.009	-0.149	0.715	0.189	0.696	*bioF*	Metabolism of Cofactors, Coenzymes and Vitamins	Biotin metabolism
RCAP_rcc03365	-0.176	NA	-0.211	0.695	0.294	NA	*bioD*	Metabolism of Cofactors, Coenzymes and Vitamins	Biotin metabolism
RCAP_rcc03366	0.008	0.982	0.201	0.424	-0.112	0.799	*bioA*	Metabolism of Cofactors, Coenzymes and Vitamins	Biotin metabolism
RCAP_rcc03367	0.071	NA	-0.996	0.113	-0.035	NA	*hypothetical protein*	Unknown	Unknown
RCAP_rcc03368	0.200	NA	-0.455	0.392	-0.073	NA	*type 11 family methyltransferase*	Metabolism of Cofactors, Coenzymes and Vitamins	Unknown
RCAP_rcc03369	-0.296	0.458	0.170	0.543	0.379	0.369	*GntR family transcriptional regulator*	Signal Transduction	Transcription Regulator
RCAP_rcc03370	-0.236	NA	-0.051	0.949	-0.038	NA	*LysR family transcriptional regulator*	Signal Transduction	Transcription Regulator
RCAP_rcc03371	-0.564	0.012	-0.568	0.008	0.349	0.257	*fabI*	Lipid Metabolism	Fatty acid biosynthesis
RCAP_rcc03372	-0.210	0.255	-0.970	0.000	0.269	0.204	*hypothetical protein*	Unknown	Unknown
RCAP_rcc03373	-0.816	0.009	-0.767	0.000	0.536	0.170	*ackA3*	Energy Metabolism	Reductive carboxylate cycle (CO2 fixation)
RCAP_rcc03374	0.110	0.730	-0.777	0.001	-0.233	0.558	*hypothetical protein*	Unknown	Unknown
RCAP_rcc03375	-0.303	0.427	-0.183	0.633	-0.315	0.446	*phnX*	Metabolism of Other Amino Acids	Phosphonate and phosphinate metabolism
RCAP_rcc03376	-0.126	0.744	0.356	0.084	0.364	0.356	*hypothetical protein*	Unknown	Unknown
RCAP_rcc03377	0.091	0.609	3.039	0.000	-0.164	0.611	*hypothetical protein*	Unknown	Unknown
RCAP_rcc03378	-0.738	0.000	0.194	0.233	0.365	0.371	*CDA peptide synthetase III*	Lipid Metabolism	Fatty acid metabolism
RCAP_rcc03379	0.491	0.014	0.438	0.010	-0.229	0.482	*hypothetical protein*	Unknown	Unknown
RCAP_rcc03380	0.124	0.633	0.197	0.392	-0.562	0.000	*smpB*	Post-translational Modification, Assembly and Chaperones	Unknown
RCAP_rcc03381	0.294	0.093	-0.131	0.380	-0.303	0.003	*sseA*	Metal and Ion Transport	Unknown
RCAP_rcc03382	0.189	0.310	-0.529	0.002	-0.008	0.978	*tyrB*	Amino Acid Metabolism	Phenylalanine, tyrosine and tryptophan biosynthesis
RCAP_rcc03383	-0.159	0.677	-0.233	0.425	-0.245	0.593	*MarR family transcriptional regulator*	Signal Transduction	Transcription Regulator
RCAP_rcc03384	-0.373	0.247	-0.312	0.053	-0.363	0.361	*emrA*	Defense Mechanisms	Unknown
RCAP_rcc03385	0.128	0.632	-0.292	0.041	-0.582	0.001	*emrB*	Metal and Ion Transport	Unknown
RCAP_rcc03386	0.490	0.005	-0.052	0.765	-0.624	0.000	*amtB*	Metal and Ion Transport	Unknown
RCAP_rcc03387	0.723	0.002	-0.241	0.287	-1.169	0.000	*glnB2*	Signal Transduction	Transcription Regulator
RCAP_rcc03388	-0.249	0.211	-0.130	0.223	0.188	0.357	*1A family penicillin-binding protein*	Glycan Biosynthesis and Metabolism	Peptidoglycan biosynthesis
RCAP_rcc03389	0.398	0.059	-0.014	0.929	-0.180	0.482	*toluene tolerance family protein*	Secondary metabolites biosynthesis, transport, and catabolism	Unknown
RCAP_rcc03390	0.400	0.018	0.191	0.214	-0.457	0.004	*VacJ family lipoprotein*	Cell Envelope Biosynthesis	Cell Wall Biosynthesis
RCAP_rcc03391	-0.116	0.545	-0.279	0.018	0.102	0.577	*type I secretion system ATPase*	Trafficking and Secretion	Secretion
RCAP_rcc03392	-0.909	0.000	-0.490	0.000	0.447	0.122	*hemolysin D*	Trafficking and Secretion	Secretion
RCAP_rcc03393	-0.057	0.793	-0.201	0.255	0.159	0.459	*lytic murein transglycosylase*	Glycan Biosynthesis and Metabolism	Glycosaminoglycan degradation
RCAP_rcc03394	-0.464	0.029	-0.085	0.753	0.341	0.206	*hypothetical protein*	Unknown	Unknown
RCAP_rcc03395	-0.841	0.002	0.752	0.000	0.402	0.267	*fmt*	Translation, ribosomal structure and biogenesis	Aminoacyl-tRNA biosynthesis
RCAP_rcc03396	-0.345	0.176	0.825	0.001	0.214	0.430	*def1*	Translation, ribosomal structure and biogenesis	Unknown
RCAP_rcc03397	0.376	0.055	0.832	0.000	-0.316	0.324	*def2*	Translation, ribosomal structure and biogenesis	Unknown
RCAP_rcc03398	0.127	0.601	0.452	0.001	-0.163	0.428	*malY*	Amino Acid Metabolism	Unknown
RCAP_rcc03399	0.590	0.013	0.067	0.743	-0.494	0.043	*hypothetical protein*	Unknown	Unknown
RCAP_rcc03400	-0.272	0.503	-0.340	0.201	0.577	0.131	*hypothetical protein*	Unknown	Unknown
RCAP_rcc03401	0.582	0.002	-0.101	0.516	-0.179	0.452	*band 7 protein family*	Post-translational Modification, Assembly and Chaperones	Unknown
RCAP_rcc03402	0.113	0.687	-0.294	0.187	0.347	0.122	*hypothetical protein*	Unknown	Unknown
RCAP_rcc03403	0.608	0.048	-2.322	0.000	-0.226	0.504	*hypothetical protein*	Unknown	Unknown
RCAP_rcc03404	0.643	0.001	0.494	0.001	-0.543	0.011	*pyrF*	Nucleotide Metabolism	Pyrimidine metabolism
RCAP_rcc03405	0.346	0.286	1.220	0.000	-0.619	0.046	*hypothetical protein*	Unknown	Unknown
RCAP_rcc03406	0.572	0.013	-0.352	0.201	-0.407	0.059	*clpB*	Post-translational Modification, Assembly and Chaperones	Unknown
RCAP_rcc03407	-0.435	0.089	-0.088	0.716	0.002	0.995	*dinB*	Replication, Recombination and Repair	Replication
RCAP_rcc03408	0.595	0.016	-0.150	0.398	-0.293	0.277	*hutG*	Carbohydrate Metabolism	Glyoxylate and dicarboxylate metabolism
RCAP_rcc03409	0.369	0.095	0.760	0.014	-0.194	0.601	*rpmJ*	Translation, ribosomal structure and biogenesis	Unknown
RCAP_rcc03410	-0.474	0.038	0.134	0.598	0.232	0.516	*HAD superfamily hydrolase*	Unknown	Unknown
RCAP_rcc03411	-0.988	0.002	0.058	0.752	0.534	0.171	*methyltransferase*	Replication, Recombination and Repair	Unknown
RCAP_rcc03412	-0.225	0.371	0.119	0.478	0.292	0.267	*thcD*	Lipid Metabolism	Fatty acid metabolism
RCAP_rcc03413	-0.341	0.181	0.089	0.795	0.220	0.511	*peroxiredoxin*	Post-translational Modification, Assembly and Chaperones	Unknown
RCAP_rcc03414	-0.121	0.669	-0.411	0.012	0.126	0.706	*hypothetical protein*	Unknown	Unknown
RCAP_rcc03415	-0.229	0.327	-0.101	0.649	0.369	0.065	*ugpQ*	Lipid Metabolism	Glycerophospholipid metabolism
RCAP_rcc03416	0.511	0.007	-0.060	0.798	-0.195	0.519	*endoribonuclease L-PSP family protein*	Unknown	Unknown
RCAP_rcc03417	0.564	0.008	0.119	0.437	-0.172	0.640	*hypothetical protein*	Unknown	Unknown
RCAP_rcc03418	0.125	0.542	1.374	0.000	0.115	0.593	*smc*	Cell Division	Chromosome Partitioning
RCAP_rcc03419	-0.454	0.127	-1.208	0.000	-0.211	0.526	*LrgA family protein*	Unknown	Unknown
RCAP_rcc03420	0.034	0.929	-1.572	0.000	-0.129	0.657	*LrgB family protein*	Cell Envelope Biosynthesis	Cell Wall Biosynthesis
RCAP_rcc03421	-0.378	0.108	0.819	0.001	-0.300	0.050	*rnhA2*	Replication, Recombination and Repair	Unknown
RCAP_rcc03422	0.162	0.350	0.430	0.001	-0.004	0.991	*LysE family transporter*	Unknown	Unknown
RCAP_rcc03423	0.029	0.881	0.403	0.008	-0.178	0.456	*ispH*	Lipid	Terpenoid backbone biosynthesis
RCAP_rcc03424	0.074	0.812	-0.971	0.000	0.042	0.851	*winged helix family two component transcriptional regulator*	Signal Transduction	Transcription Regulator
RCAP_rcc03425	-0.224	0.311	-0.688	0.000	0.259	0.211	*sensor histidine kinase/response regulator receiver protein*	Signal Transduction	Transcription Regulator
RCAP_rcc03426	0.624	0.001	1.203	NA	-0.015	0.973	*AMP-dependent synthetase and ligase*	Lipid Metabolism	Fatty acid metabolism
RCAP_rcc03427	0.494	0.047	1.193	NA	-0.059	0.846	*livG3*	Amino Acid Metabolism	Amino Acid Transport

RCAP_rcc03428	0.423	0.050	1.237	0.004	-0.001	0.997	*livH3*	Amino Acid Metabolism	Amino Acid Transport
RCAP_rcc03429	0.004	0.987	-2.301	0.000	-0.045	0.899	*hypothetical protein*	Unknown	Unknown
RCAP_rcc03430	0.360	0.076	-2.101	0.000	-0.001	0.995	*livM3*	Amino Acid Metabolism	Amino Acid Transport
RCAP_rcc03431	0.590	0.002	-0.682	0.054	-0.058	0.840	*livK2*	Amino Acid Metabolism	Amino Acid Transport
RCAP_rcc03432	0.360	0.054	-0.913	0.001	0.044	0.888	*livF3*	Amino Acid Metabolism	Amino Acid Transport
RCAP_rcc03433	-0.222	0.266	0.290	0.052	0.293	0.236	*paaK*	Amino Acid Metabolism	Phenylalanine metabolism
RCAP_rcc03434	0.246	0.189	0.480	0.000	-0.274	0.142	*map*	Translation, ribosomal structure and biogenesis	Unknown
RCAP_rcc03435	0.435	0.039	-0.021	0.928	-0.187	0.514	*molybdopterin binding domain-containing protein*	Metabolism of Cofactors, Coenzymes and Vitamins	Unknown
RCAP_rcc03436	0.016	0.969	-0.451	0.054	0.205	0.600	*GNAT family acetyltransferase*	Cell Division	Chromosome Partitioning
RCAP_rcc03437	-0.078	0.818	-0.375	0.009	0.057	0.869	*OmpA/MotB domain-containing protein*	Cell Envelope Biosynthesis	Cell Wall Biosynthesis
RCAP_rcc03438	0.190	0.426	0.194	0.410	-0.088	0.720	*LysR family transcriptional regulator*	Signal Transduction	Transcription Regulator
RCAP_rcc03439	-0.689	0.004	-0.503	0.012	0.104	0.628	*fadH*	Lipid Metabolism	Fatty acid metabolism
RCAP_rcc03440	-0.711	0.001	-0.302	0.234	0.477	0.010	*hypothetical protein*	Unknown	Unknown
RCAP_rcc03441	0.052	0.832	-0.468	0.006	0.076	0.756	*hypothetical protein*	Unknown	Unknown
RCAP_rcc03442	-0.050	0.860	-0.095	0.592	0.248	0.266	*lipoprotein*	Predicted Function	Unknown
RCAP_rcc03443	-1.087	0.000	-0.272	0.113	0.757	0.038	*BadF/BadG/BcrA/BcrD family ATPase*	Carbohydrate Metabolism	Amino sugar and nucleotide sugar metabolism
RCAP_rcc03444	-0.617	0.019	-0.061	0.777	0.447	0.231	*nagA*	Carbohydrate Metabolism	Amino sugar and nucleotide sugar metabolism
RCAP_rcc03445	0.306	0.176	0.373	0.012	-0.238	0.406	*serB*	Energy Metabolism	Methane metabolism
RCAP_rcc03446	0.300	0.182	0.119	0.753	-0.093	0.782	*serC*	Metabolism of Cofactors, Coenzymes and Vitamins	Vitamin B6 metabolism
RCAP_rcc03447	0.592	0.007	0.335	0.290	-0.317	0.268	*serA*	Energy Metabolism	Methane metabolism
RCAP_rcc03448	0.662	0.000	-0.925	0.000	-0.310	0.443	*serine/threonine-protein phosphatase*	Signal Transduction	Kinase/Phosphorelay
RCAP_rcc03449	-0.544	0.024	-0.184	0.427	-0.024	0.964	*atoB3*	Carbohydrate Metabolism	Glyoxylate and dicarboxylate metabolism
RCAP_rcc03450	-0.569	0.022	-0.837	0.000	0.467	0.195	*pbpC*	Glycan Biosynthesis and Metabolism	Peptidoglycan biosynthesis
RCAP_rcc03451	-0.079	0.714	-0.878	0.000	0.367	0.002	*alpha-2-macroglobulin domain-containing protein*	Unknown	Unknown
RCAP_rcc03452	-0.063	0.795	3.467	0.000	0.081	0.824	*sensor histidine kinase/response regulator receiver protein*	Signal Transduction	Transcription Regulator
RCAP_rcc03453	-0.596	0.022	0.656	0.000	0.089	0.809	*recQ*	Replication, Recombination and Repair	Unknown
RCAP_rcc03454	0.445	0.045	0.179	0.346	-0.451	0.072	*hypothetical protein*	Unknown	Unknown
RCAP_rcc03455	-0.022	0.936	0.126	0.662	-0.090	0.743	*hypothetical protein*	Unknown	Unknown
RCAP_rcc03456	0.412	0.035	-0.485	0.006	-0.054	0.886	*major facilitator superfamily protein*	Unknown	Unknown
RCAP_rcc03457	-0.072	0.817	-0.667	0.000	0.089	0.796	*mepA*	Cell Envelope Biosynthesis	Cell Wall Biosynthesis
RCAP_rcc03458	-0.015	0.958	-0.500	0.002	0.480	0.024	*hypothetical protein*	Unknown	Unknown
RCAP_rcc03459	0.515	0.121	0.461	0.030	-0.208	0.556	*hypothetical protein*	Unknown	Unknown
RCAP_rcc03460	0.426	0.061	0.315	0.096	-0.210	0.531	*xseB*	Replication, Recombination and Repair	Replication
RCAP_rcc03461	-0.237	0.514	0.219	NA	0.360	0.137	*ispA*	Lipid	Terpenoid backbone biosynthesis
RCAP_rcc03462	-0.140	0.374	0.401	0.004	0.062	0.687	*dxs2*	Photosynthesis	Terpenoid backbone biosynthesis
RCAP_rcc03463	-0.708	0.000	-0.058	0.700	0.347	0.171	*arcB2*	Amino Acid Metabolism	Arginine and proline metabolism
RCAP_rcc03464	-0.193	0.356	-0.174	0.259	-0.132	0.628	*HAD superfamily hydrolase*	Xenobiotics Biodegradation and Metabolism	Tetrachloroethene degradation
RCAP_rcc03465	-0.563	0.025	-0.048	0.874	0.184	NA	*membrane transport family protein*	Unknown	Unknown
RCAP_rcc03466	-0.413	0.116	-1.313	0.001	-0.204	0.651	*hypothetical protein*	Unknown	Unknown
RCAP_rcc03467	-0.275	0.541	-0.089	0.860	0.103	0.842	*hypothetical protein*	Unknown	Unknown
RCAP_rcc03468	0.248	0.282	-0.041	0.834	-0.514	0.059	*pncA*	Metabolism of Cofactors, Coenzymes and Vitamins	Nicotinate and nicotinamide metabolism
RCAP_rcc03469	0.130	0.558	0.060	0.752	-0.320	0.027	*pncB*	Metabolism of Cofactors, Coenzymes and Vitamins	Nicotinate and nicotinamide metabolism
RCAP_rcc03470	-0.147	0.651	0.277	0.061	0.196	0.222	*phosphate transporter*	Metal and Ion Transport	Unknown
RCAP_rcc03471	-0.011	0.975	0.021	0.956	-0.040	0.888	*NUDIX superfamily hydrolase*	Replication, Recombination and Repair	Unknown
RCAP_rcc03472	-0.651	0.006	-0.288	0.144	0.429	0.182	*thrB*	Unknown	Unknown
RCAP_rcc03473	-0.411	0.029	0.118	0.529	0.006	0.986	*gabT2*	Carbohydrate Metabolism	Propanoate metabolism
RCAP_rcc03474	-0.279	0.208	-0.110	0.678	-0.341	0.207	*class I peptide chain release factor*	Translation, ribosomal structure and biogenesis	Unknown
RCAP_rcc03475	-0.437	0.068	0.071	0.758	0.026	0.937	*MiaB family RNA modification enzyme*	Translation, ribosomal structure and biogenesis	Unknown
RCAP_rcc03476	-0.117	0.722	-0.081	0.751	0.141	0.701	*dapF*	Amino Acid Metabolism	Lysine biosynthesis
RCAP_rcc03477	-0.211	0.458	0.107	0.659	-0.015	0.974	*msrB2*	Post-translational Modification, Assembly and Chaperones	Unknown
RCAP_rcc03478	0.247	0.299	0.106	0.494	0.079	0.789	*msrA2*	Post-translational Modification, Assembly and Chaperones	Unknown
RCAP_rcc03479	0.204	0.309	1.610	0.000	0.020	0.968	*fliP*	Motility	Flagellar Assembly
RCAP_rcc03480	-0.164	0.555	2.836	0.000	0.379	0.241	*fliN*	Motility	Flagellar Assembly
RCAP_rcc03481	-0.259	0.308	3.448	0.000	0.057	0.880	*fliH*	Motility	Flagellar Assembly
RCAP_rcc03482	-0.246	0.269	3.313	0.000	0.169	0.579	*fliF*	Motility	Flagellar Assembly
RCAP_rcc03483	-0.382	0.162	3.787	0.000	0.420	0.167	*fliL1*	Motility	Flagellar Assembly
RCAP_rcc03484	-0.133	0.609	2.860	0.000	0.131	0.682	*hypothetical protein*	Unknown	Unknown
RCAP_rcc03485	-0.055	0.832	3.412	0.000	0.069	0.886	*hypothetical protein*	Unknown	Unknown
RCAP_rcc03486	0.105	0.691	3.419	0.000	0.091	0.825	*motA*	Motility	Chemotaxis
RCAP_rcc03487	-0.542	0.021	3.301	0.000	0.426	0.053	*hypothetical protein*	Unknown	Unknown
RCAP_rcc03488	-0.248	0.294	0.187	0.363	0.089	0.710	*hemS*	Metabolism of Cofactors, Coenzymes and Vitamins	Heme Biosynthesis
RCAP_rcc03489	-0.214	0.238	0.362	0.020	-0.019	0.953	*hemN3*	Metabolism of Cofactors, Coenzymes and Vitamins	Heme Biosynthesis
RCAP_rcc03490	0.495	0.027	0.289	0.032	-0.243	0.488	*endoribonuclease, L-PSP family*	Unknown	Unknown
RCAP_rcc03491	-0.001	0.997	0.329	0.003	0.021	0.935	*nucleoside-triphosphatase*	Metabolism of Cofactors, Coenzymes and Vitamins	Thiamine metabolism
RCAP_rcc03492	0.396	0.079	0.225	0.157	-0.157	0.590	*rph*	Translation, ribosomal structure and biogenesis	Unknown
RCAP_rcc03493	0.211	0.232	0.110	0.473	-0.224	0.289	*hrcA*	Stress Response	Unknown
RCAP_rcc03494	0.138	0.503	0.305	0.049	-0.072	0.805	*grpE*	Post-translational Modification, Assembly and Chaperones	Unknown
RCAP_rcc03495	0.063	0.749	0.368	0.001	-0.109	0.520	*mutS*	Replication, Recombination and Repair	Repair
RCAP_rcc03496	0.341	0.039	-0.093	0.627	-0.194	0.492	*maeB2*	Energy Metabolism	Carbon fixation in photosynthetic organisms
RCAP_rcc03497	-0.008	0.969	-0.353	0.016	0.082	0.730	*rbsK*	Carbohydrate Metabolism	Pentose phosphate pathway
RCAP_rcc03498	0.031	0.908	0.186	0.364	-0.192	0.429	*phoB*	Signal Transduction	Transcription Regulator
RCAP_rcc03499	0.015	0.957	0.202	0.359	-0.082	0.787	*phoU*	Signal Transduction	Transcription Regulator
RCAP_rcc03500	0.173	0.501	0.160	0.538	-0.119	0.610	*pstB*	Metal and Ion Transport	Unknown
RCAP_rcc03501	-0.308	0.192	0.137	0.664	0.159	0.350	*pstA*	Metal and Ion Transport	Unknown
RCAP_rcc03502	-0.124	0.670	0.113	0.753	0.095	0.566	*pstC*	Metal and Ion Transport	Unknown
RCAP_rcc03503	0.283	0.272	0.561	0.079	-0.243	0.290	*pstS*	Metal and Ion Transport	Unknown
RCAP_rcc03504	-0.433	0.167	-0.253	0.449	-0.077	0.887	*cbiX*	Metabolism of Cofactors, Coenzymes and Vitamins	Cobalamin Biosynthesis

RCAP_rcc03505	0.010	0.969	0.006	0.974	-0.199	0.476	*hypothetical protein*	Unknown	Unknown
RCAP_rcc03506	-0.638	0.021	-0.044	0.801	0.459	0.109	*YCII-related domain-containing protein*	Unknown	Unknown
RCAP_rcc03507	-0.416	0.082	0.263	0.075	0.263	0.418	*gpsA*	Lipid Metabolism	Glycerophospholipid metabolism
RCAP_rcc03508	-0.295	0.345	0.325	0.136	0.239	0.506	*gcp*	Post-translational Modification, Assembly and Chaperones	Peptidase
RCAP_rcc03509	-0.288	0.393	-0.219	0.437	0.622	0.070	*hemD*	Metabolism of Cofactors, Coenzymes and Vitamins	Heme Biosynthesis
RCAP_rcc03510	-0.463	0.157	0.406	0.072	0.355	0.195	*hypothetical protein*	Unknown	Unknown
RCAP_rcc03511	0.133	0.449	0.423	0.006	0.060	0.693	*HemY domain-containing protein*	Metabolism of Cofactors, Coenzymes and Vitamins	Heme Biosynthesis
RCAP_rcc03512	0.268	0.224	2.597	0.000	0.005	0.991	*fliL2*	Motility	Flagellar Assembly
RCAP_rcc03513	-0.144	0.466	3.422	0.000	0.486	0.027	*flgH*	Motility	Flagellar Assembly
RCAP_rcc03514	-0.858	0.009	3.468	0.000	0.925	0.003	*flgA*	Motility	Flagellar Assembly
RCAP_rcc03515	0.003	0.991	3.618	0.000	0.125	0.731	*flgG*	Motility	Flagellar Assembly
RCAP_rcc03516	0.012	0.956	3.564	0.000	0.163	0.670	*flgF*	Motility	Flagellar Assembly
RCAP_rcc03517	0.290	0.148	3.393	0.000	-0.027	0.964	*fliQ*	Motility	Flagellar Assembly
RCAP_rcc03518	-0.194	0.359	3.205	0.000	0.120	0.782	*fliE*	Motility	Flagellar Assembly
RCAP_rcc03519	0.388	0.078	3.302	0.000	0.027	0.964	*flgC*	Motility	Flagellar Assembly
RCAP_rcc03520	-0.139	0.647	3.403	0.000	0.242	0.504	*flgB*	Motility	Flagellar Assembly
RCAP_rcc03521	-0.688	0.029	2.716	0.000	0.539	0.027	*fliI*	Motility	Flagellar Assembly
RCAP_rcc03522	-0.205	0.504	3.974	0.000	0.195	0.540	*flagellar protein*	Motility	Flagellar Assembly
RCAP_rcc03523	-0.531	0.078	4.475	0.000	0.384	0.171	*flbT*	Motility	Flagellar Assembly
RCAP_rcc03524	0.011	0.973	4.137	0.000	0.127	0.750	*flagellar FlaF family protein*	Motility	Flagellar Assembly
RCAP_rcc03525	0.003	0.995	3.364	0.000	0.200	0.556	*flaA*	Motility	Flagellar Assembly
RCAP_rcc03526	-0.088	0.747	2.587	0.000	0.194	0.591	*hypothetical protein*	Unknown	Unknown
RCAP_rcc03527	0.357	0.156	2.322	0.000	0.012	0.983	*flgJ*	Motility	Flagellar Assembly
RCAP_rcc03528	-0.594	0.041	3.200	0.000	0.701	0.000	*hypothetical protein*	Unknown	Unknown
RCAP_rcc03529	0.271	0.271	2.429	0.000	0.004	0.991	*flgD*	Motility	Flagellar Assembly
RCAP_rcc03530	-0.030	0.880	0.773	0.000	0.066	0.727	*ubiB*	Energy Metabolism	Biosynthesis of Ubiquinone
RCAP_rcc03531	0.544	0.003	0.969	0.000	-0.306	0.323	*ubiE*	Energy Metabolism	Biosynthesis of Ubiquinone
RCAP_rcc03532	0.070	0.806	0.148	0.533	0.033	0.927	*mutM*	Replication, Recombination and Repair	Unknown
RCAP_rcc03533	0.054	0.872	-0.294	0.137	0.035	0.914	*paaF*	Xenobiotics Biodegradation and Metabolism	Caprolactam degradation
RCAP_rcc03534	-0.115	0.747	0.480	0.000	0.329	0.157	*rpsT*	Translation, ribosomal structure and biogenesis	Unknown

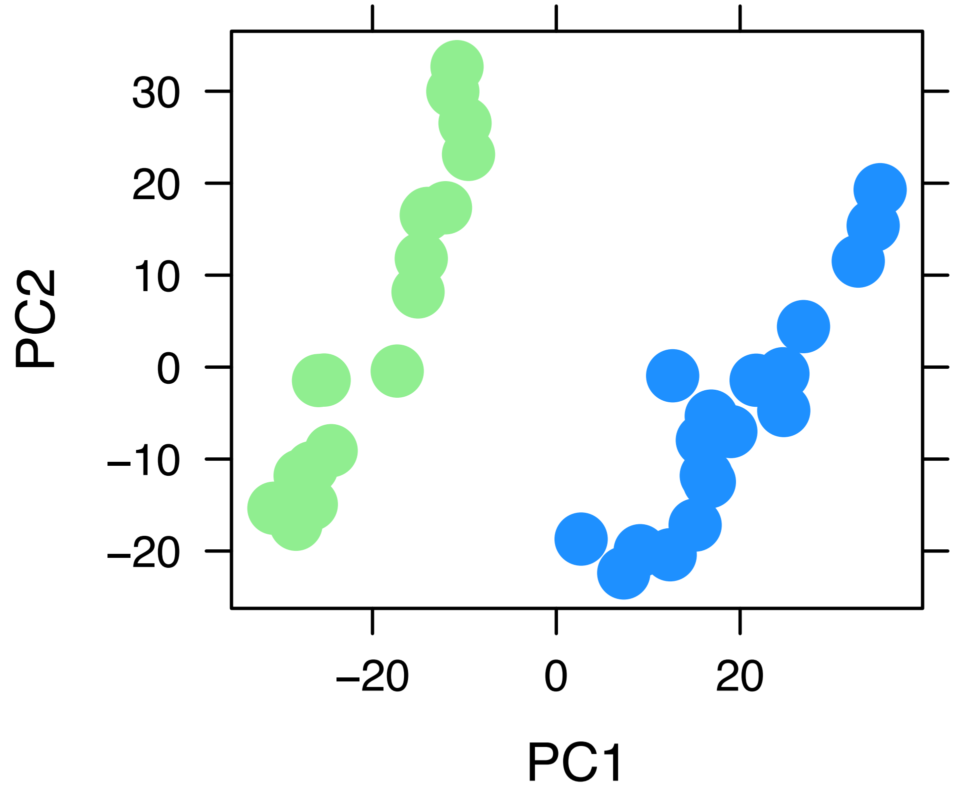

Figure S1. **Principle Component Analysis of Photosynthetic and Aerobic Replicates.** Blue dots show photosynthetic replicates (18)**,** green dots show aerobic replicates (18).

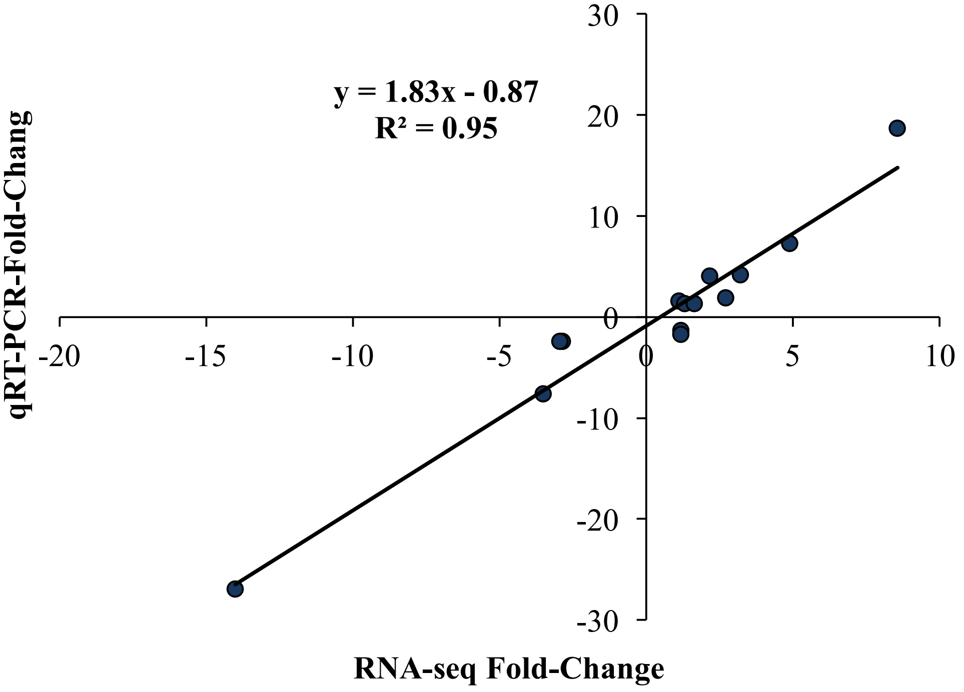

Figure S2. **Validation of RNA-seq by qRT-PCR and their fold-change correlation**. 14 genes were chosen which displayed positive and negative fold differences based on RNA-seq data. See Table S1for list of genes.

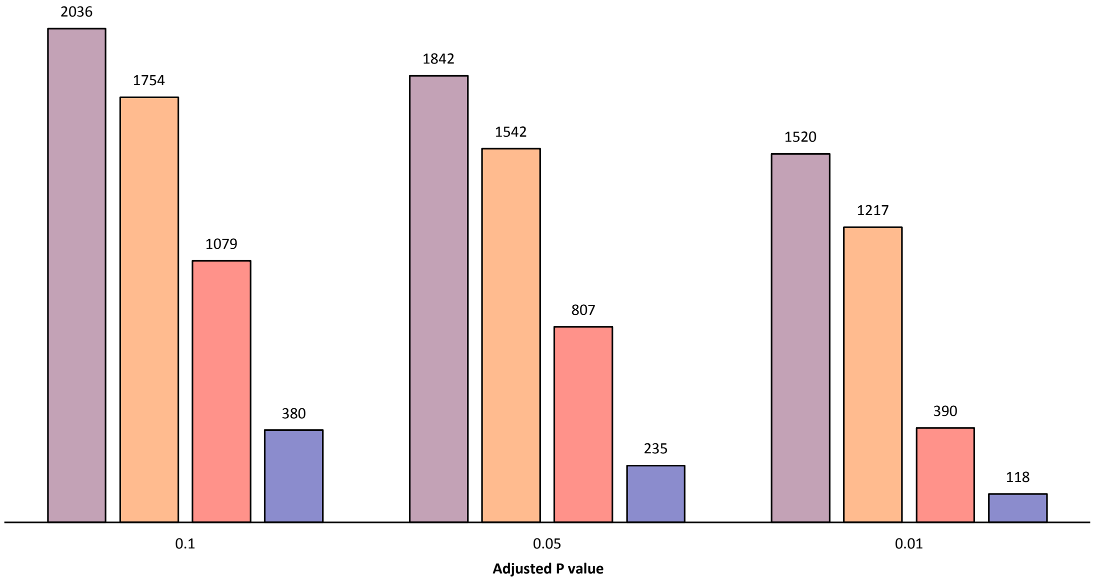

Figure S3. **Number of differentially expressed genes as a function of different p-value cutoffs.** Adjusted p values for aerobic/photosynthetic comparison of wild type cells (purple bar), relative to aerobic/photosynthetic comparison of cells disrupted for the redox responding regulators *regA* vs WT (orange), disruption of *fnrL* vs WT (red), disruption of *crtJ* vs WT (blue).

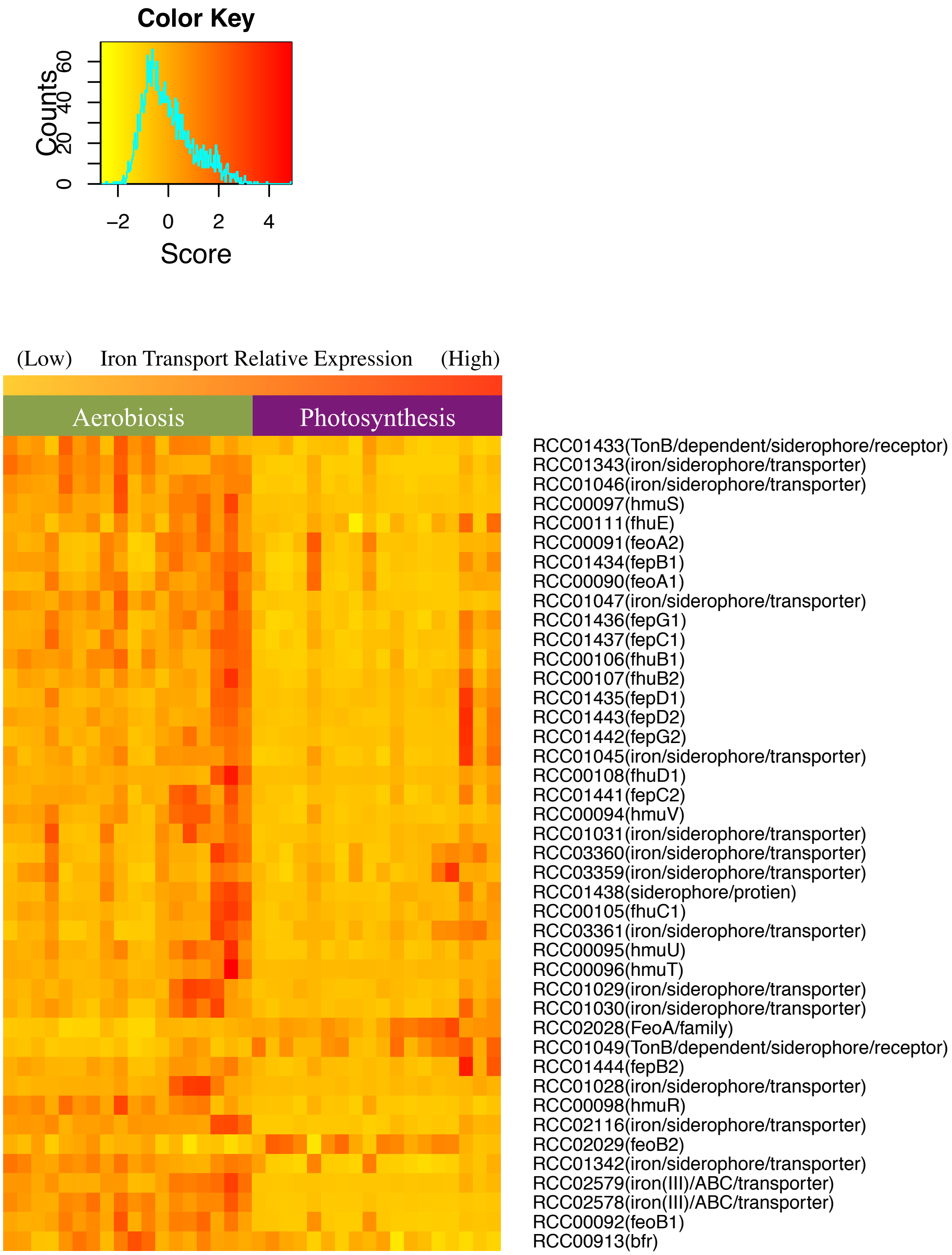

Figure S4. **Heat map of relative expression profiles of iron transport genes during aerobiosis and photosynthesis**. Expression levels are normalized per row and represent relative differences for a particular gene. For actual mRNA expression values see Table S5. These genes have been annotated using eggNOG serverhttp://eggnog.embl.de/version_3.0/.

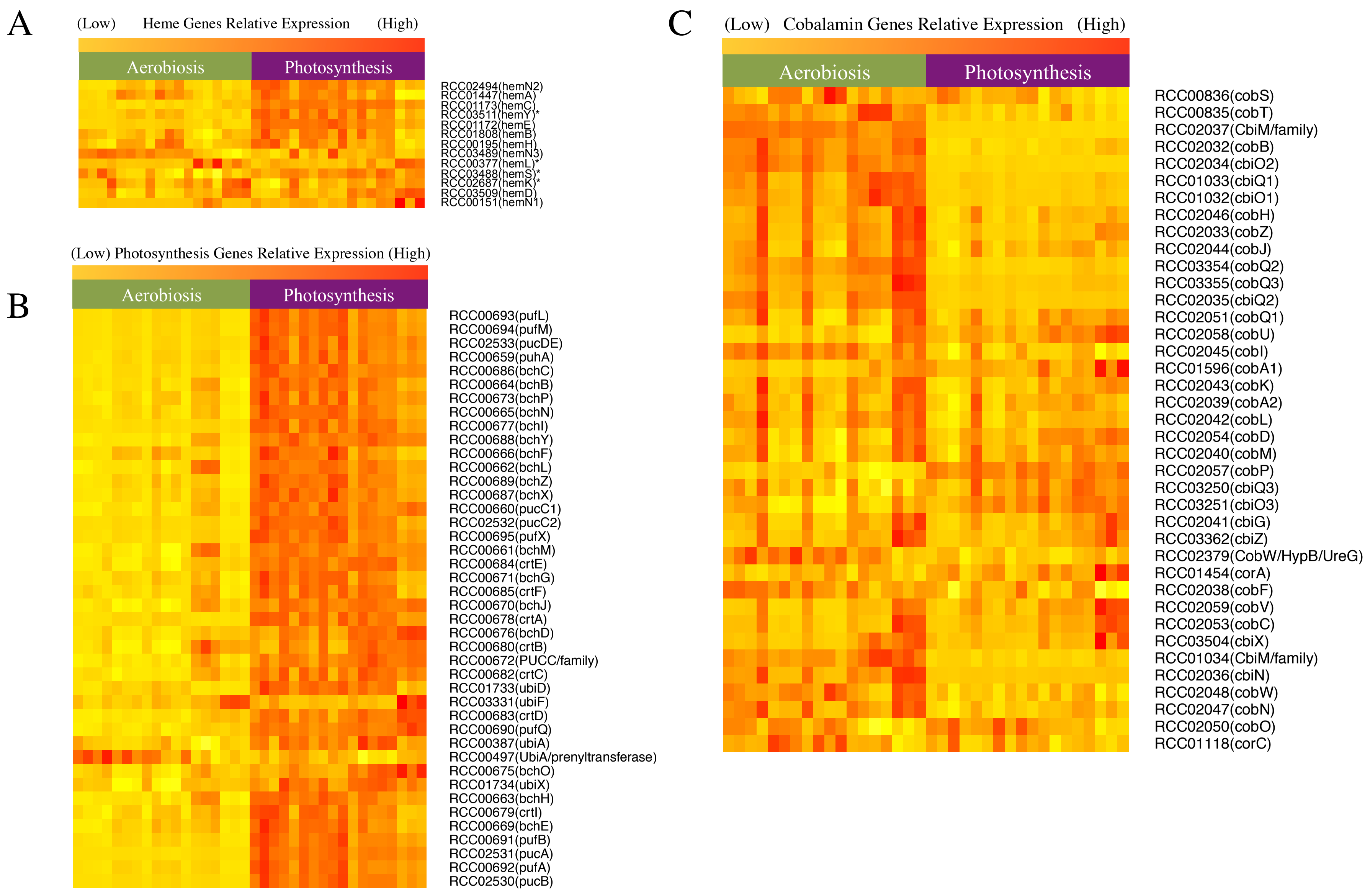

Figure S5. **Heatmap of relative expression profiles of tetrapyrrole biosynthesis genes during aerobiosis and photosynthesis**. (A) heat map of heme genes, (B) heat map of bacteriochlorophyll biosynthesis genes, (C) heat map of cobalamin biosynthesis genes. Asterisks denote genes annotated as *hem* genes that have indeterminate function.
